# Catalytic Enantioselective Synthesis of 1,4-(Hetero) Dicarbonyl Compounds through α-Carbonyl Umpolung

**DOI:** 10.1021/jacs.4c14826

**Published:** 2024-12-30

**Authors:** Till Friedmann, Karl Schuppe, Michael Laue, Ole Goldammer, Christoph Schneider

**Affiliations:** Institute of Organic Chemistry, University of Leipzig, 04103 Leipzig, Germany

## Abstract

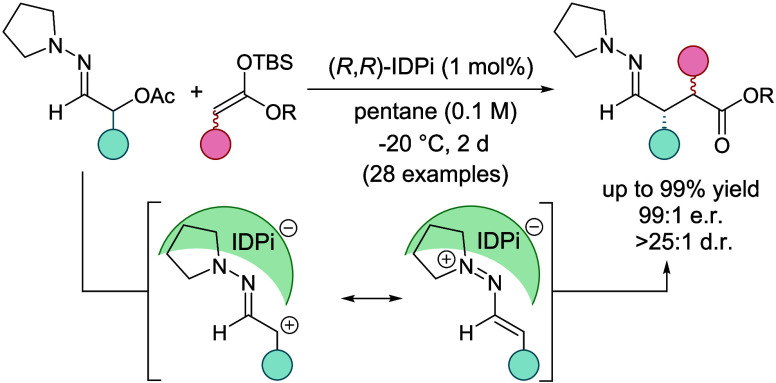

The enantioselective synthesis of 1,4-dicarbonyl compounds continues to pose a significant challenge in organic synthesis, and a catalytic process which generates two adjacent stereogenic centers with full stereochemical control is lacking until now. The 1,4-relationship of the functional groups requires an Umpolung strategy as one of the α-carbonyl positions has to be inverted into an electrophilic center to react with a normal enolate. We report herein the highly enantio- and diastereoselective addition of silyl ketene acetals toward electrophilic 1-azaallyl cations to furnish chiral 4-hydrazonoesters, which are masked 1,4-dicarbonyl compounds. The products carrying up to 2 new stereogenic centers were obtained in excellent yields across a broad substrate scope. As precursors to the 1-azaallyl cations, α-acetoxy hydrazones were employed and ionized with a strongly Lewis acidic, chiral silylium imidodiphosphorimidate (IDPi). The resulting ion pair was characterized with NMR and mass spectroscopy, while DFT calculations provided further insights into the reaction mechanism. In addition, the products were successfully converted into enantiomerically highly enriched b-cyano and b-formyl esters as well as γ-lactams and γ-amino acids, as demonstrated by syntheses of the anticonvulsant agent pregabalin and a brivaracetam precursor.

## Introduction

The 1,n-dicarbonyl motif is not only found in natural products and drug scaffolds,^1−5^ but more importantly is a central building block for further transformations in organic chemistry.^6−10^ As a result, their stereoselective assembly from two distinct carbonyl units ranges among the most valuable transformations in organic synthesis. Given the inherent polarity of the carbonyl group 1,3- and 1,5-dioxygenated compounds are readily accessible employing normal enolate chemistry.^11−14^ On the contrary, the synthesis of 1,4-dicarbonyl compounds is more challenging and requires an Umpolung strategy, meaning polarity inversion, of one of the reaction partners.^15−19^ Thus, the normally nucleophilic polarity of the carbonyl α-position is inverted into an electrophilic center in an enolonium-type compound that can be trapped with a regular enolate to forge the central C(2)–C(3)-bond in the most straightforward fashion.^20,21^

**Figure 1:**
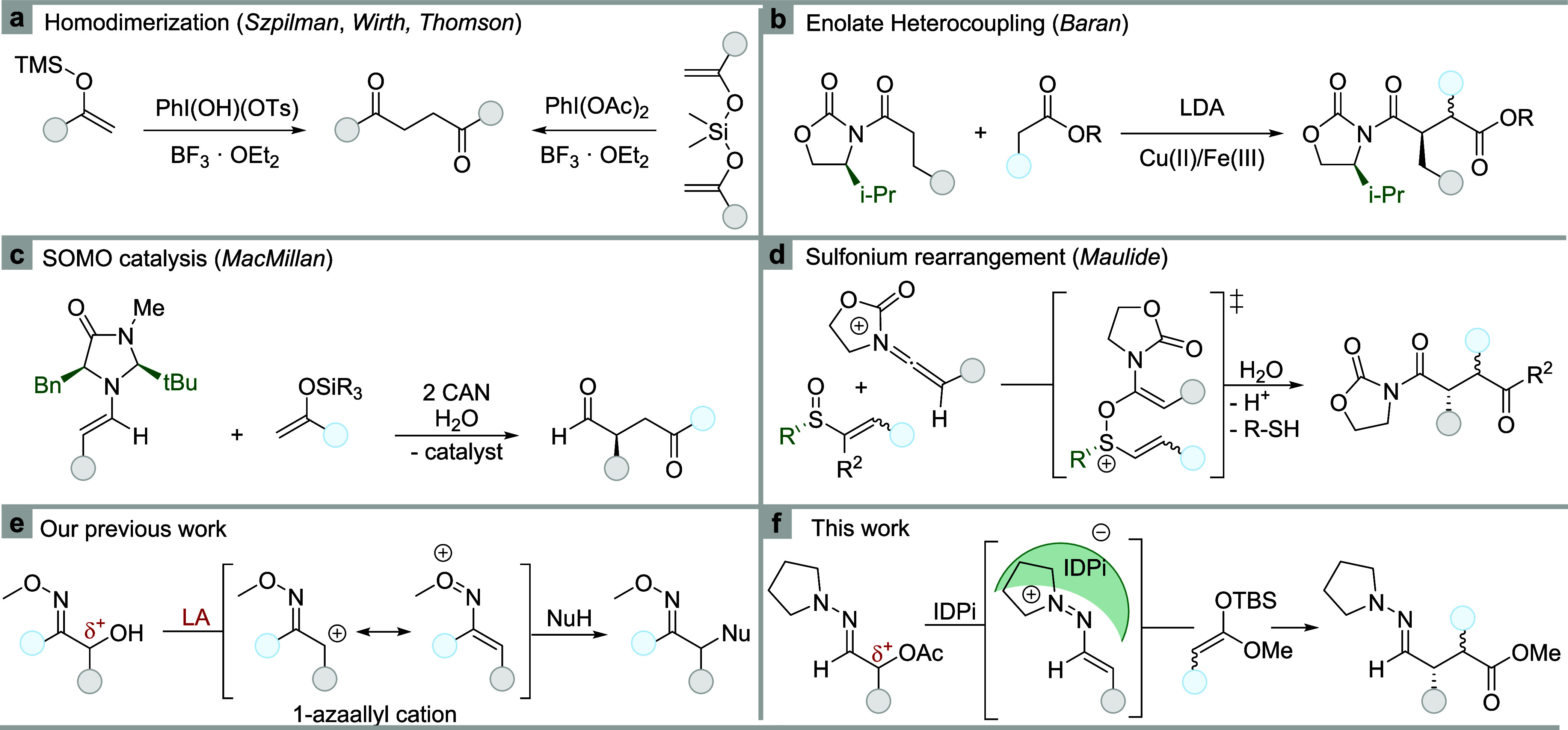
Outline of this study. (a) Homodimerization approach by Szpilman and Wirth. (b) Enolate heterocoupling by Baran et al. (c) SOMO catalysis by MacMillan and co-workers. (d) Sulfonium rearrangement by Maulide and co-workers. (e) Previous utilization of 1-azaallyl cations as umpoled synthons. (f) This work: catalytic, enantioselective 1,4-(hetero)dicarbonyl synthesis.

Seminal reports by the groups of Baran, Wirth, Szpilman, and Thomson focused on oxidative Umpolung strategies with metal oxidants and hypervalent iodine compounds, respectively, which often suffer from homocouplings, moderate yields, and low stereoselectivity, however (Figure 1a,b).^10,22−24^ Maulide and co-workers generated enolonium compounds from keteniminium ions and reacted them with enolates in a chemoselective, yet not stereoselective fashion.^25,26^ In a further very elegant development the Maulide group trapped keteniminium ions with a chiral vinyl sulfoxide to effect a charge-accelerated sulfonium [3,3]-sigmatropic rearrangement furnishing 2,3-disubstituted 1,4-dicarbonyl compounds with excellent stereocontrol (Figure 1d).^27,28^ This process currently constitutes the single highly stereoselective process for the synthesis of this product class. It suffers, however, from the stoichiometric use of a chiral reagent which unfortunately cannot be recycled. Finally, the concept of SOMO catalysis pioneered by MacMillan et al. remains the sole other catalytic, enantioselective 1,4-dicarbonyl synthesis to date based upon Umpolung of the carbonyl α-position (Figure 1c).^29,30^ However, the synthesis of 2,3-disubstituted 1,4-dicarbonyl motifs has not been reported using this strategy until now and the precise adjustment of redox potentials limits the substrate flexibility.

In previous work we have established a broad range of nucleophilic additions toward 1-azaallyl cation intermediates derived from α-hydroxy oxime ethers with (hetero)aromatic compounds and b-ketoesters (Figure 1e).^31−34^ The mechanistic basis of this chemistry is the electrophilic nature of the transient 1-azaallyl cation formed by Lewis acid-catalyzed dehydration of the α-hydroxy oxime ether, which is reversed to the normally nucleophilic polarity of the carbonyl α-position.

## Results and Discussion

Building on this precedence, we now report a catalytic, highly enantioselective addition of silyl ketene acetals to 1-azaallyl cations giving rise to valuable 4-hydrazonoesters with up to 2 new stereogenic centers (Figure 1f). As 1-azaallyl cation precursors we employed α-acetoxy hydrazones in place of the α-hydroxy oxime ethers as we hypothesized that *N*,*N*-dialkyl hydrazones could stabilize the positive charge in the α position of the heterocarbonyl group even more effectively than oxime ethers. In fact, the Enders group had pursued this very approach with their covalently bound, chiral SAMP-hydrazones, but obtained only moderate diastereoselectivity in this reaction even with stoichiometric amounts of a Lewis acid.^35^ As catalytic Lewis acid for the envisioned enantioselective process we considered strongly acidic and confined silylium imidodiphosphorimidates (IDPi) ideal which the List group has introduced and pioneered in recent years.^36,37^ The silylium-IDPi was intended to ionize the starting α-acetoxy hydrazone into a chiral ion pair composed of an 1-azaallyl cation and a chiral anion along with the released silyl acetate. The chiral ion pair should then react to the product under regeneration of the silylium Lewis acid.

### Reaction Optimization

At the onset of this study, we investigated the reaction of ethyl-substituted α-acetoxy hydrazone **1a** and *tert*-butyldimethylsilyl (TBS) ketene acetal **2a** catalyzed by various IDPis **3** at low temperatures (Table 1). To address solubility issues and facilitate comparability, we initially explored this reaction in a solvent mixture of pentane and CH_2_Cl_2_ (1:1). After examining a wide range of BINOL substitution patterns (Supporting Information Figures 2–4), we discovered that meta-substituted IDPi catalysts, such as **3a** and **3e**, exhibited promising levels of enantioselectivity, with larger *meta*-aryl groups providing superior results. Modification of the sulfonamide moiety to larger perfluorinated groups (**3b**–**d**) resulted in lower enantioselectivity.^38−40^

**Table 1:**
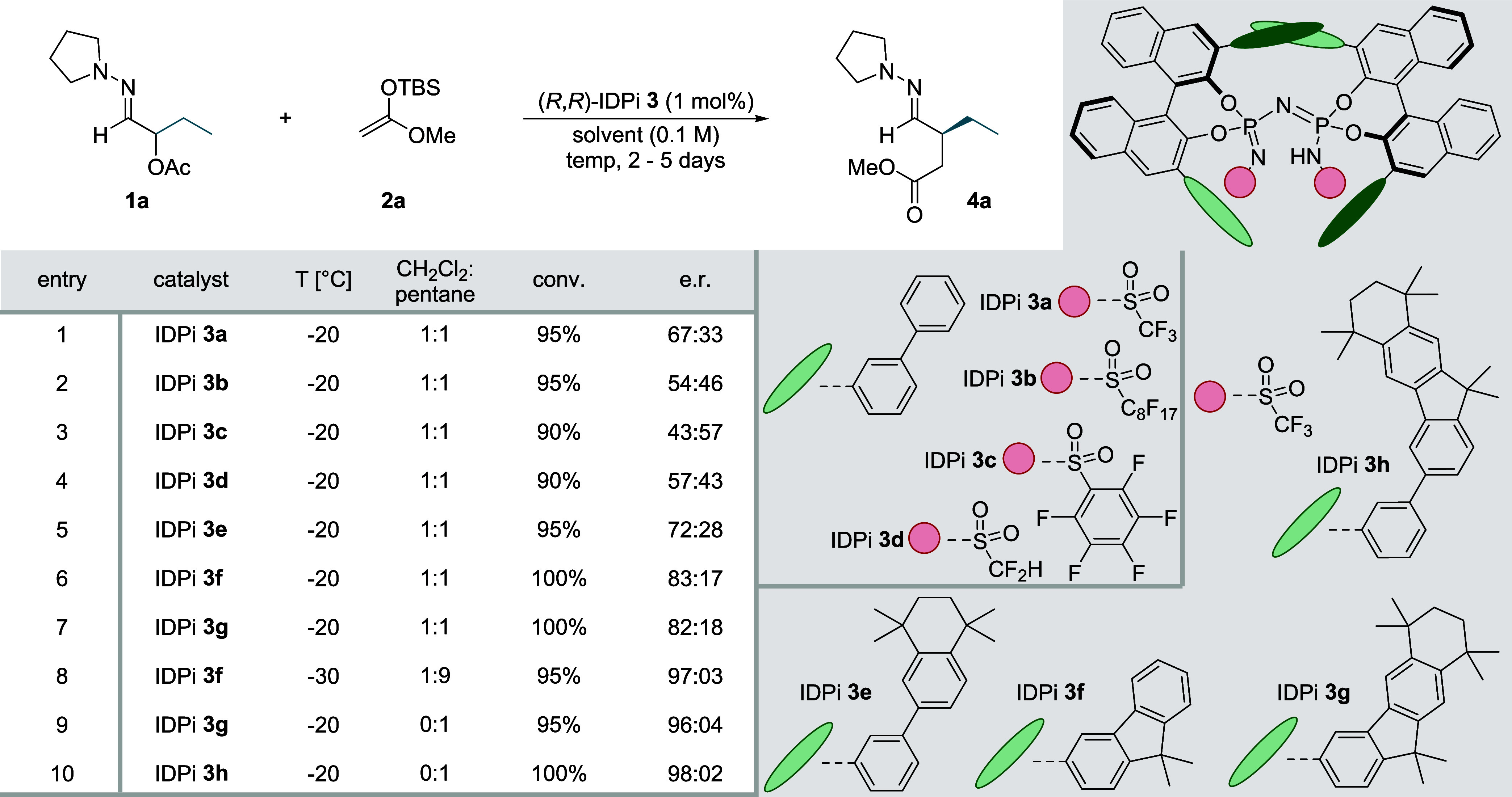
Reaction Optimization^a^

a Reactions were conducted on a 0.10 mmol scale, using 2.0 equiv (0.20 mmol) of **2a**. Conversions and enantiomeric ratios were determined by chiral HPLC of the crude reaction mixtures. HPLC method: Chiralpac IA, 99:1 hexanes/*iso*-propanol, 1 mL/min, 248 nm absorbance. See Supporting Information for further details.

**Figure 2:**
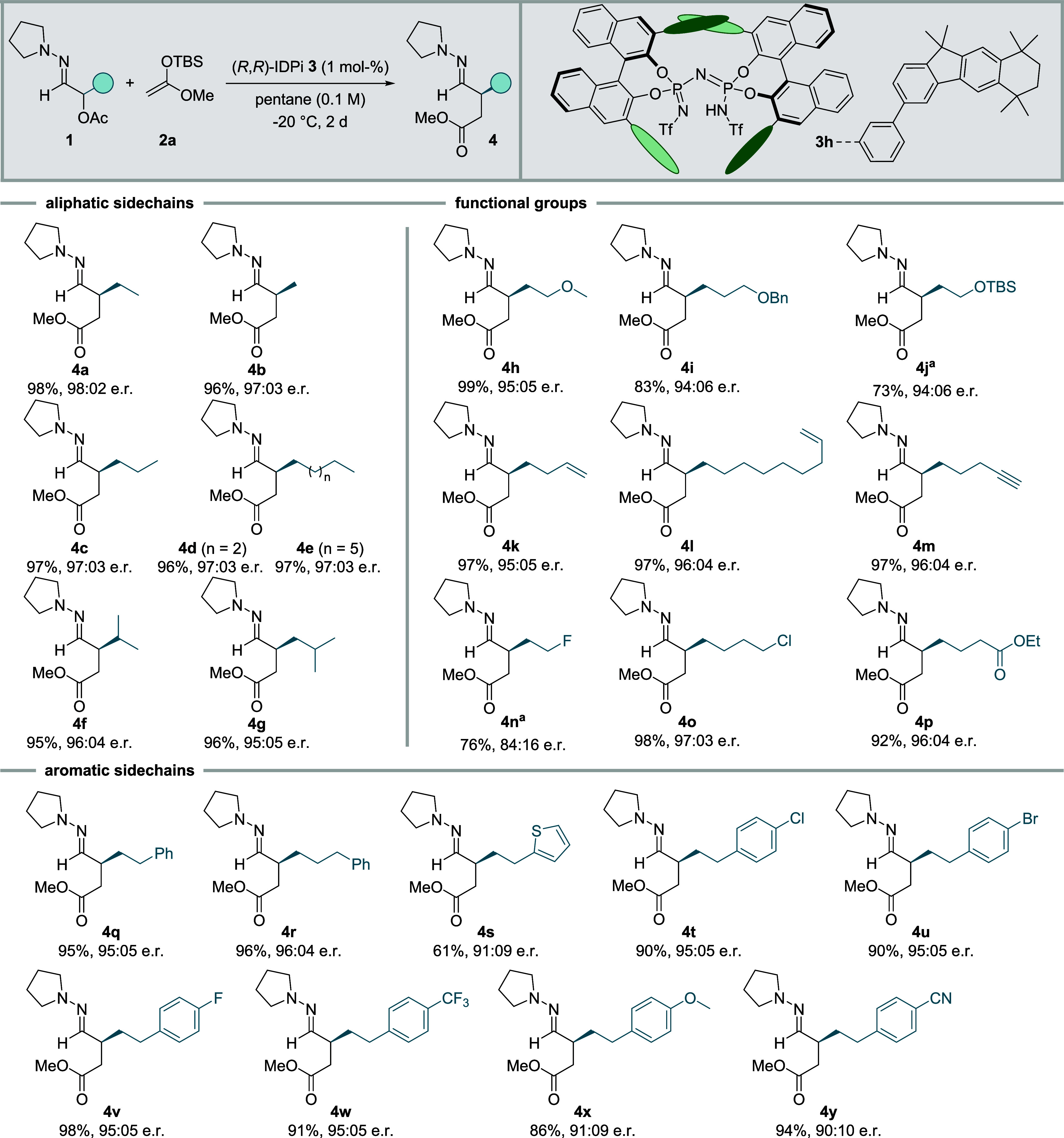
Substrate Scope. Reaction of hydrazones **1** with silyl ketene acetal **2a** using IDPi catalyst **3h** on a 0.20 mmol scale. Reactions were conducted at −20 °C for 2 days with 2.0 equiv of **2a**; isolated yields. ^a^IDPi **3g** was used for this reaction.

In further optimization studies we developed new 3-fluorenyl substituted IDPi catalysts (**3f**–**h**) combining a meta-aryl substituent and a large *para*-alkyl group at the same time. Using our established conditions, promising results were obtained, particularly with IDPi catalysts **3f** and **3g** (entries 6 and 7). On the basis of their increased solubility in hydrocarbon solvents a 1:9-solvent mixture of CH_2_Cl_2_/pentane or even pure pentane could be used resulting in excellent enantioselectivity for the model reaction (entries 8 and 9). As these catalysts covered only a limited substrate scope, a final round of optimization was conducted (Supporting Information Figures 5 and 6). To our delight, the less rigid IDPi **3h** emerged as the most versatile and selective catalyst (entry 10), which achieved complete conversion and excellent enantioselectivity across a much wider substrate scope and was therefore investigated in full (Figure 2).

### Substrate Scope

In general, the addition of silyl ketene acetal **2a** to α-acetoxy hydrazones **1** yielded the desired products **4a**–**g** in nearly perfect yields and with very high enantioselectivities exceeding 95:05 e. r. regardless of steric hindrance and chain length. Even the small methyl group in **4b** gave rise to excellent enantiofacial discrimination by the catalyst just as effective as observed with longer alkyl chains. Moreover, hydrazones with branched alkyl groups such as *iso*-propyl and *iso*-butyl furnished the corresponding products **4f** and **4g**, respectively, in high yield and enantioselectivity as well. Important precursors for two pharmaceuticals (**4c** and **4g**) were obtained with comparable enantioselectivity. The functional group tolerance was then explored by subjecting hydrazones carrying various Lewis-basic groups that could potentially inhibit the activity of the silylium ion catalyst. To our relieve, hydrazones containing different ether and ester substituents, **4h**–**j** and **4p**, respectively, were obtained with unaltered enantioselectivity and without any side product formation. When using terminal alkenes and alkynes, we observed nearly quantitative yields with e. r. values of up to 96:04. Aliphatic halides, specifically chloride **4o**, were well tolerated with results similar to those of pure aliphatic alkyl chains. However, a fluorine atom in the γ-position resulted in a diminished yield and selectivity of 84:16 e. r. in product **4n**. Regarding aromatic side chains, various substitutions were well tolerated without significant changes in selectivity (95:05 e. r.) compared to the homobenzylic substrate **4q**. However, polar aromatic groups in the substrates like anisole, benzonitrile, and thiophene led to poorer solubility in pentane and eventually diminished selectivity of only around 90:10 e. r. Thus, solubility issues when using pure pentane as solvent are likely the only limitation for obtaining high enantioselectivity. Besides products **4j** and **4n**, for which catalyst **3g** yielded better results, IDPi **3h** proved to be the most general and selective catalyst for the title reaction giving rise to excellent yields of over 90% and enantioselectivities exceeding 95:05 e. r.

**Figure 3:**
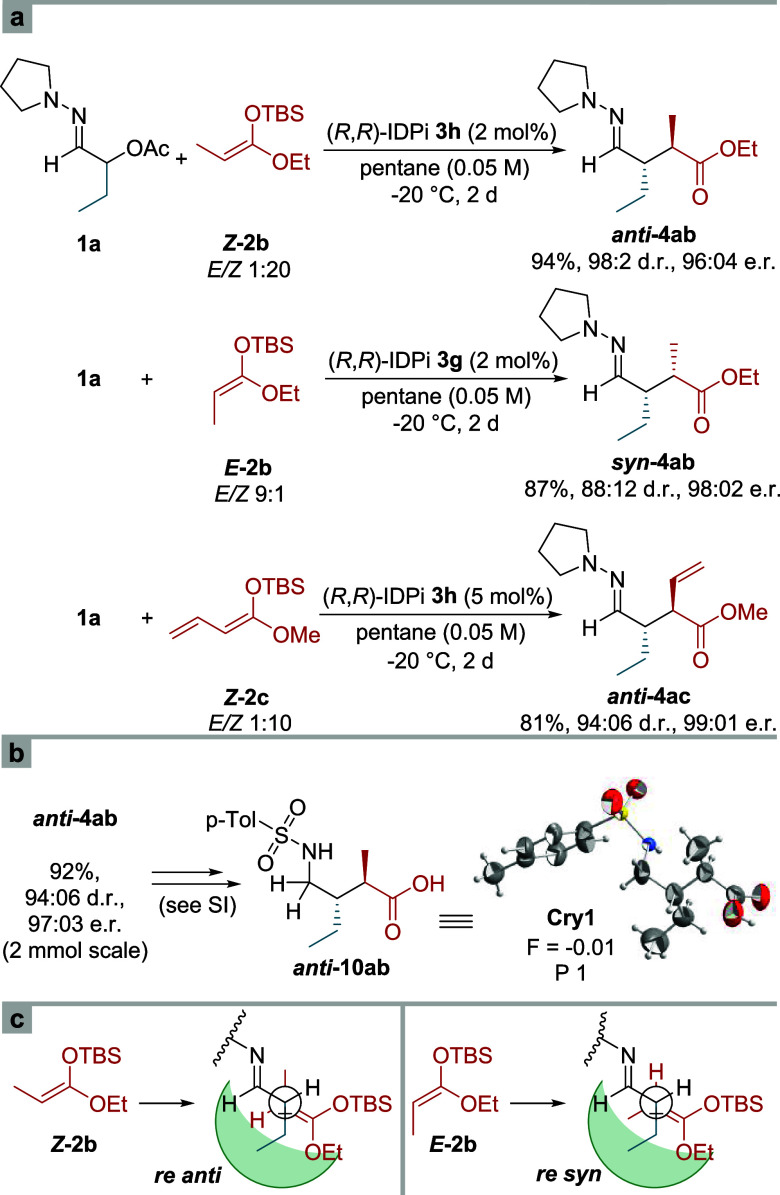
Diastereoselective Reactions. (a) Synthesis of 2,3-disubstituted 1,4-dicarbonyl compounds. (b) Crystal structure **Cry1** derived from *anti*-product **4ab**. (c) Newman projections of proposed transition state assemblies leading to *syn*- and *anti*-products.

A second stereogenic center can be installed in this process using a substituted and thus prostereogenic silyl ketene acetal as nucleophile (Figure 3a). Employing the ethyl propionate-derived silyl ketene acetal ***Z*****-2b** (1:20 *E*/*Z*) in the reaction with α-acetoxy hydrazone **1a** catalyzed by IDPi **3h**, we obtained *anti*-product ***anti*****-4ab** in nearly quantitative yield and with both excellent diastereoselectivity and enantioselectivity of 98:02 d. r. and 96:04 e. r., respectively. Its relative configuration was determined by X-ray crystal structure analysis of the corresponding sulfonamido acid ***anti*****-10ab** (Figure 3b). Interestingly, when the opposite *E*-configured silyl ketene acetal ***E*****-2b** (9:1 *E*/*Z*) was subjected to the reaction with hydrazone **1a** and IDPi catalyst **3g**, the *syn*-diastereomer ***syn*****-4ab** was isolated with 87% yield, a superb enantioselectivity of 98:02 e. r. and slightly diminished diastereoselectivity of 88:12 d. r. The erosion of diastereoselectivity corresponds to the less selective synthesis of silyl ketene acetal ***E*****-2b** which was employed as a 9:1-*E*/*Z*-mixture. This observation suggests that the reaction follows a strictly stereoconservative pathway with the starting ketene acetal configuration determining the configuration at the second stereogenic center. Thus, both product diastereomers can be stereoselectively accessed by choosing the appropriate nucleophile configuration.

When we employed vinyl silyl ketene acetal ***Z*****-2c** in the IDPi-catalyzed addition to α-acetoxy hydrazone **1a**, we observed the formation of a single regioisomeric α-addition product ***anti*****-4ac** which was obtained with good yield and excellent diastereo- and enantioselectivity. The stereochemical outcome of these three reactions can be explained by assuming the extended open transition state (TS) assemblies illustrated in Figure 3c with a transient, *E*-configured 1-azaallyl cation. The depicted transition state conformations which minimize 1,2-gauche interactions are in good agreement with the computed transition state Ts *re* shown in Figure 5b, with the large OTBS group pointing out of the narrow catalytic pocket (vide infra).

### Large-Scale Synthesis and Postmodifications

**Figure 4:**
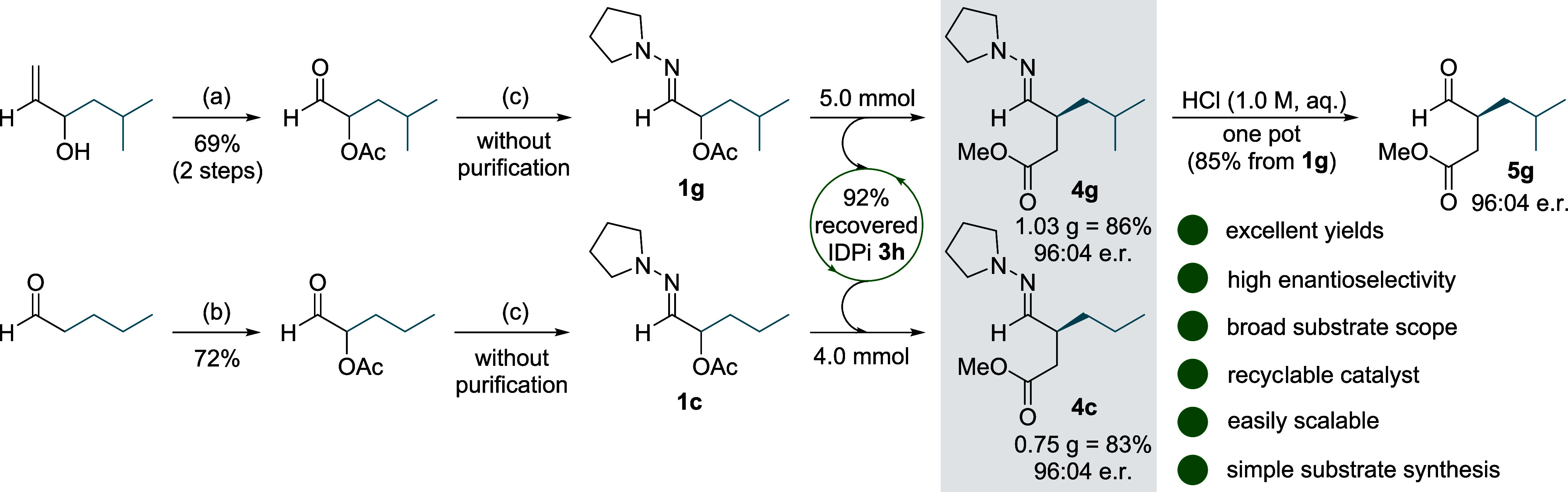
Large-scale experiments. Reactions were conducted on a 5.0 and 4.0 mmol scale using the optimized reaction conditions (Table 1). (a) Ac_2_O, DMAP, NEt_3_, CH_2_Cl_2_, 0 °C, 16 h. O_3_, NEt_3_, CH_2_Cl_2_, −78 °C, 45 min. (b) *t*-BuOOH, *n*-Bu_4_NI, AcOH, piperidine, EtOAc, 50 °C, 3 h. (c) *N*-aminopyrrolidine, MgSO_4_, Et_2_O, 0 °C, 5 h.

The practicality and scalability of this method was highlighted by the synthesis of hydrazones **4c** and **4g** on a gram scale (Figure 4). Although a slight decrease in yield was noted, the enantioselectivity did not suffer, and the products were obtained with almost identical enantioselectivity as in the small scale experiments (96:04 e. r.). The starting α-acetoxy hydrazones **1c** and **1g** can be readily obtained within 2–3 steps from two inexpensive and commercially available building blocks. In particular, the hydrazone condensation reaction of the α-acetoxy aldehydes proceeded in quantitative yield and required no further purification. For economic reasons, IDPi catalyst **3h** was recovered after the reaction and was directly used for the second gram-scale experiment without any loss of catalytic activity. Interestingly, the acidic hydrolysis of hydrazone **4g** could be conducted simply by addition of aqueous hydrochloric acid (1 M) to furnish 4-oxoester **5g** with 85% overall yield and 96:04 e. r. in a one pot operation. This provides direct access to enantiomerically enriched 1,4-dicarbonyl compounds from α-oxidized aldehydes with only one final purification step.

**Figure 5:**
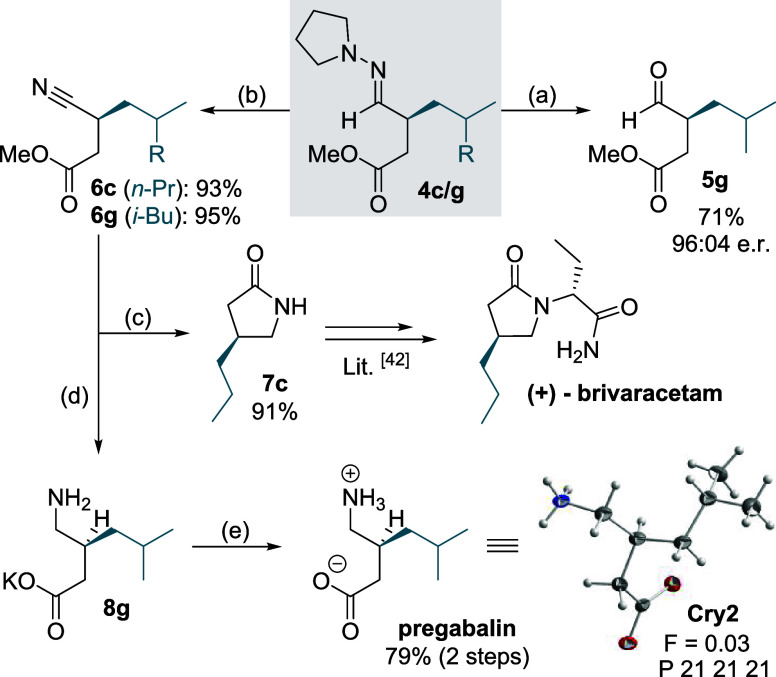
Follow-up chemistry. (a) O_3_, CH_2_Cl_2_, −78 °C, 10 min; (b) MMPP, H_2_O/MeOH, pH 7, 0 °C, 2 h; (c) Raney-Ni, MeOH, 70 °C, 16 h; (d) Raney-Ni, KOH, EtOH/H_2_O, rt, 16 h; (e) AcOH, EtOH, 70 °C, 30 min.

The significance of this Umpolung strategy was demonstrated by the transformation of **4c**/**g** into medicinally important drug molecules (Figure 5).^41^ While aldehydes such as **5g** can be obtained racemization-free in good yields by simple acidic workup (Figure 4) or ozonolysis (Figure 5), the 4-hydrazonoesters can as well be oxidized into the corresponding nitriles with magnesium monoperoxyphthalate (MMPP) under mild conditions. Thus, **4c** and **4g** were converted into nitriles **6c** and **6g**, respectively, with almost quantitative yields.^7^ With Raney-Nickel, nitrile **6c** was then directly converted into γ-lactam **7c** in good yield, which is a common precursor for the antiepileptic drug brivaracetam.^7,42^ With a slightly modified protocol, we obtained the enantiomerically highly enriched potassium salt of γ-amino acid **8g**.^4^ The importance of this substrate class is exemplified by the conversion into pregabalin, one of the most widely prescribed anticonvulsant agents globally. Furthermore, the X-ray crystal structure analysis of pregabalin confirmed the stereogenic center to be (*S*)-configured, which is in accordance with the specific rotation value measured for our material [α]_D_^25^ = +10.0° (c 1.0, H_2_O) and that reported in the literature [α]_D_^25^ = +10.1° (c 1.1, H_2_O).^43−46^

### Mechanistic Investigations

**Figure 6:**
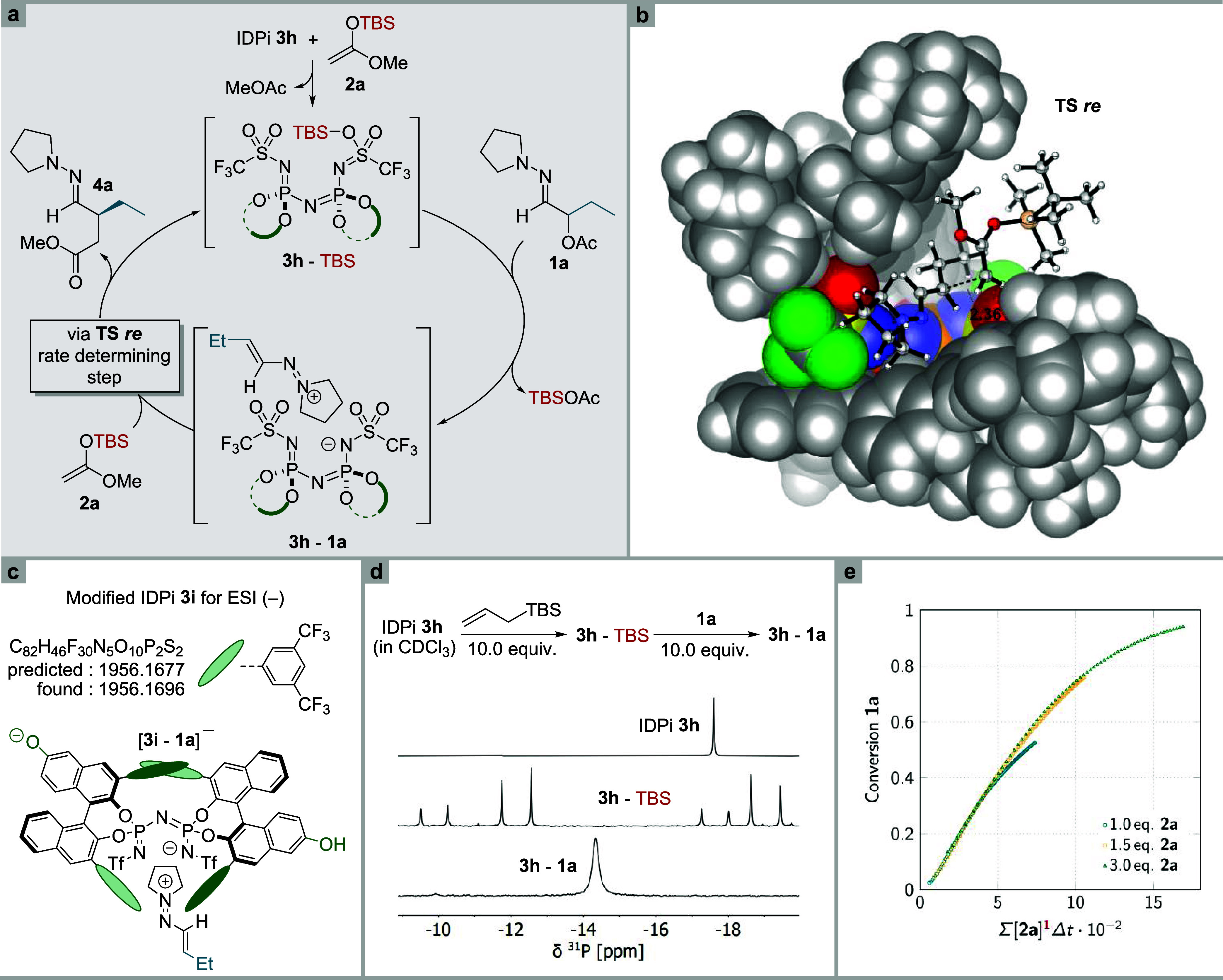
Mechanistic investigations. (a) Proposed catalytic cycle. (b) Transition state modulated by DFT methods. (c) ESI (−) MS characterization of **3i**–**1a**. (d) Characterization of intermediates by ^31^P NMR. (e) Burés plot revealing 1. order kinetics with regard to nucleophile **2a**.

To gain insight into the reaction mechanism, we initially investigated the formation of the active silylium catalyst using ^31^P NMR-spectroscopy (Figure 6d).^38^ We treated IDPi **3h** with an excess of allyl(*tert*-butyl) dimethylsilane which effected protodesilylation and caused the distinct singlet of **3h** to split into four sets of doublets due to coordination of the silylium ion to the IDPi anion (Figure 6a, 3**3h**-TBS). The different intensities suggest a preferential binding to one side of the IDPi center. Upon adding an excess of α-acetoxy hydrazone **1a** the signals merged to a distinct singlet at −14.2 ppm, indicating the formation of a new species which is presumed to be ion pair **3h**–**1a**. Monitoring the reaction progress via ^31^P NMR-spectroscopy over 14 h revealed a single, constant signal at −14.5 ppm, which we attribute to ion pair **3h**–**1a** as the catalyst resting state (Supporting Information Figure 11). The accurate characterization of a corresponding ion pair by (−)ESI-MS was enabled by the use of a modified IDPi **3i** (Figure 5c), containing additional phenolic groups in the remote 6,6′-positions of the BINOL-skeleton, under standard reaction conditions (Figure 6c).

Furthermore, we monitored the reaction progress with ^1^H NMR-spectroscopy under slightly modified conditions. The reaction was performed in a 1:1 mixture of pentane-d_12_:CD_2_Cl_2_ at −5 °C to ensure complete solubility of all intermediates. We analyzed the reactions with varying equivalents of silyl ketene acetal **2a** through variable time normalization analysis, using kinetic data obtained from the ^1^H NMR experiments. Following the procedure described by the Burés group, we observed a good alignment with first-order kinetics regarding the silyl nucleophile (Figure 6e, Supporting Information Figures 12 and 13).^47^

The proposed mechanism, illustrated in Figure 6a, is supported by the characterization of intermediates and kinetic investigations. The reaction is initiated through protodesilylation of ketene acetals **2** by the highly acidic IDPi catalyst to produce **3**-TBS as the active species. Upon addition of α-acetoxy hydrazones **1**, the silylium Lewis acid abstracts the acetate from **1** and generates the 1-azaallyl cation. This highly electrophilic cation is embedded within the catalytic pocket of the IDPi anion, described as intermediate **3**–**1**. The confined active site of the IDPi enables efficient enantiodiscrimination during nucleophilic attack of the silyl ketene acetals **2**. Finally, upon release of the 4-hydrazonoesters **4** the active silylium Lewis acid is regenerated and the catalytic cycle is closed.

Taking all mechanistic observations into account, we propose a defined ion pair **3**–**1** as the resting state of this reaction. Nucleophilic attack of **2** onto this ion pair then represents the rate-determining step of the catalytic cycle. These findings also explain the reduced yields and selectivities for products **4s**, **4x** and **4y**, where precipitation was observed following the addition of the hydrazones.

Finally, computational studies on the reaction of silyl ketene acetal **2a** with hydrazone **1a** catalyzed by IDPi **3h** were performed. Considering the complexity of the transformation and the enormous size of the structures that are required to be evaluated, we limited our computational analysis to the stereodetermining step that follows the formation of the 1-azaallyl cation while also employing an ONIOM-approach (PBE-D3BJ/def2-SVP: GFN2-xTB) as the calculation method.^48,49^ This type of calculation completely relies on quantum electronic structure methods rather than on empirical force fields and was shown to give reliable results for larger systems like IDPis without the necessity of significantly simplifying any structures of interest. A manual conformational search has been performed on possible catalyst substrate orientations that were subsequently optimized at the ONIOM(PBE-D3BJ/def2-SVP: GFN2-xTB, gas phase) level of theory. Transition states were located by employing the Climbing Image Nudged Elastic Band (NEB-CI) method starting from relaxed ground state structures of the reactants followed by a TS-optimization and frequency analysis at the same level of theory (see the Supporting Information). Finally, the free energy was further refined using a perturbatively corrected double hybrid DFT method for the higher quantum mechanical region in combination with the ALPB solvation model for the complete system and *n*-hexane as solvent (referred to as ONIOM(B2PLYP-D3BJ/def2-TZVP(ALPB:*n*-hexane):GFN2-xTB)).^50,51^

These calculations propose a preferential *re*-face attack in the depicted TS-model (Figure 6b) which is energetically favored by 1.9 kcal/mol compared to the corresponding *si*-face attack. This energetic difference corresponds to a calculated e. r. of 98:2 and is in good agreement with the experimentally observed enantiomeric ratio.

**Figure 7:**
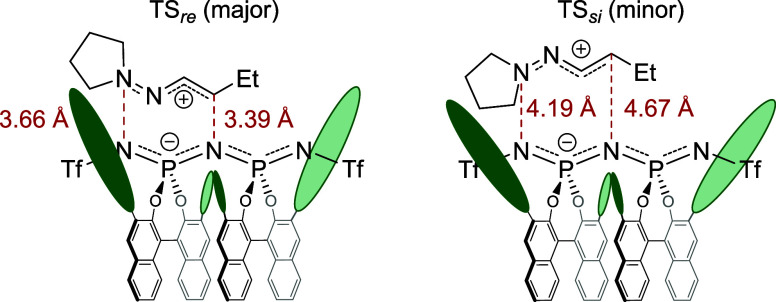
Transition states. Anion–cation distance in TS *re* (left) TS *si* (right).

The calculations also reveal a shorter distance in TS *re* (major) between the W-shaped 1-azaallyl cation and the IDPi anion forming a tighter ion pair (Figure 7). In TS *si*, leading to the minor enantiomer, steric repulsion between the pyrrolidine of the M-shaped cation and the BINOL backbone of the anion leads to a more separated ion pair and widening of the catalytic pocket.

## Conclusions

We have developed the first catalytic, enantio- and diastereoselective synthesis of 1,4-(hetero)dicarbonyl products utilizing a novel Umpolung strategy. As central intermediate a chiral ion pair composed of an 1-azaallyl cation and an IDPI anion is attacked by a silyl ketene acetal to furnish 4-hydrazonoesters with generally >90% yield, excellent enantioselectivity of up to 99:01 e. r. and very high diastereoselectivity of up to 50:1 d. r. Mechanistic studies were conducted with NMR and MS spectroscopy which confirmed the existence of the chiral ion pair and its conversion to the product as the rate-determining step of the catalytic cycle. Possible transition states responsible for the high enantiofacial discrimination were modeled with DFT and revealed good agreement with the experimental results. The use of prostereogenic, propionate-based silyl ketene acetals enabled access to products with 2 adjacent stereogenic centers. The corresponding *syn*- and *anti*-diastereomers were isolated with good diastereoselectivity depending upon the choice of the nucleophile configuration. Furthermore, the 4-hydrazonoesters thus obtained hold significant potential for further manipulations. Enantiomerically highly enriched β-cyano and β-formyl esters as well as γ-lactams and γ-amino acids were easily accessed, including the anticonvulsant agents pregabalin and brivaracetam. These examples demonstrate the high potential of this reaction for the synthesis of various 1,4-dicarbonyl compounds with excellent stereochemical control over two adjacent stereogenic centers.

## Supporting Information Available

Additional experimental details NMR spectra of all compounds, HPLC chromatograms, crystallographic details, and details on DFT calculations (PDF)

## Abbreviations

IDPi: imidodiphosphorimidate
TBS: *tert*-butyldimethylsilyl
MMPP: magnesium monoperoxyphthalate
TS: transition state
