# Supplementary material for: Catalytic Enantioselective Synthesis of 1,4-(Hetero) Dicarbonyl Compounds through α-Carbonyl Umpolung
Source: J Am Chem Soc. 2024 Dec 30;147(2):1948–56. doi: 10.1021/jacs.4c14826 (PMC11744765; doi:10.1021/jacs.4c14826)
Supplement: Supplementary file 1 — ja4c14826_si_001.pdf [file ja4c14826_si_001.pdf]

# Supplementary Information

## Catalytic Enantioselective Synthesis of 1,4-(Hetero) Dicarbonyl Compounds through *α*-Carbonyl Umpolung

Till Friedmann,[1] Karl Schuppe,[1] Michael Laue,[1] Ole Goldammer,[1] and Christoph Schneider*[1]

[1]Institute of Organic Chemistry, University of Leipzig, 04103 Leipzig, Germany.

*Email: schneider@chemie.uni-leipzig.de

## Content

Methods ........ 2
General information ........ 2
Reaction development ........ 3
General procedure for aldehyde *α*-oxidation (GP1) ........ 6
General procedure for ozonolysis (GP2) ........ 12
General procedure for hydrazone synthesis (GP3) ........ 13
General procedure for the *α*-substitution reaction (GP4) ........ 20
Postmodifications ........ 30
Synthesis of catalysts ........ 35
Mechanistic investigations ........ 40
NMR Spectra ........ 44
HPLC Chromatograms ........ 147
Crystallographic data ........ 176
Computational details ........ 197
References ........ 274

# Methods

## General information

All reactions in dry solvents were conducted under argon or nitrogen atmosphere, liquid reagents and solvents were transferred via syringe using standard Schlenk techniques. Dichloromethane, diethyl ether, tetrahydrofuran and toluene were dried by a solvent purification system (MB SPS-800 Braun). Chloroform (HPLC grade) and *n*-pentane (HPLC grade) were used without further purification. Solvents for extraction, column chromatography, preparative as well as analytical TLC were distilled from appropriate drying agents (hexane, diethyl ether: KOH, dichloromethane: $CaH_2$). Ethyl acetate and Methyl-*tert*-butyl ether were used without further purification. Flash chromatography was performed using Merck silica gel 60 230-400 mesh (0.040- 0.063 mm). Preparative and analytical TLC was performed using silica gel pre coated plates ALUGRAM Xtra SIL G/ UV254 and aluminum oxide pre coated plates ALUGRAM ALOX N/ UV254 by Macherey Nagel. $^{1}H$, $^{13}C$, $^{19}F$ and $^{31}P$ NMR spectra were recorded in $CDCl_3$ or $D_2O$ using a Bruker Avance III HD ($^{1}H$: 400 MHz; $^{13}C$: 100 MHz, $^{19}F$: 377 MHz; $^{31}P$: 162 MHz), a Varian MERCURYplus 400 ($^{1}H$: 400 MHz; $^{13}C$: 100 MHz, $^{19}F$: 377 MHz; $^{31}P$: 162 MHz) and a Varian MERCURYplus 300 ($^{1}H$: 300 MHz; $^{13}C$: 75 MHz, $^{31}P$: 121 MHz) spectrometer. The signals were referenced to residual chloroform ($\delta$ = 7.26 ppm for $^{1}H$ NMR, $\delta$ = 77.16 ppm for $^{13}C$ NMR) or $D_2O$ ($\delta$ = 4.79 ppm for $^{1}H$ NMR). Chemical shifts are reported in ppm, multiplicities are indicated by s (singlet), d (doublet), t (triplet), q (quartet), hept (heptet), dd (doublet of doublet), dt (doublet of triplet), dq (doublet of quartet), td (triplet of doublet), qd (quartet of doublet), qt (quartet of triplet), ddd (doublet of doublet of doublets), ddt (doublet of doublet of triplets), dtd (doublet of triplet of doublets), dddd (doublet of doublet of doublet of doublets), quin (quintet), sex (sextet), sep (septet), m (multiplet) and the prefix br (broad). Melting points were determined with a Büchi M-560 melting point apparatus and are uncorrected. IR spectra were obtained with a FTIR spectrometer (JASCO FT/IR-4100) and are reported in frequency of absorption ($cm^{-1}$). Optical rotations were measured using a Polarotronic polarimeter (Schmidt & Haensch). All ESI mass spectra were recorded on a Bruker ESI-TOF microTOF. Enantiomeric ratios (e.r.) were determined via HPLC on a JASCO MD-4015 instrument with a chiral stationary phase (Daicel Chiralpak IA, IC, IF, column). Diastereomeric ratios were determined by HPLC or $^{1}H$ NMR analysis of the crude reaction mixture.

Aliphatic aldehydes were prepared by Swern oxidation starting from commercially available alcohols. Bishomobenzylic aldehydes were prepared via Heck reaction starting from commercially available aryl iodides and 4-buten-1-ol.[1] Allyl acetates were prepared by acetyl protection starting from commercially available allyl alcohols.[2] N-Amino pyrrolidine was prepared following a literature known procedure.[3] The silylketene acetals **2a-2c** were prepared according to a literature known procedure.[4]

# Reaction development

## Optimization of the Catalyst:

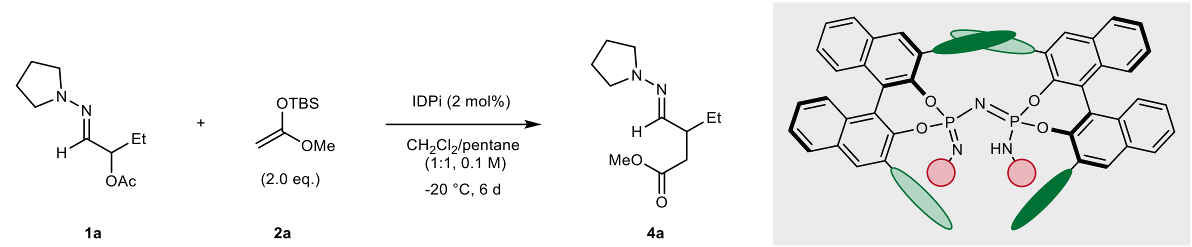


**Supplementary Figure 1:** Conditions for the catalyst optimization.

The catalyst optimization was carried out in $CH_2Cl_2$/pentane (1:1) as solvent mixture to ensure complete solubility of all reagents and intermediates. An oven dried screwcap vial was charged with a stir bar and the IDPi (2 mol%, 2 µmol). Subsequently $CH_2Cl_2$ (0.5 mL) followed by pentane (0.5 mL) and the silyl ketene acetal **2a** (2 equiv., 0.2 mmol) were added and the mixture was stirred at room temperature for 5 minutes. After cooling to -20 °C and stirring for further 5 minutes the hydrazone **1a** (1.0 equiv., 0.1 mmol) was added and the vial was sealed for 6 days. Finally an aliquot of the reaction mixture was used for the determination of enantioselectivity on a chiral HPLC (Chiralpak IA, hexane/iPrOH 99/1, flow rate = 1.0 mL/min, λ = 248 nm).

## General screening on BINOL backbone:

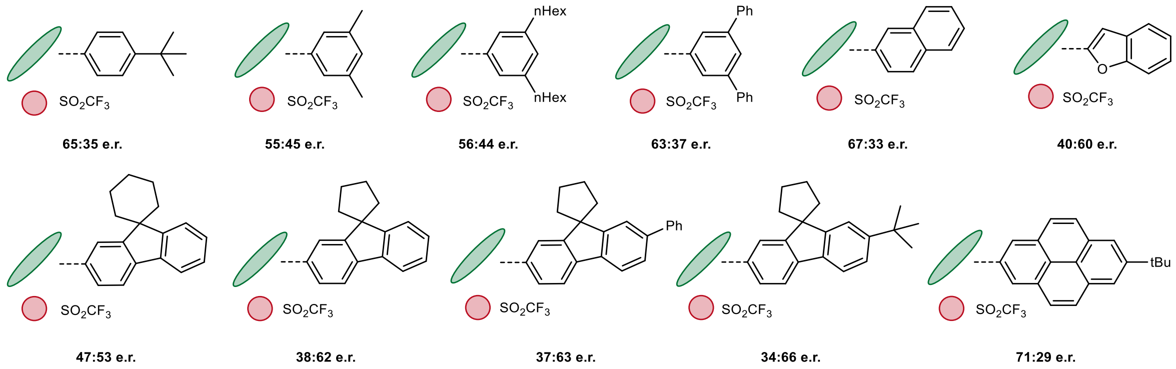


**Supplementary Figure 2:** Screening of general IDPi backbones.

The first attempt on screening different BINOL backbones revealed aromatic substituents in the *meta* position (2-naphthyl, pyrene, *meta*-biphenyl) as well as *para*-*t*butyl-phenyl as promising starting points for the optimization. Due to the relatively easy modification of the *meta*-aryl moiety and a large number of literature known IDPis bearing this scaffold we investigated several *meta*-substitutions.[5]

**Screening on *meta*-substituted backbone:**

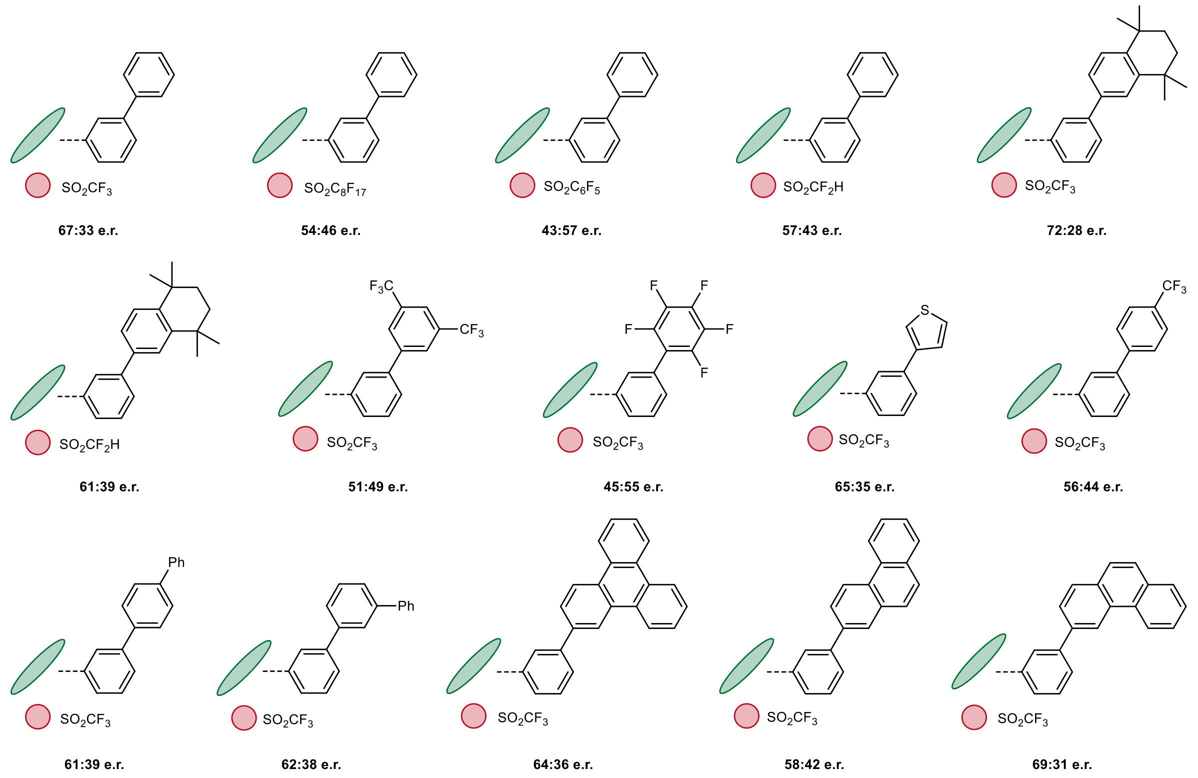


**Supplementary Figure 3:** Screening of *m*-aryl substituted backbones.

The screening of differently *meta*-substituted backbones revealed that bulky and non-polar aliphatic annulated rings gave only a small improvement. As variation of the *meta*-position itself was not sufficient for good enantioselectivity, new 3-fluorenyl substituted IDPis were developed.

**Screening on 3-fluorenyl backbone:**

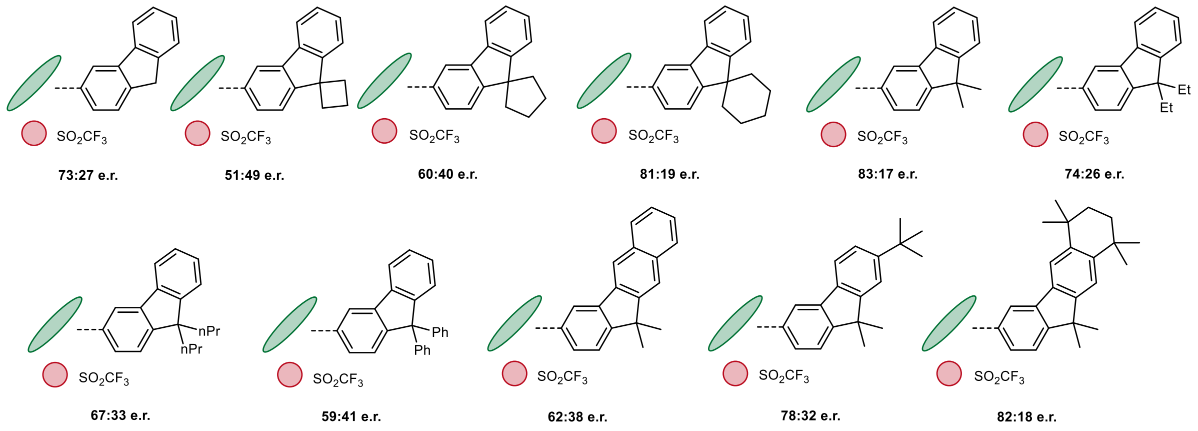


**Supplementary Figure 4:** Screening of 3-fluorenyl backbones.

9-Dimethyl-3-fluorenyl groups turned out to be superior 3,3´-substituents of the IDPi backbone. Annulation with a bulky aliphatic ring gave catalyst **3g** with a better solubility in pentane. Finally, incorporating an aryl linkage between the BINOL-backbone and the 3,3´-substituents revealed IDPi **3h** to be the optimal catalyst for the reaction in pure pentane.

## Final solvent and temperature investigation:

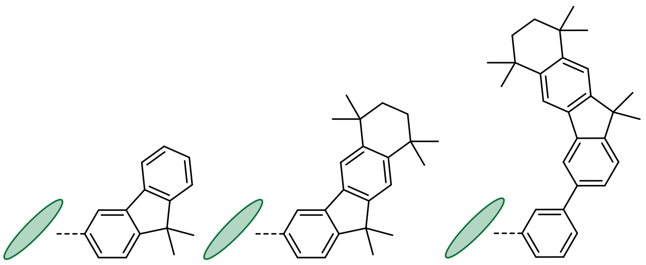

| Condition | $SO_2CF_3$ 3f | $SO_2CF_3$ 3g | $SO_2CF_3$ 3h |
|---|---|---|---|
| $CH_2Cl_2$/pentane (1:1) -20°C | 100% conv. 83:17 e.r. | 100% cov. 82:18 e.r. | |
| $CH_2Cl_2$/pentane (1:9) -30°C | 95% conv. 97:3 e.r. | 95% conv. 92:8 e.r. | 100% cov. 96:4 e.r. |
| pentane -20 °C | 80% conv. 96:4 e.r. | 95% cov. 96:4 e.r. | 100% cov. 98:2 e.r. |

**Supplementary Figure 5:** Solvent screening for the major IDPis using 1 mol% of IDPi.

## Investigation on catalyst scope:

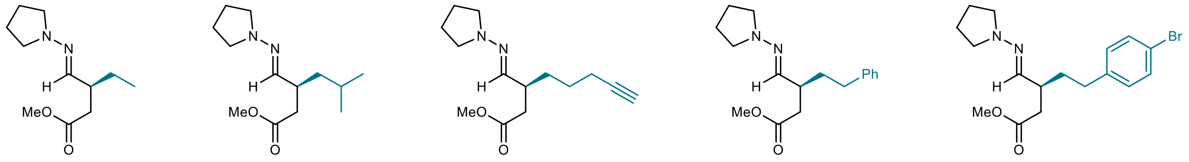


| 4a | 4g | 4m | 4q | 4u |
|---|---|---|---|---|
| IDPi 3f 97:03 e.r. | IDPi 3f 70:30 e.r. | IDPi 3f 94:06 e.r. | IDPi 3f 90:10 e.r. | IDPi 3f 80:20 e.r. |
| IDPi 3g 92:08 e.r. | IDPi 3g 80:20 e.r. | IDPi 3g 85:15 e.r. | IDPi 3g 93:07 e.r. | IDPi 3g 88:12 e.r. |
| IDPi 3h 98:02 e.r. | IDPi 3h 95:05 e.r. | IDPi 3h 96:04 e.r. | IDPi 3h 95:05 e.r. | IDPi 3h 95:05 e.r. |

**Supplementary Figure 6:** Substrate scope using different IDPi catalysts (1 mol%). Reactions with IDPi **3f** were carried out in $CH_2Cl_2$/pentane 1:9 at -30 °C. Reactions with IDPis **3g** and **3h** were carried out in pentane at -20 °C.

The differences in enantioselectivity using catalysts **3f** and **3g** are exemplified by the substrates shown in Supplementary Figure 6. In the event IDPi **3h** overcame this problem and showed a broad applicability for the complete substrate scope with only few examples where catalyst **3g** remained slightly more selective.

## Limitations:

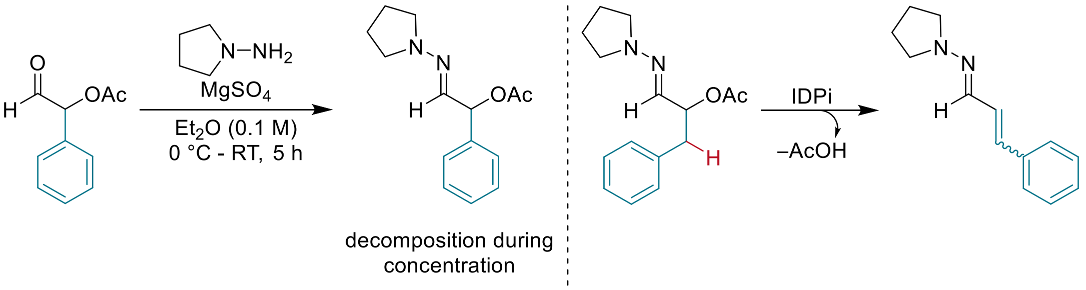


**Supplementary Figure 7:** Limitations of the substrate scope with aromatic substituents.

α-Aryl-substituted hydrazones decomposed during concentration of the reaction mixture at rt under argon. Hydrazones with a benzylic substituent at the α-position could be synthesized, but rapidly underwent elimination under GP 4 forming α,β-unsaturated hydrazones.

## General procedure for aldehyde *α*-oxidation (GP1)

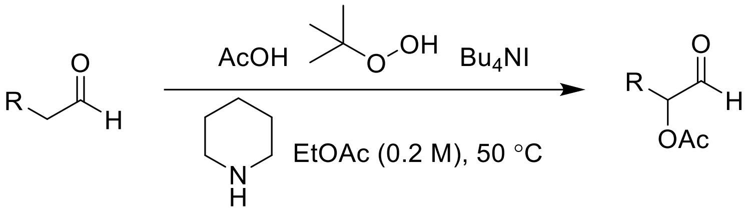


The *α*-oxidation was performed using a modified literature procedure:[6] To a stirred mixture of aldehyde (1.0 equiv.), acetic acid (1.0 equiv.), piperidine (5 mol%) and $Bu_4NI$ (10 mol%) in EtOAc (5 mL/mmol aldehyde)) was added *t*-butyl hydroperoxide (5.5M in decane, 1.1 eq.) at room temperature. The resulting mixture was heated to 50 °C. The reaction was monitored by TLC analysis. After the reaction was completed, the reaction mixture was cooled to room temperature and poured into $Na_2S_2O_3$ (5 mL/mmol) and $NaHCO_3$ (5 mL/mmol), extracted with EtOAc (twice), and washed with brine. The combined organic layers were dried over anhydrous $Na_2SO_4$ and the solvents were removed in vacuo. The residue was purified by flash column chromatography on silica gel (hexane–EtOAc as eluent). The aldehydes were further purified by vacuum distillation to give colorless oils.

**1-Oxopentan-2-yl acetate:**

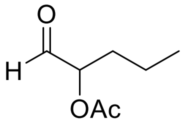


Prepared according to GP1, colorless oil, 74%. **$^1$H NMR** (400 MHz, $CDCl_3$): $\delta$ 9.51 (d, $J$ = 0.8 Hz, 1H), 5.00 (ddd, $J$ = 8.4, 4.8, 0.8 Hz, 1H), 2.17 (s, 3H), 1.87 – 1.61 (m, 2H), 1.53 – 1.36 (m, 2H), 0.95 (t, $J$ = 7.4 Hz, 3H). **$^{13}$C NMR** (101 MHz, $CDCl_3$): $\delta$ 199.9, 168.9, 78.1, 37.2, 22.4, 18.2, 13.0. **HRMS** *m/z* (+ESI): calc. for $C_7H_{12}O_3$ [M+H]: 145.0859; found: 145.0864. **IR** (film) ν [cm$^{-1}$]: 3462, 2964, 2939, 2876, 2816, 2732, 1739, 1467, 1436, 1372, 1239, 1146, 1128, 1120, 1083, 1032, 973, 929, 822, 741, 629, 608, 475. **B.p.**: 40 °C (0.5 mbar).

**1-Oxoheptan-2-yl acetate:**

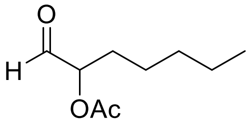


Prepared according to GP1, colorless oil, 69%. **$^1$H NMR** (400 MHz, $CDCl_3$): $\delta$ 9.51 (d, J = 0.8 Hz, 1H), 4.98 (ddd, $J$ = 8.4, 4.8, 0.8 Hz, 1H), 2.17 (s, 3H), 1.88 – 1.64 (m, 2H), 1.48 – 1.36 (m, 2H), 1.33 – 1.23 (m, 5H), 0.95 – 0.83 (m, 3H). **$^{13}$C NMR** (101 MHz, $CDCl_3$): $\delta$ 201.4, 169.4, 79.4, 35.7, 28.1, 25.4, 22.3, 19.9, 14.5. **HRMS** *m/z* (+ESI): calc. for $C_9H_{16}O_3$ [M+H]: 173.1172; found: 173.1183. **IR** (film) ν [cm$^{-1}$]: 3462, 2957, 2932, 2862, 2730, 1741, 1468, 1437, 1372, 1239, 1146, 1126, 1086, 1032, 951, 899, 822, 769, 728, 608, 487, 403. **B.p.**: 52 °C ($5\cdot10^{-2}$ mbar).

**1-Oxo-4-phenylbutan-2-yl acetate:**

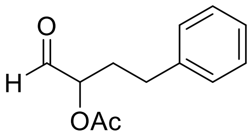


Prepared according to GP1, colorless oil, 66%. **$^1$H NMR** (400 MHz, $CDCl_3$): $\delta$ 9.49 (s, 1H), 7.34 – 7.28 (m, 2H), 7.24 – 7.20 (m, 1H), 7.20 – 7.14 (m, 3H), 4.99 (ddd, $J$ = 8.6, 4.6, 0.7 Hz, 1H), 2.84 – 2.59 (m, 3H), 2.19 (s, 3H), 2.18 – 2.00 (m, 2H). **$^{13}$C NMR** (101 MHz, $CDCl_3$): $\delta$ 198.1, 170.5, 140.2, 128.6, 128.4, 126.4, 77.6, 31.1, 30.3, 20.5. **HRMS** *m/z* (+ESI): calc. for $C_{12}H_{14}O_3Na$ [M+Na]: 229.0835; found: 229.0846. **IR** (film) ν [cm$^{-1}$]: 3461, 3086, 3062, 3028, 2932, 2861, 2827, 1739, 1604, 1496, 1455, 1435, 1372, 1234, 1182, 1143, 1103, 1079, 1043, 957, 817, 750, 701, 638, 605, 507, 495, 460, 427, 418, 403. **B.p.**: 106 °C ($1\cdot10^{-2}$ mbar).

**6-Chloro-1-oxohexan-2-yl acetate:**

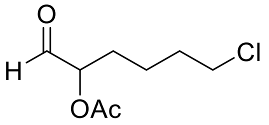

Prepared according to GP1, colorless oil, 72%. **¹H NMR** (400 MHz, $CDCl_3$): $\delta$ 9.52 (d, $J$ = 0.8 Hz, 1H), 4.99 (ddd, $J$ = 8.4, 4.7, 0.8 Hz, 1H), 3.54 (t, $J$ = 6.5 Hz, 2H), 2.18 (s, 3H), 1.95 – 1.67 (m, 5H), 1.64 – 1.50 (m, 2H). **¹³C NMR** (101 MHz, $CDCl_3$): $\delta$ 199.3, 170.6, 79.3, 43.5, 31.2, 28.6, 23.1, 19.8. **HRMS** *m/z* (+ESI): calc. for $C_8H_{14}O_3$ [M+H]: 193.0626; found: 193.0622. **IR** (film) ν [$cm^{-1}$]: 3460, 3126, 2954, 2870, 2731, 1739, 1651, 1458, 1446, 1436, 1372, 1290, 1233, 1163, 1104, 1069, 1052, 1033, 931, 871, 813, 740, 650, 608, 489, 457, 448, 419, 410. **B.p.**: 68 °C ($1\cdot10^{-2}$ mbar).

**4-Methoxy-1-oxobutan-2-yl acetate:**

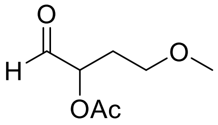

Prepared according to GP1, colorless oil, 83%. **¹H NMR** (400 MHz, $CDCl_3$): δ 9.50 (s, 1H), 5.13 (t, $J$ = 5.9 Hz, 1H), 3.57 – 3.38 (m, 3H), 3.29 (s, 4H), 2.17 (s, 3H), 2.13 – 2.04 (m, 2H). **¹³C NMR** (101 MHz, $CDCl_3$): δ 197.7, 171.0, 74.6, 67.3, 59.4, 30.9, 21.0. **HRMS** *m/z* (+ESI): calc. for $C_7H_{12}O_4Na$ [M+Na]: 183.0628; found: 183.0623. **IR** (film) ν [$cm^{-1}$]: 3454, 2979, 2933, 2876, 2833, 2817, 2737, 1739, 1653, 1482, 1432, 1373, 1240, 1190, 1157, 1116, 1045, 1033, 962, 951, 934, 921, 893, 863, 820, 794, 724, 668, 637, 607, 505, 490, 456, 418, 408. **B.p.**: 46 °C (0.2 mbar).

**4-(4-Bromophenyl)-1-oxobutan-2-yl acetate:**

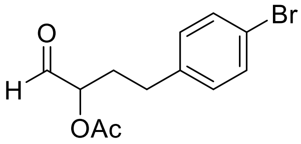

Prepared according to GP1, colorless oil, 55%. **¹H NMR** (400 MHz, $CDCl_3$): δ 9.50 (d, $J$ = 0.7 Hz, 1H), 7.49 – 7.34 (m, 2H), 7.13 – 6.96 (m, 2H), 4.97 (ddd, $J$ = 8.5, 4.6, 0.7 Hz, 1H), 2.78 – 2.61 (m, 2H), 2.18 (s, 3H), 2.16 – 1.94 (m, 2H). **¹³C NMR** (101 MHz, $CDCl_3$): δ 198.6, 170.5, 139.2, 131.7, 130.2, 120.3, 77.5, 30.6, 30.1, 20.6.**HRMS** *m/z* (+ESI): calc. for $C_{12}H_{13}BrO_3Na$ [M+Na]: 306.9940; found: 306.9941. **IR** (film) ν [$cm^{-1}$]: 3460, 3162, 3093, 3061, 3025, 2931, 2863, 2850, 2825, 2729, 1901, 1739, 1644, 1591, 1489, 1454, 1435, 1405, 1371, 1305, 1233, 1105, 1072, 1047, 1011, 957, 908, 890, 877, 802, 767, 713, 665, 638, 624, 606, 557, 516, 489, 481, 455, 448, 417, 406. **B.p.**: 125 °C ($1\cdot10^{-3}$ mbar).

**1-Oxodecan-2-yl acetate:**

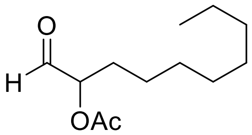

Prepared according to GP1, colorless oil, 70%. **¹H NMR** (400 MHz, $CDCl_3$): δ 9.51 (d, $J$ = 0.8 Hz, 1H), 4.98 (ddd, $J$ = 8.4, 4.8, 0.8 Hz, 1H), 2.17 (s, 3H), 1.89 – 1.65 (m, 2H), 1.47 – 1.35 (m, 2H), 1.34 – 1.23 (m, 10H), 0.92 – 0.84 (m, 3H). **¹³C NMR** (101 MHz, $CDCl_3$): δ 198.3, 170.6, 78.3, 31.8, 29.2, 29.2, 29.1, 28.6, 24.9, 22.6, 20.6, 14.1. **HRMS** *m/z* (+ESI): calc. for $C_{12}H_{23}O_3$ [M+H]: 215.1642; found: 215.1647. **IR** (film) ν [$cm^{-1}$]: 3462, 2954, 2926, 2856, 2729, 1742, 1652, 1466, 1438, 1372, 1235, 1181, 1126, 1045, 943, 895, 822, 759, 723, 665, 638, 607, 490, 469, 453, 435. **B.p.**: 75 °C ($1\cdot10^{-2}$ mbar).

**1-Oxohex-5-en-2-yl acetate:**

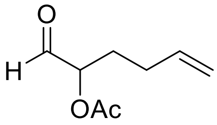


Prepared according to GP1, colorless oil, 68%. **$^{1}$H NMR** (300 MHz, $CDCl_3$): δ 9.52 (d, *J* = 0.7 Hz, 1H), 5.78 (ddt, *J* = 16.9, 10.2, 6.6 Hz, 1H), 5.11 – 4.97 (m, 3H), 2.27 – 2.12 (m, 5H), 2.03 – 1.73 (m, 2H). **$^{13}$C NMR** (75 MHz, $CDCl_3$): δ 198.2, 170.5, 136.5, 116.2, 77.6, 29.0, 27.9, 20.6. **HRMS** *m/z* (+ESI): calc. for $C_8H_{13}O_3$ [M+H]: 157.0859; found: 157.0851. **IR** (film) ν [$cm^{-1}$]: 3586, 3566, 3462, 3079, 2979, 2925, 2851, 1741, 1642, 1447, 1438, 1419, 1373, 1236, 1143, 1048, 997, 955, 916, 888, 823, 765, 721, 639, 608, 490, 458, 449. **B.p.**: 40 °C (0.1 mbar).

**4-Fluoro-1-oxobutan-2-yl acetate:**

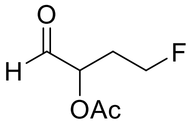


Prepared according to GP1, colorless oil, 46%. **$^{1}$H NMR** (300 MHz, $CDCl_3$): δ 9.55 (dd, *J* = 2.1, 0.6 Hz, 1H), 4.66 (qdd, *J* = 9.5, 6.6, 4.7 Hz, 1H), 4.50 (qdd, *J* = 9.5, 6.6, 4.7 Hz, 1H), 2.42 – 1.96 (m, 5H). **$^{13}$C NMR** (75 MHz, $CDCl_3$): δ 197.5, 170.3, 80.4, 78.1, 74.8, 74.8, 30.1, 29.8, 20.5. **$^{19}$F NMR** (376 MHz, $CDCl_3$): δ -219.79 (tt, *J* = 46.9, 25.9 Hz). **HRMS** *m/z* (+ESI): calc. for $C_6H_{10}FO_3$ [M+H]: 149.0608; found: 149.0601. **IR** (film) ν [$cm^{-1}$]: 3648, 3627, 3565, 3462, 2980, 2915, 2845, 1739, 1652, 1636, 1475, 1428, 1374, 1319, 1235, 1129, 1088, 1048, 1022, 996, 973, 956, 937, 920, 904, 824, 804, 719, 671, 607, 490, 457, 433, 425, 406. **B.p.**: 40 °C (0.4 mbar).

**4-(4-Chlorophenyl)-1-oxobutan-2-yl acetate:**

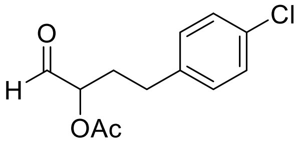


Prepared according to GP1, colorless oil, 57%. **$^{1}$H NMR** (400 MHz, $CDCl_3$): δ 9.50 (d, *J* = 0.7 Hz, 1H), 7.48 – 7.22 (m, 2H), 7.19 – 7.04 (m, 1H), 4.97 (ddd, *J* = 8.6, 4.6, 0.7 Hz, 1H), 2.89 – 2.54 (m, 2H), 2.19 (s, 3H), 2.17 – 1.97 (m, 2H). **$^{13}$C NMR** (101 MHz, $CDCl_3$): δ 198.0, 170.5, 138.6, 132.3, 129.8, 129.7, 128.8, 128.6, 77.5, 30.5, 30.2, 20.6. **HRMS** *m/z* (+ESI): calc. for $C_{12}H_{14}ClO_3$ [M+H]: 241.0626; found: 241.0627. **IR** (film) ν [$cm^{-1}$]: 3460, 3063, 3028, 2931, 2850, 2730, 2041, 1901, 1834, 1739, 1597, 1575, 1493, 1455, 1409, 1372, 1324, 1233, 1143, 1092, 1047, 1015, 957, 907, 876, 819, 807, 774, 757, 716, 664, 630, 607, 526, 464, 450, 429, 412. **B.p.**: 112 °C ($1 \cdot 10^{-3}$ mbar).

**4-(4-Fluorophenyl)-1-oxobutan-2-yl acetate:**

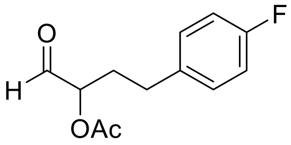


Prepared according to GP1, colorless oil, 61%. **$^{1}$H NMR** (400 MHz, $CDCl_3$): δ 9.49 (d, *J* = 0.7 Hz, 1H), 7.18 – 7.08 (m, 2H), 7.04 – 6.93 (m, 2H), 4.97 (ddd, *J* = 8.5, 4.6, 0.7 Hz, 1H), 2.72 (dt, *J* = 8.6, 6.5 Hz, 2H), 2.19 (s, 3H), 2.20 – 1.96 (m, 2H). **$^{13}$C NMR** (101 MHz, $CDCl_3$): δ 198.1, 170.5, 162.8, 160.4, 135.8, 135.8, 129.9, 129.8, 115.5, 115.3, 77.5, 30.4, 30.4, 30.3, 20.6. **$^{19}$F NMR** (377 MHz, $CDCl_3$): δ -116.72 (tt, *J* = 9.2, 5.3 Hz). **HRMS** *m/z* (+ESI): calc. for $C_{12}H_{13}FO_3Na$ [M+Na]: 247.0741; found: 247.0746. **IR** (film) ν [$cm^{-1}$]: 3459, 2932, 2865, 1739, 1602, 1510, 1454, 1436, 1373, 1227, 1159, 1104, 1071, 1047, 1016, 825, 771, 608, 419, 411. **B.p.**: 90 °C ($2 \cdot 10^{-3}$ mbar).

**1-Oxo-4-(4-(trifluoromethyl)phenyl)butan-2-yl acetate:**

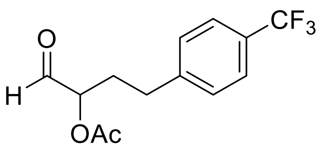


Prepared according to GP1, colorless oil, 60%. **$^1$H NMR** (400 MHz, $CDCl_3$): δ 9.51 (d, *J* = 0.7 Hz, 1H), 7.56 (br-d, *J* = 8.1 Hz, 2H), 7.30 (br-d, *J* = 8.0 Hz, 2H), 4.99 (dd, *J* = 8.5, 4.6 Hz, 1H), 2.80 (ddd, *J* = 9.0, 7.0, 0.7 Hz, 2H), 2.18 (s, 3H), 2.15 – 1.99 (m, 2H). **$^{13}$C NMR** (101 MHz, $CDCl_3$): δ 197.9, 170.5, 144.4, 128.8, 125.6, 125.6, 125.6, 125.5, 77.4, 31.0, 30.0, 20.5. **$^{19}$F NMR** (377 MHz, $CDCl_3$): δ -62.45. **HRMS** *m/z* (+ESI): calc. for $C_{13}H_{14}F_3O_3$ [M+H]: 275.0890; found: 275.0892. **IR** (film) ν [cm$^{-1}$]: 3462, 3232, 3140, 3071, 3046, 3023, 2936, 2848, 2732, 2647, 2448, 2307, 2230, 2184, 2133, 1922, 1740, 1653, 1619, 1584, 1540, 1519, 1454, 1436, 1419, 1373, 1327, 1234, 1187, 1164, 1121, 1067, 1049, 1018, 989, 955, 908, 890, 877, 844, 823, 760, 734, 667, 637, 598, 559, 517, 496, 451, 415. **B.p.**: 94 °C (1·10$^{-3}$ mbar).

**1-Oxohept-6-yn-2-yl acetate:**

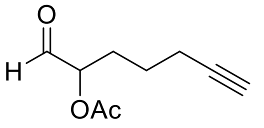


Prepared according to GP1, colorless oil, 48%. **$^1$H NMR** (400 MHz, $CDCl_3$): δ 9.52 (d, *J* = 0.7 Hz, 1H), 5.02 (ddd, *J* = 8.6, 4.6, 0.7 Hz, 1H), 2.24 (td, *J* = 6.9, 2.6 Hz, 2H), 2.18 (s, 3H), 2.06 – 1.92 (m, 2H), 1.92 – 1.78 (m, 1H), 1.72 – 1.57 (m, 2H). **$^{13}$C NMR** (101 MHz, $CDCl_3$): δ 198.0, 170.5, 83.1, 77.8, 69.3, 27.6, 23.8, 20.6, 18.1. **HRMS** *m/z* (+ESI): calc. for $C_9H_{16}NO_3$ [M+NH$_4$]: 186.1125; found: 186.1128. **IR** (film) ν [cm$^{-1}$]: 3465, 3291, 2939, 2871, 2846, 2116, 2042, 1738, 1520, 1506, 1456, 1436, 1374, 1238, 1063, 1027, 963, 909, 818, 645, 508, 484, 456. **B.p.**: 60 °C (3·10$^{-3}$ mbar).

**1-Oxoundec-10-en-2-yl acetate:**

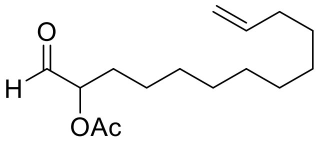


Prepared according to GP1, colorless oil, 73%. **$^1$H NMR** (400 MHz, $CDCl_3$): δ 9.51 (d, *J* = 0.8 Hz, 1H), 5.80 (ddt, *J* = 17.0, 10.1, 6.7 Hz, 1H), 5.04 – 4.87 (m, 3H), 2.17 (s, 3H), 2.07 – 1.99 (m, 2H), 1.91 – 1.63 (m, 2H), 1.54 – 1.13 (m, 10H). **$^{13}$C NMR** (101 MHz, $CDCl_3$): δ 198.3, 170.6, 139.0, 114.2, 78.3, 33.7, 29.2, 29.1, 28.9, 28.8, 28.6, 24.9, 20.6. **HRMS** *m/z* (+ESI): calc. for $C_{13}H_{23}O_3$ [M+H]: 227.1642; found: 227.1665. **IR** (film) ν [cm$^{-1}$]: 3467, 3077, 2978, 2927, 2855, 1743, 1640, 1464, 1440, 1371, 1239, 1197, 1078, 1044, 995, 910, 872, 724, 636, 609. **B.p.**: 73 °C (1·10$^{-3}$ mbar).

**5-(Benzyloxy)-1-oxopentan-2-yl acetate:**

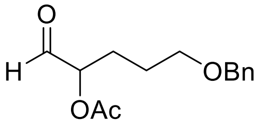


Prepared according to GP1, colorless oil, 69%. **$^1$H NMR** (400 MHz, $CDCl_3$): δ 9.51 (s, 1H), 7.56 – 7.17 (m, 5H), 5.02 (dd, *J* = 8.3, 4.7 Hz, 1H), 4.50 (s, 2H), 3.50 (t, *J* = 6.1 Hz, 2H), 2.16 (s, 3H), 2.06 – 1.92 (m, 1H), 1.83 (dtd, *J* = 13.8, 8.4, 5.7 Hz, 1H), 1.78 – 1.67 (m, 2H). **$^{13}$C NMR** (101 MHz, $CDCl_3$): δ 198.1, 170.6, 138.3, 128.4, 127.7, 127.7, 78.0, 73.0, 69.3, 25.6, 25.2, 20.6. **HRMS** *m/z* (+ESI): calc. for $C_{14}H_{19}O_4$ [M+H]: 251.1278; found: 251.1296. **IR** (film) ν [cm$^{-1}$]: 3457, 3063, 3031, 2938, 2857, 1738, 1496, 1480, 1454, 1372, 1237, 1102, 1078, 1047, 1028, 926, 820, 740, 699, 608, 456. **B.p.**: 124 °C (1·10$^{-3}$ mbar).

**1-Oxo-5-phenylpentan-2-yl acetate:**

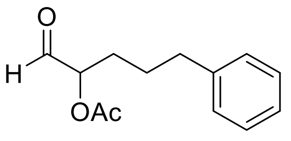


Prepared according to GP1, colorless oil, 71%. $^{1}$**H NMR** (400 MHz, $CDCl_3$): δ 9.50 (d, *J* = 0.8 Hz, 1H), 7.34 – 7.25 (m, 2H), 7.25 – 7.14 (m, 3H), 5.07 – 4.91 (m, 1H), 2.72 – 2.60 (m, 2H), 2.17 (s, 3H), 1.95 – 1.69 (m, 4H). $^{13}$**C NMR** (101 MHz, $CDCl_3$): δ 198.2, 170.6, 141.3, 128.5, 128.4, 126.1, 78.1, 35.4, 28.2, 26.7, 20.6. **HRMS** *m/z* (+ESI): calc. for $C_{13}H_{17}O_3$ [M+H]: 221.1172; found: 221.1185. **IR** (film) ν [cm$^{-1}$]: 3460, 3062, 3027, 2936, 2862, 1739, 1604, 1496, 1454, 1435, 1372, 1237, 1100, 1074, 1047, 1030, 750, 701, 607. **B.p.**: 105 °C (2·10$^{-3}$ mbar).

**4-(4-Cyanophenyl)-1-oxobutan-2-yl acetate:**

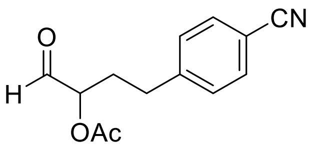


Prepared according to GP1, yellow oil, 37%. $^{1}$**H NMR** (400 MHz, $CDCl_3$): δ 9.51 (s, 1H), 7.63 – 7.54 (m, 2H), 7.32 – 7.25 (m, 2H), 4.98 (dd, *J* = 8.3, 4.6 Hz, 1H), 2.80 (ddd, *J* = 8.6, 6.9, 2.0 Hz, 2H), 2.32 – 1.87 (m, 5H). $^{13}$**C NMR** (101 MHz, $CDCl_3$): δ 197.9, 170.4, 145.9, 132.5, 129.3, 118.8, 110.5, 77.3, 31.2, 29.8, 20.5. **HRMS** *m/z* (+ESI): calc. for $C_{13}H_{13}NO_3Na$ [M+Na]: 254.0788; found: 254.0786. **IR** (film) ν [cm$^{-1}$]: 3461, 2974, 2933, 2863, 1739, 1489, 1460, 1435, 1404, 1371, 1238, 1196, 1180, 1102, 1072, 1047, 1022, 1011, 822, 798, 633, 605, 514, 506. **B.p.**: 147 °C (1·10$^{-3}$ mbar).

**4-(4-Methoxyphenyl)-1-oxobutan-2-yl acetate:**

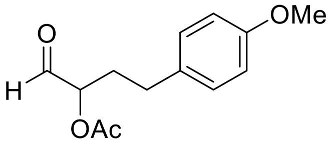


Prepared according to GP1, colorless oil, 56%. $^{1}$**H NMR** (400 MHz, $CDCl_3$): δ 9.48 (d, *J* = 0.8 Hz, 1H), 7.17 – 7.05 (m, 2H), 6.88 – 6.79 (m, 2H), 4.97 (dd, *J* = 8.7, 4.5 Hz, 1H), 3.79 (s, 3H), 2.67 (tdd, *J* = 13.9, 8.8, 6.5 Hz, 2H), 2.19 (s, 3H), 2.17 – 1.95 (m, 2H). $^{13}$**C NMR** (101 MHz, $CDCl_3$): δ 198.2, 170.6, 158.2, 132.1, 129.4, 114.0, 77.6, 55.3, 30.5, 30.2, 20.6. **HRMS** *m/z* (+ESI): calc. for $C_{13}H_{17}O_4$ [M+H]: 237.1121; found: 237.1126. **IR** (film) ν [cm$^{-1}$]: 3460, 3031, 2995, 2953, 2935, 2837, 1739, 1612, 1583, 1514, 1465, 1455, 1443, 1372, 1301, 1247, 1179, 1113, 1100, 1073, 1036, 957, 829, 822, 608, 523, 402. **B.p.**: 117 °C (1.0·10$^{-3}$ mbar).

**1-Oxo-4-(thiophen-2-yl)butan-2-yl acetate:**

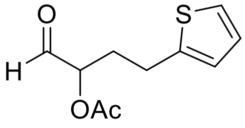


Prepared according to GP1, colorless oil, 37%. $^{1}$**H NMR** (400 MHz, $CDCl_3$): δ 9.50 (d, *J* = 0.7 Hz, 1H), 7.15 (dd, *J* = 5.2, 1.2 Hz, 1H), 6.93 (dd, *J* = 5.2, 3.4 Hz, 1H), 6.85 – 6.77 (m, 1H), 5.02 (dd, *J* = 8.7, 4.4 Hz, 1H), 3.06 – 2.89 (m, 2H), 2.35 – 2.05 (m, 5H). $^{13}$**C NMR** (101 MHz, $CDCl_3$): δ 197.8, 170.5, 142.6, 127.0, 125.0, 123.8, 77.3, 30.6, 25.3, 20.6. **HRMS** *m/z* (+ESI): calc. for $C_8H_{12}OS$ [M-OAc]: 153.0369; found: 153.1404. **IR** (film) ν [cm$^{-1}$]: 3459, 3109, 2926, 2850, 2827, 1739, 1441, 1372, 1233, 1133, 1098, 1078, 1049, 1035, 953, 849, 828, 702, 607. **B.p.**: 83 °C (1·10$^{-3}$ mbar).

**4-((tert-Butyldimethylsilyl)oxy)-1-oxobutan-2-yl acetate:**

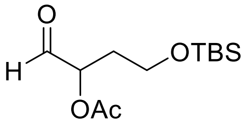


Prepared according to GP1, colorless oil, 64%. $^{1}$**H NMR** (400 MHz, $CDCl_3$): δ 9.53 (s, 1H), 5.15 (dd, *J* = 6.6, 5.2 Hz, 1H), 3.73 (dddd, *J* = 30.4, 10.2, 6.5, 5.0 Hz, 2H), 2.17 (s, 3H), 2.13 – 1.95 (m, 2H), 0.87 (s, 9H), 0.04 (s, 6H). $^{13}$**C NMR** (101 MHz, $CDCl_3$): δ 197.9, 170.4, 75.7, 58.0, 32.4, 25.8, 20.6, 18.2, -5.5, -5.6. **HRMS** *m/z* (+ESI): calc. for $C_{12}H_{25}SiO4$ [M+H]: 261.1517; found: 261.1528. **IR** (film) ν [cm$^{-1}$]: 3468, 2956, 2930, 2883, 2857, 1743, 1472, 1465, 1372, 1254, 1237, 1199, 1099, 1025, 1007, 878, 838, 808, 777, 664. **B.p.**: 80 °C (1·10$^{-3}$ mbar).

**Ethyl 5-acetoxy-6-oxohexanoate:**

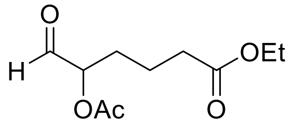

Prepared according to GP1, colorless oil, 57%. **$^{1}$H NMR** (400 MHz, $CDCl_3$): δ 9.51 (d, *J* = 0.8 Hz, 1H), 4.99 (dd, *J* = 7.8, 4.6 Hz, 1H), 4.13 (q, *J* = 7.1 Hz, 2H), 2.34 (t, *J* = 7.1 Hz, 2H), 2.18 (s, 3H), 2.01 – 1.67 (m, 4H), 1.25 (t, *J* = 7.1 Hz, 3H). **$^{13}$C NMR** (101 MHz, $CDCl_3$): δ 198.0, 172.8, 170.5, 77.9, 60.5, 33.6, 28.0, 20.6, 20.4, 14.2. **HRMS** *m/z* (+ESI): calc. for $C_{10}H_{17}O5$ [M+H]: 217.1071; found: 217.1067. **IR** (film) ν [$cm^{-1}$]: 3502, 3460, 3447, 2981, 2940, 1791, 1736, 1507, 1457, 1449, 1373, 1300, 1234, 1168, 1100, 1071, 1031, 607. **B.p.**: 86 °C ($1\cdot10^{-3}$ mbar).

## General procedure for ozonolysis (GP2)

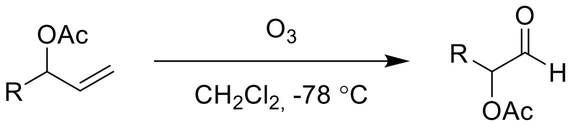


In a Schlenk flask the allylic acetate (1.0 equiv.) was dissolved in $CH_2Cl_2$ (0.15 M). The mixture was cooled to -78 °C and ozone was bubbled through the solution until a bright blue color was observed. The gas flow was switched to argon until the color faded and the reaction was quenched with $NEt_3$ (2.0 equiv.) at -78 °C and the reaction was allowed to warm to room temperature. After evaporation of the solvent pentane was added and the mixture was filtered through celite to remove the ammonium salt. The filtrate was washed with water and dried over $Na_2SO_4$. After evaporation of pentane the oily product was distilled under reduced pressure.

**1-Oxopropan-2-yl acetate:**

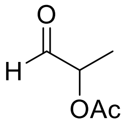


Prepared according to GP2, colorless oil, 45%. **$^1$H NMR** (400 MHz, $CDCl_3$): δ 9.54 (d, $J$ = 0.7 Hz, 1H), 5.06 (qd, $J$ = 7.2, 0.7 Hz, 1H), 2.17 (s, 3H), 1.39 (d, $J$ = 7.2 Hz, 3H). **$^{13}$C NMR** (101 MHz, $CDCl_3$): δ 198.5, 170.4, 74.6, 20.7, 14.1. **HRMS** *m/z* (+ESI): calc. for $C_5H_9O_3$ [M+H]: 117.0546; found: 117.0537. **IR** (film) ν [$cm^{-1}$]: 3462, 2991, 2942, 2829, 2727, 1739, 1450, 1373, 1238, 1179, 1126, 1074, 1018, 967, 949, 885, 861, 820, 668, 608. **B.p.**: 50 °C (18 mbar).

**1-Oxobutan-2-yl acetate:**

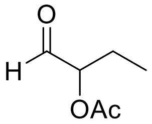


Prepared according to GP2, colorless oil, 67%. **$^1$H NMR** (300 MHz, $CDCl_3$): δ 9.51 (d, $J$ = 0.8 Hz, 1H), 4.93 (ddd, $J$ = 7.8, 5.0, 0.8 Hz, 1H), 2.17 (s, 3H), 1.99 – 1.67 (m, 2H), 1.00 (t, $J$ = 7.4 Hz, 3H). **$^{13}$C NMR** (75 MHz, $CDCl_3$): δ 198.4, 170.6, 79.3, 22.1, 20.5, 9.3. **HRMS** *m/z* (+ESI): calc. for $C_6H_{11}O_3$ [M+H]: 131.0703; found: 131.0706. **IR** (film) ν [$cm^{-1}$]: 3460, 2976, 2941, 1739, 1463, 1373, 1234, 1100, 1080, 1065, 1024, 974, 822, 775, 599, 587, 446. **B.p.**: 36 °C (2 mbar).

**3-Methyl-1-oxobutan-2-yl acetate:**

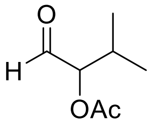


Prepared according to GP2, colorless oil, 51%. **$^1$H NMR** (400 MHz, $CDCl_3$): δ 9.53 (d, $J$ = 0.9 Hz, 1H), 4.85 (dd, $J$ = 4.7, 0.9 Hz, 1H), 2.26 (hd, $J$ = 6.9, 4.7 Hz, 1H), 2.18 (s, 3H), 1.02 (dd, $J$ = 20.9, 6.9 Hz, 6H). **$^{13}$C NMR** (101 MHz, $CDCl_3$): δ 198.7, 170.7, 82.3, 28.9, 20.5, 18.7, 17.2. **HRMS** *m/z* (+ESI): calc. for $C_7H_{12}O_3$ [M+H]: 145.0859; found: 145.0855. **IR** (film) ν [$cm^{-1}$]: 3460, 2970, 2937, 2879, 2822, 1739, 1468, 1435, 1373, 1260, 1234, 1185, 1148, 1114, 1068, 1039, 916, 813, 608. **B.p.**: 46 °C (0.5 mbar).

**4-Methyl-1-oxopentan-2-yl acetate:**

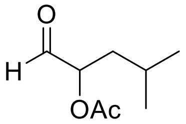


Prepared according to GP2, colorless oil, 75%. **$^1$H NMR** (400 MHz, $CDCl_3$) δ 9.50 (d, $J$ = 0.7 Hz, 1H), 5.08 – 4.99 (m, 1H), 2.17 (s, 3H), 1.87 – 1.71 (m, 1H), 1.63 (ddd, $J$ = 8.0, 5.5, 2.2 Hz, 2H), 0.95 (dd, $J$ = 10.8, 6.6 Hz, 6H). $^{13}$C NMR (101 MHz, $CDCl_3$) δ 198.4, 170.6, 77.1, 38.3, 24.5, 23.1, 21.6, 20.6. **HRMS** *m/z* (+ESI): calc. for $C_8H_{15}O_3$ [M+H]: 159.1016 found: 159.1018. **IR** (film) ν [$cm^{-1}$]: 3460, 2960, 2939, 2873, 1740, 1470, 1434, 1372, 1241, 1085, 1025. **B.p.**: 45 °C (0.25 mbar).

# General procedure for hydrazone synthesis (GP3)

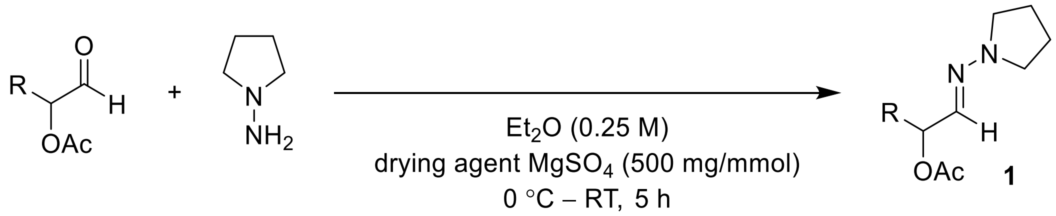


The hydrazones were synthesized following a modified literature procedure:[7] A solution of the 2-acetoxy aldehyde (1.0 equiv.) in diethyl ether (0.25 M) in the presence of $MgSO_4$ (500 mg/mmol) as drying agent was cooled to 0 °C. Then the hydrazine (1.0 equiv.) was added dropwise with stirring and the resulting mixture was allowed to warm to room temperature over 5 hours. After filtration and evaporation of the solvent the crude product was used without further purification. Decomposition of the hydrazones was observed during NMR measurement and even under argon at -30 °C. Traces of diethyl ether could not be removed from some of the hydrazones without the risk of decomposition. The hydrazones could only be stored for 4 – 8 weeks in the freezer. Optimal results were obtained by using freshly prepared hydrazones.

**(E)-1-(Pyrrolidin-1-ylimino)butan-2-yl acetate (1a):**

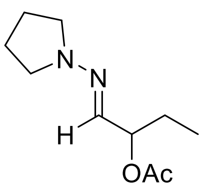


Prepared according to GP3 as clear oil without further purification. **$^{1}$H NMR** (300 MHz, $CDCl_3$): δ 6.33 (dt, *J* = 5.9, 0.7 Hz, 1H), 5.23 (td, *J* = 6.7, 5.8 Hz, 1H), 3.26 – 3.08 (m, 4H), 2.05 (s, 3H), 1.94 – 1.82 (m, 4H), 1.85 – 1.66 (m, 2H), 0.92 (t, *J* = 7.4 Hz, 3H). **$^{13}$C NMR** (75 MHz, $CDCl_3$): δ 170.4, 131.6, 76.0, 50.7, 26.1, 23.3, 21.3, 9.6. **HRMS** *m/z* (+ESI): calc. for $C_{10}H_{19}N_2O_2$ [M+H]: 199.1441; found: 199.1448. **IR** (film) ν [$cm^{-1}$]: 2966, 2935, 2876, 1717, 1559, 1458, 1363, 1339, 1252, 1143, 1010, 969, 878, 664, 613, 600, 450.

**(E)-1-(Pyrrolidin-1-ylimino)propan-2-yl acetate (1b):**

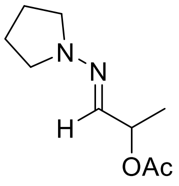


Prepared according to GP3 as clear oil without further purification. **$^{1}$H NMR** (400 MHz, $CDCl_3$): δ 6.38 (d, *J* = 5.4 Hz, 1H), 5.41 (qd, *J* = 6.5, 5.3 Hz, 1H), 3.27 – 2.99 (m, 4H), 2.04 (s, 3H), 1.92 – 1.82 (m, 4H), 1.38 (d, *J* = 6.5 Hz, 3H). **$^{13}$C NMR** (101 MHz, $CDCl_3$): δ 170.4, 132.1, 71.3, 50.7, 23.4, 21.4, 18.6. **HRMS** *m/z* (+ESI): calc. for $C_{19}H_{17}N_2O_2$ [M+H]: 185.1285; found: 185.1290. **IR** (film) ν [$cm^{-1}$]: 2971, 2935, 2876, 2575, 2484, 1717, 1614, 1557, 1457, 1419, 1384, 1374, 1341, 1253, 1144, 1046, 1011, 878, 663, 615, 450.

**(E)-1-(Pyrrolidin-1-ylimino)pentan-2-yl acetate (1c):**

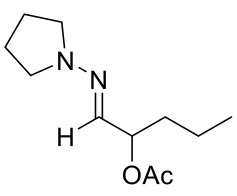


Prepared according to GP3 as clear oil without further purification. **$^{1}$H NMR** (400 MHz, $CDCl_3$): δ 6.32 (d, *J* = 5.7 Hz, 1H), 5.30 (dt, *J* = 7.4, 6.1 Hz, 1H), 3.32 – 2.96 (m, 4H), 2.03 (s, 3H), 1.93 – 1.78 (m, 4H), 1.79 – 1.61 (m, 2H), 1.47 – 1.29 (m, 2H), 0.91 (t, *J* = 7.4 Hz, 3H). **$^{13}$C NMR** (101 MHz, $CDCl_3$): δ 170.4, 131.9, 74.6, 50.7, 35.2, 23.4, 21.3, 18.5, 13.9. **HRMS** *m/z* (+ESI): calc. for $C_{11}H_{21}N_2O_2$ [M+H]: 213.1598; found: 213.1602. **IR** (film) ν [$cm^{-1}$]: 2961, 2935, 2873, 1737, 1592, 1460, 1435, 1371, 1340, 1238, 1147, 1124, 1104, 1017, 944, 920, 609.

**(E)-1-(Pyrrolidin-1-ylimino)heptan-2-yl acetate (1d):**

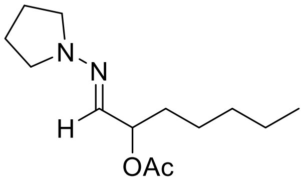


Prepared according to GP3 as clear oil without further purification. **$^1$H NMR** (400 MHz, $CDCl_3$): δ 6.32 (d, *J* = 5.9 Hz, 1H), 5.43 – 5.16 (m, 1H), 3.39 – 3.08 (m, 4H), 2.04 (s, 3H), 1.92 – 1.80 (m, 4H), 1.79 – 1.62 (m, 2H), 1.40 – 1.14 (m, 6H), 0.93 – 0.82 (m, 3H). **$^{13}$C NMR** (101 MHz, $CDCl_3$): δ 170.4, 132.0, 74.8, 50.7, 33.0, 31.6, 24.9, 23.4, 22.5, 21.4, 14.0. **HRMS** *m/z* (+ESI): calc. for $C_{13}H_{24}N_2O_2Na$ [M+Na]: 263.1730; found: 263.1751. **IR** (film) ν [$cm^{-1}$]: 2956, 2930, 2871, 2859, 1737, 1593, 1460, 1371, 1340, 1240, 1145, 1125, 1109, 1044, 1017, 951, 893, 609.

**(E)-1-(Pyrrolidin-1-ylimino)decan-2-yl acetate (1e):**

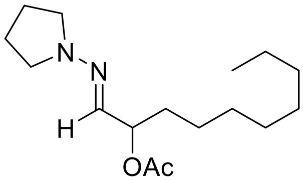


Prepared according to GP3 as clear oil without further purification. **$^1$H NMR** (400 MHz, $CDCl_3$): δ 6.33 (d, *J* = 5.9 Hz, 1H), 5.36 – 5.19 (m, 1H), 3.23 – 2.97 (m, 4H), 2.05 (s, 3H), 1.95 – 1.82 (m, 4H), 1.78 – 1.65 (m, 2H), 1.40 – 1.16 (m, 12H), 0.92 – 0.83 (m, 3H). **$^{13}$C NMR** (101 MHz, $CDCl_3$): δ 170.4, 131.9, 74.8, 50.7, 33.0, 31.8, 29.4, 29.4, 29.2, 25.2, 23.3, 22.6, 21.3, 14.1. **HRMS** *m/z* (+ESI): calc. for $C_{16}H_{31}N_2O_2$ [M+H]: 283.2380; found: 283.2384. **IR** (film) ν [$cm^{-1}$]: 2955, 2925, 2854, 2575, 2486, 1722, 1715, 1563, 1462, 1415, 1369, 1340, 1241, 1142, 1013, 968, 876, 723, 666, 613, 449.

**(E)-3-Methyl-1-(pyrrolidin-1-ylimino)butan-2-yl acetate (1f):**

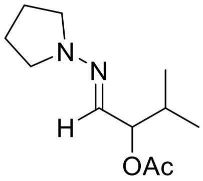


Prepared according to GP3 as clear oil without further purification. **$^1$H NMR** (400 MHz, $CDCl_3$): δ 6.37 – 6.16 (m, 1H), 5.10 (t, *J* = 6.5 Hz, 1H), 3.23 – 2.98 (m, 4H), 2.05 (s, 3H), 2.04 – 1.96 (m, 1H), 1.93 – 1.83 (m, 4H), 0.93 (dd, *J* = 6.8, 3.1 Hz, 6H). **$^{13}$C NMR** (101 MHz, $CDCl_3$): δ 170.3, 131.2, 79.1, 50.8, 31.5, 23.3, 21.3, 18.3, 18.1. **HRMS** *m/z* (+ESI): calc. for $C_{19}H_{17}N_2$ [M-OAc]: 153.1386; found: 153.1405. **IR** (film) ν [$cm^{-1}$]: 2965, 2875, 2837, 1735, 1592, 1461, 1385, 1371, 1340, 1238, 1149, 1133, 1018, 972, 956, 930, 905.

**(E)-4-Methyl-1-(pyrrolidin-1-ylimino)pentan-2-yl acetate (1g):**

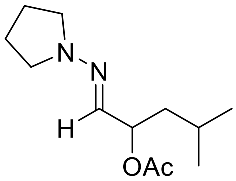


Prepared according to GP3 as clear oil without further purification. **$^1$H NMR** (400 MHz, $CDCl_3$): δ 6.49 – 6.15 (m, 1H), 5.38 (dt, *J* = 8.1, 5.9 Hz, 1H), 3.27 – 2.99 (m, 4H), 2.03 (s, 3H), 1.94 – 1.77 (m, 4H), 1.76 – 1.59 (m, 2H), 1.57 – 1.46 (m, 1H), 0.91 (dd, *J* = 7.5, 6.4 Hz, 6H). **$^{13}$C NMR** (101 MHz, $CDCl_3$): δ 170.4, 132.2, 73.3, 50.7, 41.9, 24.5, 23.3, 22.8, 22.4, 21.4. **HRMS** *m/z* (+ESI): calc. for $C_{12}H_{23}N_2O_2$ [M+H]: 227.1754; found: 227.1766. **IR** (film) ν [$cm^{-1}$]: 2956, 2871, 1753, 1720, 1557, 1465, 1410, 1384, 1367, 1340, 1255, 1010, 971, 877, 615, 450.

**(E)-4-Methoxy-1-(pyrrolidin-1-ylimino)butan-2-yl acetate (1h):**

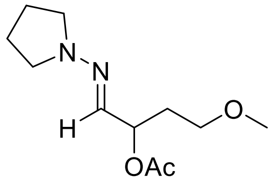


Prepared according to GP3 as clear oil without further purification. **$^{1}$H NMR** (400 MHz, $CDCl_3$): δ 6.48 – 6.26 (m, 1H), 5.42 (td, *J* = 6.7, 5.4 Hz, 1H), 3.44 (td, *J* = 6.6, 3.2 Hz, 2H), 3.31 (s, 3H), 3.25 – 3.07 (m, 4H), 2.09 – 1.98 (m, 5H), 1.95 – 1.84 (m, 4H). **$^{13}$C NMR** (101 MHz, $CDCl_3$): δ 170.3, 130.9, 72.0, 68.9, 58.6, 50.7, 32.8, 23.4, 21.3. **HRMS** *m/z* (+ESI): calc. for $C_{11}H_{21}N_2O_3$ [M+H]: 229.1547; found: 229.1547. **IR** (film) ν [cm$^{-1}$]: 2972, 2931, 2875, 2828, 2578, 2487, 1750, 1720, 1644, 1556, 1484, 1459, 1415, 1383, 1340, 1246, 1196, 1117, 1029, 1010, 971, 876, 665, 613, 450.

**(E)-5-(Benzyloxy)-1-(pyrrolidin-1-ylimino)pentan-2-yl acetate (1i):**

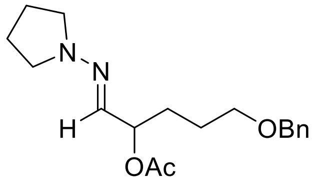


Prepared according to GP3 as clear oil without further purification. **$^{1}$H NMR** (400 MHz, $CDCl_3$): δ 7.46 – 7.14 (m, 5H), 6.33 (d, *J* = 5.8 Hz, 1H), 5.33 (q, *J* = 6.6 Hz, 1H), 4.50 (s, 2H), 3.49 (t, *J* = 6.5 Hz, 2H), 3.23 – 3.07 (m, 4H), 2.04 (s, 3H), 1.95 – 1.79 (m, 6H), 1.75 – 1.58 (m, 2H). **$^{13}$C NMR** (101 MHz, $CDCl_3$): δ 170.4, 138.5, 131.3, 128.4, 127.6, 127.5, 74.5, 72.9, 70.0, 50.7, 29.7, 25.6, 23.4, 21.4. **HRMS** *m/z* (+ESI): calc. for $C_{18}H_{27}N_2O_3$ [M+H]: 319.2016; found: 319.2014. **IR** (film) ν [cm$^{-1}$]: 2943, 2856, 1719, 1557, 1496, 1454, 1384, 1362, 1340, 1245, 1102, 1028, 1010, 970, 739, 699, 612, 450.

**(E)-4-((tert-Butyldimethylsilyl)oxy)-1-(pyrrolidin-1-ylimino)butan-2-yl acetate (1j):**

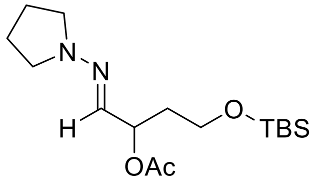


Prepared according to GP3 as clear oil without further purification. **$^{1}$H NMR** (400 MHz, $CDCl_3$): δ 6.40 (dd, *J* = 5.3, 0.9 Hz, 1H), 5.42 (dt, *J* = 7.4, 5.8 Hz, 1H), 3.68 (t, *J* = 6.5 Hz, 2H), 3.22 – 3.03 (m, 4H), 2.04 (s, 3H), 2.03 – 1.96 (m, 2H), 1.91 – 1.86 (m, 4H), 0.88 (s, 9H), 0.03 (s, 6H). **$^{13}$C NMR** (101 MHz, $CDCl_3$): δ 170.3, 131.2, 71.8, 59.4, 50.7, 35.8, 25.9, 23.4, 21.3, 18.3, -5.4. **HRMS** *m/z* (+ESI): calc. for $C_{16}H_{33}N_3O_3Si$ [M+H]: 329.2255; found: 329.2255. **IR** (film) ν [cm$^{-1}$]: 2955, 2930, 2882, 2856, 1719, 1559, 1471, 1463, 1385, 1362, 1254, 1098, 1007, 876, 837, 812, 777, 664, 450.

**(E)-1-(Pyrrolidin-1-ylimino)hex-5-en-2-yl acetate (1k):**

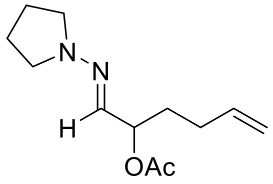


Prepared according to GP3 as clear oil without further purification. **$^{1}$H NMR** (400 MHz, $CDCl_3$): δ 6.33 (d, *J* = 5.7 Hz, 1H), 5.81 (ddt, *J* = 16.9, 10.1, 6.5 Hz, 1H), 5.37 – 5.26 (m, 1H), 5.09 – 4.90 (m, 2H), 3.26 – 2.96 (m, 4H), 2.25 – 1.97 (m, 5H), 1.95 – 1.75 (m, 6H). **$^{13}$C NMR** (101 MHz, $CDCl_3$): δ 170.4, 137.7, 131.3, 115.0, 74.2, 50.7, 32.2, 29.5, 23.4, 21.3. **HRMS** *m/z* (+ESI): calc. for $C_{12}H_{21}N_2O_2$ [M+H]: 225.1598; found: 225.1597. **IR** (film) ν [cm$^{-1}$]: 3077, 2974, 2848, 1719, 1640, 1559, 1458, 1434, 1415, 1384, 1367, 1340, 1246, 1003, 912, 613, 450.

**(E)-1-(Pyrrolidin-1-ylimino)undec-10-en-2-yl acetate (1l):**

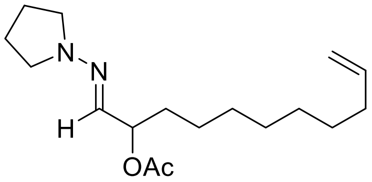


Prepared according to GP3 as clear oil without further purification. **$^1$H NMR** (400 MHz, $CDCl_3$): δ 6.33 (d, *J* = 5.9 Hz, 1H), 5.80 (ddt, *J* = 16.9, 10.1, 6.7 Hz, 1H), 5.29 (q, *J* = 6.6 Hz, 1H), 5.03 – 4.88 (m, 2H), 3.21 – 3.13 (m, 4H), 2.15 – 1.97 (m, 5H), 1.95 – 1.82 (m, 4H), 1.79 – 1.65 (m, 2H), 1.44 – 1.20 (m, 10H). **$^{13}$C NMR** (101 MHz, $CDCl_3$): δ 170.4, 139.2, 131.9, 114.1, 74.8, 50.8, 33.8, 33.1, 29.3, 29.3, 29.0, 28.9, 25.2, 23.4, 21.4. **HRMS** *m/z* (+ESI): calc. for $C_{17}H_{31}N_2O_2$ [M+H]: 295.2380; found: 295.2382. **IR** (film) ν [cm$^{-1}$]: 3076, 2973, 2927, 2855, 1736, 1658, 1641, 1592, 1489, 1460, 1441, 1370, 1340, 1238, 1017, 995, 955, 909, 608, 403.

**(E)-1-(Pyrrolidin-1-ylimino)hept-6-yn-2-yl acetate (1m):**

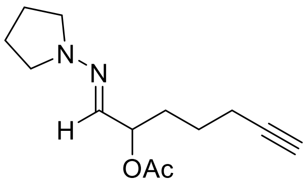


Prepared according to GP3 as clear oil without further purification. **$^1$H NMR** (300 MHz, $CDCl_3$): δ 6.32 (d, *J* = 5.7 Hz, 1H), 5.32 (td, *J* = 6.8, 5.7 Hz, 1H), 3.23 – 3.08 (m, 4H), 2.22 (td, *J* = 7.1, 2.6 Hz, 2H), 2.05 (s, 3H), 1.94 (t, *J* = 2.6 Hz, 1H), 1.92 – 1.79 (m, 6H), 1.68 – 1.48 (m, 2H). **$^{13}$C NMR** (101 MHz, $CDCl_3$): δ 170.4, 131.0, 84.0, 74.2, 68.6, 50.7, 32.1, 24.3, 23.4, 21.3, 18.2. **HRMS** *m/z* (+ESI): calc. for $C_{13}H_{21}N_2O_2$ [M+H]: 237.1598; found: 237.1593. **IR** (film) ν [cm$^{-1}$]: 3291, 2967, 2946, 2866, 2847, 1718, 1557, 1485, 1459, 1435, 1383, 1369, 1339, 1239, 1131, 1109, 1046, 1011, 970, 877, 660, 639, 619, 449.

**(E)-4-Fluoro-1-(pyrrolidin-1-ylimino)butan-2-yl acetate (1n):**

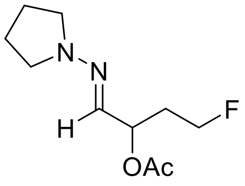


Prepared according to GP3 as clear oil without further purification. **$^1$H NMR** (400 MHz, $CDCl_3$): δ 6.39 (d, *J* = 5.2 Hz, 1H), 5.48 (dt, *J* = 7.4, 5.6 Hz, 1H), 4.59 (td, *J* = 5.9, 1.3 Hz, 1H), 4.48 (td, *J* = 5.9, 1.5 Hz, 1H), 3.25 – 3.06 (m, 4H), 2.32 – 2.10 (m, 2H), 2.06 (s, 3H), 1.93 – 1.83 (m, 4H). **$^{13}$C NMR** (101 MHz, $CDCl_3$): δ 170.4, 129.9, 81.4, 79.8, 71.1, 71.1, 50.7, 33.5, 33.3, 23.4, 21.2. **$^{19}$F NMR** (377 MHz, $CDCl_3$): δ -220.38 (tt, *J* = 47.7, 24.6 Hz). **HRMS** *m/z* (+ESI): calc. for $C_{10}H_{18}FN_2O_2$ [M+H]: 217.1347; found: 217.1350. **IR** (film) ν [cm$^{-1}$]: 3440, 2968, 2877, 1835, 1736, 1722, 1584, 1548, 1458, 1448, 1372, 1340, 1228, 1144, 1108, 1018, 881, 753, 614, 605, 452.

**(E)-6-Chloro-1-(pyrrolidin-1-ylimino)hexan-2-yl acetate (1o):**

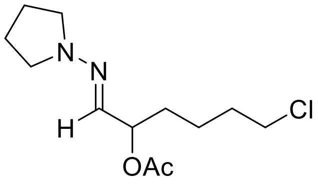


Prepared according to GP3 as clear oil without further purification. **$^1$H NMR** (400 MHz, $CDCl_3$): δ 6.32 (d, *J* = 5.7 Hz, 1H), 5.31 (q, *J* = 6.5 Hz, 1H), 3.53 (t, *J* = 6.7 Hz, 2H), 3.27 – 3.02 (m, 4H), 2.05 (s, 3H), 1.89 (td, *J* = 7.9, 6.8, 4.6 Hz, 4H), 1.83 – 1.71 (m, 4H), 1.50 (ddq, *J* = 14.2, 8.4, 7.0 Hz, 2H). **$^{13}$C NMR** (101 MHz, $CDCl_3$): δ 170.4, 131.1, 74.4, 50.7, 44.8, 32.3, 32.1, 23.4, 22.6, 21.3. **HRMS** *m/z* (+ESI): calc. for $C_{12}H_{22}ClN_2O_2$ [M+H]: 261.1364; found: 261.1359. **IR** (film) ν [cm$^{-1}$]: 2957, 2867, 1718, 1558, 1458, 1447, 1384, 1371, 1340, 1250, 1009, 971, 876, 726, 651, 614, 450.

**Ethyl (E)-5-acetoxy-6-(pyrrolidin-1-ylimino)hexanoate (1p):**

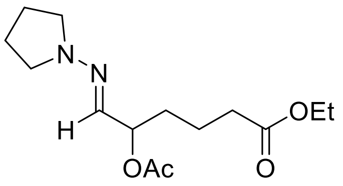


Prepared according to GP3 as clear oil without further purification. **$^1$H NMR** (400 MHz, $CDCl_3$): δ 6.32 (d, $J$ = 5.7 Hz, 1H), 5.31 (q, $J$ = 6.3 Hz, 1H), 4.12 (q, $J$ = 7.1 Hz, 2H), 3.17 (td, $J$ = 6.8, 3.3 Hz, 4H), 2.32 (t, $J$ = 7.3 Hz, 2H), 2.05 (s, 3H), 1.94 – 1.79 (m, 4H), 1.79 – 1.63 (m, 4H), 1.25 (t, $J$ = 7.1 Hz, 3H). **$^{13}$C NMR** (101 MHz, $CDCl_3$): δ 173.4, 170.4, 130.9, 74.3, 60.3, 50.7, 34.0, 32.3, 23.4, 21.3, 20.8, 14.2. **HRMS** *m/z* (+ESI): calc. for $C_{12}H_{21}N_2O_2$ [M-OAc]: 225.1598; found: 225.1622. **IR** (film) ν [$cm^{-1}$]: 3502, 3460, 3447, 2981, 2940, 1791, 1736, 1507, 1457, 1449, 1373, 1300, 1234, 1168, 1100, 1071, 1031, 607.

**(E)-4-Phenyl-1-(pyrrolidin-1-ylimino)butan-2-yl acetate (1q):**

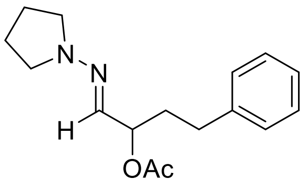


Prepared according to GP3 as clear oil without further purification. **$^1$H NMR** (400 MHz, $CDCl_3$): δ 7.33 – 7.23 (m, 1H), 7.18 (d, $J$ = 7.3 Hz, 1H), 6.34 (dt, $J$ = 5.6, 0.7 Hz, 0H), 5.36 (dt, $J$ = 7.2, 6.0 Hz, 0H), 3.23 – 3.11 (m, 2H), 2.85 – 2.54 (m, 1H), 2.17 – 2.04 (m, 2H), 1.92 – 1.81 (m, 2H). **$^{13}$C NMR** (101 MHz, $CDCl_3$): δ 170.4, 141.6, 131.1, 128.4, 128.4, 125.8, 74.3, 50.7, 34.6, 31.7, 23.4, 21.3. **HRMS** *m/z* (+ESI): calc. for $C_{14}H_{19}N_2$ [M-OAc]: 215.1543; found: 215.1569. **IR** (film) ν [$cm^{-1}$]: 3026, 2967, 2941, 2859, 1716, 1602, 1584, 1554, 1496, 1454, 1384, 1371, 1339, 1256, 1030, 1011, 879, 750, 700, 616, 451.

**(E)-5-Phenyl-1-(pyrrolidin-1-ylimino)pentan-2-yl acetate (1r):**

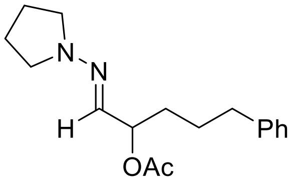


Prepared according to GP3 as clear oil without further purification. **$^1$H NMR** (400 MHz, $CDCl_3$): δ 7.32 – 7.22 (m, 2H), 7.22 – 7.11 (m, 3H), 6.31 (d, $J$ = 5.9 Hz, 1H), 5.35 (dt, $J$ = 7.2, 6.0 Hz, 1H), 3.26 – 3.07 (m, 4H), 2.64 (t, $J$ = 7.5 Hz, 2H), 2.05 (s, 3H), 1.94 – 1.78 (m, 4H), 1.85 – 1.59 (m, 4H). **$^{13}$C NMR** (101 MHz, $CDCl_3$): δ 170.4, 142.2, 131.4, 128.4, 128.3, 125.8, 74.6, 50.7, 35.6, 32.7, 27.1, 23.4, 21.4. **HRMS** *m/z* (+ESI): calc. for $C_{17}H_{25}N_2O_2$ [M+H]: 289.1911; found: 289.1928. **IR** (film) ν [$cm^{-1}$]: 3460, 3027, 2936, 2862, 1739, 1496, 1454, 1435, 1372, 1237, 1100, 1074, 1047, 1030, 750, 701.

**(E)-1-(Pyrrolidin-1-ylimino)-4-(thiophen-2-yl)butan-2-yl acetate (1s):**

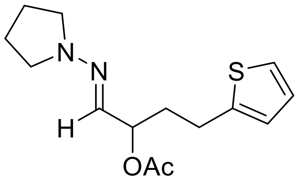


Prepared according to GP3 as clear oil without further purification. **$^1$H NMR** (400 MHz, $CDCl_3$) δ 7.10 (dd, $J$ = 5.2, 1.2 Hz, 1H), 6.90 (dd, $J$ = 5.1, 3.4 Hz, 1H), 6.84 – 6.76 (m, 1H), 6.33 (d, $J$ = 5.6 Hz, 1H), 5.37 (dt, $J$ = 7.3, 5.9 Hz, 1H), 3.26 – 3.12 (m, 4H), 3.01 – 2.81 (m, 2H), 2.22 – 2.08 (m, 2H), 2.06 (s, 3H), 1.95 – 1.84 (m, 4H). **$^{13}$C NMR** (101 MHz, $CDCl_3$) δ 170.4, 144.3, 130.6, 126.8, 124.3, 123.1, 74.0, 50.7, 34.8, 25.8, 23.4, 21.3. **HRMS** *m/z* (+ESI): calc. for $C_{14}H_{21}N_2O_2S$ [M+H]: 281.1318; found: 281.1327. **IR** (film) ν [$cm^{-1}$]: 2968, 2933, 2873, 1718, 1555, 1538, 1439, 1384, 1372, 1340, 1240, 1032, 1011, 968, 849, 825, 697, 613, 453.

**(E)-4-(4-Chlorophenyl)-1-(pyrrolidin-1-ylimino)butan-2-yl acetate (1t):**

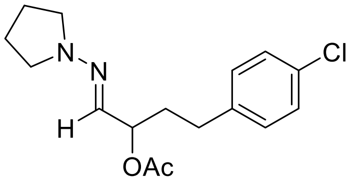


Prepared according to GP3 as clear oil without further purification. **$^1$H NMR** (400 MHz, $CDCl_3$): δ 7.26 – 7.19 (m, 2H), 7.14 – 7.06 (m, 2H), 6.31 (dt, *J* = 5.7, 0.8 Hz, 1H), 5.33 (dt, *J* = 7.1, 5.9 Hz, 1H), 3.24 – 3.01 (m, 4H), 2.65 (qdd, *J* = 14.0, 9.2, 6.5 Hz, 2H), 2.11 – 1.98 (m, 5H), 1.95 – 1.78 (m, 4H). **$^{13}$C NMR** (101 MHz, $CDCl_3$): δ 170.4, 140.0, 131.6, 130.7, 129.8, 128.5, 74.2, 50.7, 34.4, 31.1, 23.4, 21.3. **HRMS** *m/z* (+ESI): calc. for $C_{16}H_{22}N_2O_2Cl$ [M+H]: 309.1364; found: 309.1367. **IR** (film) ν [cm$^{-1}$]: 3025, 2970, 2935, 2871, 2581, 1718, 1595, 1556, 1492, 1458, 1434, 1407, 1365, 1340, 1246, 1092, 1045, 1014, 970, 877, 837, 807, 664, 614, 450.

**(E)-4-(4-Bromophenyl)-1-(pyrrolidin-1-ylimino)butan-2-yl acetate (1u):**

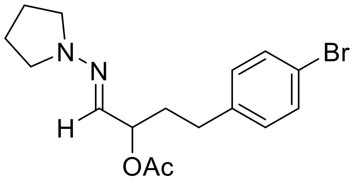


Prepared according to GP3 as clear oil without further purification. **$^1$H NMR** (400 MHz, $CDCl_3$): δ 7.38 (d, *J* = 8.3 Hz, 2H), 7.05 (d, *J* = 8.3 Hz, 2H), 6.30 (d, *J* = 5.6 Hz, 1H), 5.43 – 5.12 (m, 1H), 3.25 – 2.89 (m, 4H), 2.77 – 2.45 (m, 2H), 2.16 – 1.97 (m, 5H), 1.95 – 1.82 (m, 4H). **$^{13}$C NMR** (101 MHz, $CDCl_3$): δ 170.4, 140.5, 131.4, 130.7, 130.2, 119.6, 74.2, 50.7, 34.3, 31.1, 23.4, 21.3. **HRMS** *m/z* (+ESI): calc. for $C_{16}H_{22}BrN_2O_2$ [M+H]: 375.0859; found: 375.0850. **IR** (film) ν [cm$^{-1}$]: 3023, 2971, 2860, 1717, 1589, 1556, 1488, 1457, 1434, 1403, 1369, 1340, 1246, 1112, 1071, 1011, 970, 877, 833, 803, 665, 614, 450.

**(E)-4-(4-Fluorophenyl)-1-(pyrrolidin-1-ylimino)butan-2-yl acetate (1v):**

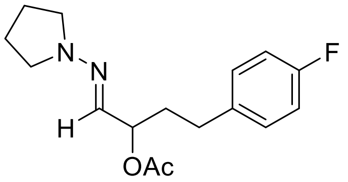


Prepared according to GP3 as clear oil without further purification. **$^1$H NMR** (400 MHz, $CDCl_3$): δ 7.13 (dd, *J* = 8.5, 5.5 Hz, 2H), 7.01 – 6.89 (m, 2H), 6.32 (d, *J* = 5.6 Hz, 1H), 5.33 (dt, *J* = 7.3, 6.0 Hz, 1H), 3.26 – 3.09 (m, 4H), 2.65 (qdd, *J* = 14.0, 9.2, 6.4 Hz, 2H), 2.19 – 1.98 (m, 5H), 1.94 – 1.82 (m, 4H). **$^{13}$C NMR** (101 MHz, $CDCl_3$): δ 170.4, 162.5, 160.1, 137.2, 137.1, 130.8, 129.8, 129.8, 129.7, 115.2, 115.0, 74.2, 50.7, 34.6, 30.9, 23.4, 21.3. **$^{19}$F NMR** (377 MHz, $CDCl_3$): δ -117.29 – -118.54 (m). **HRMS** *m/z* (+ESI): calc. for $C_{14}H_{18}FN_2$ [M-OAc]: 233.1449; found: 233.1490. **IR** (film) ν [cm$^{-1}$]: 2971, 2934, 2871, 1735, 1600, 1509, 1457, 1371, 1340, 1235, 1158, 1097, 1018, 957, 876, 833, 826, 609, 504, 488, 472, 414.

**(E)-1-(Pyrrolidin-1-ylimino)-4-(4-(trifluoromethyl)phenyl)butan-2-yl acetate (1w):**

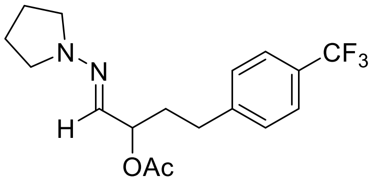


Prepared according to GP3 as clear oil without further purification. **$^1$H NMR** (400 MHz, $CDCl_3$): δ 7.52 (d, *J* = 8.0 Hz, 2H), 7.29 (d, *J* = 8.0 Hz, 2H), 6.30 (d, *J* = 5.5 Hz, 1H), 5.43 – 5.26 (m, 1H), 3.16 (qd, *J* = 5.4, 3.5 Hz, 4H), 2.83 – 2.65 (m, 2H), 2.10 (dq, *J* = 9.4, 7.0 Hz, 2H), 2.05 (s, 3H), 1.95 – 1.83 (m, 4H). **$^{13}$C NMR** (101 MHz, $CDCl_3$): δ 170.4, 145.7, 130.4, 128.8, 128.5, 128.2, 125.4, 125.3, 125.3, 125.2, 74.1, 50.7, 34.2, 31.6, 23.4, 21.3. **$^{19}$F NMR** (377 MHz, $CDCl_3$): δ -62.34. **HRMS** *m/z* (+ESI): calc. for $C_{15}H_{18}N_2O_2F_3$ [M-OAc]: 283.1417; found: 283.1427. **IR** (film) ν [cm$^{-1}$]: 3041, 2972, 2866, 1719, 1618, 1556, 1486, 1459, 1417, 1384, 1372, 1327, 1237, 1187, 1163, 1121, 1067, 1018, 970, 876, 847, 823, 636, 613, 599, 450.

**(E)-4-(4-Methoxyphenyl)-1-(pyrrolidin-1-ylimino)butan-2-yl acetate (1x):**

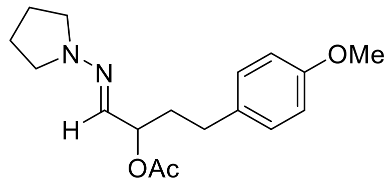


Prepared according to GP3 as clear oil without further purification. **$^1$H NMR** (400 MHz, $CDCl_3$): δ 7.17 – 7.05 (m, 2H), 6.87 – 6.76 (m, 2H), 6.37 – 6.30 (m, 1H), 5.33 (dt, *J* = 7.3, 6.0 Hz, 1H), 3.78 (s, 3H), 3.25 – 3.05 (m, 4H), 2.62 (qdd, *J* = 13.9, 9.3, 6.4 Hz, 2H), 2.10 – 1.98 (m, 5H), 1.97 – 1.83 (m, 4H). **$^{13}$C NMR** (101 MHz, $CDCl_3$): δ 170.5, 157.8, 133.6, 131.2, 129.3, 113.8, 74.3, 55.3, 50.7, 34.8, 30.7, 23.4, 21.4. **HRMS** *m/z* (+ESI): calc. for $C_{15}H_{21}N_2O$ [M-OAc]: 245.1648; found: 245.1700. **IR** (film) ν [$cm^{-1}$]: 3028, 2953, 2935, 2873, 2835, 1718, 1611, 1583, 1557, 1512, 1462, 1442, 1384, 1371, 1340, 1300, 1247, 1177, 1109, 1036, 1012, 970, 877, 822, 614.

**(E)-4-(4-Cyanophenyl)-1-(pyrrolidin-1-ylimino)butan-2-yl acetate (1y):**

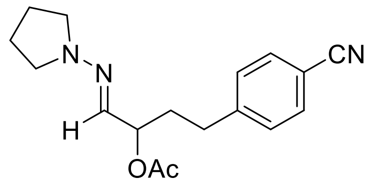


Prepared according to GP3 as clear oil without further purification. **$^1$H NMR** (400 MHz, $CDCl_3$): δ 7.60 – 7.51 (m, 2H), 7.34 – 7.23 (m, 2H), 6.36 – 6.22 (m, 1H), 5.41 – 5.25 (m, 1H), 3.21 – 3.12 (m, 4H), 2.83 – 2.65 (m, 2H), 2.25 – 1.99 (m, 5H), 1.89 (br-quin, *J* = 3.8 Hz, 4H). **$^{13}$C NMR** (101 MHz, $CDCl_3$): δ 170.4, 147.3, 132.2, 130.1, 129.3, 119.1, 109.8, 73.9, 50.6, 33.9, 31.9, 23.4, 21.3. **HRMS** *m/z* (+ESI): calc. for $C_{15}H_{18}N_3$ [M-OAc]: 240.1495; found: 240.1553. **IR** (film) ν [$cm^{-1}$]: 2970, 2935, 2872, 2226, 1718, 1606, 1550, 1505, 1458, 1414, 1384, 1372, 1340, 1248, 1177, 1110, 1019, 971, 876, 849, 822, 665, 615, 556, 450.

## General procedure for the *α*-substitution reaction (GP4)

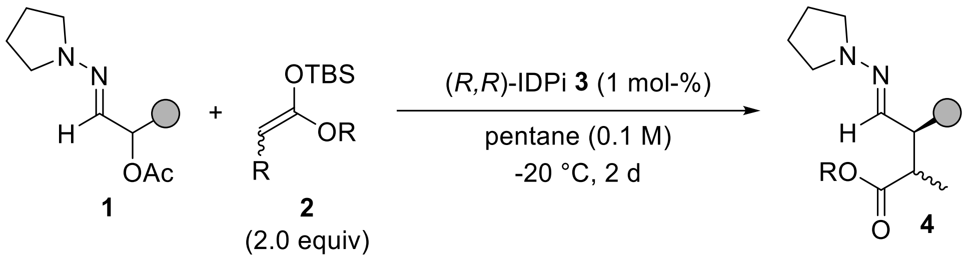


An oven-dried screw cap vial was charged with a stirring bar, the IDPi **3h** (1 mol%, 4.9 mg), and 2 mL of pentane. After the addition of the silyl nucleophile **2a** (2.0 equiv., 0.4 mmol, 88.3 µL), the mixture was stirred for 5 minutes at room temperature. The mixture was then cooled to -20 °C and stirred for an additional 5 minutes before the corresponding hydrazone **1** was added (1.0 eq., 0.2 mmol). Upon completion of the reaction, as monitored by TLC, the reaction was quenched with minimal amounts of $NEt_3$ and *i*PrOH. The solvent was removed, and the product was purified by column chromatography using a hexane:EtOAc eluent system.

**Methyl (S, E)-3-((pyrrolidin-1-ylimino)methyl)pentanoate (4a):**

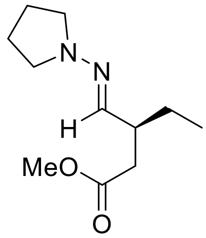


Prepared according to GP4 in a 0.20 mmol scale using catalyst **3h** at -20 °C for 2 d, 41 mg, colorless oil, 98%. **$R_F$** = 0.39 (nHex/EtOAc 4:1). **$^1$H NMR** (400 MHz, $CDCl_3$): δ 6.47 (d, *J* = 5.8 Hz, 1H), 3.64 (s, 3H), 3.13 – 2.99 (m, 4H), 2.65 (br-sex, 6.5 Hz, 1H), 2.50 (dd, *J* = 15.1, 7.7 Hz, 1H), 2.39 (dd, *J* = 15.1, 6.5 Hz, 1H), 1.92 – 1.78 (m, 4H), 1.50 (br-quin, *J* = 7.3 Hz, 2H), 0.91 (t, *J* = 7.4 Hz, 3H). **$^{13}$C NMR** (101 MHz, $CDCl_3$): δ 173.2, 139.7, 51.5, 51.4, 40.5, 37.8, 26.4, 22.9, 11.4. **HRMS** *m/z* (+ESI): calc. for $C_{11}H_{20}N_2O_2Na$ [M+Na]: 235.1417; found: 235.1412. **IR** (film) ν [cm$^{-1}$]: 3458, 2964, 2875, 2820, 1739, 1602, 1460, 1436, 1384, 1338, 1278, 1254, 1191, 1169, 1134, 1096, 1015. The enantiomeric ratio was measured by HPLC analysis using Chiralpak IA, hexane/iPrOH 99/1, flow rate = 1.0 mL/min, λ = 248 nm, 298 K, $t_R$ = 6.6 min (major) and $t_R$ = 7.6 min (minor). **e.r.** = 98:2. $[\boldsymbol{\alpha}]_D^{25}$ = +12.0 (*c* 1.0, $CHCl_3$).

**Methyl (S, E)-3-methyl-4-(pyrrolidin-1-ylimino)butanoate (4b):**

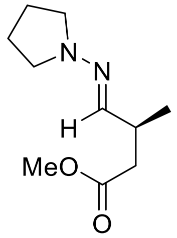


Prepared according to GP4 in a 0.20 mmol scale using catalyst **3h** at -20 °C for 2 d, 38 mg, colorless oil, 96%. **$R_F$** = 0.35 (nHex/EtOAc 4:1). **$^1$H NMR** (400 MHz, $CDCl_3$): δ 6.49 (d, *J* = 5.1 Hz, 1H), 3.66 (s, 3H), 3.22 – 3.00 (m, 4H), 2.84 (br-sepd, *J* = 7.0, 4.9 Hz, 1H), 2.56 (dd, *J* = 15.2, 6.9 Hz, 1H), 2.32 (dd, *J* = 15.2, 7.4 Hz, 1H), 1.98 – 1.73 (m, 4H), 1.11 (d, *J* = 6.9 Hz, 3H). **$^{13}$C NMR** (101 MHz, $CDCl_3$): δ 173.1, 140.3, 51.4, 39.6, 33.8, 23.0, 18.8. **HRMS** *m/z* (+ESI): calc. for $C_{10}H_{19}N_2O_2$ [M+H]: 199.1441; found: 199.1465. **IR** (film) ν [cm$^{-1}$]: 2966, 2874, 2822, 1738, 1602, 1458, 1437, 1384, 1339, 1281, 1254, 1194, 1169, 1105, 1031, 1009, 881, 488. The enantiomeric ratio was measured by HPLC analysis using Chiralpak IA, hexane/iPrOH 99/1, flow rate = 1.0 mL/min, λ = 248 nm, 298 K, $t_R$ = 6.9 min (major) and $t_R$ = 7.7 min (minor). **e.r.** = 97:3. $[\boldsymbol{\alpha}]_D^{25}$ = +3.0 (*c* 1.0, $CHCl_3$).

**Methyl (S, E)-3-((pyrrolidin-1-ylimino)methyl)hexanoate (4c):**

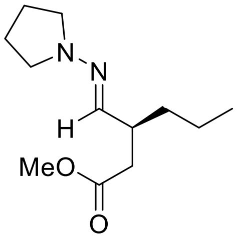

Prepared according to GP4 in a 0.20 mmol scale using catalyst **3h** at -20 °C for 2 d, 44 mg, colorless oil, 97%. **R$_F$** = 0.46 (nHex/EtOAc 4:1). **$^1$H NMR** (400 MHz, $CDCl_3$): δ 6.47 (d, *J* = 5.9 Hz, 1H), 3.65 (s, 3H), 3.13 – 3.03 (m, 4H), 2.73 (br-ddt, *J* = 13.7, 7.6, 6.1 Hz, 1H), 2.50 (dd, *J* = 15.1, 7.7 Hz, 1H), 2.39 (dd, *J* = 15.1, 6.5 Hz, 1H), 1.92 – 1.79 (m, 4H), 1.51 – 1.23 (m, 4H), 0.90 (t, *J* = 7.1 Hz, 3H). **$^{13}$C NMR** (101 MHz, $CDCl_3$): δ 173.1, 140.0, 51.5, 51.4, 38.9, 38.2, 35.7, 22.9, 20.1, 14.1. **HRMS** *m/z* (+ESI): calc. for $C_{12}H_{22}N_2O_2Na$ [M+Na]: 249.1573; found: 249.1602. **IR** (film) ν [cm$^{-1}$]: 2956, 2926, 2871, 2854, 1740, 1603, 1486, 1460, 1436, 1415, 1383, 1359, 1339, 1287, 1248, 1191, 1169, 1136, 1102, 1019, 870, 741, 640, 595, 470, 461, 449. The enantiomeric ratio was measured by HPLC analysis using Chiralpak IA, hexane/iPrOH 99/1, flow rate = 1.0 mL/min, λ = 248 nm, 298 K, $t_R$ = 6.5 min (major) and $t_R$ = 7.3 min (minor). **e.r.** = 97:3. $[\alpha]_D^{25}$ = -2.0 (*c* 1.0, $CHCl_3$).

**Methyl (S, E)-3-((pyrrolidin-1-ylimino)methyl)octanoate (4d):**

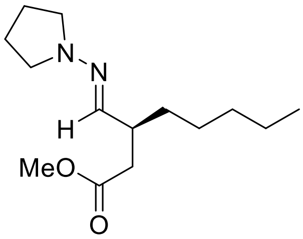

Prepared according to GP4 in a 0.20 mmol scale using catalyst **3h** at -20 °C for 2 d, 49 mg, colorless oil, 96%. **R$_F$** = 0.48 (nHex/EtOAc 4:1). **$^1$H NMR** (400 MHz, $CDCl_3$): δ 6.47 (d, *J* = 5.9 Hz, 1H), 3.64 (s, 3H), 3.14 – 2.97 (m, 4H), 2.71 (br-ddt, *J* = 13.8, 7.5, 6.3 Hz, 1H), 2.50 (dd, *J* = 15.1, 7.7 Hz, 1H), 2.39 (dd, *J* = 15.1, 6.5 Hz, 1H), 1.92 – 1.79 (m, 4H), 1.50 – 1.38 (m, 2H), 1.36 – 1.18 (m, 6H), 0.94 – 0.78 (m, 3H). **$^{13}$C NMR** (101 MHz, $CDCl_3$): δ 173.1, 140.1, 51.6, 51.4, 39.1, 38.2, 33.5, 31.9, 26.6, 22.9, 22.5, 14.0. **HRMS** *m/z* (+ESI): calc. for $C_{14}H_{26}N_2O_2Na$ [M+Na]: 277.1886; found: 277.1915. **IR** (film) ν [cm$^{-1}$]: 2954, 2925, 2871, 2855, 1740, 1603, 1460, 1436, 1415, 1377, 1339, 1260, 1200, 1164, 1136, 1110, 1018, 877, 725. The enantiomeric ratio was measured by HPLC analysis using Chiralpak IA, hexane/iPrOH 99/1, flow rate = 1.0 mL/min, λ = 248 nm, 298 K, $t_R$ = 6.6 min (major) and $t_R$ = 7.9 min (minor). **e.r.** = 97:3. $[\alpha]_D^{25}$ = -6.0 (*c* 1.0, $CHCl_3$).

**Methyl (S, E)-3-((pyrrolidin-1-ylimino)methyl)undecanoate (4e):**

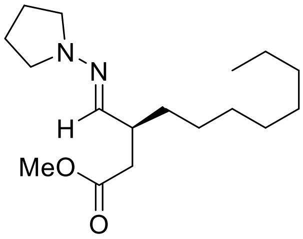

Prepared according to GP4 in a 0.20 mmol scale using catalyst **3h** at -20 °C for 2 d, 58 mg, colorless oil, 98%. **R$_F$** = 0.56 (nHex/EtOAc 4:1). Prepared according to GP4a using catalyst **3h** at -20 °C for 2 d, 49 mg, colorless oil, 96%. **$^1$H NMR** (400 MHz, $CDCl_3$): δ 6.47 (d, *J* = 5.8 Hz, 1H), 3.65 (s, 3H), 3.18 – 2.93 (m, 4H), 2.79 – 2.63 (m, 1H), 2.50 (dd, *J* = 15.1, 7.7 Hz, 1H), 2.39 (dd, *J* = 15.1, 6.4 Hz, 1H), 1.92 – 1.80 (m, 4H), 1.48 – 1.38 (m, 2H), 1.37 – 1.16 (m, 12H), 0.87 (t, *J* = 6.8 Hz, 3H). **$^{13}$C NMR** (101 MHz, $CDCl_3$): δ 173.1, 140.1, 51.6, 51.4, 39.1, 38.3, 33.5, 31.9, 29.7, 29.5, 29.3, 26.9, 22.9, 22.7, 14.1. **HRMS** *m/z* (+ESI): calc. for $C_{17}H_{32}N_2O_2Na$ [M+Na]: 319.2356; found: 319.2362. **IR** (film) ν [cm$^{-1}$]: 3457, 2953, 2925, 2854, 2039, 1834, 1740, 1602, 1458, 1437, 1383, 1339, 1229, 1195, 1162, 1117, 1017, 876, 840, 722, 667, 643, 607, 486, 468, 455. The enantiomeric ratio was measured by HPLC analysis using Chiralpak IA, hexane/iPrOH 99/1, flow rate = 1.0 mL/min, λ = 248 nm, 298 K, $t_R$ = 5.9 min (major) and $t_R$ = 6.8 min (minor). **e.r.** = 97:3. $[\alpha]_D^{25}$ = -6.0 (*c* 1.0, $CHCl_3$).

**Methyl (S, E)-4-methyl-3-((pyrrolidin-1-ylimino)methyl)pentanoate (4f):**

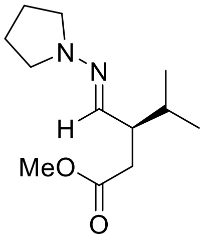

Prepared according to GP4 in a 0.20 mmol scale using catalyst **3h** at -20 °C for 2 d, 43 mg, colorless oil, 95%. **$R_F$** = 0.45 (nHex/EtOAc 4:1). **$^1$H NMR** (400 MHz, $CDCl_3$): δ 6.51 (d, *J* = 5.7 Hz, 1H), 3.64 (s, 3H), 3.11 – 3.04 (m, 4H), 2.67 – 2.48 (m, 2H), 2.41 (dd, *J* = 14.7, 5.2 Hz, 1H), 1.90 – 1.75 (m, 5H), 0.92 (t, *J* = 6.5 Hz, 6H). **$^{13}$C NMR** (101 MHz, $CDCl_3$): δ 173.5, 138.7, 51.7, 51.4, 45.0, 35.2, 30.9, 22.9, 20.0, 19.4. **HRMS** *m/z* (+ESI): calc. for $C_{12}H_{22}N_2O_2Na$ [M+Na]: 249.1573; found: 249.1566. **IR** (film) ν [cm$^{-1}$]: 2959, 2873, 2826, 1739, 1601, 1486, 1461, 1436, 1385, 1369, 1339, 1293, 1258, 1225, 1193, 1167, 1106, 1018, 995, 888. The enantiomeric ratio was measured by HPLC analysis using Chiralpak IA, hexane/iPrOH 99/1, flow rate = 1.0 mL/min, λ = 248 nm, 298 K, $t_R$ = 6.6 min (major) and $t_R$ = 8.0 min (minor). **e.r.** = 96:4. $[\alpha]_D^{25}$ = +10.0 (*c* 1.0, $CHCl_3$).

**Methyl (S, E)-5-methyl-3-((pyrrolidin-1-ylimino)methyl)hexanoate (4g):**

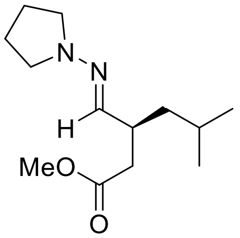

Prepared according to GP4 in a 0.20 mmol scale using catalyst **3h** at -20 °C for 2 d, 46 mg, colorless oil, 96%. **$R_F$** = 0.49 (nHex/EtOAc 4:1). **$^1$H NMR** (400 MHz, $CDCl_3$): δ 6.45 (d, *J* = 6.1 Hz, 1H), 3.64 (s, 3H), 3.12 – 3.03 (m, 4H), 2.79 (br-ddq, *J* = 9.0, 7.7, 6.1 Hz, 1H), 2.46 (dd, *J* = 15.1, 7.7 Hz, 1H), 2.37 (dd, *J* = 15.1, 6.4 Hz, 1H), 1.92 – 1.81 (m, 4H), 1.71 – 1.55 (m, 1H), 1.39 (ddd, *J* = 13.6, 9.0, 5.8 Hz, 1H), 1.23 (ddd, *J* = 13.8, 8.3, 5.9 Hz, 1H), 0.90 (d, *J* = 6.7, 3H), 0.89 (d, *J* = 6.7, 3H). **$^{13}$C NMR** (101 MHz, $CDCl_3$): δ 173.0, 140.1, 51.5, 51.4, 42.8, 38.8, 37.1, 25.5, 23.2, 22.9, 22.2. **HRMS** *m/z* (+ESI): calc. for $C_{13}H_{24}N_2O_2Na$ [M+Na]: 263.1730; found: 263.1717. **IR** (film) ν [cm$^{-1}$]: 2954, 2871, 2840, 2817, 1739, 1602, 1462, 1436, 1385, 1367, 1339, 1270, 1247, 1189, 1169, 1114, 1027, 880, 422, 414. The enantiomeric ratio was measured by HPLC analysis using Chiralpak IF, hexane/iPrOH 99/1, flow rate = 1.0 mL/min, λ = 248 nm, 298 K, $t_R$ = 11.7 min (major) and $t_R$ = 24.6 min (minor). **e.r.** = 95:5. $[\alpha]_D^{25}$ = +2.0 (*c* 1.0, $CHCl_3$).

**Methyl (S, E)-5-methoxy-3-((pyrrolidin-1-ylimino)methyl)pentanoate (4h):**

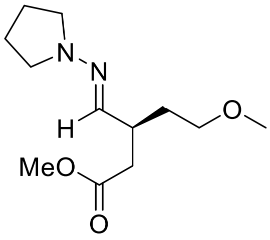

Prepared according to GP4 in a 0.20 mmol scale using catalyst **3h** at -20 °C for 2 d, 48 mg, colorless oil, 99%. **$R_F$** = 0.20 (nHex/EtOAc 4:1). **$^1$H NMR** (400 MHz, $CDCl_3$): δ 6.49 (d, *J* = 5.6 Hz, 1H), 3.64 (s, 3H), 3.41 (td, *J* = 6.7, 0.9 Hz, 2H), 3.30 (s, 3H), 3.14 – 2.98 (m, 4H), 2.85 (br-ddt, *J* = 13.8, 7.7, 6.1 Hz, 1H), 2.54 (dd, *J* = 15.4, 7.6 Hz, 1H), 2.43 (dd, *J* = 15.4, 6.5 Hz, 1H), 1.96 – 1.81 (m, 4H), 1.81 – 1.70 (m, 2H). **$^{13}$C NMR** (101 MHz, $CDCl_3$): δ 172.9, 138.9, 70.5, 58.5, 51.5, 51.4, 38.2, 36.3, 33.1, 22.9. **HRMS** *m/z* (+ESI): calc. for $C_{12}H_{22}N_2O_3Na$ [M+Na]: 265.1523; found: 265.1520. **IR** (film) ν [cm$^{-1}$]: 2951, 2927, 2872, 2829, 1738, 1601, 1483, 1460, 1437, 1385, 1373, 1340, 1259, 1196, 1164, 1118, 1012, 597, 459, 449, 403. The enantiomeric ratio was measured by HPLC analysis using Chiralpak IA, hexane/iPrOH 95/5, flow rate = 1.0 mL/min, λ = 248 nm, 298 K, $t_R$ = 7.4 min (major) and $t_R$ = 8.9 min (minor). **e.r.** = 95:5. $[\alpha]_D^{25}$ = -18.0 (*c* 1.0, $CHCl_3$).

**Methyl (S, E)-6-(benzyloxy)-3-((pyrrolidin-1-ylimino)methyl)hexanoate (4i):**

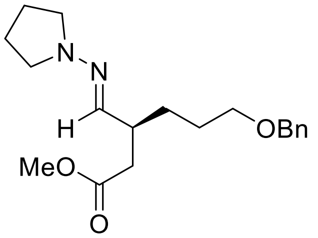


Prepared according to GP4 in a 0.20 mmol scale using catalyst **3h** at -20 °C for 2 d, 55 mg, colorless oil, 83%. **R$_F$** = 0.23 (nHex/EtOAc 4:1). **$^1$H NMR** (400 MHz, $CDCl_3$): δ 7.38 – 7.22 (m, 5H), 6.45 (d, *J* = 5.8 Hz, 1H), 4.49 (s, 2H), 3.64 (s, 3H), 3.47 (t, *J* = 6.4 Hz, 2H), 3.12 – 3.03 (m, 4H), 2.74 (br-sex, *J* = 6.7 Hz, 1H), 2.52 (dd, *J* = 15.2, 7.6 Hz, 1H), 2.40 (dd, *J* = 15.2, 6.5 Hz, 1H), 1.92 – 1.80 (m, 4H), 1.75 – 1.50 (m, 4H). **$^{13}$C NMR** (101 MHz, $CDCl_3$): δ 172.9, 139.1, 138.6, 128.3, 127.6, 127.5, 72.9, 70.3, 51.5, 51.4, 38.9, 38.3, 30.0, 27.2, 22.9. **HRMS** *m/z* (+ESI): calc. for $C_{19}H_{28}N_2O_3Na$ [M+Na]: 355.1992; found: 355.1989. **IR** (film) ν [cm$^{-1}$]: 2947, 2855, 1738, 1454, 1436, 1384, 1362, 1340, 1256, 1202, 1163, 1103, 1028, 738, 698, 464. The enantiomeric ratio was measured by HPLC analysis using Chiralpak IA, hexane/iPrOH 98/2, flow rate = 1.0 mL/min, λ = 248 nm, 298 K, $t_R$ = 11.4 min (major) and $t_R$ = 13.0 min (minor). **e.r.** = 94:6. $[\alpha]_D^{25}$ = -6.0 (*c* 1.0, $CHCl_3$).

**Methyl (S, E)-5-((tert-butyldimethylsilyl)oxy)-3-((pyrrolidin-1-ylimino)methyl) pentanoate (4j):**

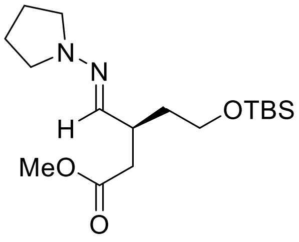


Prepared according to GP4 in a 0.20 mmol scale using catalyst **3g** at -30 °C for 5 d, 50 mg, colorless oil, 73%. **R$_F$** = 0.59 (nHex/EtOAc 4:1). **$^1$H NMR** (400 MHz, $CDCl_3$): δ 6.51 (d, *J* = 5.5 Hz, 1H), 3.81 – 3.52 (m, 5H), 3.08 (br-sep, *J* = 3.9 Hz, 4H), 2.91 – 2.79 (m, 1H), 2.54 (dd, *J* = 15.3, 7.7 Hz, 1H), 2.44 (dd, *J* = 15.3, 6.4 Hz, 1H), 1.91 – 1.78 (m, 4H), 1.77 – 1.65 (m, 2H), 0.88 (s, 9H), 0.03 (s, 6H). **$^{13}$C NMR** (101 MHz, $CDCl_3$): δ 172.9, 139.1, 61.1, 51.4, 51.4, 38.2, 36.2, 36.0, 25.9, 22.9, 18.3, -5.3. **HRMS** *m/z* (+ESI): calc. for $C_{17}H_{34}N_2O_3SiNa$ [M+Na]: 365.2231; found: 365.2217. **IR** (film) ν [cm$^{-1}$]: 2953, 2929, 2896, 2883, 2857, 1740, 1471, 1462, 1436, 1386, 1361, 1339, 1255, 1165, 1099, 1007, 940, 880, 837, 811, 776, 663. The enantiomeric ratio was measured by HPLC analysis using Chiralpak IA, hexane/iPrOH 99/1, flow rate = 1.0 mL/min, λ = 248 nm, 298 K, $t_R$ = 5.1 min (major) and $t_R$ = 6.0 min (minor). **e.r.** = 94:6. $[\alpha]_D^{25}$ = -8.0 (*c* 1.0, $CHCl_3$).

**Methyl (S, E)-3-((pyrrolidin-1-ylimino)methyl)hept-6-enoate (4k):**

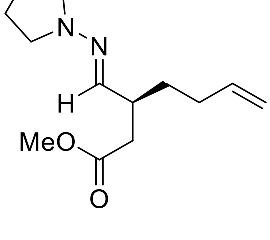


Prepared according to GP4 in a 0.20 mmol scale using catalyst **3h** at -20 °C for 2 d, 46 mg, colorless oil, 97%. **R$_F$** = 0.45 (nHex/EtOAc 4:1). **$^1$H NMR** (400 MHz, $CDCl_3$): δ 6.47 (d, *J* = 5.8 Hz, 1H), 5.80 (ddt, *J* = 16.9, 10.2, 6.6 Hz, 1H), 5.01 (ddt, *J* = 17.1, 1.7, 1.7 Hz, 1H), 4.95 (ddt, *J* = 10.2, 2.2, 1.3 Hz, 1H), 3.65 (s, 3H), 3.15 – 3.03 (m, 4H), 2.75 (br-ddt, *J* = 13.9, 7.7, 6.2 Hz, 1H), 2.52 (dd, *J* = 15.2, 7.6 Hz, 1H), 2.41 (dd, *J* = 15.2, 6.5 Hz, 1H), 2.09 (br-dtt, *J* = 14.7, 6.9, 1.4 Hz, 2H), 1.93 – 1.78 (m, 4H), 1.66 – 1.49 (m, 2H). **$^{13}$C NMR** (101 MHz, $CDCl_3$): δ 172.9, 139.2, 138.3, 114.8, 51.5, 51.4, 38.6, 38.2, 32.7, 31.2, 22.9. **HRMS** *m/z* (+ESI): calc. for $C_{13}H_{22}N_2O_2Na$ [M+Na]: 261.1573; found: 261.1564. **IR** (film) ν [cm$^{-1}$]: 2951, 2926, 2875, 2851, 1739, 1640, 1454, 1436, 1384, 1339, 1254, 1202, 1163, 995, 910, 639, 603, 454. The enantiomeric ratio was measured by HPLC analysis using Chiralpak IF, hexane/iPrOH 99/1, flow rate = 1.0 mL/min, λ = 248 nm, 298 K, $t_R$ = 27.4 min (major) and $t_R$ = 36.2 min (minor). **e.r.** = 96:4. $[\alpha]_D^{25}$ = -7.0 (*c* 1.0, $CHCl_3$).

**Methyl (S, E)-3-((pyrrolidin-1-ylimino)methyl)dodec-11-enoate (4l):**

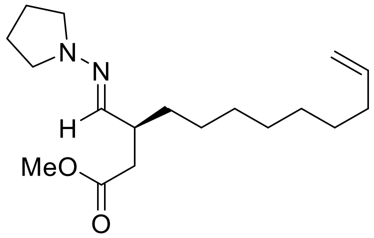


Prepared according to GP4 in a 0.20 mmol scale using catalyst **3h** at -20 °C for 2 d, 60 mg, colorless oil, 97%. **$R_F$** = 0.50 (nHex/EtOAc 4:1). **$^1$H NMR** (400 MHz, $CDCl_3$): δ 6.50 (d, *J* = 5.9 Hz, 1H), 5.83 (ddt, *J* = 16.9, 10.1, 6.7 Hz, 1H), 5.01 (ddt, *J* = 17.1, 1.7, 1.7 Hz, 1H), 4.94 (ddt, *J* = 10.2, 2.3, 1.2 Hz, 1H), 3.67 (s, 3H), 3.15 – 3.06 (m, 4H), 2.73 (br-ddt, *J* = 13.7, 7.5, 6.3 Hz, 1H), 2.52 (dd, *J* = 15.1, 7.6 Hz, 1H), 2.41 (dd, *J* = 15.1, 6.5 Hz, 1H), 2.10 – 2.00 (m, 2H), 1.95 – 1.82 (m, 4H), 1.57 – 1.20 (m, 12H). **$^{13}$C NMR** (101 MHz, $CDCl_3$): δ 173.1, 140.0, 139.2, 114.1, 51.6, 51.4, 39.1, 38.3, 33.8, 33.5, 29.6, 29.3, 29.1, 28.9, 26.9, 22.9. **HRMS** *m/z* (+ESI): calc. for $C_{18}H_{32}N_2O_2Na$ [M+Na]: 331.2356; found: 331.2347. **IR** (film) ν [$cm^{-1}$]: 3456, 3076, 2969, 2926, 2853, 2821, 1739, 1640, 1603, 1459, 1436, 1415, 1384, 1339, 1255, 1196, 1162, 1126, 1102, 1084, 994, 908, 879, 435, 425, 412. The enantiomeric ratio was measured by HPLC analysis using Chiralpak IA, hexane/iPrOH 99/1, flow rate = 1.0 mL/min, λ = 248 nm, 298 K, $t_R$ = 6.2 min (major) and $t_R$ = 7.3 min (minor). **e.r.** = 96:4. $[\alpha]_D^{25}$ = -4.0 (*c* 1.0, $CHCl_3$).

**Methyl (S, E)-3-((pyrrolidin-1-ylimino)methyl)oct-7-ynoate (4m):**

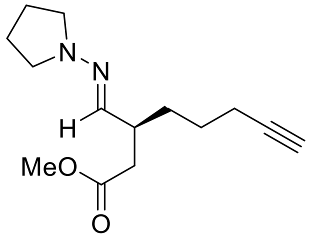


Prepared according to GP4 in a 0.20 mmol scale using catalyst **3h** at -20 °C for 2 d, 49 mg, colorless oil, 97%. **$R_F$** = 0.63 (nHex/EtOAc 4:1). **$^1$H NMR** (400 MHz, $CDCl_3$): δ 6.44 (d, *J* = 5.9 Hz, 1H), 3.65 (s, 3H), 3.12 – 3.03 (m, 4H), 2.80 – 2.67 (m, 1H), 2.52 (dd, *J* = 15.1, 7.6 Hz, 1H), 2.40 (dd, *J* = 15.2, 6.5 Hz, 1H), 2.25 – 2.15 (m, 2H) 1.93 (t, *J* = 2.6 Hz, 1H), 1.89 – 1.82 (m, 4H), 1.64 – 1.48 (m, 4H). **$^{13}$C NMR** (101 MHz, $CDCl_3$): δ 172.9, 138.9, 84.2, 68.4, 51.5, 51.4, 38.7, 38.3, 32.5, 25.9, 22.9, 18.4. **HRMS** *m/z* (+ESI): calc. for $C_{14}H_{22}N_2O_2Na$ [M+Na]: 273.1573; found: 273.1573. **IR** (film) ν [$cm^{-1}$]: 3291, 2949, 2867, 1737, 1601, 1458, 1436, 1384, 1362, 1340, 1263, 1196, 1147, 1107, 1014, 638, 447. The enantiomeric ratio was measured by HPLC analysis using Chiralpak IA, hexane/iPrOH 98/2, flow rate = 1.0 mL/min, λ = 248 nm, 298 K, $t_R$ = 8.7 min (major) and $t_R$ = 10.0 min (minor). **e.r.** = 94:6. $[\alpha]_D^{25}$ = -1.0 (*c* 1.0, $CHCl_3$).

**Methyl (R, E)-5-fluoro-3-((pyrrolidin-1-ylimino)methyl)pentanoate (4n):**

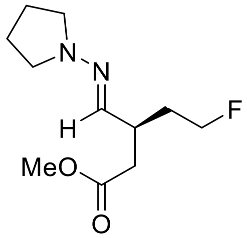


Prepared according to GP4 in a 0.20 mmol scale using catalyst **3g** at -30 °C for 5 d, 35 mg, colorless oil, 76%. **$R_F$** = 0.29 (nHex/EtOAc 4:1). **$^1$H NMR** (400 MHz, $CDCl_3$): δ 6.49 (d, *J* = 5.4 Hz, 1H), 4.67 – 4.51 (m, 1H), 4.50 – 4.39 (m, 1H), 3.66 (s, 3H), 3.13 – 3.04 (m, 4H), 2.98 – 2.85 (m, 1H), 2.58 (dd, *J* = 15.5, 7.4 Hz, 1H), 2.46 (dd, *J* = 15.5, 6.6 Hz, 1H), 2.06 – 1.77 (m, 6H). **$^{13}$C NMR** (101 MHz, $CDCl_3$): δ 172.7, 137.4, 83.0, 81.4, 51.5, 51.4, 38.1, 35.6, 35.6, 33.8, 33.6, 23.0. **$^{19}$F NMR** (377 MHz, $CDCl_3$): δ -218.06 (tdd, *J* = 47.1, 26.7, 23.8 Hz). **HRMS** *m/z* (+ESI): calc. for $C_{11}H_{29}FN_2O_2Na$ [M+Na]: 253.1323; found: 253.1317. **IR** (film) ν [$cm^{-1}$]: 2966, 2876, 2822, 1738, 1599, 1460, 1437, 1384, 1373, 1340, 1284, 1256, 1209, 1167, 1005, 858, 469, 458. The enantiomeric ratio was measured by HPLC analysis using Chiralpak IA, hexane/iPrOH 99/1, flow rate = 1.0 mL/min, λ = 248 nm, 298 K, $t_R$ = 9.5 min (major) and $t_R$ = 10.8 min (minor). **e.r.** = 84:16. $[\alpha]_D^{25}$ = -4.0 (*c* 1.0, $CHCl_3$).

**Methyl (S, E)-7-chloro-3-((pyrrolidin-1-ylimino)methyl)heptanoate (4o):**

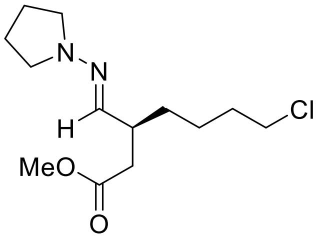


Prepared according to GP4 in a 0.20 mmol scale using catalyst **3h** at -20 °C for 2 d, 54 mg, colorless oil, 98%. **R$_F$** = 0.23 (nHex/EtOAc 4:1). **$^1$H NMR** (400 MHz, $CDCl_3$): δ 6.45 (d, *J* = 5.8 Hz, 1H), 3.65 (s, 3H), 3.52 (t, *J* = 6.7 Hz, 2H), 3.12 – 3.04 (m, 4H), 2.73 (br-sex, *J* = 6.5 Hz, 1H), 2.51 (dd, *J* = 15.2, 7.4 Hz, 1H), 2.39 (dd, *J* = 15.2, 6.7 Hz, 1H), 1.90 – 1.82 (m, 4H), 1.77 (dtd, *J* = 11.5, 6.5, 3.0 Hz, 2H), 1.58 – 1.38 (m, 4H). **$^{13}$C NMR** (101 MHz, $CDCl_3$): δ 172.9, 139.0, 51.5, 51.5, 44.9, 38.9, 38.2, 32.6, 32.5, 24.2, 22.9. **HRMS** *m/z* (+ESI): calc. for $C_{13}H_{23}ClN_2O_2Na$ [M+Na]: 297.1340; found: 297.1367. **IR** (film) ν [cm$^{-1}$]: 3453, 2950, 2865, 1738, 1601, 1486, 1459, 1436, 1384, 1364, 1340, 1284, 1252, 1196, 1167, 1142, 1105, 1015, 876, 739, 726, 650, 594, 489, 469, 450. The enantiomeric ratio was measured by HPLC analysis using Chiralpak IA, hexane/iPrOH 99/1, flow rate = 1.0 mL/min, λ = 248 nm, 298 K, $t_R$ = 10.0 min (major) and $t_R$ = 12.7 min (minor). **e.r.** = 97:3. $[\alpha]_D^{25}$ = -8.0 (*c* 1.0, $CHCl_3$).

**7-Ethyl 1-methyl (S, E)-3-((pyrrolidin-1-ylimino)methyl)heptanedioate (4p):**

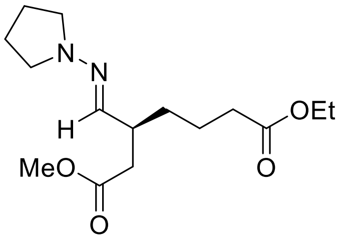


Prepared according to GP4 in a 0.20 mmol scale using catalyst **3h** at -20 °C for 2 d, 55 mg, colorless oil, 92%. **R$_F$** = 0.20 (nHex/EtOAc 4:1). **$^1$H NMR** (400 MHz, $CDCl_3$): δ 6.44 (d, *J* = 5.8 Hz, 1H), 4.11 (q, *J* = 7.1 Hz, 2H), 3.64 (s, 3H), 3.13 – 3.04 (m, 4H), 2.73 (br-ddt, *J* = 13.6, 7.4, 6.1 Hz, 1H), 2.51 (dd, *J* = 15.2, 7.6 Hz, 1H), 2.39 (dd, *J* = 15.2, 6.6 Hz, 1H), 2.29 (td, *J* = 7.4, 2.5 Hz, 2H), 1.91 – 1.76 (m, 4H), 1.75 – 1.58 (m, 2H), 1.55 – 1.40 (m, 2H), 1.24 (t, *J* = 7.1 Hz, 3H). **$^{13}$C NMR** (101 MHz, $CDCl_3$): δ 173.5, 172.9, 138.8, 60.2, 51.5, 51.4, 38.8, 38.2, 34.2, 32.8, 22.9, 22.4, 14.2. **HRMS** *m/z* (+ESI): calc. for $C_{15}H_{26}N_2O_4Na$ [M+Na]: 321.1785; found: 321.1786. **IR** (film) ν [cm$^{-1}$]: 2952, 2872, 2849, 2821, 1737, 1601, 1459, 1437, 1419, 1383, 1374, 1340, 1250, 1176, 1110, 1032, 864, 422, 413. The enantiomeric ratio was measured by HPLC analysis using Chiralpak IA, hexane/iPrOH 98/2, flow rate = 1.0 mL/min, λ = 248 nm, 298 K, $t_R$ = 13.0 min (major) and $t_R$ = 15.2 min (minor). **e.r.** = 96:4. $[\alpha]_D^{25}$ = -4.0 (*c* 1.0, $CHCl_3$).

**Methyl (S, E)-5-phenyl-3-((pyrrolidin-1-ylimino)methyl)pentanoate (4q):**

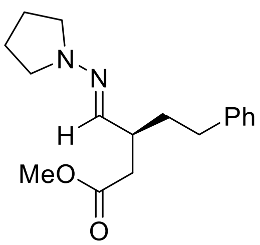


Prepared according to GP4 in a 0.20 mmol scale using catalyst **3h** at -20 °C for 2 d, 55 mg, colorless oil, 95%. **R$_F$** = 0.33 (nHex/EtOAc 4:1). **$^1$H NMR** (400 MHz, $CDCl_3$): δ 7.32 – 7.22 (m, 2H), 7.22 – 7.12 (m, 2H), 6.49 (d, *J* = 5.8 Hz, 1H), 3.65 (s, 3H), 3.14 – 3.04 (m, 4H), 2.80 (br-ddt, *J* = 13.7, 7.6, 6.1 Hz, 1H), 2.66 (br-ddt, *J* = 16.5, 9.4, 7.1 Hz, 2H), 2.56 (dd, *J* = 15.2, 7.6 Hz, 1H), 2.45 (dd, *J* = 15.2, 6.6 Hz, 1H), 2.00 – 1.80 (m, 4H), 1.83 – 1.73 (m, 2H). **$^{13}$C NMR** (101 MHz, $CDCl_3$): δ 172.9, 142.1, 138.9, 128.4, 128.3, 125.8, 51.5, 51.4, 38.9, 38.3, 35.2, 33.3, 22.9. **HRMS** *m/z* (+ESI): calc. for $C_{12}H_{24}N_2O_2Na$ [M+Na]: 311.1730; found: 311.1741. **IR** (film) ν [cm$^{-1}$]: 3026, 2950, 2855, 2821, 1738, 1603, 1496, 1455, 1436, 1339, 1254, 1196, 1146, 1030, 1014, 750, 700, 489, 457. The enantiomeric ratio was measured by HPLC analysis using Chiralpak IA, hexane/iPrOH 98/2, flow rate = 1.0 mL/min, λ = 248 nm, 298 K, $t_R$ = 8.6 min (major) and $t_R$ = 12.1 min (minor). **e.r.** = 95:5. $[\alpha]_D^{25}$ = -12.0 (*c* 1.0, $CHCl_3$).

**Methyl (S, E)-6-phenyl-3-((pyrrolidin-1-ylimino)methyl)hexanoate (4r):**

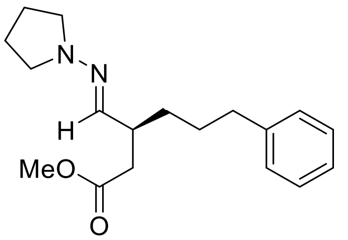

Prepared according to GP4 in a 0.20 mmol scale using catalyst **3h** at -20 °C for 2 d, 58 mg, colorless oil, 96%. **$R_F$** = 0.32 (nHex/EtOAc 4:1). **$^1$H NMR** (400 MHz, $CDCl_3$): δ 7.31 – 7.22 (m, 2H), 7.21 – 7.13 (m, 2H), 6.45 (d, *J* = 5.9 Hz, 1H), 3.64 (s, 3H), 3.13 – 3.03 (m, 4H), 2.76 (br-ddt, *J* = 13.8, 7.5, 6.3 Hz, 1H), 2.60 (br-sep, *J* = 6.7 Hz, 2H), 2.51 (dd, *J* = 15.1, 7.6 Hz, 1H), 2.39 (dd, *J* = 15.1, 6.5 Hz, 1H), 1.93 – 1.83 (m, 4H), 1.75 – 1.59 (m, 2H), 1.58 – 1.45 (m, 2H). **$^{13}$C NMR** (101 MHz, $CDCl_3$): δ 173.0, 142.3, 139.4, 128.4, 128.2, 125.7, 51.5, 51.4, 38.9, 38.2, 35.8, 33.0, 28.7, 22.9. **HRMS** *m/z* (+ESI): calc. for $C_{18}H_{26}N_2O_2Na$ [M+Na]: 325.1886; found: 325.1890. **IR** (film) ν [$cm^{-1}$]: 3025, 2934, 2857, 1738, 1603, 1496, 1454, 1436, 1384, 1361, 1339, 1257, 1196, 1166, 1144, 1105, 1030, 1013, 880, 750, 700, 505. The enantiomeric ratio was measured by HPLC analysis using Chiralpak IA, hexane/iPrOH 99/1, flow rate = 1.0 mL/min, λ = 248 nm, 298 K, $t_R$ = 9.0 min (major) and $t_R$ = 11.1 min (minor). **e.r.** = 96:4. $[\alpha]_D^{25}$ = -10.0 (*c* 1.0, $CHCl_3$).

**Methyl (S, E)-3-((pyrrolidin-1-ylimino)methyl)-5-(thiophen-2-yl)pentanoate (4s):**

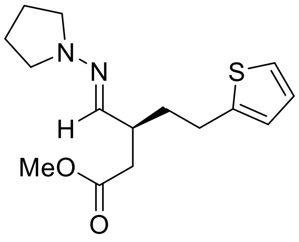

Prepared according to GP4 in a 0.20 mmol scale using catalyst **3h** at -20 °C for 2 d, 36 mg, yellow oil, 61%. **$R_F$** = 0.35 (nHex/EtOAc 4:1). **$^1$H NMR** (400 MHz, $CDCl_3$): δ 7.10 (dd, *J* = 5.1, 1.2 Hz, 1H), 6.90 (dd, *J* = 5.1, 3.4 Hz, 1H), 6.79 (dt, *J* = 3.4, 1.0 Hz, 1H), 6.47 (d, *J* = 5.8 Hz, 1H), 3.66 (s, 3H), 3.13 – 3.05 (m, 4H), 2.95 – 2.77 (m, 3H), 2.56 (dd, *J* = 15.2, 7.5 Hz, 1H), 2.44 (dd, *J* = 15.2, 6.6 Hz, 1H), 1.97 – 1.80 (m, 6H). **$^{13}$C NMR** (101 MHz, $CDCl_3$): δ 172.8, 144.9, 138.3, 126.7, 124.2, 123.0, 51.5, 51.5, 38.7, 38.3, 35.4, 27.5, 23.0. **HRMS** *m/z* (+ESI): calc. for $C_{15}H_{22}N_2O_2SK$ [M+K]: 333.1034; found: 333.1035. **IR** (film) ν [$cm^{-1}$]: 3458, 2949, 2875, 2847, 2821, 1737, 1599, 1437, 1415, 1384, 1364, 1340, 1281, 1253, 1198, 1163, 1103, 1013, 879, 849, 824, 696, 506, 439, 418, 406. The enantiomeric ratio was measured by HPLC analysis using Chiralpak IA, hexane/iPrOH 98/2, flow rate = 1.0 mL/min, λ = 248 nm, 298 K, $t_R$ = 9.6 min (major) and $t_R$ = 13.8 min (minor). **e.r.** = 91:9. $[\alpha]_D^{25}$ = -10.0 (*c* 1.0, $CHCl_3$).

**Methyl (S, E)-5-(4-chlorophenyl)-3-((pyrrolidin-1-ylimino)methyl)pentanoate (4t):**

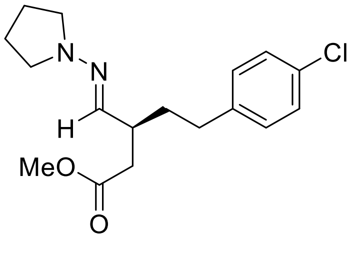

Prepared according to GP4 in a 0.20 mmol scale using catalyst **3h** at -20 °C for 2 d, 58 mg, colorless oil, 90%. **$R_F$** = 0.33 (nHex/EtOAc 4:1). **$^1$H NMR** (400 MHz, $CDCl_3$): δ 7.25 – 7.19 (m, 2H), 7.14 – 7.04 (m, 2H), 6.46 (d, *J* = 5.8 Hz, 1H), 3.65 (s, 3H), 3.20 – 2.95 (m, 4H), 2.83 – 2.72 (m, 1H), 2.62 (br-td, *J* = 9.5, 6.6 Hz, 2H), 2.54 (dd, *J* = 15.2, 7.3 Hz, 1H), 2.43 (dd, *J* = 15.2, 6.7 Hz, 1H), 1.95 – 1.81 (m, 4H), 1.82 – 1.70 (m, 2H). **$^{13}$C NMR** (101 MHz, $CDCl_3$): δ 172.8, 140.6, 138.6, 131.5, 129.8, 128.4, 51.5, 38.8, 38.3, 35.0, 32.7, 23.0. **HRMS** *m/z* (+ESI): calc. for $C_{17}H_{23}ClN_2O_2Na$ [M+Na]: 345.1340; found: 345.1356. **IR** (film) ν [$cm^{-1}$]: 3453, 3024, 2949, 2929, 2857, 1737, 1598, 1492, 1457, 1436, 1408, 1383, 1340, 1254, 1197, 1148, 1092, 1014, 878, 833, 818, 807, 663, 410. The enantiomeric ratio was measured by HPLC analysis using Chiralpak IA, hexane/iPrOH 98/2, flow rate = 1.0 mL/min, λ = 248 nm, 298 K, $t_R$ = 10.5 min (major) and $t_R$ = 12.3 min (minor). **e.r.** = 95:5. $[\alpha]_D^{25}$ = -9.0 (*c* 1.0, $CHCl_3$).

**Methyl (S, E)-5-(4-bromophenyl)-3-((pyrrolidin-1-ylimino)methyl)pentanoate (4u):**

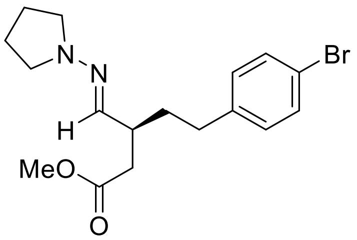

Prepared according to GP4 in a 0.20 mmol scale using catalyst **3h** at -20 °C for 2 d, 66 mg, colorless oil, 90%. **R$_F$** = 0.33 (nHex/EtOAc 4:1). **$^1$H NMR** (400 MHz, $CDCl_3$): δ 7.44 – 7.33 (m, 2H), 7.10 – 7.00 (m, 2H), 6.47 (d, $J$ = 5.8 Hz, 1H), 3.67 (s, 3H), 3.19 – 3.03 (m, 4H), 2.86 – 2.73 (m, 1H), 2.72 – 2.52 (m, 3H), 2.45 (dd, $J$ = 15.2, 6.6 Hz, 1H), 1.99 – 1.84 (m, 4H), 1.84 – 1.75 (m, 2H). **$^{13}$C NMR** (101 MHz, $CDCl_3$): δ 172.8, 141.1, 138.5, 131.4, 130.2, 119.5, 51.5, 51.4, 38.8, 38.3, 35.0, 32.8, 23.0. **HRMS** *m/z* (+ESI): calc. for $C_{17}H_{23}BrN_2O_2Na$ [M+Na]: 389.0835; found: 389.0846. **IR** (film) ν [cm$^{-1}$]: 2949, 2926, 2855, 1737, 1488, 1456, 1436, 1384, 1339, 1251, 1196, 1147, 1110, 1071, 1011, 802, 412. The enantiomeric ratio was measured by HPLC analysis using Chiralpak IA, hexane/iPrOH 98/2, flow rate = 1.0 mL/min, λ = 248 nm, 298 K, $t_R$ = 11.0 min (major) and $t_R$ = 12.7 min (minor). **e.r.** = 95:5. $[\alpha]_D^{25}$ = -14.0 (*c* 1.0, $CHCl_3$).

**Methyl (S, E)-5-(4-fluorophenyl)-3-((pyrrolidin-1-ylimino)methyl)pentanoate (4v):**

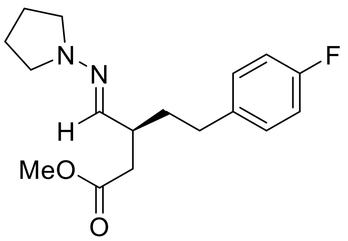

Prepared according to GP4 in a 0.20 mmol scale using catalyst **3h** at -20 °C for 2 d, 60 mg, colorless oil, 98%. **R$_F$** = 0.36 (nHex/EtOAc 4:1). **$^1$H NMR** (400 MHz, $CDCl_3$): δ 7.17 – 7.06 (m, 2H), 7.03 – 6.88 (m, 2H), 6.47 (d, $J$ = 5.8 Hz, 1H), 3.65 (s, 3H), 3.20 – 2.95 (m, 4H), 2.77 (br-dtd, $J$ = 13.4, 7.5, 5.9 Hz, 1H), 2.64 (br-dtd, $J$ = 16.7, 9.5, 6.6 Hz, 2H), 2.55 (dd, $J$ = 15.2, 7.5 Hz, 1H), 2.43 (dd, $J$ = 15.2, 6.6 Hz, 1H), 1.95 – 1.82 (m, 4H), 1.83 – 1.70 (m, 2H). **$^{13}$C NMR** (101 MHz, $CDCl_3$): δ 172.8, 162.5, 160.0, 138.7, 137.7, 137.7, 129.8, 129.7, 115.1, 114.9, 51.5, 51.5, 38.7, 38.3, 35.3, 32.5, 23.0. **$^{19}$F NMR** (377 MHz, $CDCl_3$): δ -117.87 (tt, $J$ = 8.6, 5.2 Hz). **HRMS** *m/z* (+ESI): calc. for $C_{17}H_{23}FN_2O_2Na$ [M+Na]: 329.1636; found: 329.1625. **IR** (film) ν [cm$^{-1}$]: 2950, 2873, 2859, 1737, 1601, 1509, 1457, 1437, 1415, 1384, 1373, 1361, 1340, 1248, 1221, 1200, 1157, 1105, 1015, 878, 826. The enantiomeric ratio was measured by HPLC analysis using Chiralpak IA, hexane/iPrOH 98/2, flow rate = 1.0 mL/min, λ = 248 nm, 298 K, $t_R$ = 9.3 min (major) and $t_R$ = 10.3 min (minor). **e.r.** = 94:6. $[\alpha]_D^{25}$ = -14.0 (*c* 1.0, $CHCl_3$).

**Methyl (S, E)-3-((pyrrolidin-1-ylimino)methyl)-5-(4-(trifluoromethyl)phenyl)pentanoate (4w):**

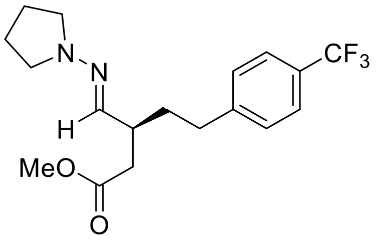

Prepared according to GP4 in a 0.20 mmol scale using catalyst **3h** at -20 °C for 2 d, 65 mg, colorless oil, 91%. **R$_F$** = 0.38 (nHex/EtOAc 4:1). **$^1$H NMR** (400 MHz, $CDCl_3$): δ 7.56 – 7.45 (m, 2H), 7.32 – 7.27 (m, 2H), 6.44 (d, $J$ = 5.8 Hz, 1H), 3.65 (s, 3H), 3.17 – 3.00 (m, 4H), 2.85 – 2.63 (m, 3H), 2.56 (dd, $J$ = 15.2, 7.3 Hz, 1H), 2.44 (dd, $J$ = 15.2, 6.7 Hz, 1H), 1.91 – 1.85 (m, 4H), 1.85 – 1.78 (m, 2H). **$^{13}$C NMR** (101 MHz, $CDCl_3$): δ 173.2, 146.9, 145.8, 141.7, 130.3, 125.3, 125.3, 125.2, 125.2, 51.5, 51.4, 38.8, 38.3, 34.8, 33.2, 23.0. **$^{19}$F NMR** (377 MHz, $CDCl_3$): δ -62.31. **HRMS** *m/z* (+ESI): calc. for $C_{18}H_{23}F_3N_2O_2Na$ [M+Na]: 379.1604; found: 379.1616. **IR** (film) ν [cm$^{-1}$]: 2952, 2874, 2861, 1738, 1618, 1602, 1458, 1437, 1417, 1383, 1327, 1251, 1189, 1162, 1121, 1067, 1018, 879, 841, 823, 636, 613, 598. The enantiomeric ratio was measured by HPLC analysis using Chiralpak IA,

hexane/iPrOH 98/2, flow rate = 1.0 mL/min, λ = 248 nm, 298 K, $t_R$ = 9.3 min (major) and $t_R$ = 10.2 min (minor). **e.r.** = 95:5. $[\alpha]_D^{25}$ = -8.0 (*c* 1.0, $CHCl_3$).

**Methyl (S, E)-5-(4-methoxyphenyl)-3-((pyrrolidin-1-ylimino)methyl)pentanoate (4x):**

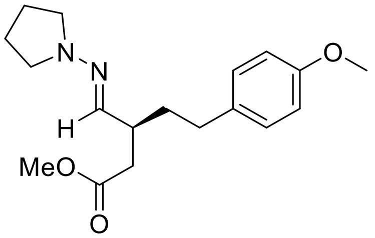

Prepared according to GP4 in a 0.20 mmol scale using catalyst **3h** at -20 °C for 2 d, 55 mg, colorless oil, 86%. **$R_F$** = 0.19 (nHex/EtOAc 4:1). **$^1$H NMR** (400 MHz, $CDCl_3$): δ 7.13 – 7.04 (m, 2H), 6.85 – 6.77 (m, 2H), 6.48 (d, *J* = 5.9 Hz, 1H), 3.78 (s, 3H), 3.65 (s, 3H), 3.13 – 3.02 (m, 4H), 2.85 – 2.72 (m, 1H), 2.69 – 2.49 (m, 3H), 2.44 (dd, *J* = 15.2, 6.5 Hz, 1H), 1.93 – 1.83 (m, 4H), 1.81 – 1.71 (m, 2H). **$^{13}$C NMR** (101 MHz, $CDCl_3$): δ 172.9, 157.8, 139.1, 134.2, 129.3, 113.8, 55.3, 51.5, 51.4, 38.8, 38.3, 35.5, 32.4, 23.0. **HRMS** *m/z* (+ESI): calc. for $C_{18}H_{26}N_2O_3Na$ [M+Na]: 341.1836; found: 341.1834. **IR** (film) ν [$cm^{-1}$]: 2950, 2875, 2834, 1737, 1612, 1584, 1513, 1457, 1438, 1384, 1374, 1339, 1300, 1247, 1197, 1177, 1146, 1111, 1037, 879, 822, 752, 525, 407. The enantiomeric ratio was measured by HPLC analysis using Chiralpak IA, hexane/iPrOH 99/1, flow rate = 1.0 mL/min, λ = 248 nm, 298 K, $t_R$ = 15.6 min (major) and $t_R$ = 21.6 min (minor). **e.r.** = 91:9. $[\alpha]_D^{25}$ = -14.0 (*c* 1.0, $CHCl_3$).

**Methyl (S, E)-5-(4-cyanophenyl)-3-((pyrrolidin-1-ylimino)methyl)pentanoate (4y):**

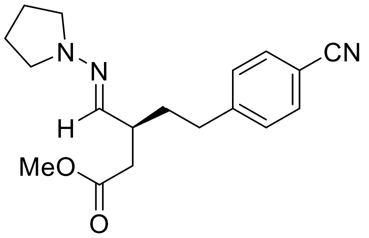

Prepared according to GP4 in a 0.20 mmol scale using catalyst **3h** at -20 °C for 2 d, 59 mg, yellow oil, 94%. **$R_F$** = 0.15 (nHex/EtOAc 4:1). **$^1$H NMR** (400 MHz, $CDCl_3$): δ 7.60 – 7.43 (m, 2H), 7.31 – 7.24 (m, 2H), 6.43 (d, *J* = 5.8 Hz, 1H), 3.65 (s, 3H), 3.15 – 2.97 (m, 4H), 2.83 – 2.62 (m, 3H), 2.55 (dd, *J* = 15.3, 7.2 Hz, 1H), 2.43 (dd, *J* = 15.3, 6.8 Hz, 1H), 1.94 – 1.84 (m, 4H), 1.83 – 1.73 (m, 2H). **$^{13}$C NMR** (101 MHz, $CDCl_3$): δ 172.7, 147.9, 137.8, 132.2, 129.3, 119.1, 109.7, 51.5, 51.4, 38.7, 38.3, 34.5, 33.5, 23.0. **HRMS** *m/z* (+ESI): calc. for $C_{18}H_{23}N_3O_2K$ [M+K]: 352.1422; found: 352.1425. **IR** (film) ν [$cm^{-1}$]: 2951, 2849, 2825, 2226, 1736, 1606, 1505, 1457, 1437, 1415, 1384, 1339, 1254, 1198, 1176, 1149, 1117, 1020, 842, 822, 562. The enantiomeric ratio was measured by HPLC analysis using Chiralpak IA, hexane/iPrOH 95/5, flow rate = 1.0 mL/min, λ = 248 nm, 298 K, $t_R$ = 17.4 min (major) and $t_R$ = 26.3 min (minor). **e.r.** = 90:10. $[\alpha]_D^{25}$ = -14.0 (*c* 1.0, $CHCl_3$).

**Ethyl (2R,3S)-2-methyl-3-((E)-(pyrrolidin-1-ylimino)methyl)pentanoate (*anti*-4ab):**

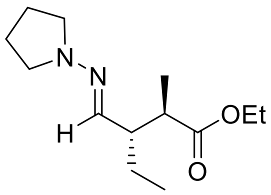

Prepared according to GP4 using hydrazone **1a** (52 mg, 0.26 mmol, 1.0 equiv.), silyl ketene acetal **(*Z*)-2b** (87 mg, 0.4 mmol, 1.5 equiv.) and catalyst **3h** (2 mol%) at -20 °C (4 mL pentane) for 3 d, 59 mg, colorless oil, 94% (50:1 d.r.). **$R_F$** = 0.62 (nHex/EtOAc 7:3). **$^1$H NMR** (400 MHz, $CDCl_3$): δ 6.32 (d, J = 7.3 Hz, 1H), 4.17 – 4.08 (m, 2H), 3.19 – 2.89 (m, 4H), 2.52 (br-dt, *J* = 13.9, 7.1 Hz, 1H), 2.41 (br-dtd, J = 9.4, 7.6, 4.5 Hz, 1H), 2.02 – 1.74 (m, 4H), 1.55 – 1.35 (m, 2H), 1.24 (t, J = 7.1 Hz, 3H), 1.12 (d, J = 7.0 Hz, 3H), 0.89 (t, J = 7.4 Hz, 3H). **$^{13}$C NMR** (101 MHz, $CDCl_3$): δ 175.8, 139.5, 60.3, 51.8, 47.3, 43.2, 24.5, 23.0, 14.7, 14.4, 11.9. **HRMS** *m/z* (+ESI): calc. for $C_{13}H_{24}N_2O_2Na$ [M+Na]: 263.1730; found: 263.1721. **IR** (film) ν [$cm^{-1}$]: 2967, 2934, 2875, 2824, 1733, 1460, 1384, 1338, 1250, 1182, 1154, 1134, 1096. The enantiomeric ratio was measured by HPLC analysis using Chiralcel ODH, hexane/EtOAc 98/2, flow rate = 1.0 mL/min, λ = 248 nm, 298 K, $t_R$ = 9.5 min (major) and $t_R$ = 12.1 min (minor). **e.r.** = 96:04. $[\alpha]_D^{25}$ = +14.0 (*c* 1.0, $CHCl_3$).

**Ethyl (2S,3S)-2-methyl-3-((E)-(pyrrolidin-1-ylimino)methyl)pentanoate (*syn*-4ab):**

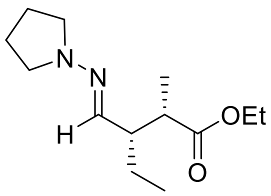

Prepared according to GP4 using hydrazone **1a** (40 mg, 0.2 mmol, 1.0 equiv.), silyl ketene acetal **(*E*)-2b** (87 mg, 0.4 mmol, 2.0 equiv.) and catalyst **3g** (2 mol%) at -20 °C (4 mL pentane) for 3 d, 42 mg, colorless oil, 87% (8:1 d.r.). **$R_F$** = 0.62 (nHex/EtOAc 7:3). **$^1$H NMR** (400 MHz, $CDCl_3$): δ 6.47 (d, *J* = 7.1 Hz, 1H), 4.18 – 4.05 (m, 2H), 3.13 – 3.08 (m, 4H), 2.62 (dt, *J* = 13.3, 6.9 Hz, 1H), 2.41 – 2.30 (m, 1H), 1.93 – 1.80 (m, 4H), 1.63 – 1.37 (m, 2H), 1.24 (t, *J* = 7.1 Hz, 3H), 1.13 (d, *J* = 7.1 Hz, 3H), 0.90 (t, *J* = 7.4 Hz, 3H). **$^{13}$C NMR** (101 MHz, $CDCl_3$): δ 175.8, 139.5, 60.3, 51.8, 47.3, 43.2, 24.5, 23.0, 14.7, 14.4, 11.9. **HRMS** *m/z* (+ESI): calc. for $C_{13}H_{24}N_2O_2Na$ [M+Na]: 263.1730; found: 263.1721. **IR** (film) ν [cm$^{-1}$]: 2967, 2934, 2875, 2824, 1733, 1460, 1384, 1338, 1250, 1182, 1154, 1134, 1096. The enantiomeric ratio was measured by HPLC analysis using Chiralcel ODH, hexane/EtOAc 98/2, flow rate = 1.0 mL/min, λ = 248 nm, 298 K, $t_R$ = 12.4 min (major) and $t_R$ = 10.4 min (minor). **e.r.** = 98:02. $[\alpha]_D^{25}$ = -8.0 (*c* 1.0, $CHCl_3$).

**Methyl (2R,3S)-3-((E)-(pyrrolidin-1-ylimino)methyl)-2-vinylpentanoate (*anti*-4ac):**

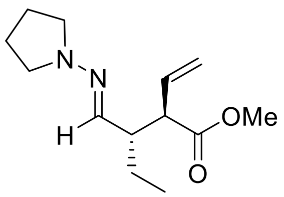

Prepared according to GP4 using using hydrazone **1a** (36 mg, 0.18 mmol, 1.0 equiv.) silyl ketene acetal **(Z)-2c** (78 mg, 0.36 mmol, 2.0 equiv.) and catalyst **3h** (5 mol%) at -20 °C (4 mL pentane) for 3 d, 35 mg, colorless oil, 81% (32:1 d.r.). **$R_F$** = 0.66 (nHex/EtOAc 7:3). **$^1$H NMR** (400 MHz, $CDCl_3$): δ 6.28 (d, J = 7.3 Hz, 1H), 5.84 (ddd, J = 17.1, 10.1, 9.3, Hz, 1H), 5.17 – 5.13 (m, 1H), 5.10 (ddt, *J* = 17.1, 1.6, 0.8 Hz, 1H), 3.68 (s, 3H), 3.21 – 3.00 (m, 5H), 2.65 – 2.55 (m, 1H), 1.95 – 1.79 (m, 4H), 1.53 – 1.39 (m, 2H), 0.91 (t, J = 7.4 Hz, 3H). **$^{13}$C NMR** (101 MHz, $CDCl_3$): δ 173.7, 138.6, 134.5, 118.4, 54.2, 51.9, 51.8, 46.5, 24.7, 23.0, 11.8. **HRMS** *m/z* (+ESI): calc. for $C_{13}H_{23}N_2O_2Na$ [M+H]: 239.1760; found: 239.1762. **IR** (film) ν [cm$^{-1}$]: 2963, 2932, 2875, 2827, 1737, 1460, 1435, 1384, 1337, 1244, 1194, 1157, 1135, 1025, 993, 923. The enantiomeric ratio was measured by HPLC analysis using Chiralcel ODH, hexane/EtOAc 98/2, flow rate = 1.0 mL/min, λ = 248 nm, 298 K, $t_R$ = 13.1 min (major) and $t_R$ = 14.6 min (minor). **e.r.** = 99:1. $[\alpha]_D^{25}$ = +18.0 (*c* 1.0, $CHCl_3$).

# Postmodifications

## One pot hydrolysis of the hydrazone

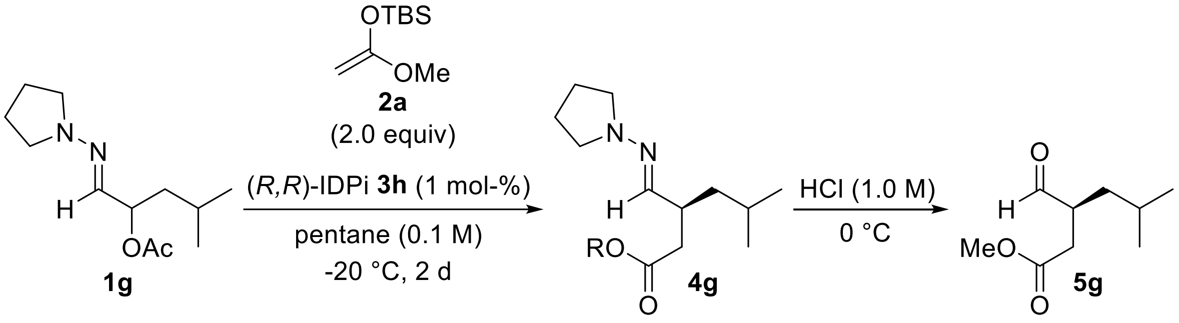


After the asymmetric attack of silyl ketene acetal **2a** following GP4 with catalyst **3h**, the reaction mixture was washed three times with aqueous HCl (1.0 M) at 0 °C. Subsequential evaporation of the solvent and purification by column chromatography using EtOAc:hexanes as eluent lead to the aldehyde **5g** as a colorless oil with 85 % yield (29 mg, **e.r.** = 95:05)

## Ozonolysis[8]

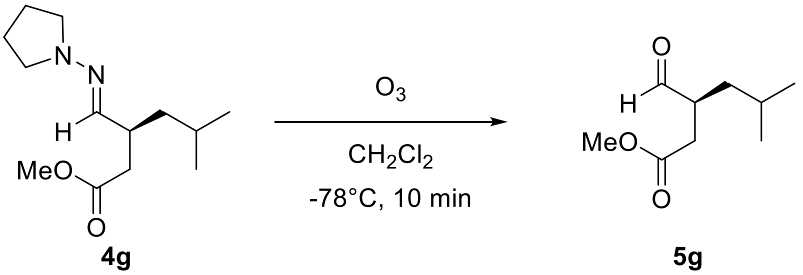


A solution of hydrazone **4g** (240 mg, 1.00 mmol) in $CH_2Cl_2$ (5 mL) was cooled to -78 °C and ozone was bubbled through the solution until a light grey color appeared (10 min). Argon was bubbled through the solution while it was allowed to warm to rt. After evaporation of solvent the crude product was purified by column chromatography using EtOAc:hexanes as eluent. The desired aldehyde **5g** was obtained as a colorless oil with 70 % yield (120 mg).

## Methyl (S)-3-formyl-5-methylhexanoate (5g):

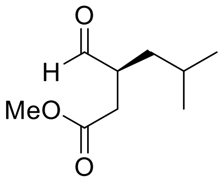

**$R_F$** = 0.28 (nHex/EtOAc 9:1). **$^1$H NMR** (400 MHz, $CDCl_3$): δ $^1$H NMR (400 MHz, $CDCl_3$) δ 9.73 (d, $J$ = 1.2 Hz, 1H), 3.70 (s, 3H), 2.96 – 2.82 (m, 1H), 2.70 (dd, $J$ = 16.6, 8.4 Hz, 1H), 2.43 (dd, $J$ = 16.6, 5.0 Hz, 1H), 1.79 – 1.53 (m, 2H), 1.37 – 1.25 (m, 1H), 0.98 (d, $J$ = 6.4 Hz, 3H), 0.95 (d, $J$ = 6.4 Hz, 3H). **$^{13}$C NMR** (101 MHz, $CDCl_3$): δ 203.1, 172.4, 51.9, 45.9, 37.8, 33.4, 25.7, 22.7, 22.3. **HRMS** *m/z* (+ESI): calc. for $C_9H_{17}O_3$ [M+H]: 173.1172; found: 173.1181. **IR** (film) ν [cm$^{-1}$]: 2958, 2936, 2871, 2848, 2722, 1738, 1469, 1438, 1415, 1386, 1368, 1255, 1225, 1194, 1175, 1116, 1024, 996, 892. The enantiomeric ratio was measured by HPLC analysis using Chiralpak IC, hexane/iPrOH 98/2, flow rate = 1.0 mL/min, λ = 212 nm, 298 K, $t_R$ = 18.8 min (major) and $t_R$ = 22.8 min (minor). **e.r.** = 96:4. $[\alpha]_D^{25}$ = -71.0 (*c* 1.0, $CHCl_3$).

**MMPP Oxidation[9] (GP5)**

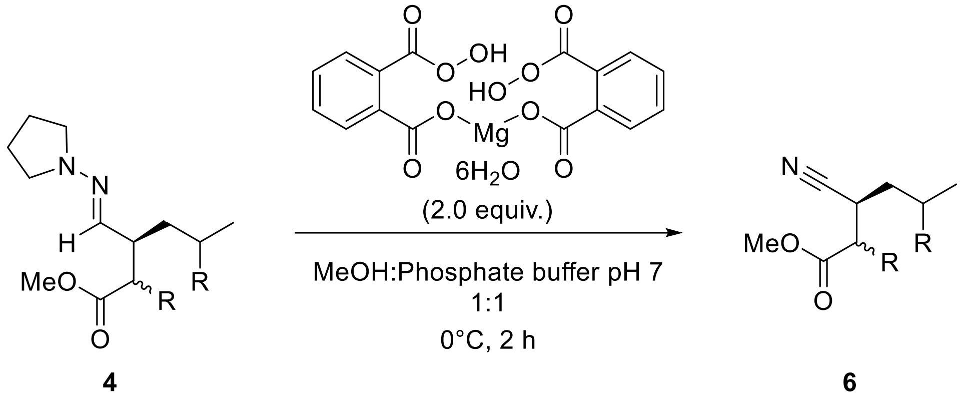

MMPP·6$H_2O$ (2.0 mmol/mmol hydrazone) was suspended in a rapidly stirred mixture of MeOH and pH 7 phosphate buffer (1:1, 5 mL/mmol MMPP each) at 0 °C. The hydrazone was dissolved in MeOH (4 mL/mmol hydrazone) and added dropwise. The mixture was stirred at 0 °C for 2 hours. The suspension was diluted with $Et_2O$ and washed with saturated aqueous $NaHCO_3$ solution. The organic layer was separated and the aqueous phase extracted with $Et_2O$ (3x). The combined organic phases were washed with $H_2O$ and brine and dried over $MgSO_4$. Evaporation and purification by column chromatography (EtOAc:hexanes 15:85) afforded the pure nitrile as a colorless liquid.

**Methyl (S)-3-cyanohexanoate (6c):**

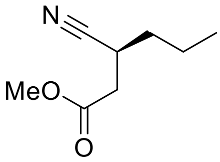

Prepared according to GP5 using hydrazone **4c** (453 mg, 2.0 mmol), 290 mg, colorless oil, 93 %. **R**$_F$ = 0.48 (nHex/EtOAc 4:1). **$^1$H NMR** (400 MHz, $CDCl_3$): δ 3.73 (s, 3H), 3.11 – 2.90 (m, 1H), 2.71 (dd, *J* = 16.6, 7.6 Hz, 1H), 2.55 (dd, *J* = 16.6, 6.8 Hz, 1H), 1.69 – 1.38 (m, 4H), 0.96 (t, *J* = 7.1 Hz, 3H). **$^{13}$C NMR** (101 MHz, $CDCl_3$): δ 170.3, 121.1, 52.2, 36.6, 33.8, 27.4, 20.2, 13.4. **HRMS** *m/z* (+ESI): calc. for $C_8H_{13}NO_2Na$ [M+Na]: 178.0838; found: 178.0847. **IR** (film) ν [cm$^{-1}$]: 2961, 2937, 2876, 2243, 1741, 1466, 1439, 1419, 1368, 1308, 1259, 1213, 1176, 1104, 1016, 988, 742, 405. $[\alpha]_D^{25}$ = -2.0 (*c* 1.0, $CHCl_3$).

**Methyl (S)-3-cyano-5-methylhexanoate (6g):**

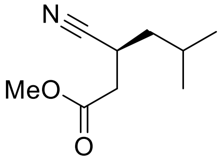

Prepared according to GP5 using hydrazone **4g** (481 mg, 2.0 mmol), 320 mg, colorless oil, 95 %. **R**$_F$ = 0.53 (nHex/EtOAc 4:1). **$^1$H NMR** (400 MHz, $CDCl_3$): δ 3.74 (s, 3H), 3.05 (dddd, *J* = 10.7, 7.6, 6.7, 5.1 Hz, 1H), 2.70 (dd, *J* = 16.6, 7.6 Hz, 1H), 2.54 (dd, *J* = 16.6, 6.7 Hz, 1H), 1.86 (dqqd, *J* = 9.4, 6.6, 6.6, 4.8 Hz, 1H), 1.64 (ddd, *J* = 13.5, 10.7, 4.8 Hz, 1H), 1.33 (ddd, *J* = 13.5, 9.4, 5.1 Hz, 1H), 0.97 (d, *J* = 4.8 Hz, 3H), 0.96 (d, *J* = 6.6 Hz, 3H). **$^{13}$C NMR** (101 MHz, $CDCl_3$): δ 170.2, 121.1, 52.2, 40.8, 37.0, 26.1, 25.8, 22.9, 21.2. **HRMS** *m/z* (+ESI): calc. for $C_9H_{19}N_2O_2$ [M+NH$_4$]: 187.1441; found: 187.1444. **IR** (film) ν [cm$^{-1}$]: 2959, 2938, 2873, 2242, 1742, 1470, 1457, 1439, 1385, 1370, 1265, 1226, 1213, 1174, 1143, 1114, 990, 418. $[\alpha]_D^{25}$ = -12.0 (*c* 1.0, $CHCl_3$).

**Ethyl (2R,3S)-3-cyano-2-methylpentanoate (*anti*-5ab):**

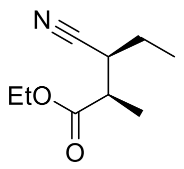


Prepared according to GP4 using hydrazone *anti*-**4ab** (390 mg, 1.6 mmol), 231 mg of the pure diastereomer, colorless oil, 84 %. **$R_F$** = 0.56 (nHex/EtOAc 4:1). **$^1$H NMR** (400 MHz, $CDCl_3$): δ 4.18 (q, *J* = 7.1 Hz, 2H), 2.90 (td, *J* = 8.1, 6.3 Hz, 1H), 2.61 (br-quin, *J* = 7.2 Hz, 1H), 1.67 – 1.51 (m, 2H), 1.37 (d, J = 7.1 Hz, 3H), 1.27 (t, *J* = 7.1 Hz, 3H), 1.11 (t, *J* = 7.4 Hz, 3H). **$^{13}$C NMR** (101 MHz, $CDCl_3$): δ 173.4, 120.2, 61.4, 41.3, 36.7, 24.3, 15.4, 14.3, 11.8. **HRMS** *m/z* (+ESI): calc. for $C_9H_{15}NO_2Na$ [M+Na]: 192.0995; found: 192.0994. **IR** (film) ν [$cm^{-1}$]: 2979, 2941, 2882, 1735, 1463, 1384, 1372, 1245, 1185, 1095. $[\alpha]_D^{25}$ = -6.0 (*c* 1.0, $CHCl_3$).

**Ethyl (2S,3S)-3-cyano-2-methylpentanoate (*syn*-5ab):**

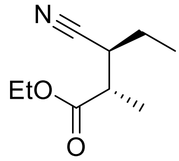


Prepared according to GP4 using hydrazone *syn*-**4ab** (345 mg, 1.4 mmol), 156 mg of the pure diastereomer, colorless oil, 64 %. **$R_F$** = 0.52 (nHex/EtOAc 4:1). **$^1$H NMR** (400 MHz, $CDCl_3$): δ 4.19 (q, *J* = 7.1 Hz, 2H), 2.88 – 2.66 (m, 2H), 1.74 – 1.52 (m, 2H), 1.38 – 1.22 (m, 6H), 1.10 (t, *J* = 7.4 Hz, 3H).**$^{13}$C NMR** (101 MHz, $CDCl_3$): δ 173.4, 120.2, 61.4, 41.3, 36.7, 24.3, 15.4, 14.3, 11.8. **HRMS** *m/z* (+ESI): calc. for $C_9H_{15}NO_2Na$ [M+Na]: 192.0995; found: 192.0996. **IR** (film) ν [$cm^{-1}$]: 2979, 2940, 2881, 2240, 1736, 1463, 1384, 1372, 1344, 1297, 1264, 1187, 1153, 1093, 1023, 864, 738. $[\alpha]_D^{25}$ = +22.0 (*c* 1.0, $CHCl_3$).

**Raney Nickel reduction towards lactams[9]**

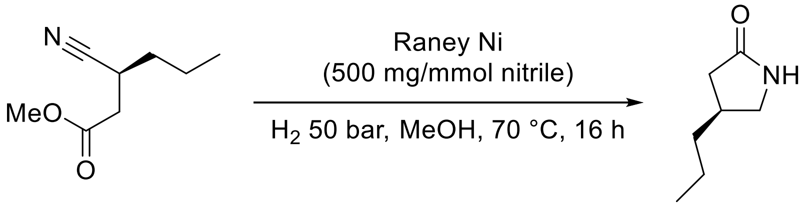


**6c** **7c**

The Raney Nickel reduction was carried out following a modified literature procedure: A Steel autoclave with glass inlet was charged with Raney® 2800 Ni (500 mg/mmol nitrile) and MeOH (10 mL/g Raney Ni). The nitrile (78 mg, 0.5 mmol) was added and the mixture was hydrogenated at 50 bar $H_2$ pressure and 70 °C for 16 h. The reaction mixture was cooled to rt and filtered over celite. Evaporation of the solvent and purification by column chromatography affords the pure product as colorless solid (58 mg, 91%).

**(S)-4-Propylpyrrolidin-2-one (7c):**

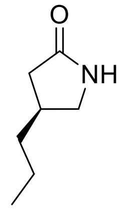


**$R_F$** = 0.23 (nHex/EtOAc 4:1). **$^1$H NMR** (400 MHz, $CDCl_3$): δ 6.23 (s, 1H), 3.47 (ddd, *J* = 9.1, 7.8, 1.1 Hz, 1H), 3.00 (dd, *J* = 9.5, 6.7 Hz, 1H), 2.55 – 2.35 (m, 2H), 2.07 – 1.91 (m, 1H), 1.50 – 1.39 (m, 2H), 1.38 – 1.24 (m, 2H), 0.91 (t, *J* = 7.2 Hz, 3H). **$^{13}$C NMR** (101 MHz, $CDCl_3$): δ 178.5, 48.1, 36.8, 36.7, 34.8, 20.6, 14.0. **HRMS** *m/z* (+ESI): calc. for $C_7H_{13}NO$ [M+Na]: 150.0889; found: 150.0884. **IR** (film) ν [$cm^{-1}$]: 3240, 3107, 2957, 2929, 2871, 2791, 1698, 1490, 1465, 1454, 1425, 1381, 1286, 1268, 1246, 1066, 765, 743, 696, 505. $[\alpha]_D^{25}$ = -8.0 (*c* 1.0, $CHCl_3$).

**General procedure for Raney Nickel reduction towards amino acids[10] (GP6)**

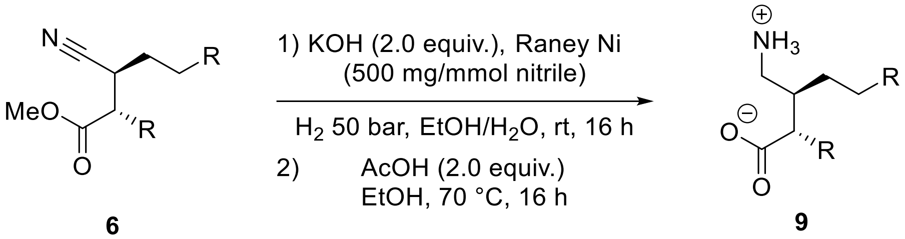


**6** **9**

The Raney Nickel reduction was carried out following a modified literature procedure: The nitrile was dissolved ethanol (1 mL/mmol nitrile) and water (1.5 mL/mmol nitrile) and potassium hydroxide (2 equiv.) was added. It was stirred for 1 h after which the mixture was added to a small vial charged with Raney® 2800 Ni (500 mg/mmol nitrile). The small vial was transferred into a steel autoclave with glass inlet and hydrogenated at 50 bar $H_2$ and rt for 16 h. The reaction mixture was filtered over celite and rinsed with ethanol and water. After evaporation of the solvent a minimal amount of ethanol and water were added together with acetic acid (2 equiv.) and the mixture was heated to 70 °C for 30 minutes. It was then slowly cooled to room temperature and further cooled to 0°C after which the precipitated product was collected by filtration and washing was cold propan-2-ol. The pure products were recrystallized from propan-2-ol for x-ray crystal structure analysis.

**(S)-3-(Ammoniomethyl)-5-methylhexanoate (pregabalin):**

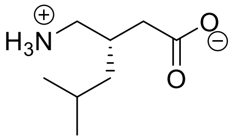

Prepared according to GP6 using nitrile **6g** (169 mg, 1.0 mmol), 126 mg, colorless solid, 79 %. **$^1$H NMR** (400 MHz, $D_2O$): δ 3.00 (qd, *J* = 13.0, 6.0 Hz, 1H), 2.39 – 2.13 (m, 1H), 1.68 (br-dquin, *J* = 13.4, 6.6 Hz, 1H), 1.24 (t, *J* = 6.8 Hz, 1H), 0.91 (d, *J* = 6.6 Hz, 1H), 0.90 (d, *J* = 6.6 Hz, 1H). **$^{13}$C NMR** (101 MHz, $D_2O$): δ 181.2, 43.7, 40.8, 40.6, 31.7, 24.4, 22.0, 21.5. **HRMS** *m/z* (+ESI): calc. for $C_8H_{17}NO_2$ [M+H]: 160.1332; found: 160.1348. **IR** (film) ν [cm$^{-1}$]: 3452, 2978, 2955, 2930, 2923, 2601, 2561, 2209, 1645, 1559, 1543, 1469, 1429, 1416, 1389, 1368, 1335, 1280, 1163, 860, 822, 702. **Mp.** = 171 °C. **$[\alpha]_D^{25}$** = +10.0 (*c* 1.0, $H_2O$).

**Synthesis of the sulfonamido acid[11]**

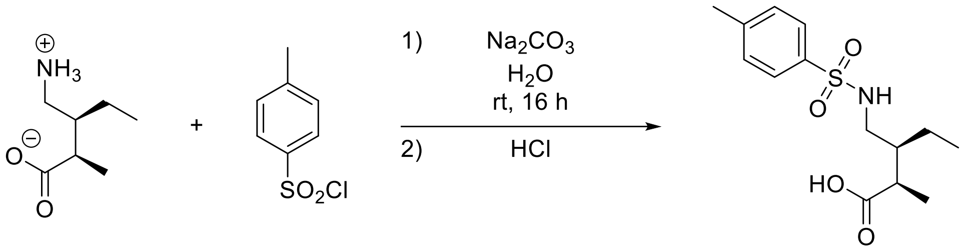


***anti*-9ab** ***anti*-10ab**

The conversion of amino acids towards sulfonamides was carried out following a modified literature procedure: The crude amino acid (after GP6) (145 mg, 1 mmol, 1.0 equiv.) and *p*-toluene sulfonyl chloride (229 mg, 1.2 mmol, 1.2 equiv.) was suspended in 4 mL water. The pH of the suspension was adjusted and was maintained at 8.0 by adding 1 mol/L $Na_2CO_3$ aqueous solution at room temperature. It took 16 hours for the reaction to complete. 6 M HCl solution was added slowly to adjust the pH to 2.0. The precipitate was collected by filtration,

washed with water and dried to afford the title compound as a white solid. The product was further purified by column chromatography using $CH_2Cl_2$ and EtOAc as eluent. The pure sulfonamide was recrystallized from $CH_2Cl_2$ and hexane for x-ray crystal structure analysis.

**(2R,3S)-2-methyl-3-(((4-methylphenyl)sulfonamido)methyl)pentanoic acid (*anti*-10ab):**

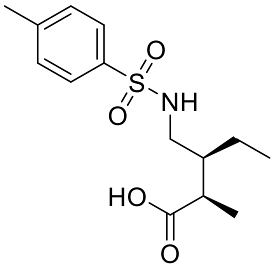


**$R_F$** = 0.31 ($CH_2Cl_2$/EtOAc 3:1). **$^1$H NMR** (400 MHz, $CDCl_3$): δ 7.76 – 7.67 (m, 2H), 7.31 – 7.23 (m, 2H), 5.49 (br-dd, *J* = 8.4, 4.9 Hz, 1H), 2.89 (br-dt, *J* = 12.8, 9.2 Hz, 1H), 2.79 (br-dt, *J* = 12.8, 4.9 Hz, 1H), 2.62 (qd, *J* = 7.1, 3.6 Hz, 1H), 2.41 (s, 3H), 2.11 (br-dtdd, *J* = 11.6, 7.3, 4.5, 3.6 Hz, 1H), 1.35 – 1.20 (m, 2H), 1.04 (d, *J* = 7.1 Hz, 3H), 0.88 (t, *J* = 7.4 Hz, 3H). **$^{13}$C NMR** (101 MHz, $CDCl_3$): δ 181.8, 143.4, 136.6, 129.7, 127.2, 43.7, 41.9, 39.2, 23.2, 21.5, 11.8, 9.8. **HRMS** *m/z* (+ESI): calc. for $C_{14}H_{21}NO_4S$ [M+H]: 300.1264; found: 300.1272. **IR** (film) ν [$cm^{-1}$]: 3262, 3224, 2967, 2928, 2880, 1708, 1597, 1458, 1425, 1327, 1292, 1266, 1217, 1163, 1091, 1044, 962, 884, 818, 703, 680, 587, 553. **Mp.** = 140 °C. $[\alpha]_D^{25}$ = -20.0 (*c* 1.0, $H_2O$).

# Synthesis of catalysts

### 3-Bromo-6,6,9,9,11,11-hexamethyl-7,8,9,11-tetrahydro-6H-benzo[b]fluorene:

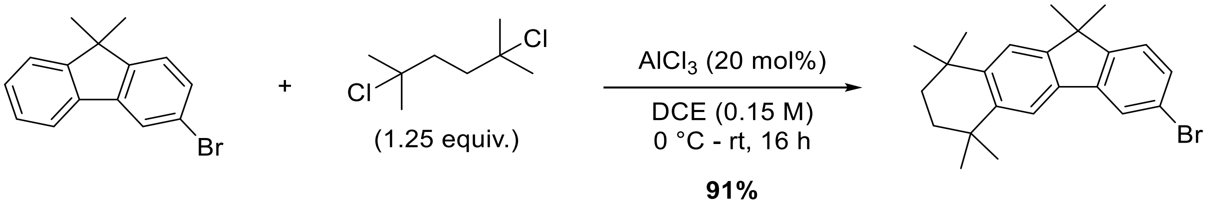


To a 50-mL Schlenk flask, 3-bromo-9,9-dimethyl-9H-fluorene (1.09 g, 4 mmol) was dissolved in dichloroethane (20 mL), the solution cooled to ~0 °C in an ice/salt bath and a catalytic amount of anhydrous $AlCl_3$ (100 mg, 0.2 equiv.) was added under an argon atmosphere. To the resulting mixture was then added a solution of 2,5-dichloro-2,5-dimethylhexane (0.92 g, 5.0 mmol, 1.25 equiv.) in dichloroethane (10 mL) dropwise during the course of 15 min and the ice bath was removed. The highly colored mixture was stirred for 16 h at room temperature under an argon atmosphere. The mixture was quenched by pouring it onto an ice (100 g)–HCl bath (10 mL), stirred for additional 15 min, and then the organic layer was separated and the aqueous layer was extracted with dichloromethane (2 x 25 mL). The combined organic layers were dried over anhydrous $MgSO_4$, filtered, and the solvent was removed. After purification by column chromatography (hexanes) the desired product was obtained as colorless solid (1.40 g, 91%).

### 3-Bromo-6,6,9,9,11,11-hexamethyl-7,8,9,11-tetrahydro-6H-benzo[b]fluorene:

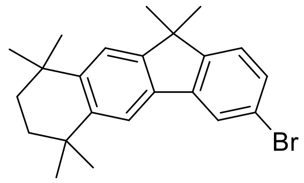


**$R_F$** = 0.72 (nHex).**$^1$H NMR** (400 MHz, $CDCl_3$): δ 7.82 (d, *J* = 1.8 Hz, 1H), 7.61 (s, 1H), 7.37 (dd, *J* = 8.0, 1.9 Hz, 1H), 7.34 (s, 1H), 7.27 – 7.22 (m, 1H), 1.73 (s, 4H), 1.45 (s, 6H), 1.36 (s, 6H), 1.34 (s, 6H). **$^{13}$C NMR** (101 MHz, $CDCl_3$): δ 152.7, 150.9, 145.2, 143.9, 141.7, 135.4, 129.3, 124.0, 122.8, 120.7, 120.4, 117.9, 46.4, 35.2, 35.2, 34.8, 34.5, 32.2, 32.2, 27.2. **HRMS** *m/z* (EI): calc. for $C_{23}H_{27}Br$ [M+H]: 382.1296; found: 382.1311. **Mp.** = 137 °C.

### BINOL synthesis[12,13]

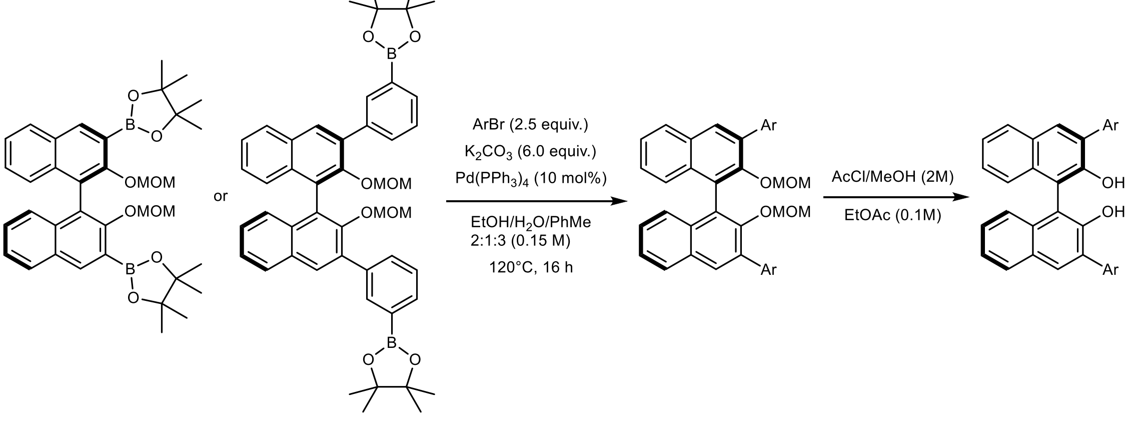


The starting BINOLs were synthesized according to a literature known procedure.[12,13] A flame-dried Schlenk flask equipped with a magnetic stir bar was charged under argon with the corresponding (*R*)-BINOL (1.0 mmol, 1.0 equiv.), the arylbromide (2.5 mmol, 2.5 equiv.) and potassium carbonate (830 mg, 6.0 mmol, 6 equiv.) and suspended in degassed EtOH:H2Odest.:PhMe (6 mL, 2:1:3 v/v%). Subsequently, tetrakis(triphenylphosphine)

palladium(0) (115 mg, 0.1 mmol, 10 mol%) was added under argon. The sealed Schlenk was heated under stirring at 120 °C for 16 hours. The mixture was cooled to room temperature and the mixture was mixed with $H_2O$ dest. (5 mL) extracted with $CH_2Cl_2$ (10 mL) for 3 times. The combined organic layers, dried over $MgSO_4$, were concentrated under reduced pressure. The crude mixture was purified by flash column chromatography (EtOAc:hexanes) to afford the product as a colorless solid.

A 2.0 M solution of HCl in EtOAc was prepared by the addition of AcCl to a solution of MeOH in EtOAc at 0°C. The cold solution (10 mL) was added to the MOM-protected BINOL and stirred upon warming to room temperature for 2 h. (Note: if the starting BINOL remained unsoluble $CH_2Cl_2$ was added). After completion of the reaction the solvent was removed under reduced pressure and the residue was dissolved in $CH_2Cl_2$ and evaporated again to remove residual HCl. The obtained colorless solid was used without further purification

**3,3'-Bis(9,9-dimethyl-9H-fluoren-3-yl)-[1,1'-binaphthalene]-2,2'-diol:**

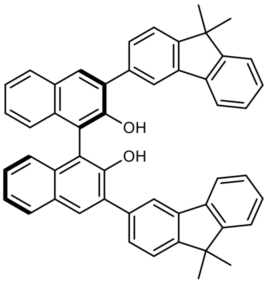


**Yield:** 573 mg, colorless solid, 85 %. **$^1$H NMR** (400 MHz, $CDCl_3$): δ 8.17 – 8.10 (m, 4H), 7.99 (d, J = 7.9 Hz, 2H), 7.81 (dt, J = 5.1, 3.0 Hz, 2H), 7.72 (dd, J = 7.8, 1.6 Hz, 2H), 7.59 (d, J = 7.8 Hz, 2H), 7.53 – 7.48 (m, 2H), 7.44 (ddd, J = 8.1, 6.8, 1.3 Hz, 2H), 7.41 – 7.35 (m, 6H), 7.32 (d, J = 8.3 Hz, 2H), 1.58 (s, 12H). **$^{13}$C NMR** (75 MHz, $CDCl_3$): δ 153.9, 152.8, 151.6, 139.4, 139.1, 137.9, 135.9, 133.7, 130.9, 130.7, 129.0, 127.9, 127.3, 127.0, 126.5, 126.5, 126.3, 125.2, 122.6, 122.4, 121.2, 120.2, 46.8, 27.3. **HRMS** *m/z* (+ESI): calc. for $C_{50}H_{38}O_2Na$ [M+Na]: 693.2764; found: 693.2784. **IR** (film) ν [cm$^{-1}$]: 3519, 3054, 2959, 1620, 1498, 1490, 1455, 1439, 1412, 1383, 1360, 1299, 1258, 1234, 1171, 1147, 1145, 1125, 1027, 889, 830, 783, 759, 747, 668, 633. **M.p.** = 253 °C. $[\alpha]_D^{25}$ = -52.0 (*c* 1.0, $CHCl_3$).

**3,3'-Bis(6,6,9,9,11,11-hexamethyl-7,8,9,11-tetrahydro-6H-benzo[b]fluoren-3-yl)-[1,1'-binaphthalene]-2,2'-diol:**

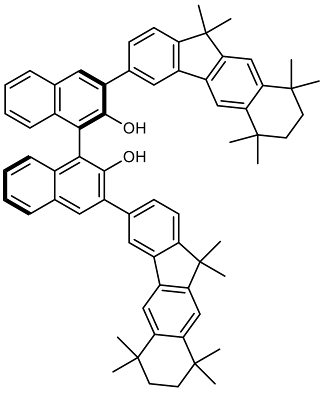


**Yield:** 732 mg, colorless solid, 82 %. **$^1$H NMR** (400 MHz, $CDCl_3$): δ 8.12 (s, 1H), 8.07 (d, *J* = 1.6 Hz, 1H), 8.00 – 7.93 (m, 1H), 7.71 (s, 1H), 7.64 (dd, *J* = 7.8, 1.6 Hz, 1H), 7.53 (d, *J* = 7.8 Hz, 1H), 7.46 – 7.32 (m, 3H), 7.30 (dd, *J* = 8.4, 1.4 Hz, 1H), 5.53 (s, 1H), 1.74 (s, 4H), 1.54 (s, 6H), 1.42 – 1.32 (m, 12H). **$^{13}$C NMR** (101 MHz, $CDCl_3$): δ 153.6, 151.0, 150.1, 144.5, 143.7, 140.0, 136.5, 136.0, 133.1, 131.2, 131.1, 129.5, 128.4, 128.1, 127.1, 124.5, 124.2, 122.7, 120.8, 120.3, 117.9, 112.8, 46.5, 35.3, 34.8, 34.6, 32.2, 32.2, 27.5. **HRMS** *m/z* (+ESI): calc. for $C_{66}H_{67}O_2$ [M+H]: 891.5136; found: 891.5122. **IR** (film) ν [cm$^{-1}$]: 3523, 3434, 2958, 2924, 2861, 1620, 1495, 1457, 1399, 1384, 1362, 1270, 1259, 1236, 1171, 1147, 1125, 885, 828, 748. **M.p.** = 268 °C. $[\alpha]_D^{25}$ = -46.0 (*c* 1.0, $CHCl_3$).

**3,3'-Bis(3-(6,6,9,9,11,11-hexamethyl-7,8,9,11-tetrahydro-6H-benzo[b]fluoren-3-yl)phenyl)-[1,1'-binaphthalene]-2,2'-diol:**

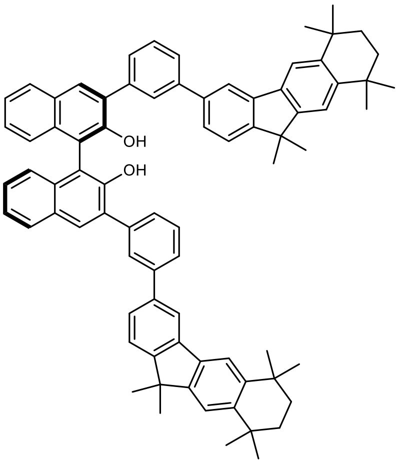

**Yield:** 791 mg, colorless solid, 69 %. **$^1$H NMR** (400 MHz, $CDCl_3$): δ 8.12 (s, 1H), 8.07 (d, *J* = 1.6 Hz, 1H), 8.00 – 7.93 (m, 1H), 7.71 (s, 1H), 7.64 (dd, *J* = 7.8, 1.6 Hz, 1H), 7.53 (d, *J* = 7.8 Hz, 1H), 7.46 – 7.32 (m, 3H), 7.30 (dd, *J* = 8.4, 1.4 Hz, 1H), 5.53 (s, 1H), 1.74 (s, 4H), 1.54 (s, 6H), 1.42 – 1.32 (m, 12H). **$^{13}$C NMR** (101 MHz, $CDCl_3$): δ 153.6, 151.0, 150.1, 144.5, 143.7, 140.0, 136.5, 136.0, 133.1, 131.2, 131.1, 129.5, 128.4, 128.1, 127.1, 124.5, 124.2, 122.7, 120.8, 120.3, 117.9, 112.8, 46.5, 35.3, 34.8, 34.6, 32.2, 32.2, 27.5. **HRMS** *m/z* (+ESI): calc. for $C_{78}H_{74}O_2Na$ [M+Na]: 1065.5581; found: 1065.5571. **IR** (film) ν [cm$^{-1}$]: 3520, 3434, 2958, 2925, 2860, 1621, 1603, 1472, 1457, 1397, 1384, 1361, 1272, 1261, 1172, 1147, 1128, 880, 797, 749, 728, 704. **M.p.** = 247 °C. $[\alpha]_D^{25}$ = -38.0 (*c* 1.0, $CHCl_3$).

**IDPi Synthesis[12,13]**

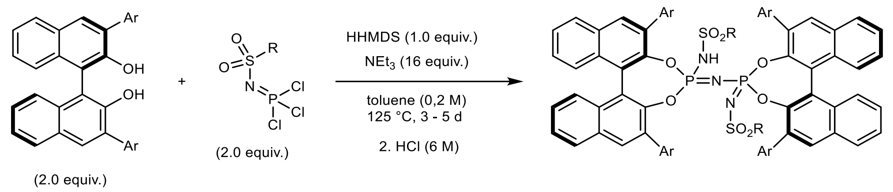


Following a known literature procedure[12,13], the BINOL (2.0 equiv., 0.5 mmol) was evacuated several times in a flame-dried Schlenk flask. After addition of toluene (2.5 mL) the phosphazene (2.0 equiv., 0.5 mmol) was added at 0 °C followed by $NEt_3$ (16 equiv., 4.0 mmol) and stirring for 30 – 60 min at room temperature. The mixture was cooled to 0 °C and HHMDS (1.0 equiv., 0.25 mmol) and stirred at room temperature for 30 minutes upon heating to 125 °C, sealing the flask and stirring for 3 – 5 days. The reaction was cooled to room temperature and diluted with $CH_2Cl_2$. After evaporation of the solvent the product was purified by column chromatography (1. EtOAc/hexanes, 2. $CH_2Cl_2$/hexanes). The purified product was acidified in 6 M HCl/$CH_2Cl_2$ (1:1, v:v) by stirring over 2 h. After extraction with $CH_2Cl_2$ (3 times) the solvent was evaporated and residual water was removed by azeotropic distillation with $CHCl_3$ to afford the IDPi acid.

**N,N'-(Azanediylbis(2,6-bis(9,9-dimethyl-9H-fluoren-3-yl)-4l5-dinaphtho[2,1-d:1',2'-f][1,3,2]dioxaphosphepine-4-yl-4-ylidene))bis(1,1,1-trifluoromethanesulfonamide) (3f):**

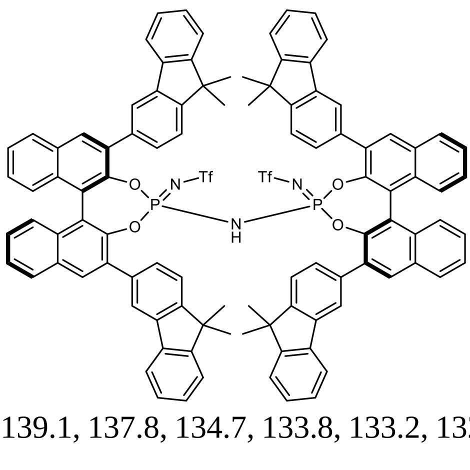

**Yield:** 230 mg, colorless solid, 54 %. **R$_F$** = 0.67 (nHex/EtOAc 3:1). **$^1$H NMR** (400 MHz, $CDCl_3$): δ 8.17 – 8.09 (m, 4H), 8.05 (d, *J* = 8.2 Hz, 2H), 7.91 (d, *J* = 1.6 Hz, 2H), 7.84 (ddd, *J* = 8.1, 6.6, 1.3 Hz, 2H), 7.76 (d, *J* = 8.4 Hz, 2H), 7.72 – 7.64 (m, 6H), 7.58 (ddd, *J* = 8.1, 6.5, 1.4 Hz, 2H), 7.49 – 7.32 (m, 10H), 7.27 (m, 6H), 7.15 (td, *J* = 7.5, 1.2 Hz, 2H), 7.03 (td, *J* = 7.5, 1.2 Hz, 2H), 6.64 (d, *J* = 7.9 Hz, 2H), 6.59 (dd, *J* = 8.0, 1.7 Hz, 2H), 6.37 (d, *J* = 8.0 Hz, 2H), 5.93 (dd, *J* = 7.9, 1.7 Hz, 2H), 1.24 (s, 3H), 1.22 (d, *J* = 1.9 Hz, 9H). **$^{13}$C NMR** (101 MHz, $CDCl_3$): δ 153.7, 153.7, 148.1, 143.7, 141.9, 139.1, 137.8, 134.7, 133.8, 133.2, 132.0, 131.8, 129.3, 128.9, 128.7, 128.1, 127.5, 127.1, 126.9,

126.7, 125.3, 123.6, 122.7, 122.3, 122.0, 121.3, 120.7, 120.0, 99.9, 49.0, 46.5, 46.5, 35.7, 29.7, 28.9, 28.2, 27.0, 26.5, 25.2, 20.5. **$^{19}$F NMR** (377 MHz, $CDCl_3$): δ -78.63. **$^{31}$P NMR** (162 MHz, $CDCl_3$): δ -15.15. **HRMS** *m/z* (-ESI): calc. for $C_{102}H_{75}F_6N_4O_8P_2S_2$ [M+$NH_2^-$]: 1723.4400; found: 1723.4418. **M.p.** = 355 °C (decomposition). $[\alpha]_D^{25}$ = -268.0 (*c* 1.0, $CHCl_3$).

**N,N'-(Azanediylbis(2,6-bis(6,6,9,9,11,11-hexamethyl-7,8,9,11-tetrahydro-6H-benzo[b]fluoren-3-yl)-4l5-dinaphtho[2,1-d:1',2'-f][1,3,2]dioxaphosphepine-4-yl-4-ylidene))bis(1,1,1-trifluoromethanesulfonamide) (3g):**

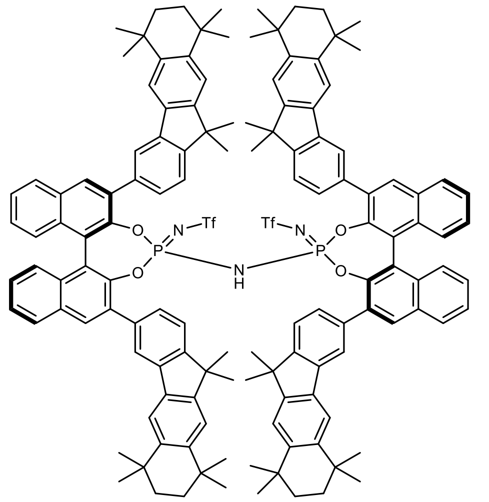


**Yield:** 292 mg, colorless solid, 54 %. **R$_F$** = 0.52 (nHex/EtOAc 9:1). **$^1$H NMR** (400 MHz, $CDCl_3$): δ 8.20 (s, 2H), 8.08 (dd, *J* = 8.3, 3.6 Hz, 4H), 7.96 (d, *J* = 1.6 Hz, 2H), 7.85 – 7.77 (m, 4H), 7.74 – 7.64 (m, 6H), 7.62 – 7.53 (m, 4H), 7.50 – 7.42 (m, 4H), 7.38 (ddd, *J* = 8.4, 6.7, 1.3 Hz, 2H), 7.17 (d, *J* = 7.6 Hz, 4H), 6.53 (d, *J* = 7.8 Hz, 2H), 6.44 (dd, *J* = 8.0, 1.7 Hz, 2H), 6.26 (d, *J* = 8.0 Hz, 2H), 5.71 (dd, *J* = 7.9, 1.7 Hz, 2H), 1.70 (m, 16H), 1.43 (m, 6H), 1.35 (m, 6H), 1.31 – 1.22 (m, 30H), 1.17 (m, 24H), 1.07 (s, 6H). **$^{13}$C NMR** (101 MHz, $CDCl_3$): δ 153.7, 153.5, 150.8, 150.8, 144.4, 143.8, 143.7, 143.7, 143.4, 143.3, 143.2, 139.7, 139.6, 136.6, 136.5, 134.6, 133.9, 133.3, 132.3, 132.1, 132.0, 131.8, 131.7, 131.4, 128.8, 128.8, 127.7, 127.4, 127.1, 127.0, 126.7, 126.7, 126.3, 123.8, 122.1, 122.0, 121.9, 120.7, 120.7, 120.4, 120.2, 120.0, 117.8, 117.6, 46.2, 46.1, 35.4, 34.7, 34.7, 34.5, 34.5, 32.4, 32.3, 32.3, 32.2, 32.1, 32.1, 32.0, 29.7, 29.4, 27.4, 26.9, 25.2. **$^{19}$F NMR** (377 MHz, $CDCl_3$): δ -78.10. **$^{31}$P NMR** (162 MHz, $CDCl_3$): δ -15.52. **HRMS** *m/z* (-ESI): calc. for $C_{134}H_{129}F_6N_3O_8P_2S_2$ [M-H]: 2146.8517; found: 2146.8582. **M.p.** = 376 °C (decomposition). $[\alpha]_D^{25}$ = -240.0 (*c* 1.0, $CHCl_3$).

**N,N'-(Azanediylbis(2,6-bis(3-(6,6,9,9,11,11-hexamethyl-7,8,9,11-tetrahydro-6H-benzo[b]fluoren-3-yl)phenyl)-4l5-dinaphtho[2,1-d:1',2'-f][1,3,2]dioxaphosphepine-4-yl-4-ylidene))bis(1,1,1-trifluoromethanesulfonamide) (3h):**

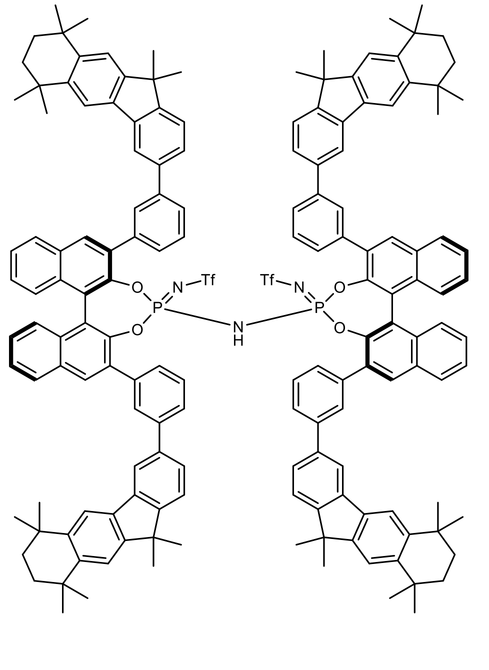


**Yield:** 551 mg, colorless solid, 61 %. **R$_F$** = 0.64 (nHex/EtOAc 9:1). **$^1$H NMR** (400 MHz, $CDCl_3$): δ 8.14 (s, 2H), 7.99 – 7.91 (m, 24), 7.85 (t, *J* = 1.8 Hz, 2H), 7.80 (ddd, *J* = 8.1, 6.8, 1.1 Hz, 2H), 7.69 (s, 2H), 7.65 – 7.53 (m, 12H), 7.49 (ddd, *J* = 8.3, 6.8, 1.3 Hz, 2H), 7.43 (d, *J* = 7.8 Hz, 2H), 7.37 – 7.28 (m, 8H), 7.26 (m, 2H), 7.19 – 7.07 (m, 12H), 6.98 (d, *J* = 8.0 Hz, 2H), 6.92 – 6.82 (m, 4H), 1.78 (s, 8H), 1.72 (s, 8H), 1.49 (m, 12H), 1.41 (m, 24H), 1.34 (m, 24H), 1.28 (s, 6H), 1.24 (s, 6H). **$^{13}$C NMR** (101 MHz, $CDCl_3$): δ 152.9, 152.8, 151.1, 151.0, 144.4, 144.0, 143.7, 143.5, 143.5, 143.0, 142.6, 140.4, 139.8, 139.7, 139.2, 136.8, 136.5, 135.7, 135.7, 133.3, 132.7, 131.9, 131.5, 130.8, 129.5, 128.7, 128.5, 128.2, 128.1, 127.8, 127.6, 127.4, 127.3, 126.8, 126.5, 126.4, 126.2, 126.0, 123.4, 122.5, 121.8, 121.3, 120.3, 120.1, 119.3, 118.9, 117.9, 117.8, 46.3, 46.1, 35.3, 34.8, 34.7, 34.6, 34.5, 32.5, 32.4, 32.3, 32.2, 32.2, 32.1, 32.1,

27.6, 27.5, 27.0. **$^{19}$F NMR** (377 MHz, $CDCl_3$): δ -78.15. **$^{31}$P NMR** (162 MHz, $CDCl_3$): δ -17.09. **HRMS** *m/z* (-ESI): calc. for $C_{158}H_{144}F_6N_3O_8P_2S_2$ [M-H]: 2450.9769; found: 2450.9828. **M.p.** = 321 °C (decomposition). $[\alpha]_D^{25}$ = -38.0 (*c* 1.0, $CHCl_3$).

# Mechanistic investigations

## Mass analysis of 3i – 1a:

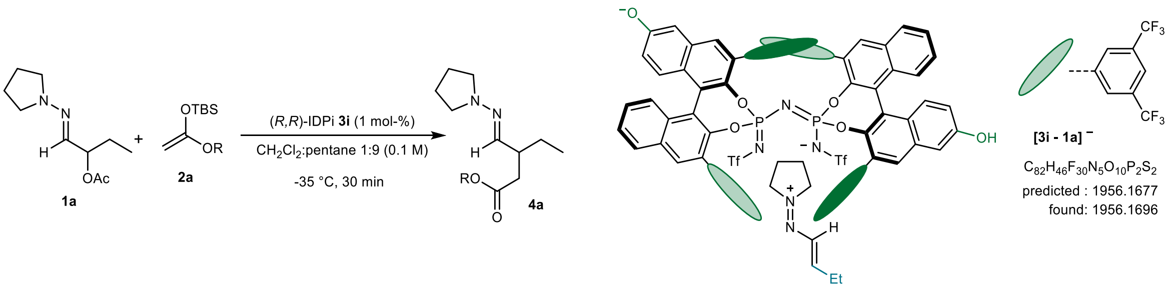


**Supplementary Figure 8:** Reaction conditions for the ESI (-) mass analysis.

An oven dried and argon filled Schlenk vial was charged with IDPi **3i** (1.8 mg, 1 mol%) and 1 mL of dry solvent was added ($CH_2Cl_2$:pentane 1:9). After addition of silylketene acetal **2a** (33 µL, 0.15 mmol, 1.5 equiv.) the mixture was stirred for 5 minutes at room temperature. It was subsequently cooled to -35 °C and stirred for further 5 minutes before hydrazone **1a** (20 mg, 0.1 mmol, 1.0 equiv.) was added. The reaction was allowed to run at -35 °C for 30 minutes and then cooled to -78 °C. The cold mixture was directly measured via (-) – ESI mass spectrometry. The expected ion pair **3i – 1a** was characterized as anion ($M\text{-}H^+ \equiv C_{82}H_{46}F_{30}N_5O_{10}P_2S_2$) with a mass of 1956.1696 (predicted: 1956.1677).

## $^{31}$P NMR of reaction intermediates:

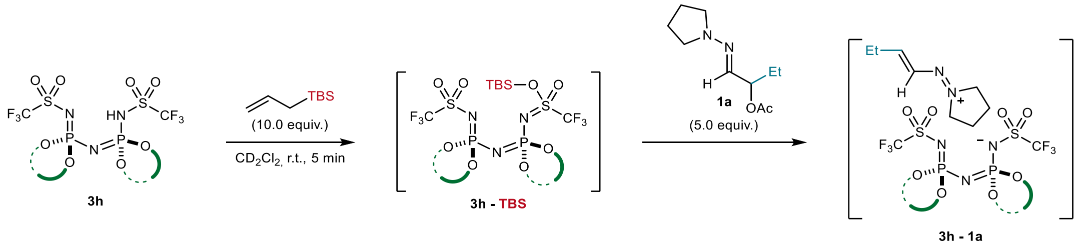


**Supplementary Figure 9:** Reaction conditions for the $^{31}$P-NMR analysis.

The $^{31}$P investigation was carried out using a modified method inspired by the group of List and co-workers.[4] In an Young NMR tube a solution of IDPi **3h** (18.5 mg, 7.54 µmol, 1.0 equiv.) in dichloromethane-$d_2$ (1.0 mL) was inserted into the NMR spectrometer and the first spectrum was recorded at rt (IDPi **3h**). In the next step *tert*-butyldimethylallylsilane (15.7 µL, 75.4 µmol, 10.0 equiv.) was added and the NMR tube was shaken for 5 minutes at rt before the next spectrum was recorded (IDPi **3h – TBS**). Subsequently, hydrazone **1a** (15.0 mg, 75.4 µmol, 10.0 equiv) was added and the mixture was shaken for 5 minutes before the last spectrum was recorded (IDPi **3h – 1a**).

## Monitoring of the reaction by $^{31}$P NMR spectroscopy:

The reaction was monitored by $^1$H and $^{31}$P NMR spectroscopy under slightly modified reaction conditions to ensure better comparability with the preliminary $^{31}$P investigation (Supplementary Fig. 9) and to maintain a constant temperature. An oven-dried NMR tube was charged with IDPi **3h** (2.5 mg, 1 mol%) and 1 mL of dichloromethane-$d_2$. After the addition of silyl ketene acetal **2a** (44 µL, 0.2 mmol, 2.0 equiv.), the mixture was shaken for 5 minutes at room temperature. The reaction mixture was then cooled to −78 °C, and hydrazone **1a** (20 mg, 0.1

mmol, 1.0 equiv.) was added. The cold NMR tube was subsequently inserted into a pre-cooled NMR spectrometer set to −5 °C, and $^{1}H$ and $^{31}P$ NMR spectra were recorded each hour.

The well separated signals of the carbonyl hydrogen atoms (**4a** at 6.42 ppm, **1a** at 6.28 ppm) were used to ensure the progress of the reaction.

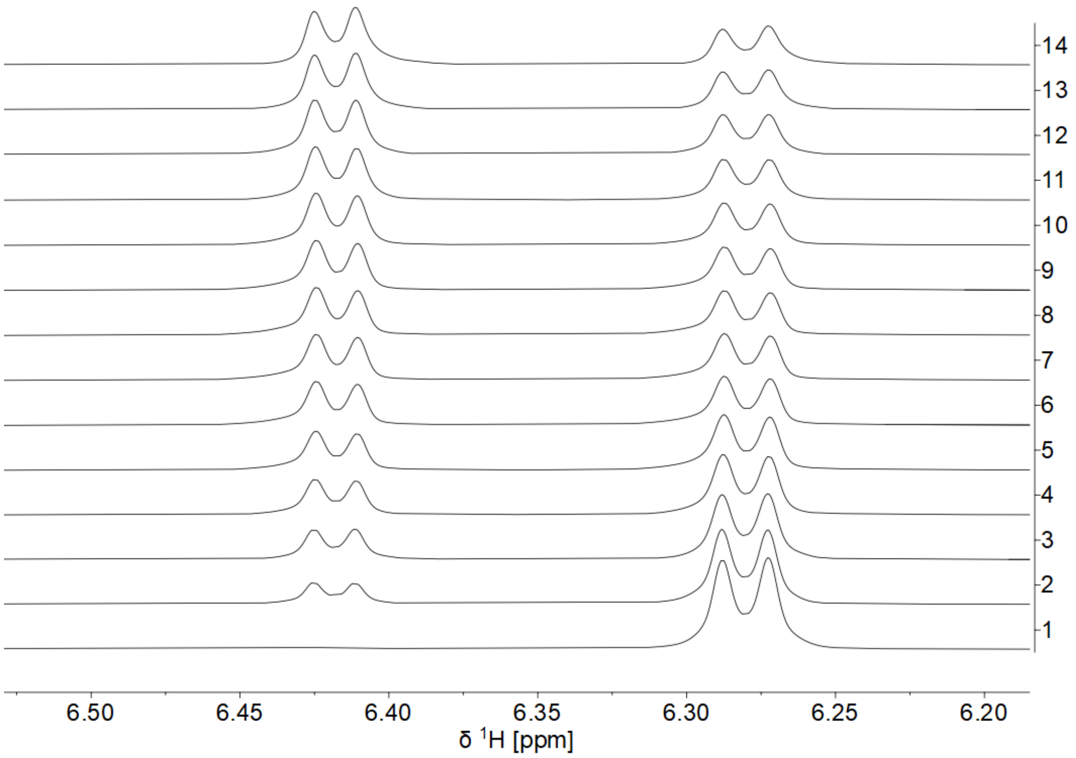


**Supplementary Figure 10:** $^{1}H$ monitoring of the reaction progress (1-14 h).

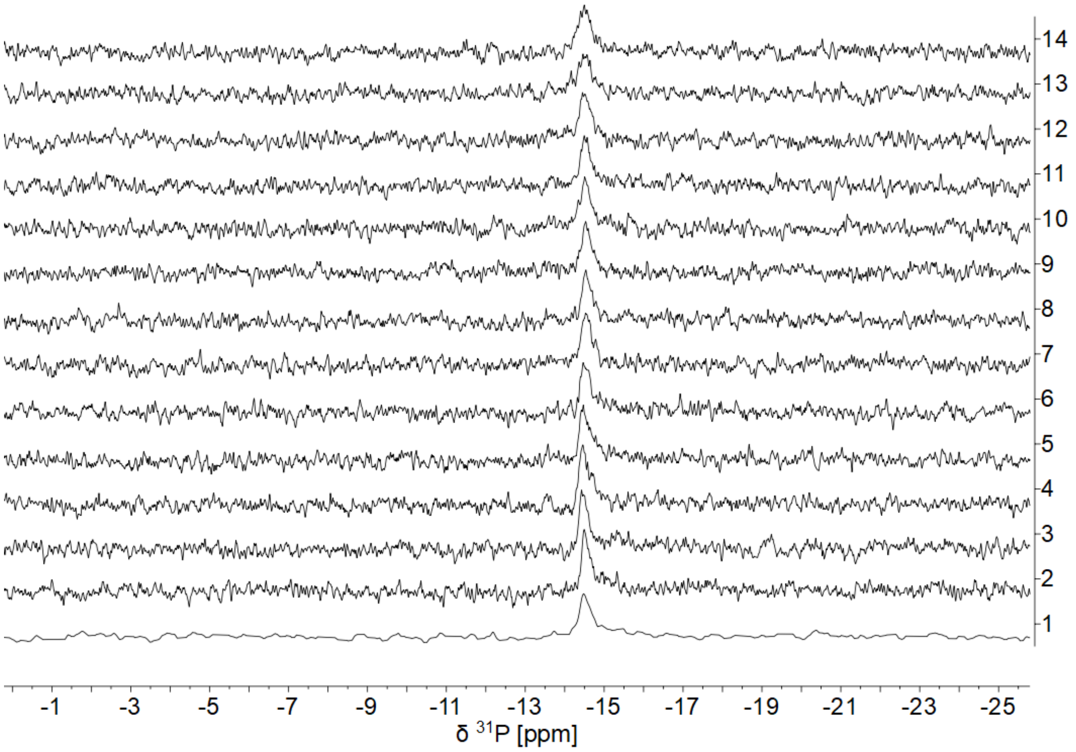


**Supplementary Figure 11:** $^{31}P$ monitoring of the reaction progress (1-14 h).

**Kinetic studies:**

Three stock solutions were prepared for the kinetic investigation:

Solution 1: IPDi **3h** in dichloromethane-d$_2$ (10 µmol/mL)

Solution 2: silylketene acetal **2a** in dichloromethane-d2 (1.0 mmol/mL)

Solution 3: hydrazone **1a** in dichloromethane-d2 (1.0 mmol/mL)

General procedure for the NMR experiments:

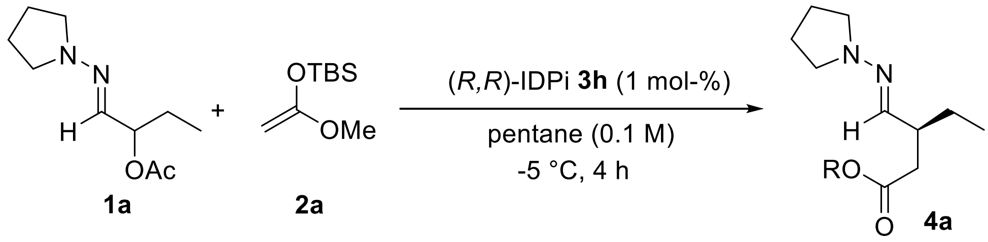


An oven dried Young NMR tube was charged with the IDPi **1h** stock solution (100 µL, 1.0 µmol, 1 mol%) and pentane-d12 (0.5 µL). After addition of the silylketene acetal **2a** solution (X µL) and $CD_2Cl_2$ (X µL) the NMR tube was shaken for 5 minutes at rt before the solution was cooled to -78 °C. Subsequently, the hydrazone **1a** solution (100 µL, 100 µmol, 1 equiv.) was added at low temperature and the NMR tube was shaken for 60 seconds and directly transferred into the precooled NMR spectrometer (-5 °C). Three experiments were conducted with variable amounts of nucleophile **2a** (1.0 equiv., 1.5 equiv. 3.0 equiv.).

1. Experiment: 100 µL of solution 2 (100 µmol, 1.0 equiv. **2a**) and 200 µL $CD_2Cl_2$.
2. Experiment: 150 µL of solution 2 (150 µmol, 1.5 equiv. **2a**) and 150 µL $CD_2Cl_2$.
3. Experiment: 300 µL of solution 2 (300 µmol, 3.0 equiv. **2a**), no additional $CD_2Cl_2$.

The first $^1$H NMR data were collected 10 minutes after the addition of hydrazone **1a** and additional data points were collected every 100 seconds. The well separated signals of the carbonyl hydrogen atoms (**4a** at 6.49 ppm, **1a** at 6.34 ppm) were used as datasets.

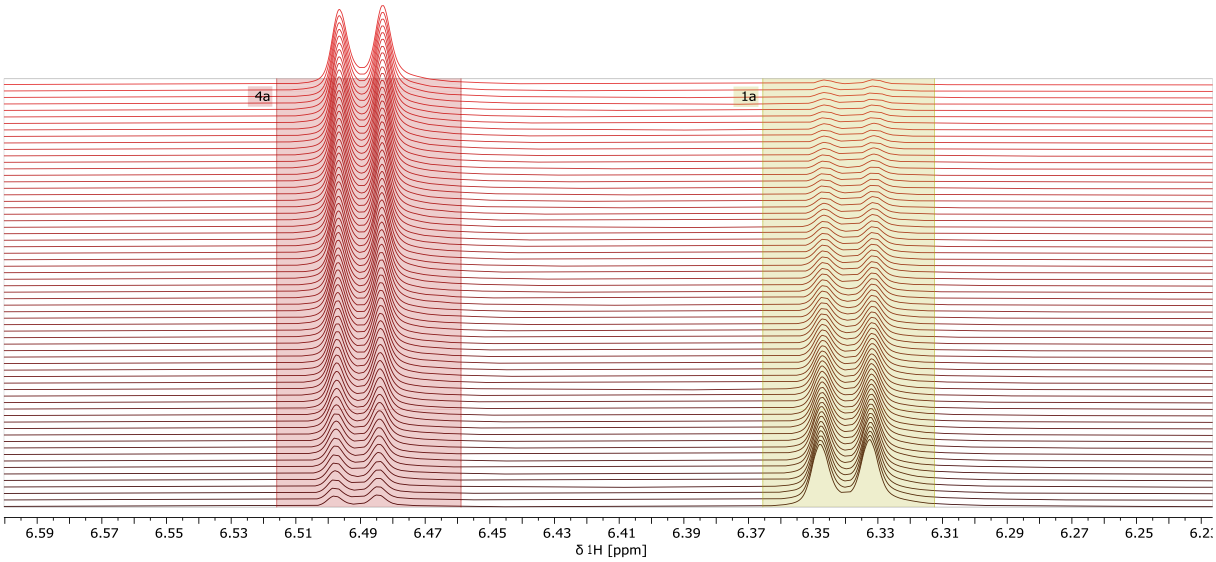


**Supplementary Figure 12:** $^1$H-NMR data of hydrazone CH-signals of **1a** (right) and **4a** (left).

The order with respect to silylketene acetal **2a** was determined by variable time normalization analysis reported by the Burés group.[14] When comparing conversion plots normalized to different nucleophile orders (Supplementary Figure 10), the curves were found to overlap when a first order with respect to the nucleophile **2a** was displayed.

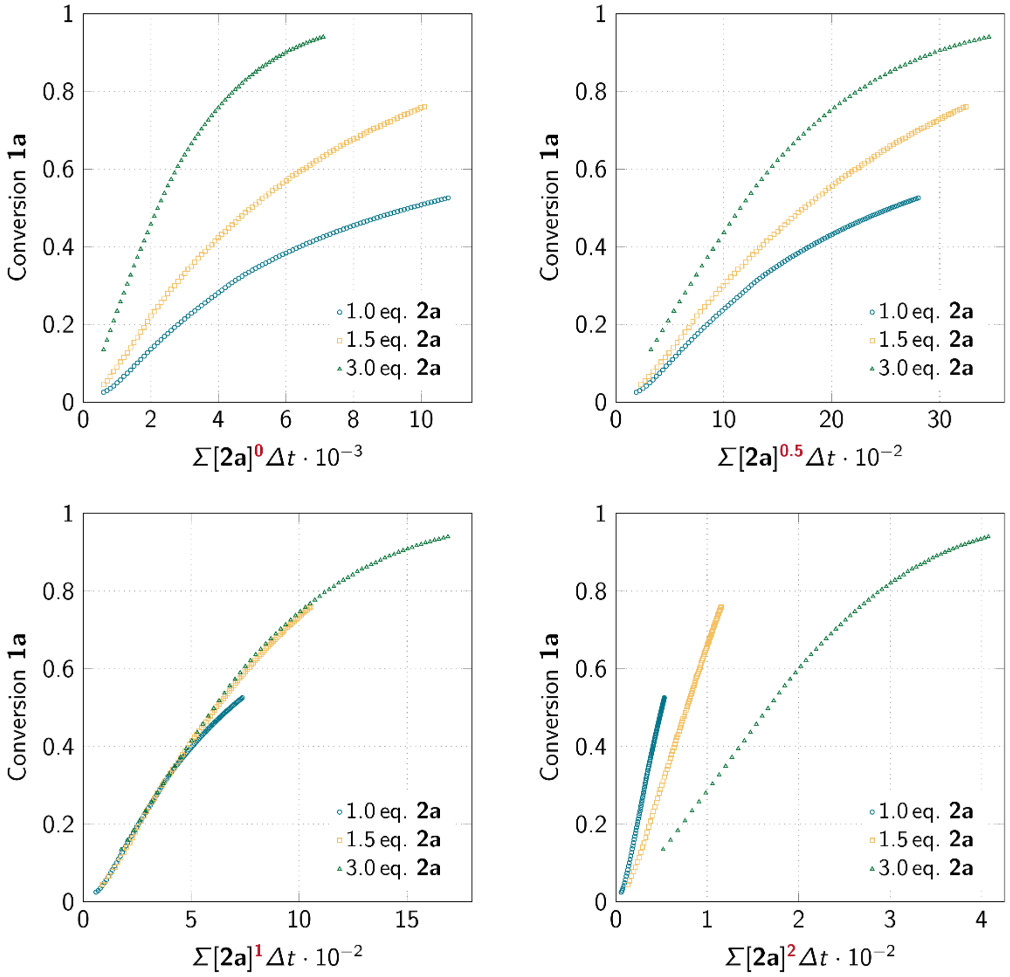


**Supplementary Figure 13:** Variable time normalization for nucleophile kinetics 0., 0.5., 1. and 2. order.[12]

Taking the characterized intermediates into account, this data strongly supports the hypothesis of a rate determining nucleophilic attack of silylketene acetal **2a** to chiral ion pair **3h** – **1a**.

# NMR Spectra

**$^1$H NMR** (300 MHz, $CDCl_3$):

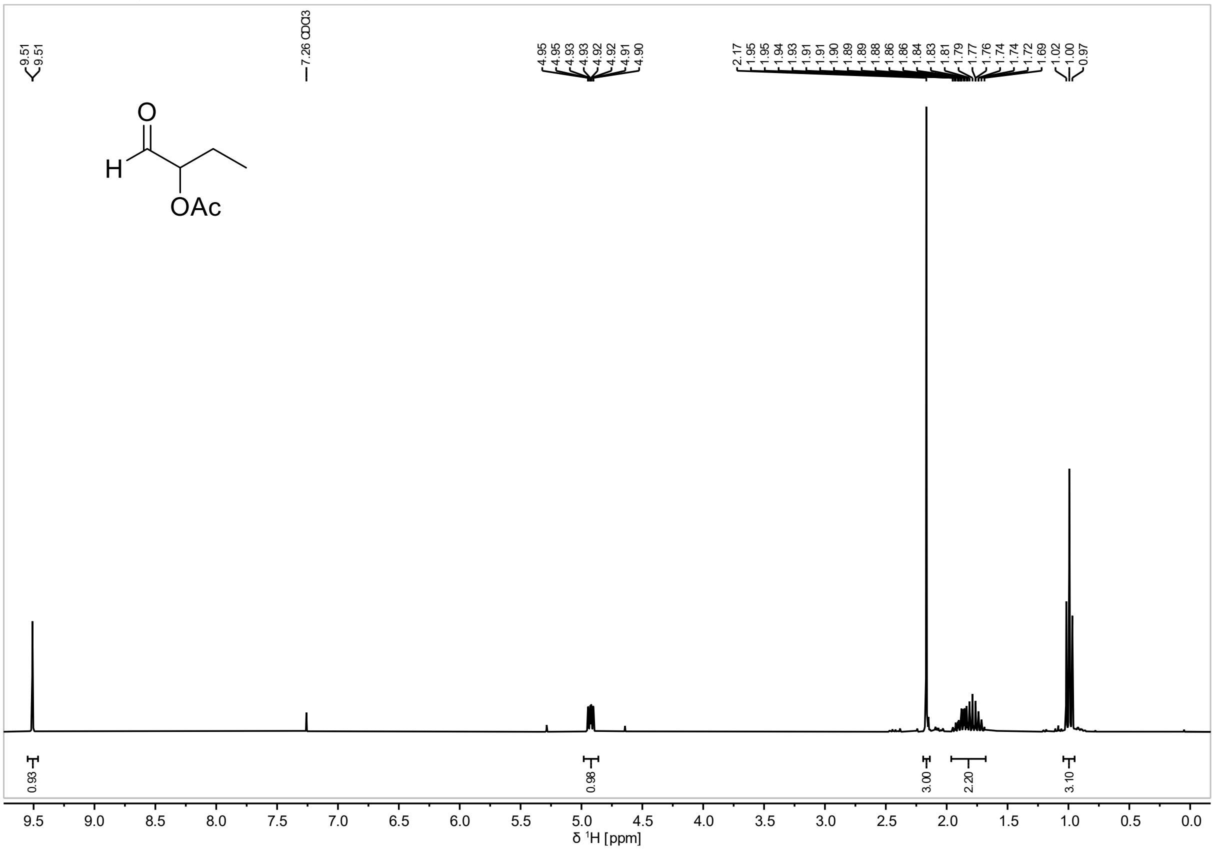

**$^{13}$C NMR** (101 MHz, $CDCl_3$):

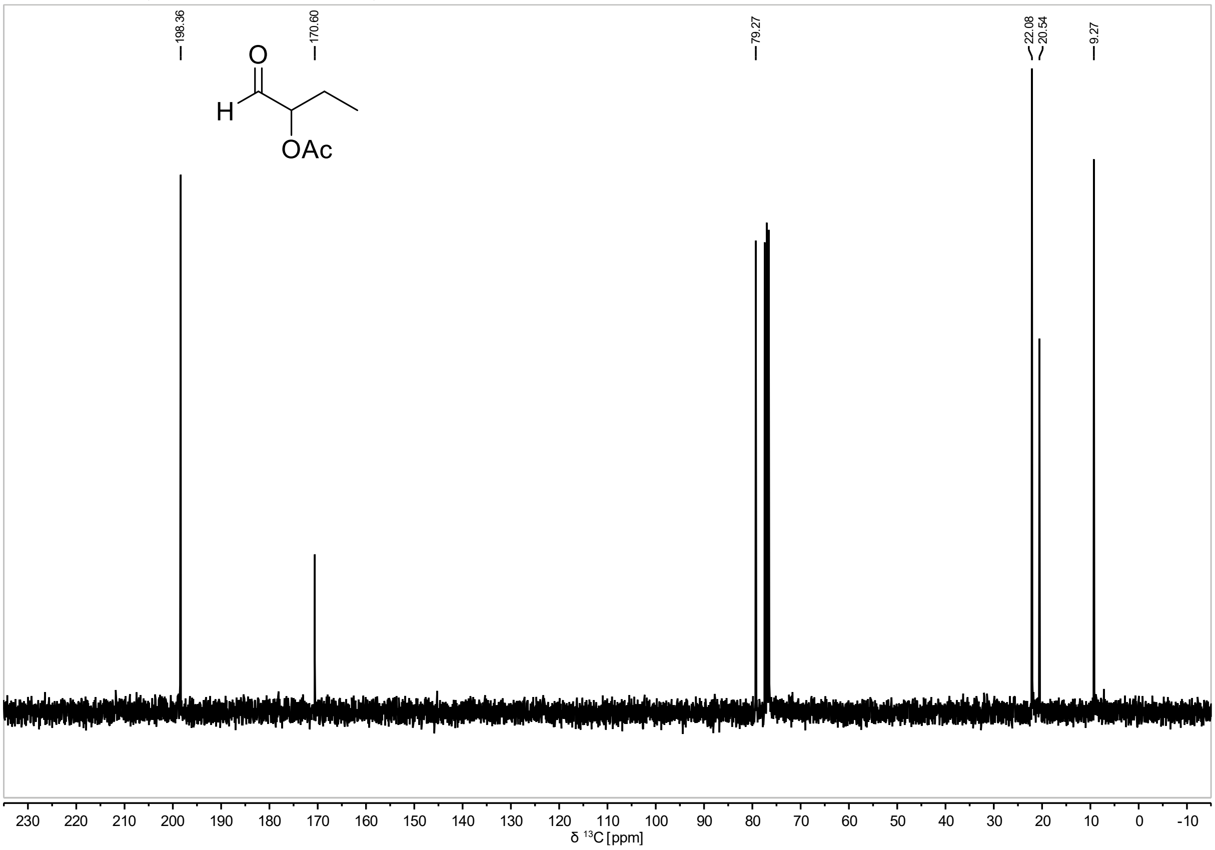

**$^{1}$H NMR** (400 MHz, $CDCl_3$):

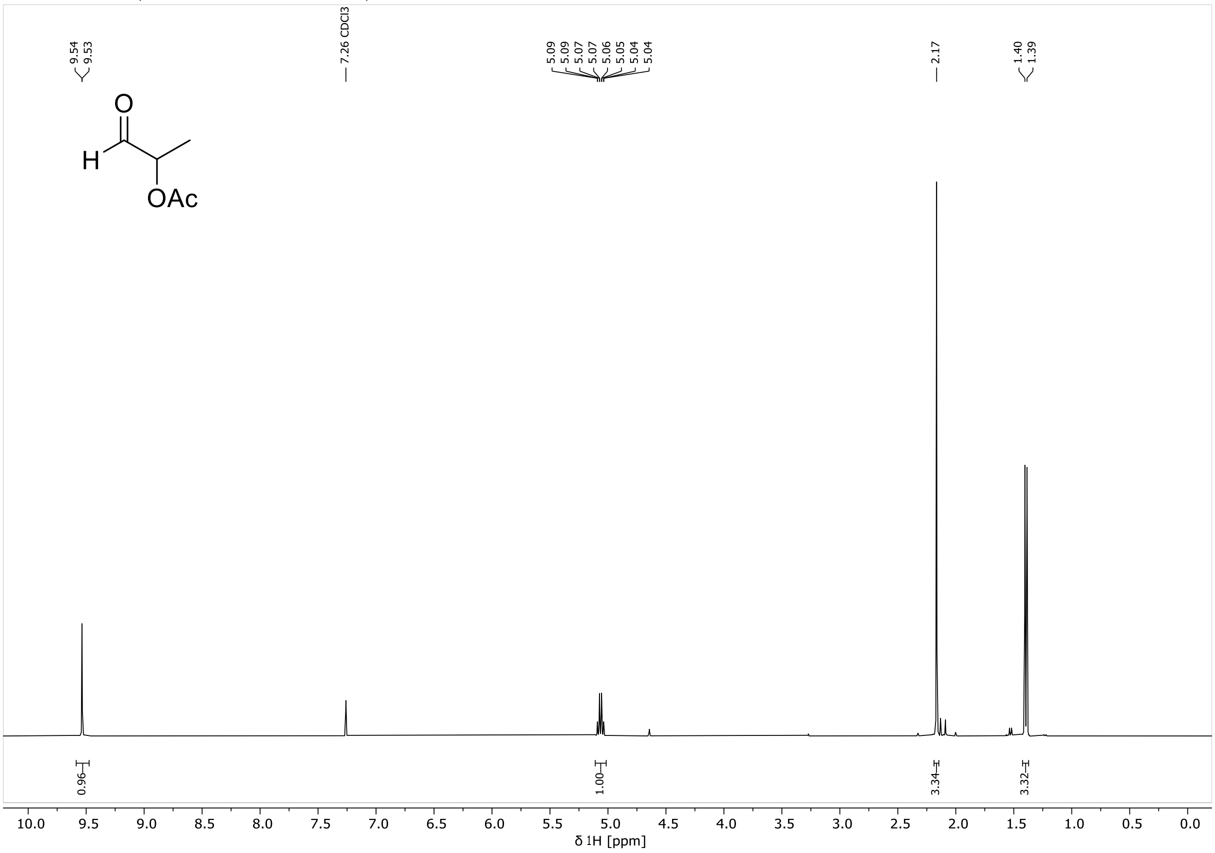


**$^{13}$C NMR** (101 MHz, $CDCl_3$):

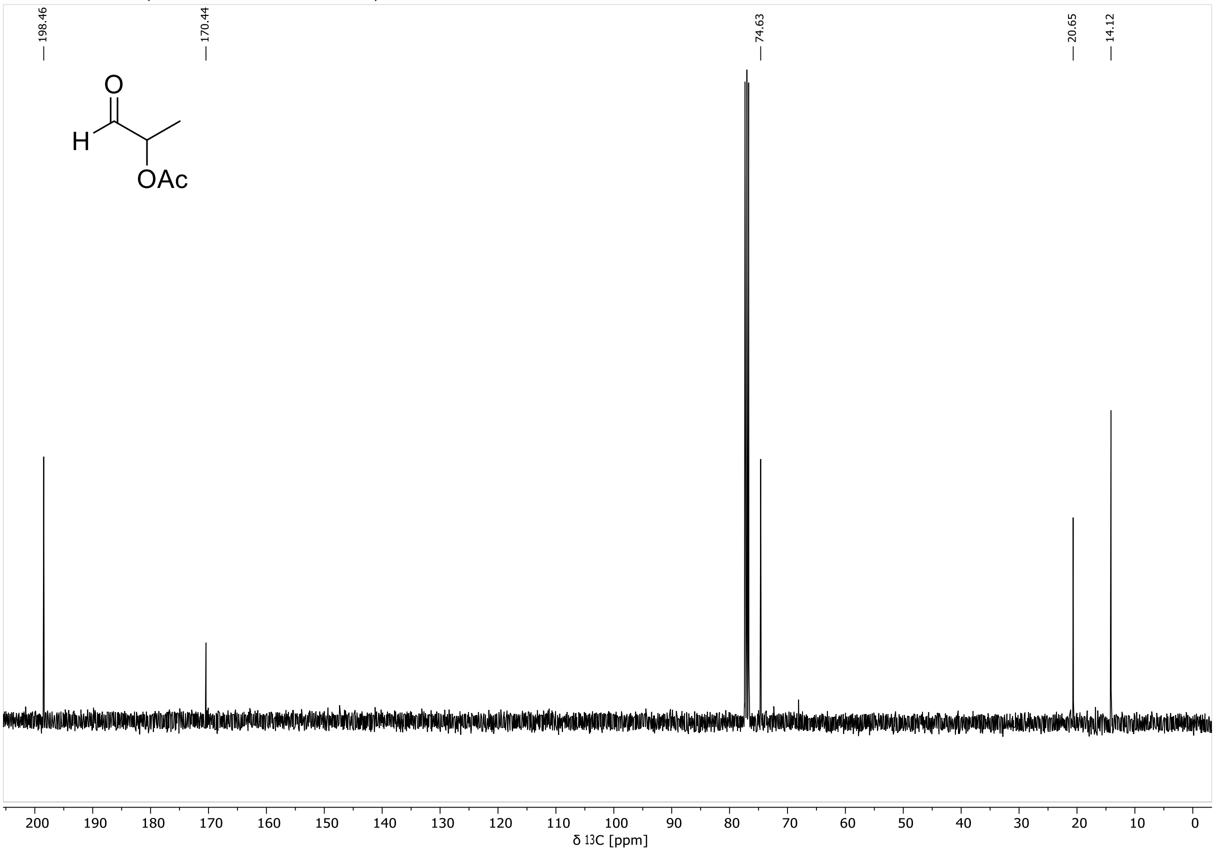

**$^{1}$H NMR** (400 MHz, $CDCl_3$):

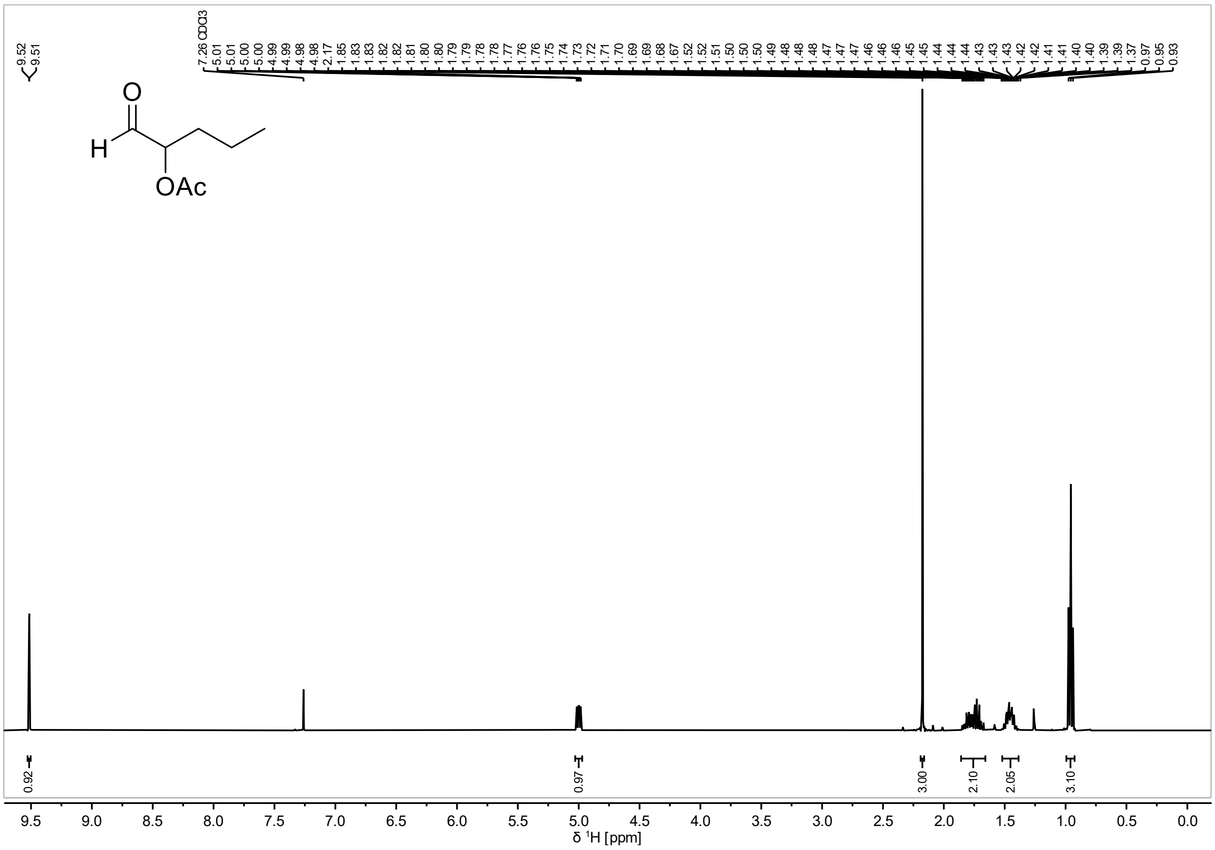

**$^{13}$C NMR** (101 MHz, $CDCl_3$):

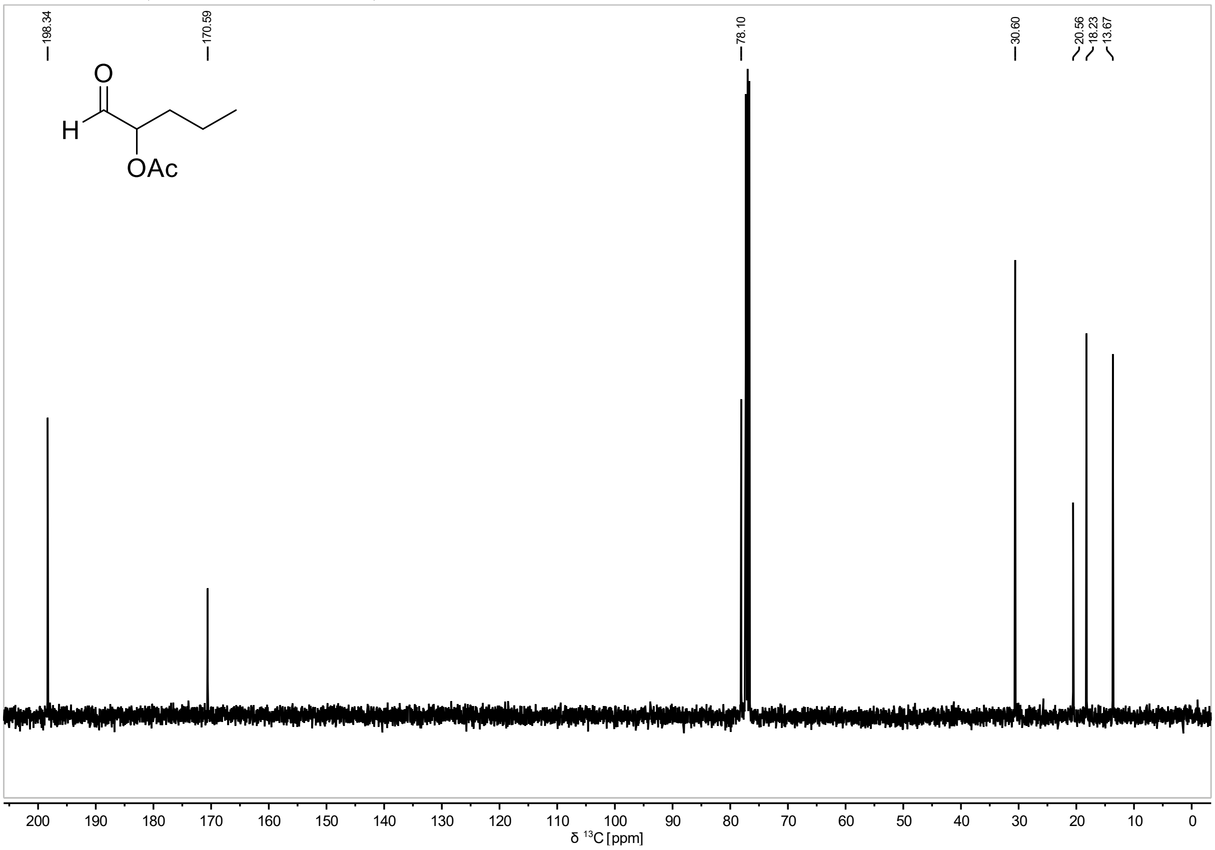

**$^{1}$H NMR** (400 MHz, $CDCl_3$):

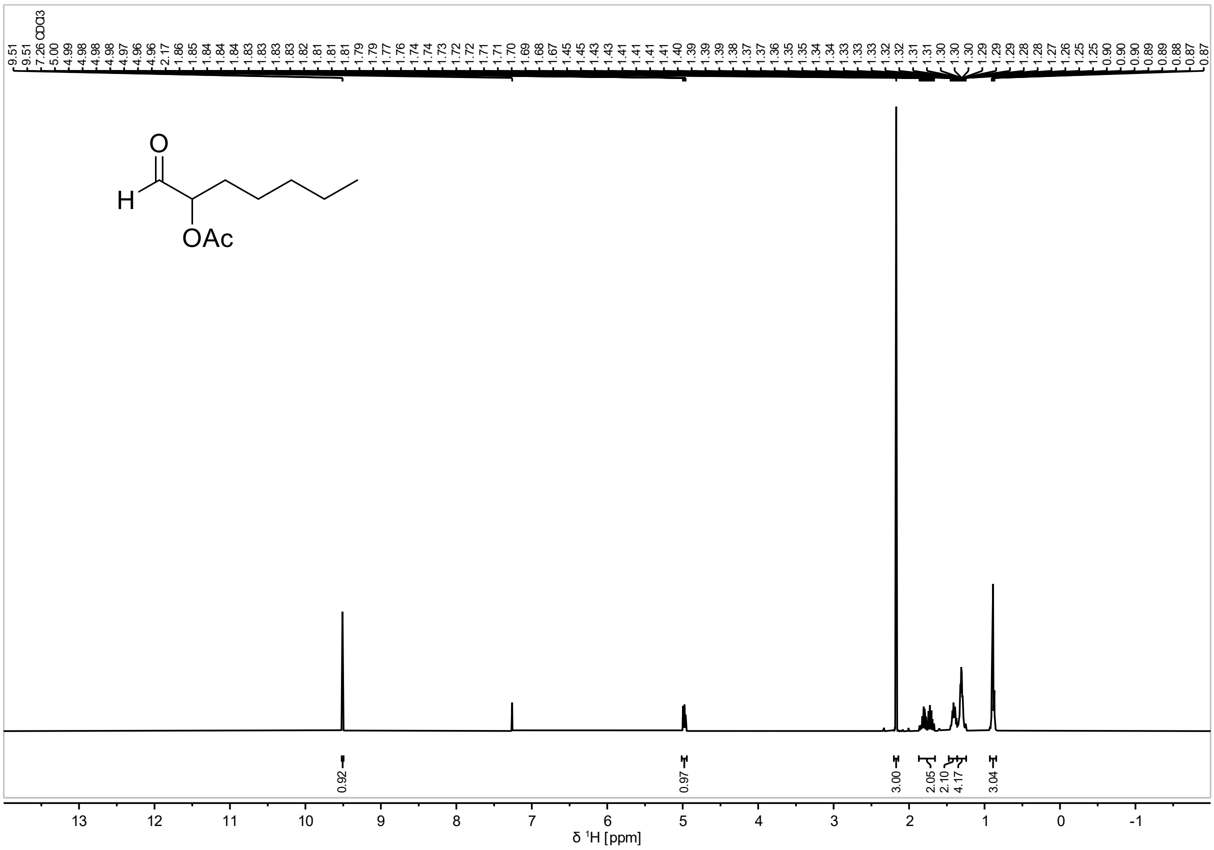

**$^{13}$C NMR** (101 MHz, $CDCl_3$):

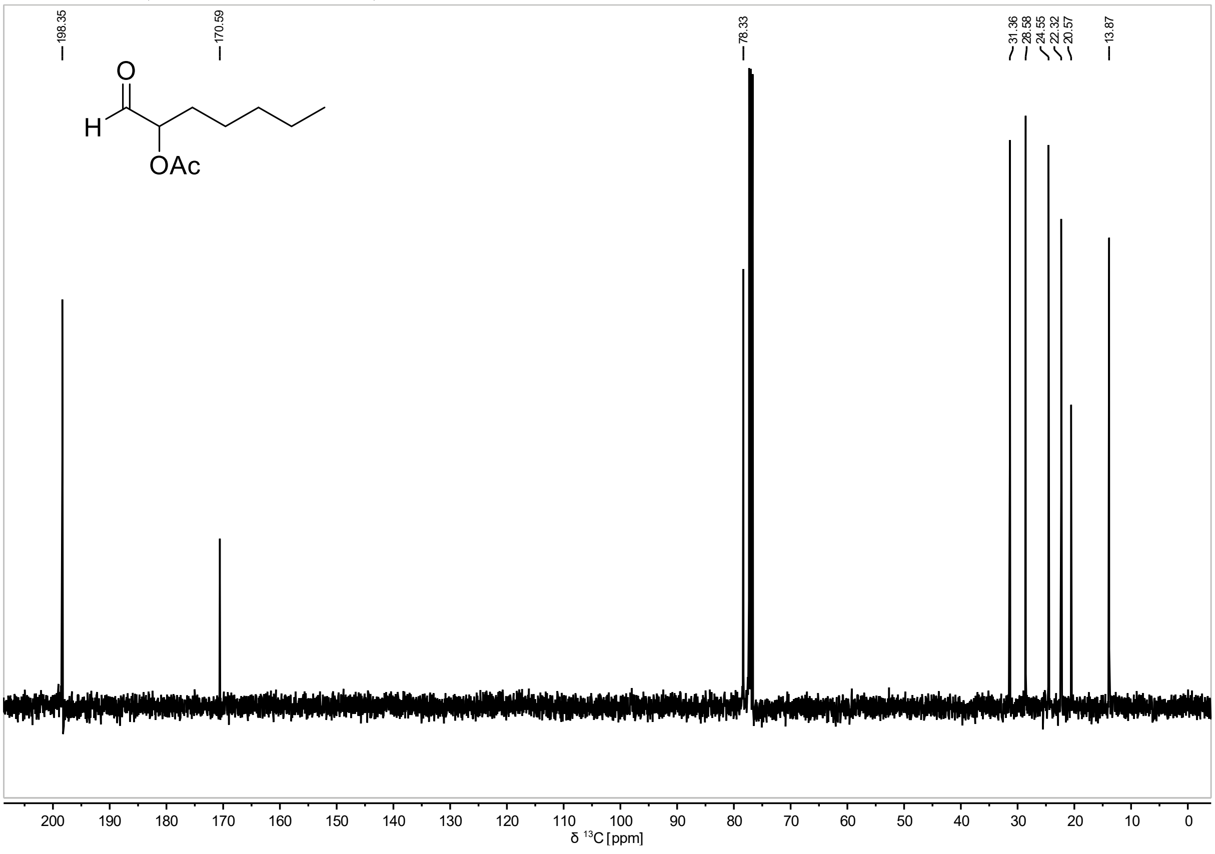

**$^{1}$H NMR** (400 MHz, $CDCl_3$):

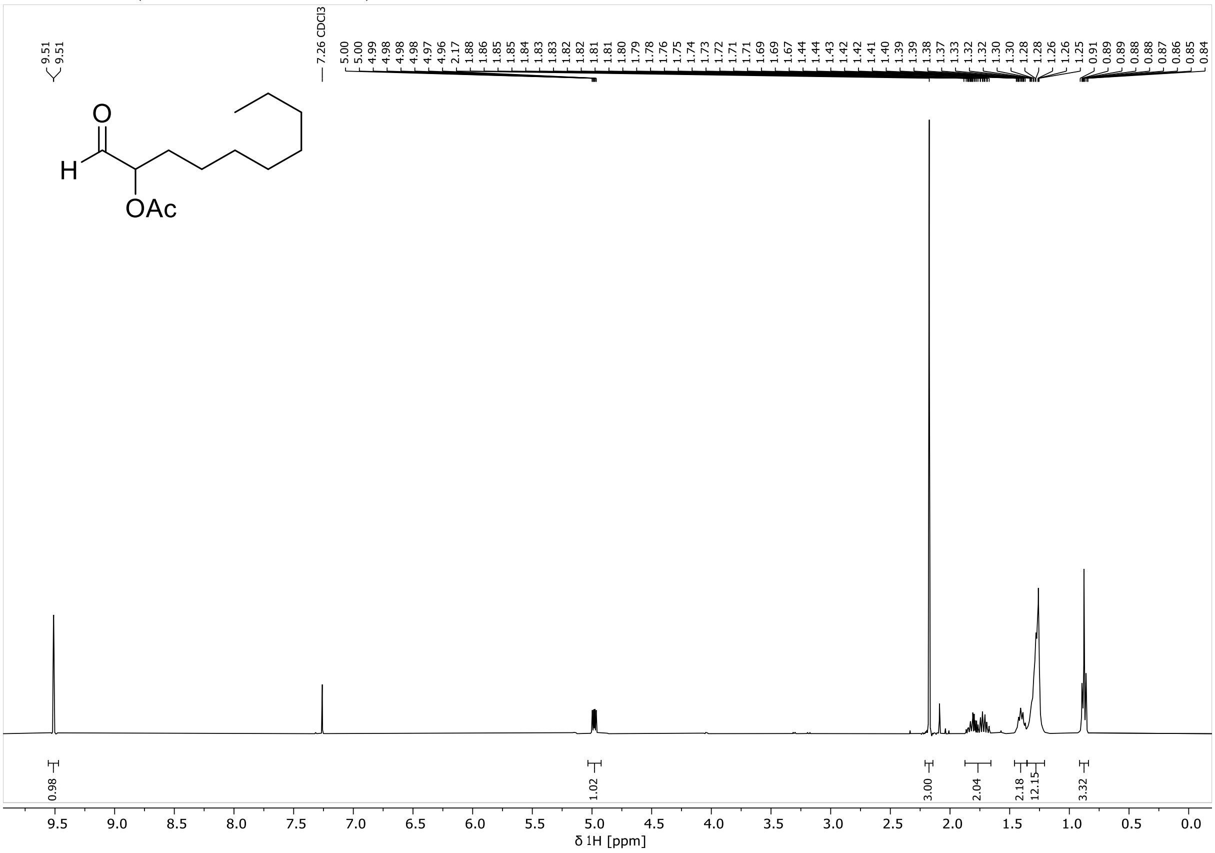


**$^{13}$C NMR** (101 MHz, $CDCl_3$):

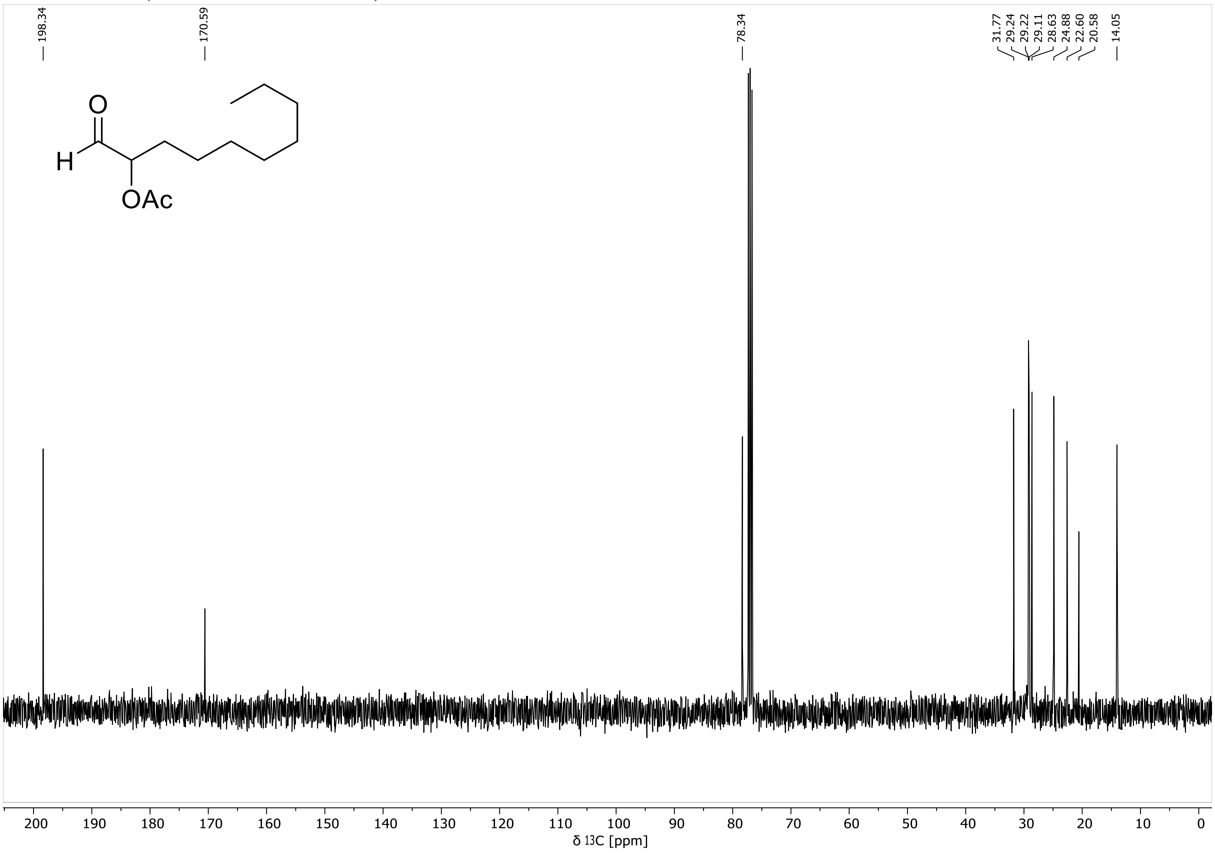

**$^{1}$H NMR** (400 MHz, $CDCl_3$):

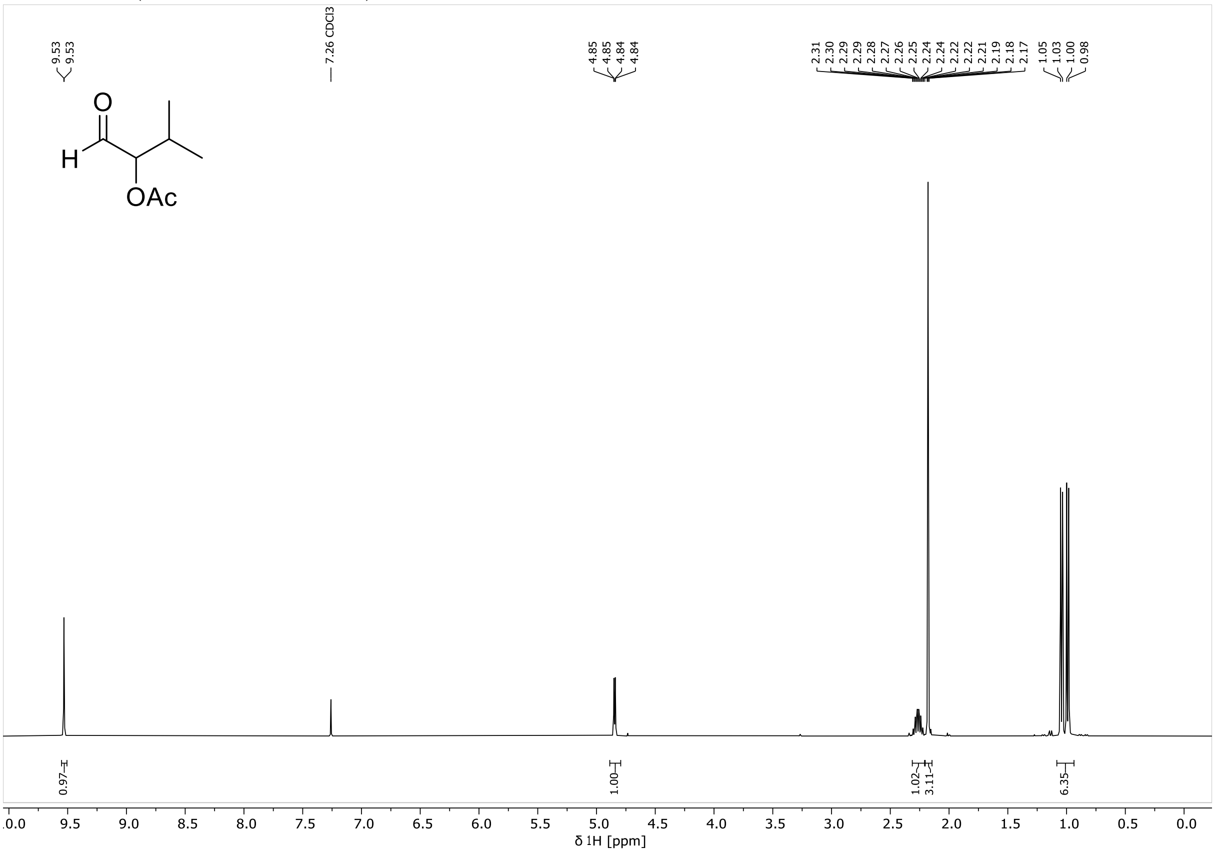


**$^{13}$C NMR** (101 MHz, $CDCl_3$):

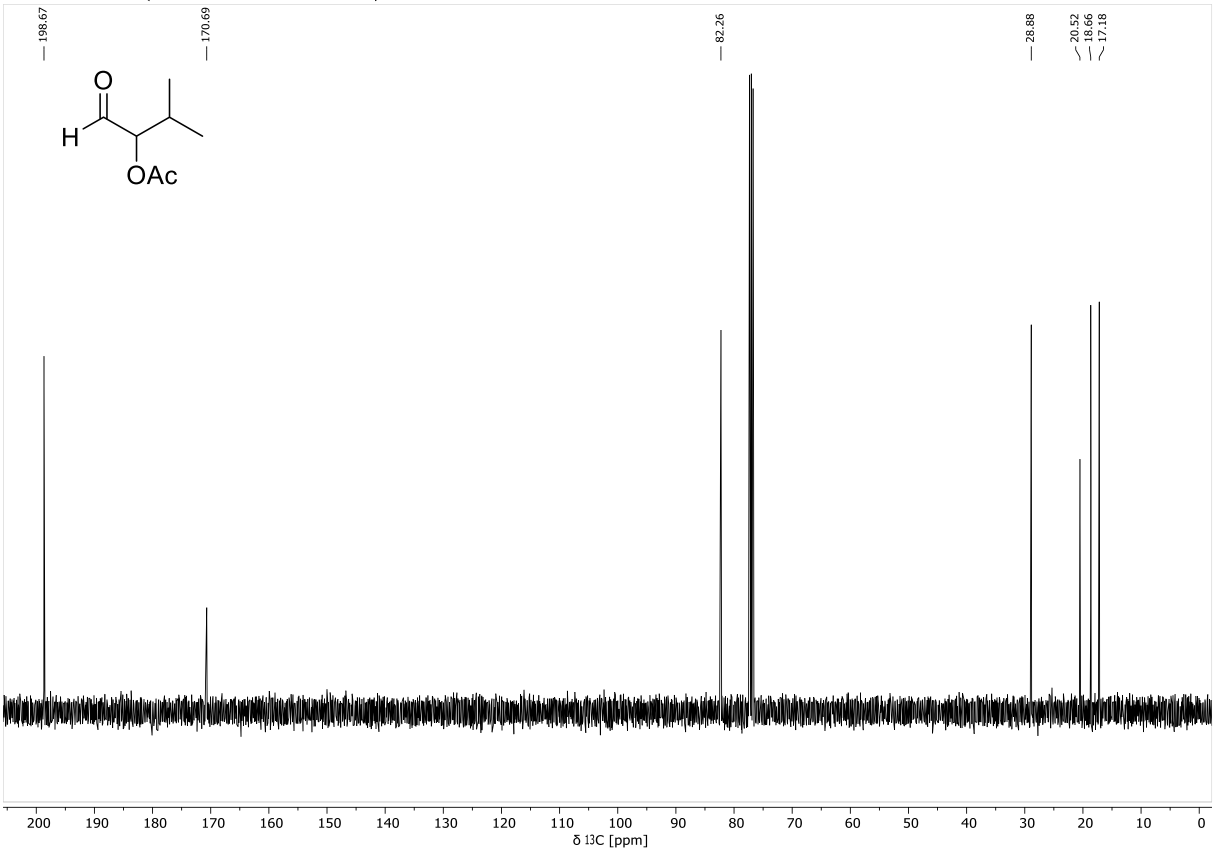

**$^{1}$H NMR** (400 MHz, $CDCl_3$):

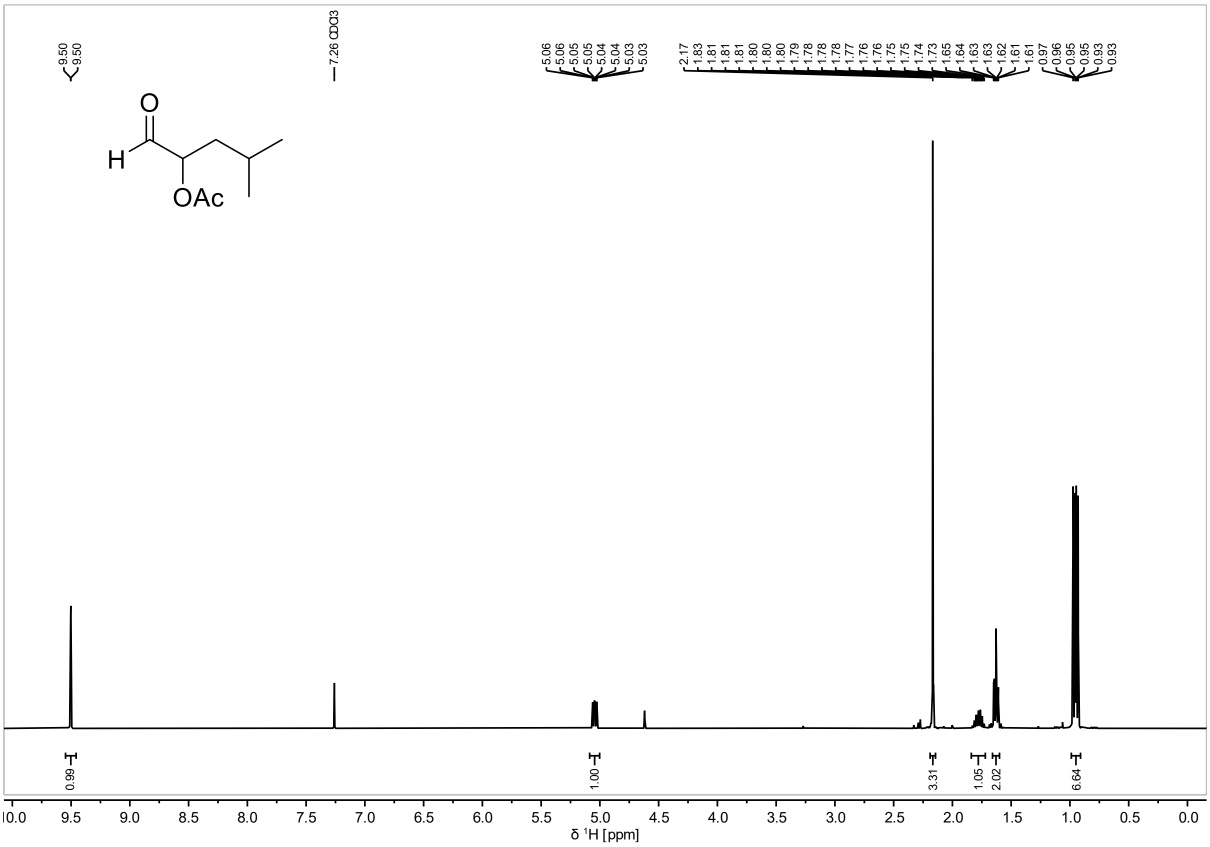


**$^{13}$C NMR** (101 MHz, $CDCl_3$):

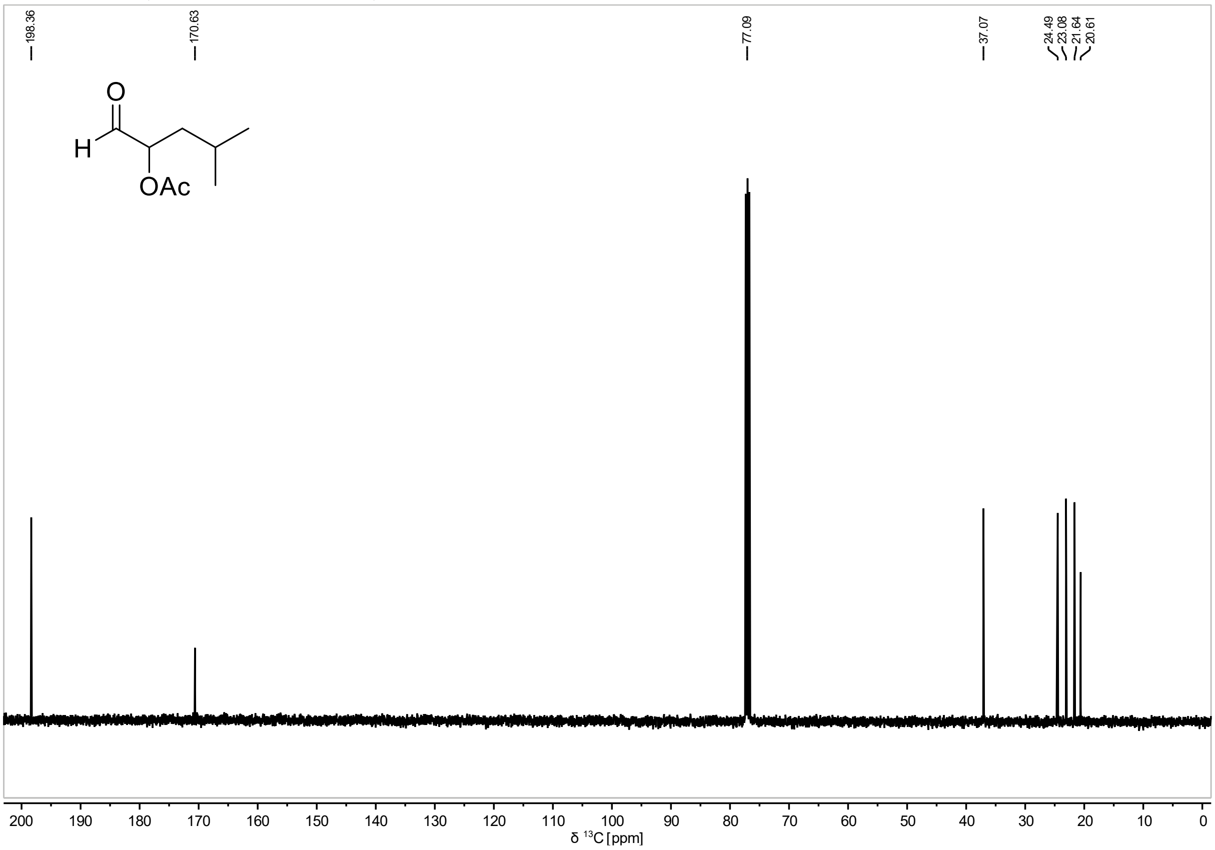

**$^{1}$H NMR** (400 MHz, $CDCl_3$):

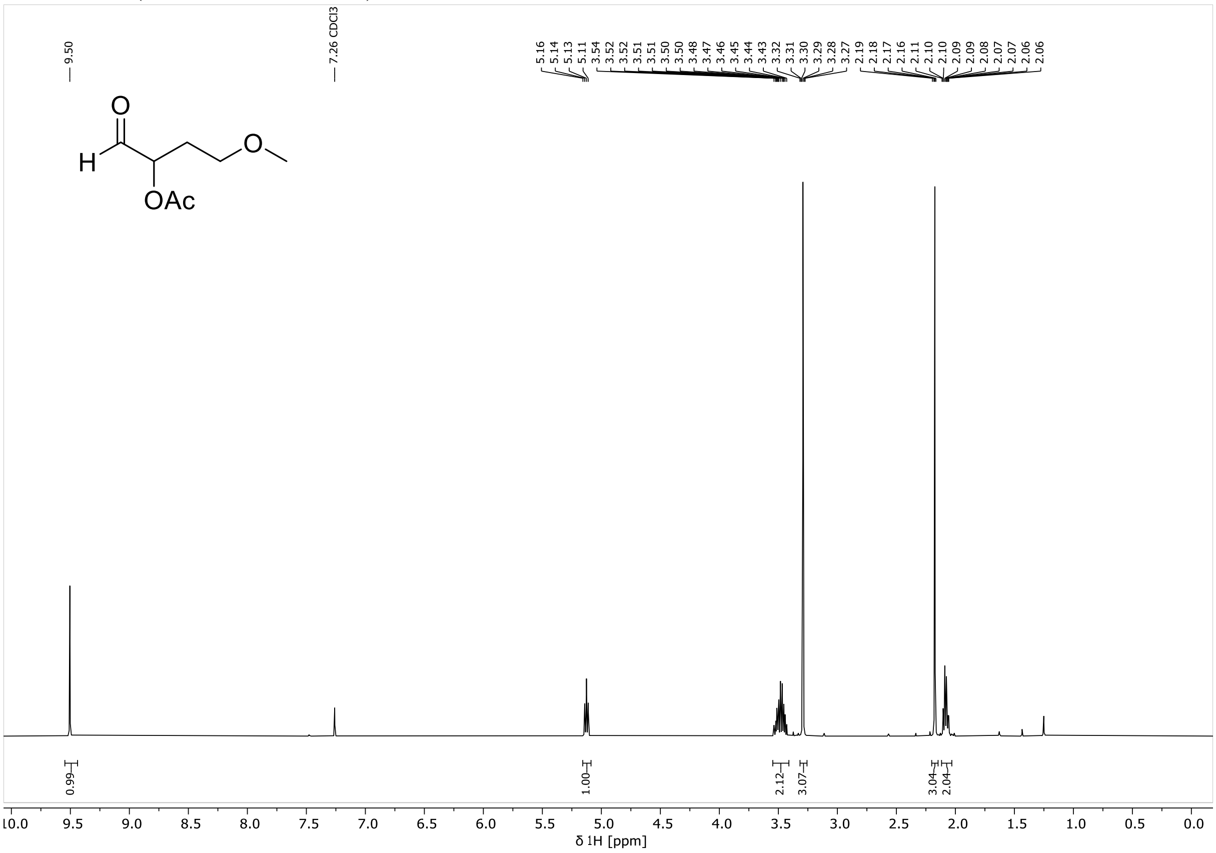


**$^{13}$C NMR** (101 MHz, $CDCl_3$):

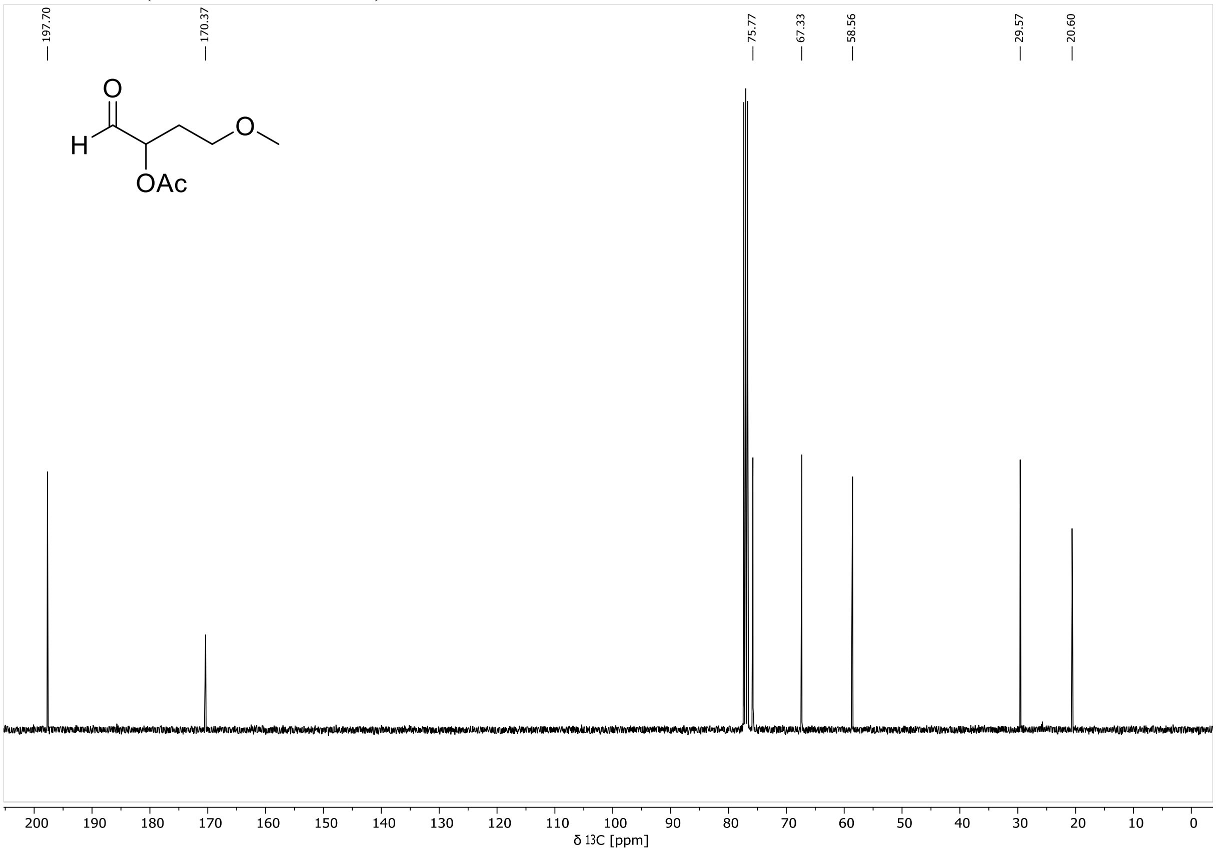

**$^1$H NMR** (400 MHz, $CDCl_3$):

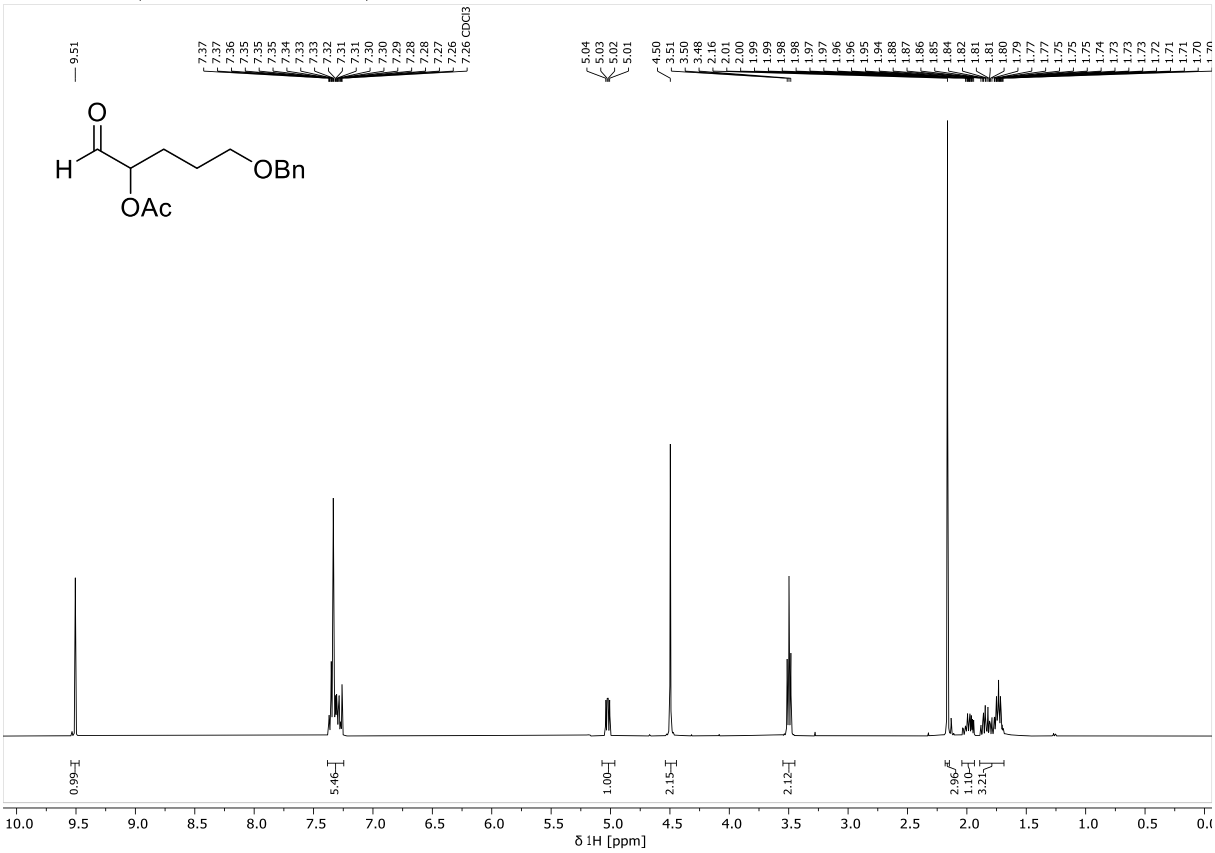


**$^{13}$C NMR** (101 MHz, $CDCl_3$):

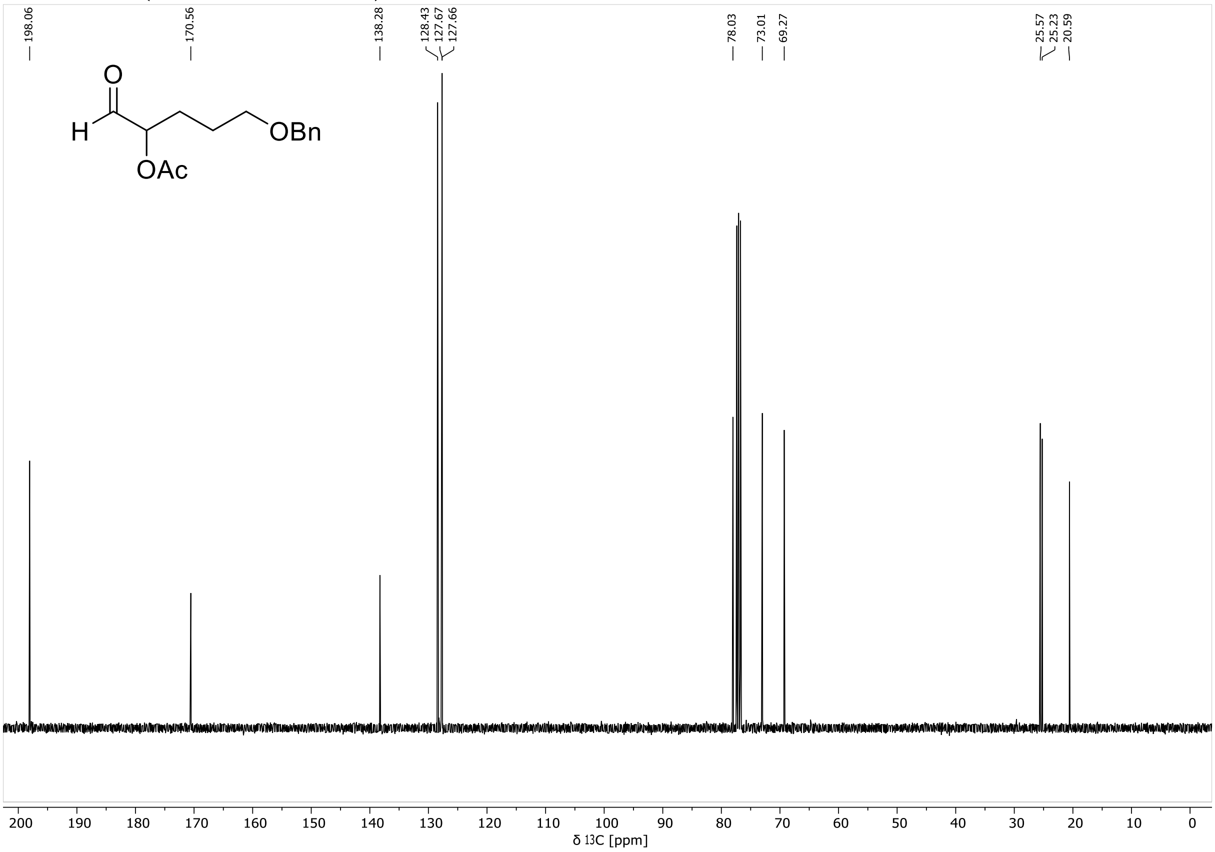

**$^1$H NMR** (400 MHz, $CDCl_3$):

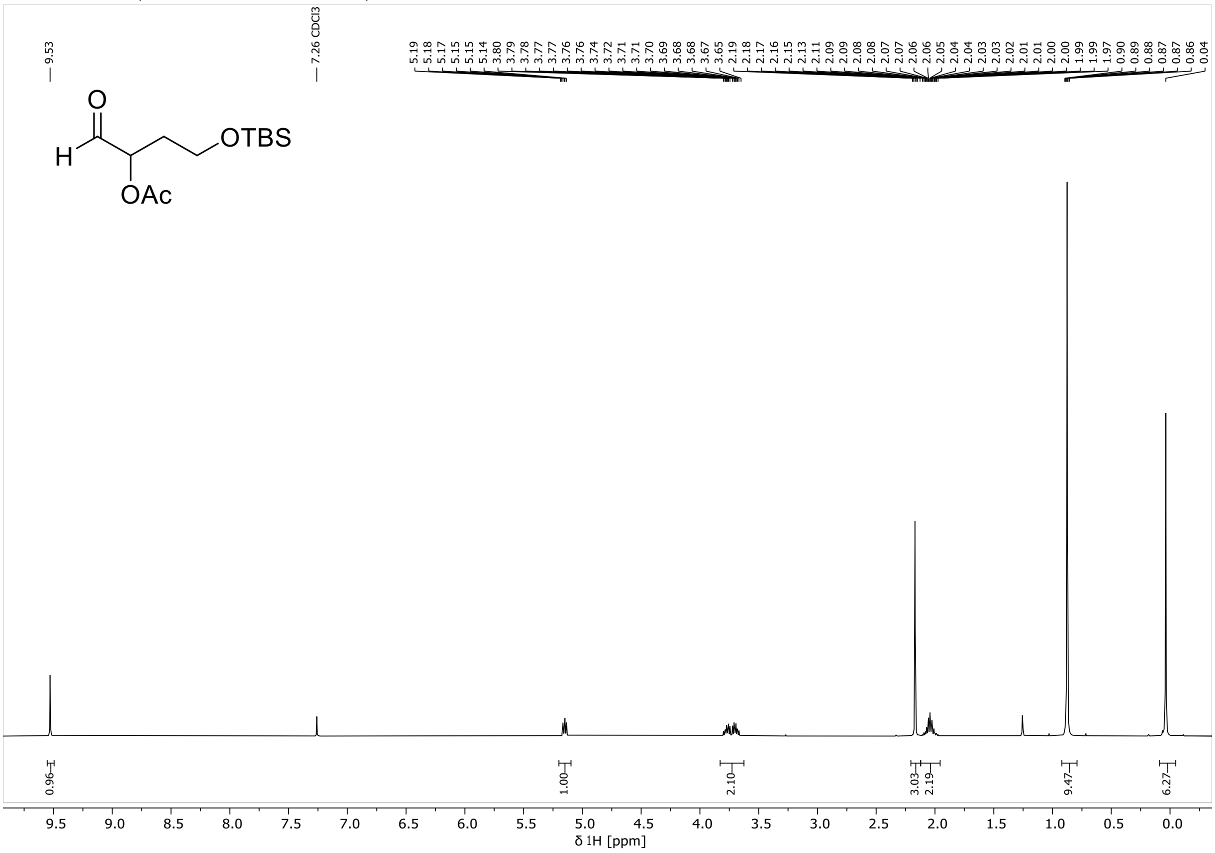


**$^{13}$C NMR** (101 MHz, $CDCl_3$):

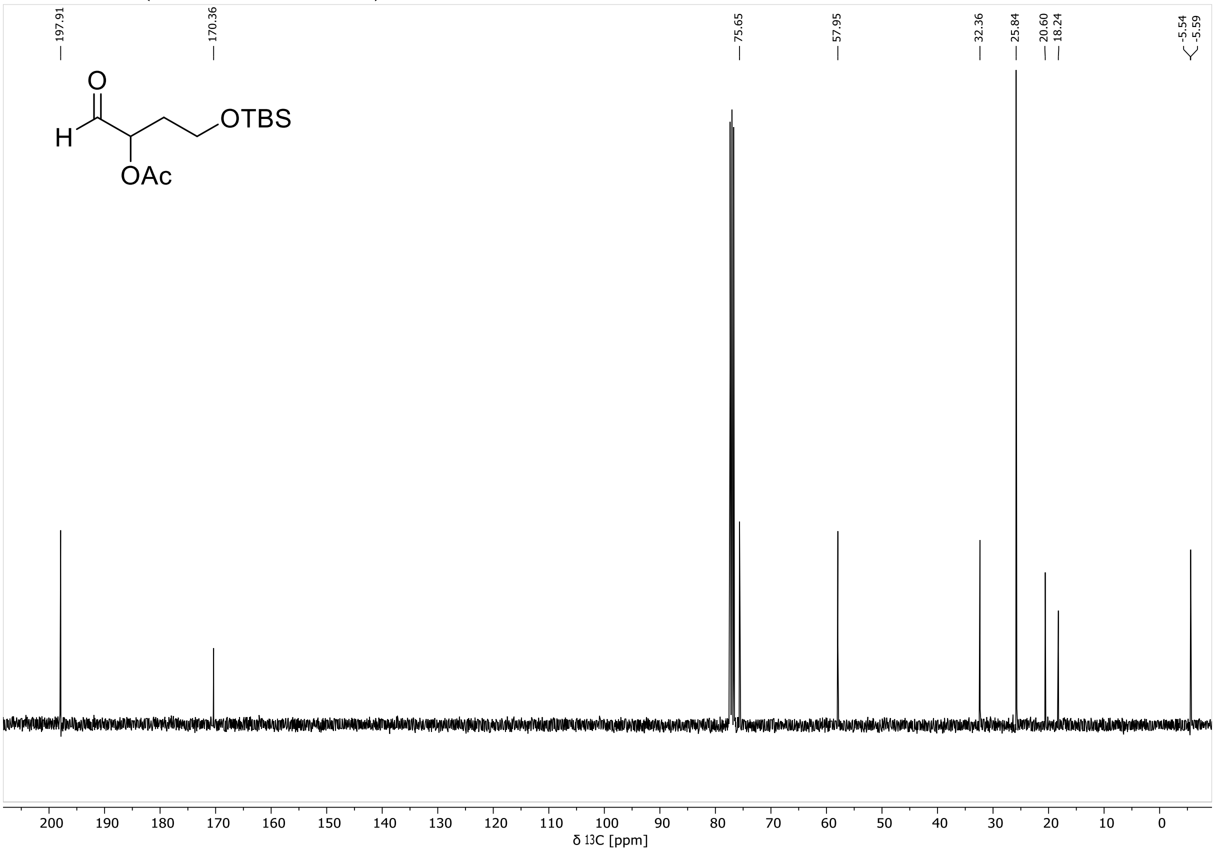

**$^1$H NMR** (300 MHz, $CDCl_3$):

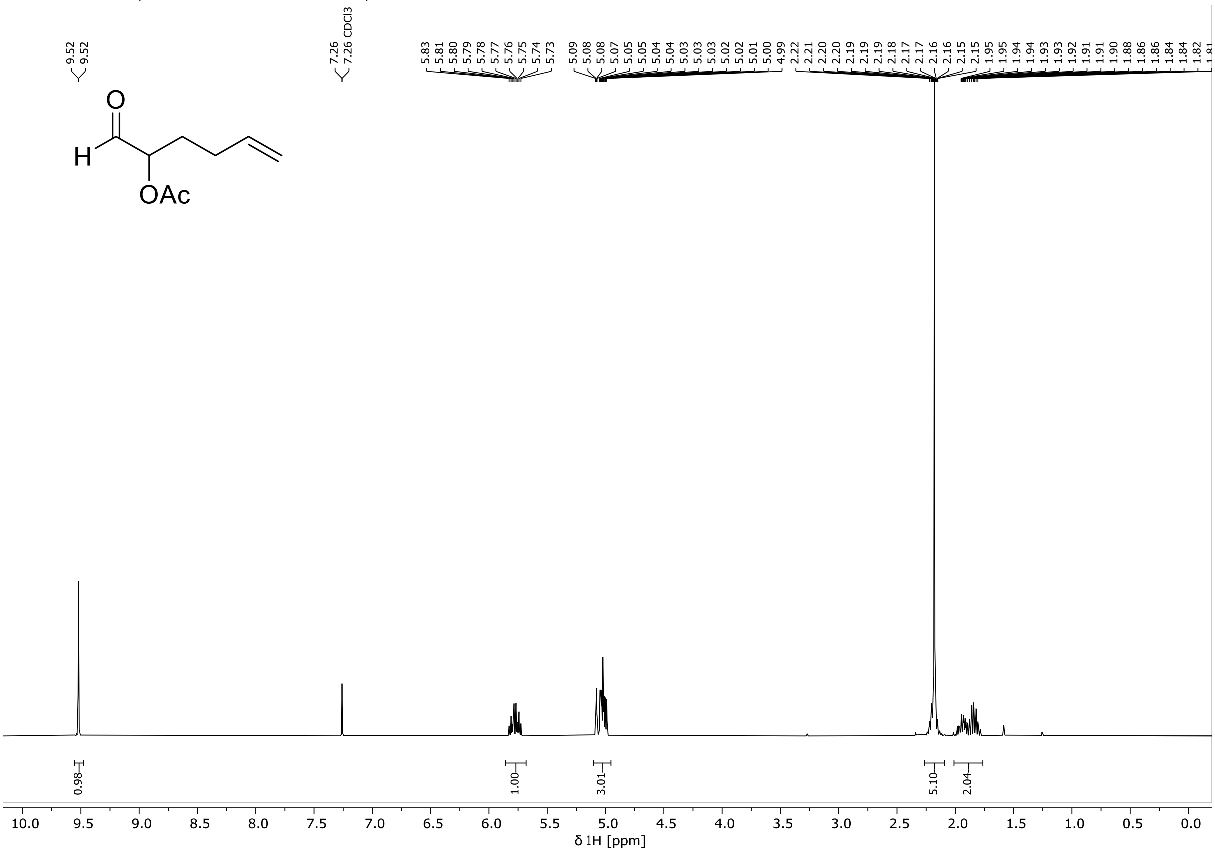


**$^{13}$C NMR** (75 MHz, $CDCl_3$):

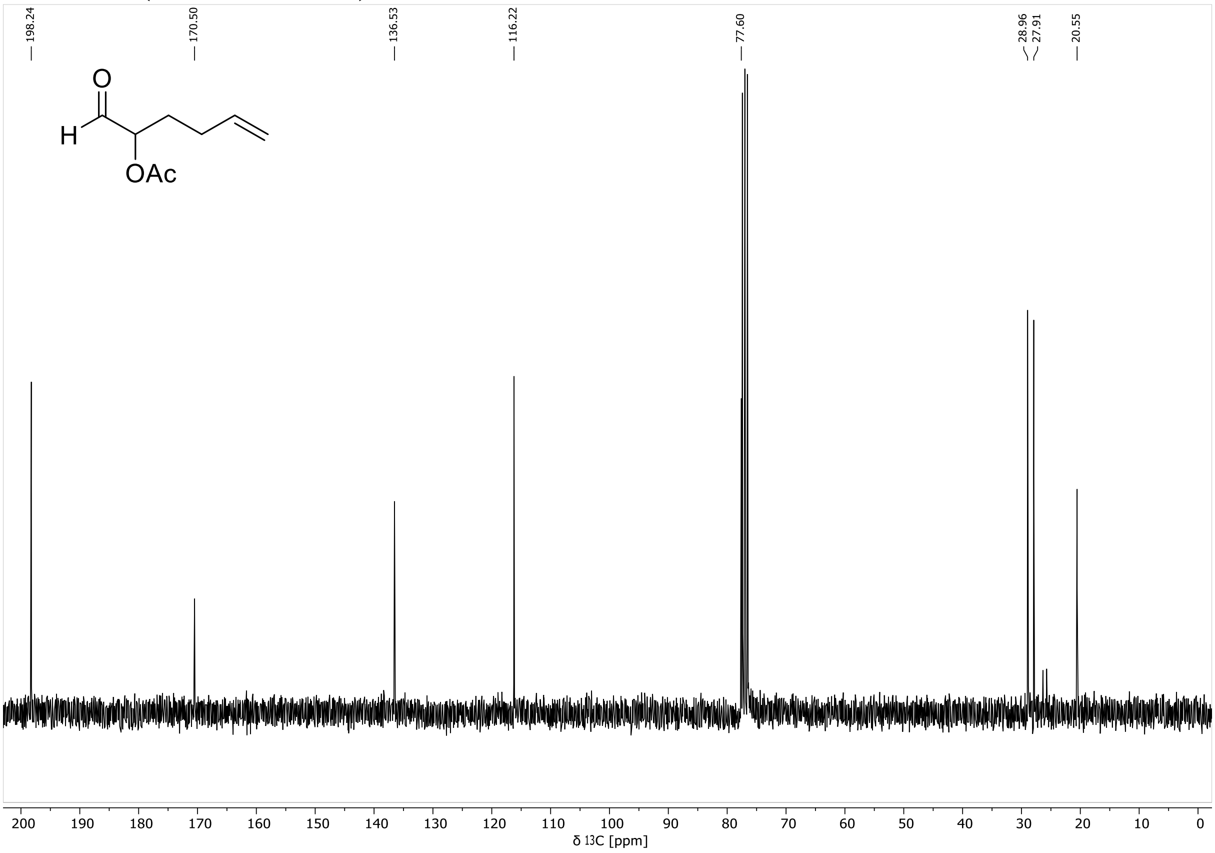

**$^{1}$H NMR** (400 MHz, $CDCl_3$):

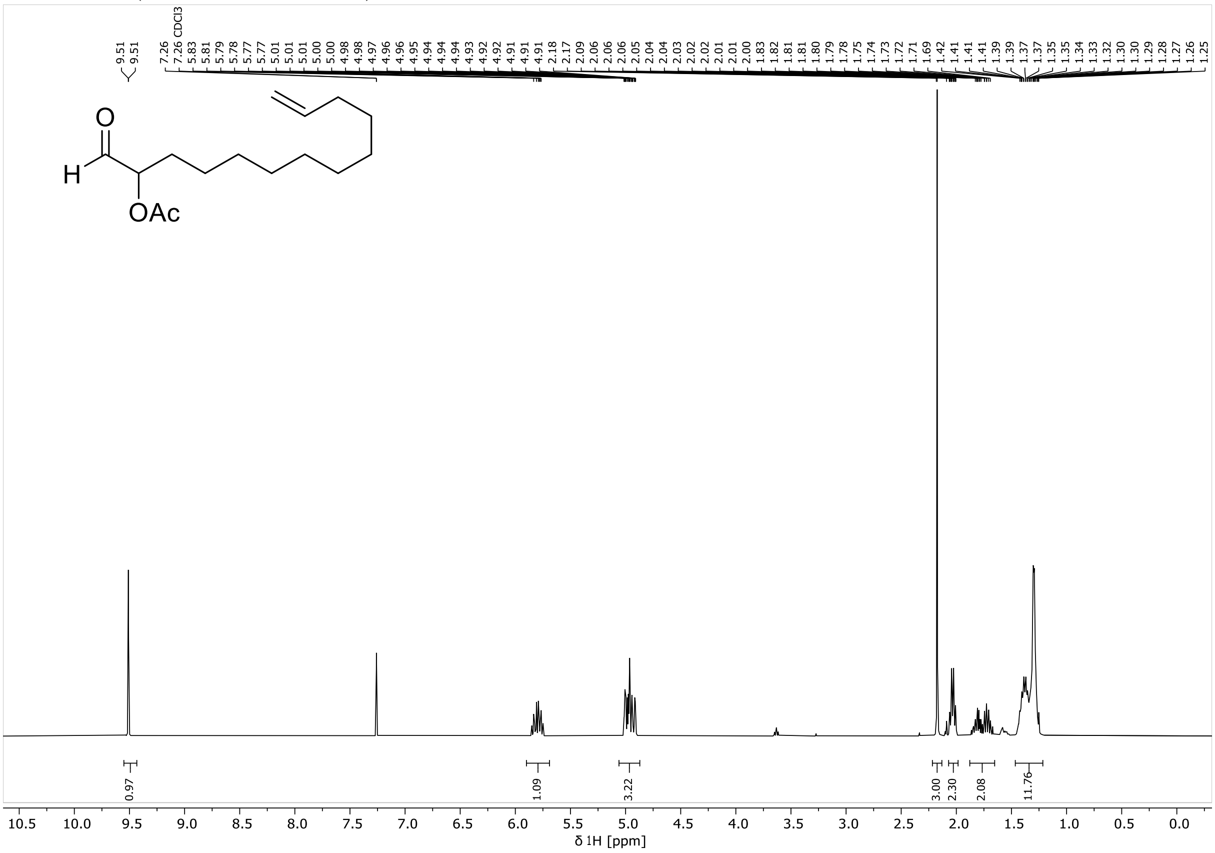


**$^{13}$C NMR** (101 MHz, $CDCl_3$):

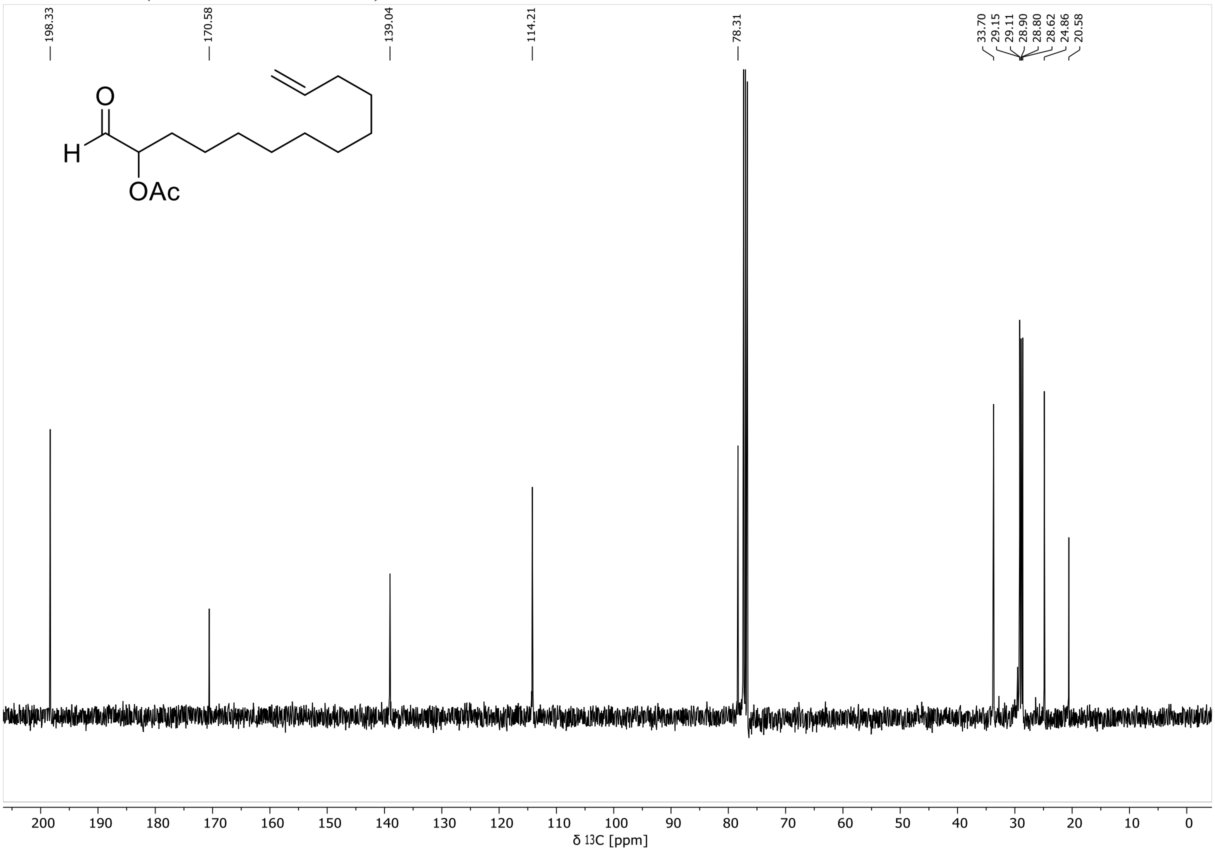

**$^{1}$H NMR** (400 MHz, $CDCl_3$):

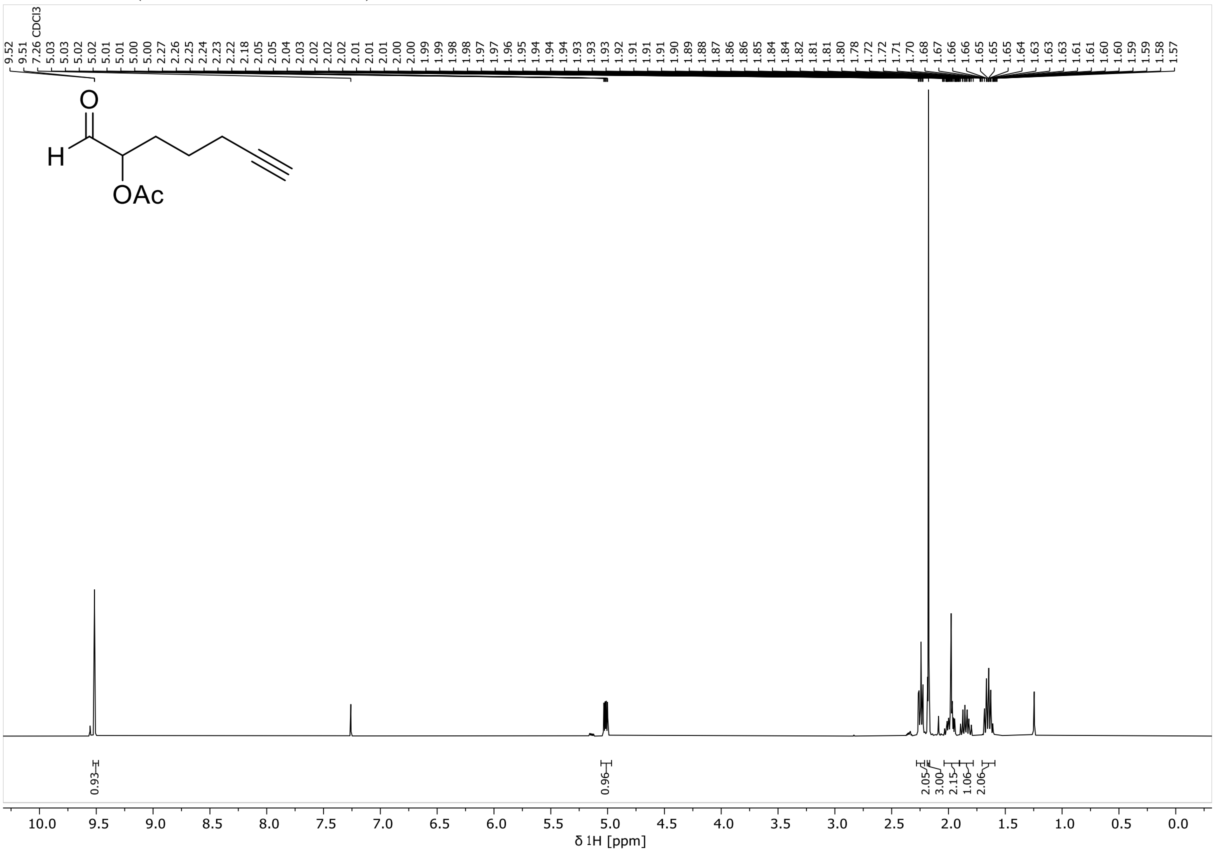

**$^{13}$C NMR** (101 MHz, $CDCl_3$):

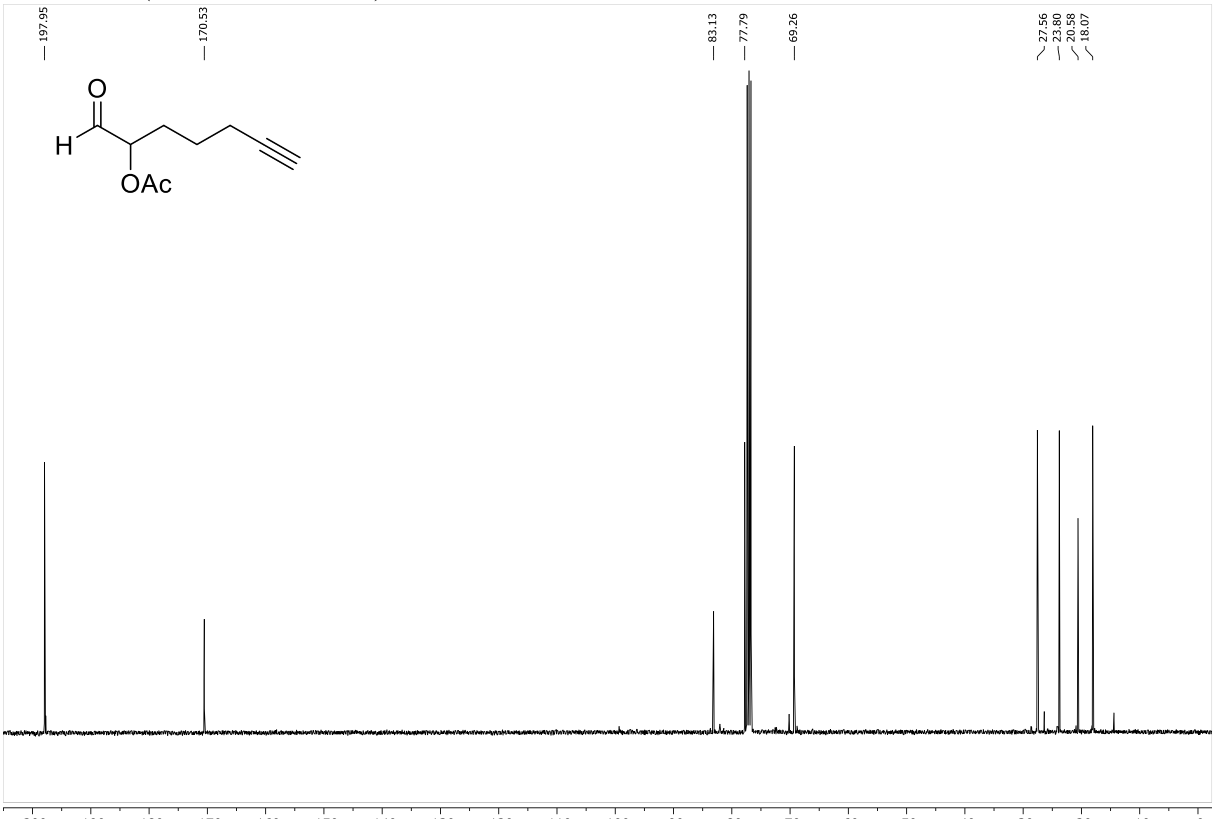

**$^{1}$H NMR** (300 MHz, $CDCl_3$):

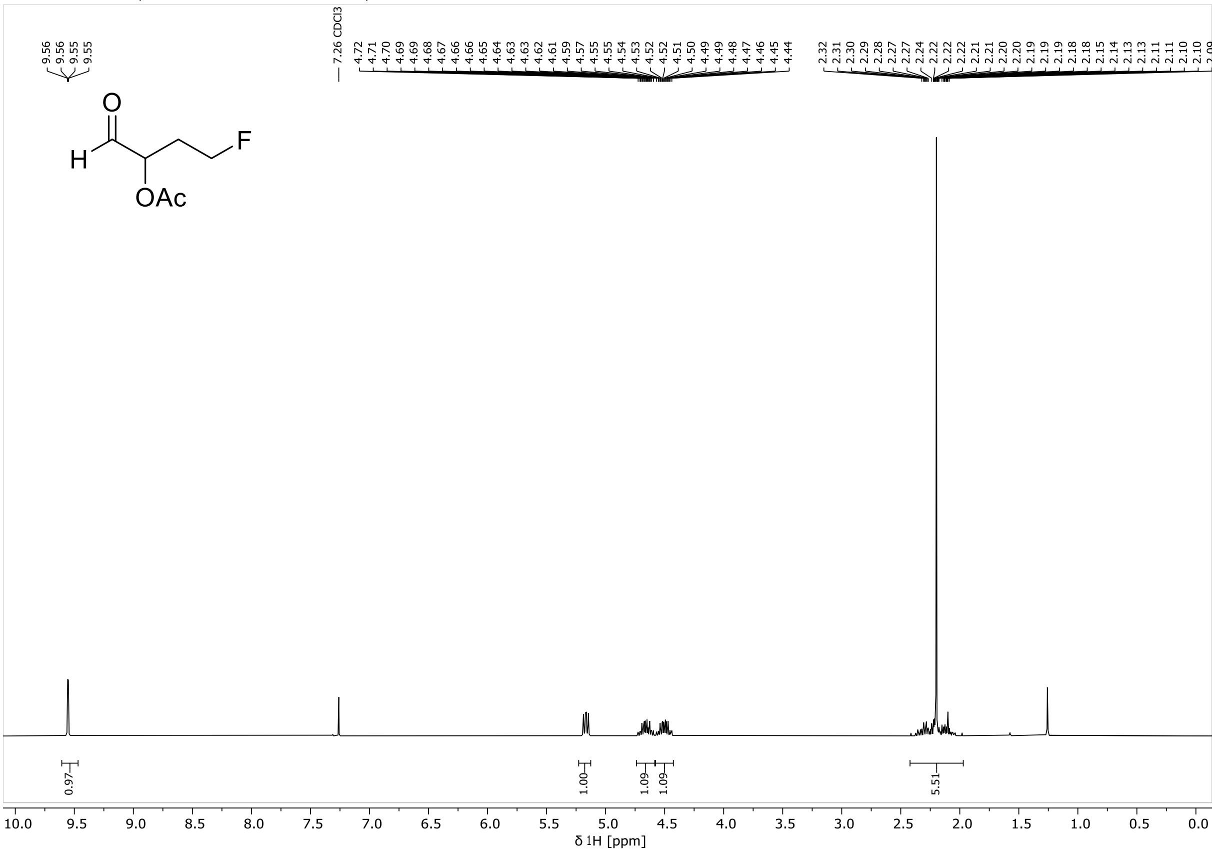

**$^{13}$C NMR** (75 MHz, $CDCl_3$):

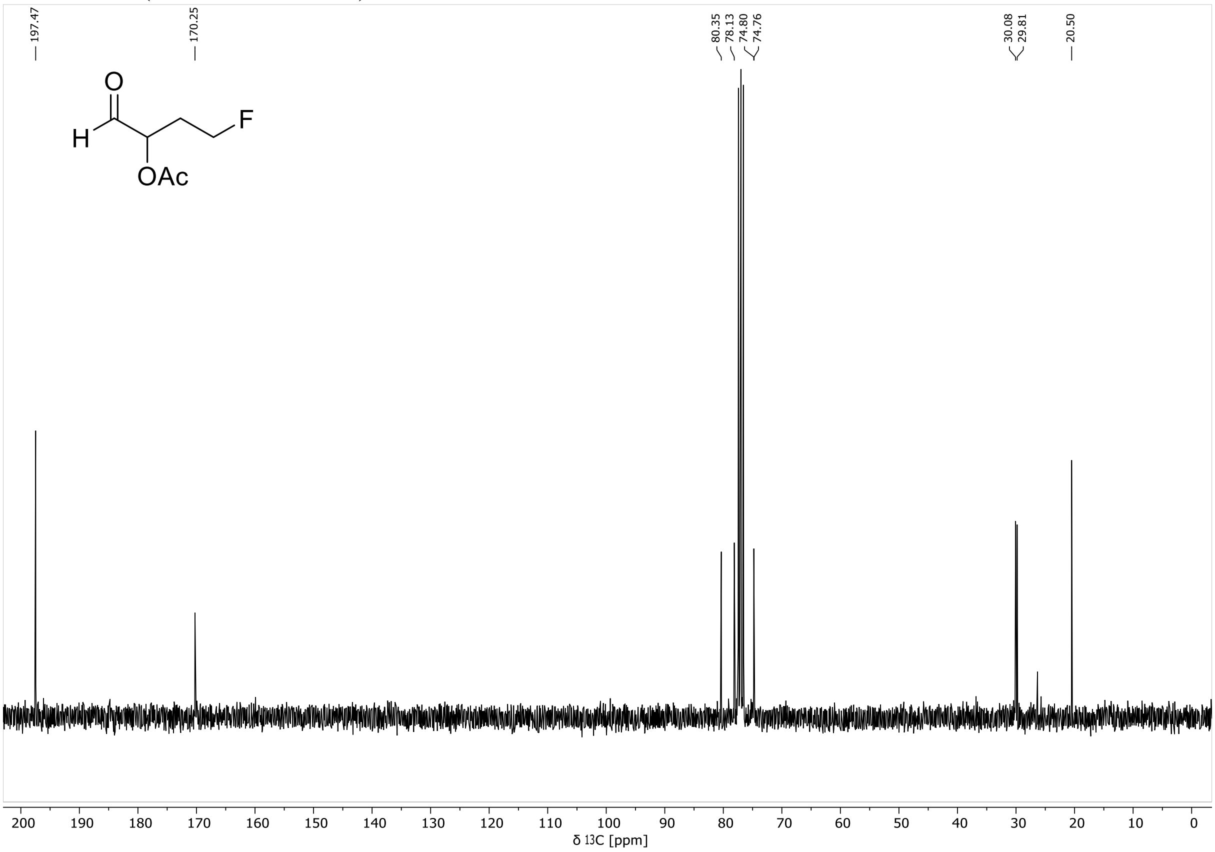

**$^{19}$F NMR** (376 MHz, $CDCl_3$):

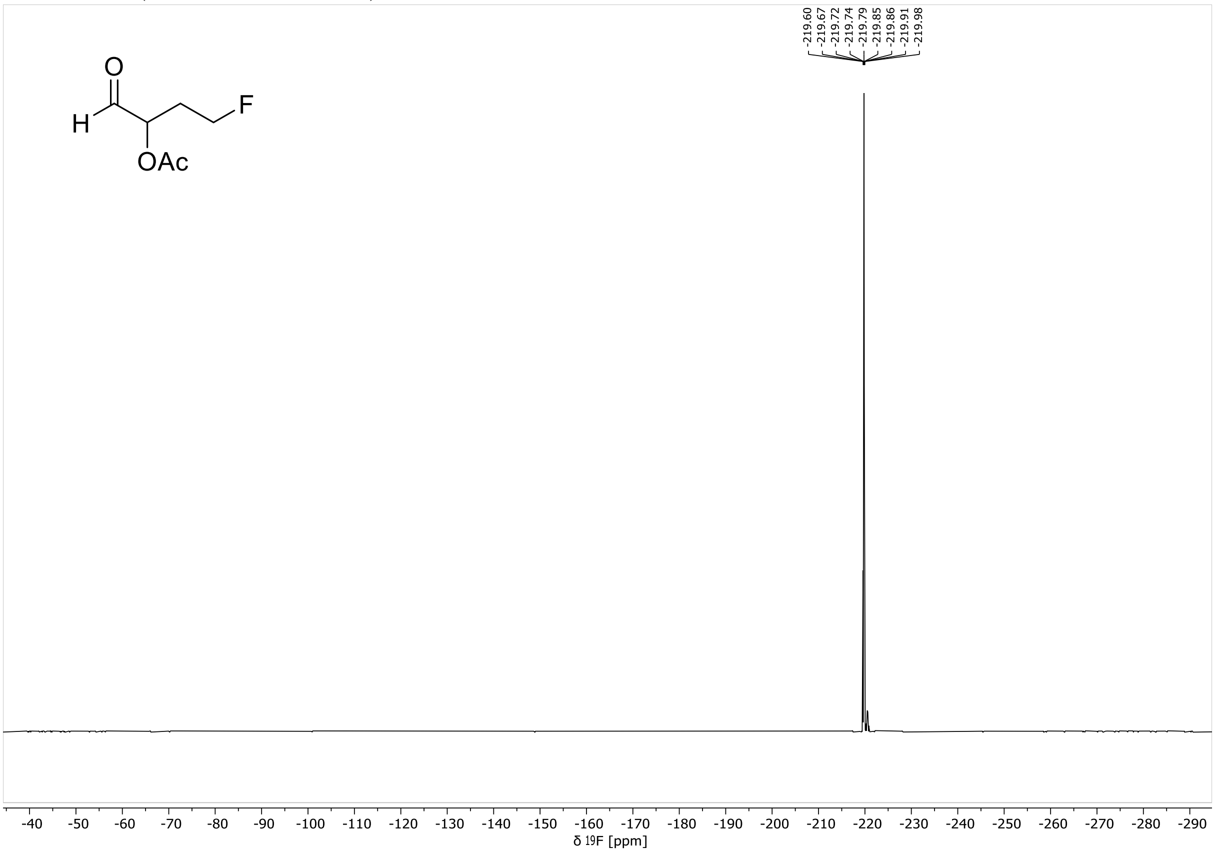

**$^{1}$H NMR** (400 MHz, $CDCl_3$):

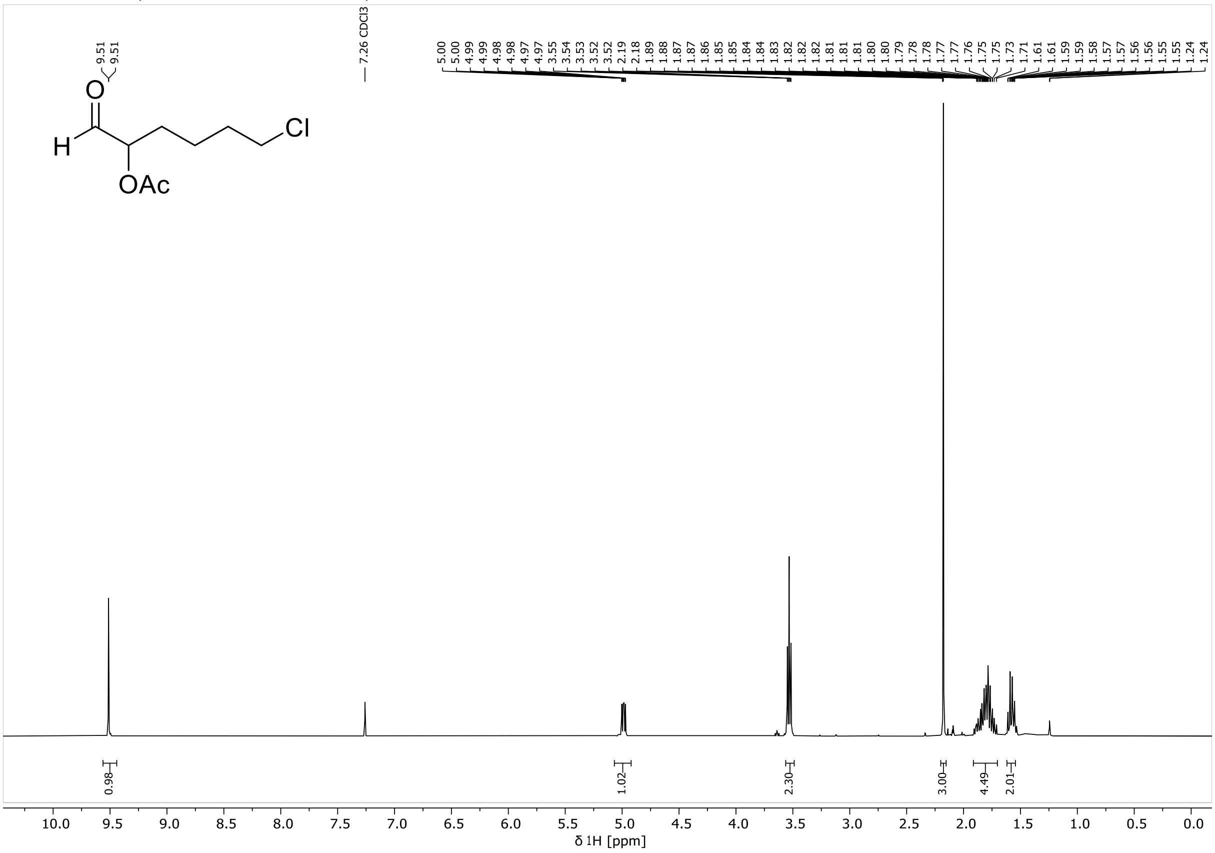

**$^{13}$C NMR** (101 MHz, $CDCl_3$):

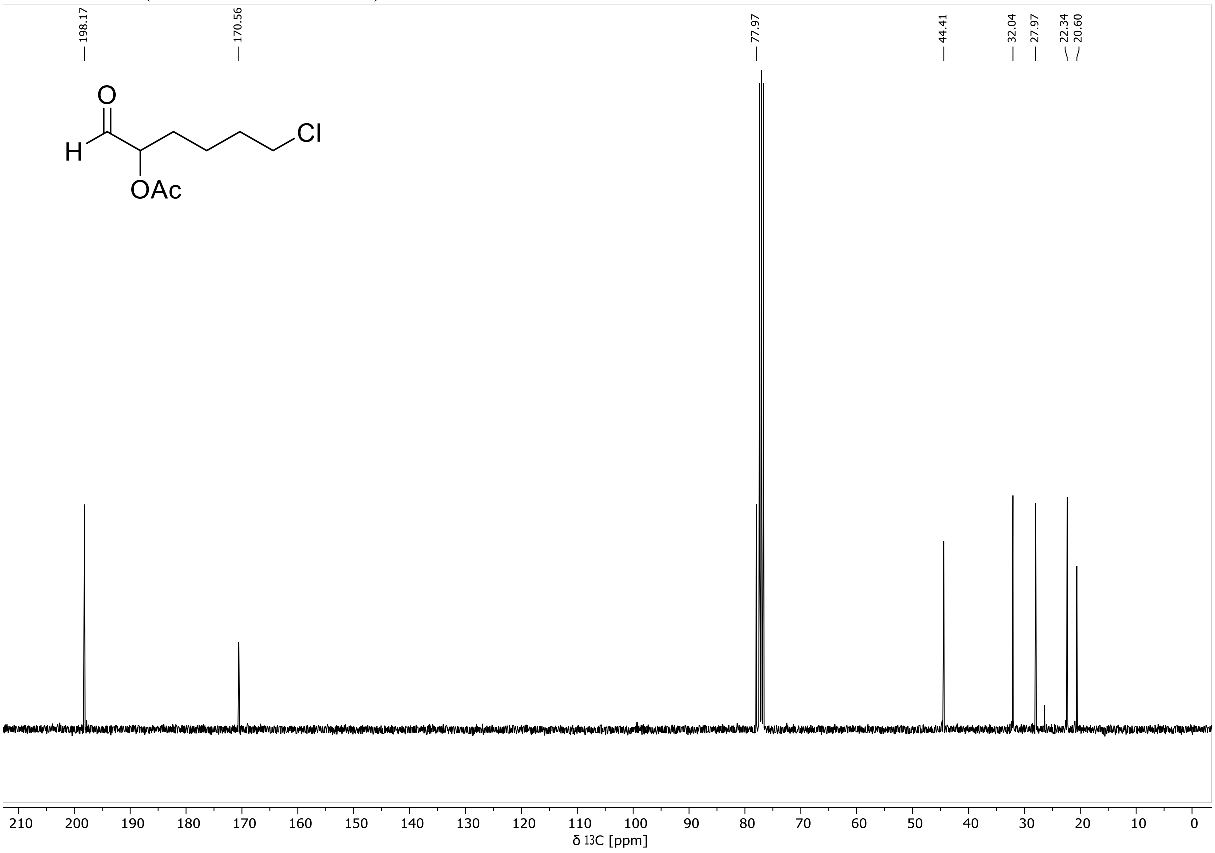

**$^{1}$H NMR** (400 MHz, $CDCl_3$):

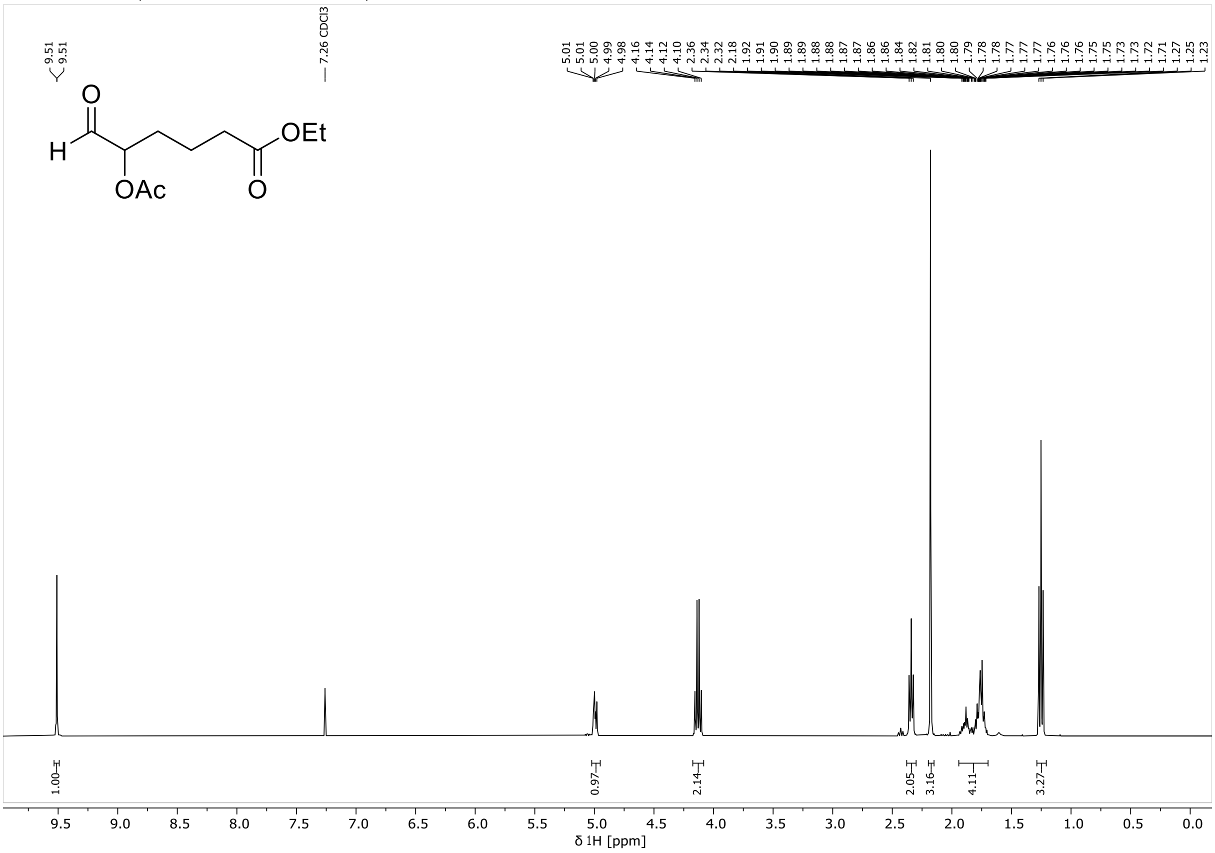


**$^{13}$C NMR** (101 MHz, $CDCl_3$):

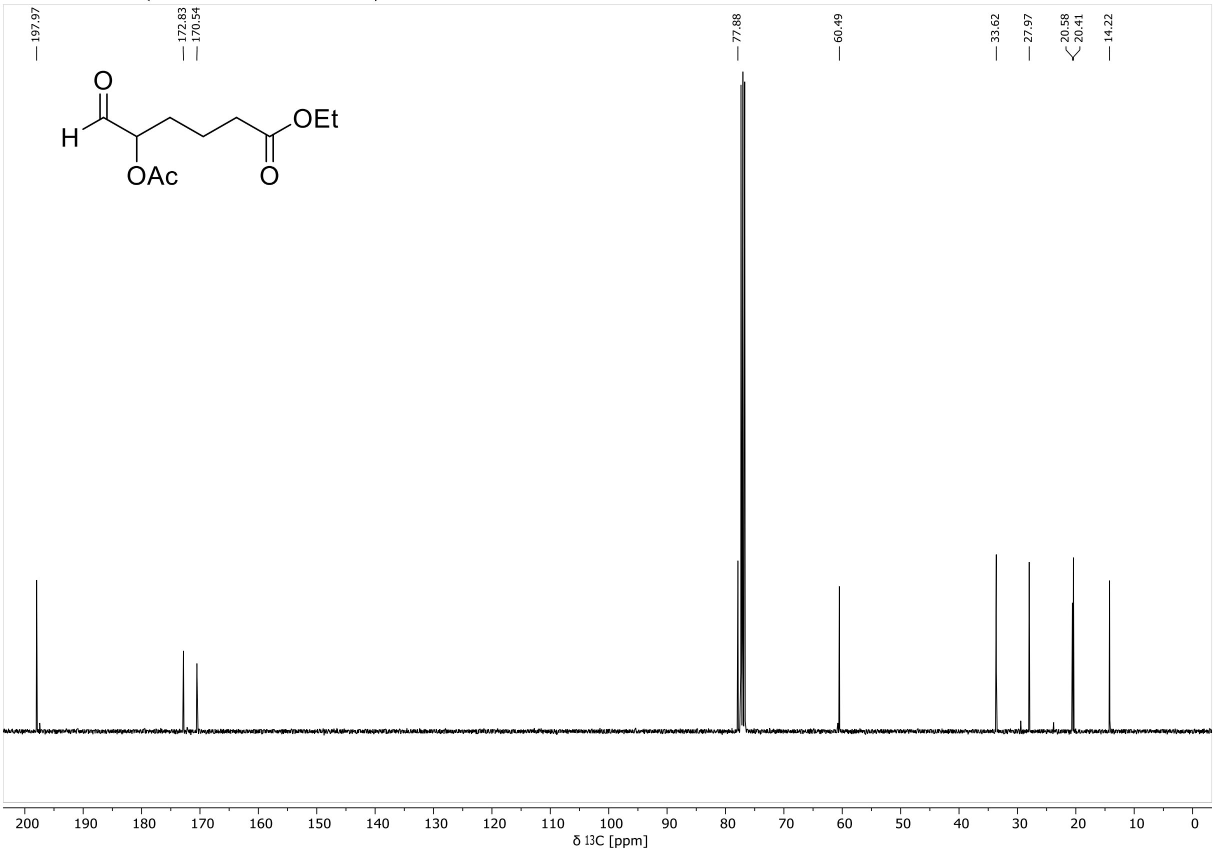

**$^1$H NMR** (400 MHz, $CDCl_3$):

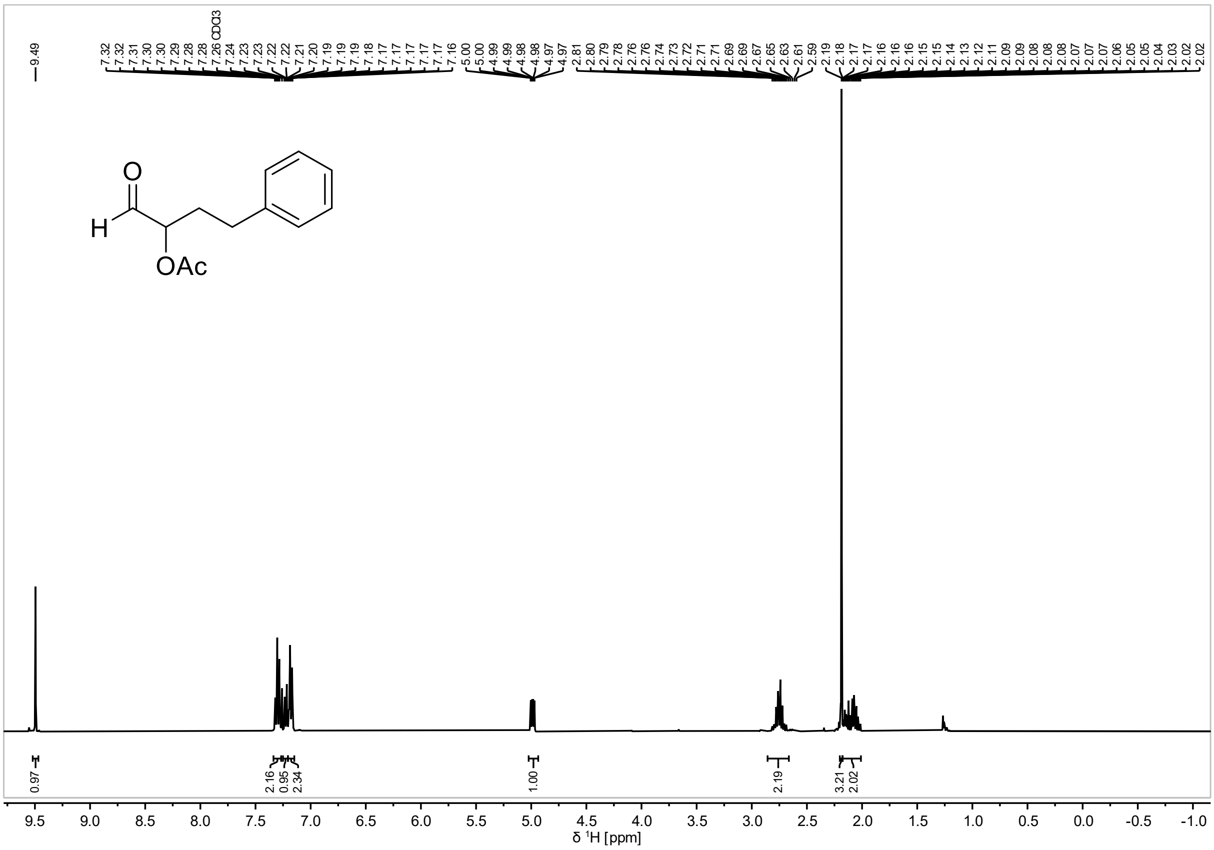

**$^{13}$C NMR** (101 MHz, $CDCl_3$):

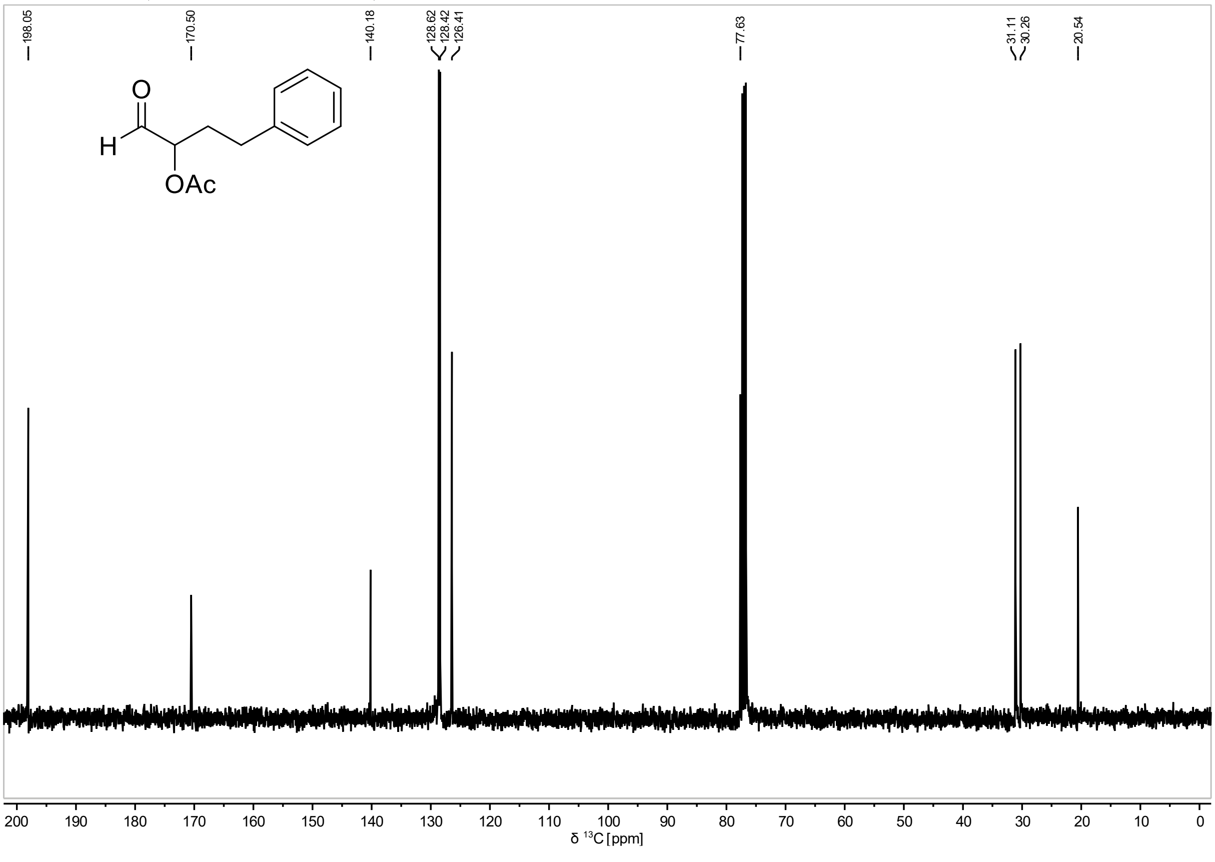

**$^{1}$H NMR** (400 MHz, $CDCl_3$):

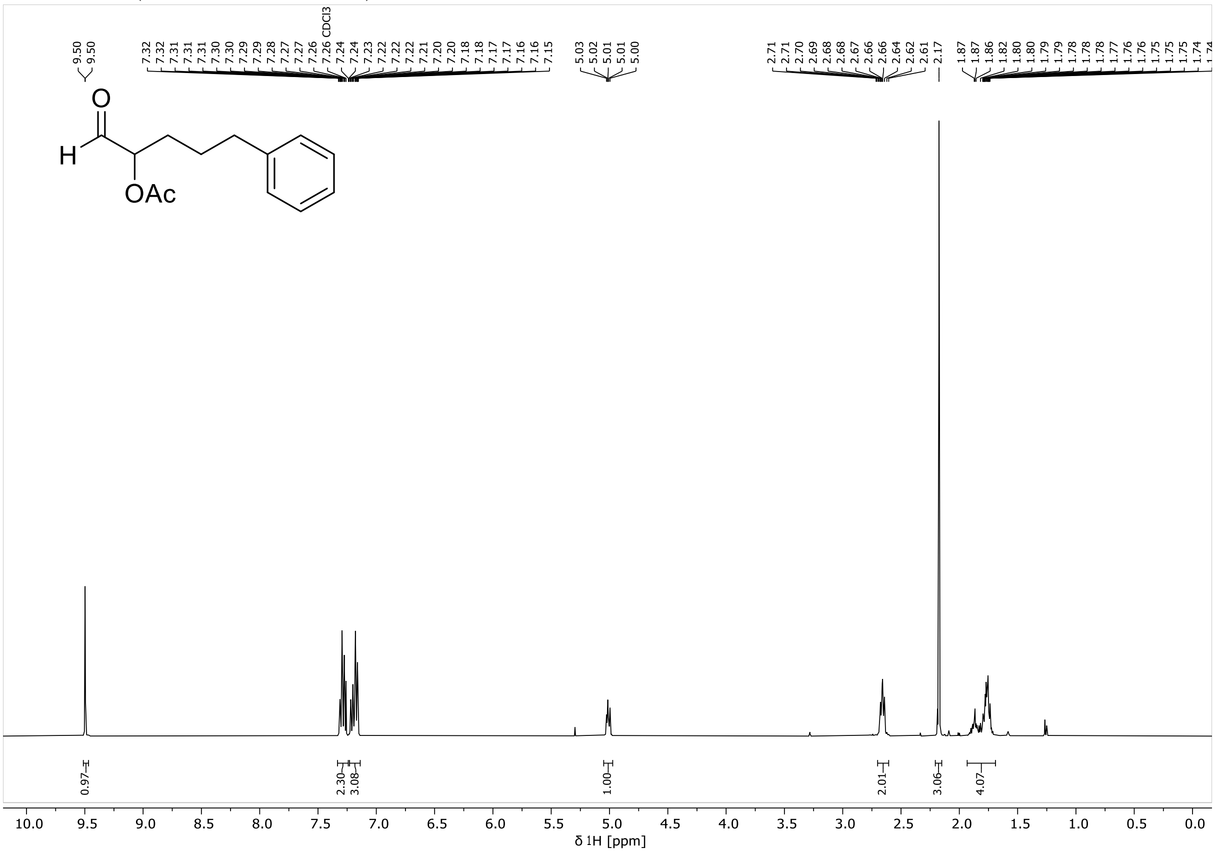

**$^{13}$C NMR** (101 MHz, $CDCl_3$):

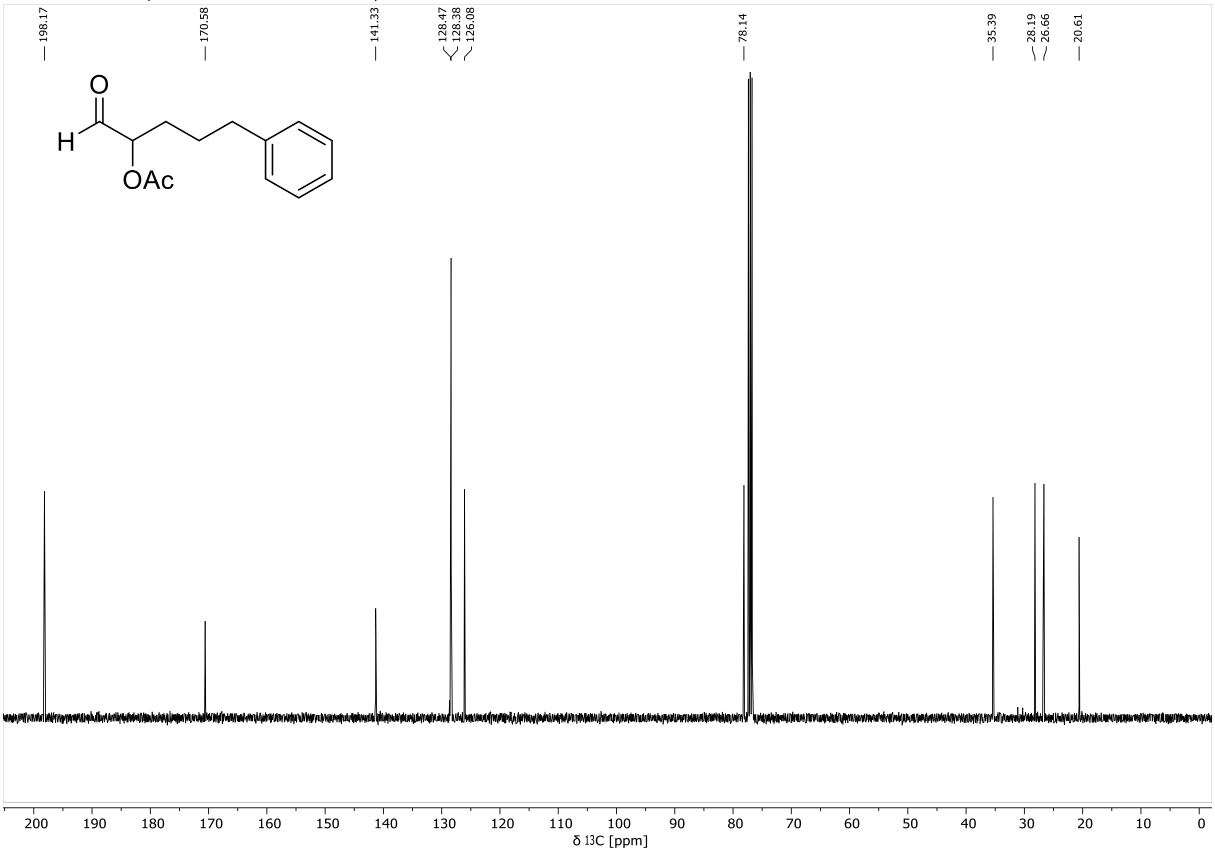

**$^{1}$H NMR** (400 MHz, $CDCl_3$):

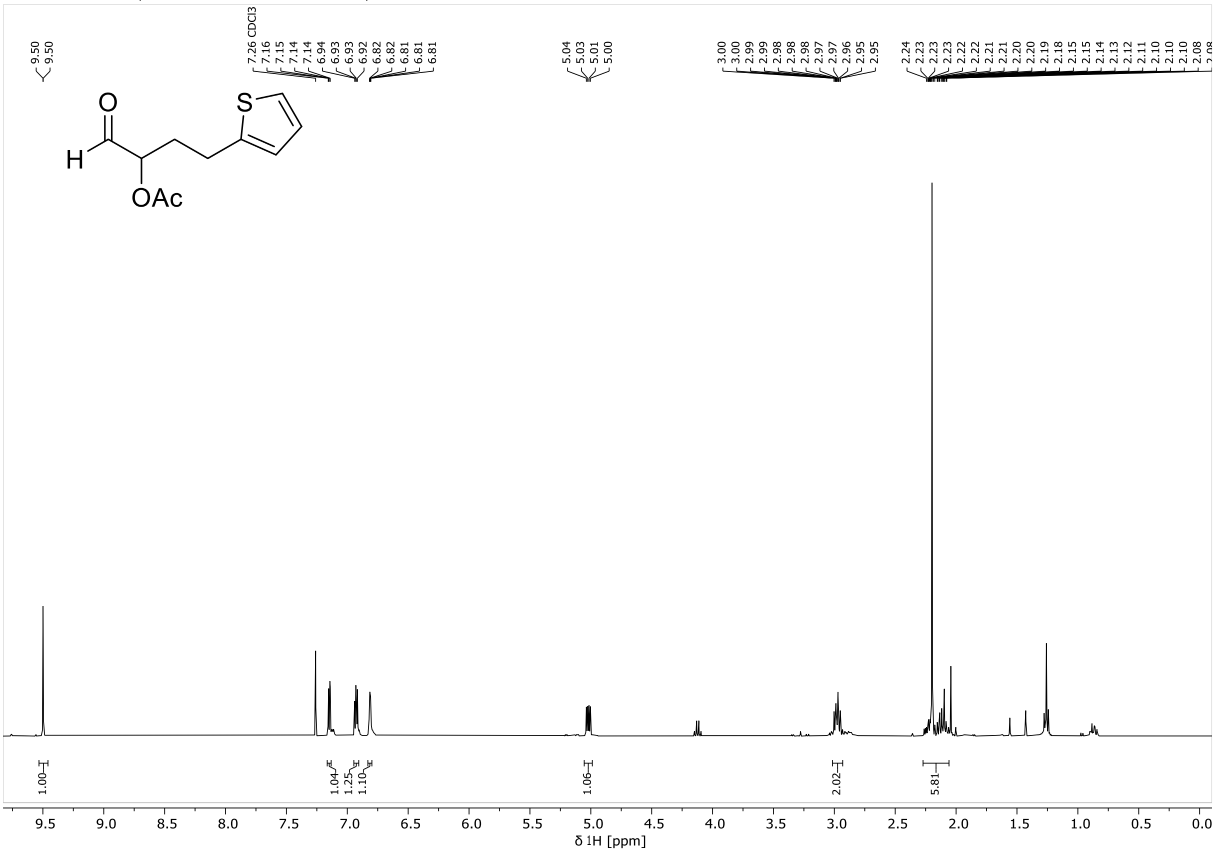

**$^{13}$C NMR** (101 MHz, $CDCl_3$):

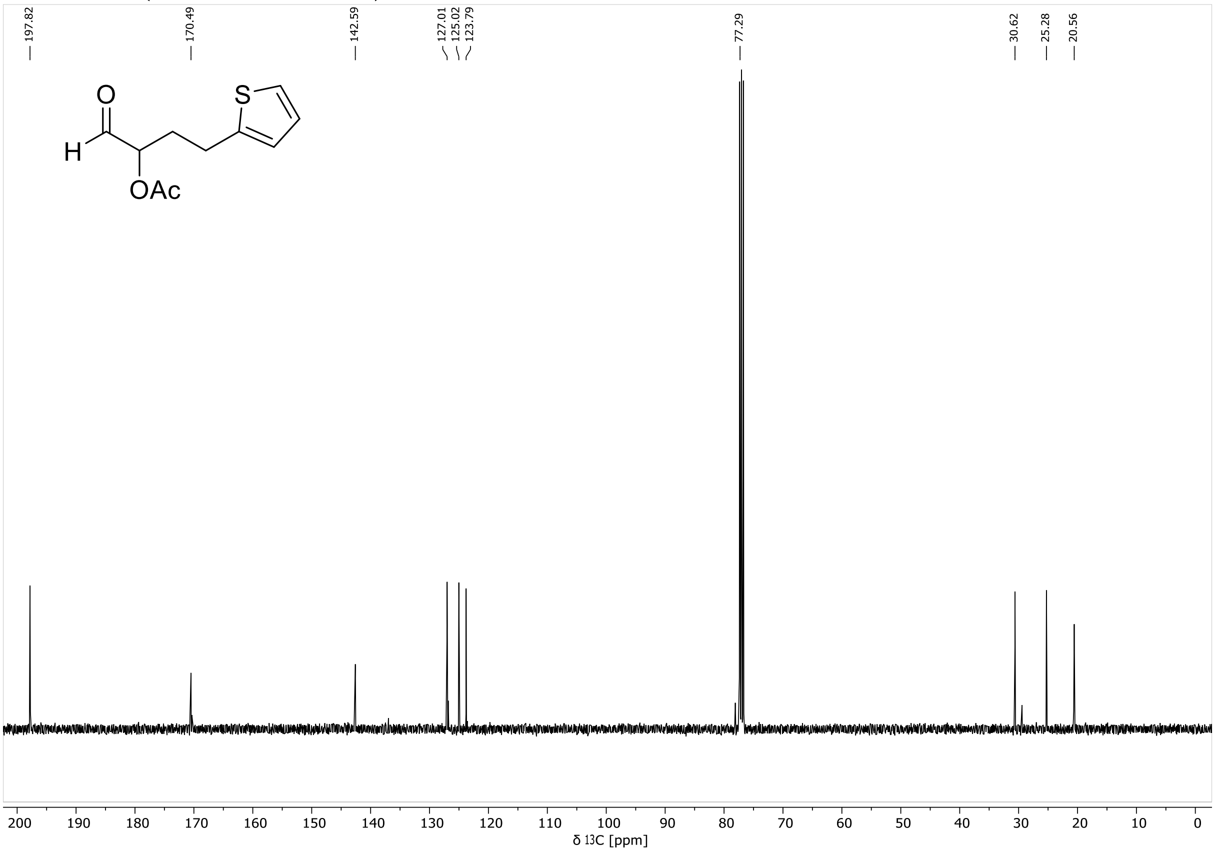

**$^1$H NMR** (400 MHz, $CDCl_3$):

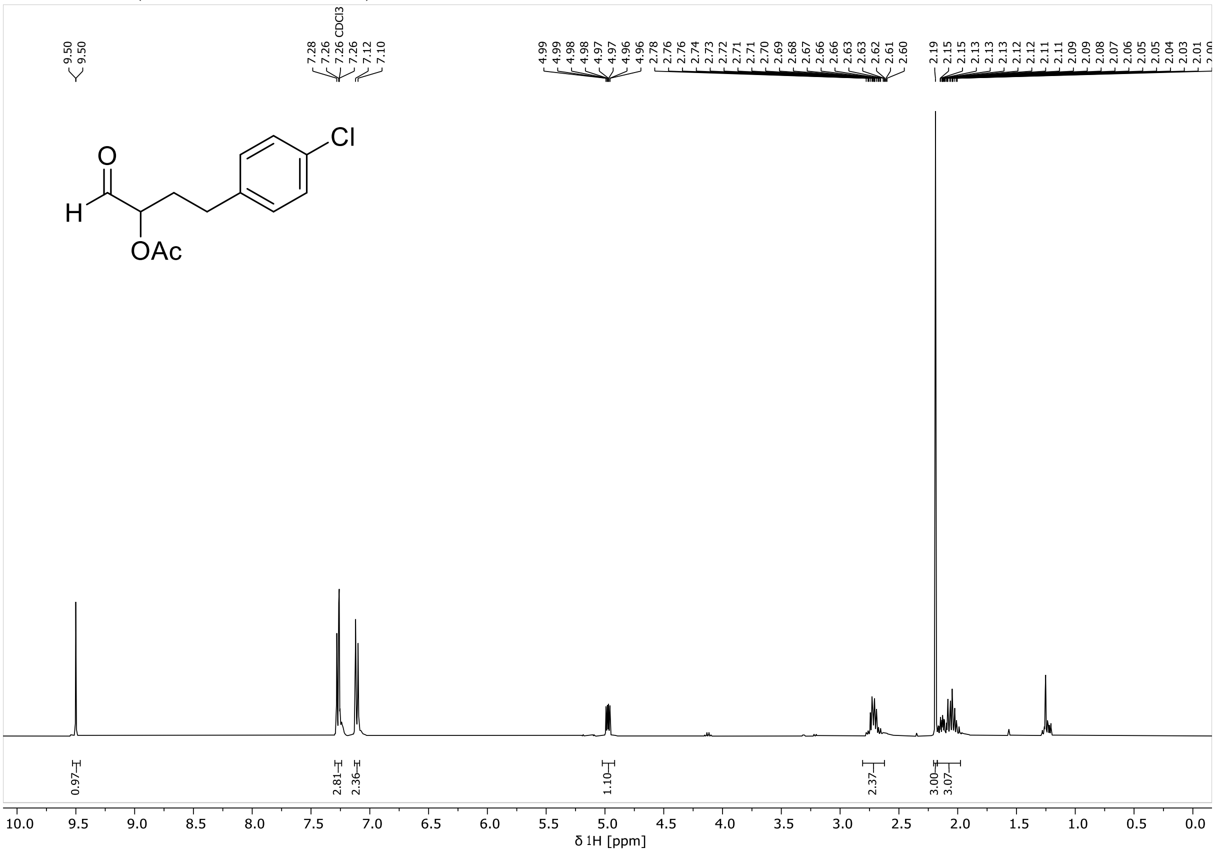

**$^{13}$C NMR** (101 MHz, $CDCl_3$):

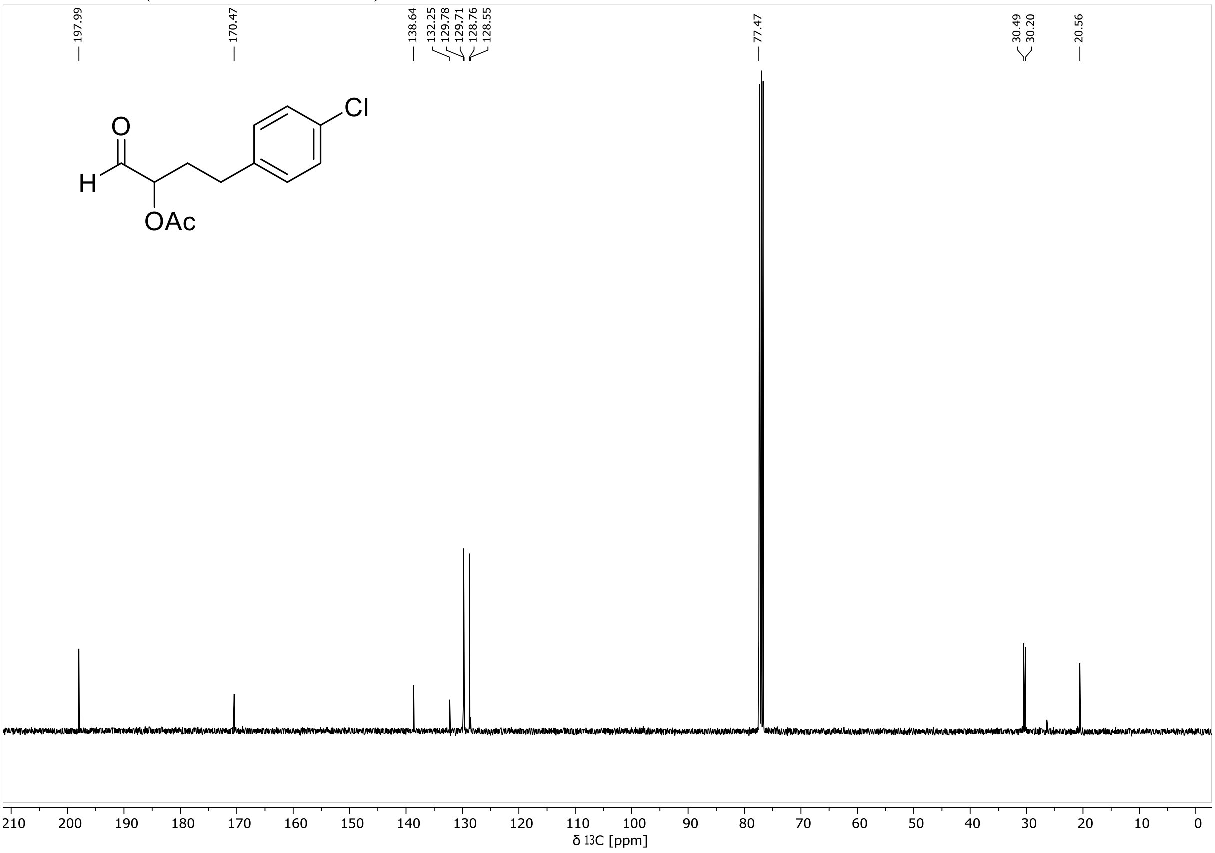

**$^{1}$H NMR** (400 MHz, $CDCl_3$):

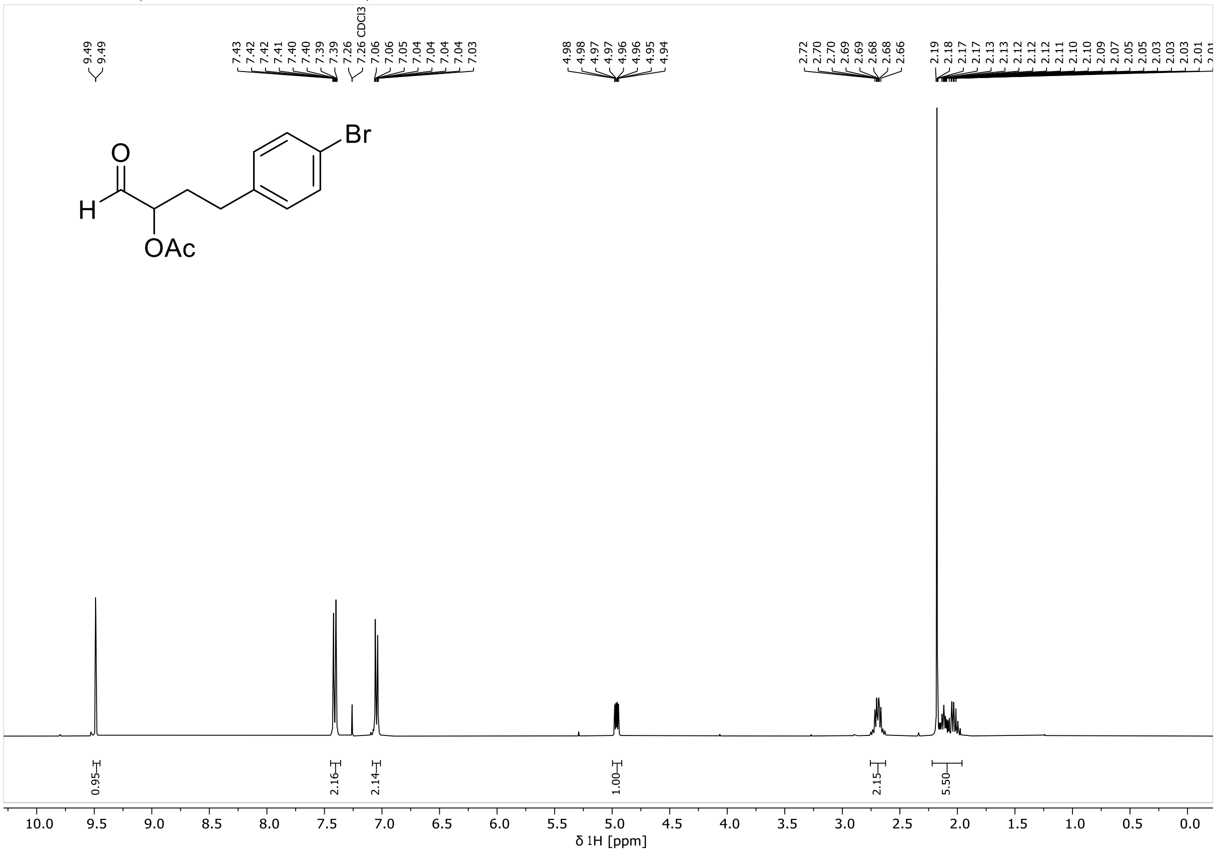

**$^{13}$C NMR** (101 MHz, $CDCl_3$):

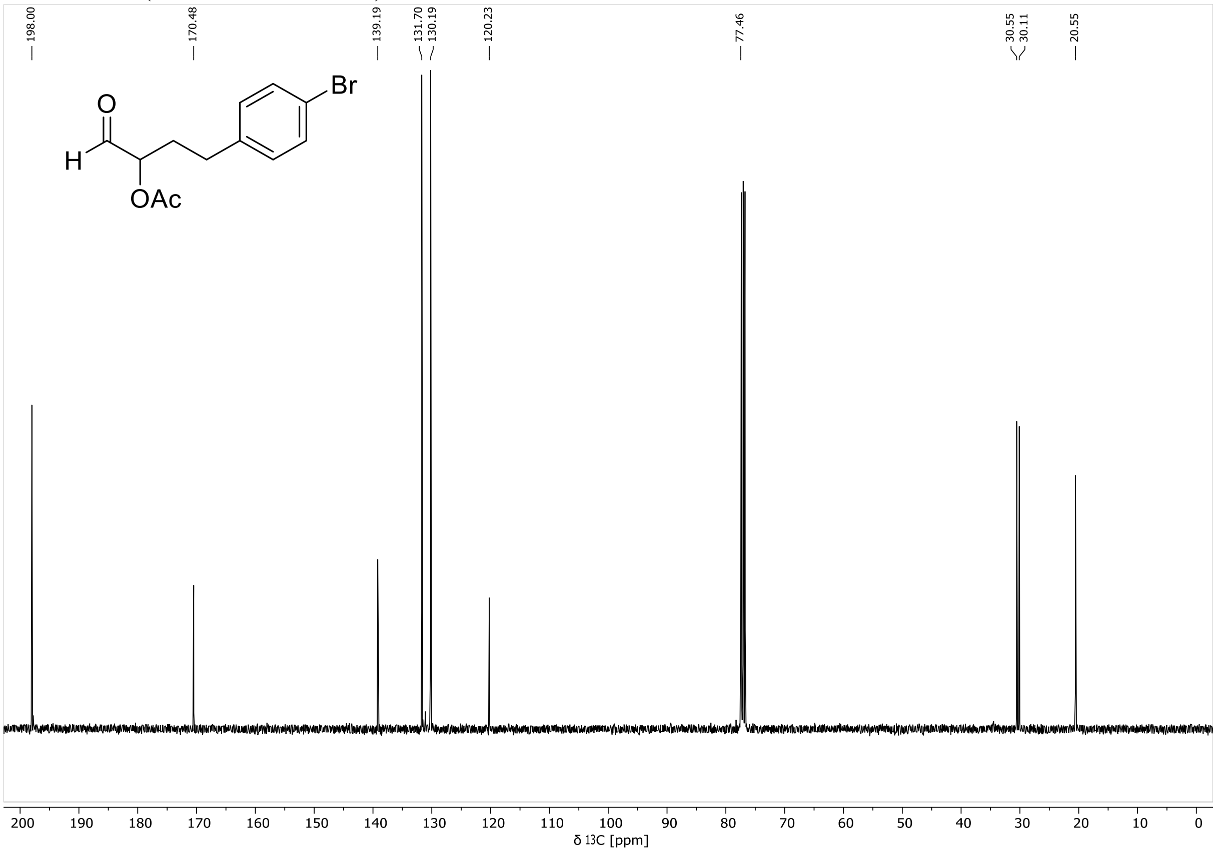

**$^{1}$H NMR** (400 MHz, $CDCl_3$):

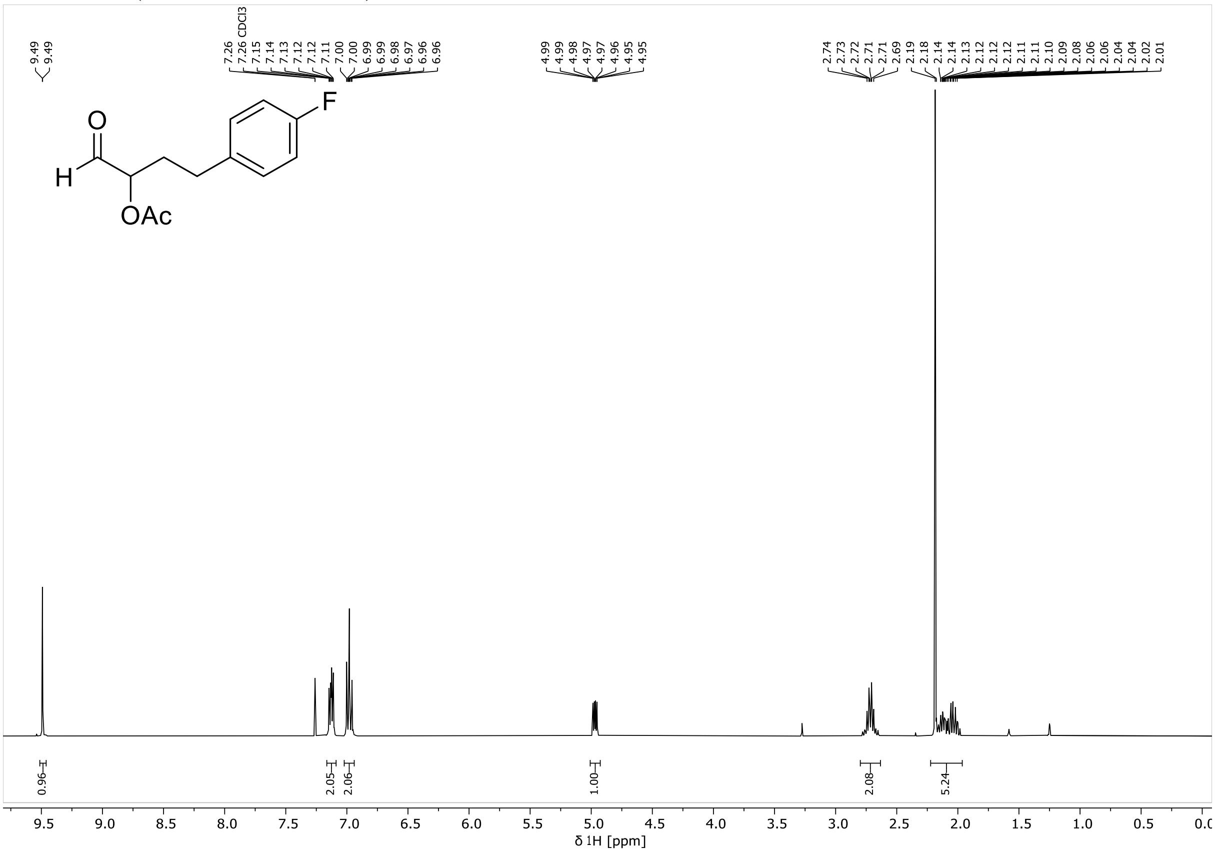


**$^{13}$C NMR** (101 MHz, $CDCl_3$):

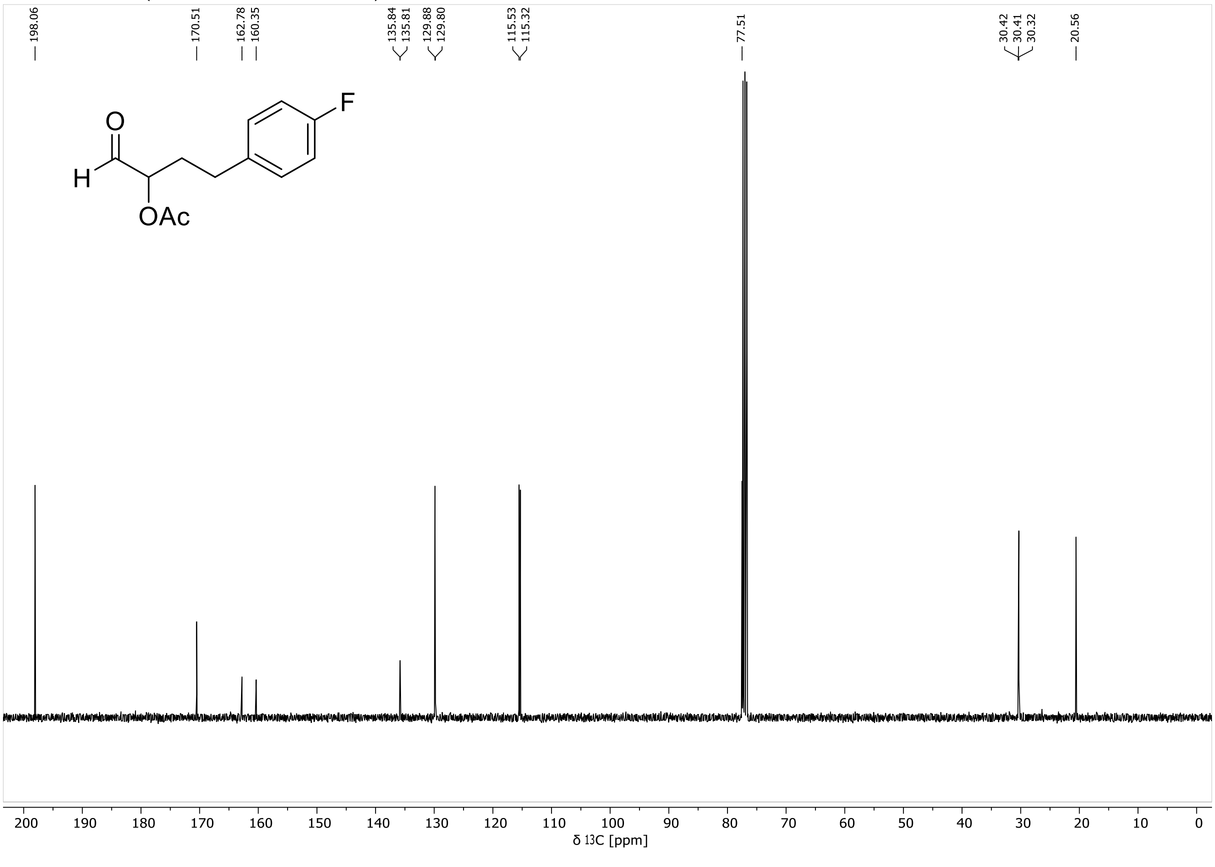

**$^{19}$F NMR** (377 MHz, $CDCl_3$):

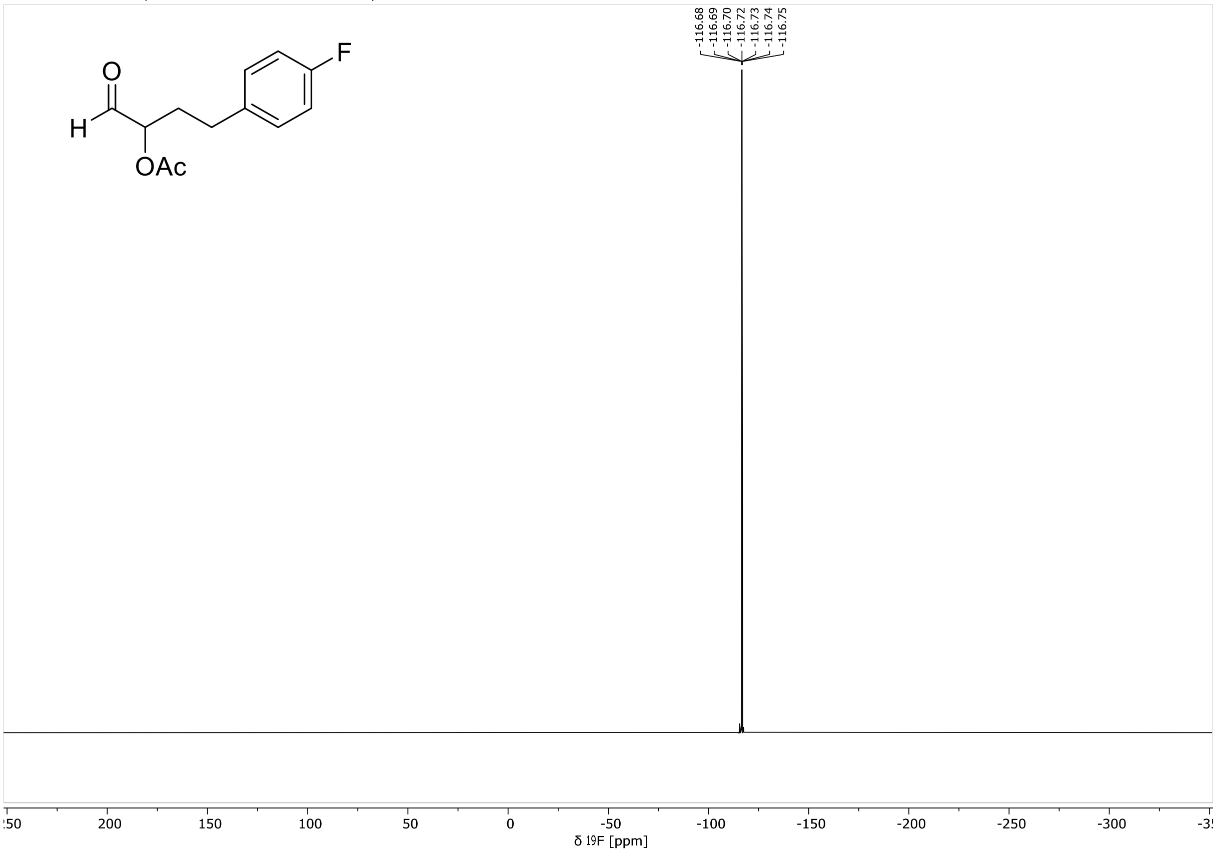

**$^{1}$H NMR** (400 MHz, $CDCl_3$):

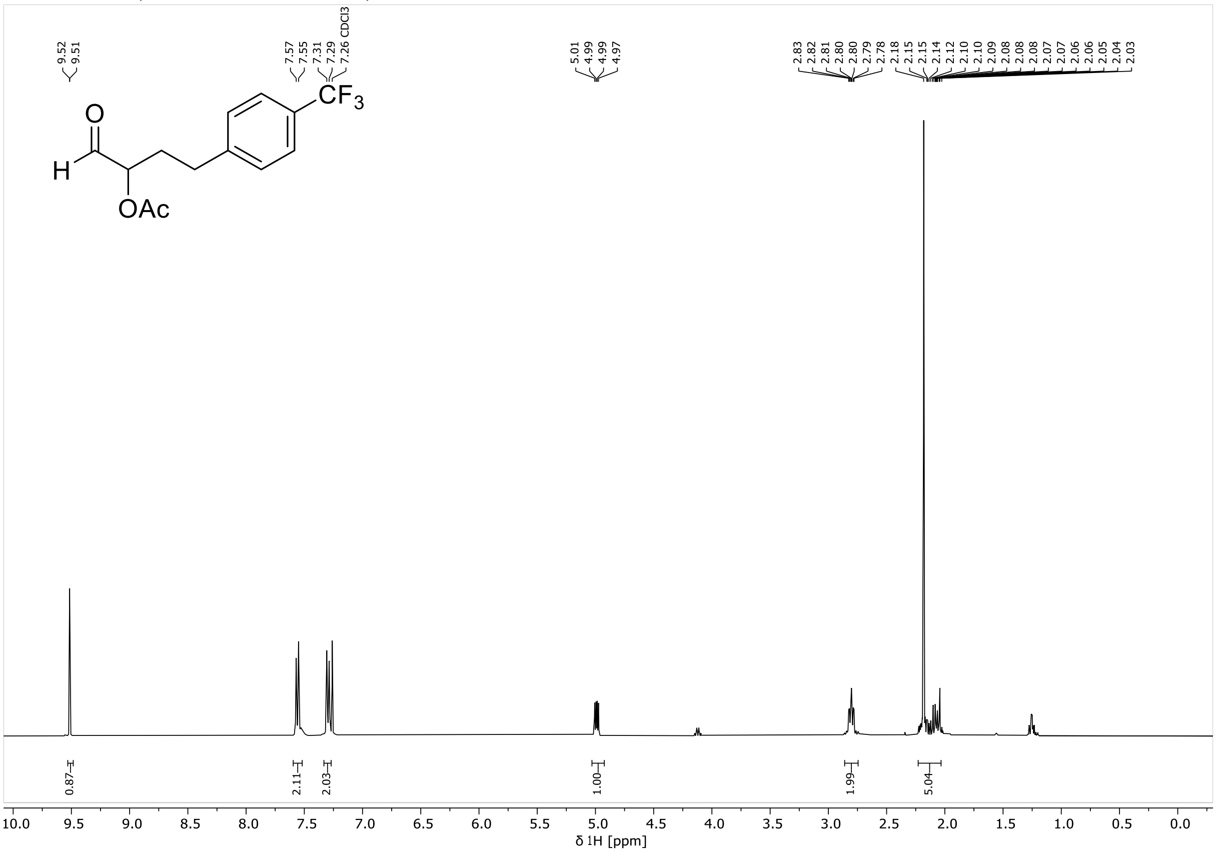

**$^{13}$C NMR** (101 MHz, $CDCl_3$):

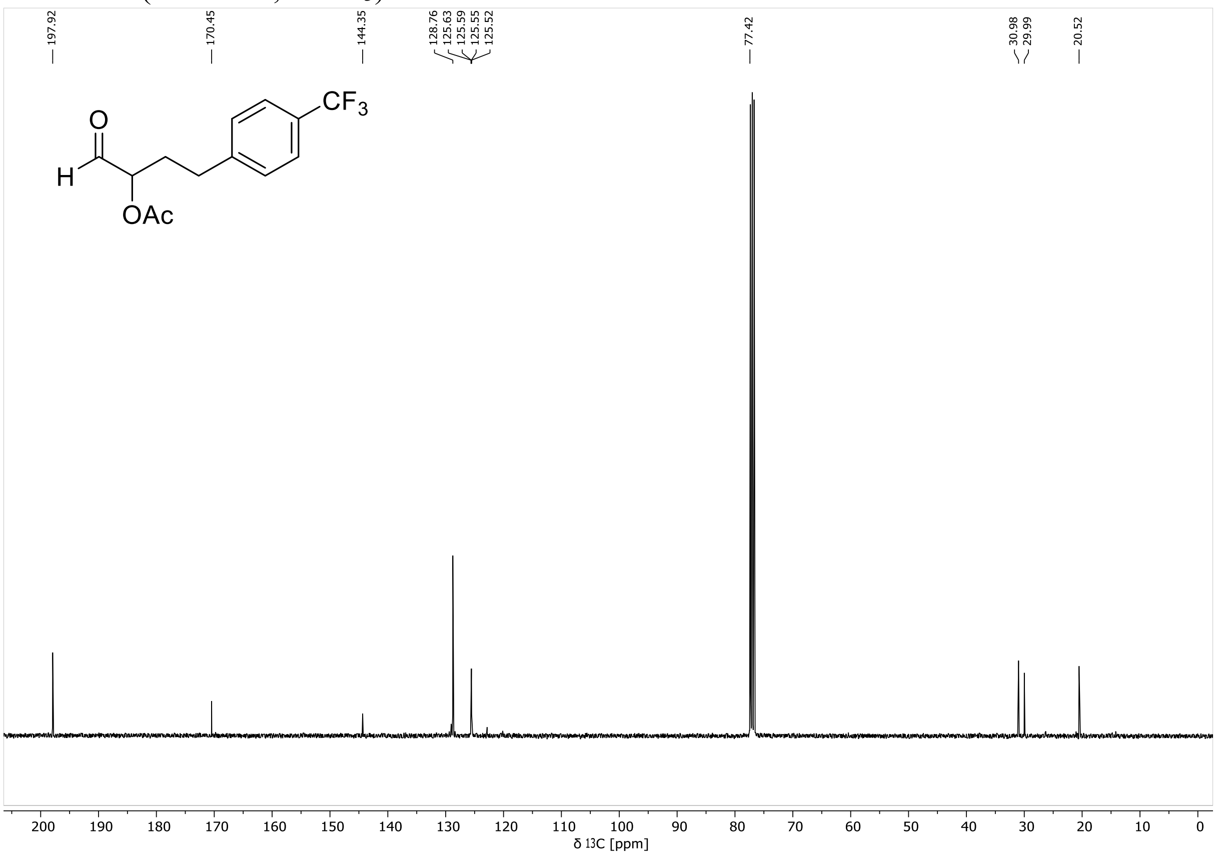

**$^{19}$F NMR** (377 MHz, $CDCl_3$):

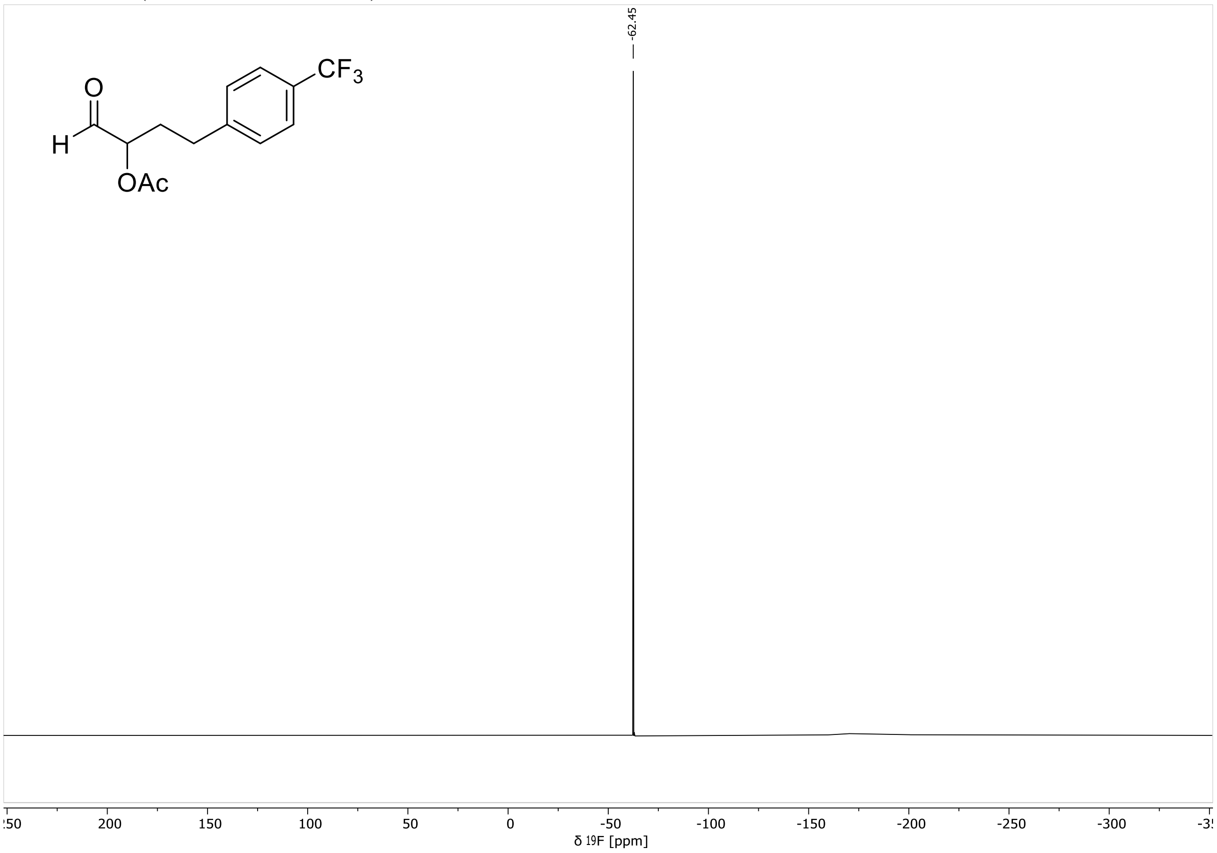

**$^{1}$H NMR** (400 MHz, $CDCl_3$):

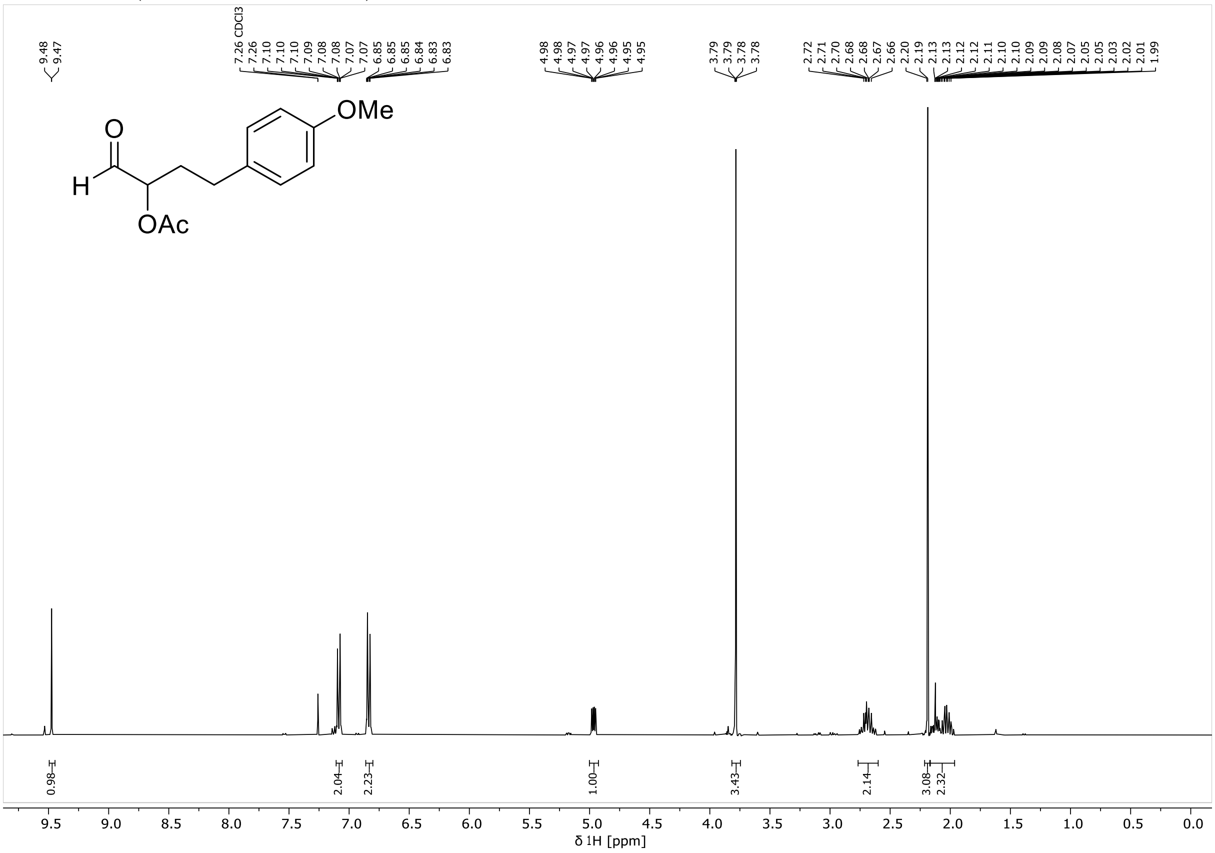


**$^{13}$C NMR** (101 MHz, $CDCl_3$):

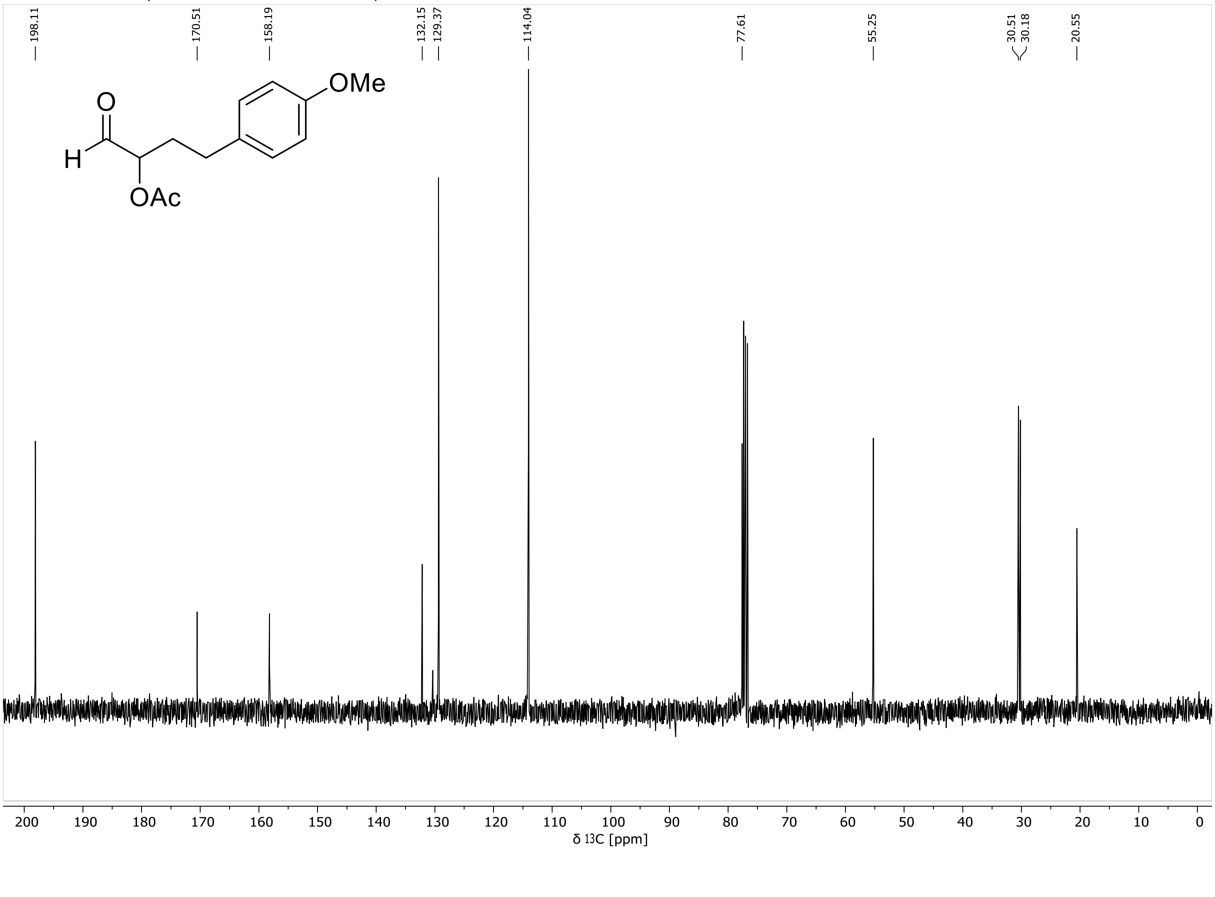

**$^{1}$H NMR** (400 MHz, $CDCl_3$):

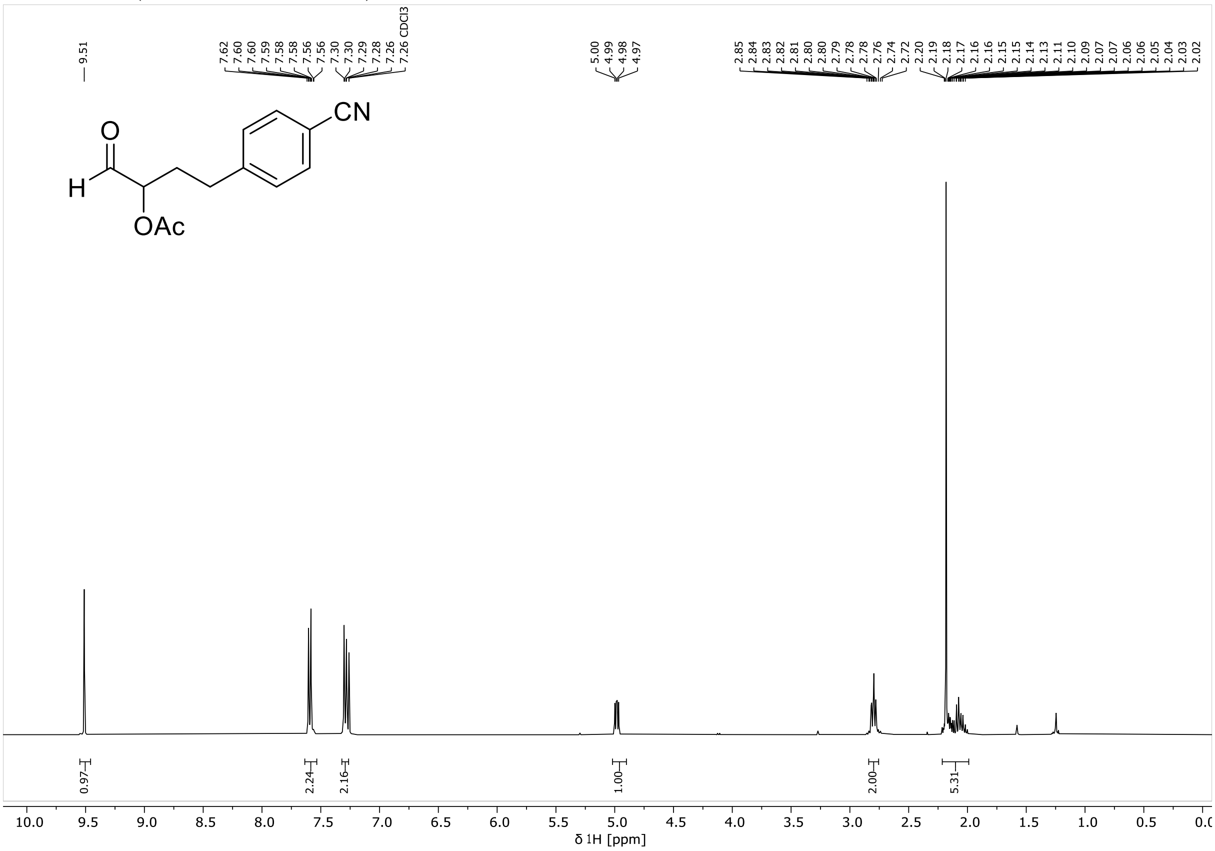


**$^{13}$C NMR** (101 MHz, $CDCl_3$):

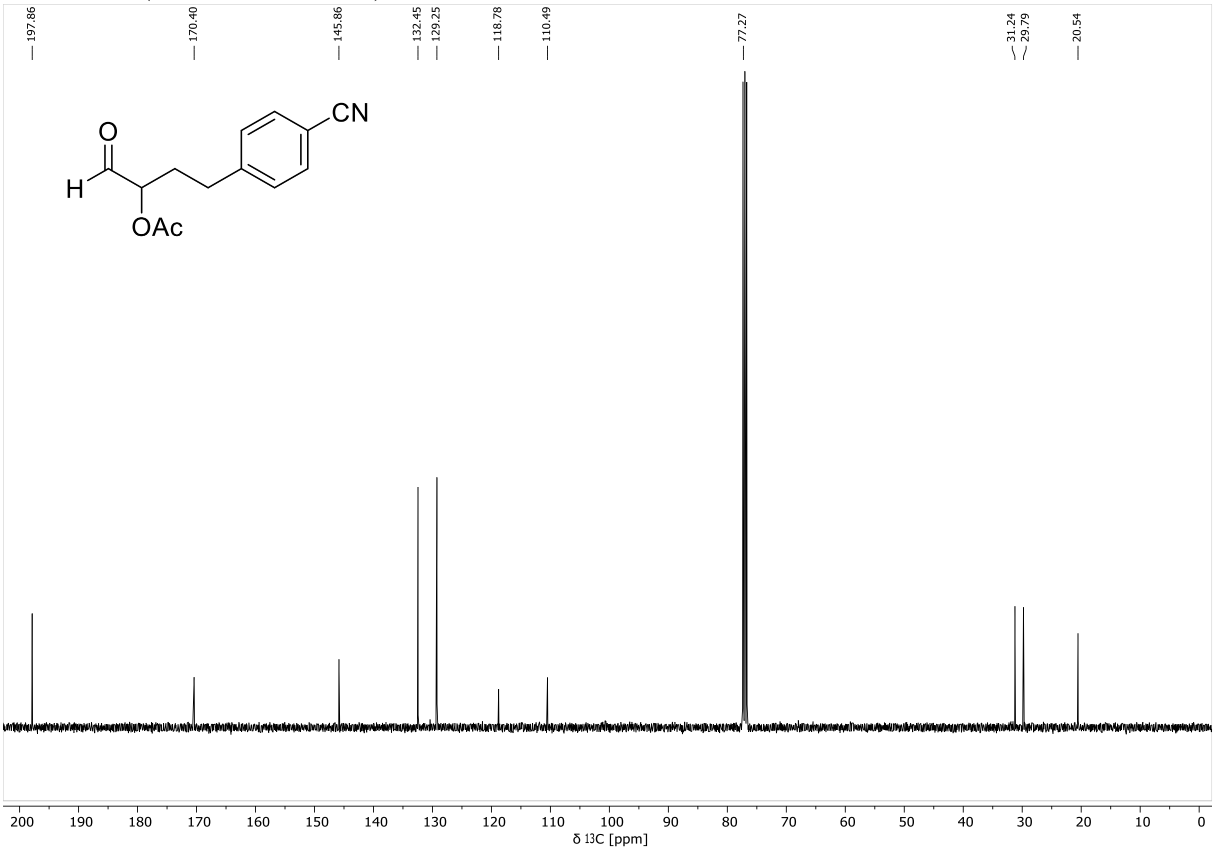

**Hydrazones:**

**$^{1}$H NMR** (300 MHz, $CDCl_3$) (**1a**):

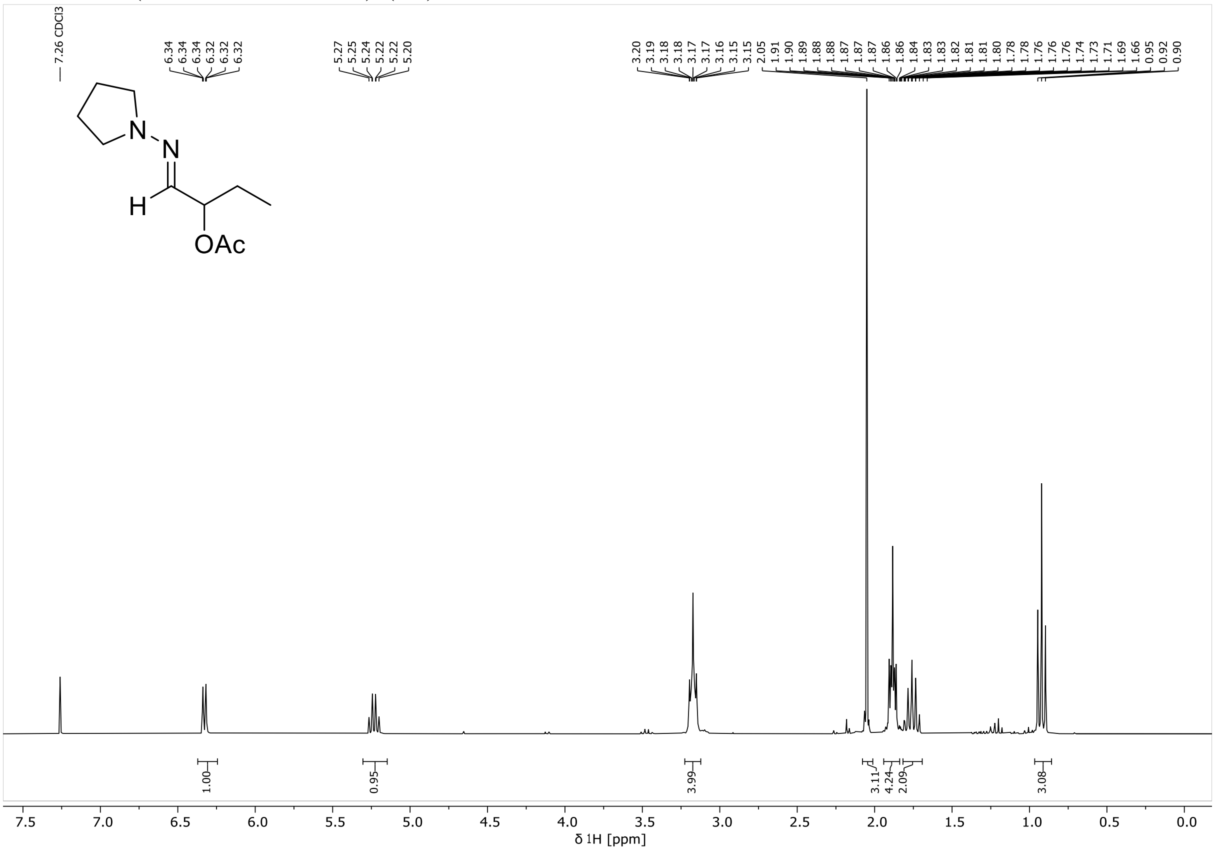


**$^{13}$C NMR** (75 MHz, $CDCl_3$) (**1a**):

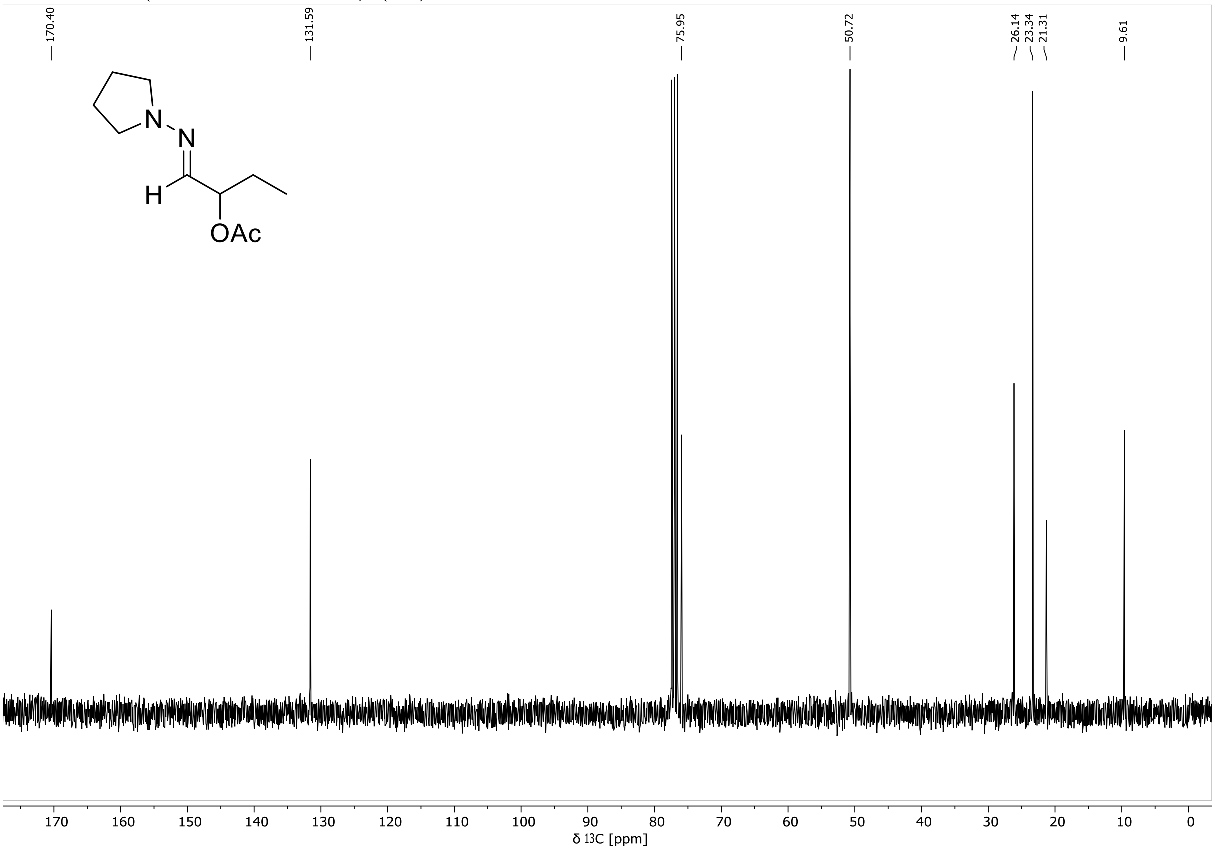

**$^{1}$H NMR** (400 MHz, $CDCl_3$) (**1b**):

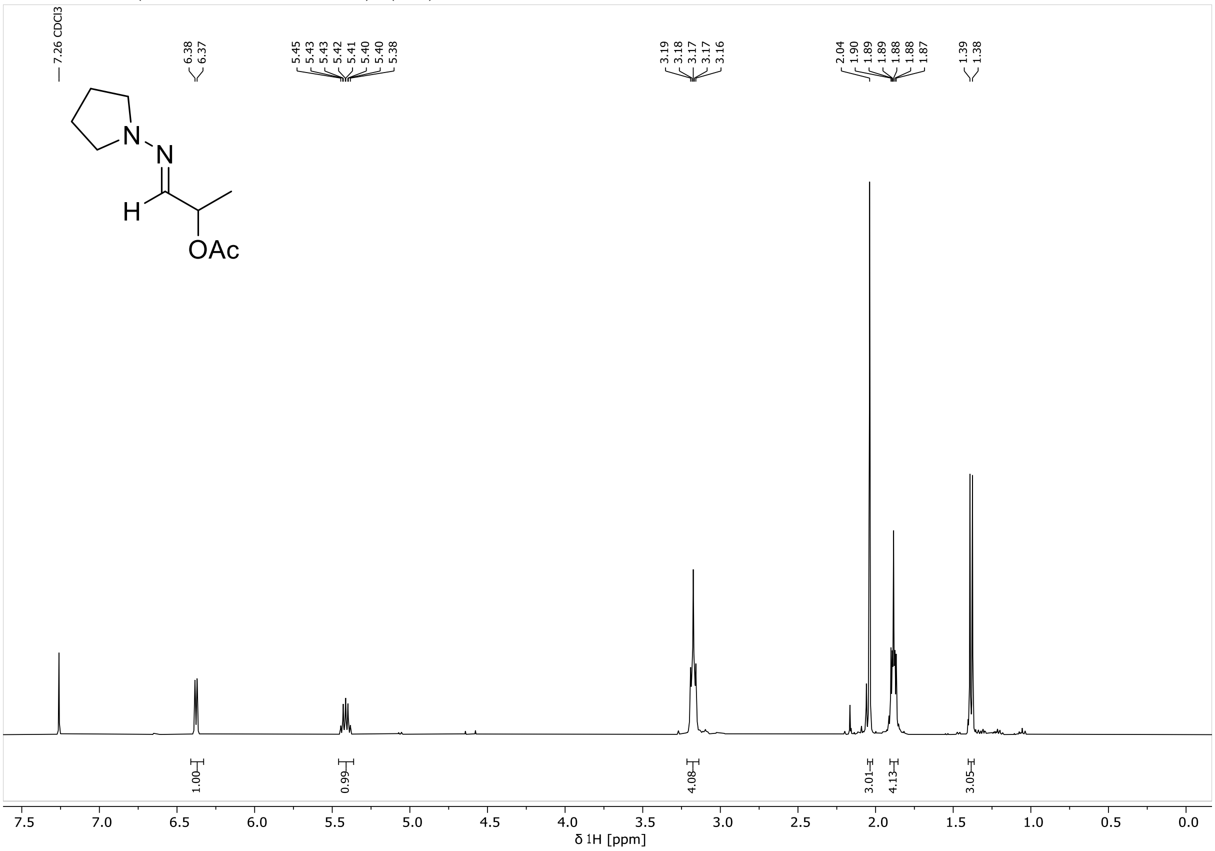


**$^{13}$C NMR** (101 MHz, $CDCl_3$) (**1b**):

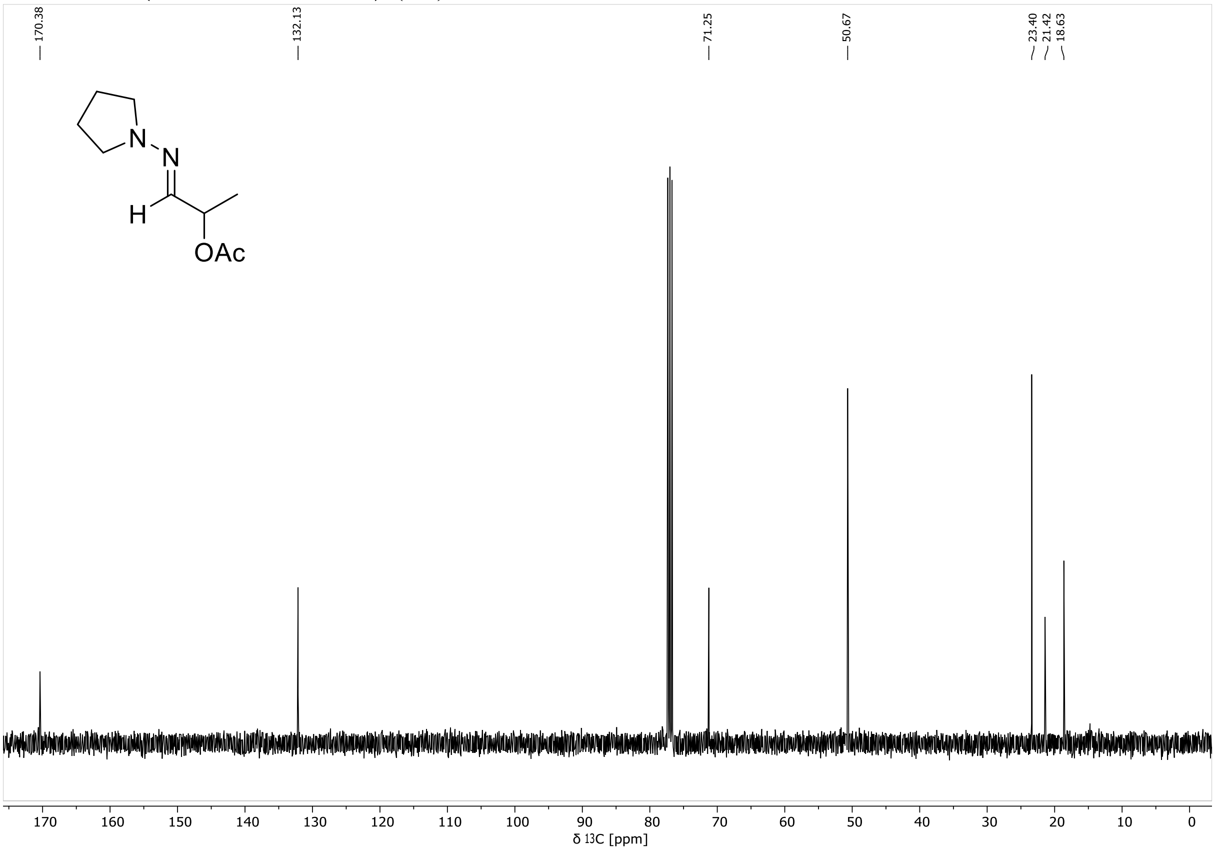

**$^1$H NMR** (400 MHz, $CDCl_3$) (**1c**):

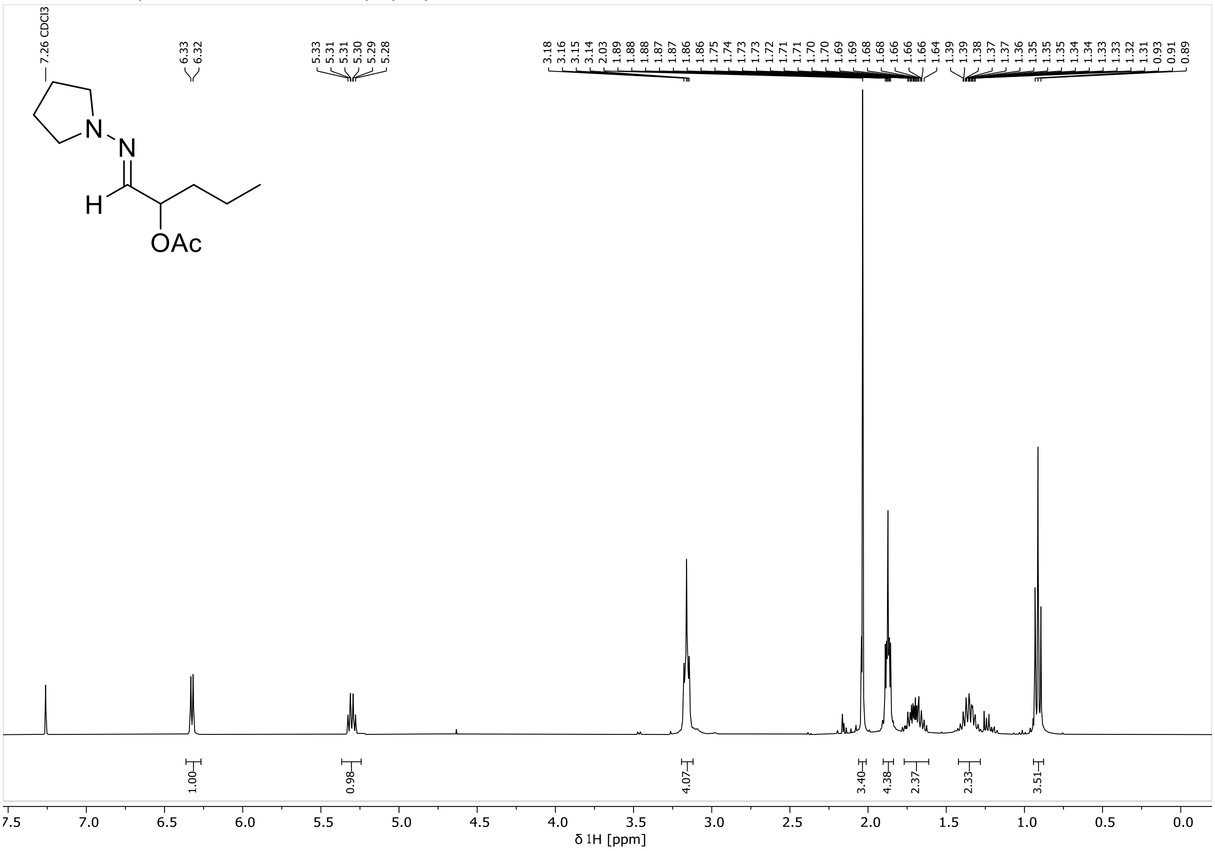


**$^{13}$C NMR** (101 MHz, $CDCl_3$) (**1c**):

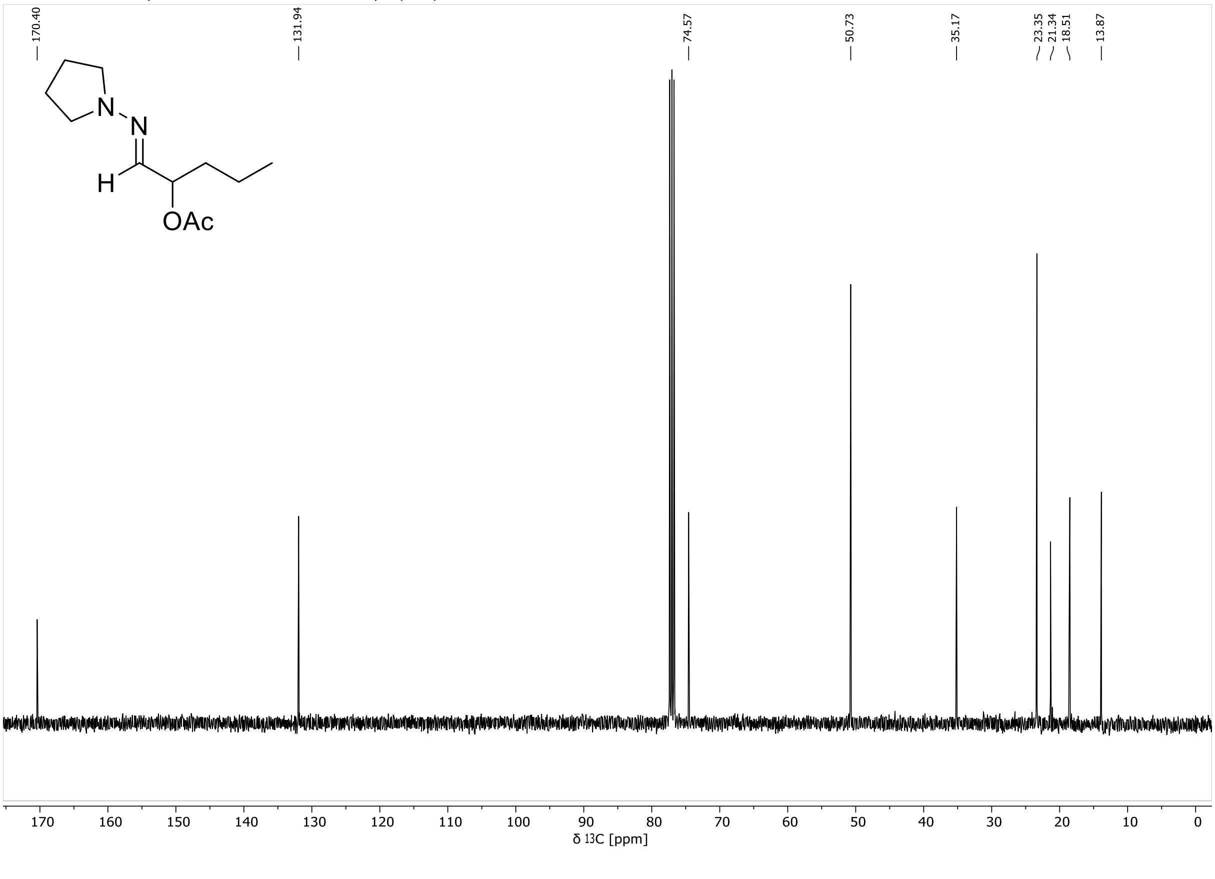

**$^{1}$H NMR** (400 MHz, $CDCl_3$) (**1d**):

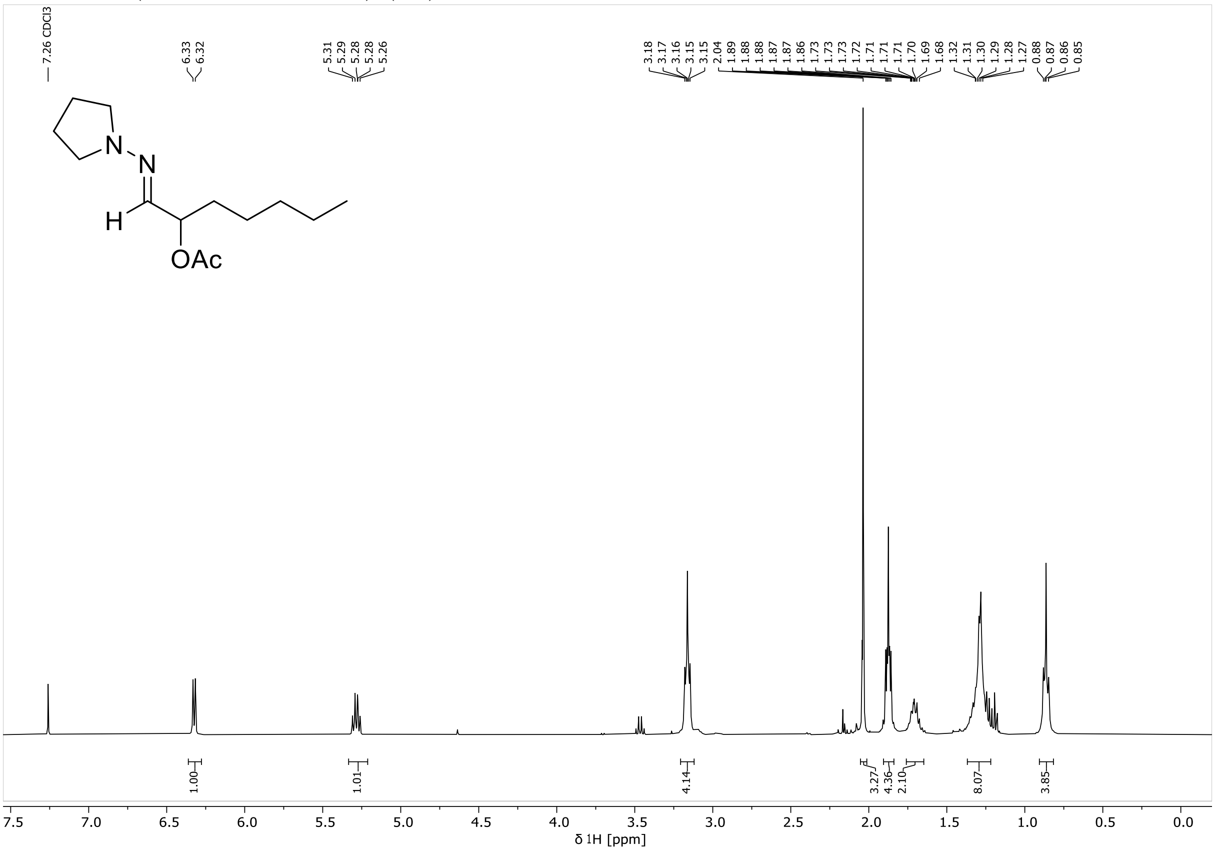


**$^{13}$C NMR** (101 MHz, $CDCl_3$) (**1d**):

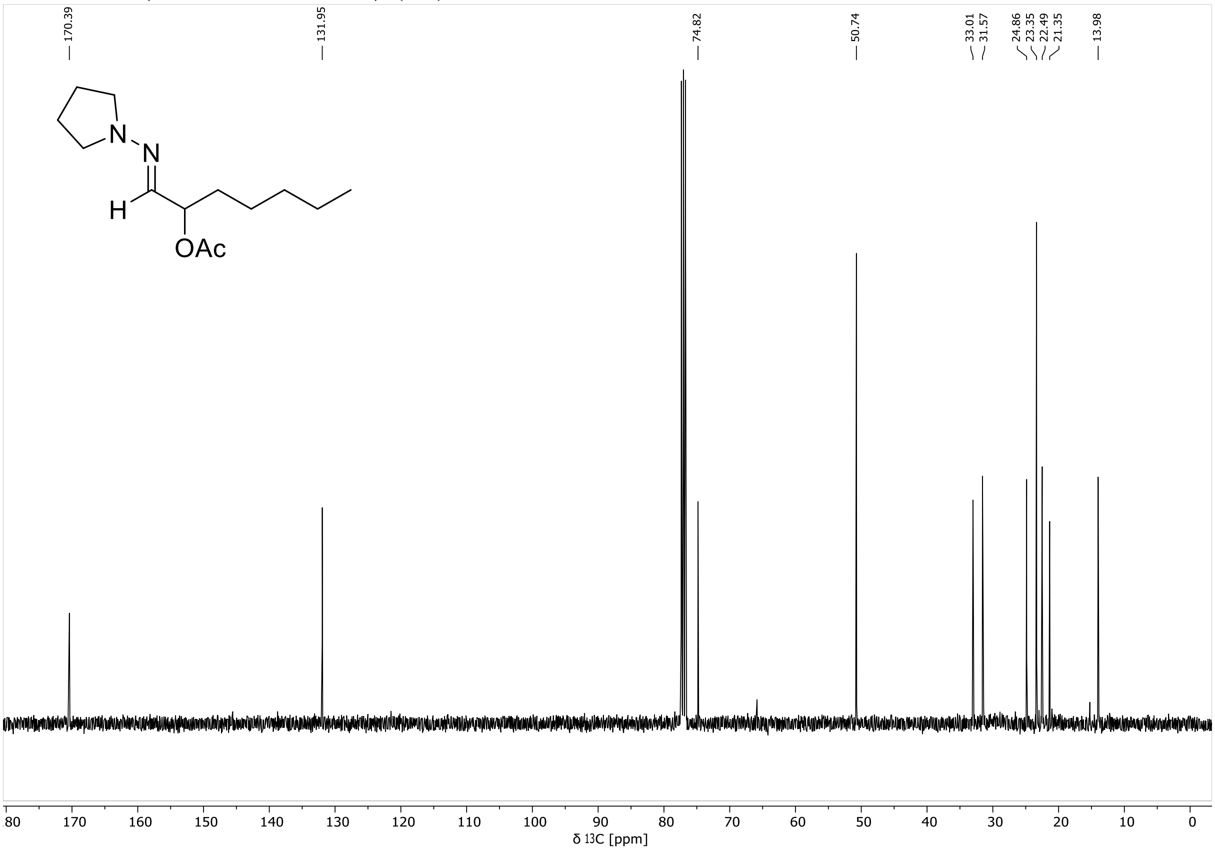

**$^{1}H$ NMR** (400 MHz, $CDCl_3$) (**1e**):

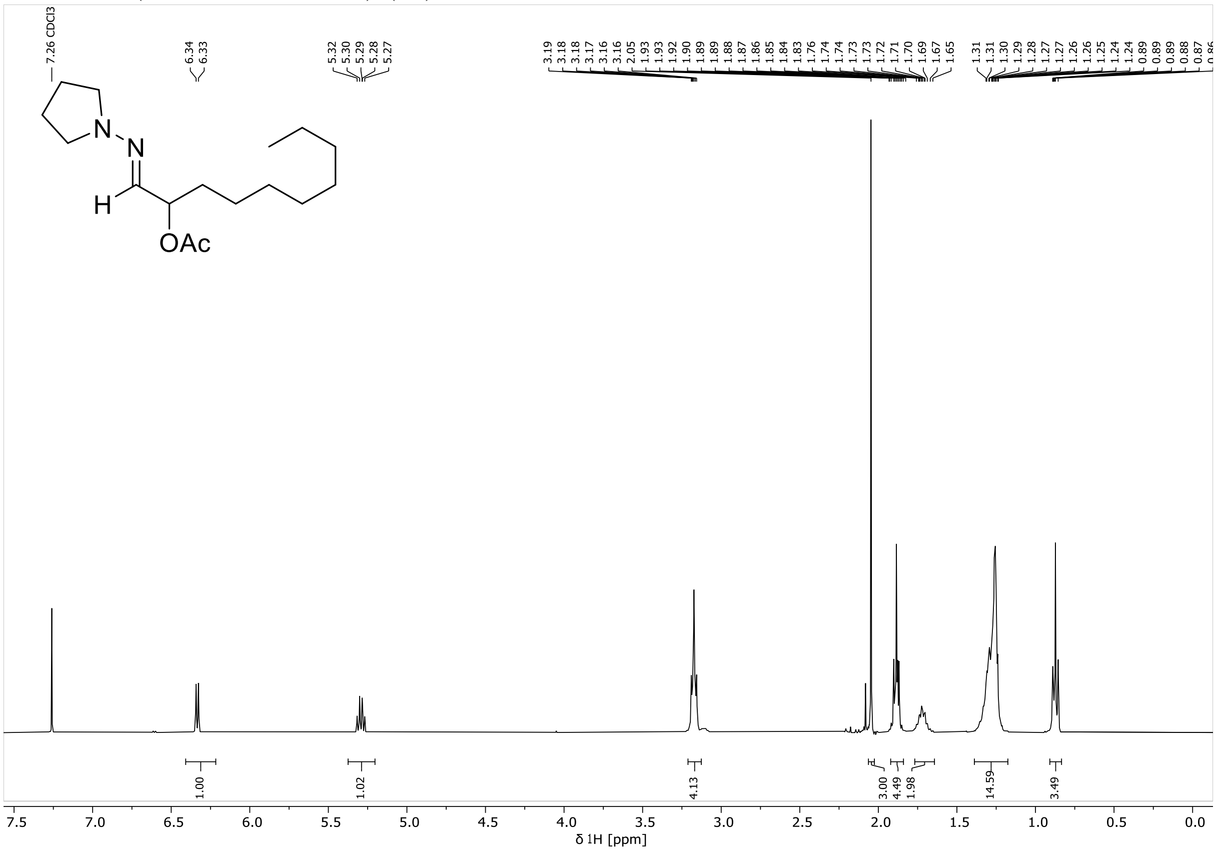


**$^{13}C$ NMR** (101 MHz, $CDCl_3$) (**1e**):

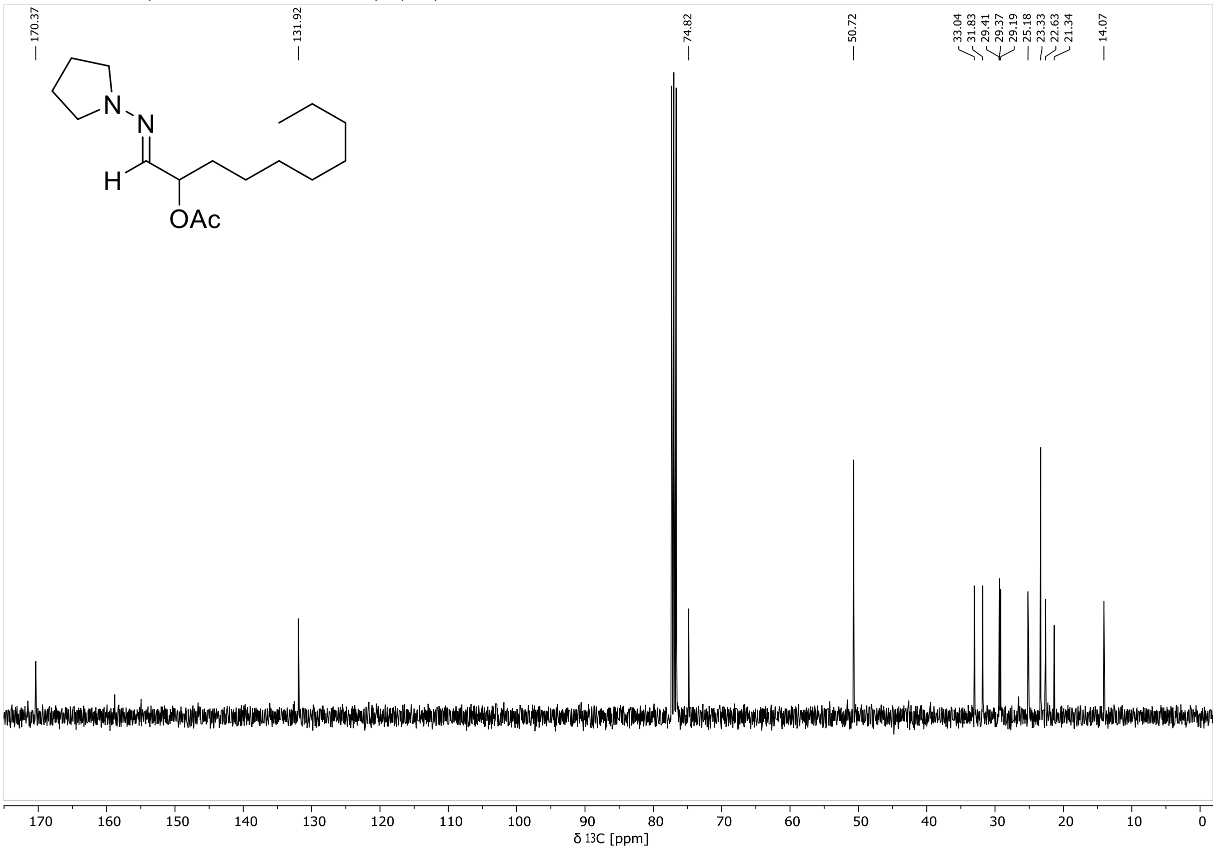

**$^{1}$H NMR** (400 MHz, $CDCl_3$) (**1f**):

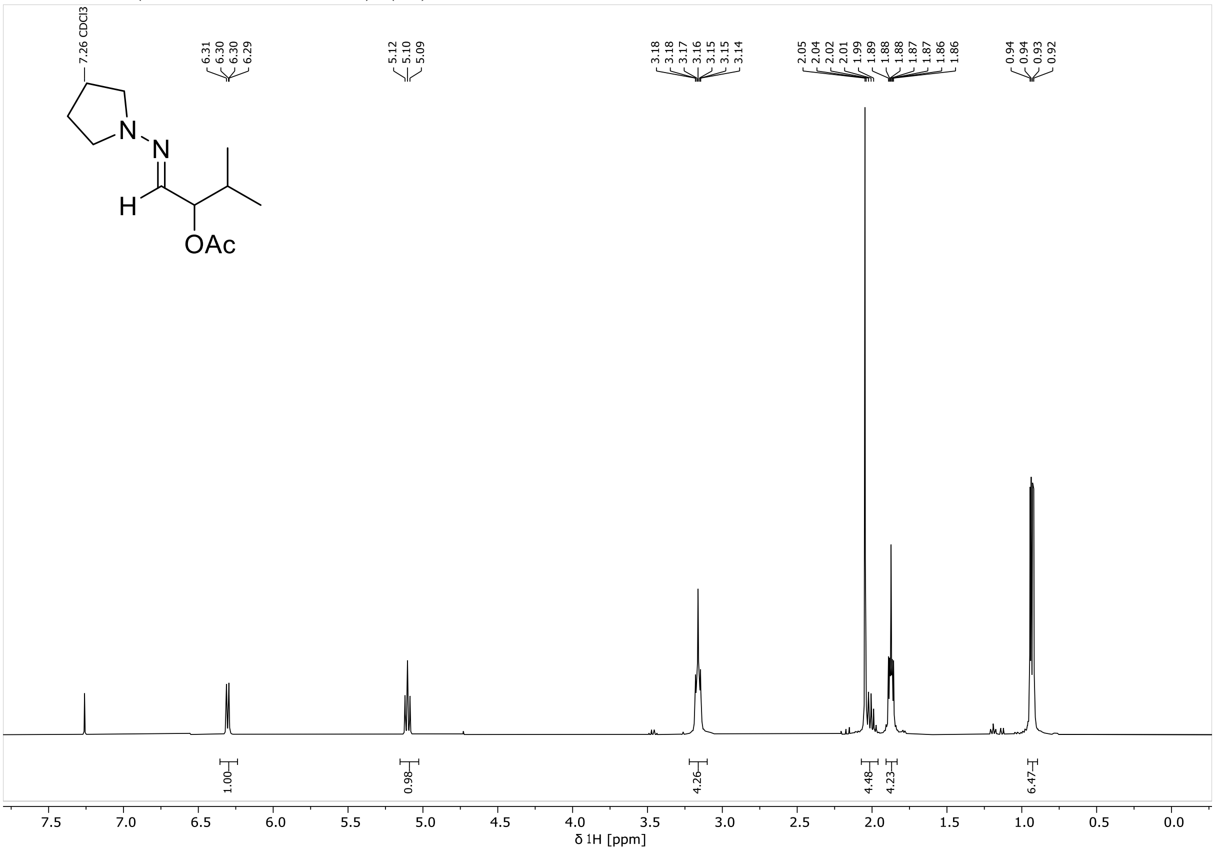

**$^{13}$C NMR** (101 MHz, $CDCl_3$) (**1f**):

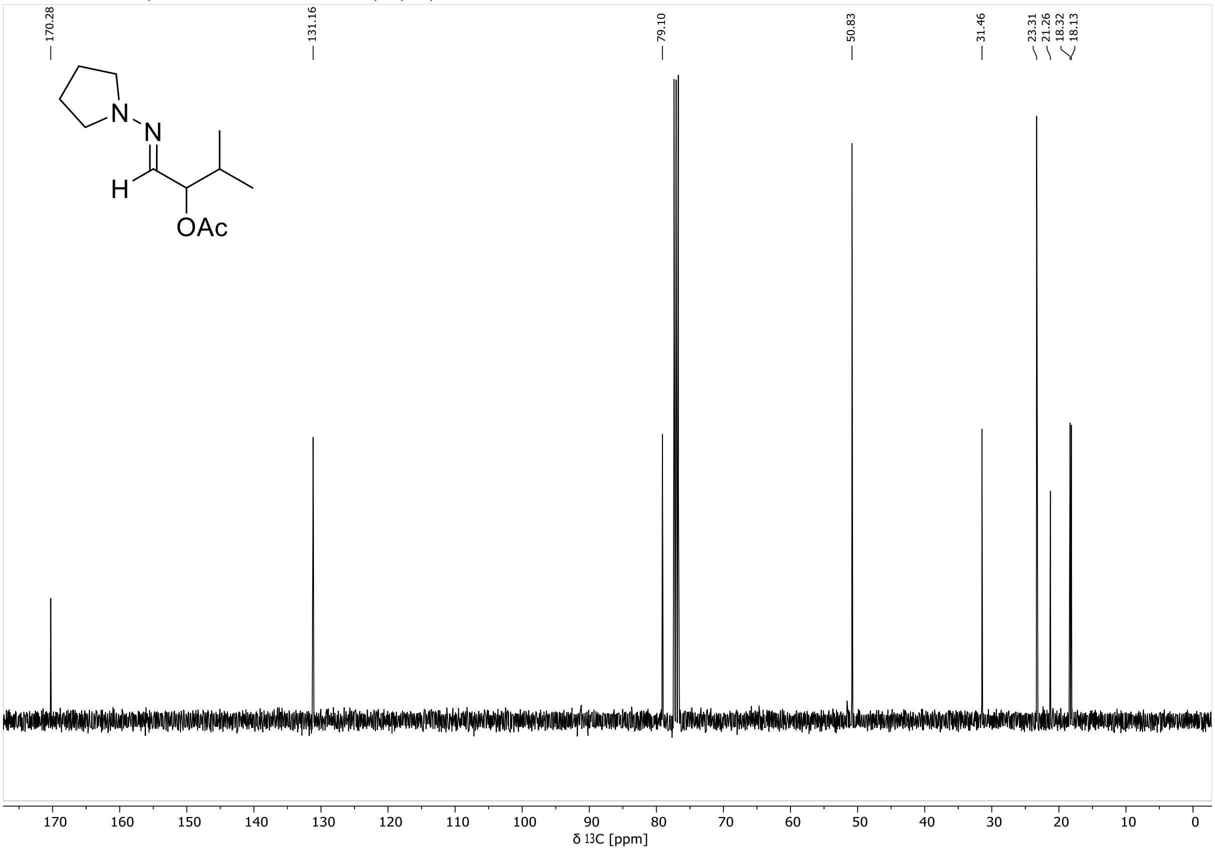

**$^{1}$H NMR** (400 MHz, $CDCl_3$) (**1g**):

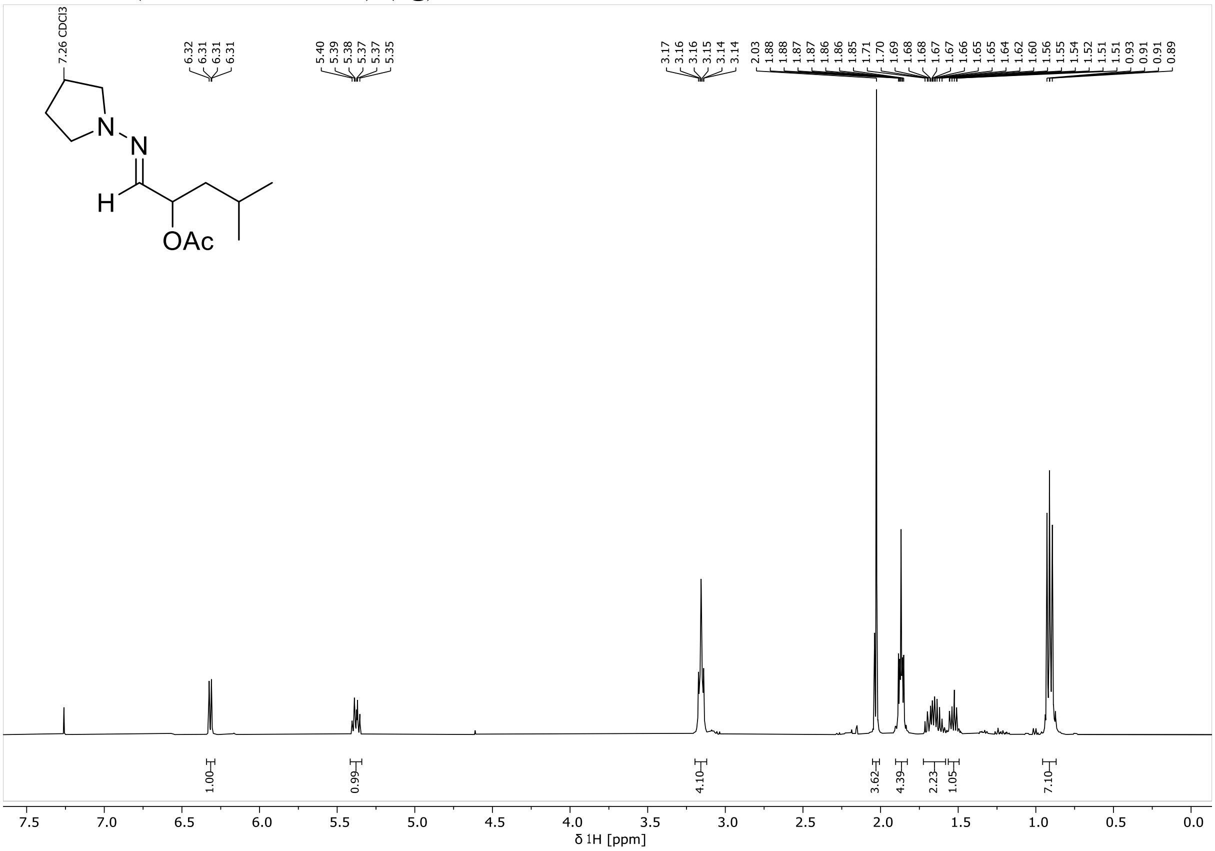

**$^{13}$C NMR** (101 MHz, $CDCl_3$) (**1g**):

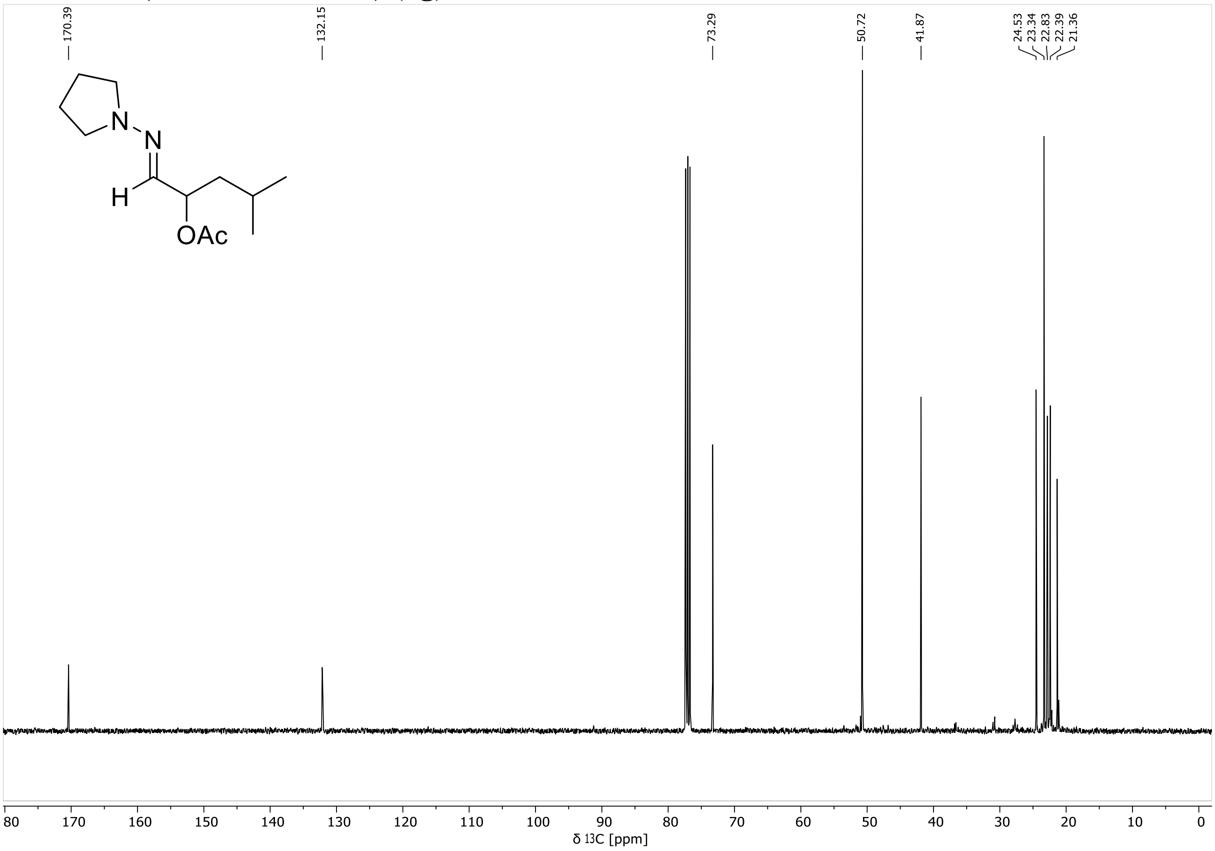

**$^{1}H$ NMR** (400 MHz, $CDCl_3$) (**1h**):

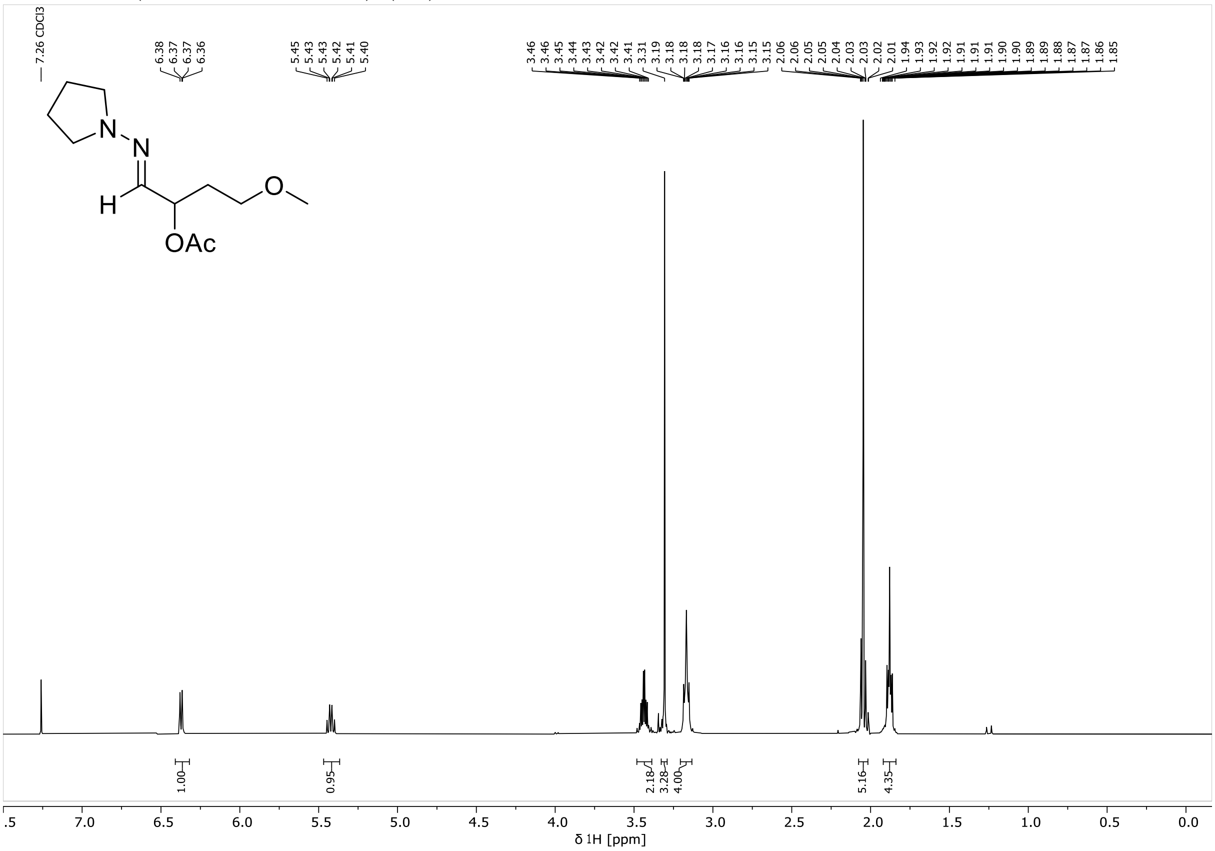


**$^{13}C$ NMR** (101 MHz, $CDCl_3$) (**1h**):

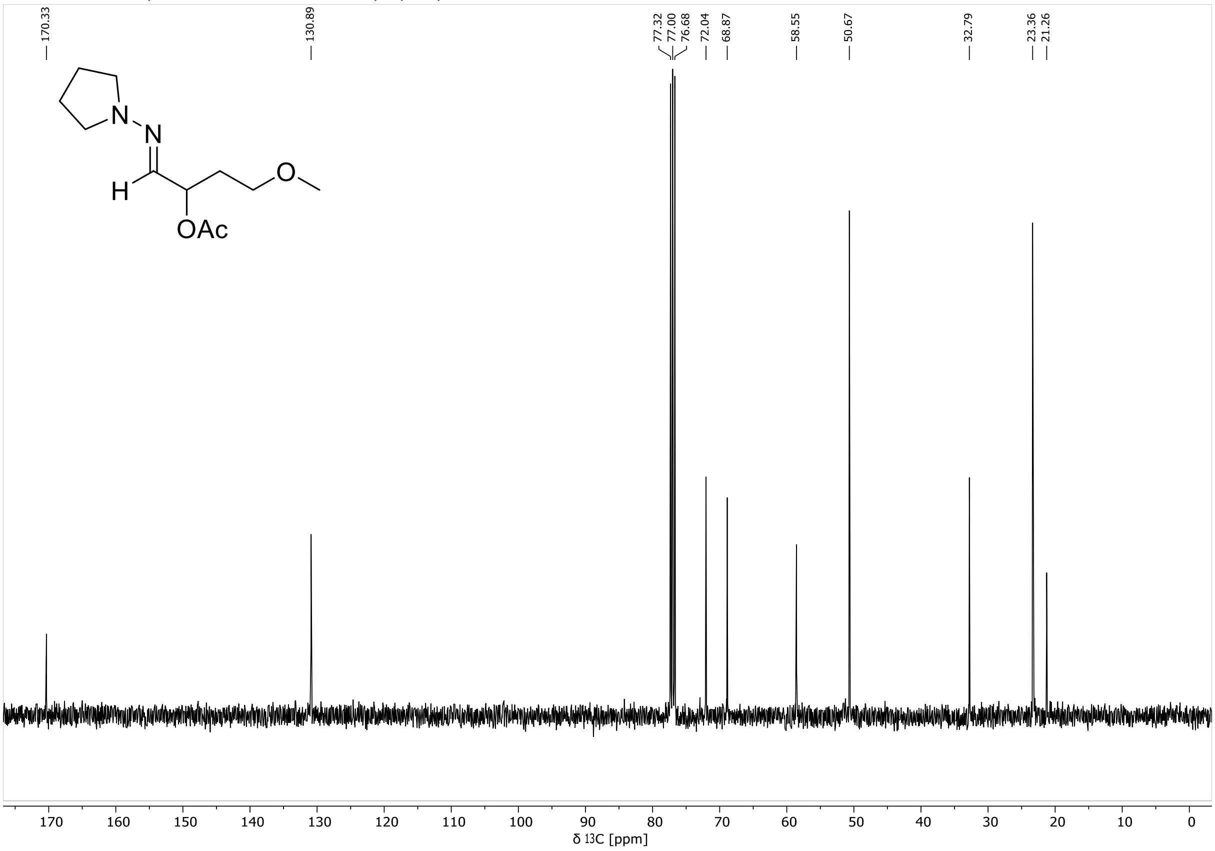

**$^{1}$H NMR** (400 MHz, CDCl$_3$) (**1i**):

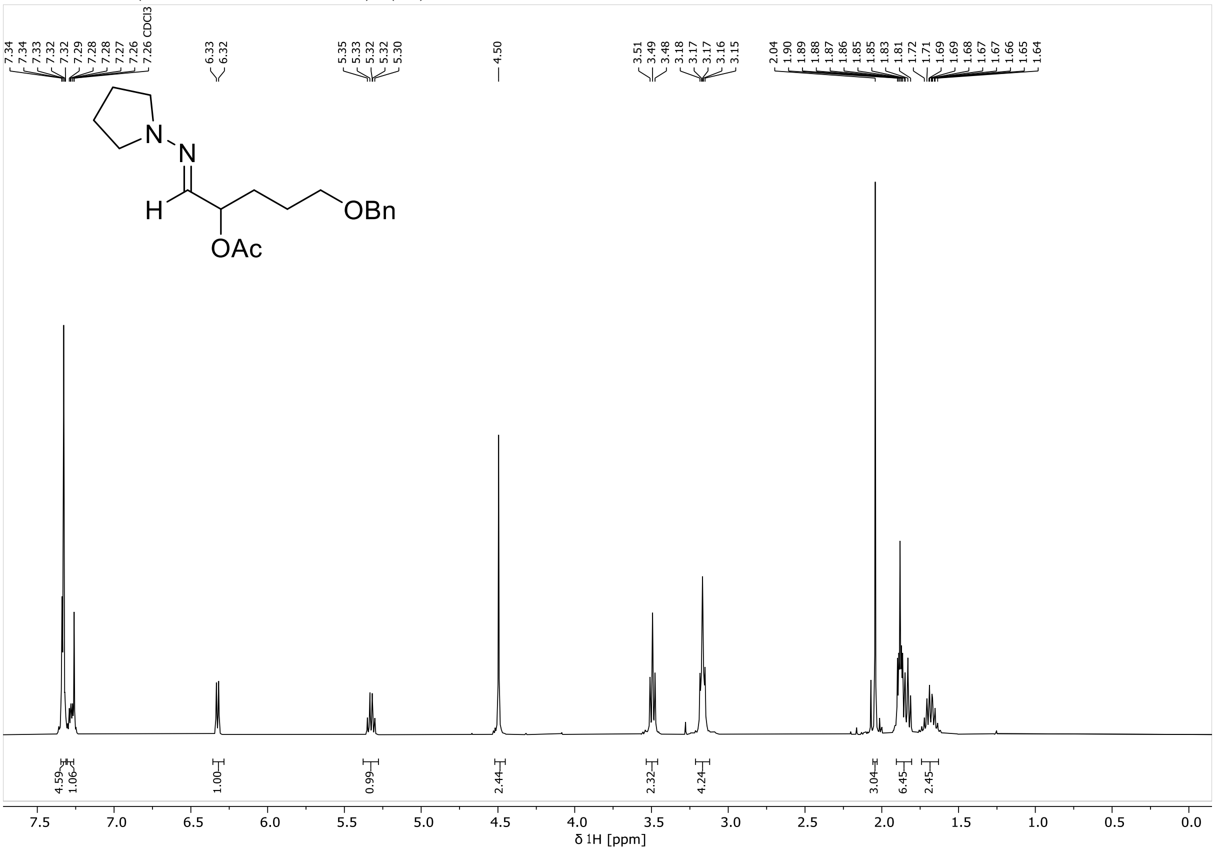

**$^{13}$C NMR** (101 MHz, CDCl$_3$) (**1i**):

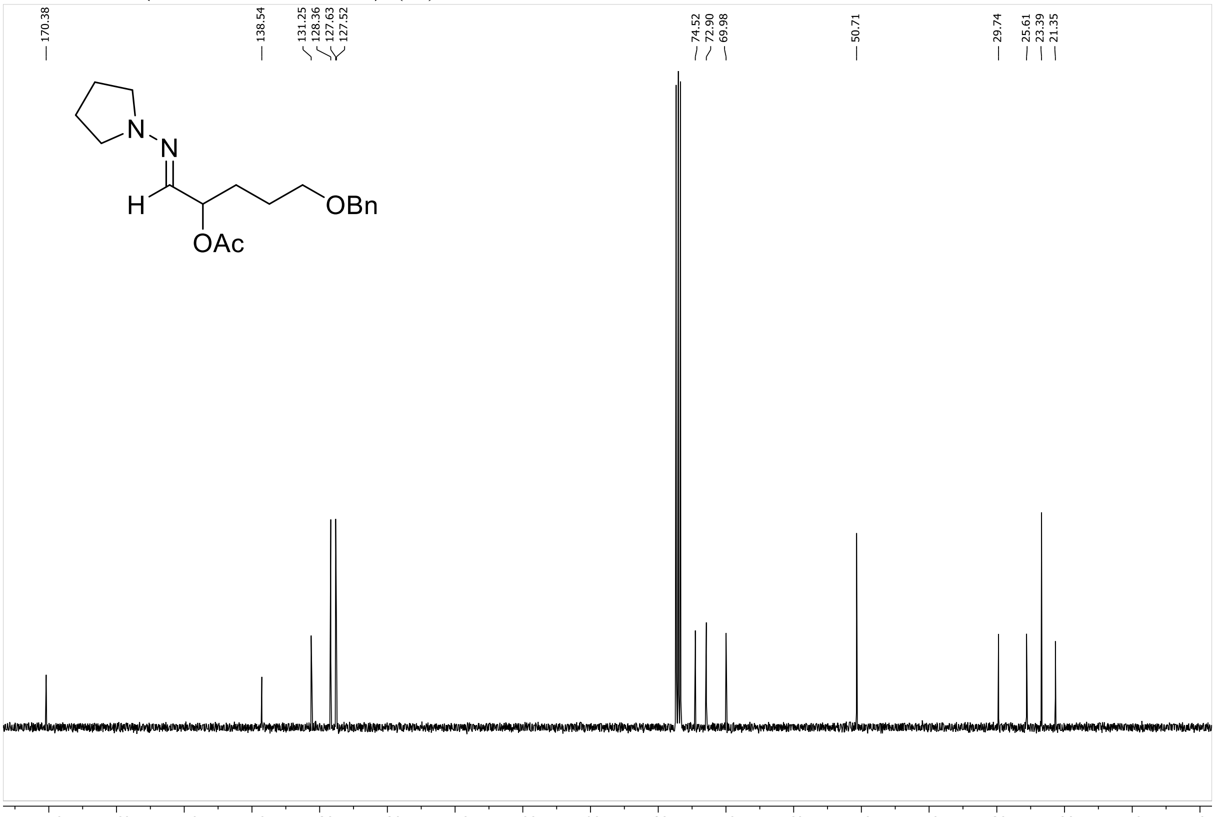

**$^{1}$H NMR** (400 MHz, $CDCl_3$) (**1j**):

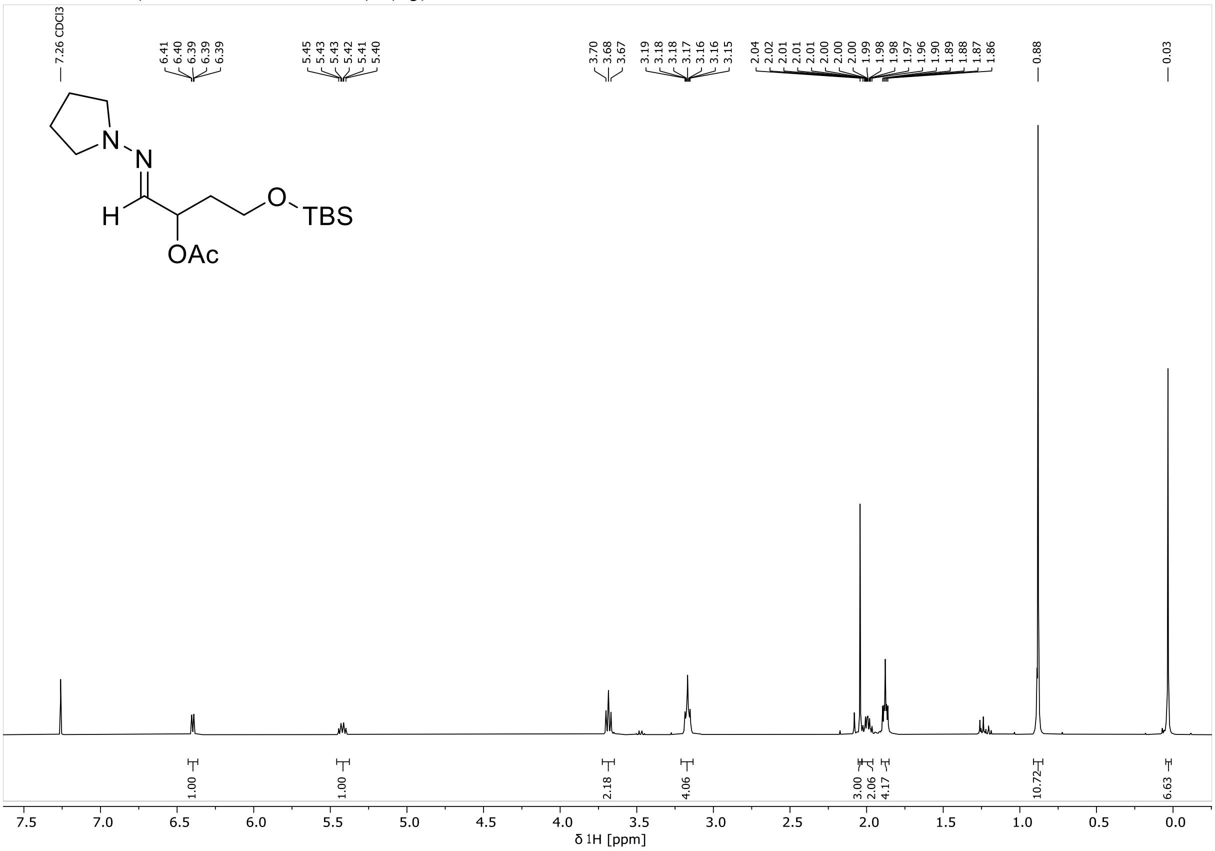

**$^{13}$C NMR** (101 MHz, $CDCl_3$) (**1j**):

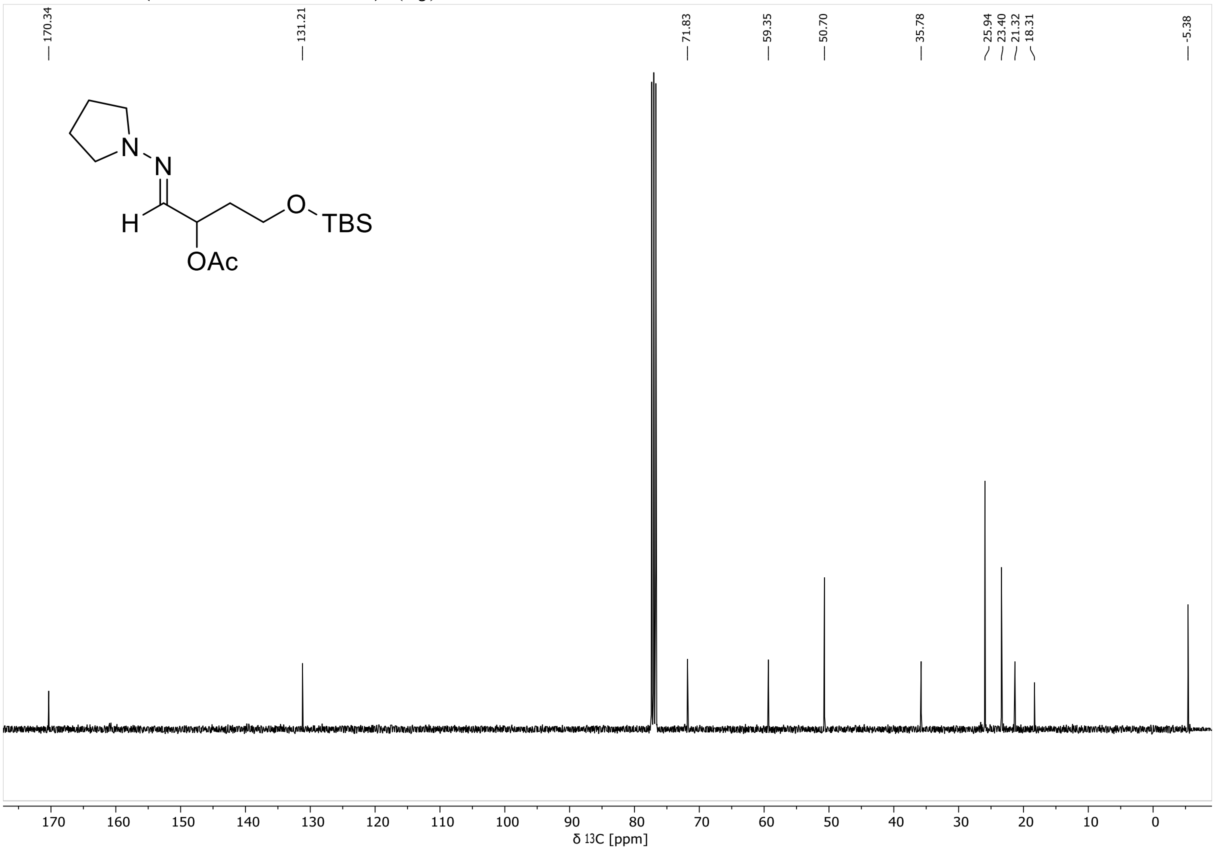

**$^1$H NMR** (400 MHz, $CDCl_3$) (**1k**):

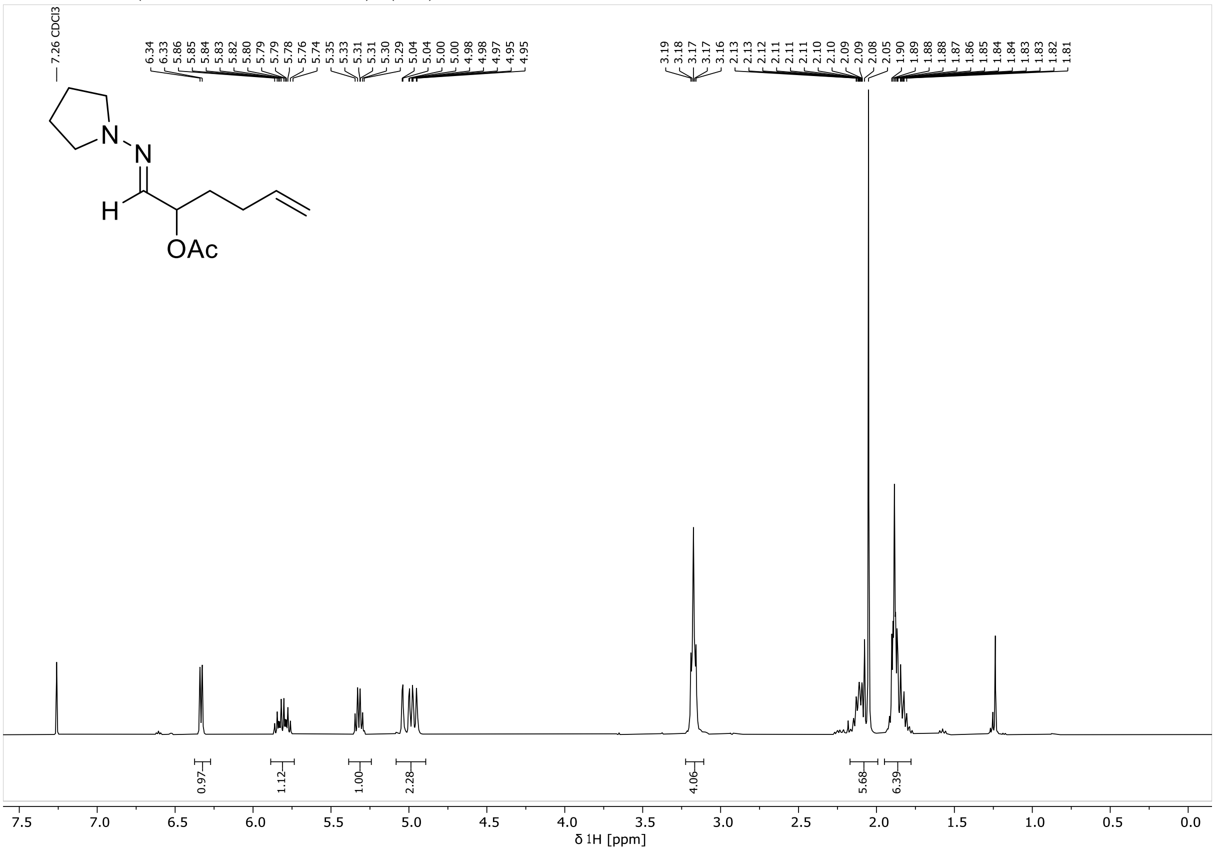

**$^{13}$C NMR** (101 MHz, $CDCl_3$) (**1k**):

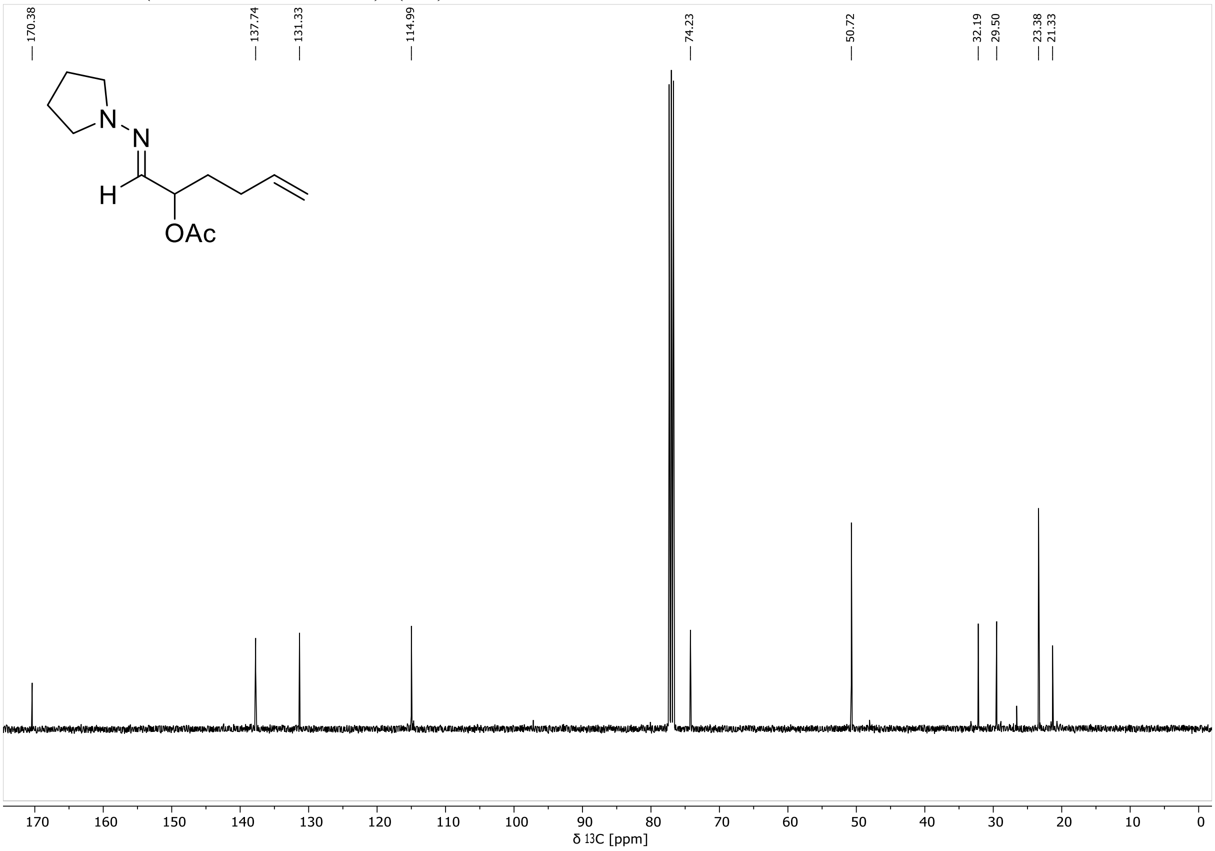

**$^{1}$H NMR** (400 MHz, $CDCl_3$) (**1l**):

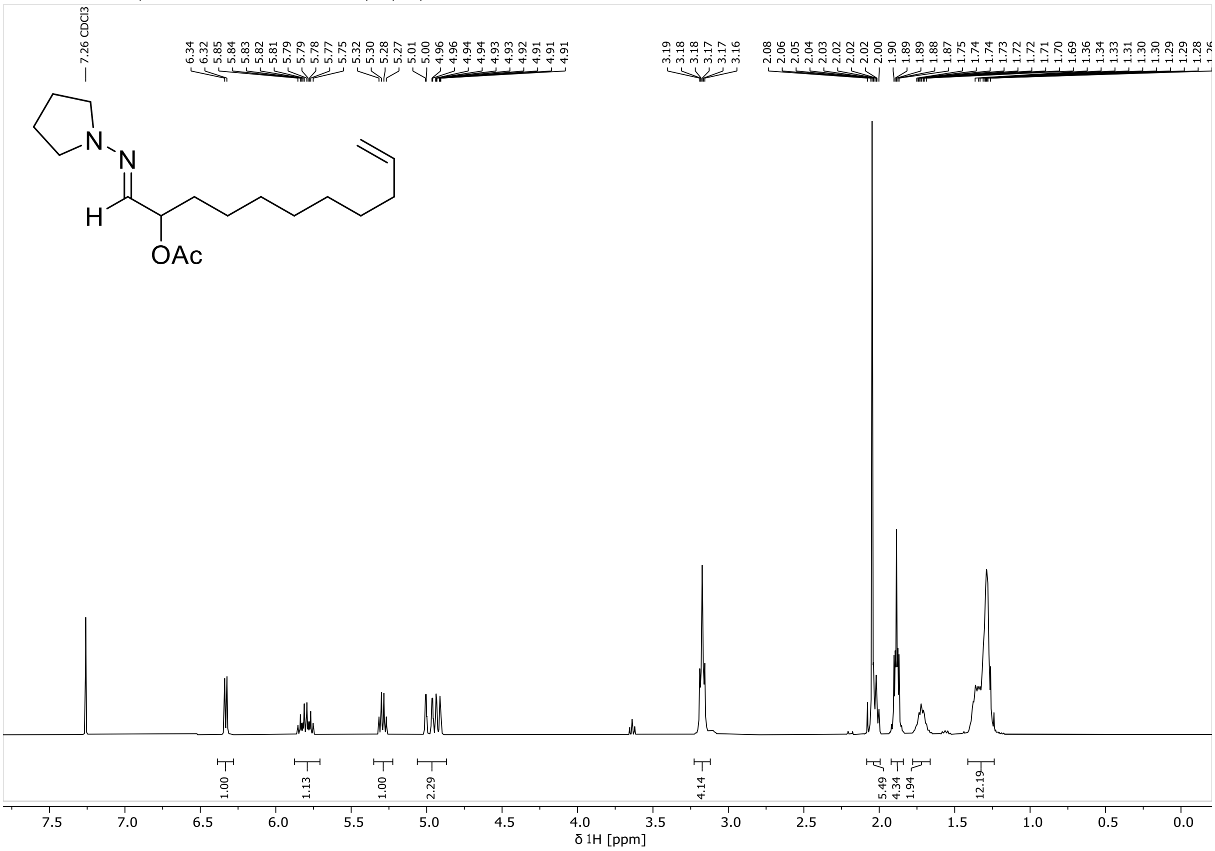


**$^{13}$C NMR** (101 MHz, $CDCl_3$) (**1l**):

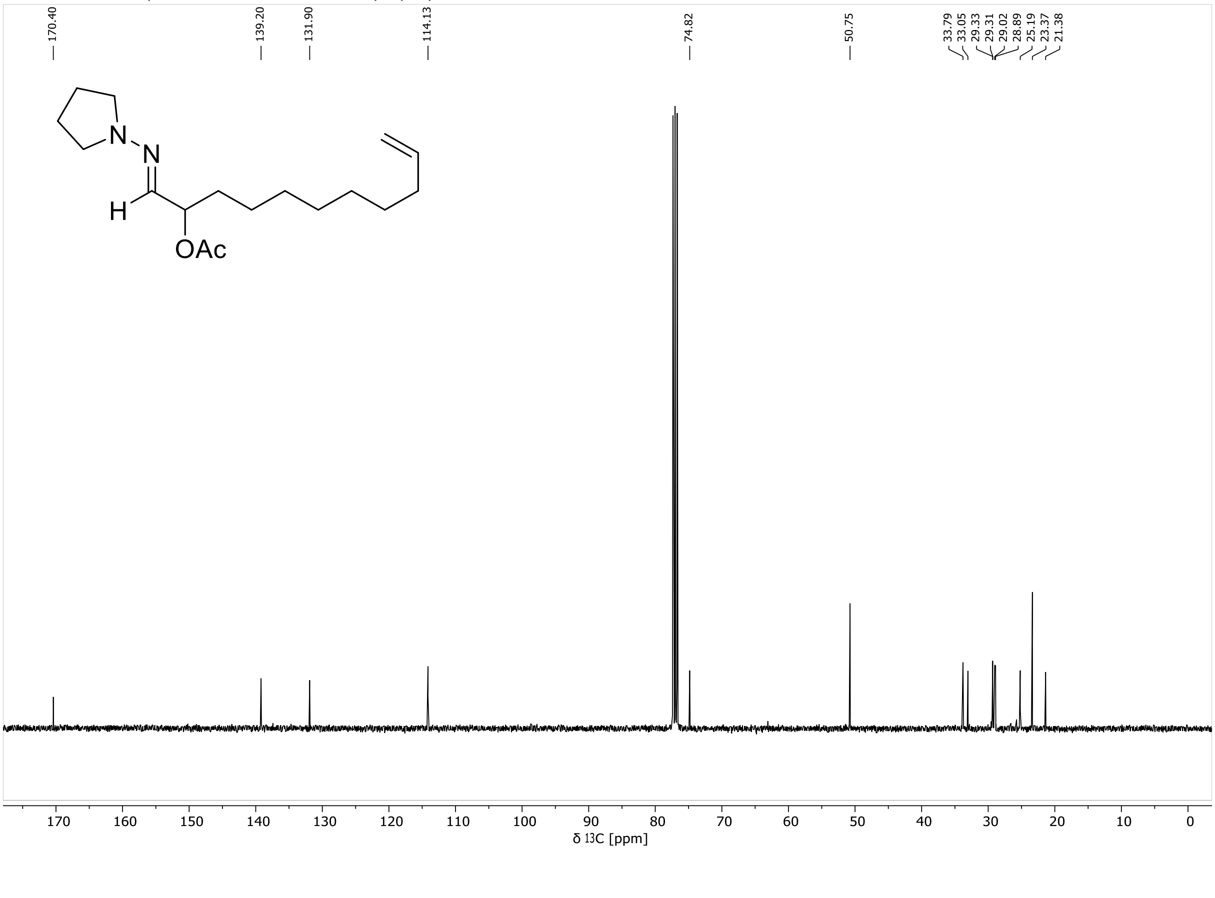

**$^{1}$H NMR** (300 MHz, $CDCl_3$) (**1m**):

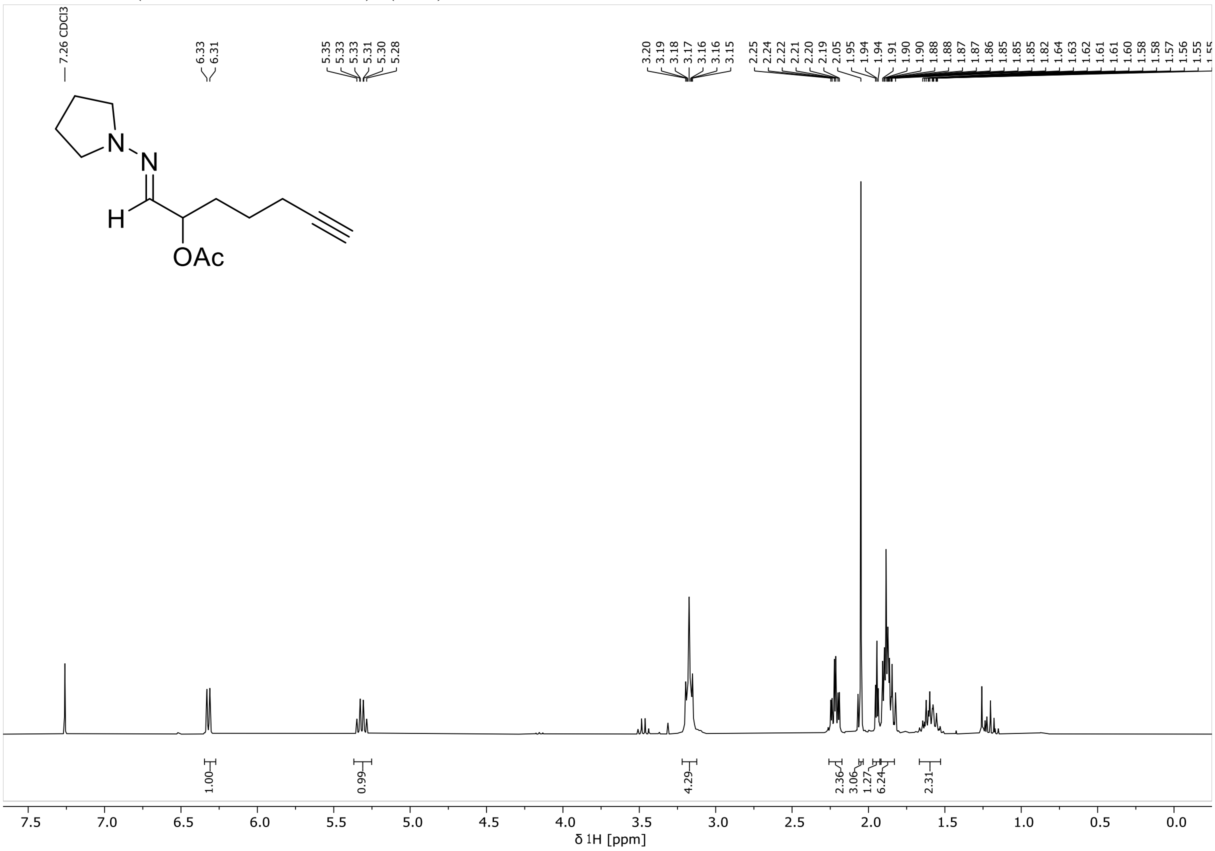

**$^{13}$C NMR** (101 MHz, $CDCl_3$) (**1m**):

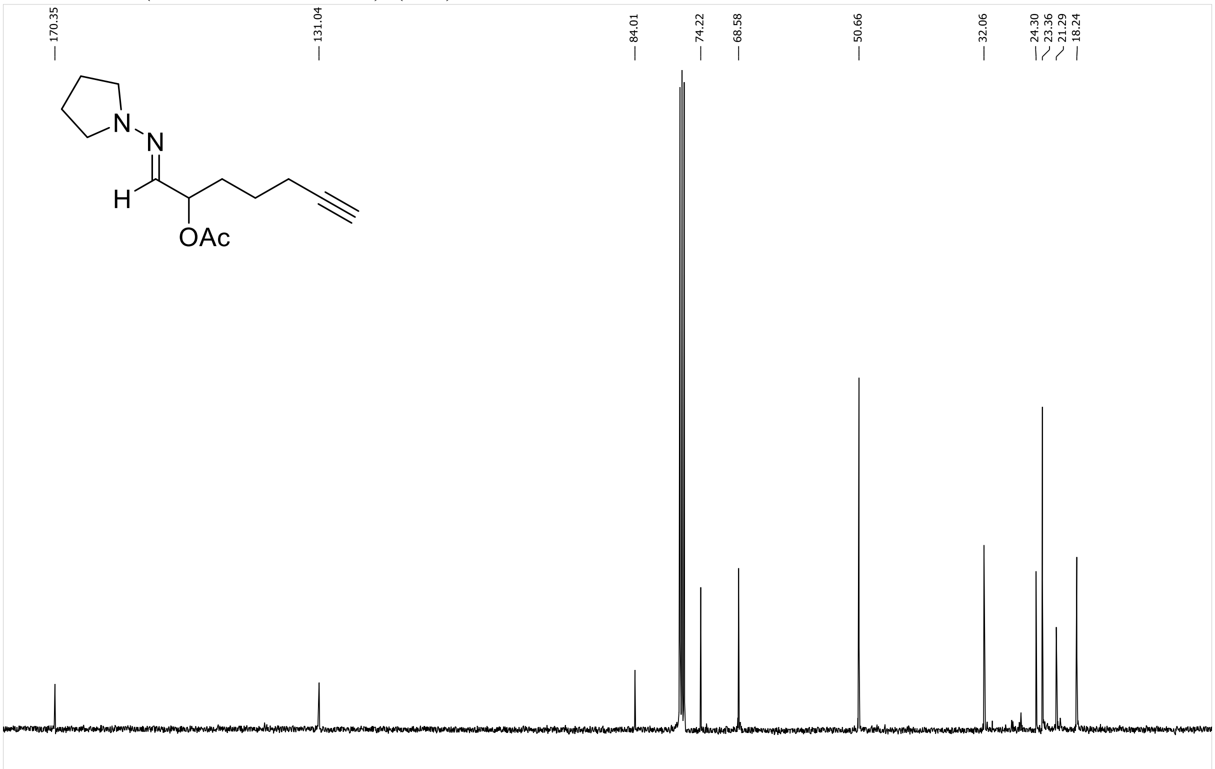

$^{1}$**H NMR** (400 MHz, $CDCl_3$) (**1n**):

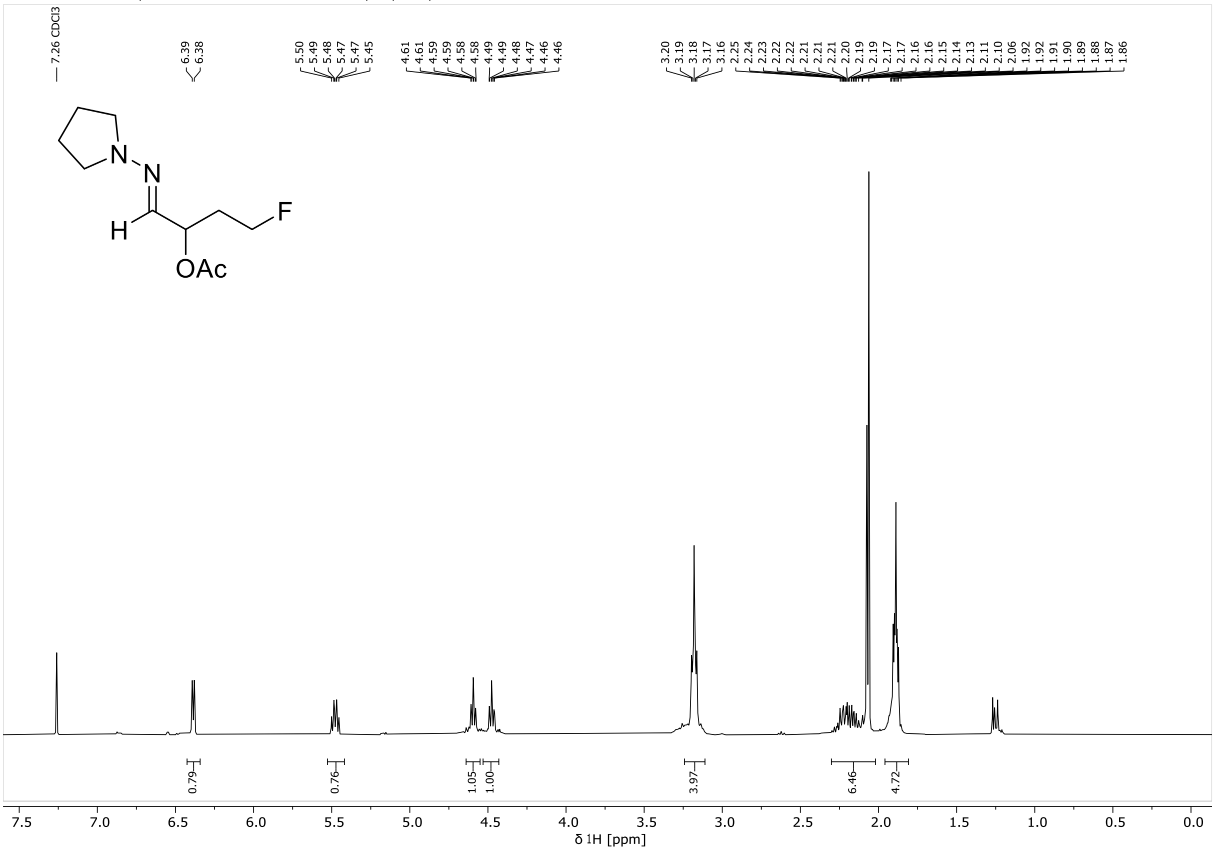


$^{13}$**C NMR** (101 MHz, $CDCl_3$) (**1n**):

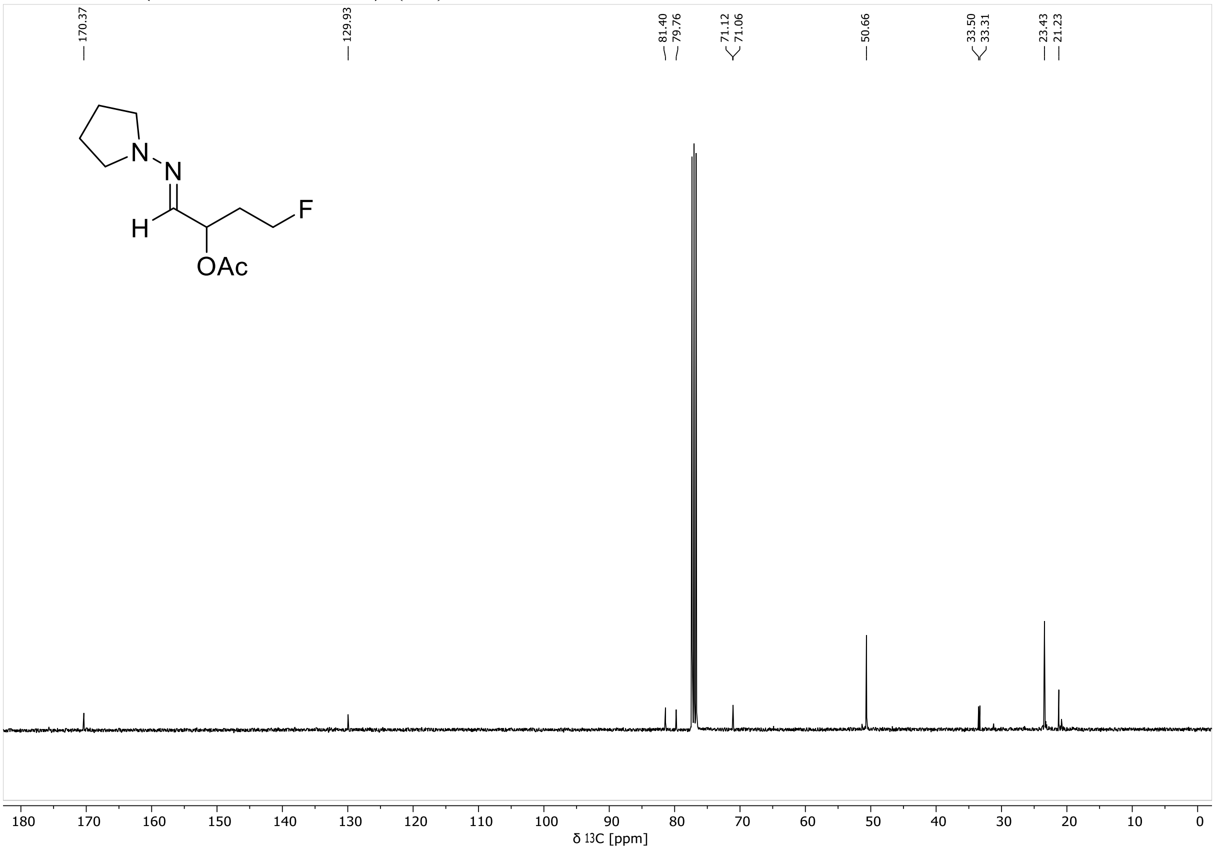

**$^{1}$H NMR** (400 MHz, CDCl$_3$) (**1o**):

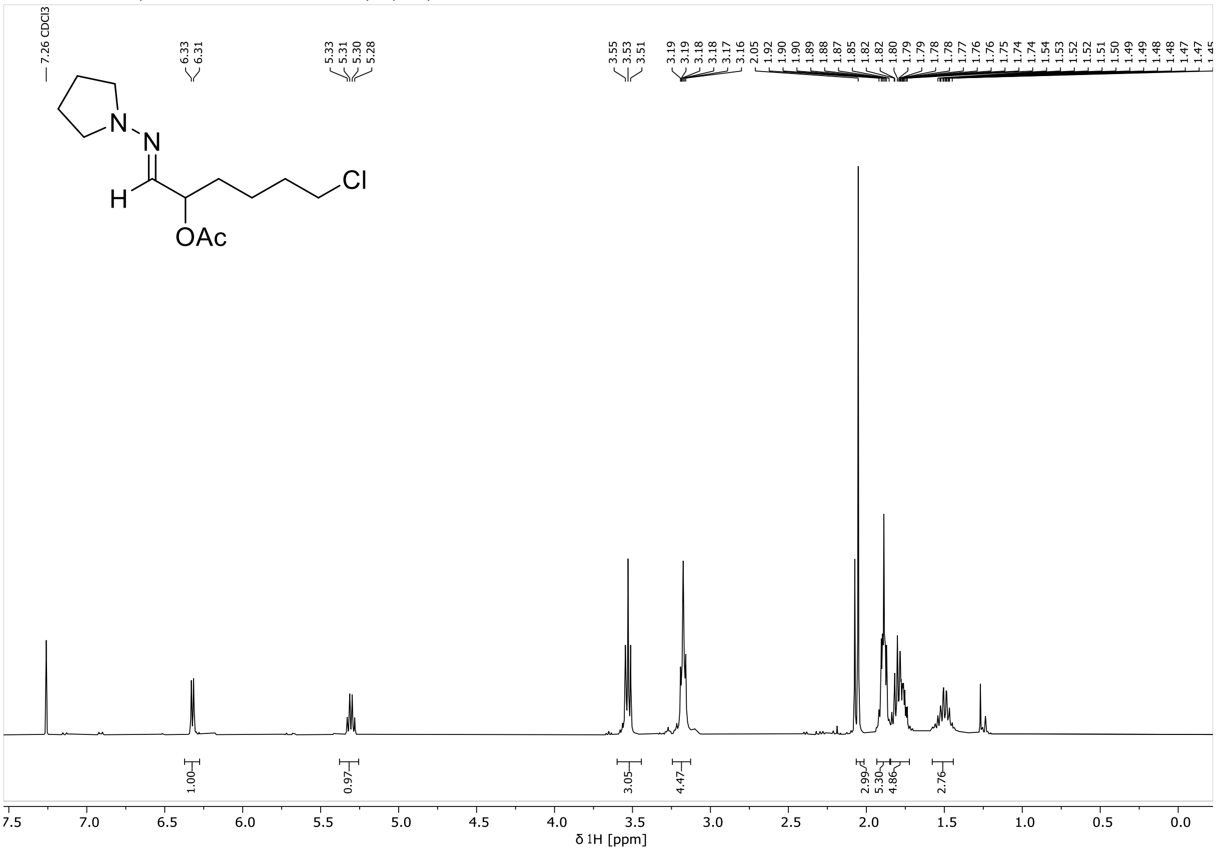

**$^{13}$C NMR** (101 MHz, CDCl$_3$) (**1o**):

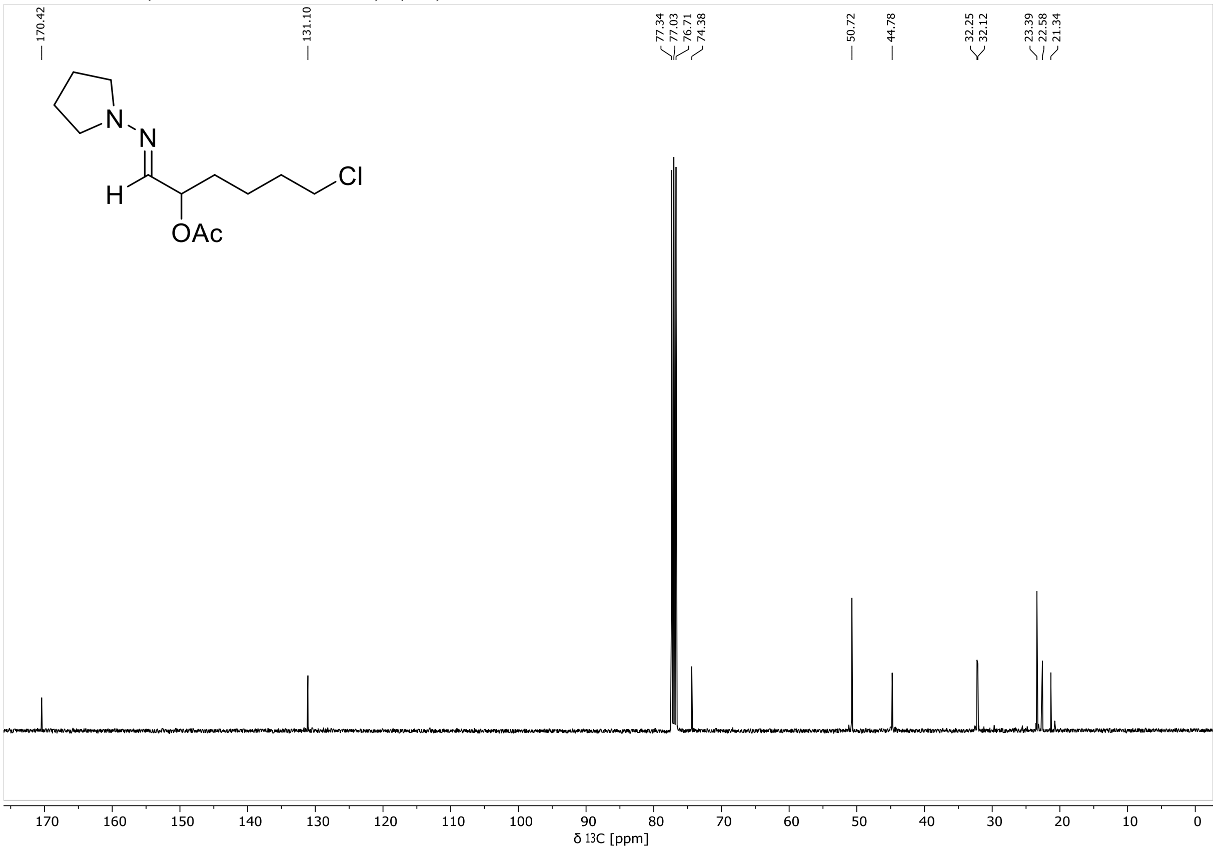

**$^{1}$H NMR** (400 MHz, $CDCl_3$) (**1p**):

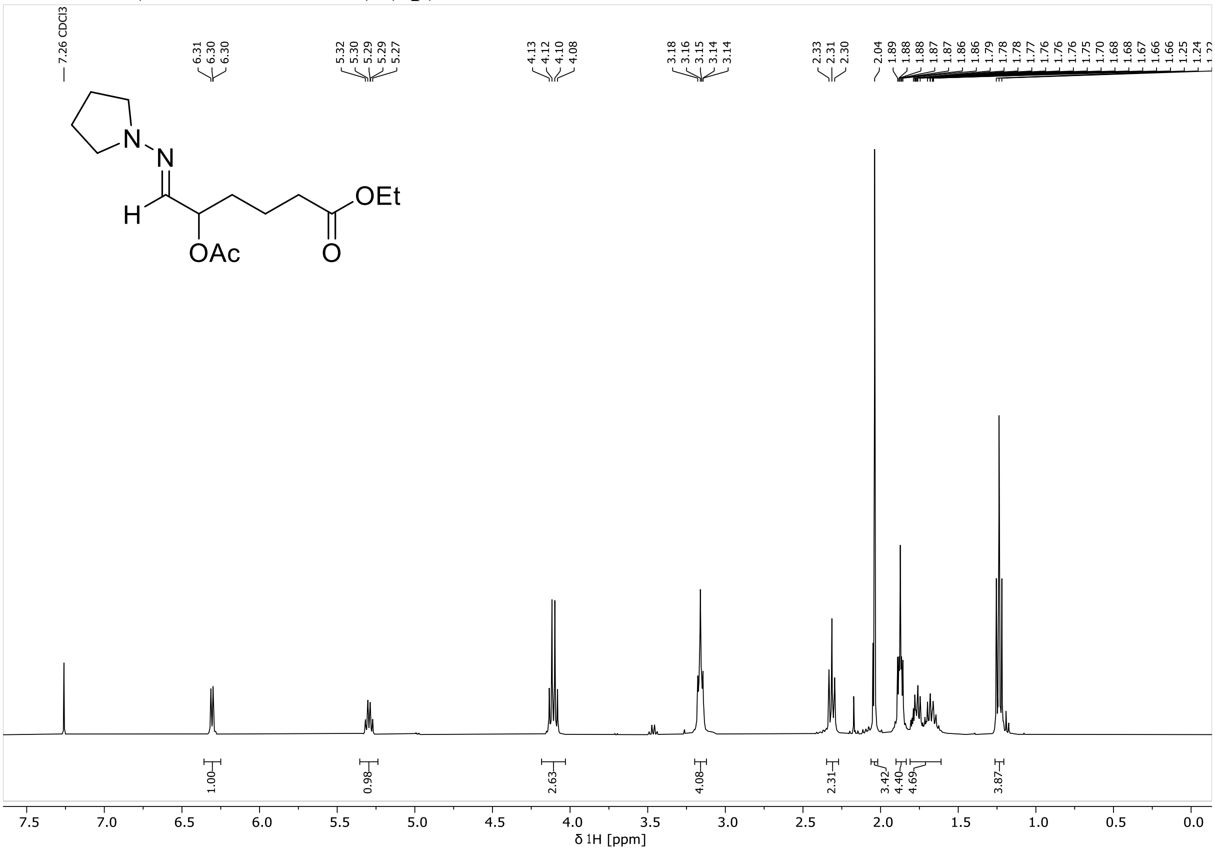

**$^{13}$C NMR** (101 MHz, $CDCl_3$) (**1p**):

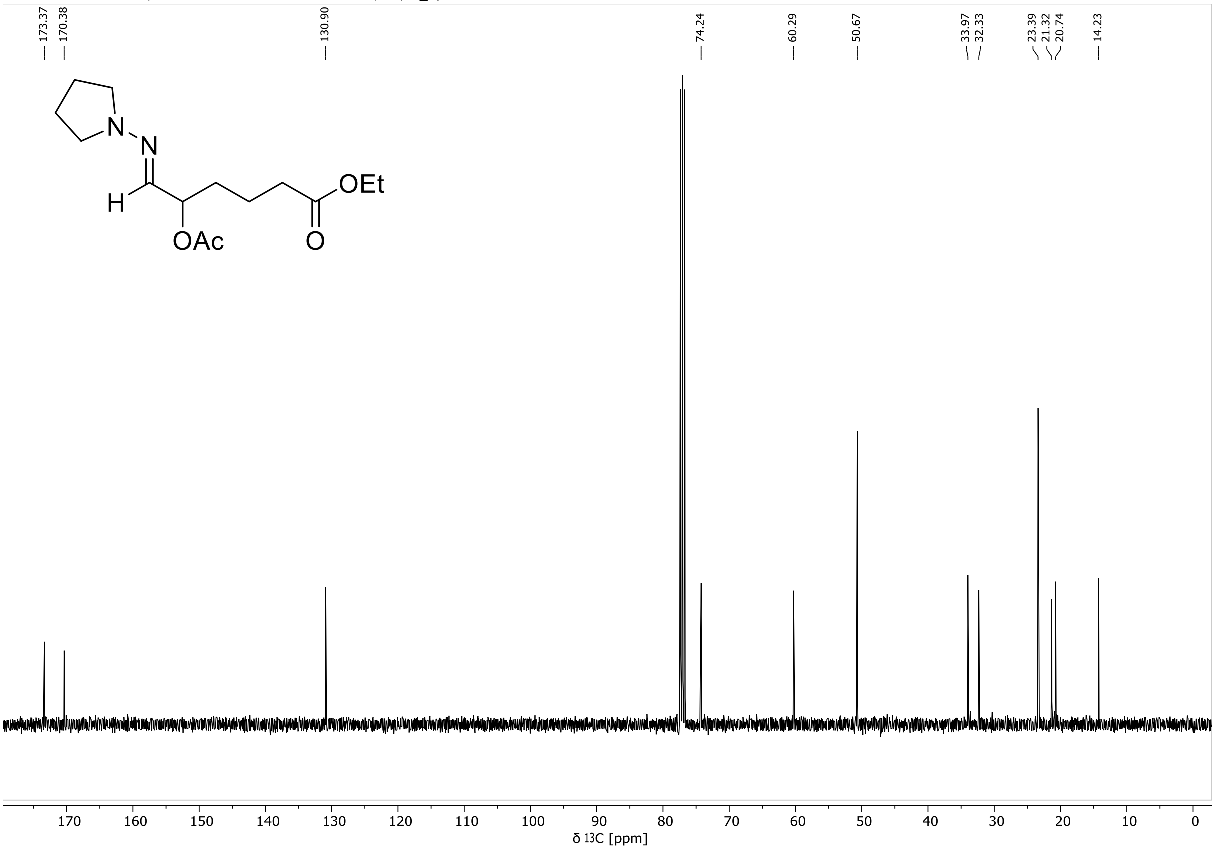

**$^1$H NMR** (400 MHz, $CDCl_3$) (**1q**):

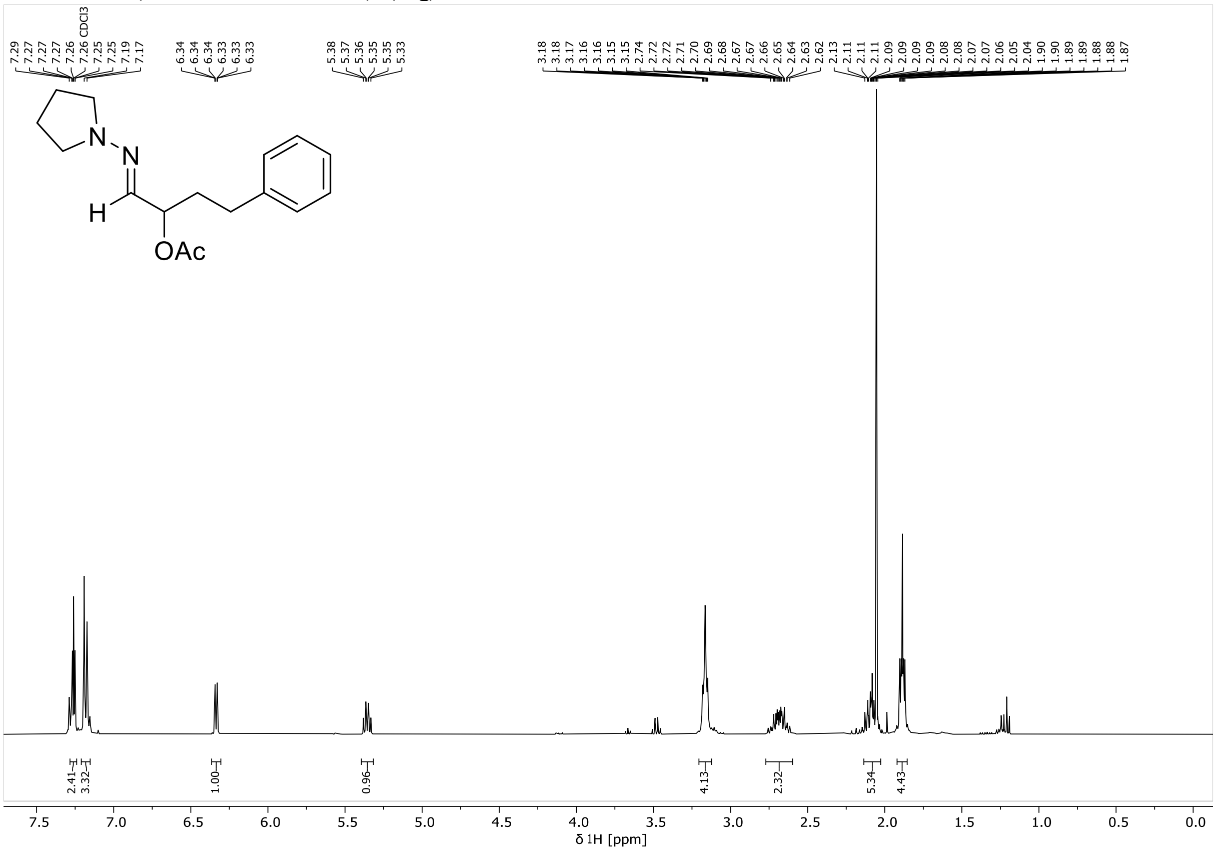


**$^{13}$C NMR** (101 MHz, $CDCl_3$) (**1q**):

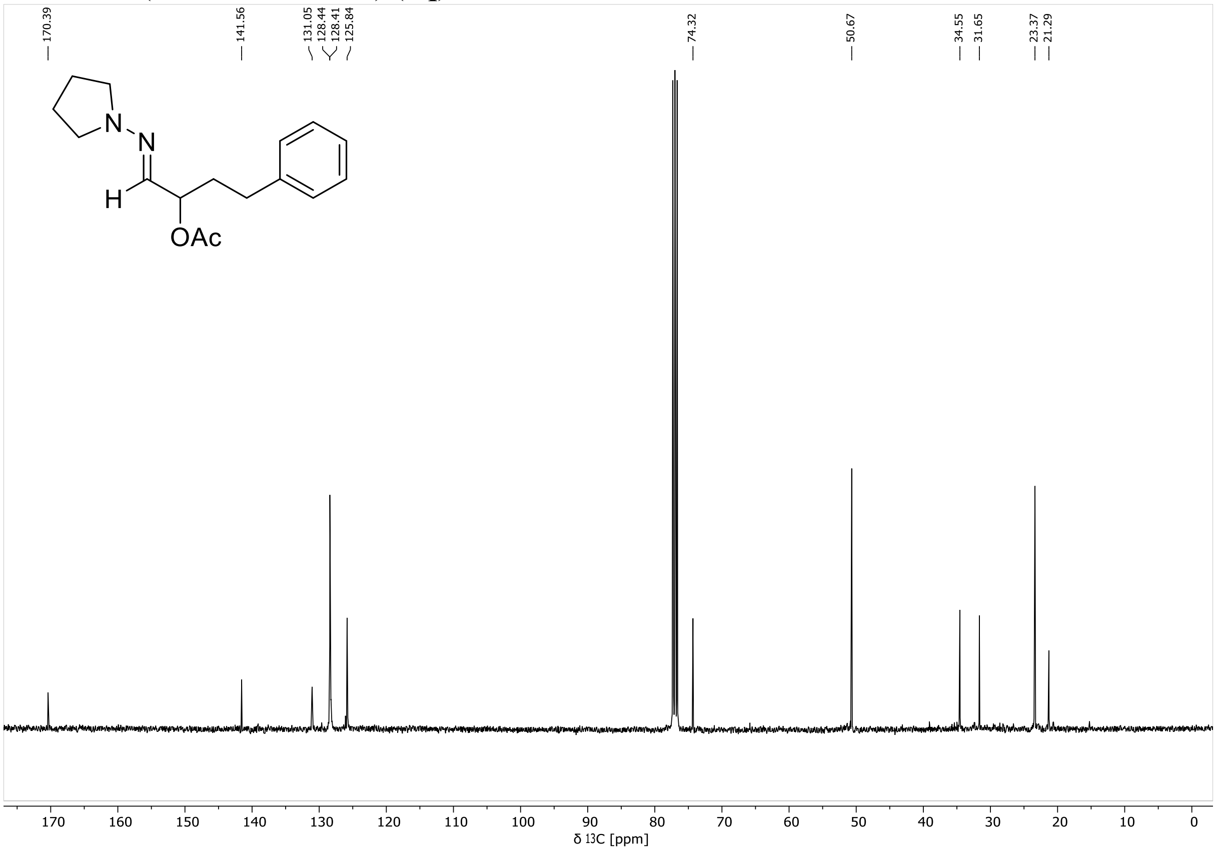

**$^{1}$H NMR** (400 MHz, $CDCl_3$) (**1r**):

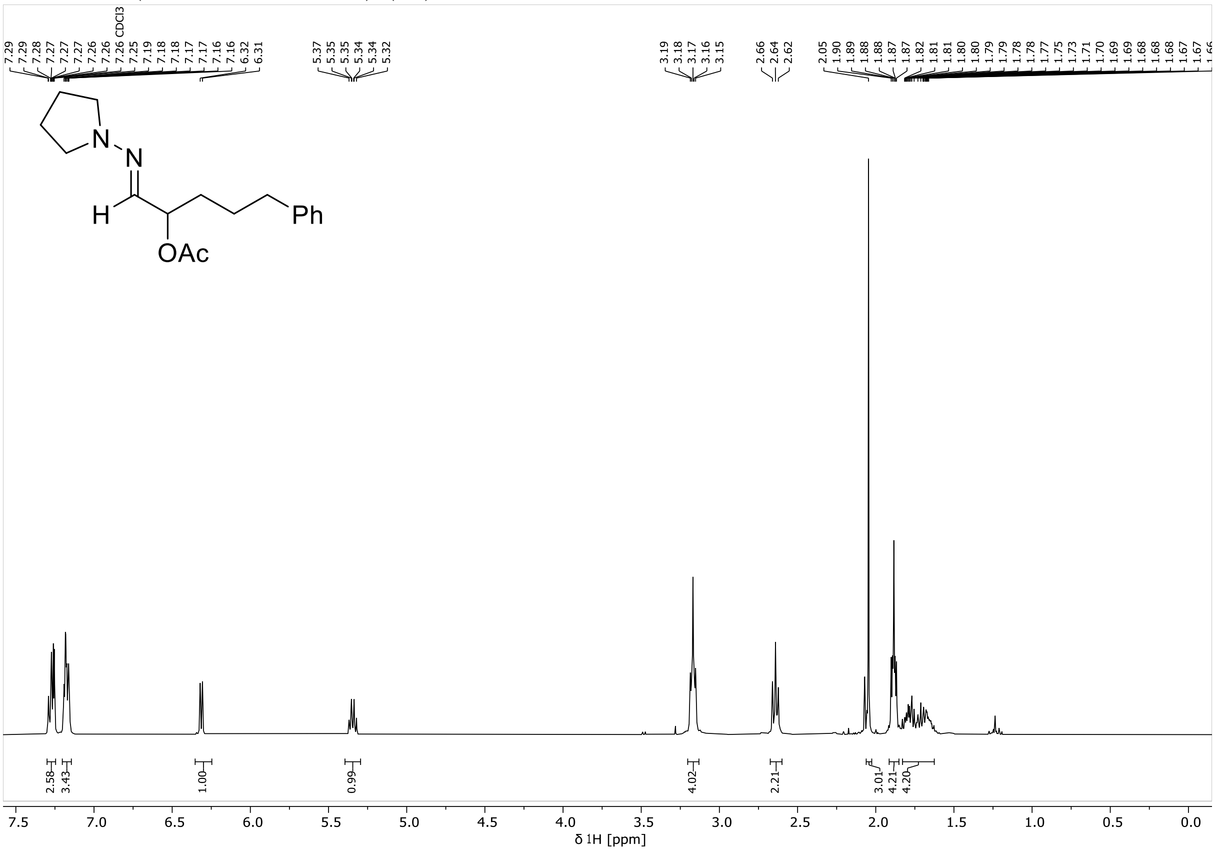

**$^{13}$C NMR** (101 MHz, $CDCl_3$) (**1r**):

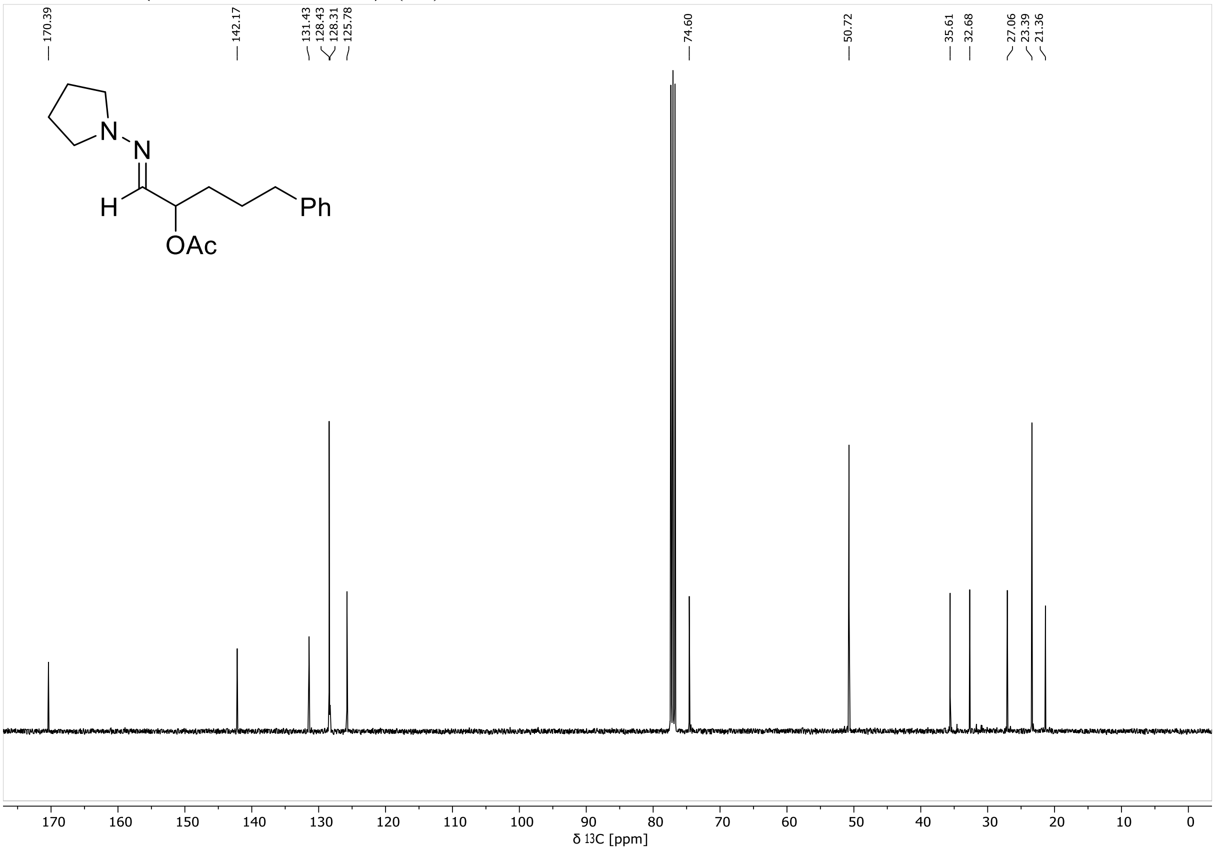

**$^{1}$H NMR** (400 MHz, $CDCl_3$) (**1s**):

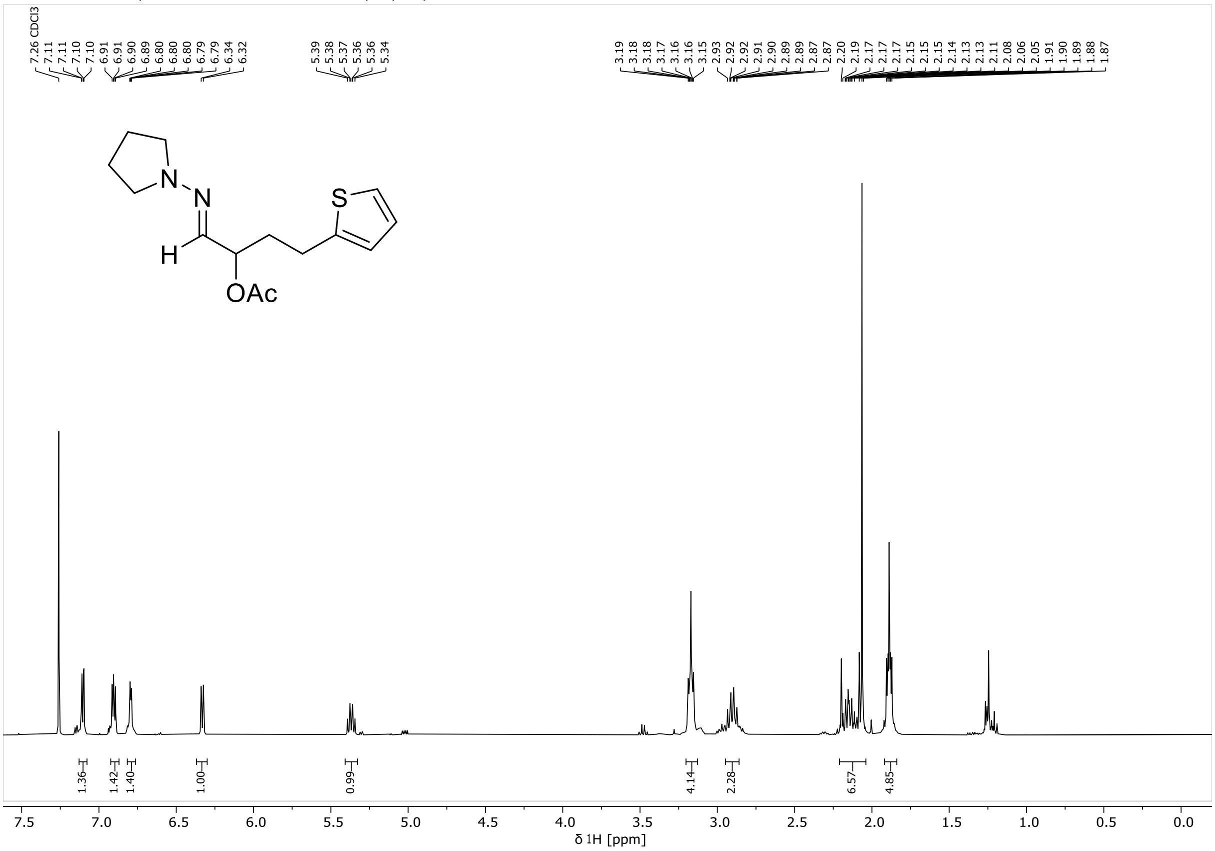

**$^{13}$C NMR** (101 MHz, $CDCl_3$) (**1s**):

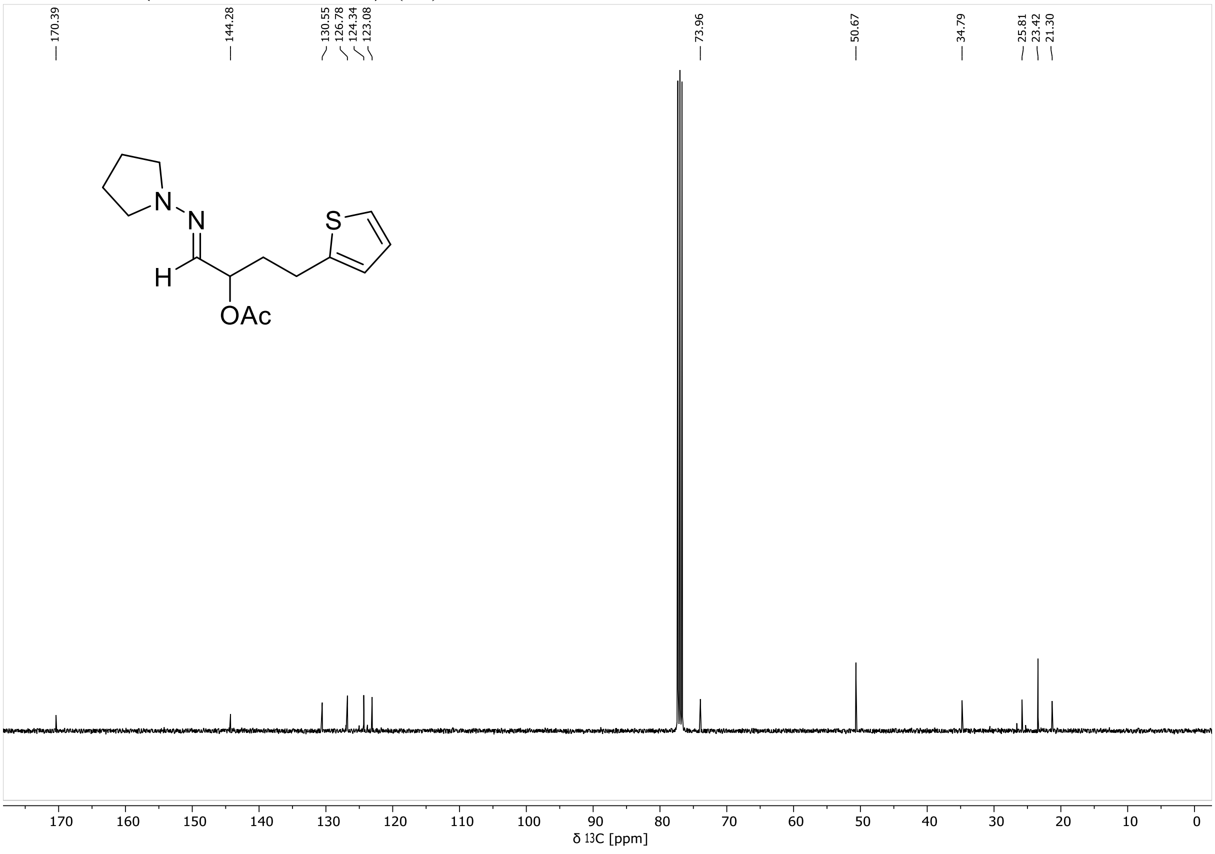

**$^1$H NMR** (400 MHz, $CDCl_3$) (**1t**):

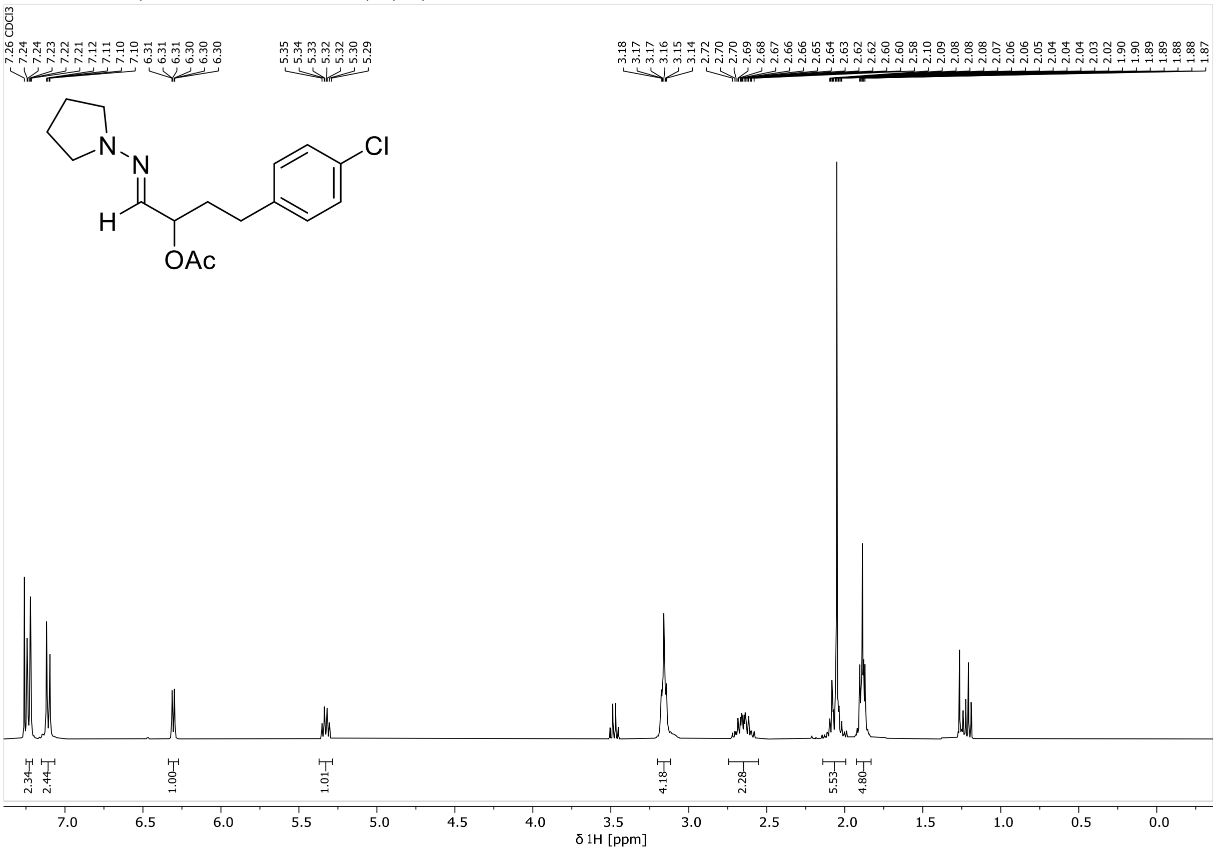

**$^{13}$C NMR** (101 MHz, $CDCl_3$) (**1t**):

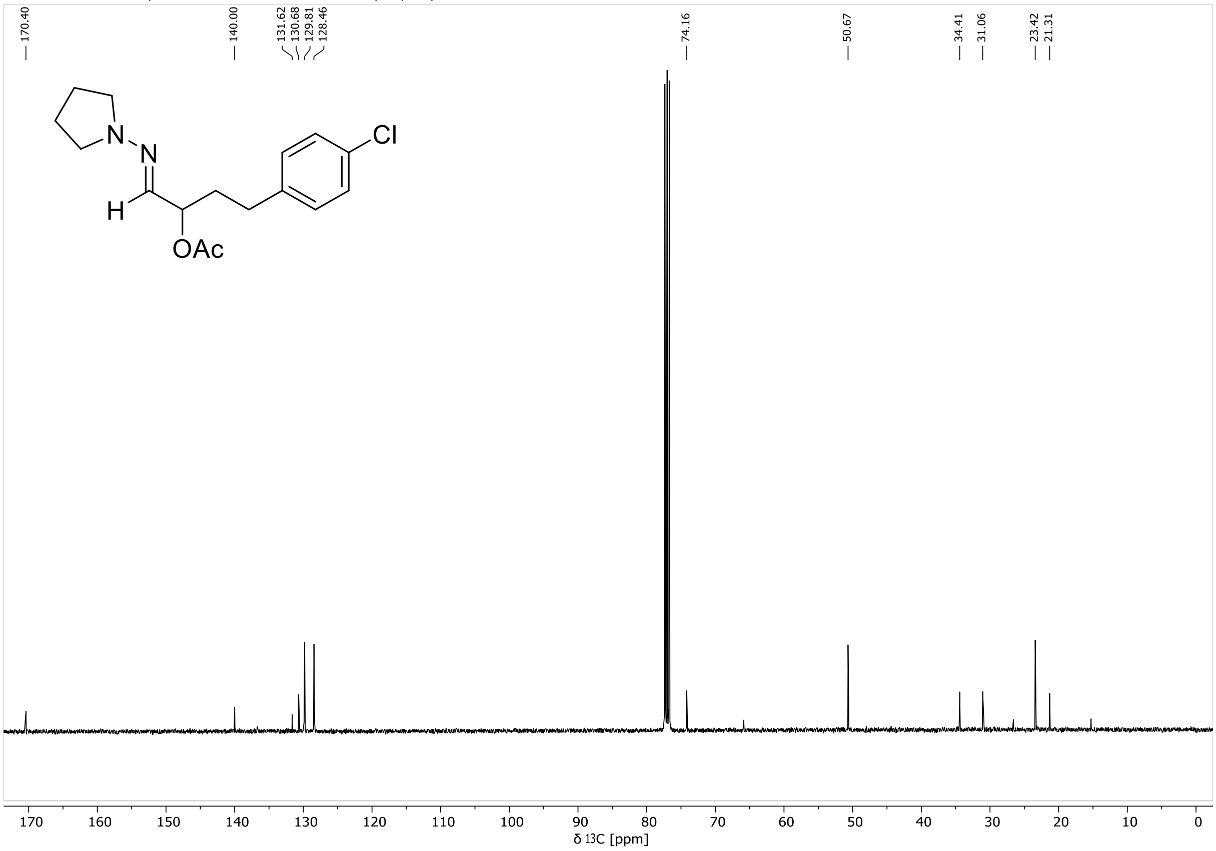

**$^{1}$H NMR** (400 MHz, $CDCl_3$) (**1u**):

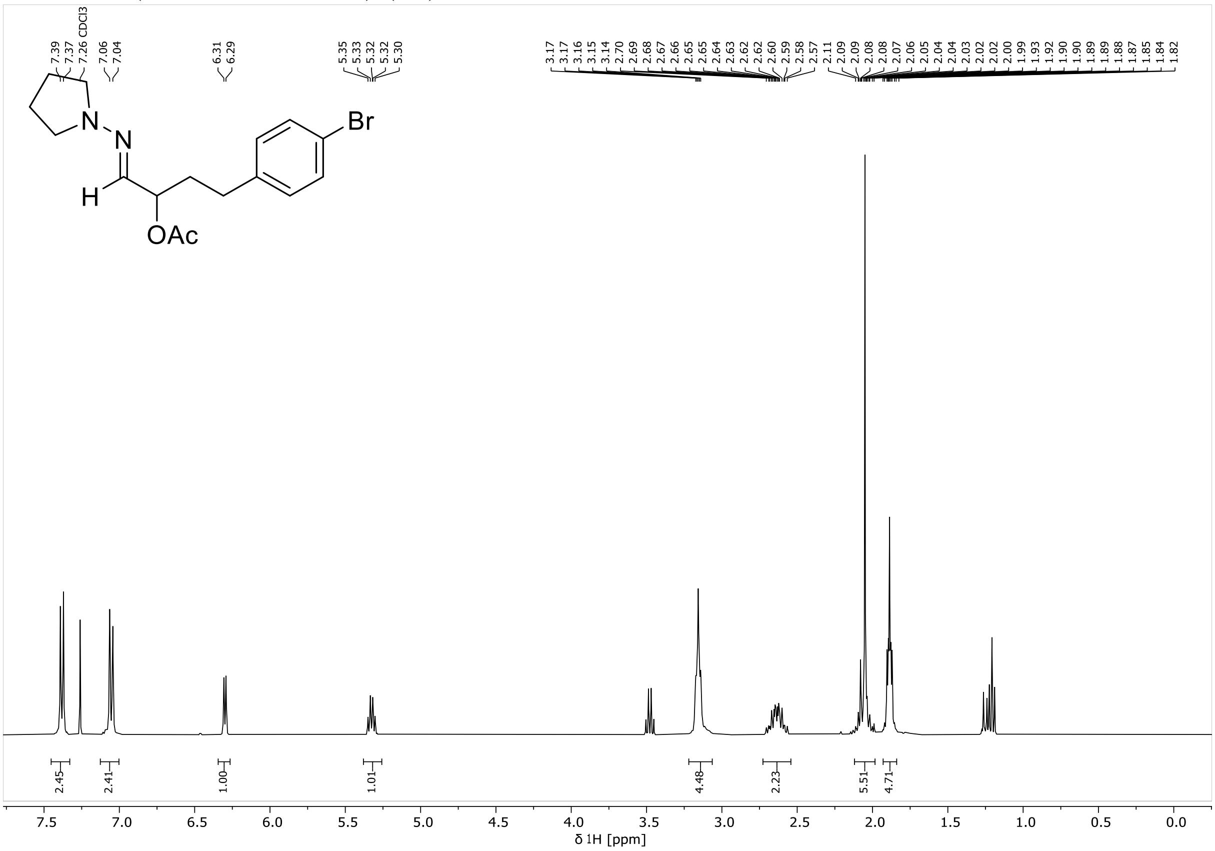

**$^{13}$C NMR** (101 MHz, $CDCl_3$) (**1u**):

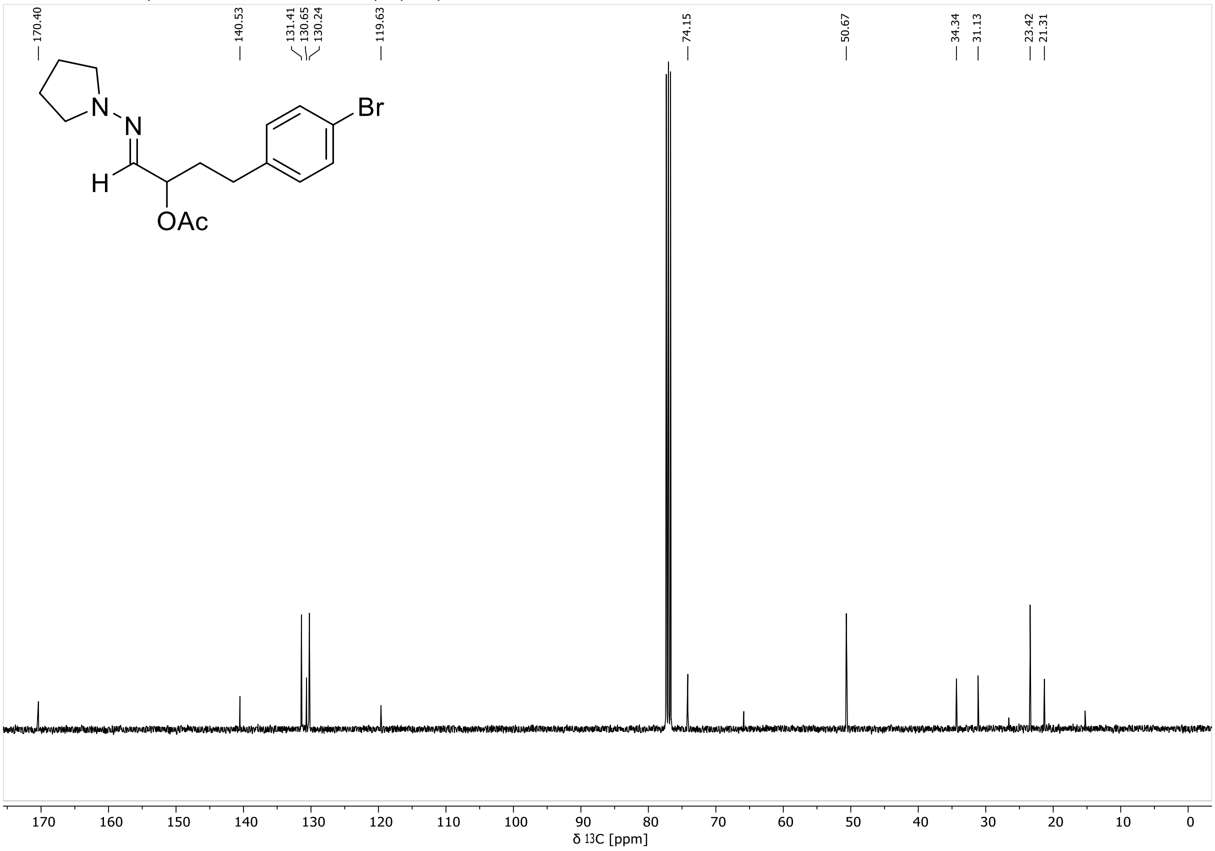

**$^{1}$H NMR** (400 MHz, $CDCl_3$) (**1w**):

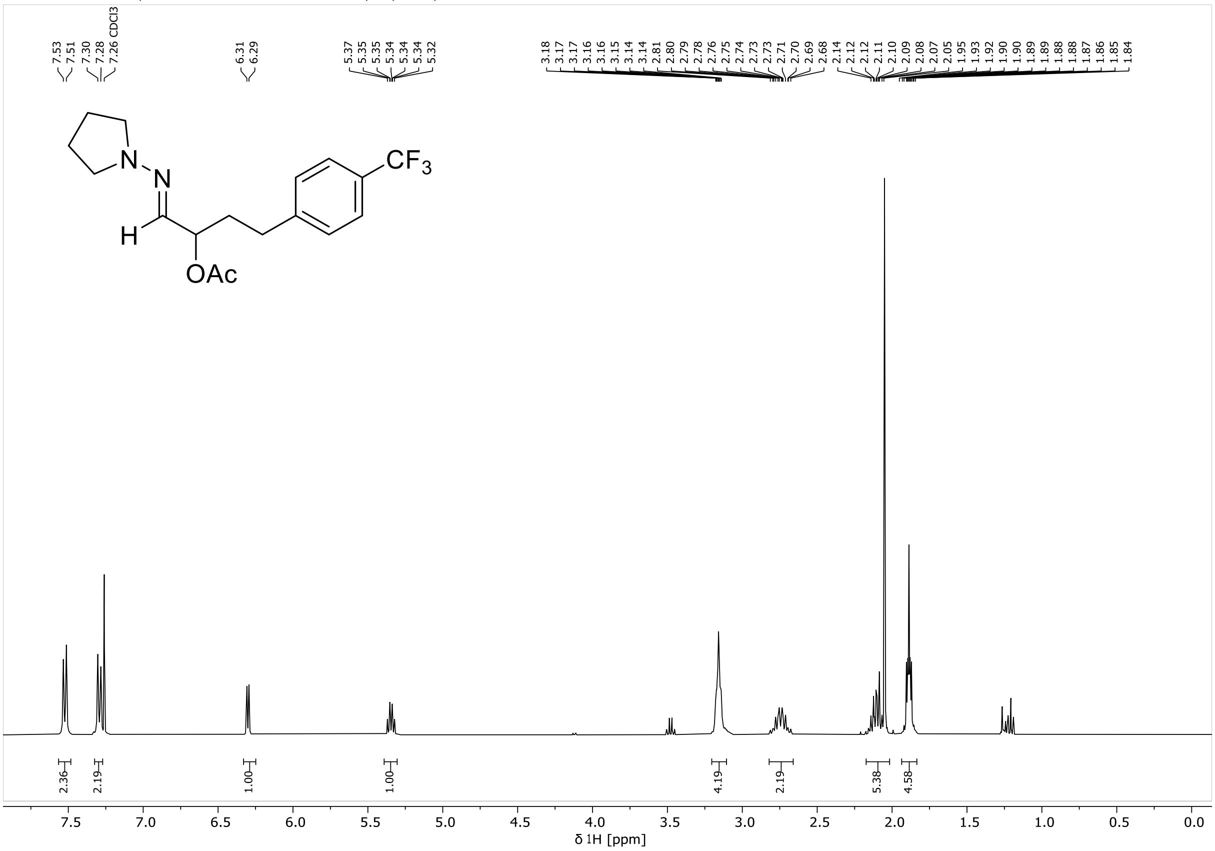


**$^{13}$C NMR** (101 MHz, $CDCl_3$) (**1w**):

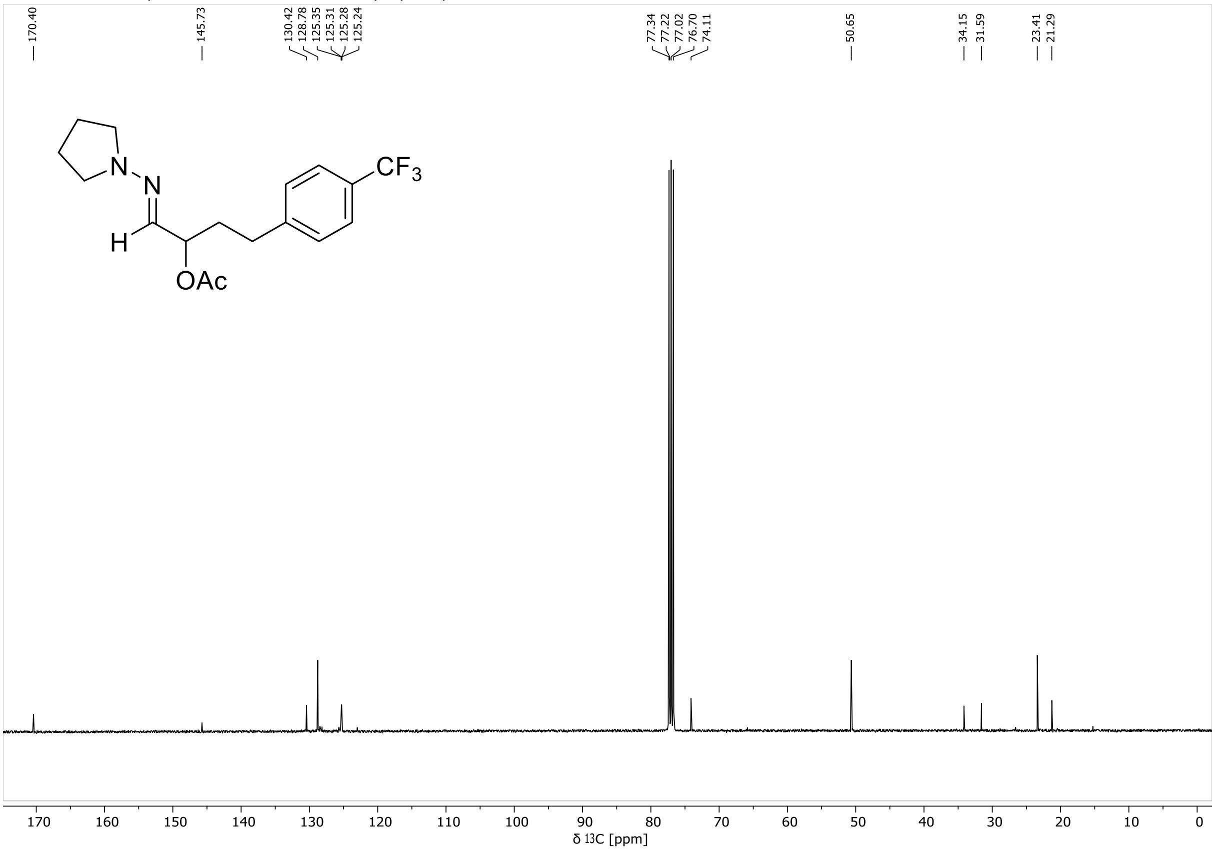

**$^{1}$H NMR** (400 MHz, $CDCl_3$) (**1v**):

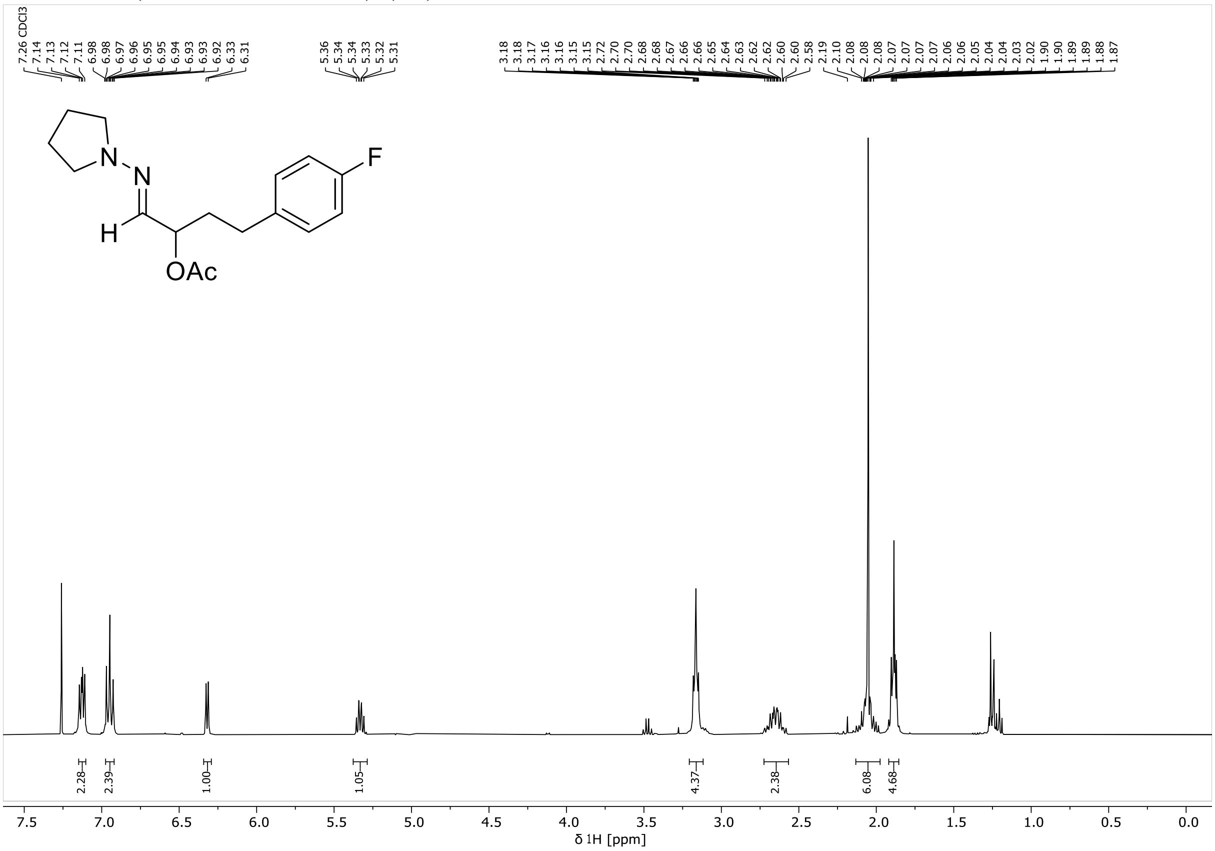

**$^{13}$C NMR** (101 MHz, $CDCl_3$) (**1v**):

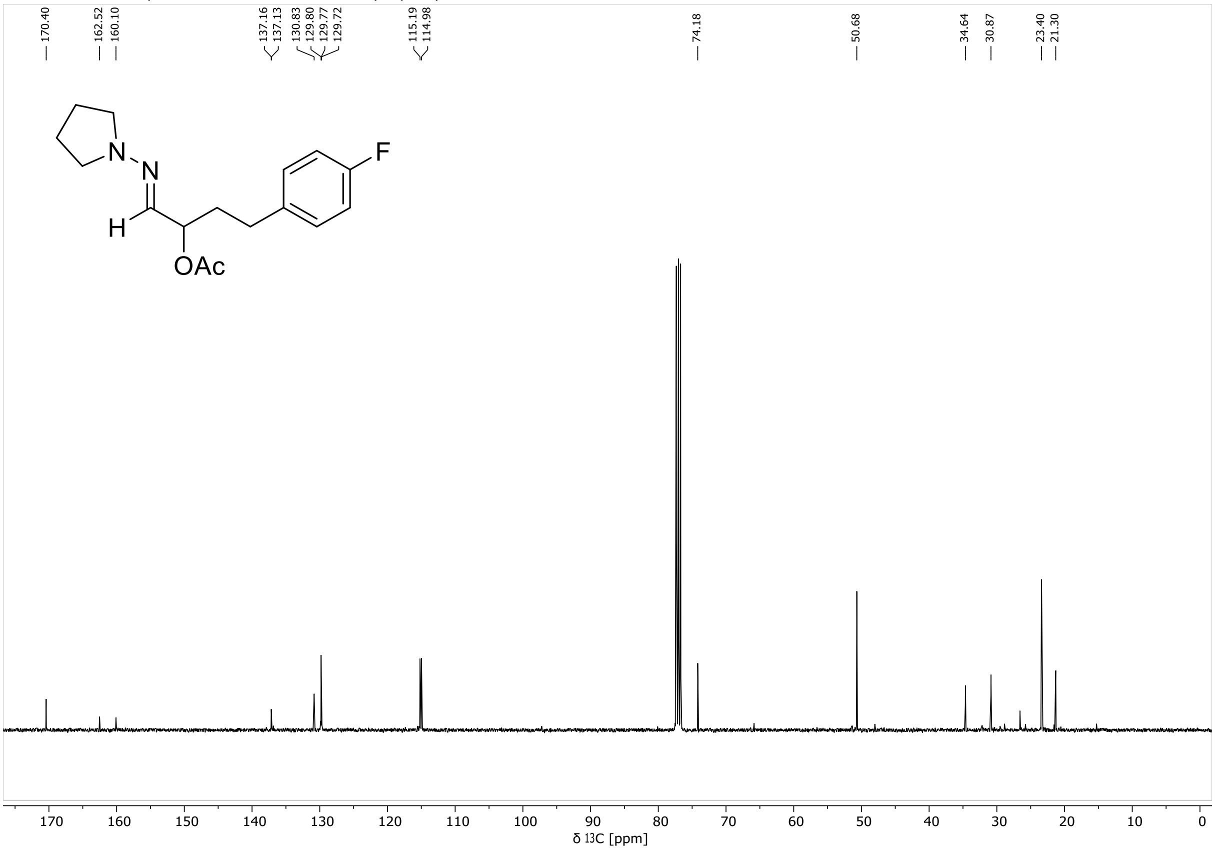

**$^{19}$F NMR** (377 MHz, $CDCl_3$) (**1v**):

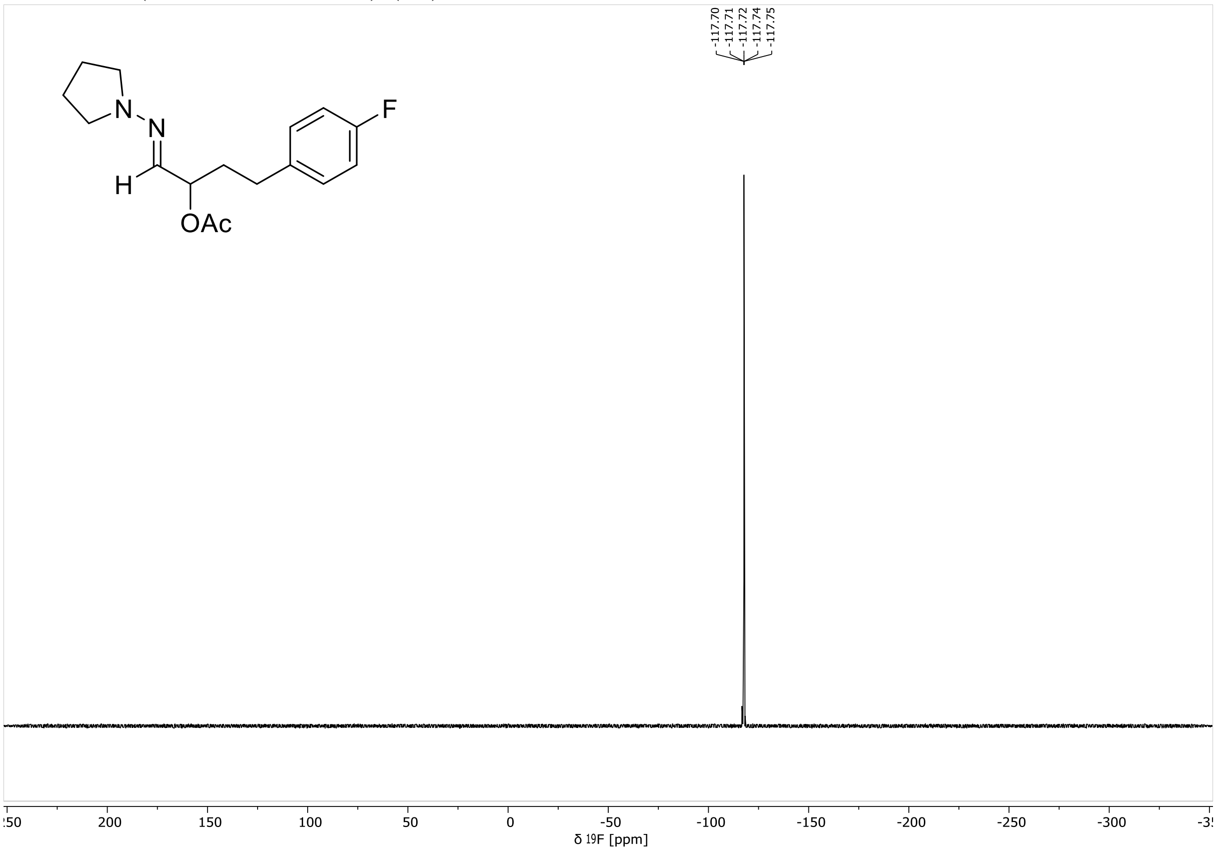


**$^{19}$F NMR** (377 MHz, $CDCl_3$) (**1w**):

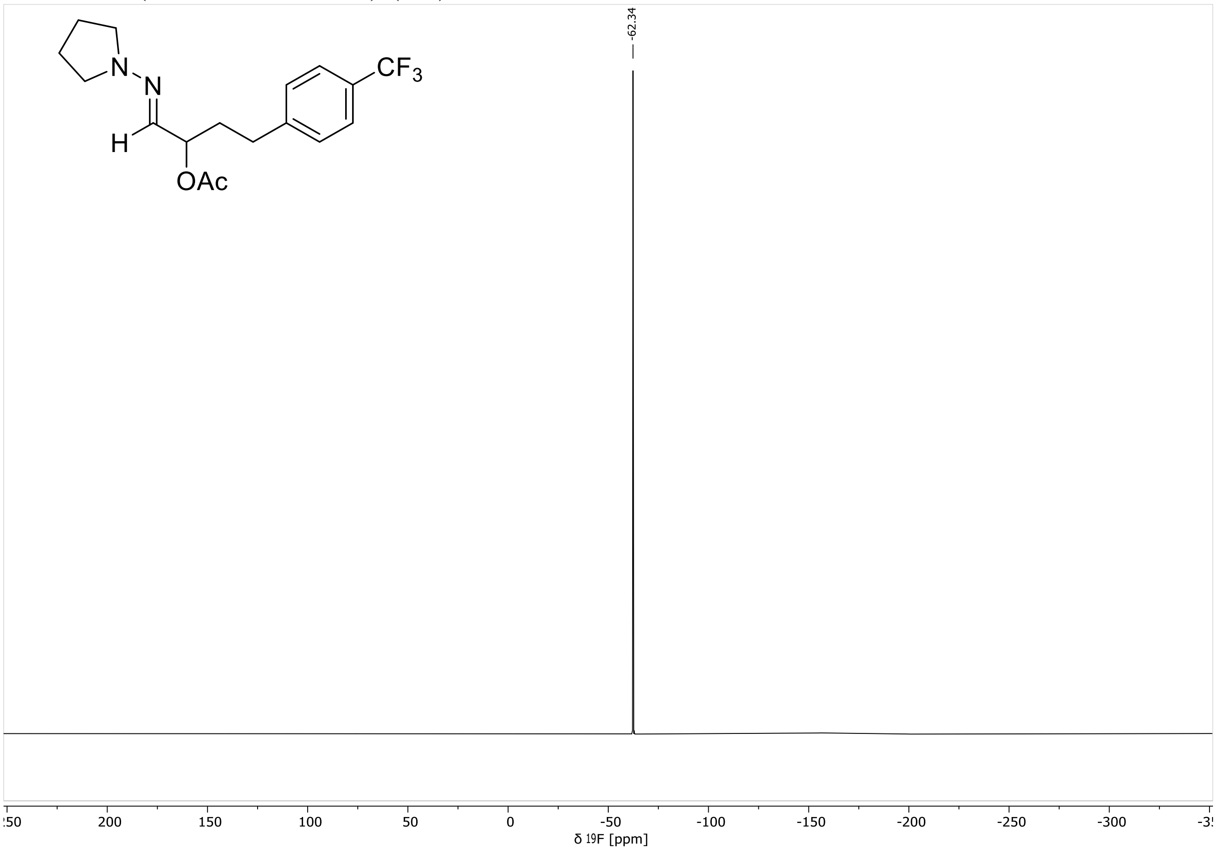

**$^{1}$H NMR** (400 MHz, $CDCl_3$) (**1x**):

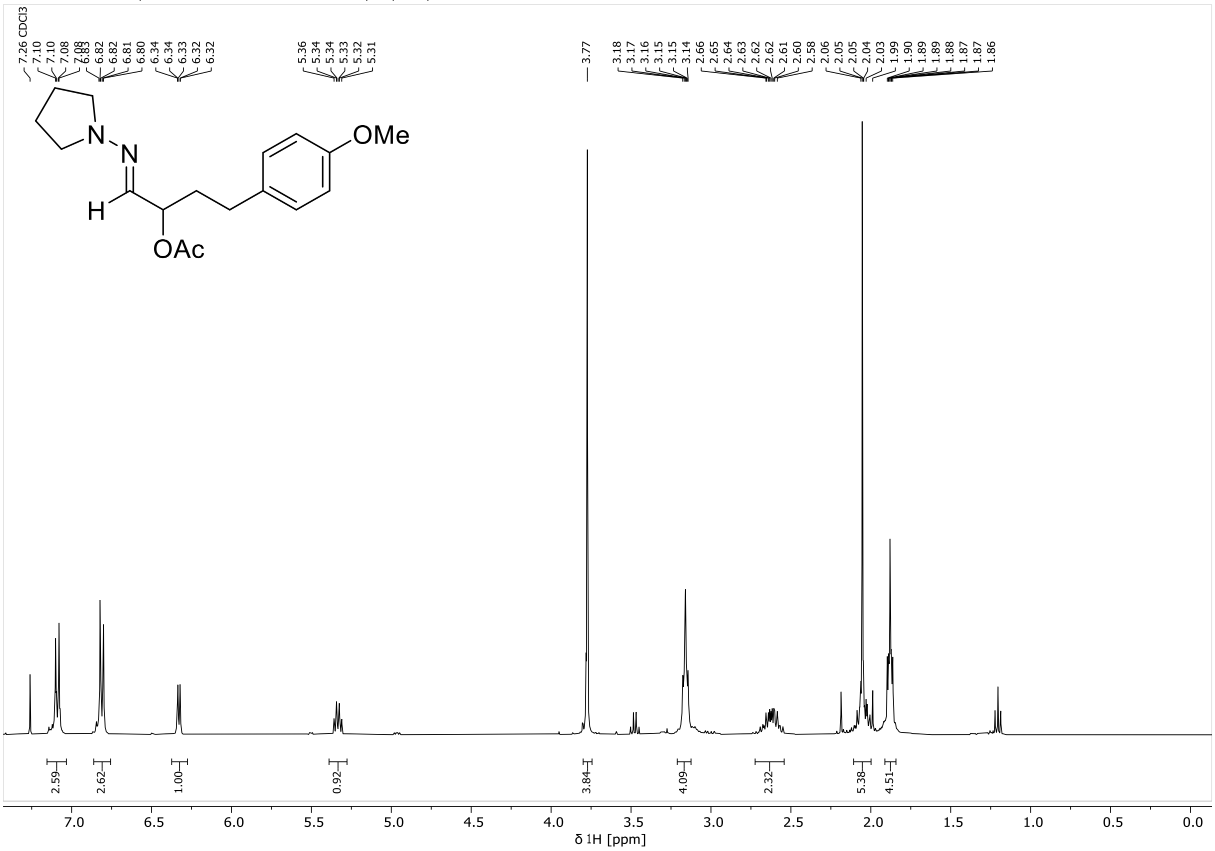

**$^{13}$C NMR** (101 MHz, $CDCl_3$) (**1x**):

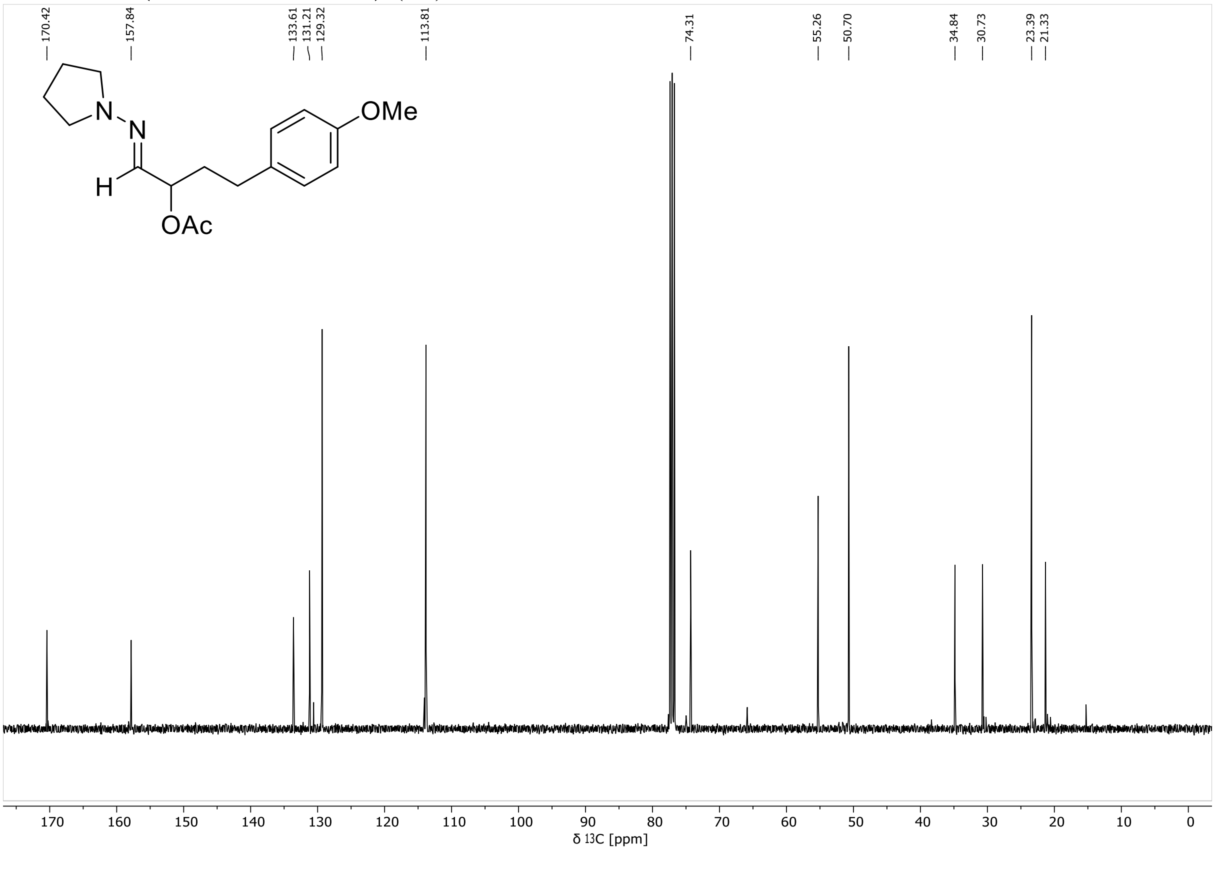

**$^{1}$H NMR** (400 MHz, $CDCl_3$) (**1y**):

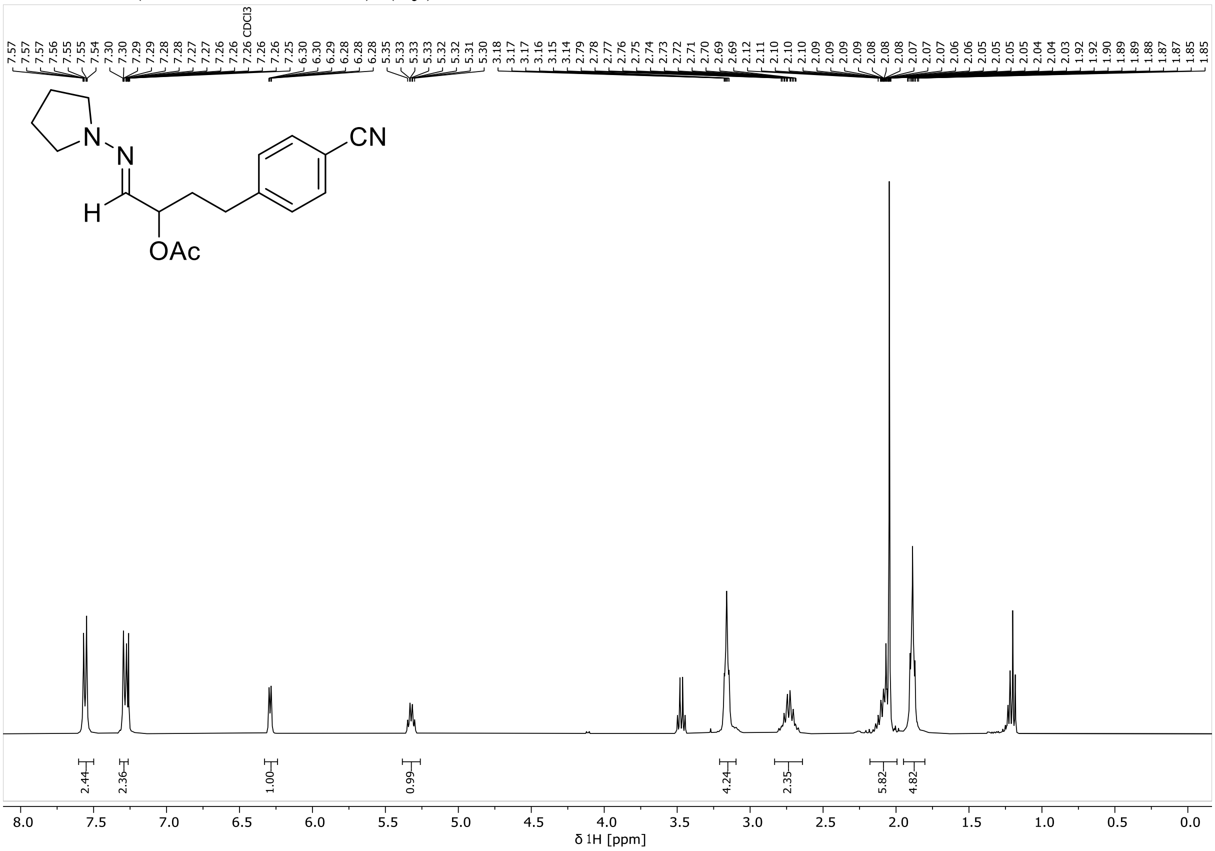


**$^{13}$C NMR** (101 MHz, $CDCl_3$) (**1y**):

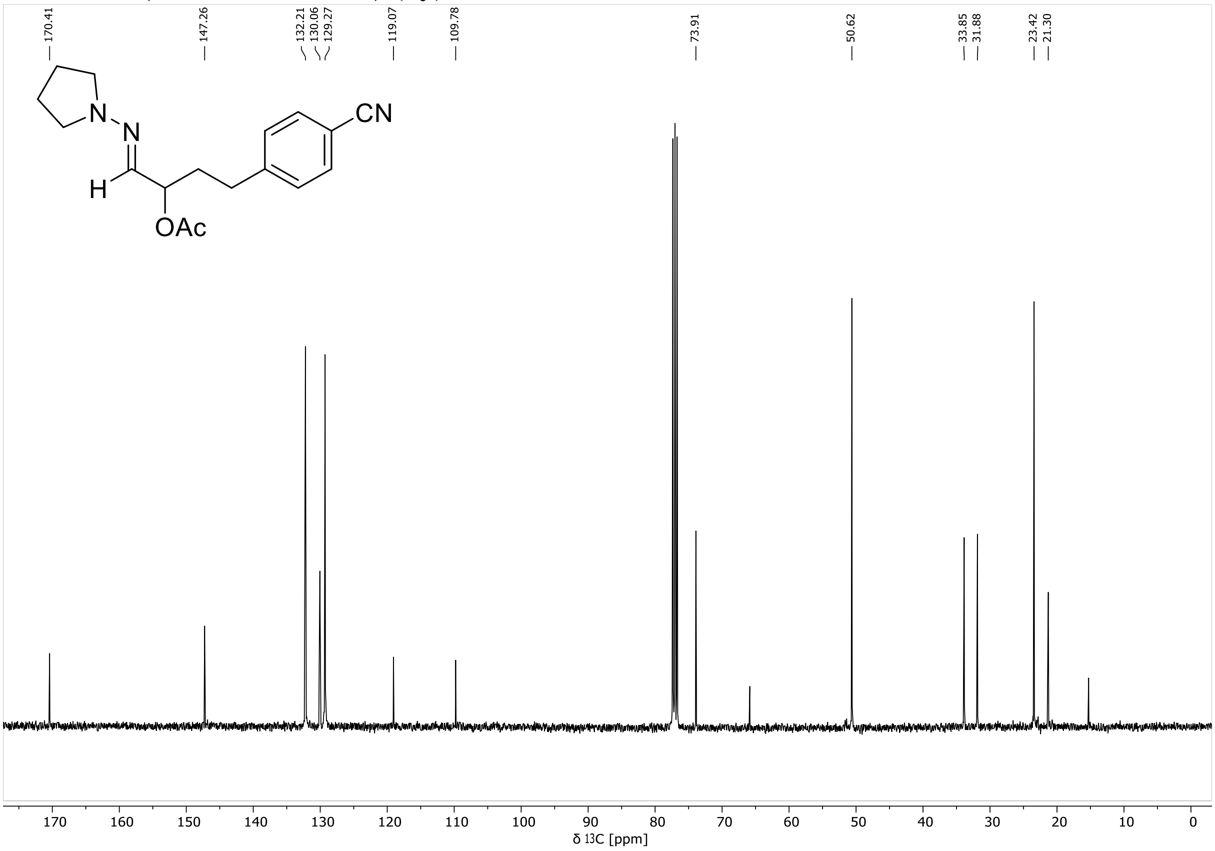

**Products:**

**$^{1}$H NMR** (400 MHz, $CDCl_3$) (**4a**):

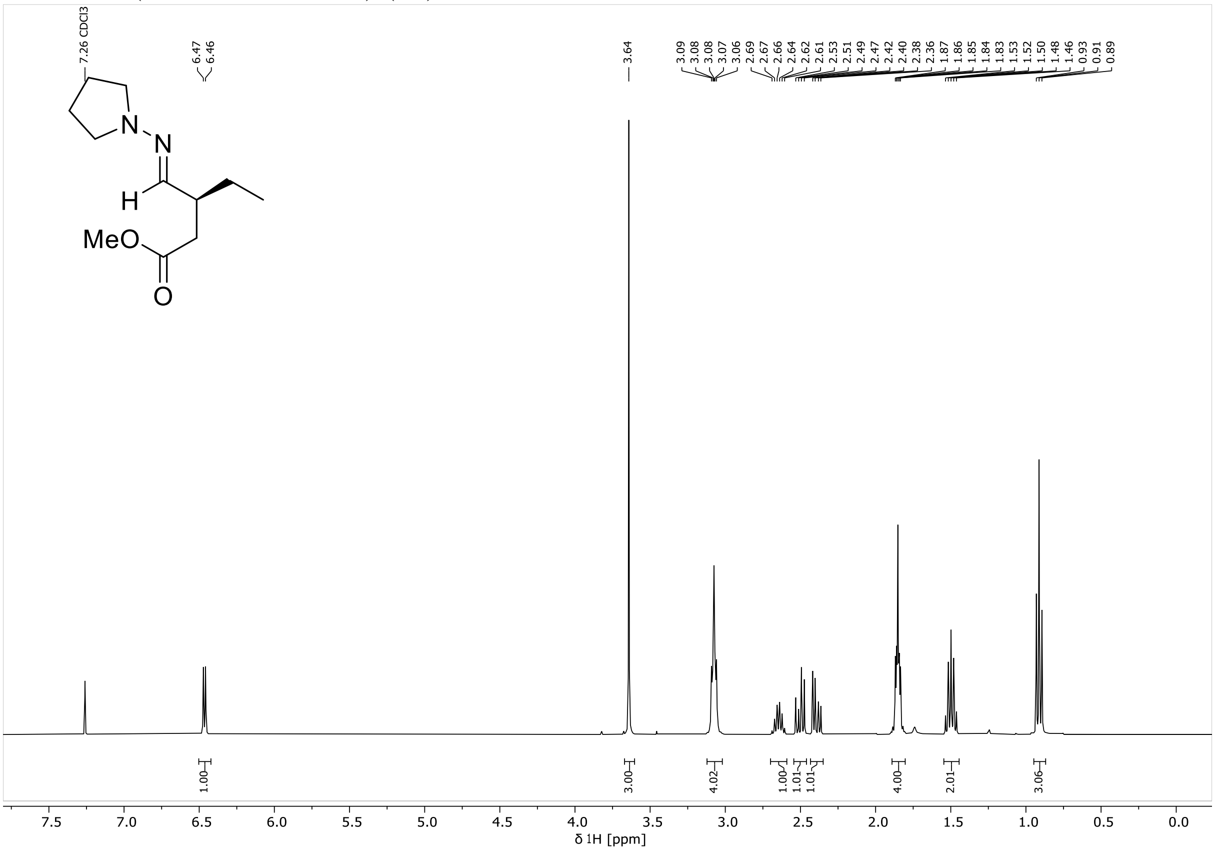

**$^{13}$C NMR** (101 MHz, $CDCl_3$) (**4a**):

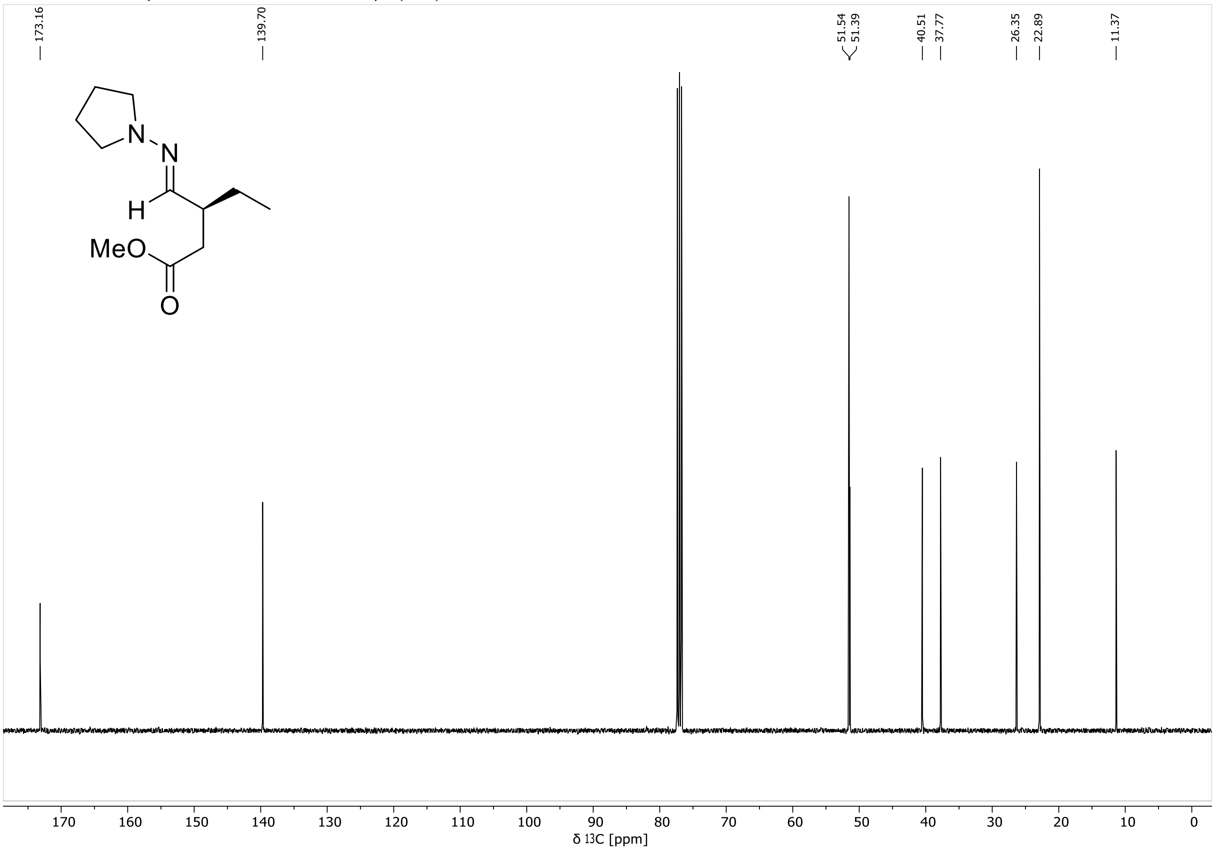

**$^{1}$H NMR** (400 MHz, $CDCl_3$) (**4b**):

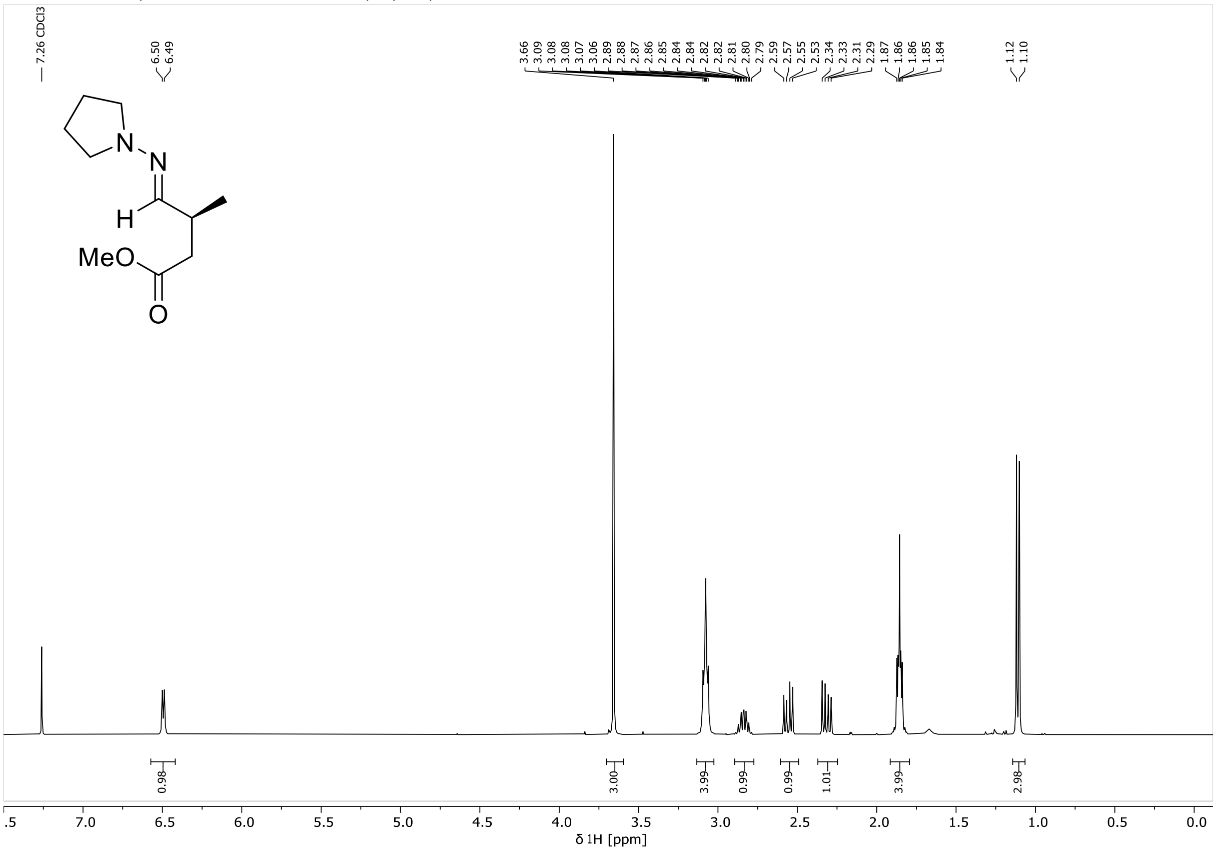

**$^{13}$C NMR** (101 MHz, $CDCl_3$) (**4b**):

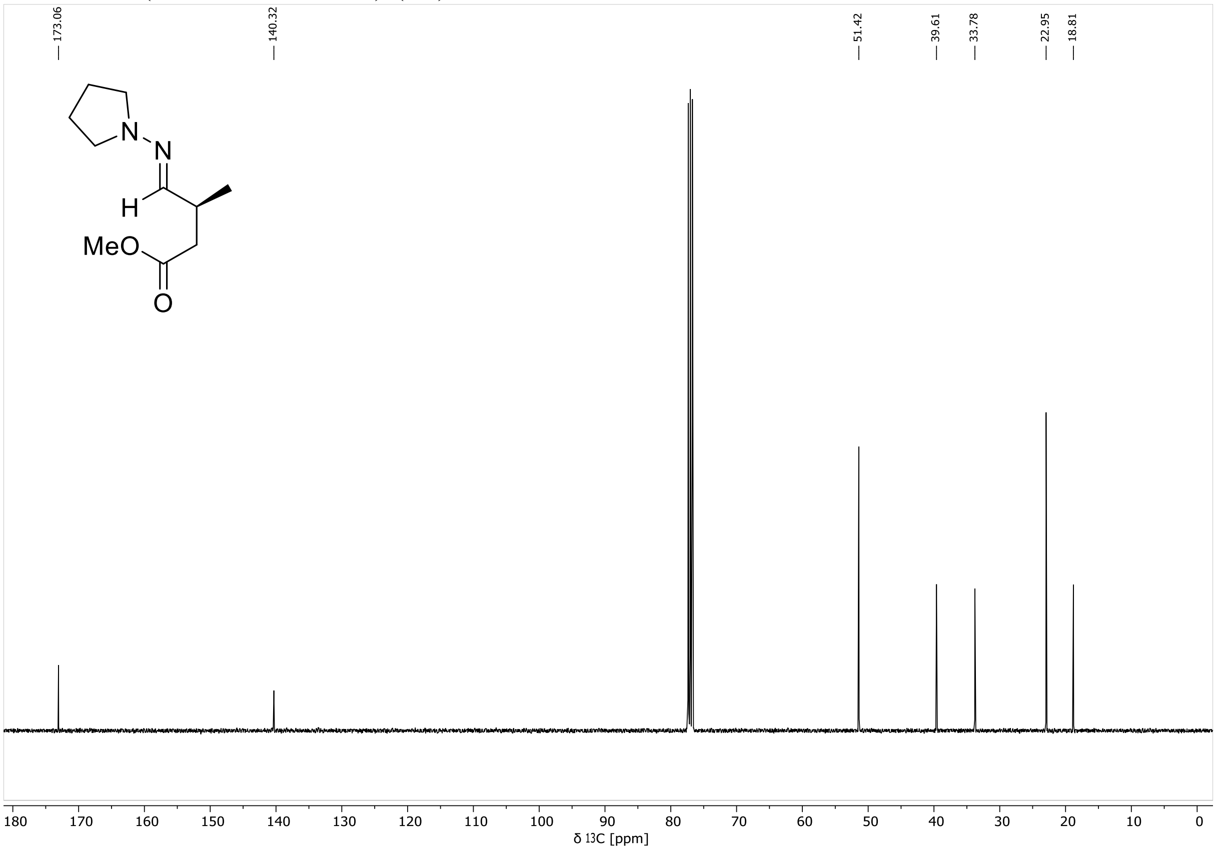

**$^{1}$H NMR** (400 MHz, $CDCl_3$) (**4c**):

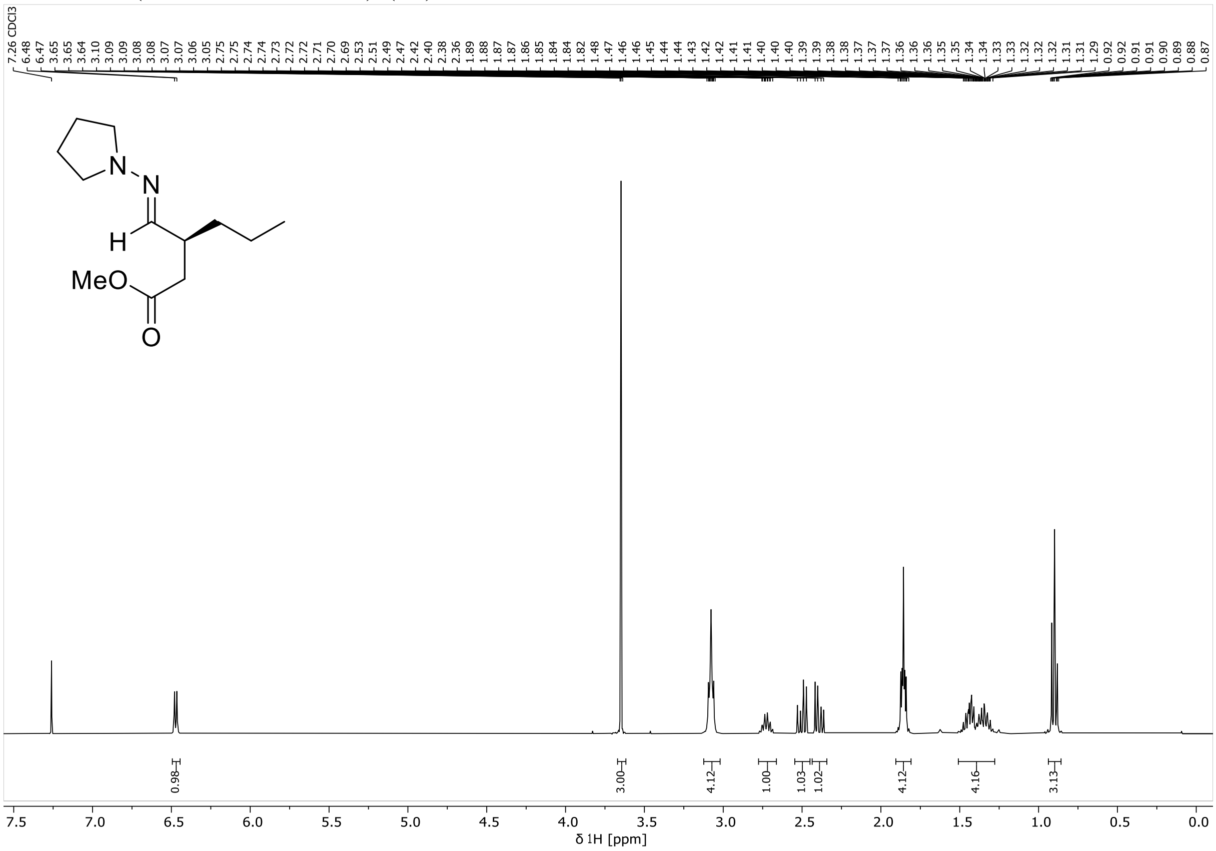


**$^{13}$C NMR** (101 MHz, $CDCl_3$) (**4c**):

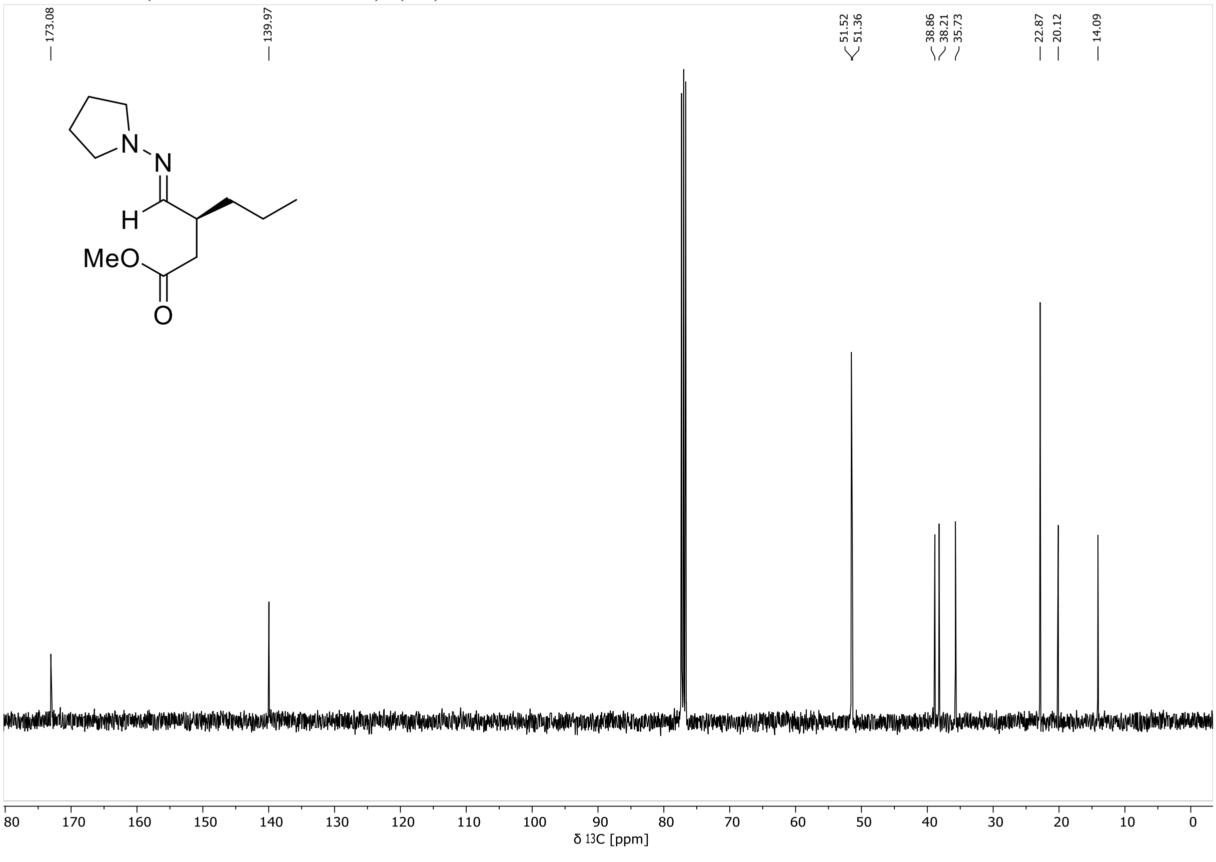

**¹H NMR** (400 MHz, CDCl₃) (**4d**):

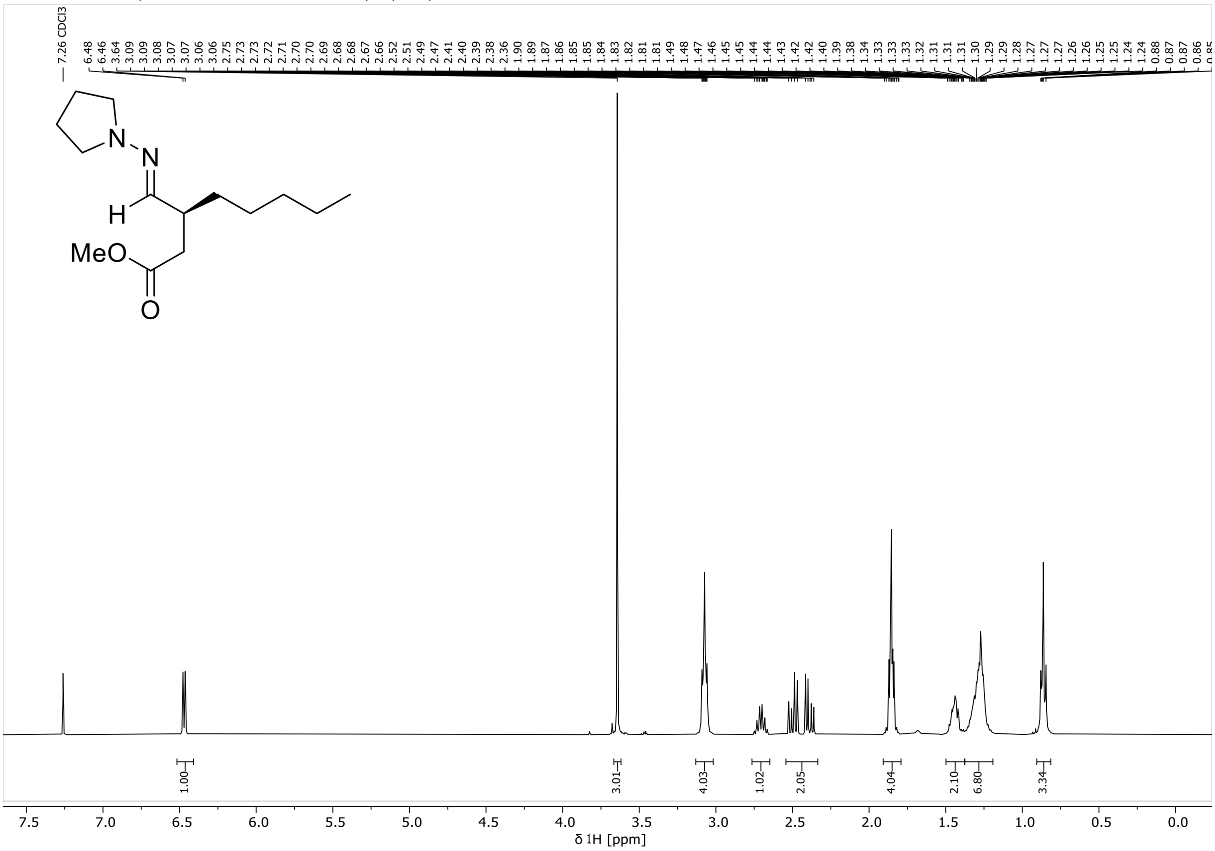

**¹³C NMR** (101 MHz, CDCl₃) (**4d**):

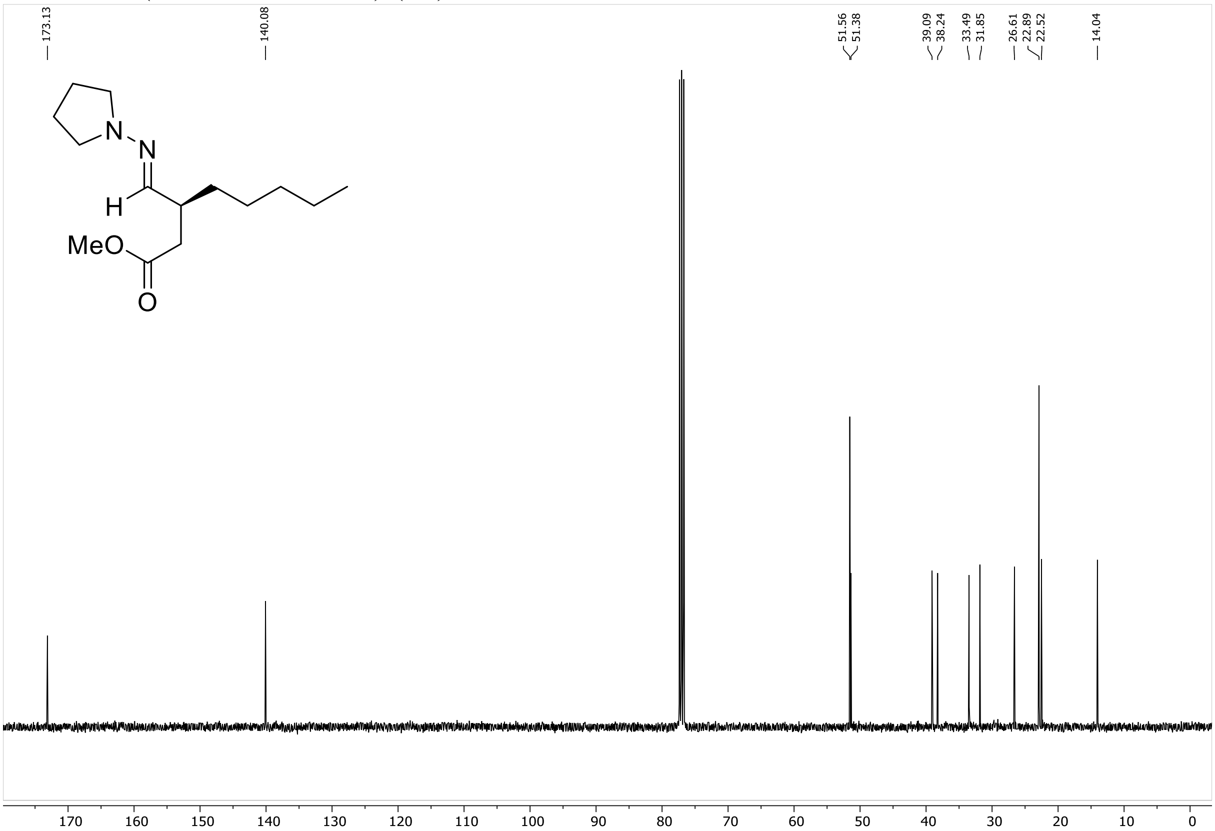

**$^{1}$H NMR** (400 MHz, $CDCl_3$) (**4e**):

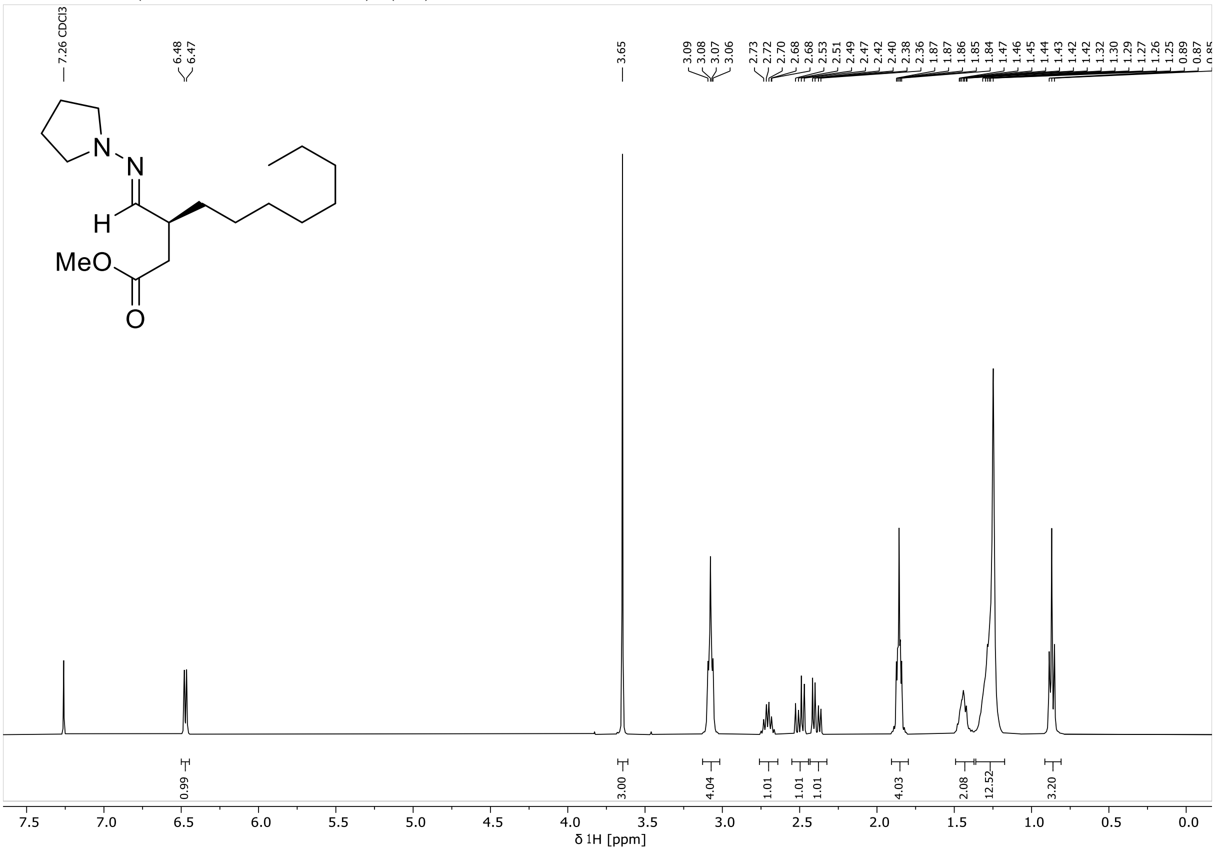

**$^{13}$C NMR** (101 MHz, $CDCl_3$) (**4e**):

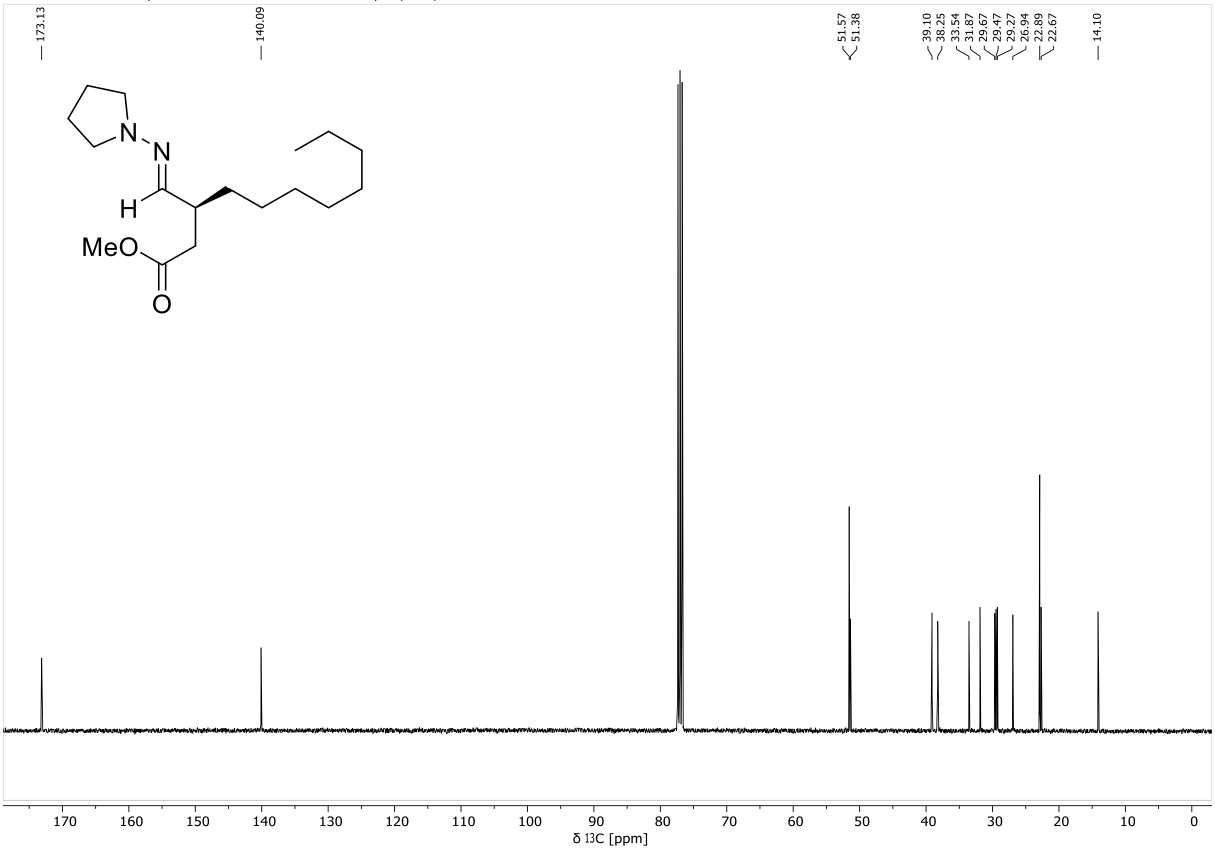

**$^{1}$H NMR** (400 MHz, $CDCl_3$) (**4f**):

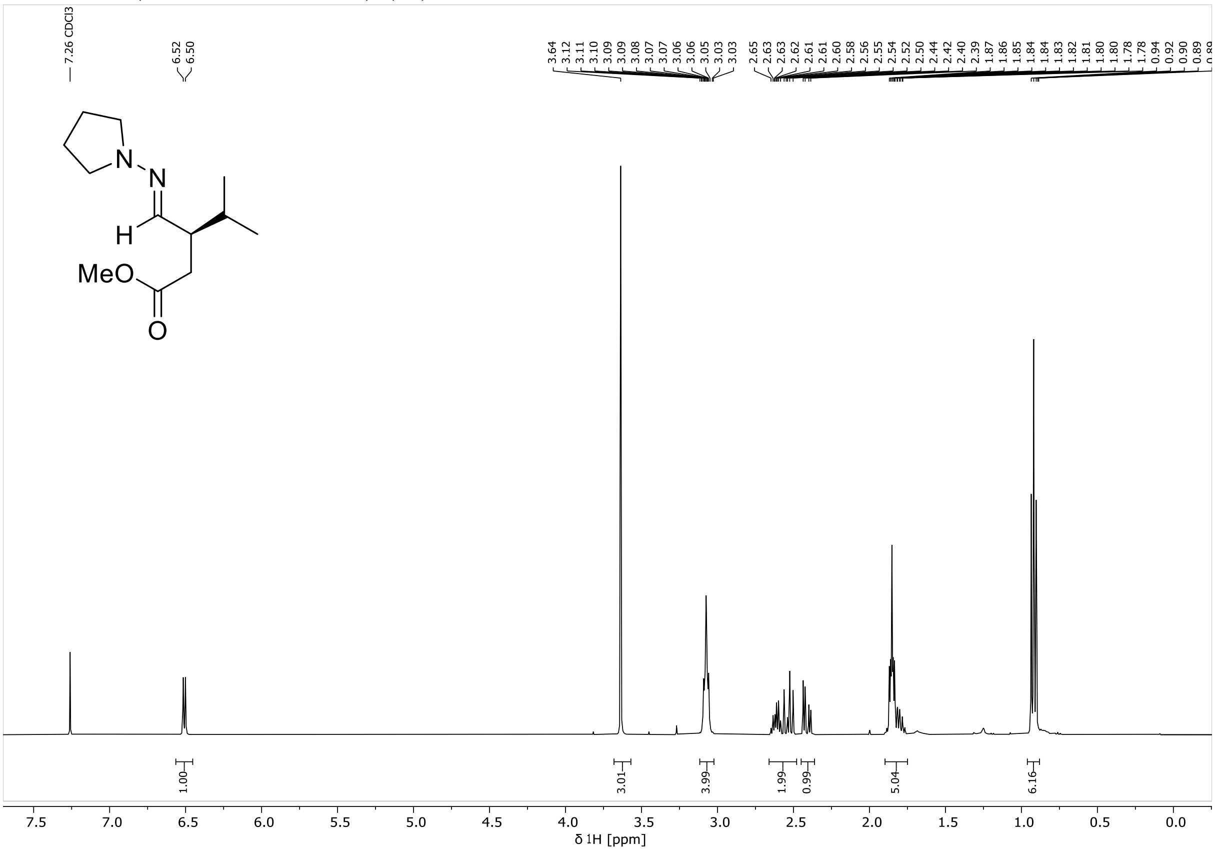


**$^{13}$C NMR** (101 MHz, $CDCl_3$) (**4f**):

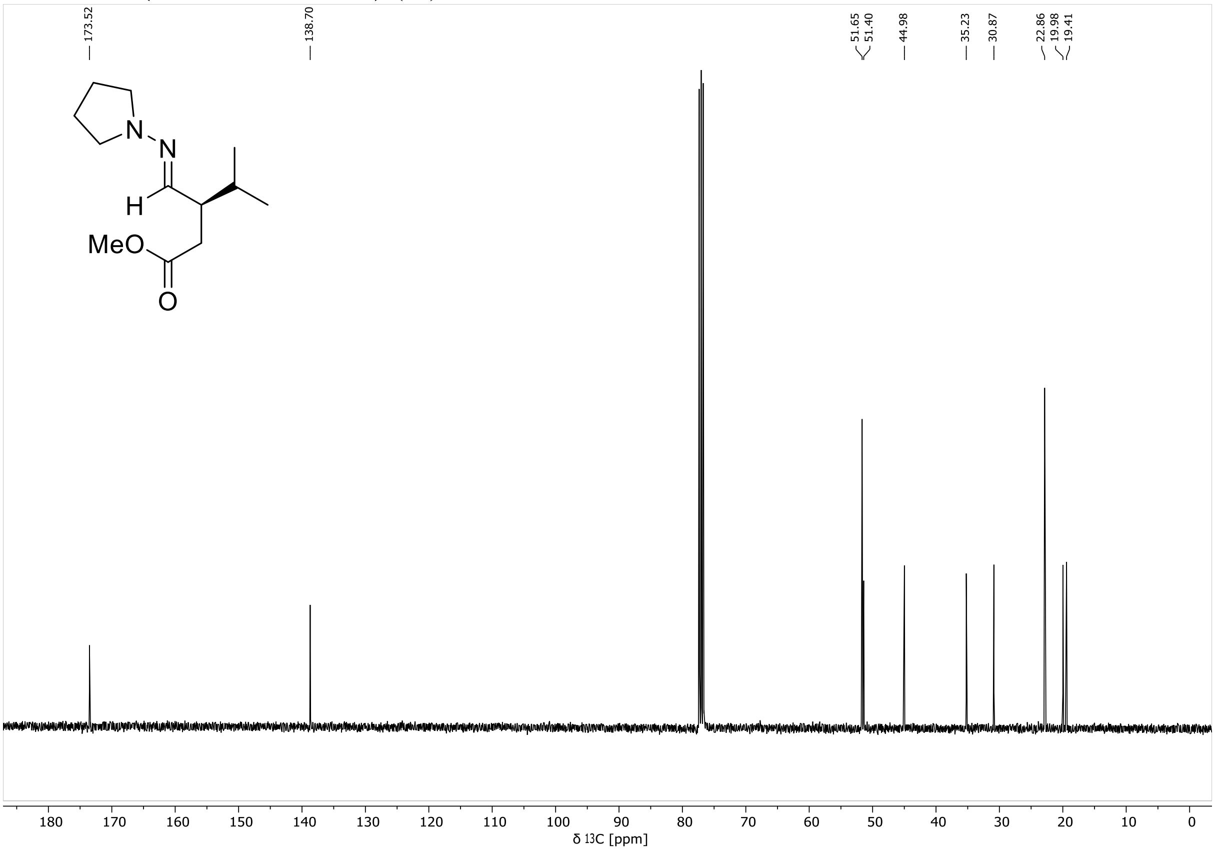

**$^{1}$H NMR** (400 MHz, $CDCl_3$) (**4g**):

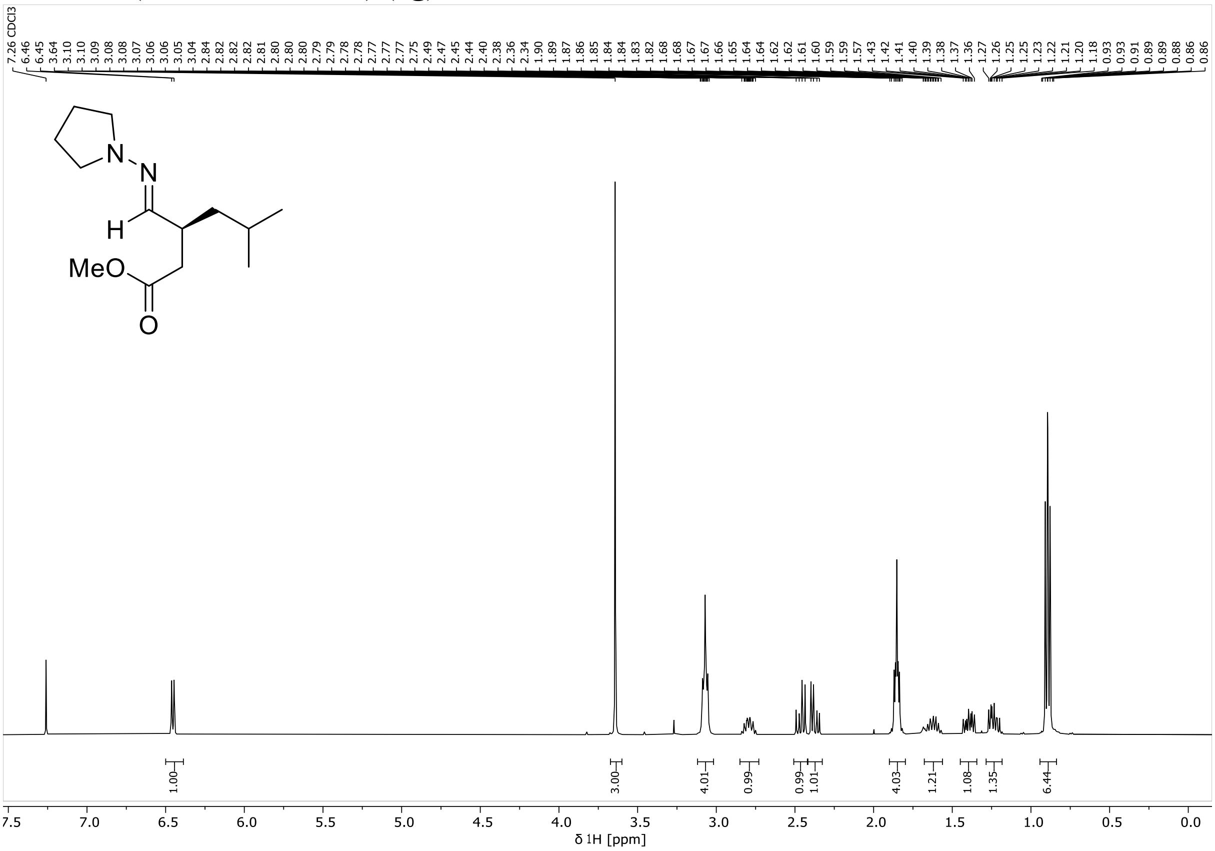


**$^{13}$C NMR** (101 MHz, $CDCl_3$) (**4g**):

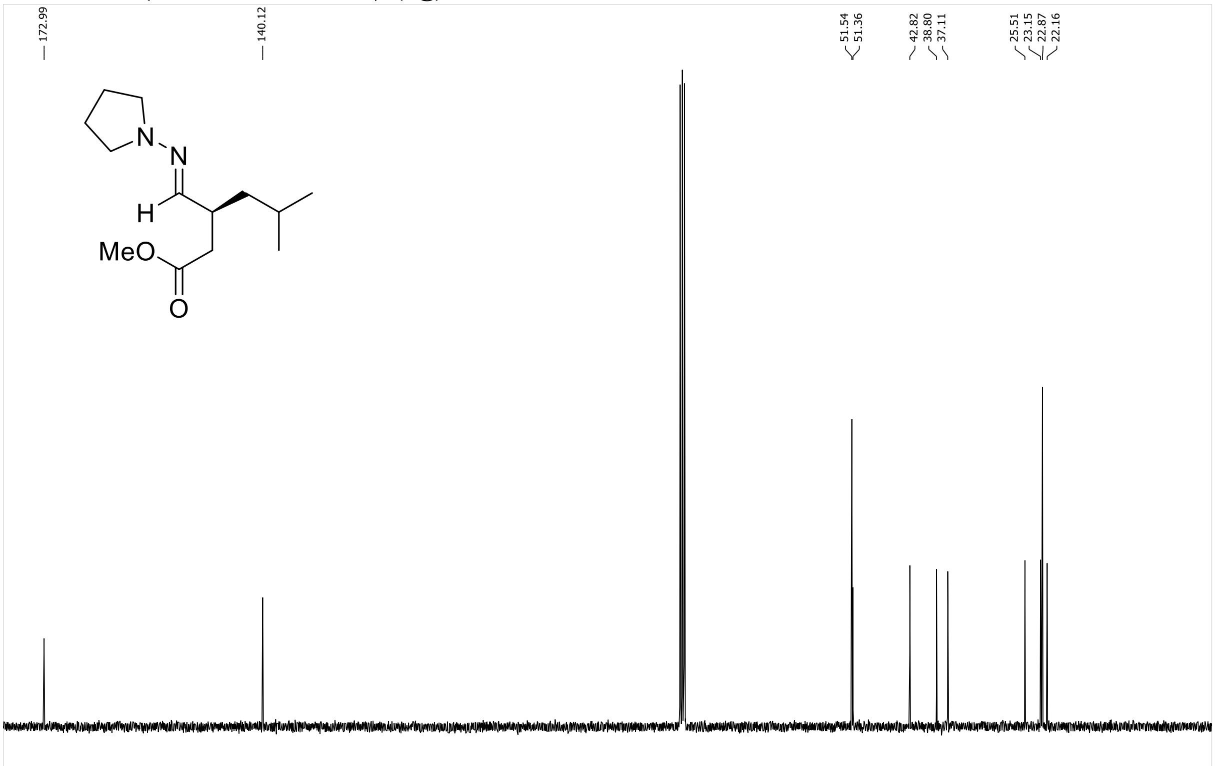

**$^{1}$H NMR** (400 MHz, $CDCl_3$) (**4h**):

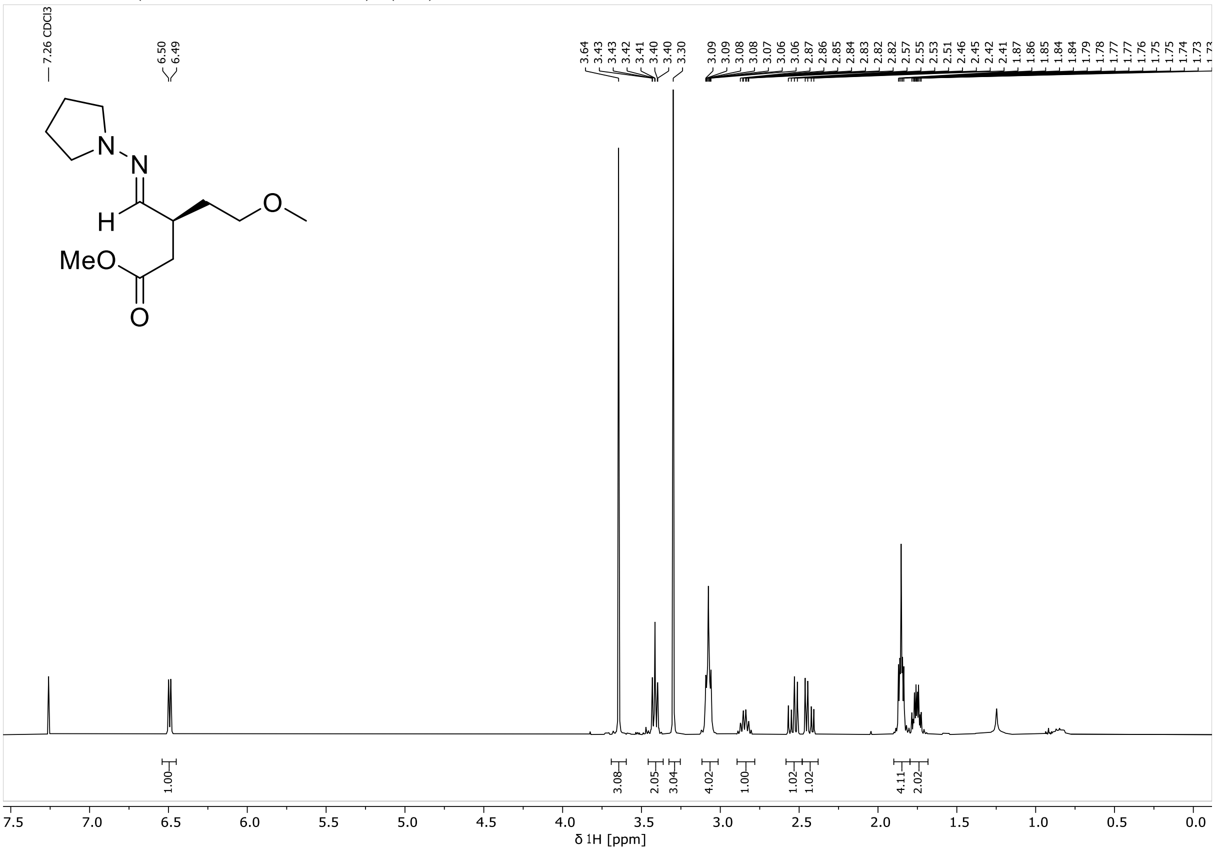

**$^{13}$C NMR** (101 MHz, $CDCl_3$) (**4h**):

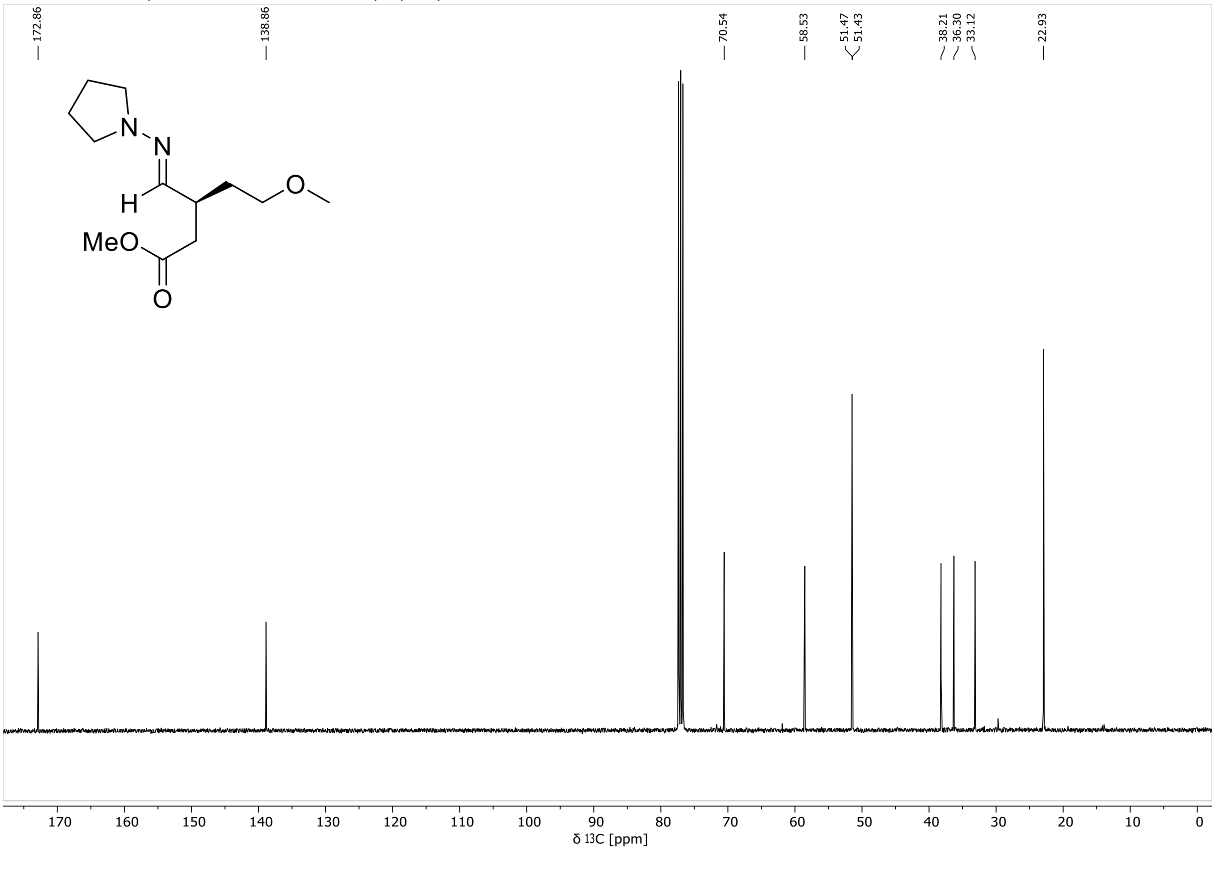

**$^{1}$H NMR** (400 MHz, $CDCl_3$) (**4i**):

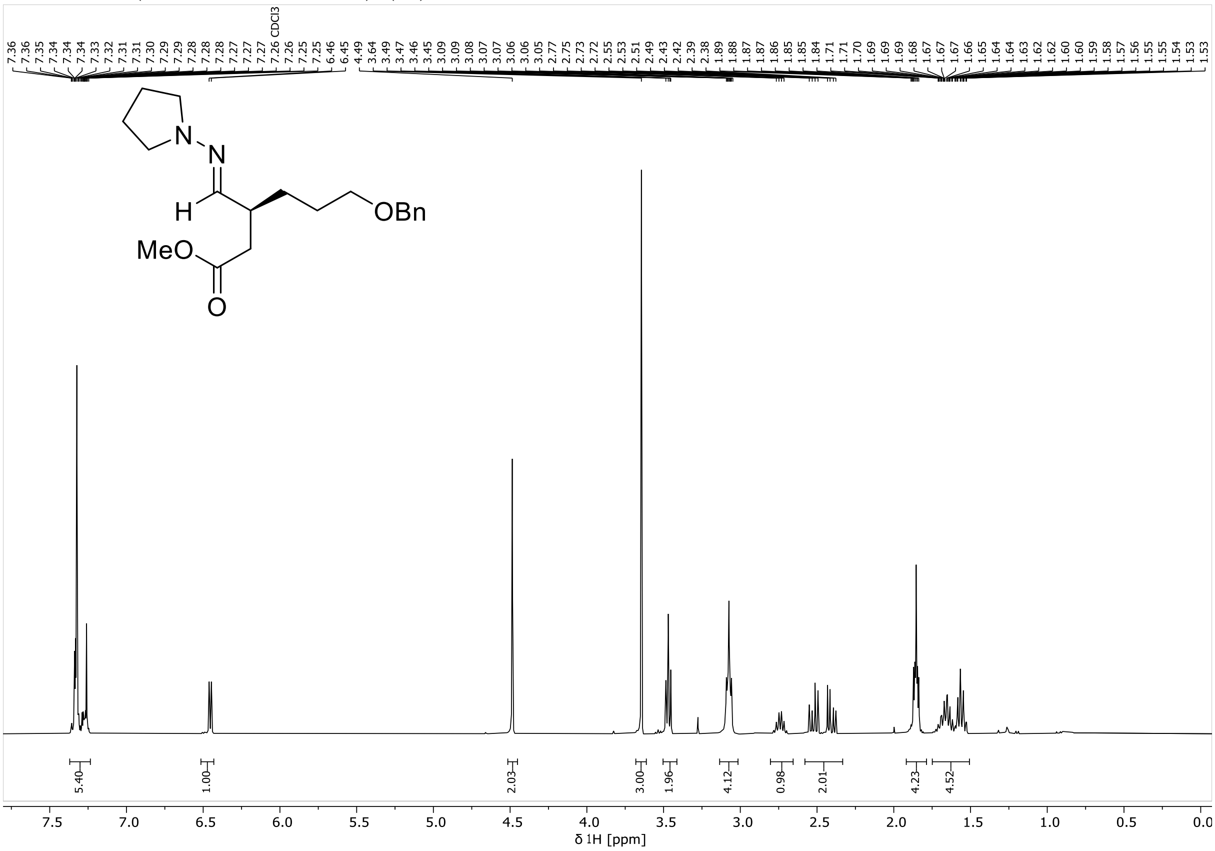

**$^{13}$C NMR** (101 MHz, $CDCl_3$) (**4i**):

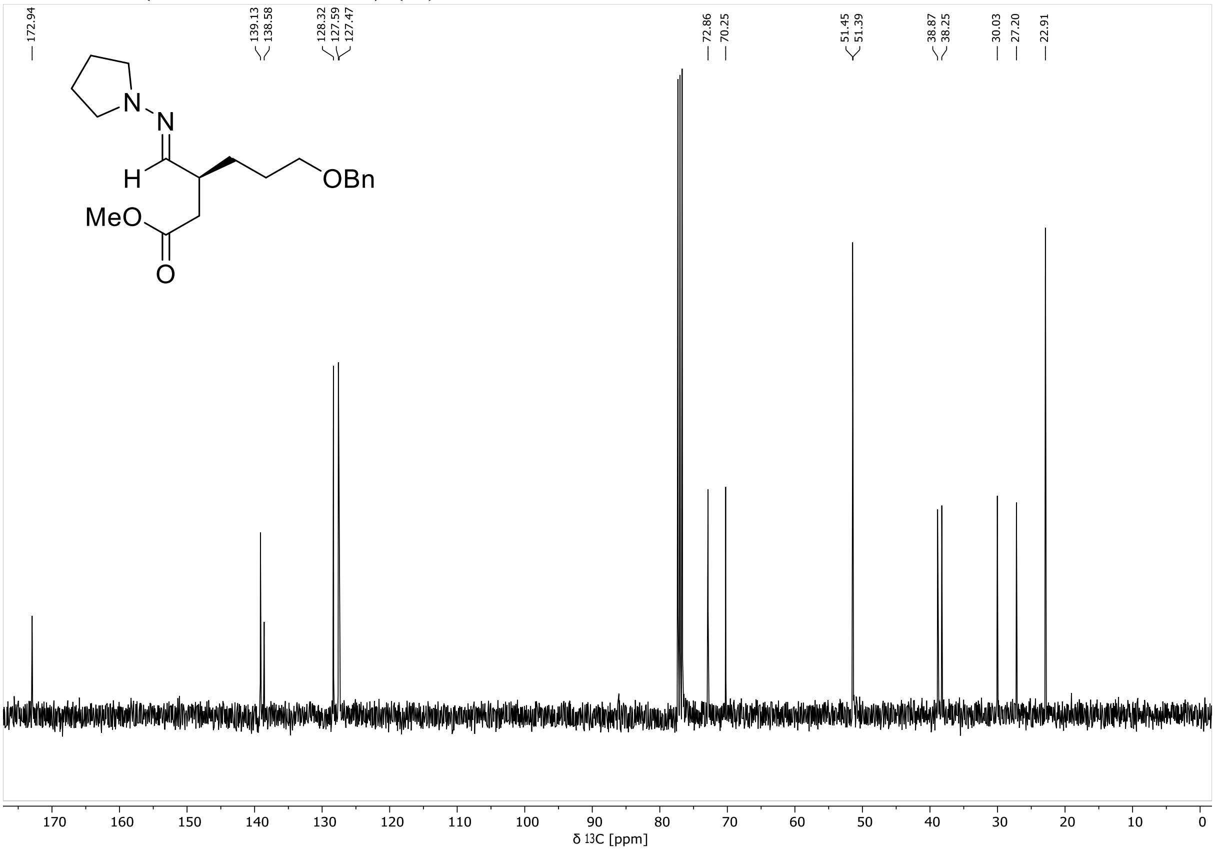

**$^{1}$H NMR** (400 MHz, $CDCl_3$) (**4j**):

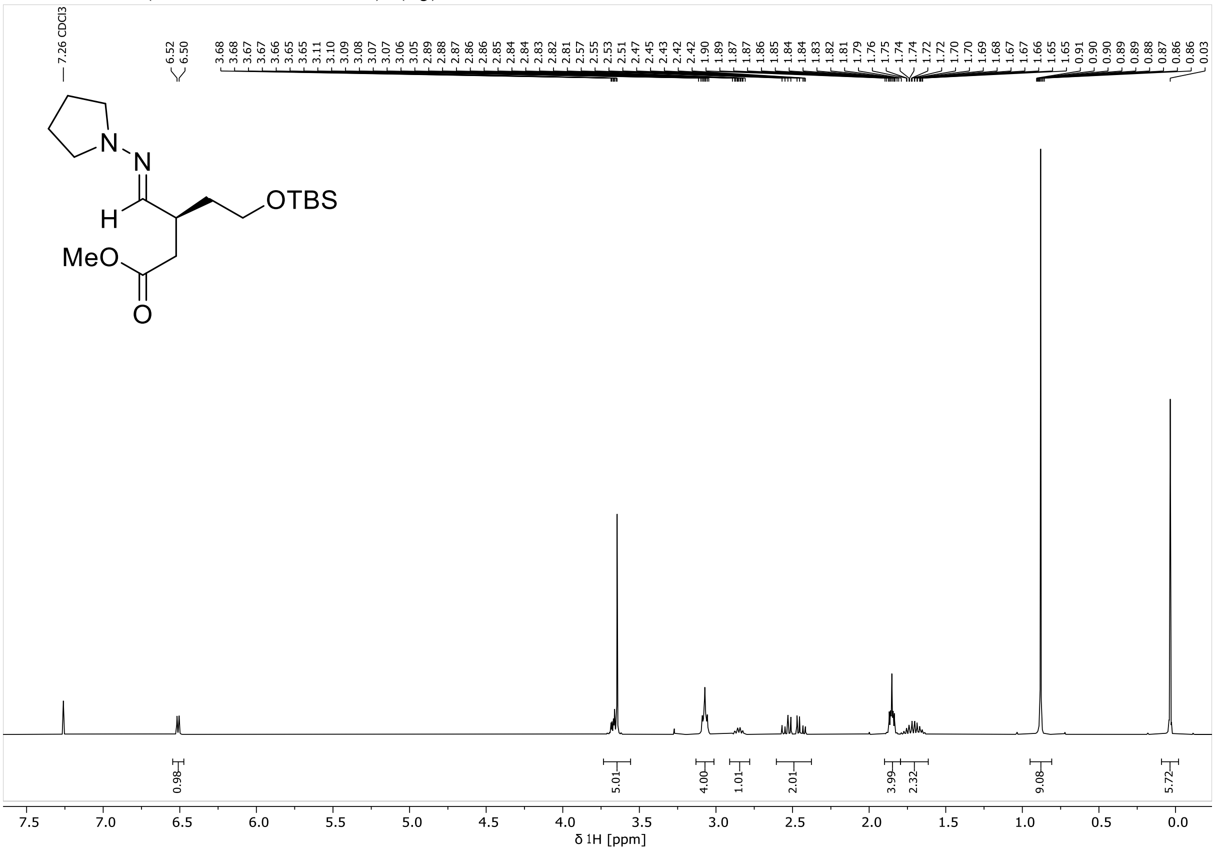

**$^{13}$C NMR** (101 MHz, $CDCl_3$) (**4j**):

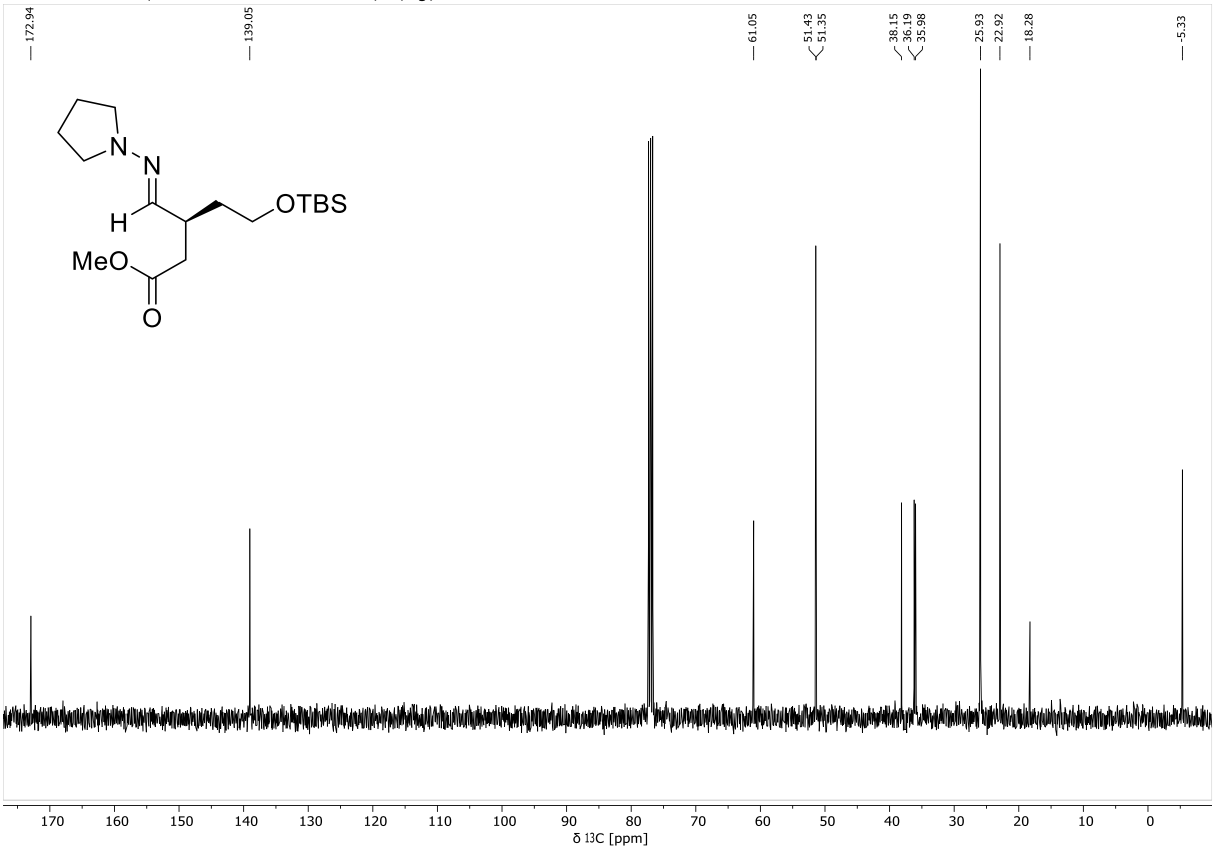

**$^{1}$H NMR** (400 MHz, $CDCl_3$) (**4k**):

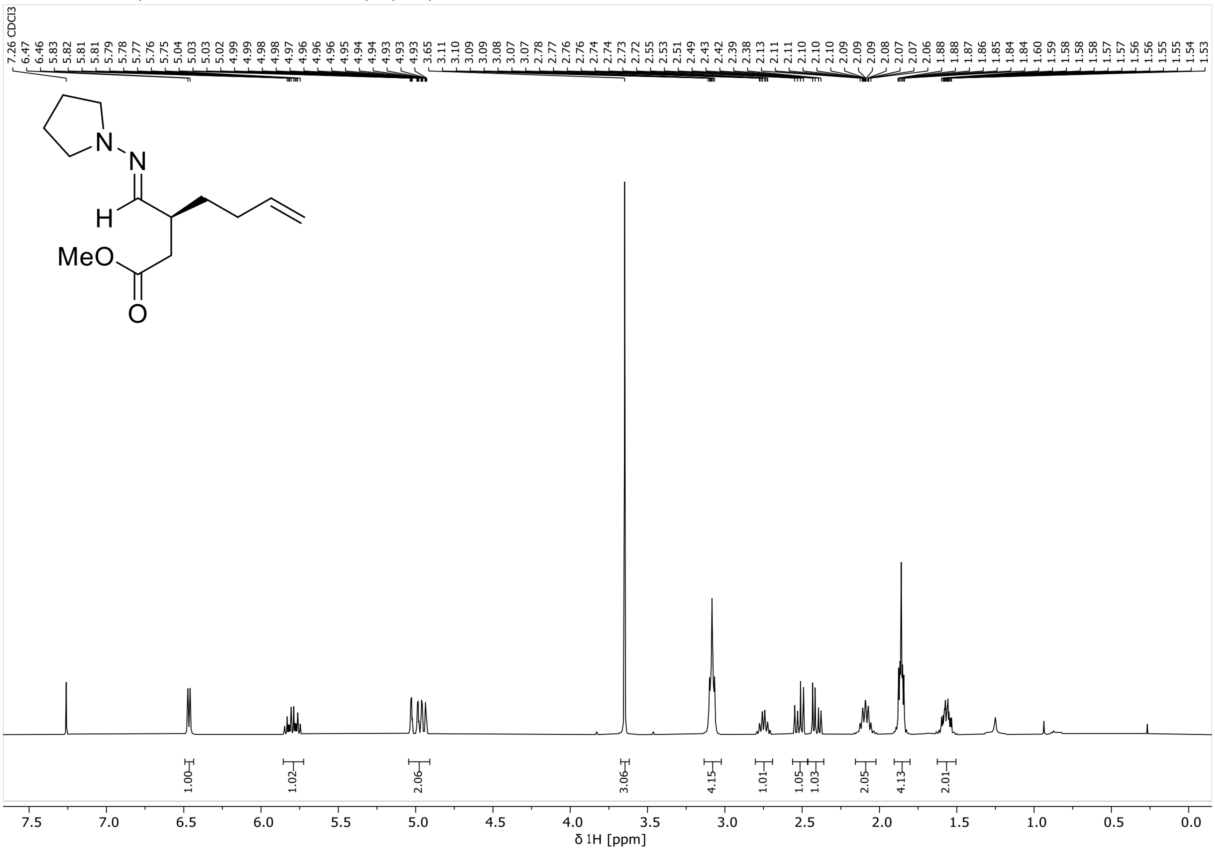


**$^{13}$C NMR** (101 MHz, $CDCl_3$) (**4k**):

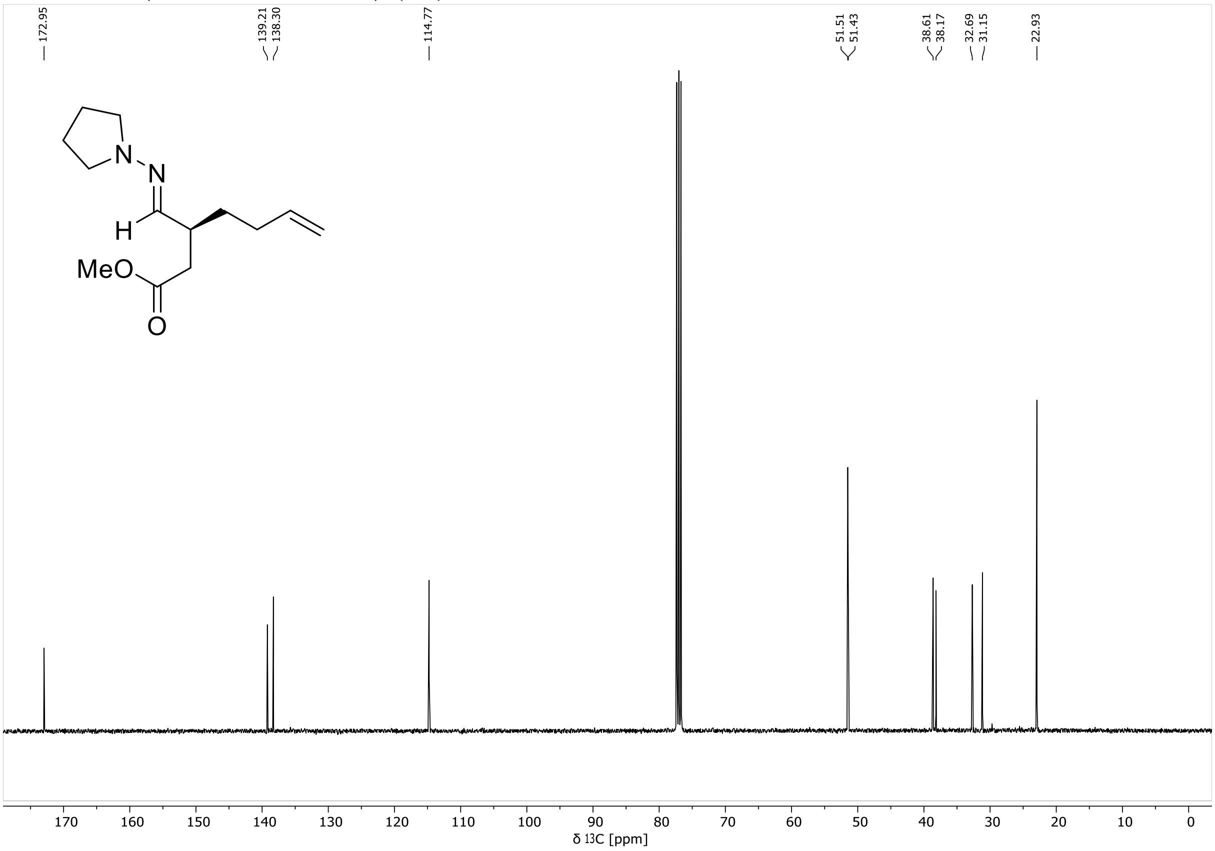

**$^{1}$H NMR** (400 MHz, CDCl$_3$) (**4l**):

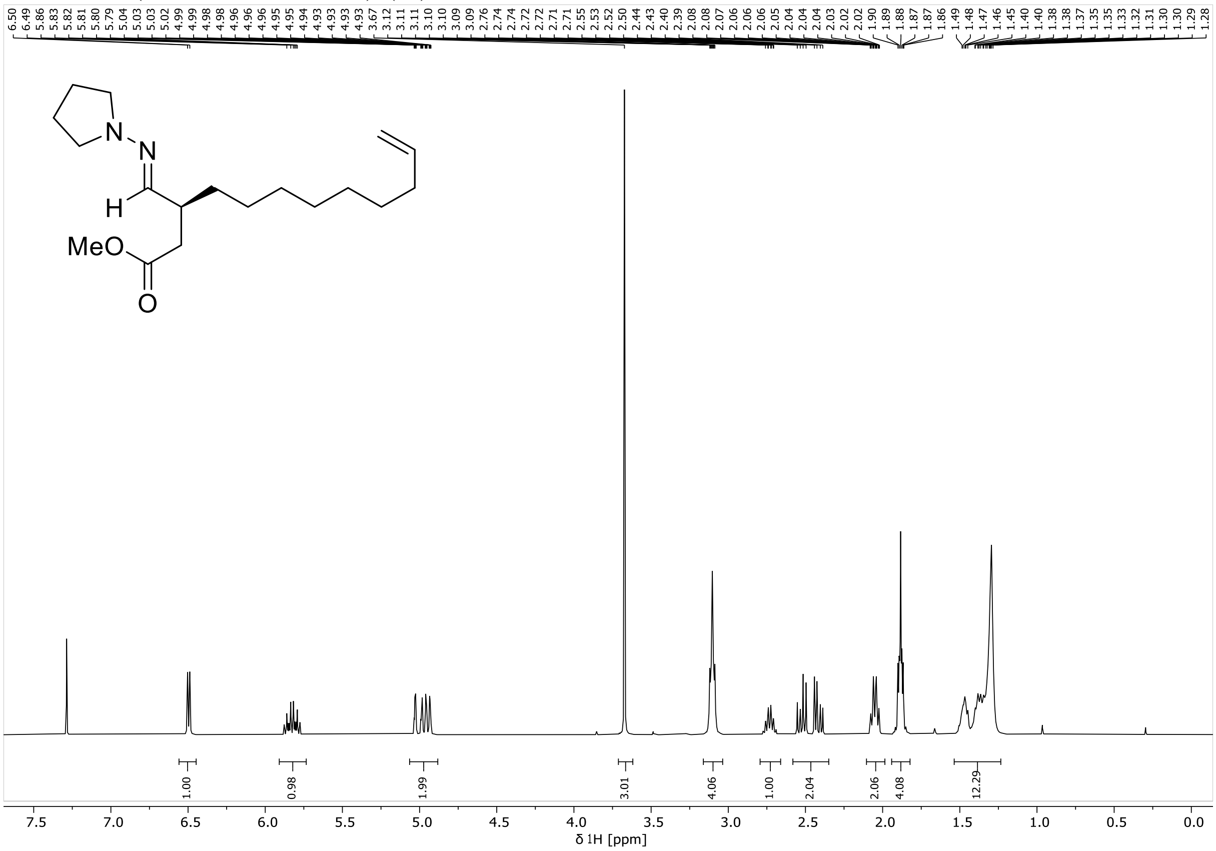


**$^{13}$C NMR** (101 MHz, CDCl$_3$) (**4l**):

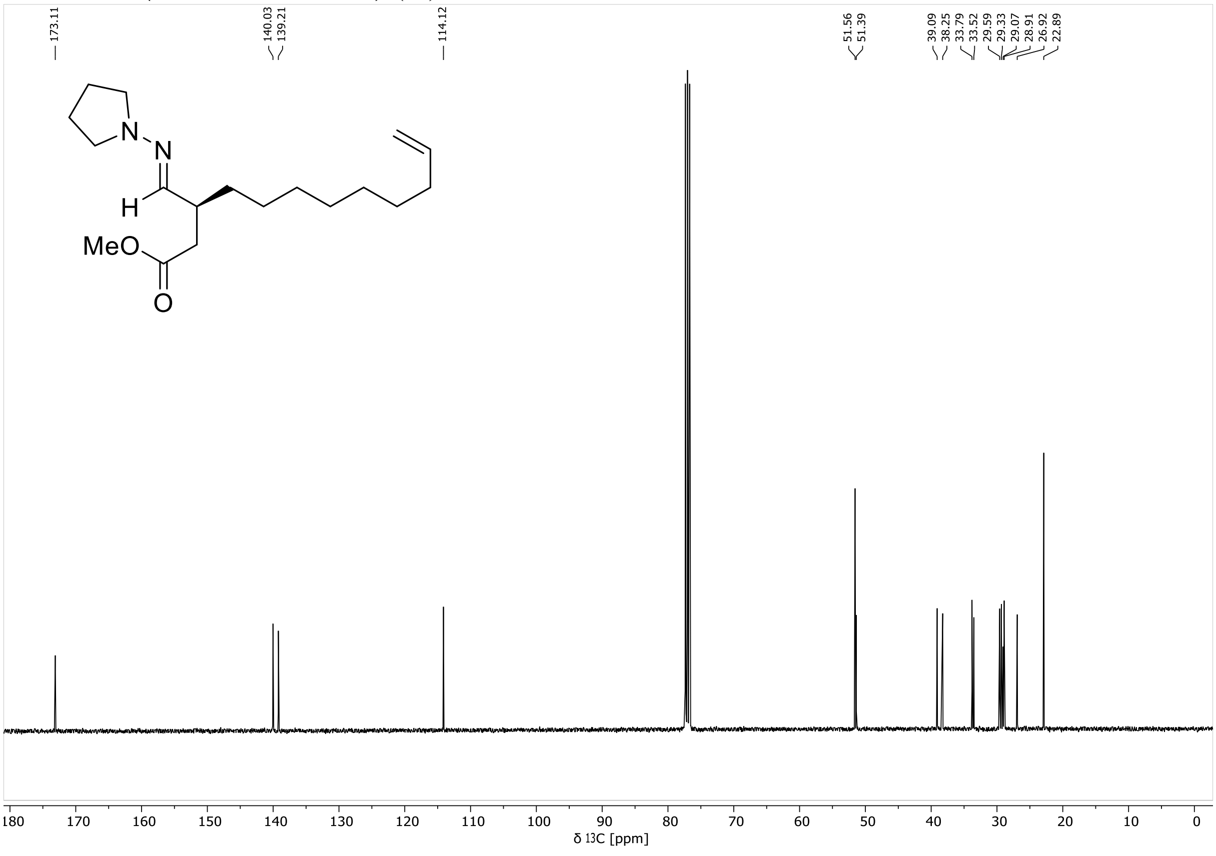

**$^{1}$H NMR** (400 MHz, $CDCl_3$) (**4m**):

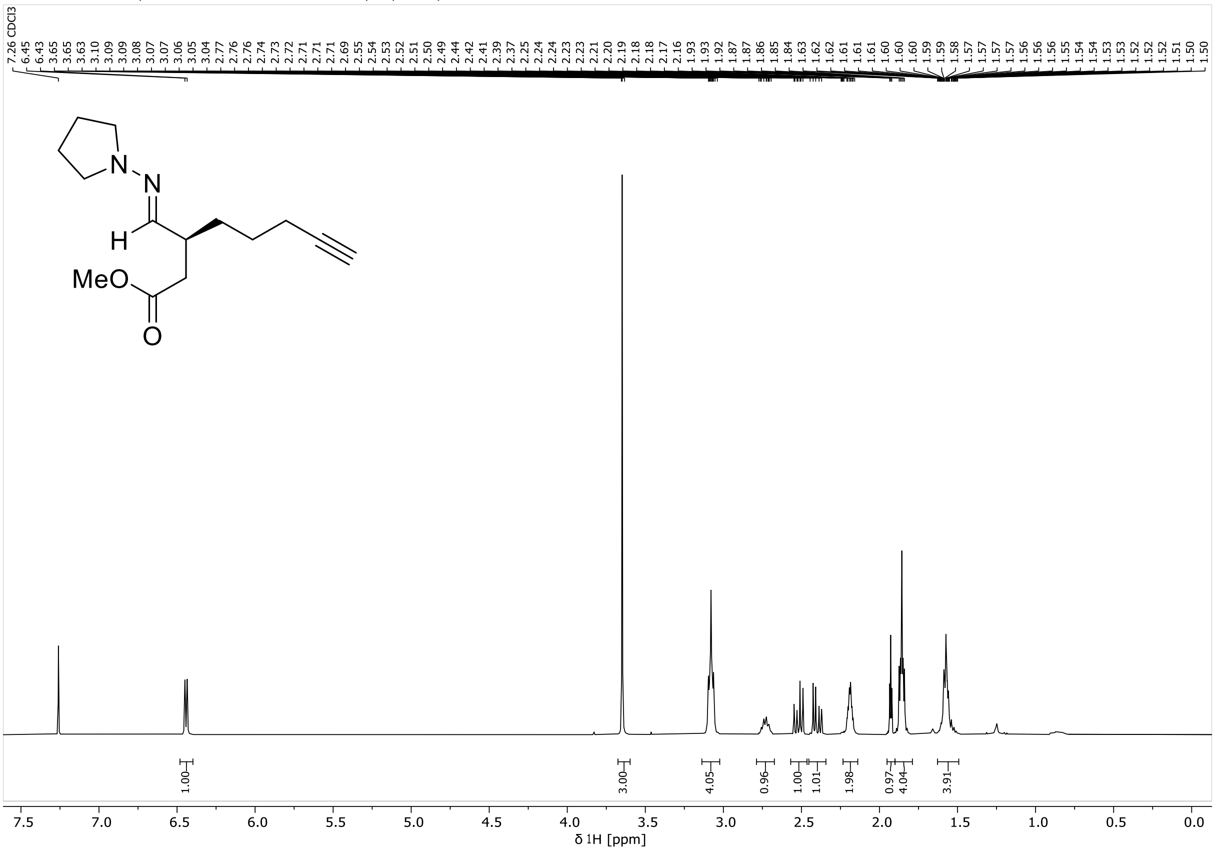


**$^{13}$C NMR** (101 MHz, $CDCl_3$) (**4m**):

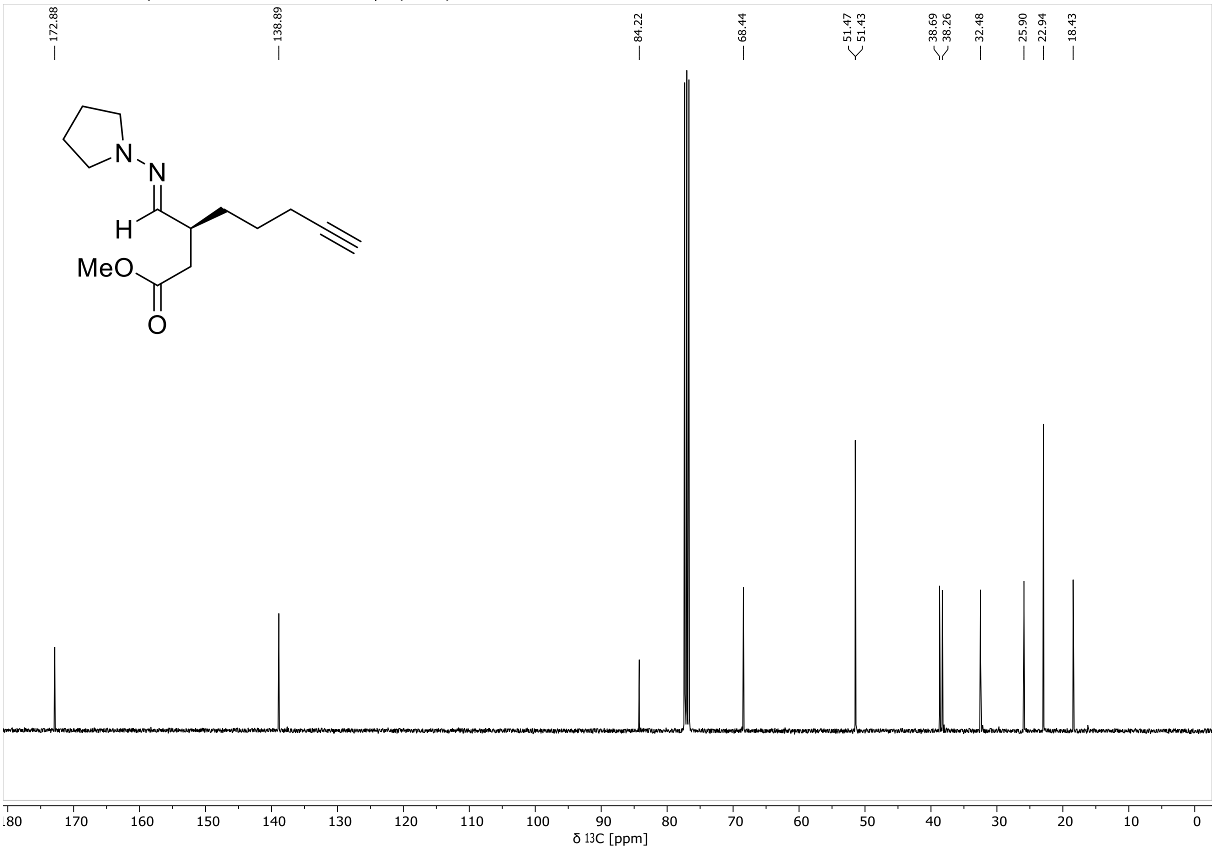

**$^{1}$H NMR** (400 MHz, $CDCl_3$) (**4n**):

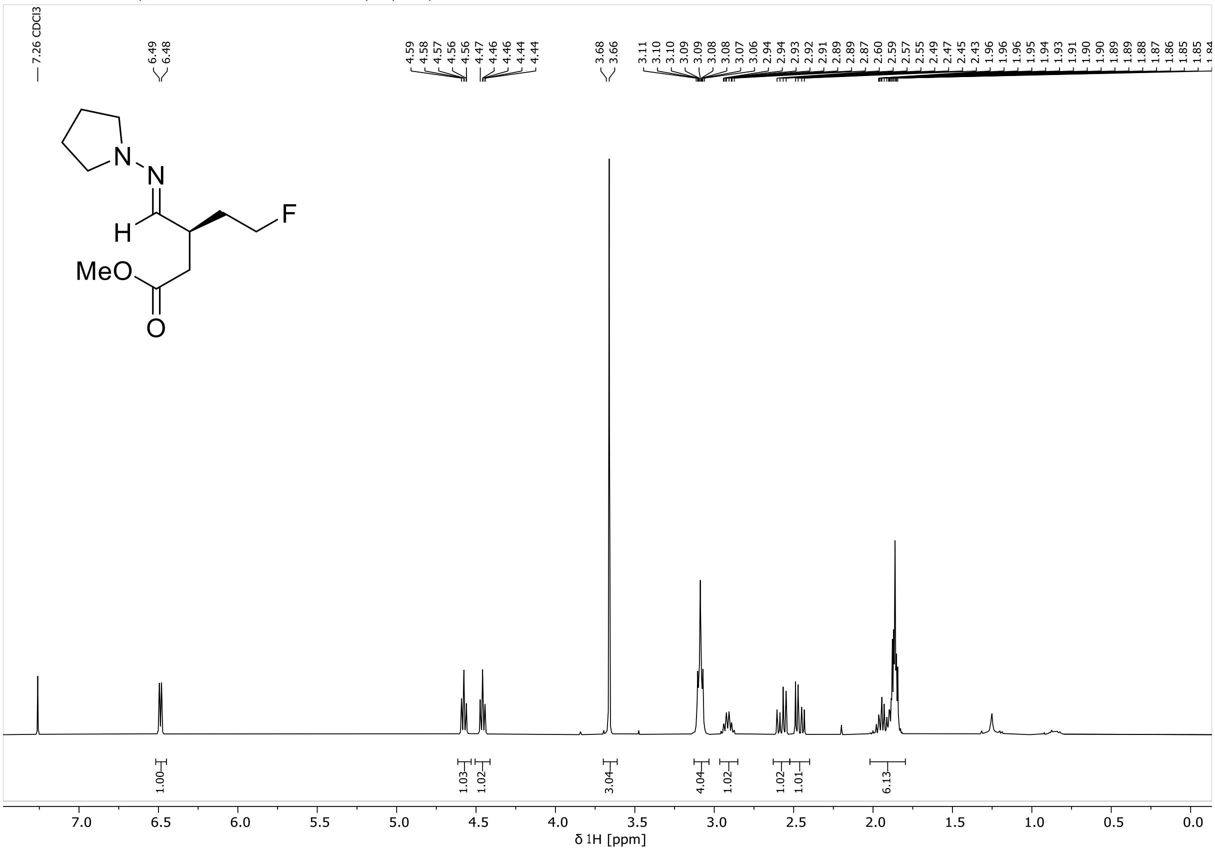

**$^{13}$C NMR** (101 MHz, $CDCl_3$) (**4n**):

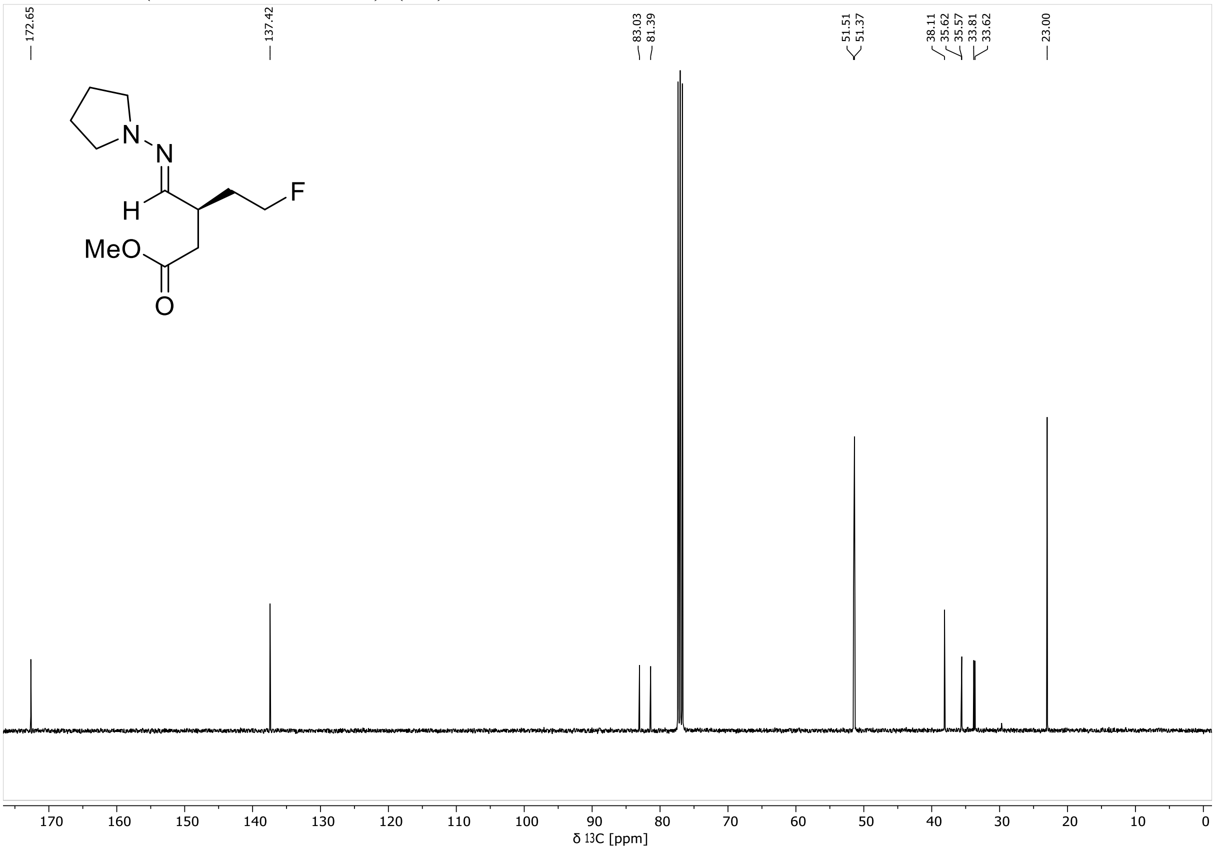

**$^{19}$F NMR** (377 MHz, $CDCl_3$) (**4n**):

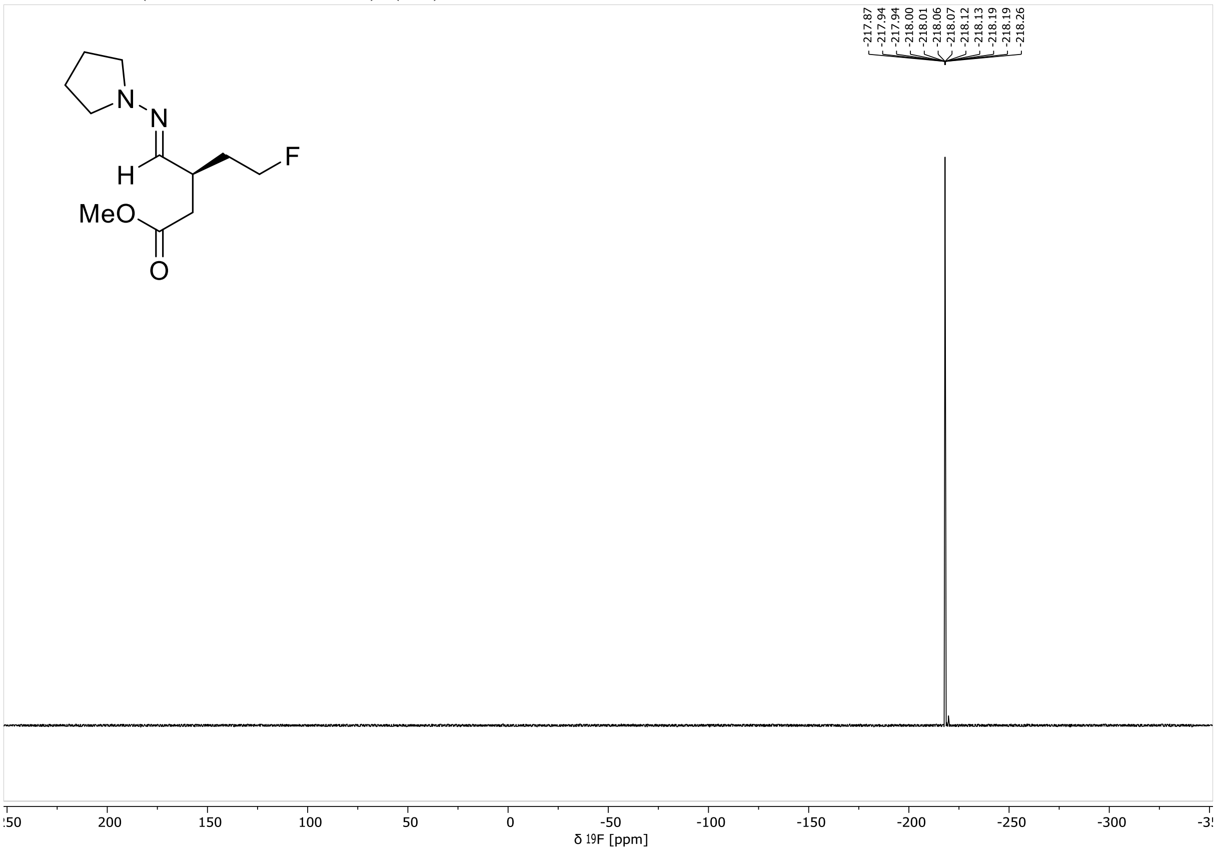


**$^{1}$H NMR** (400 MHz, $CDCl_3$) (**4o**):

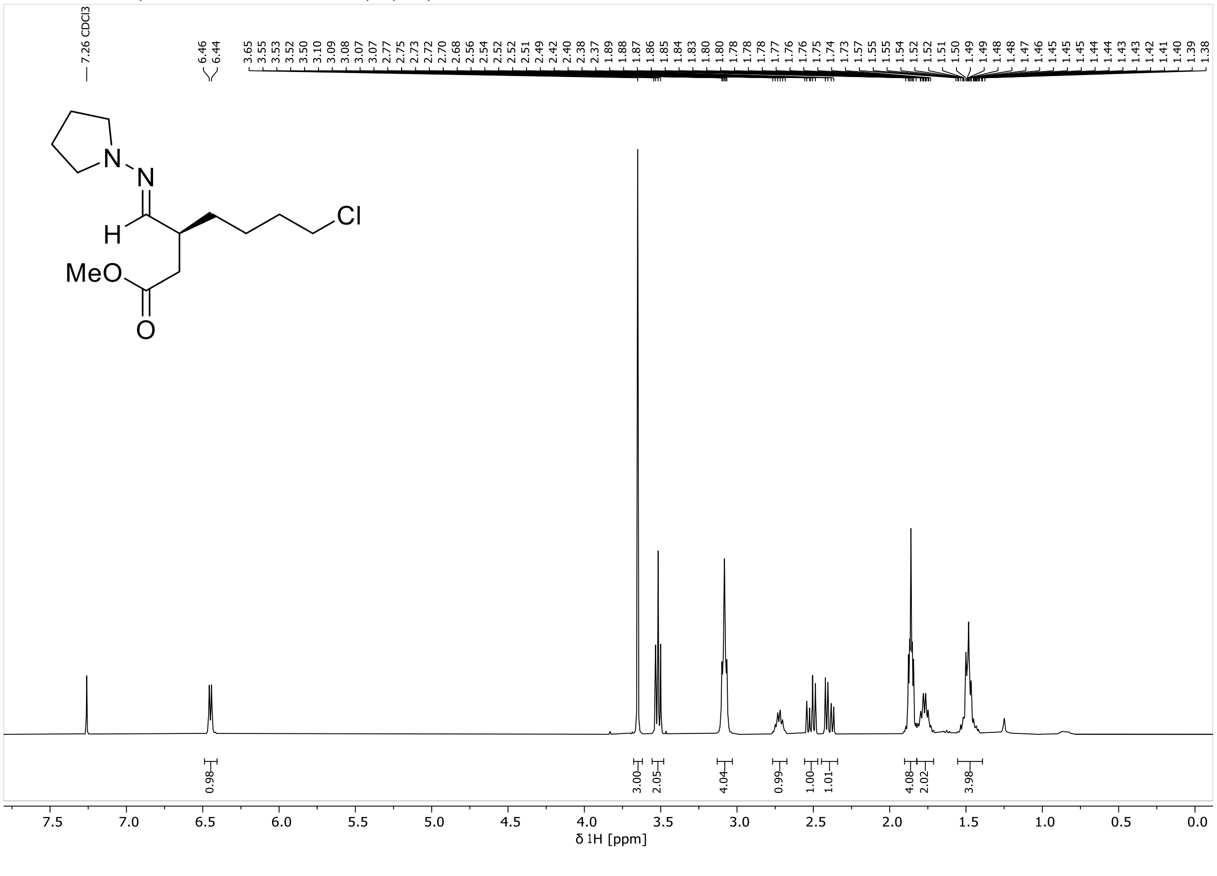

**$^{13}$C NMR** (101 MHz, $CDCl_3$) (**4o**):

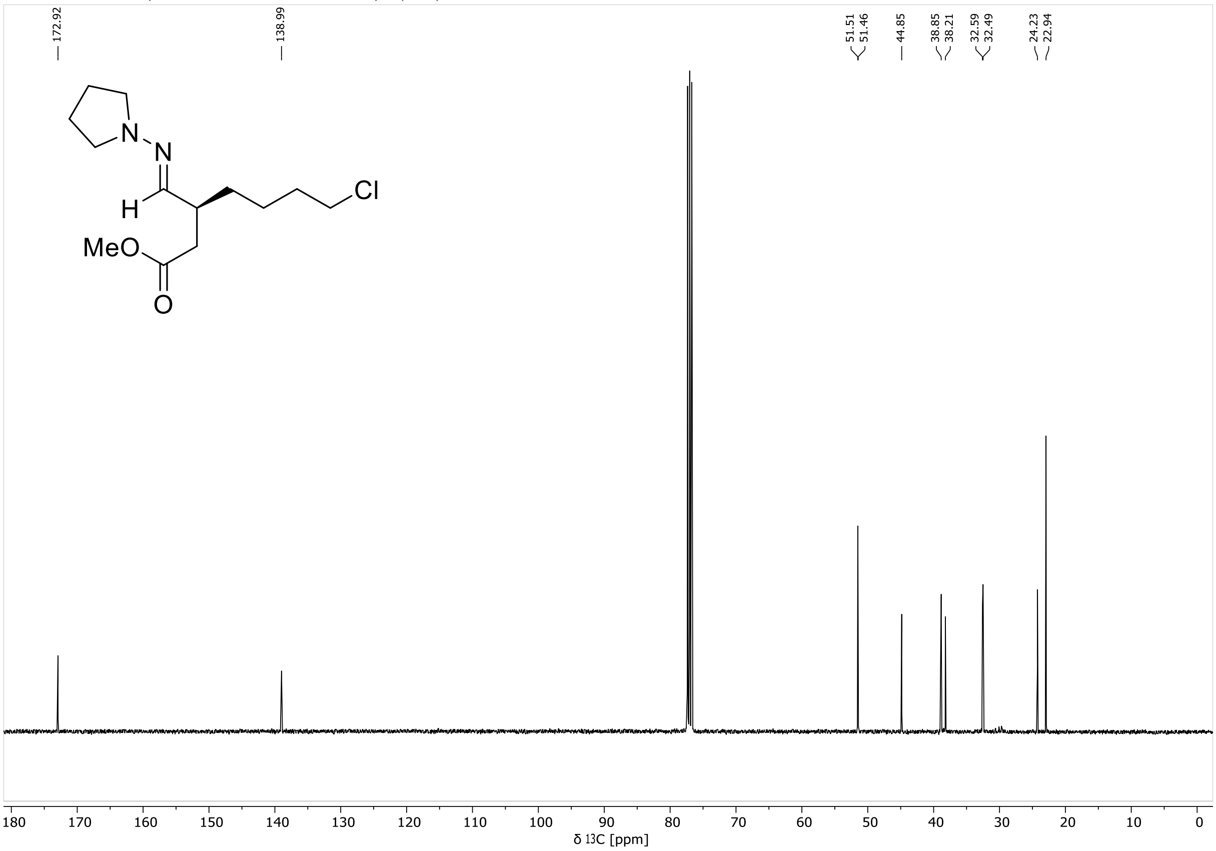


**$^{1}$H NMR** (400 MHz, $CDCl_3$) (**4p**):

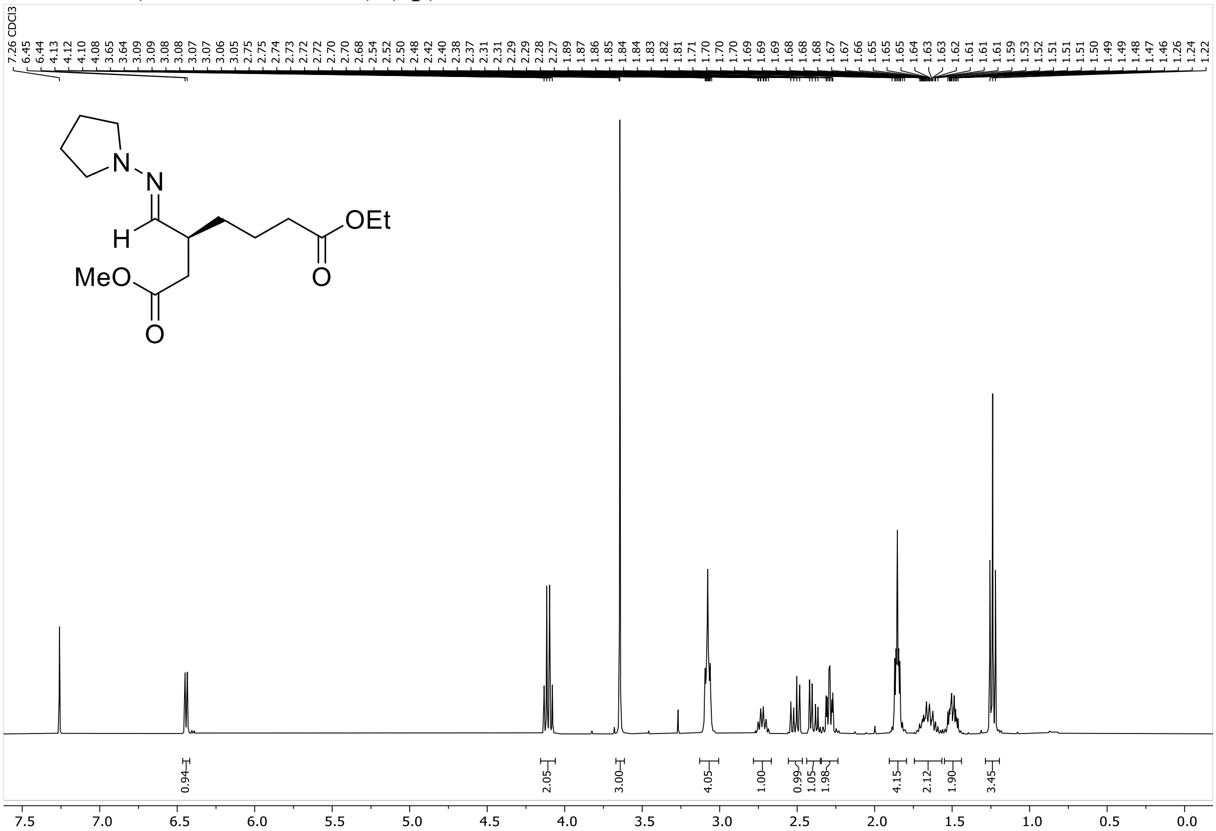

**$^{13}$C NMR** (101 MHz, $CDCl_3$) (**4p**):

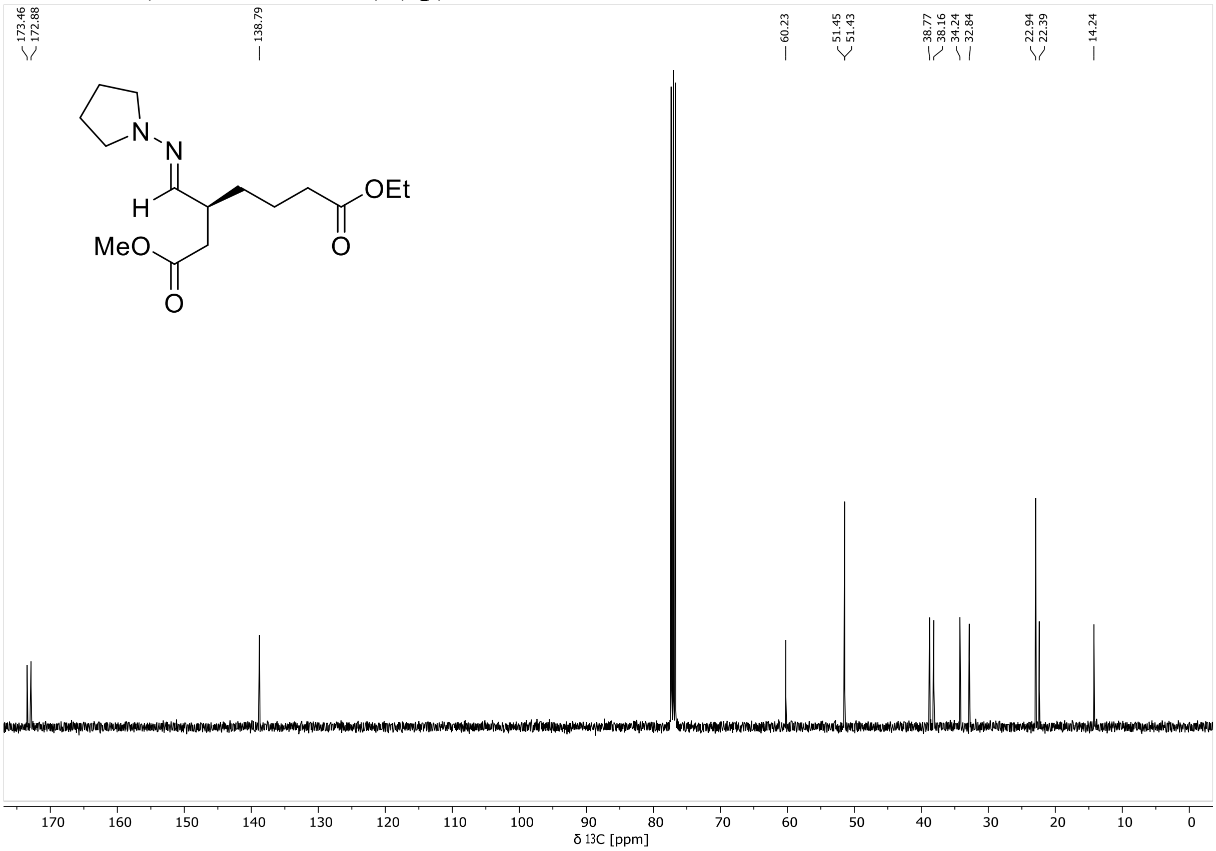


**$^{1}$H NMR** (400 MHz, $CDCl_3$) (**4q**):

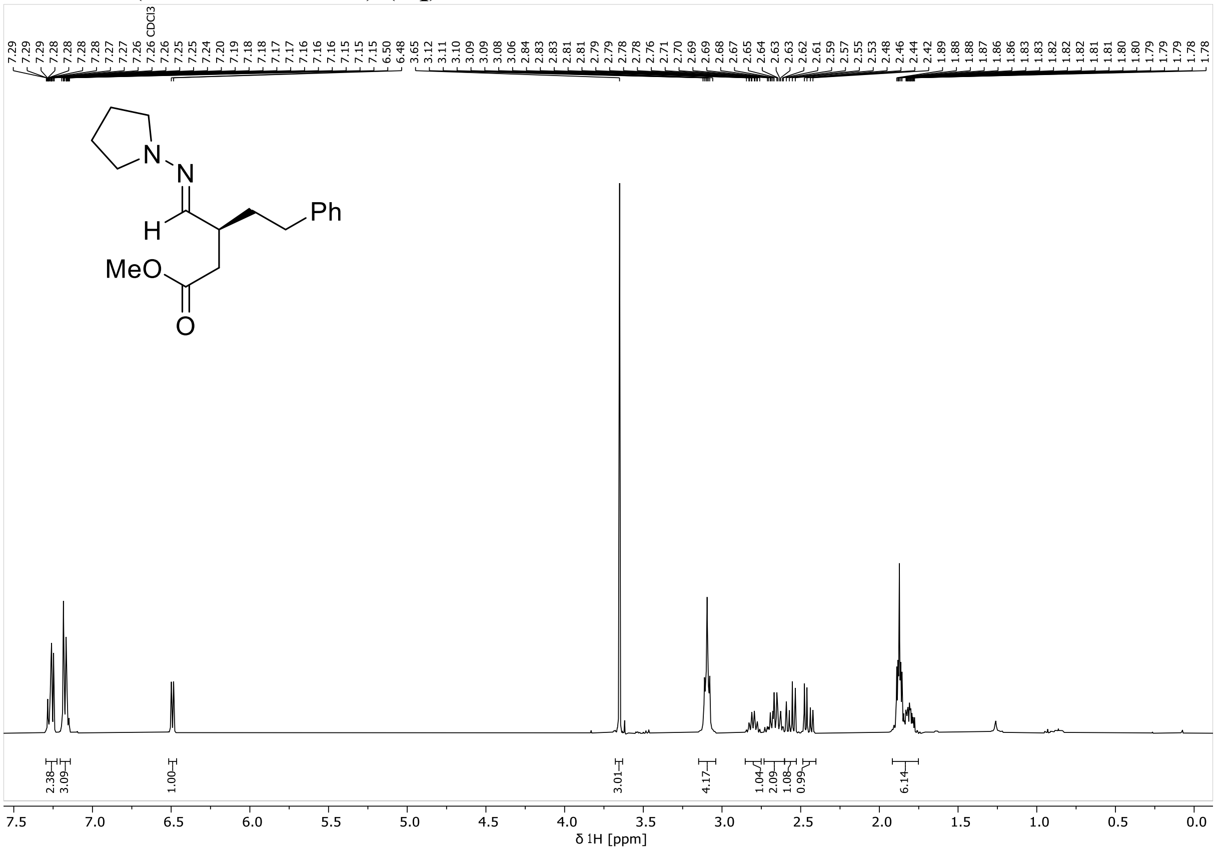

**$^{13}$C NMR** (101 MHz, $CDCl_3$) (**4q**):

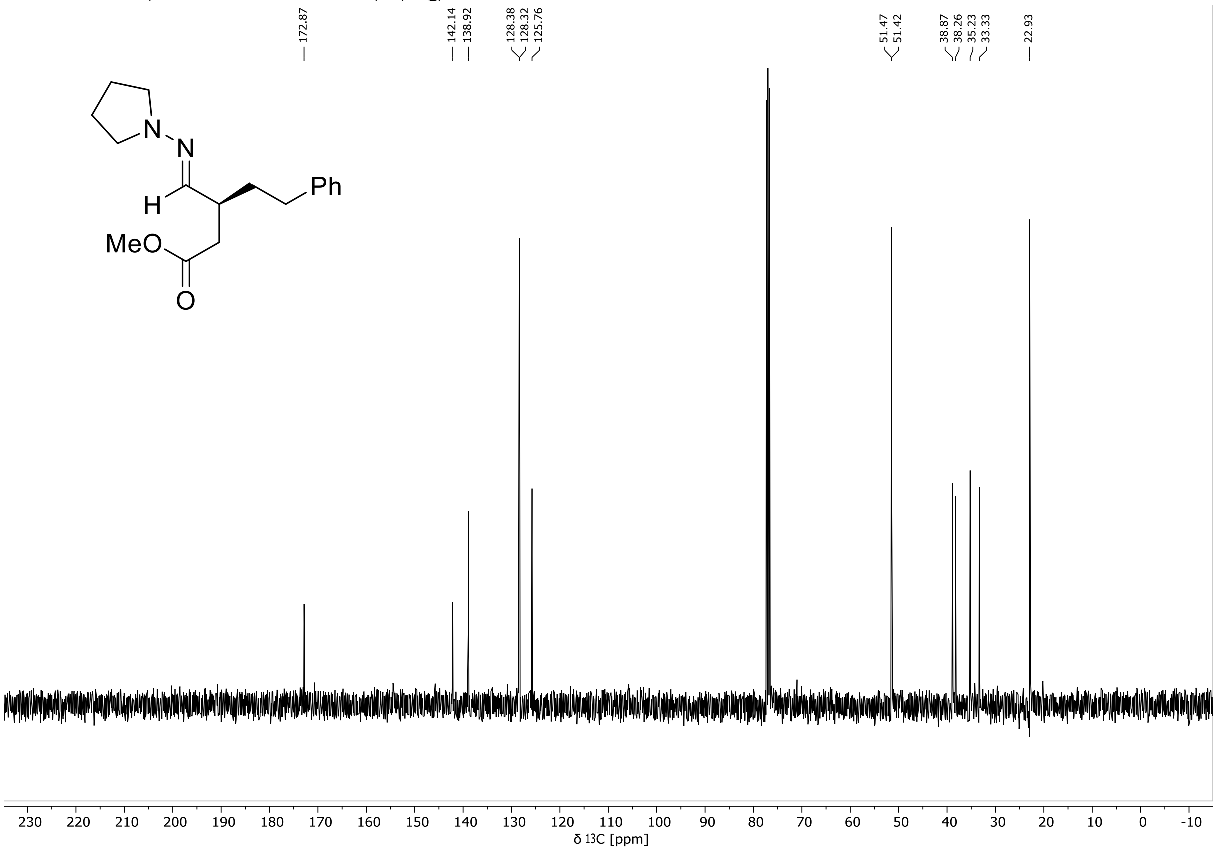

**$^{1}$H NMR** (400 MHz, $CDCl_3$) (**4r**):

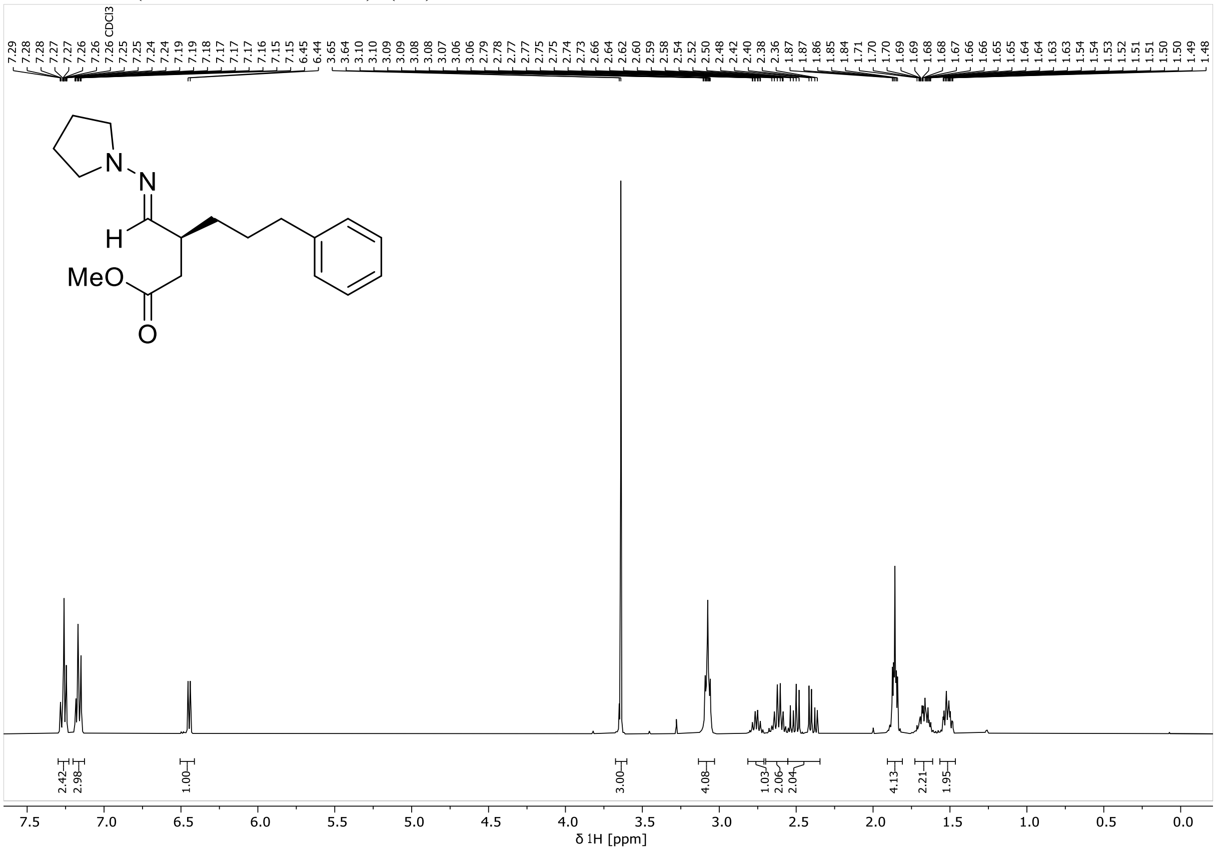

**$^{13}$C NMR** (101 MHz, $CDCl_3$) (**4r**):

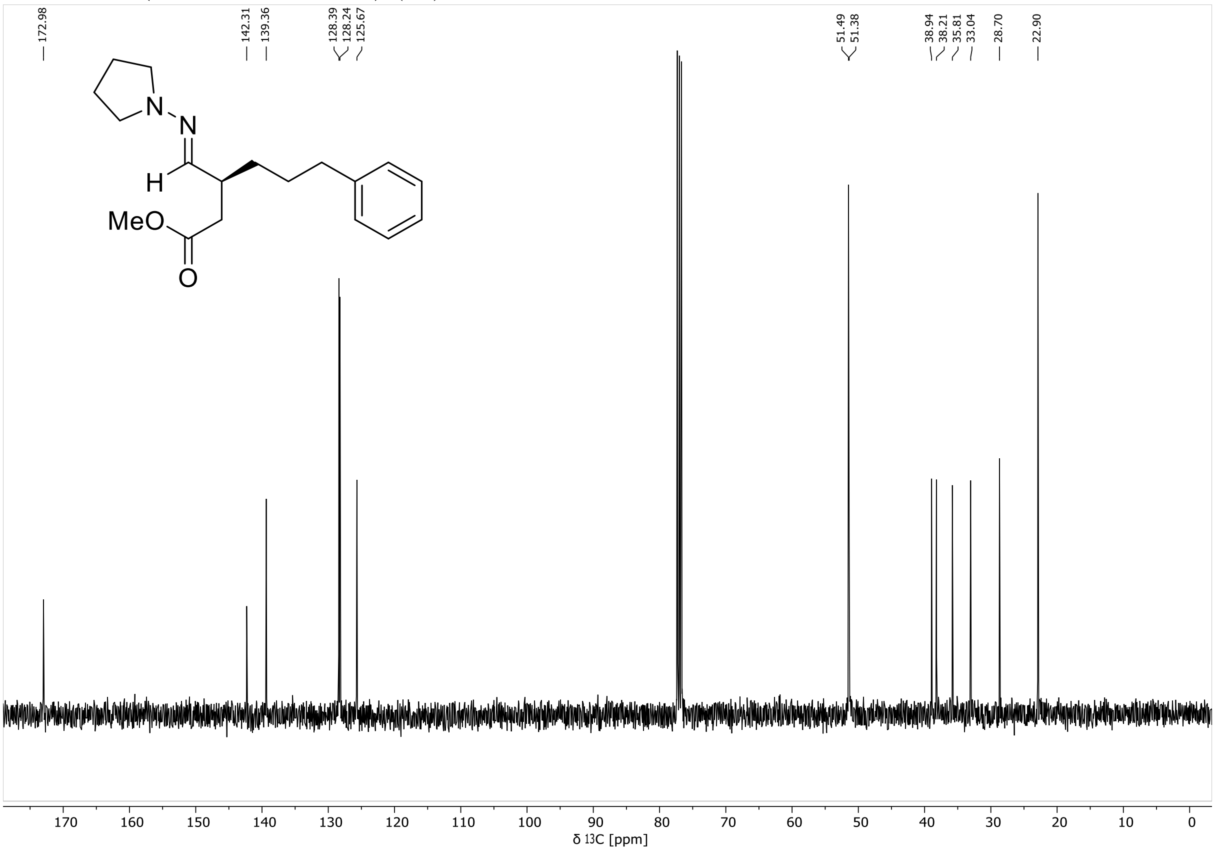


**$^{1}$H NMR** (400 MHz, $CDCl_3$) (**4s**):

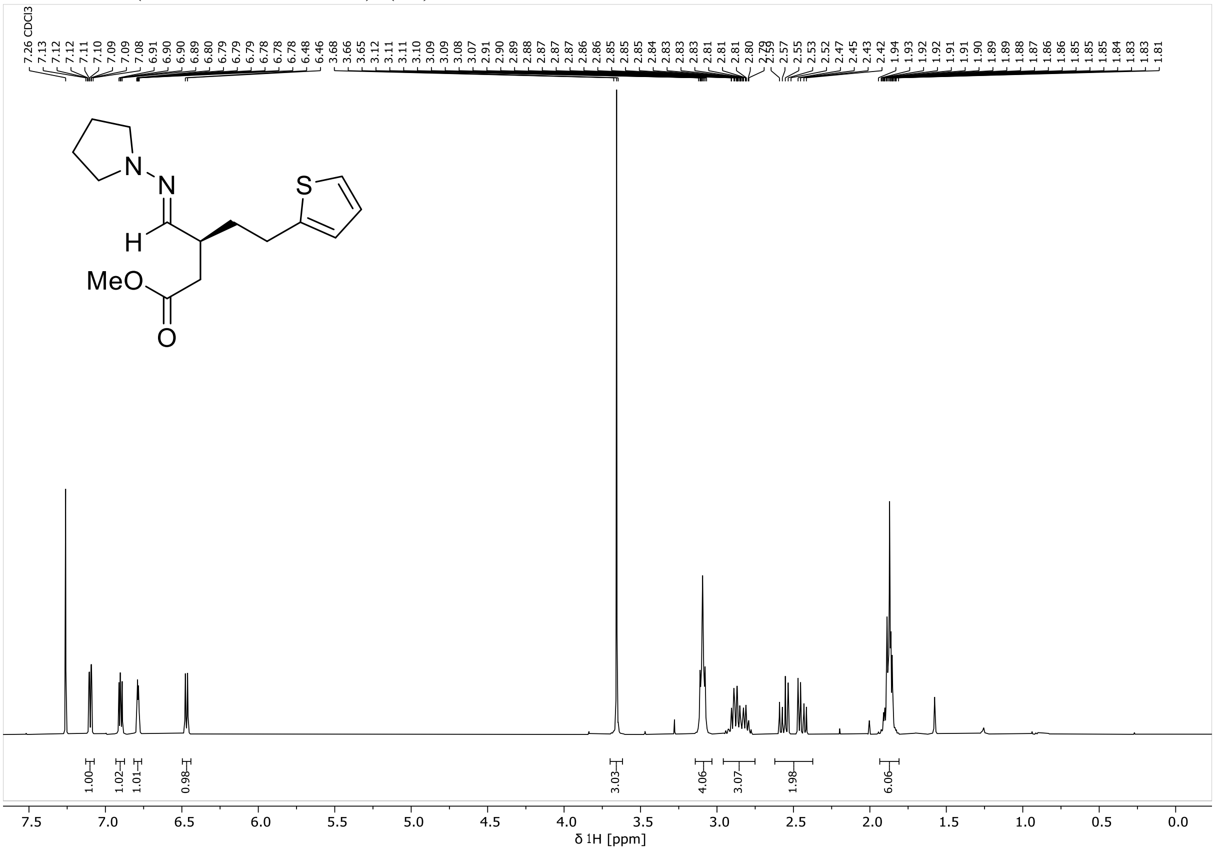

**$^{13}$C NMR** (101 MHz, $CDCl_3$) (**4s**):

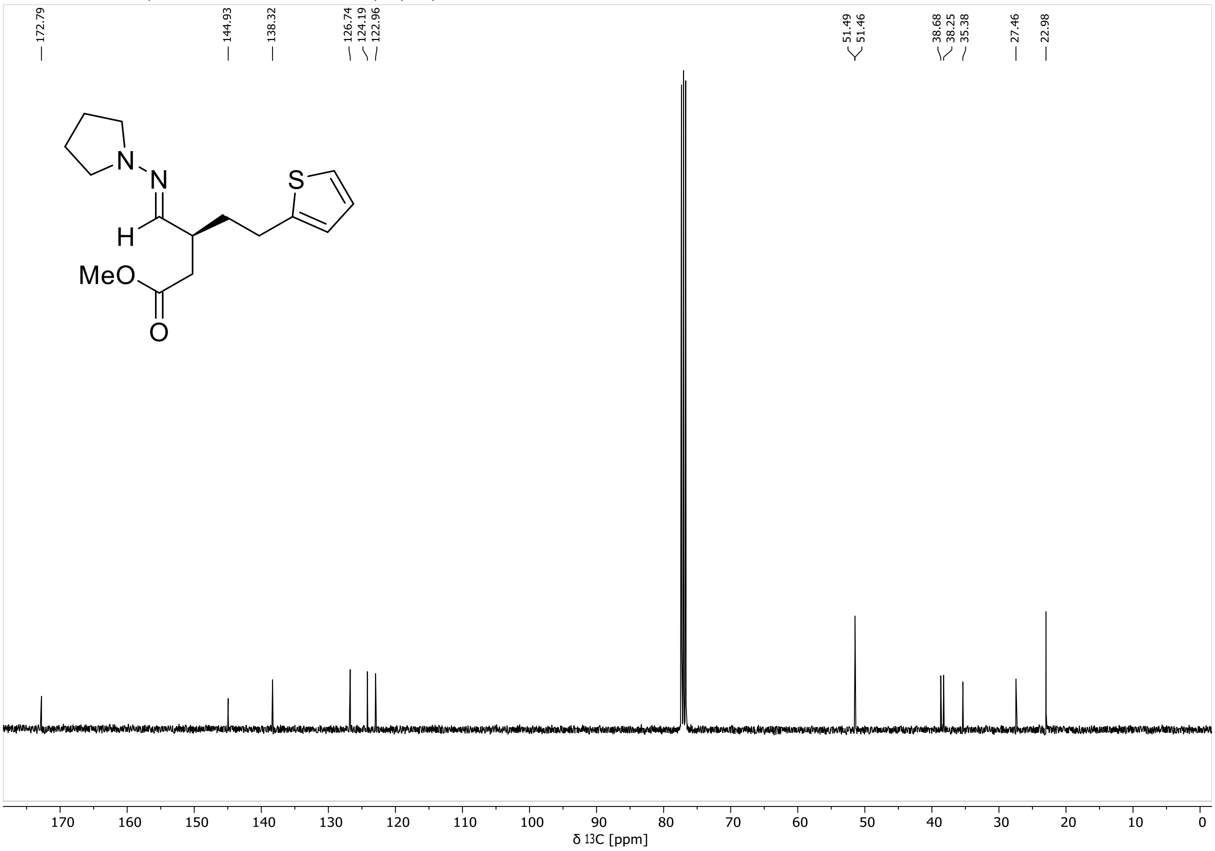

**$^{1}$H NMR** (400 MHz, $CDCl_3$) (**4t**):

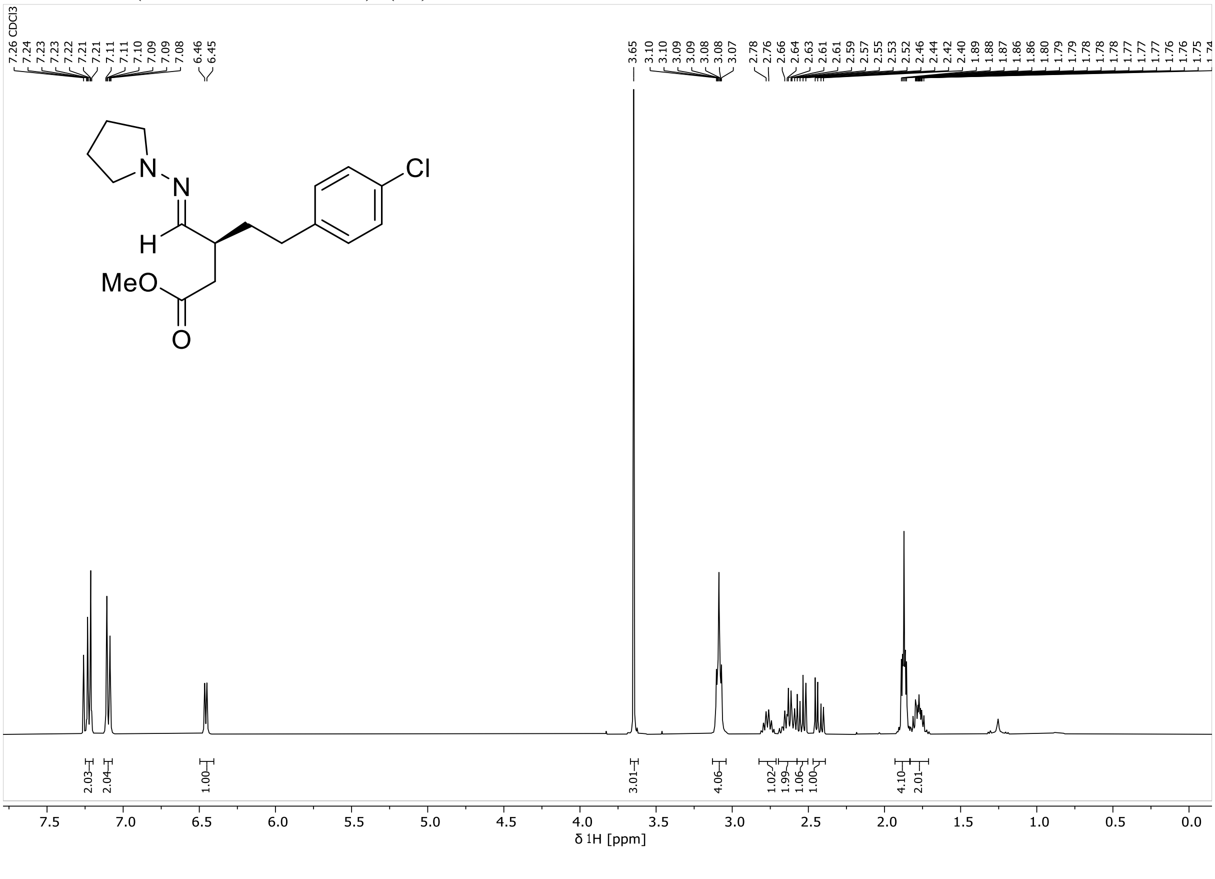

**$^{13}$C NMR** (101 MHz, $CDCl_3$) (**4t**):

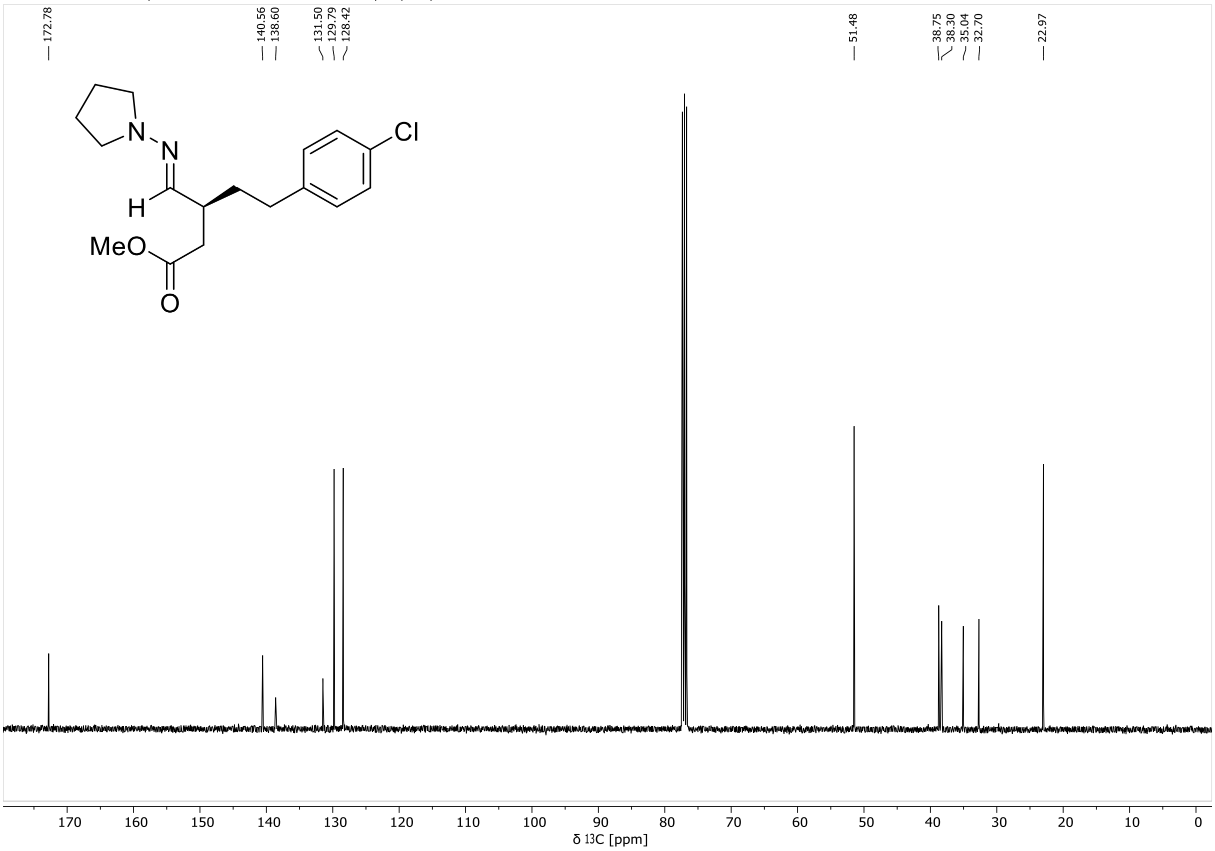


**$^{1}$H NMR** (400 MHz, $CDCl_3$) (**4u**):

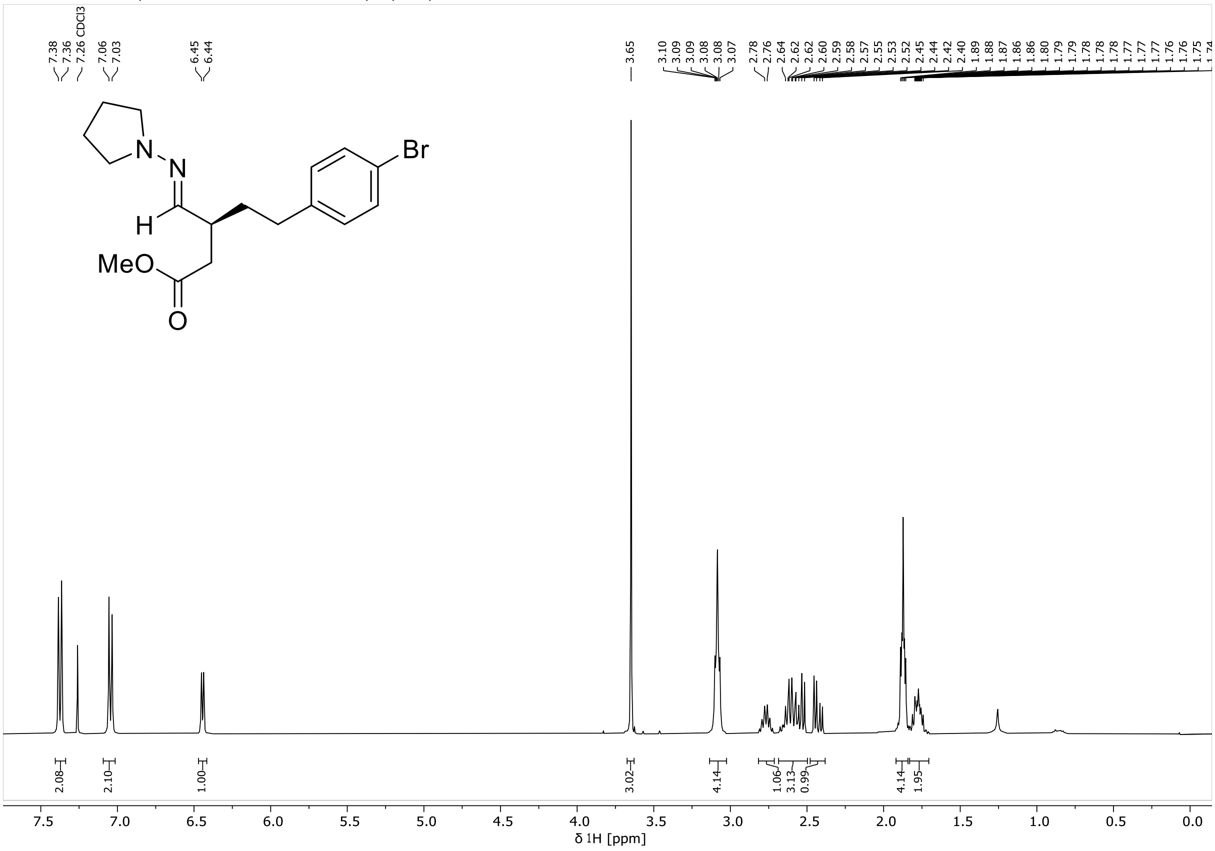

**$^{13}$C NMR** (101 MHz, $CDCl_3$) (**4u**):

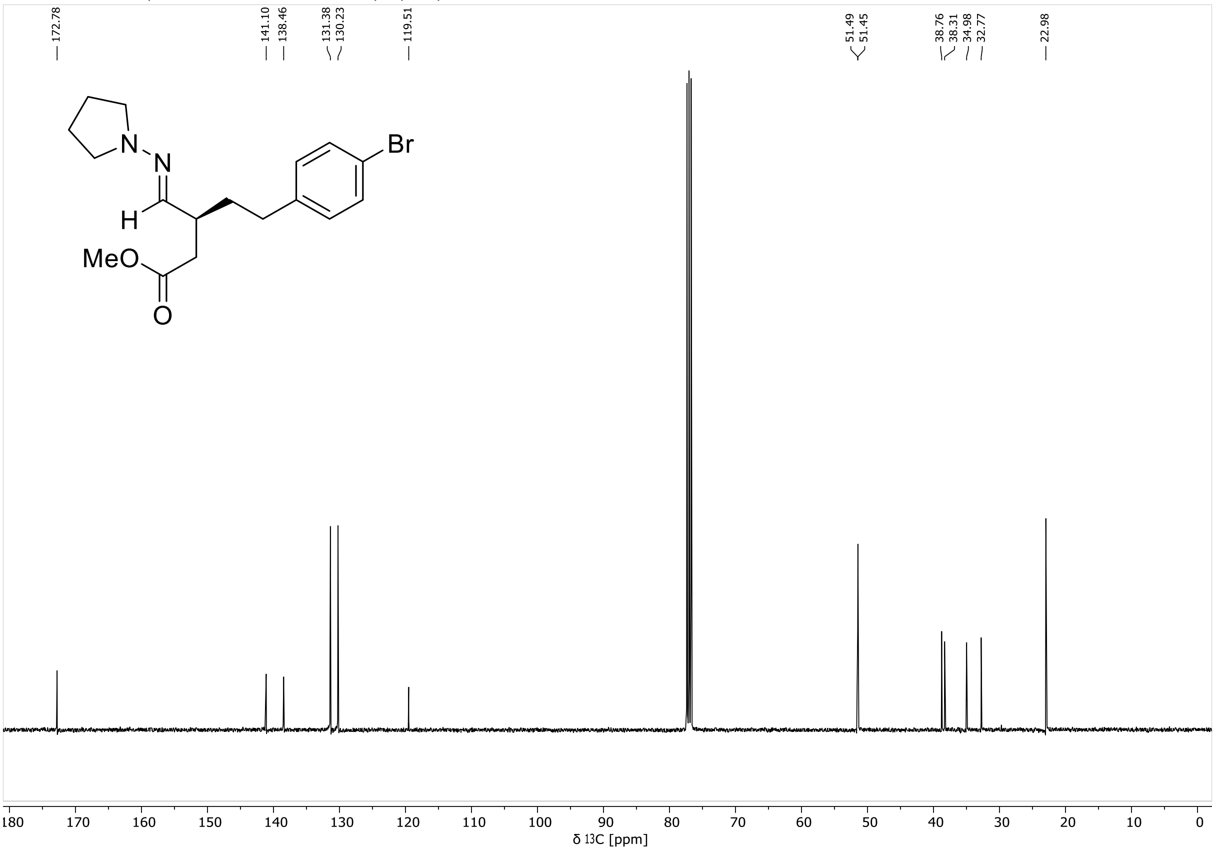


**$^{1}$H NMR** (400 MHz, $CDCl_3$) (**4v**):

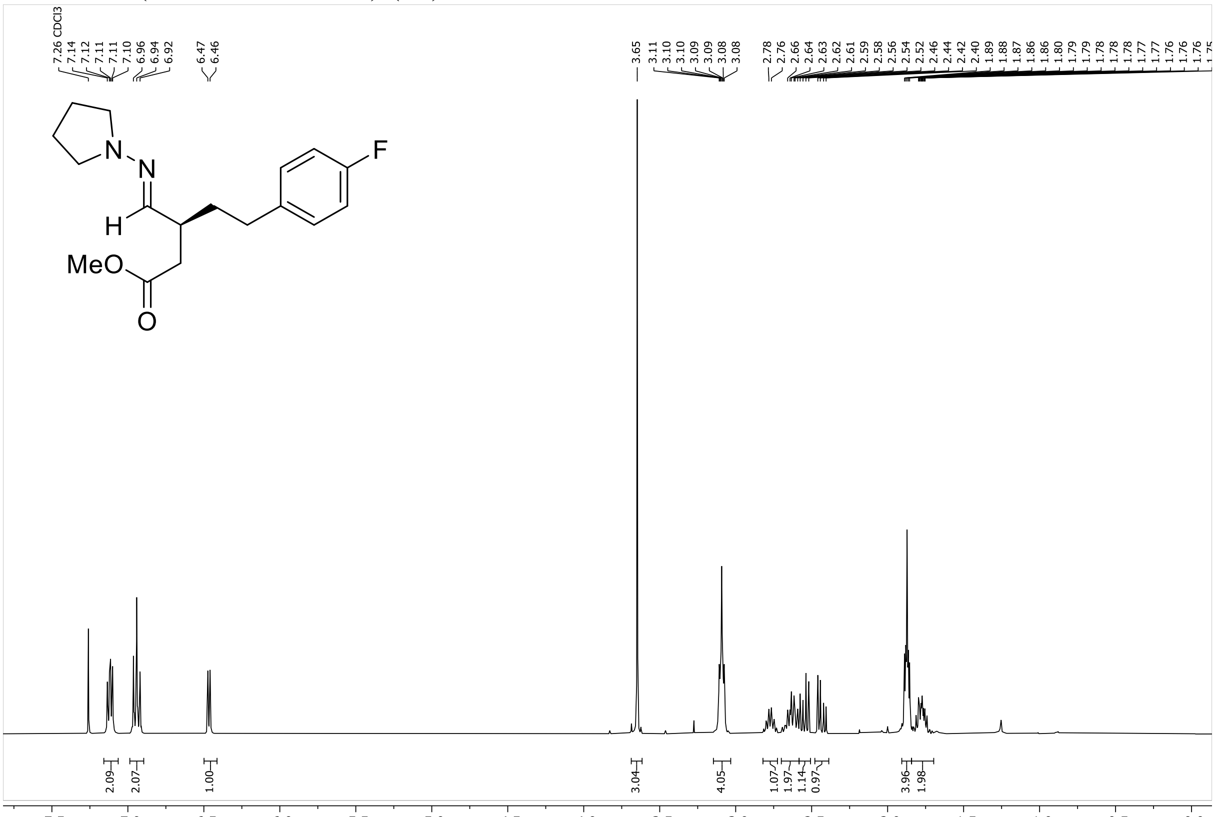

**¹³C NMR** (101 MHz, $CDCl_3$) (**4v**):

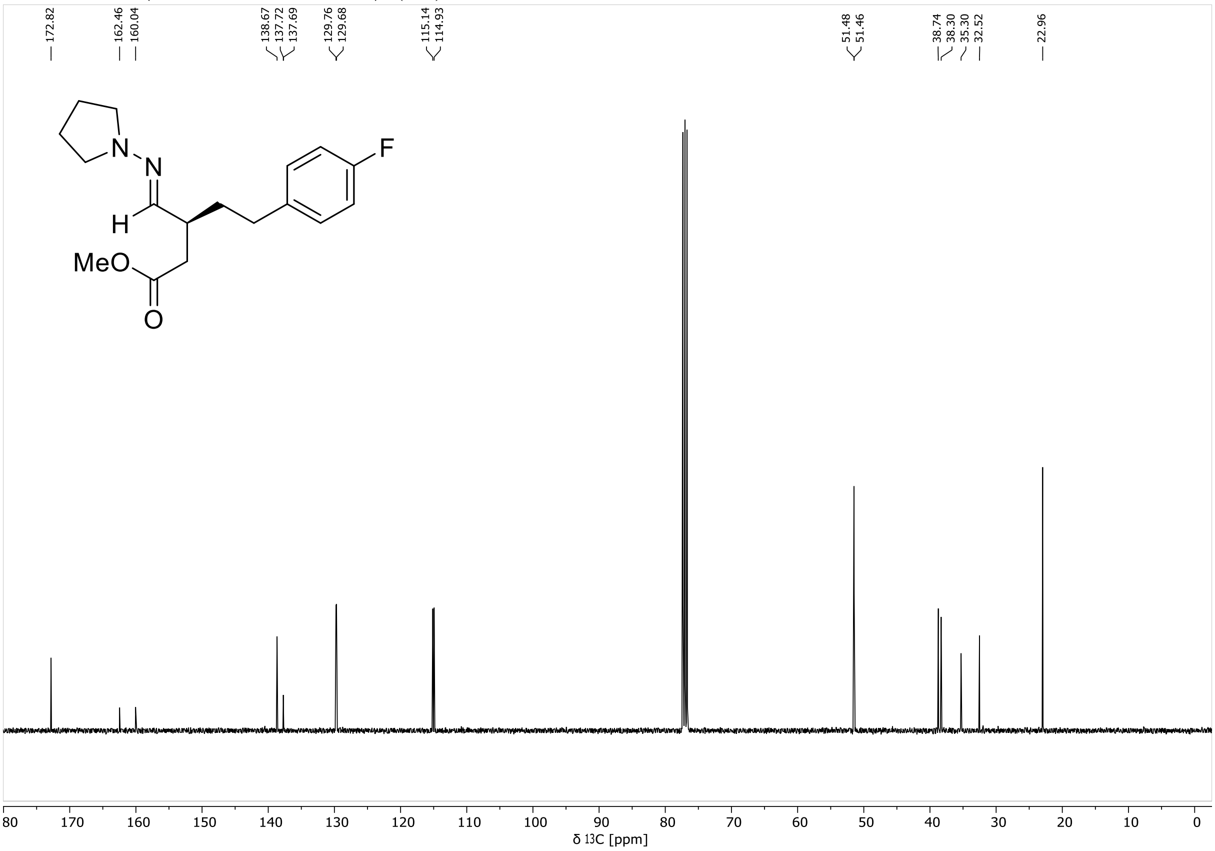


**¹⁹F NMR** (377 MHz, $CDCl_3$) (**4v**):

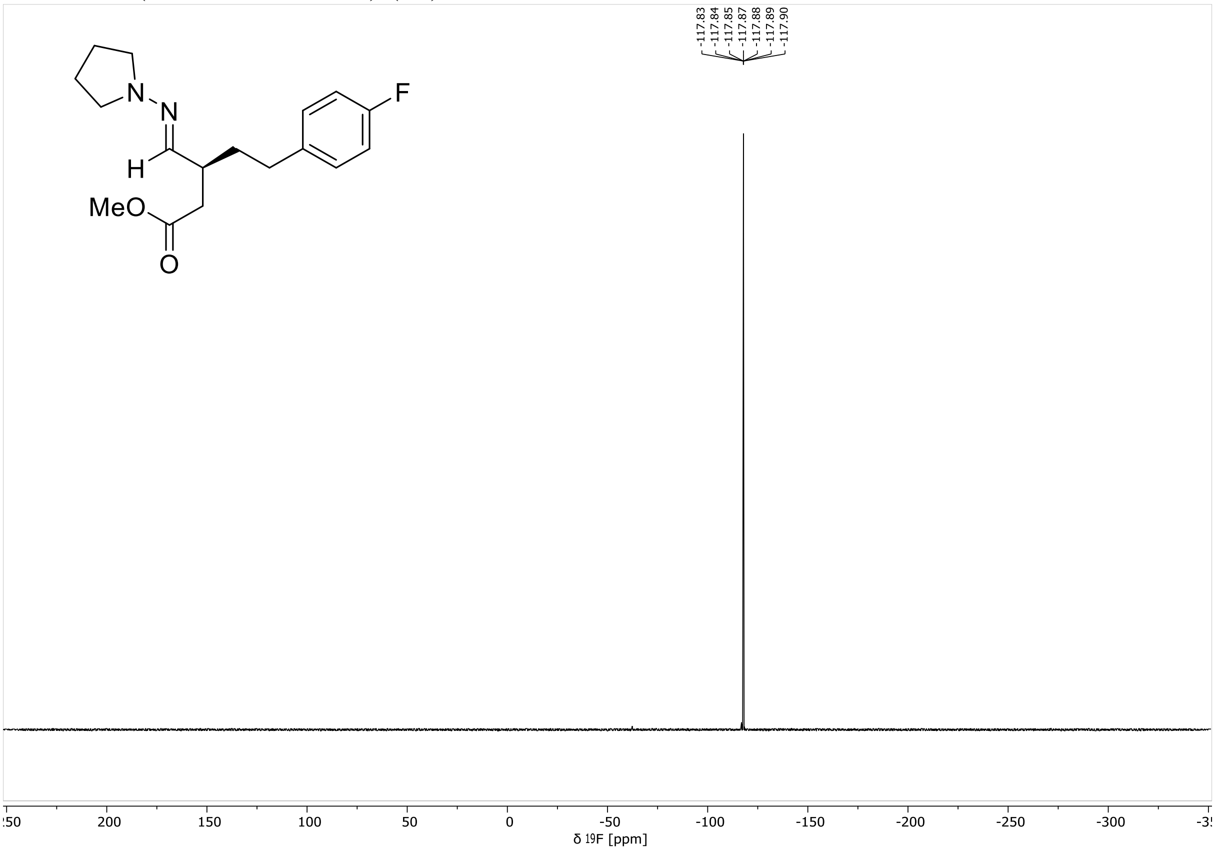

**$^{1}$H NMR** (400 MHz, $CDCl_3$) (**4w**):

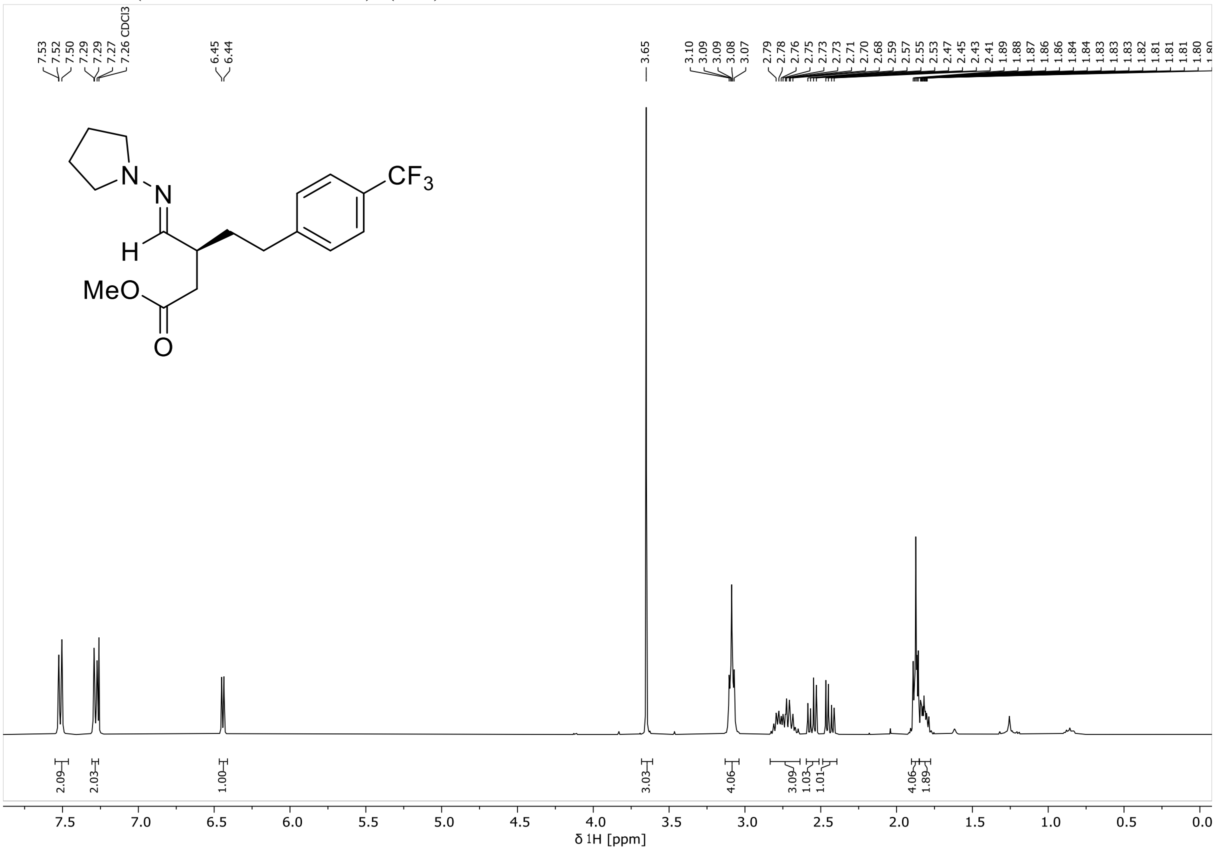

**$^{13}$C NMR** (101 MHz, $CDCl_3$) (**4w**):

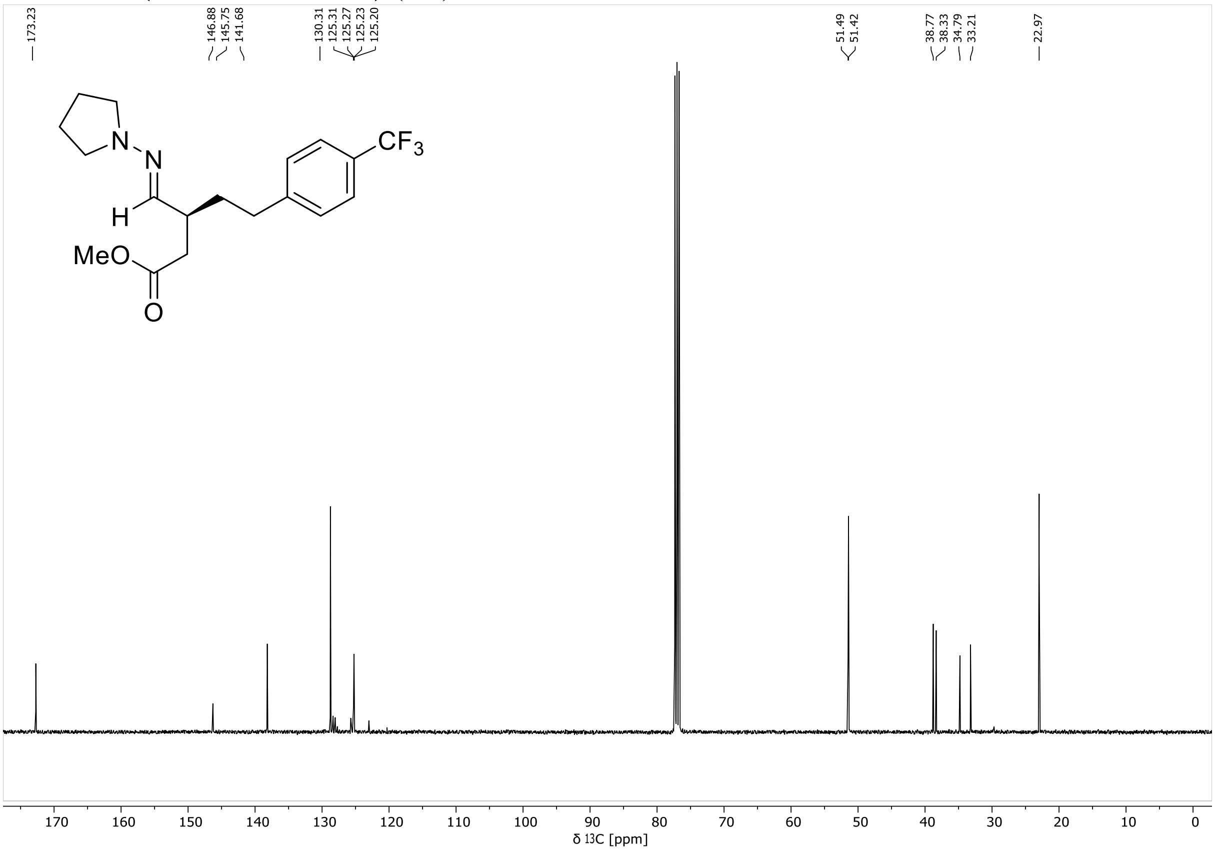

**$^{19}$F NMR** (377 MHz, $CDCl_3$) (**4w**):

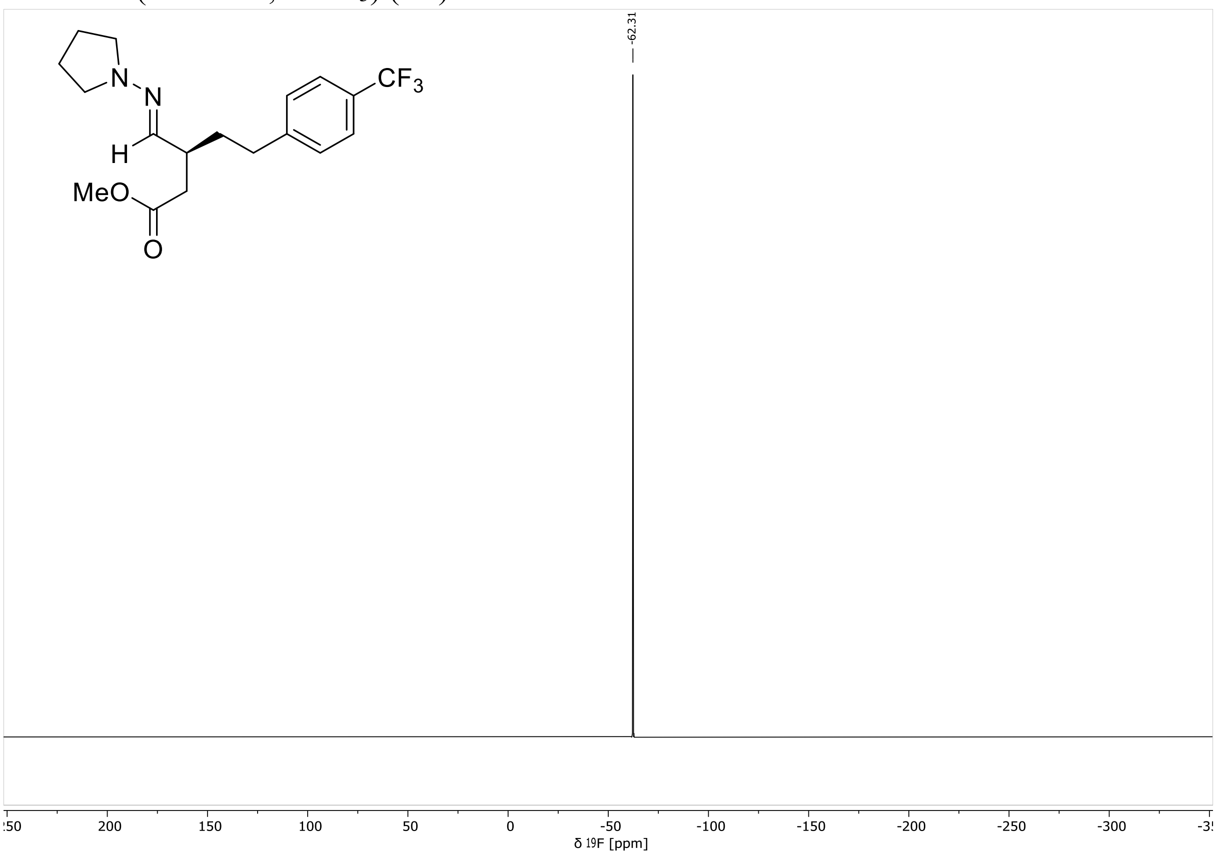


**$^{1}$H NMR** (400 MHz, $CDCl_3$) (**4x**):

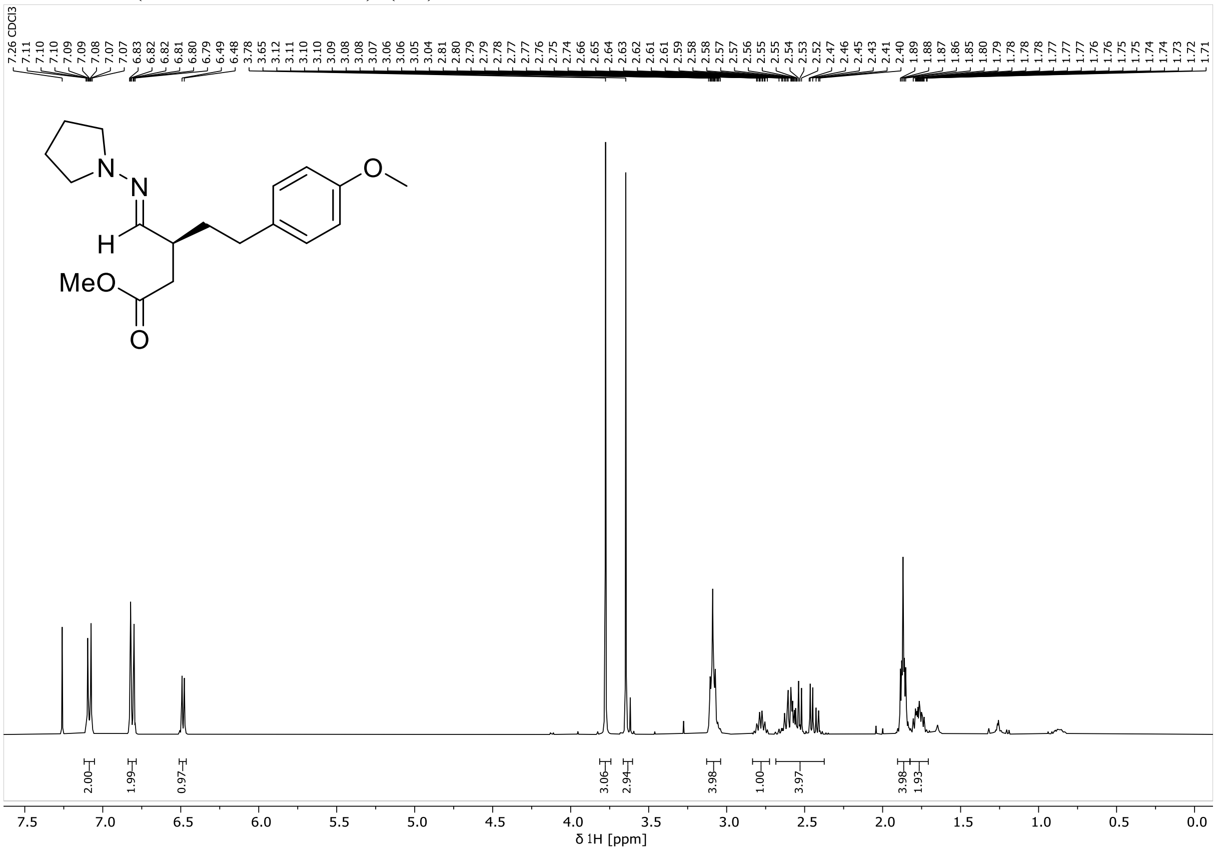

**$^{13}$C NMR** (101 MHz, $CDCl_3$) (**4x**):

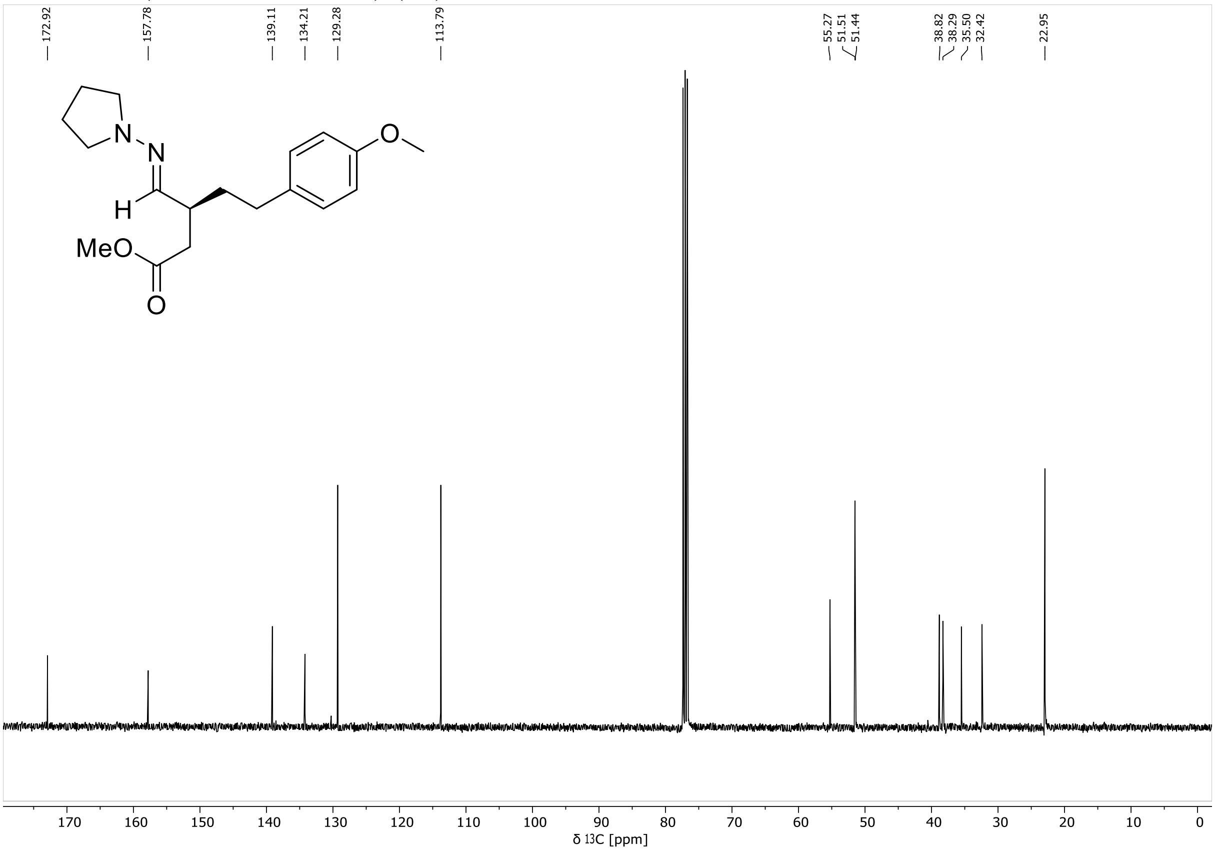

**$^{1}$H NMR** (400 MHz, $CDCl_3$) (**4y**):

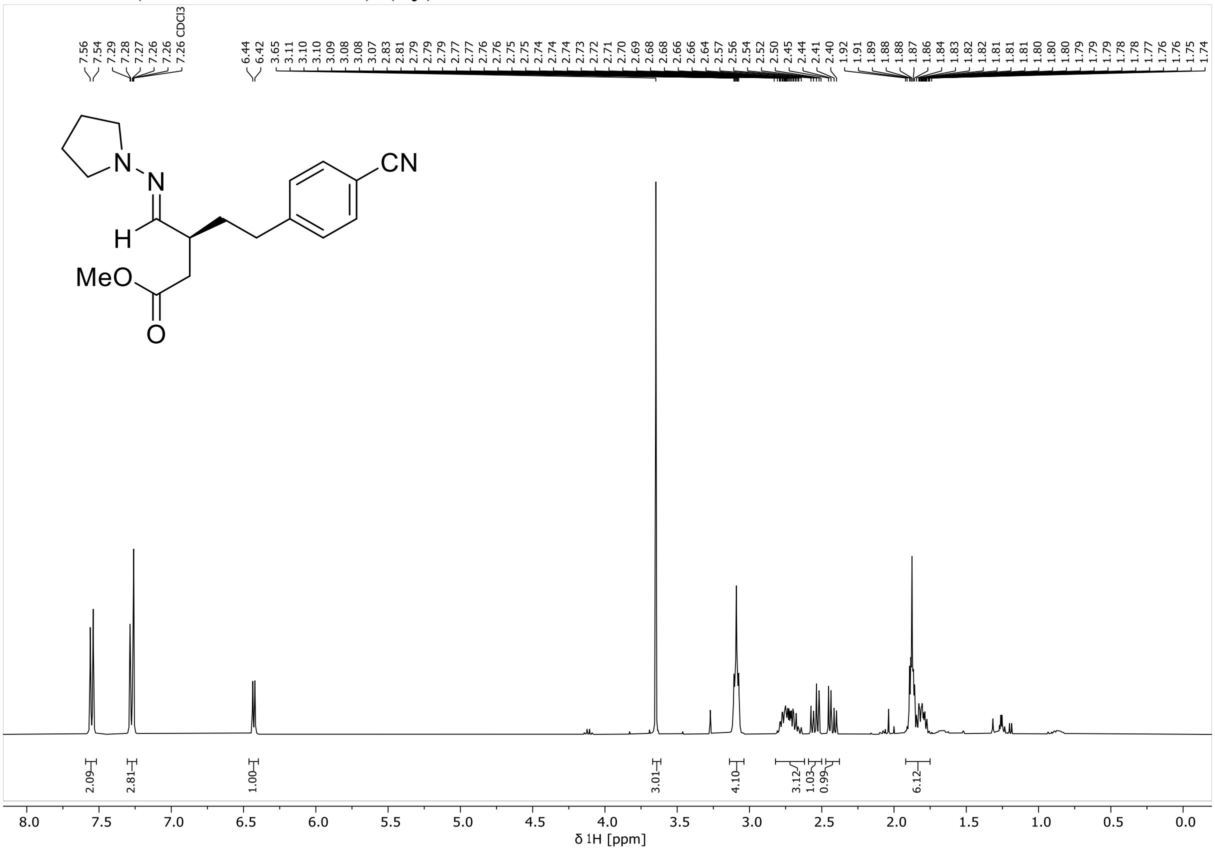

**$^{13}$C NMR** (101 MHz, $CDCl_3$) (**4y**):

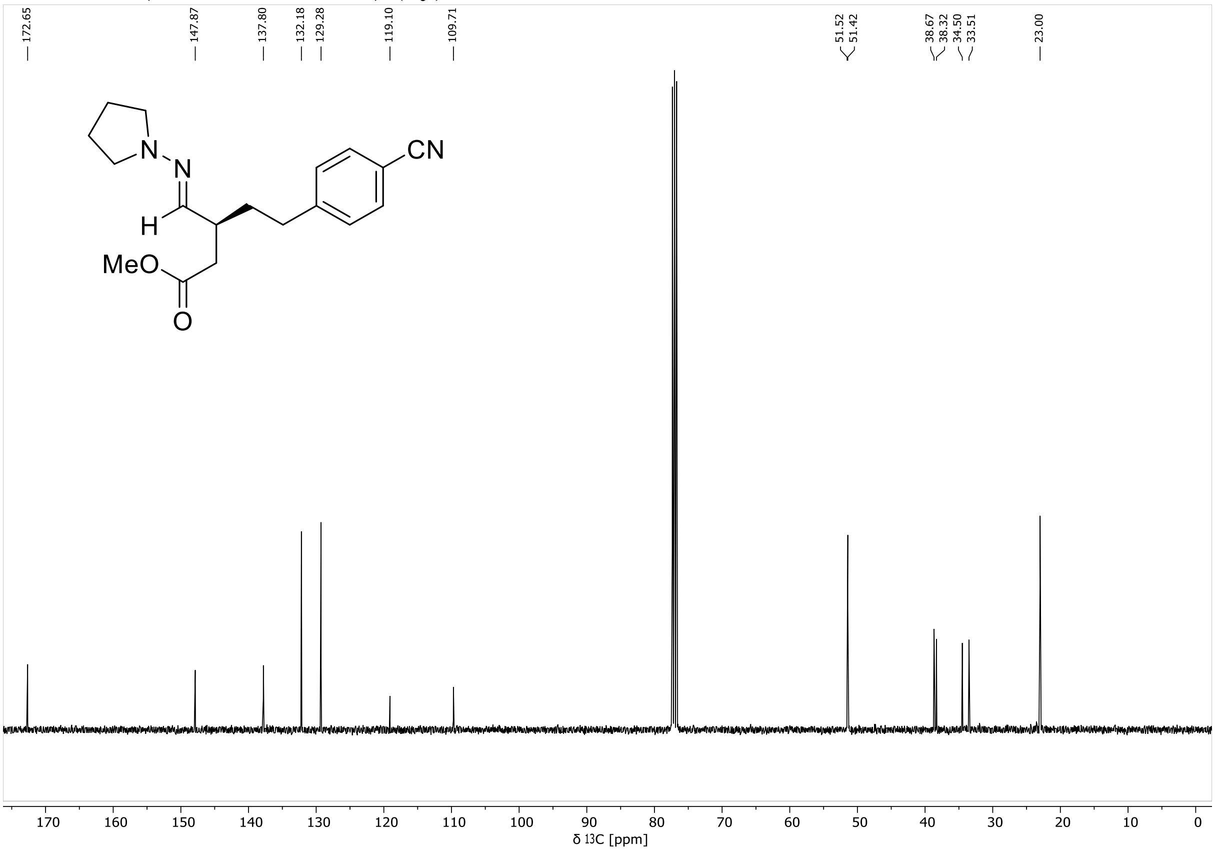

**$^{1}$H NMR** (400 MHz, $CDCl_3$) (*anti*-**4ab**):

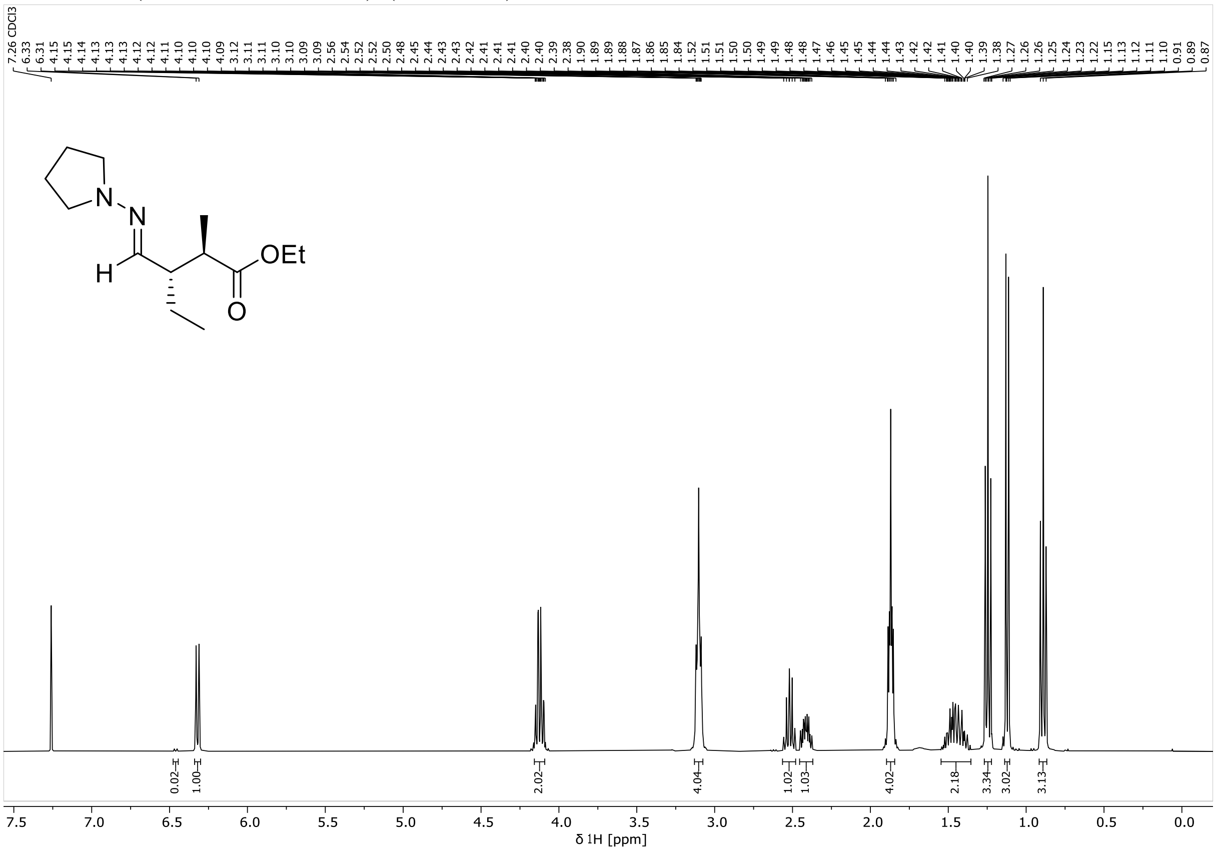


**$^{13}$C NMR** (101 MHz, $CDCl_3$) (*anti*-**4ab**):

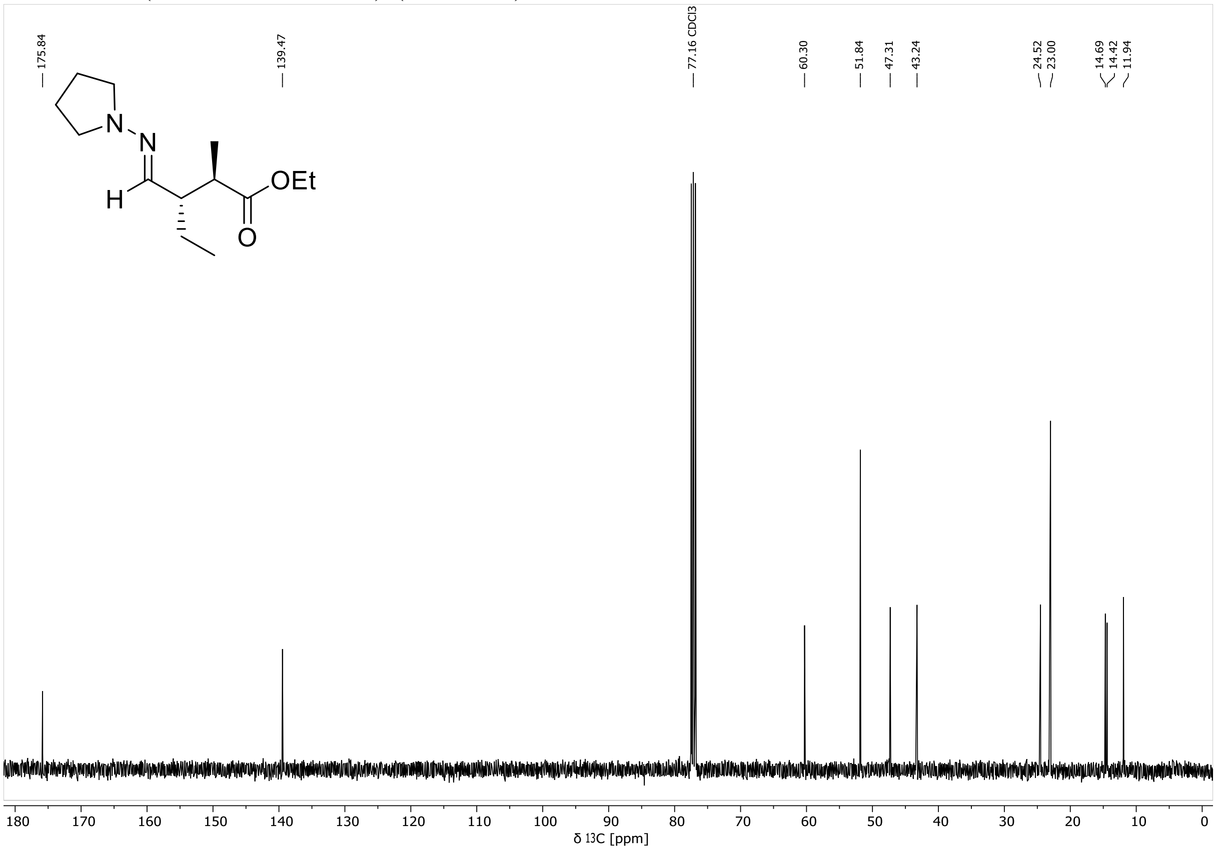

**$^{1}$H NMR** (400 MHz, $CDCl_3$) (*syn*-**4ab**):

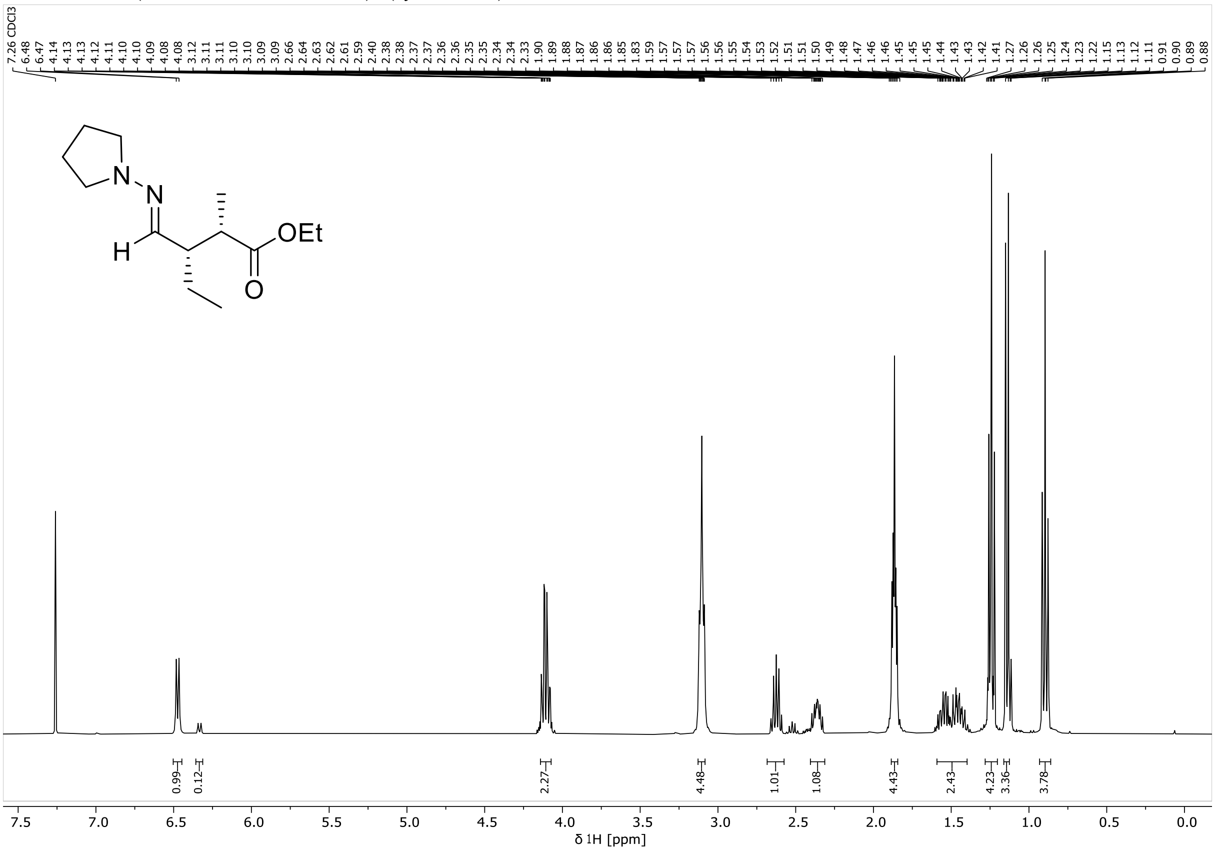

**$^{13}$C NMR** (101 MHz, $CDCl_3$) (*syn*-**4ab**):

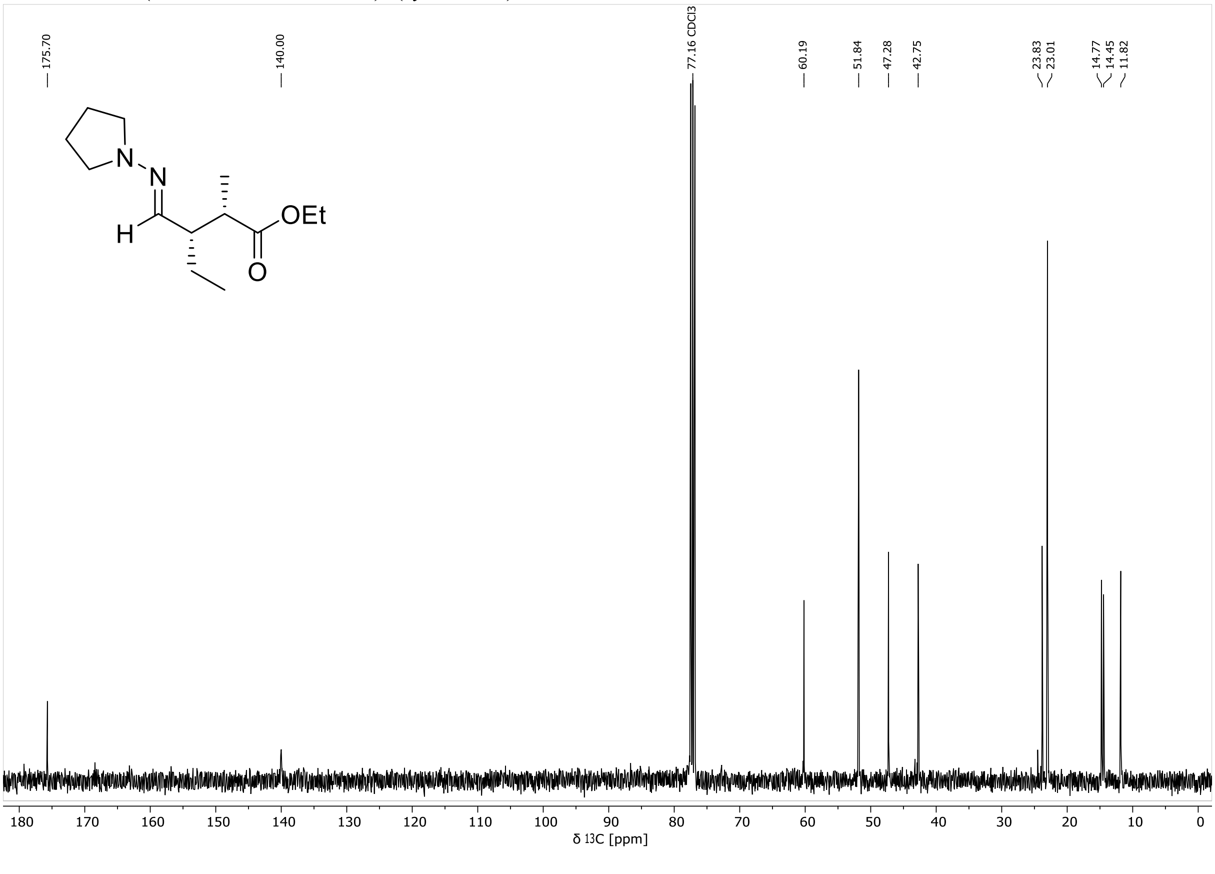

**$^{1}$H NMR** (400 MHz, $CDCl_3$) (*anti*-**4ac**):

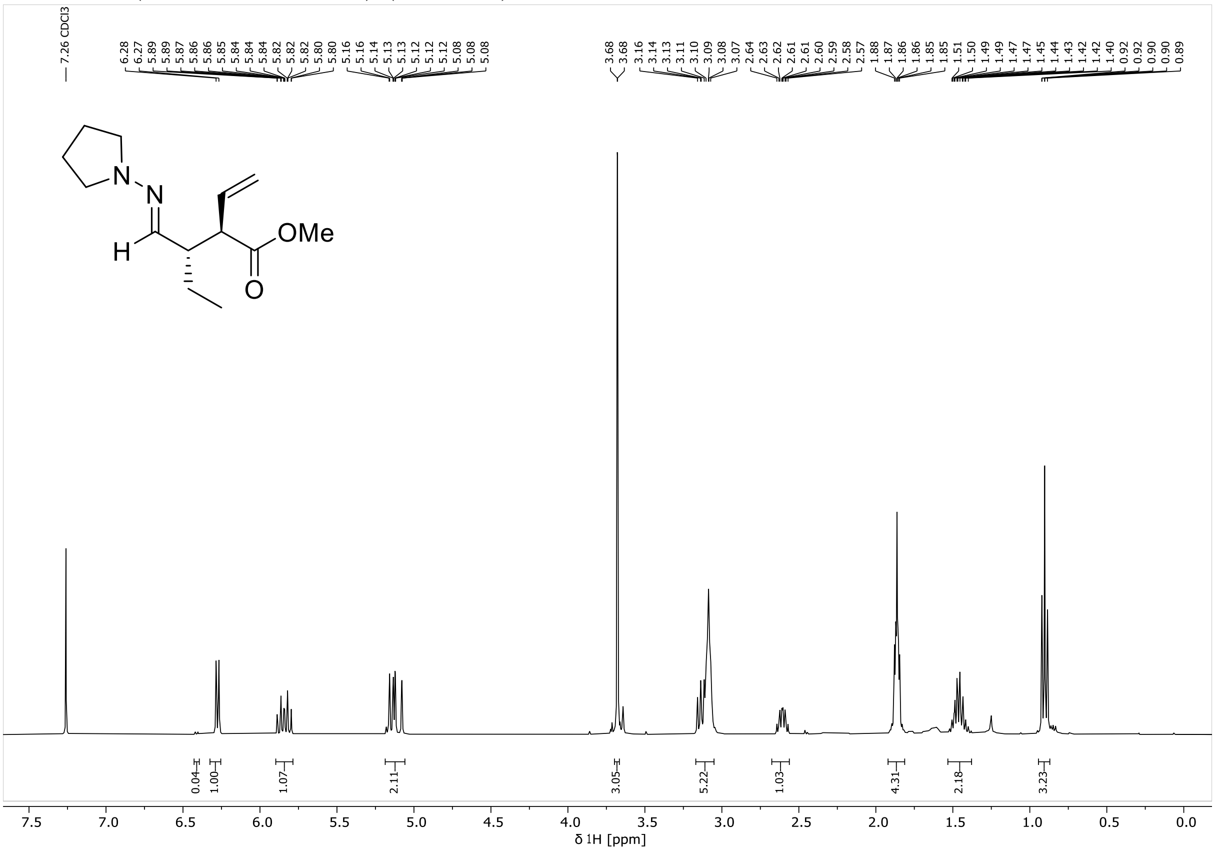


**$^{13}$C NMR** (101 MHz, $CDCl_3$) (*anti*-**4ac**):

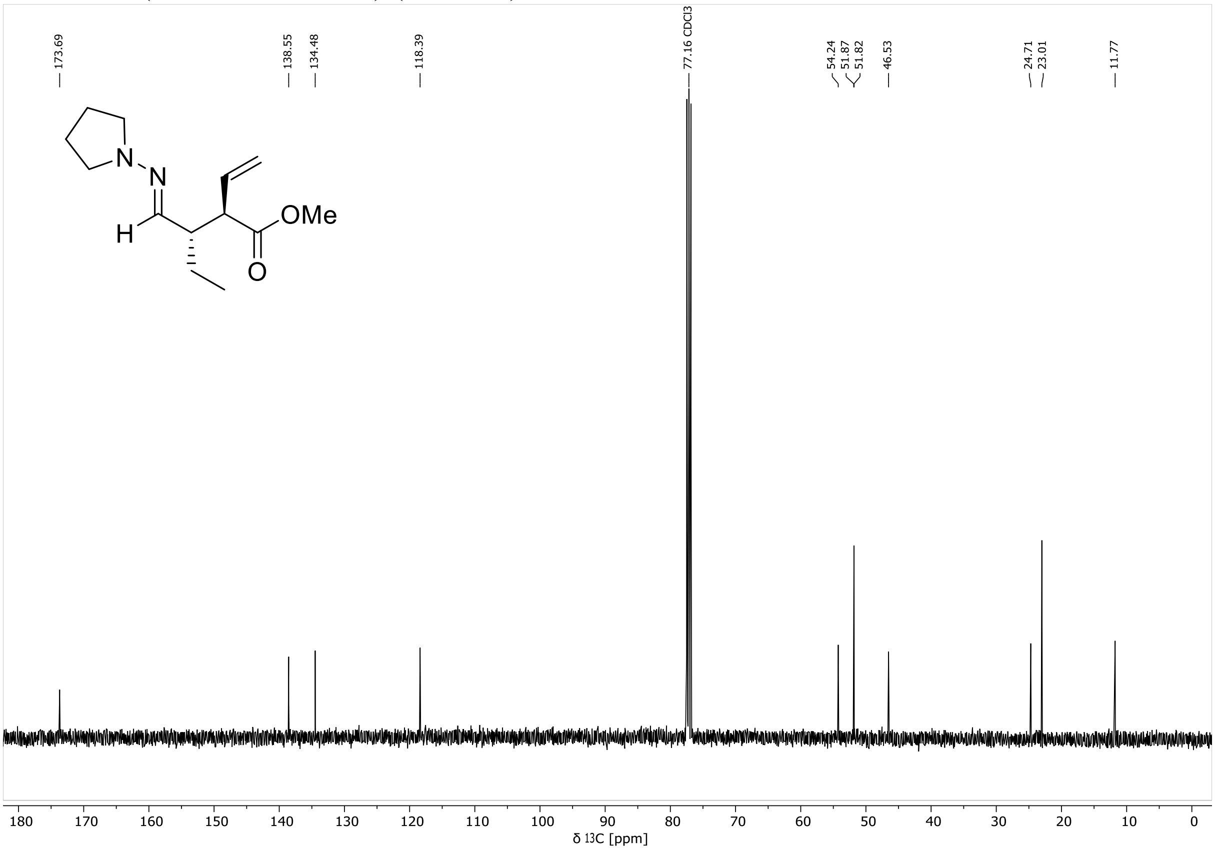

**Follow up:**

**$^1$H NMR** (400 MHz, $CDCl_3$) (**5g**):

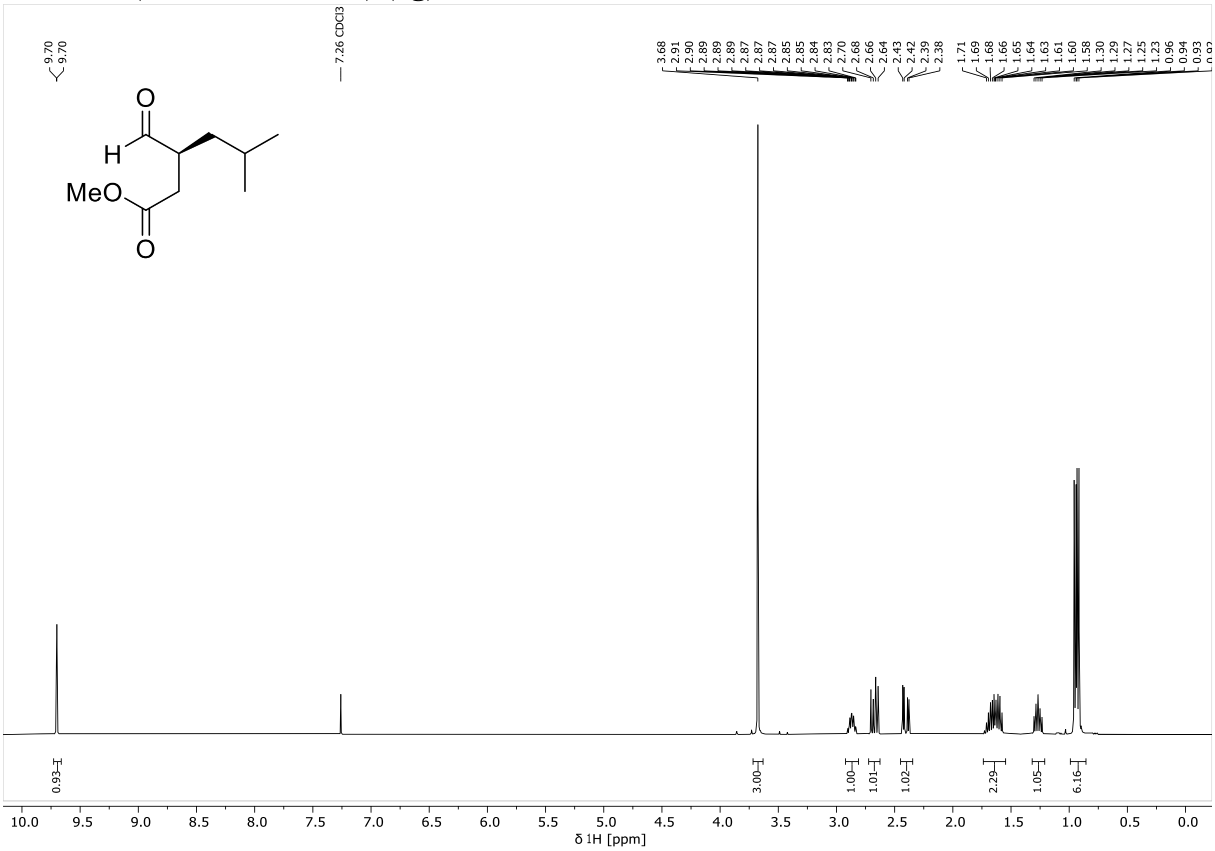

**$^{13}$C NMR** (101 MHz, $CDCl_3$) (**5g**):

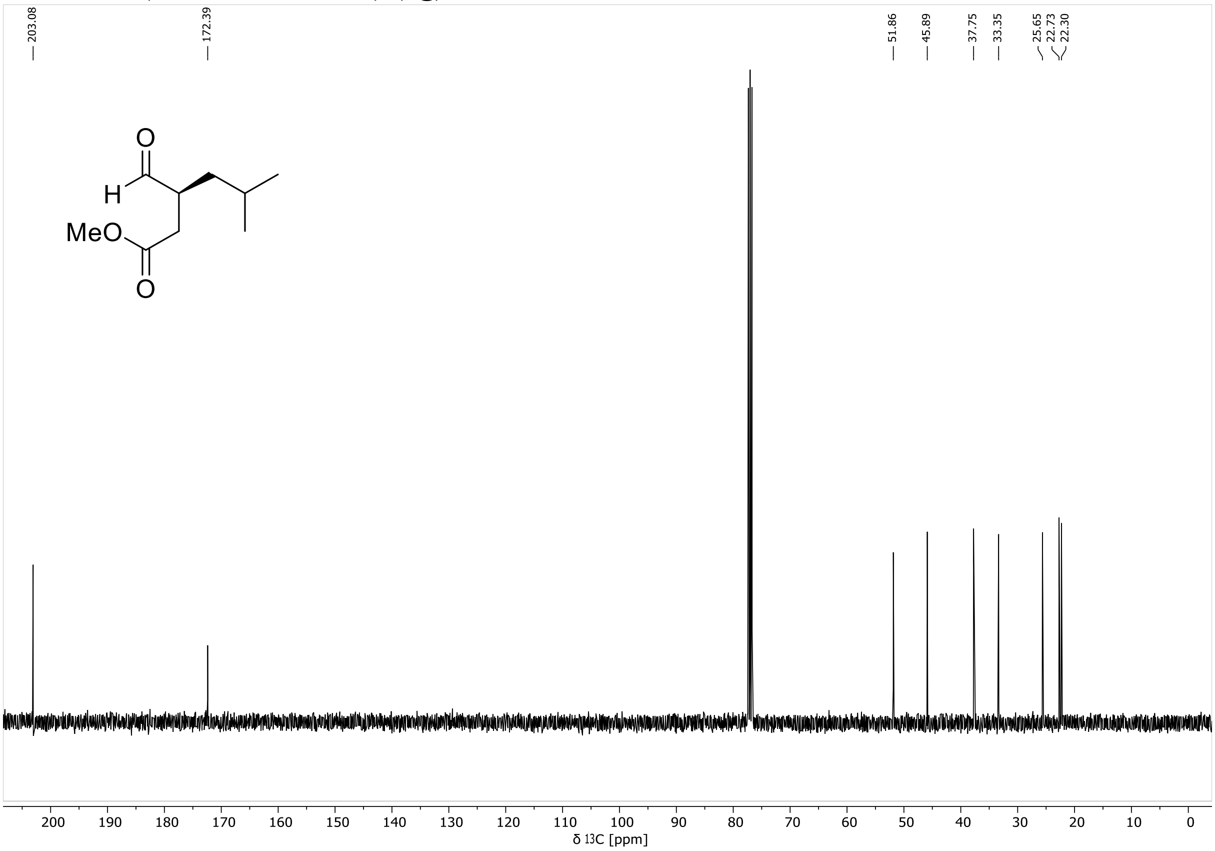

**¹H NMR** (400 MHz, $CDCl_3$) (**6c**):

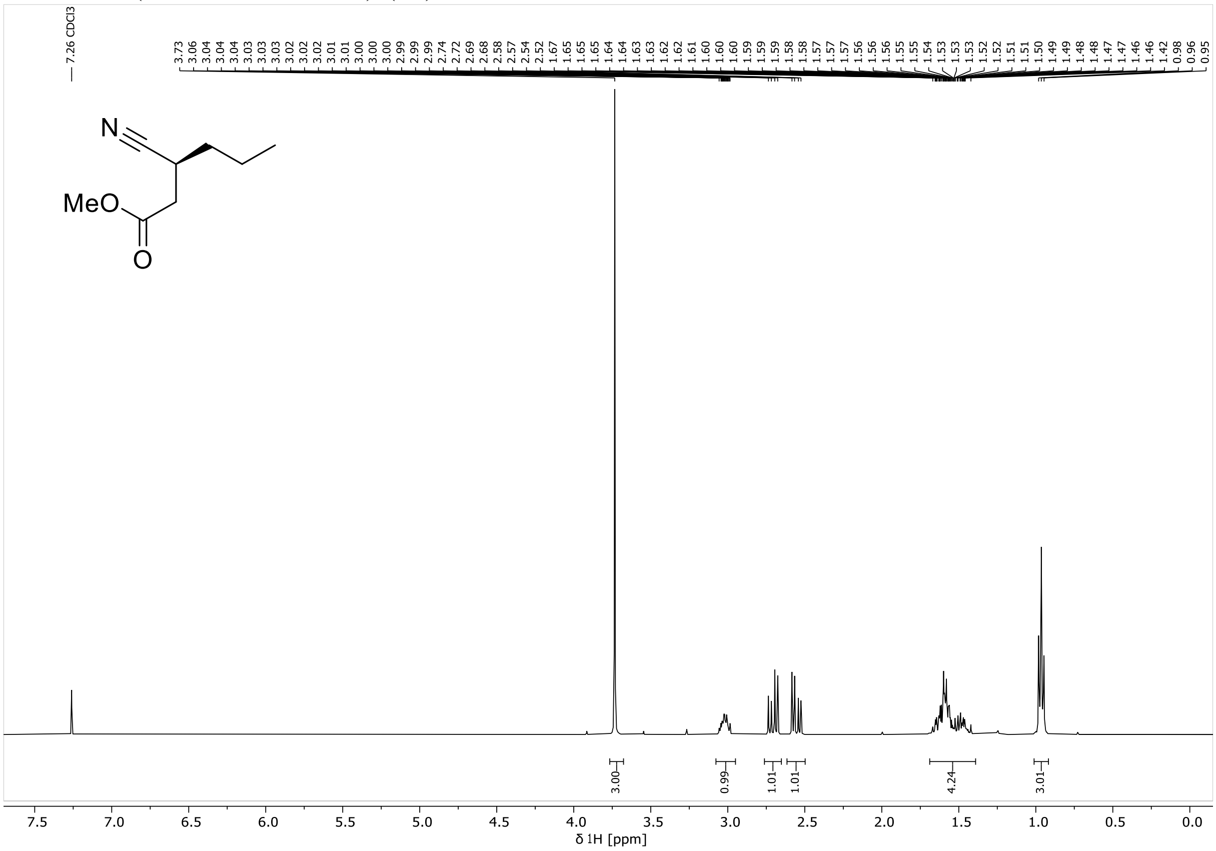

**¹³C NMR** (101 MHz, $CDCl_3$) (**6c**):

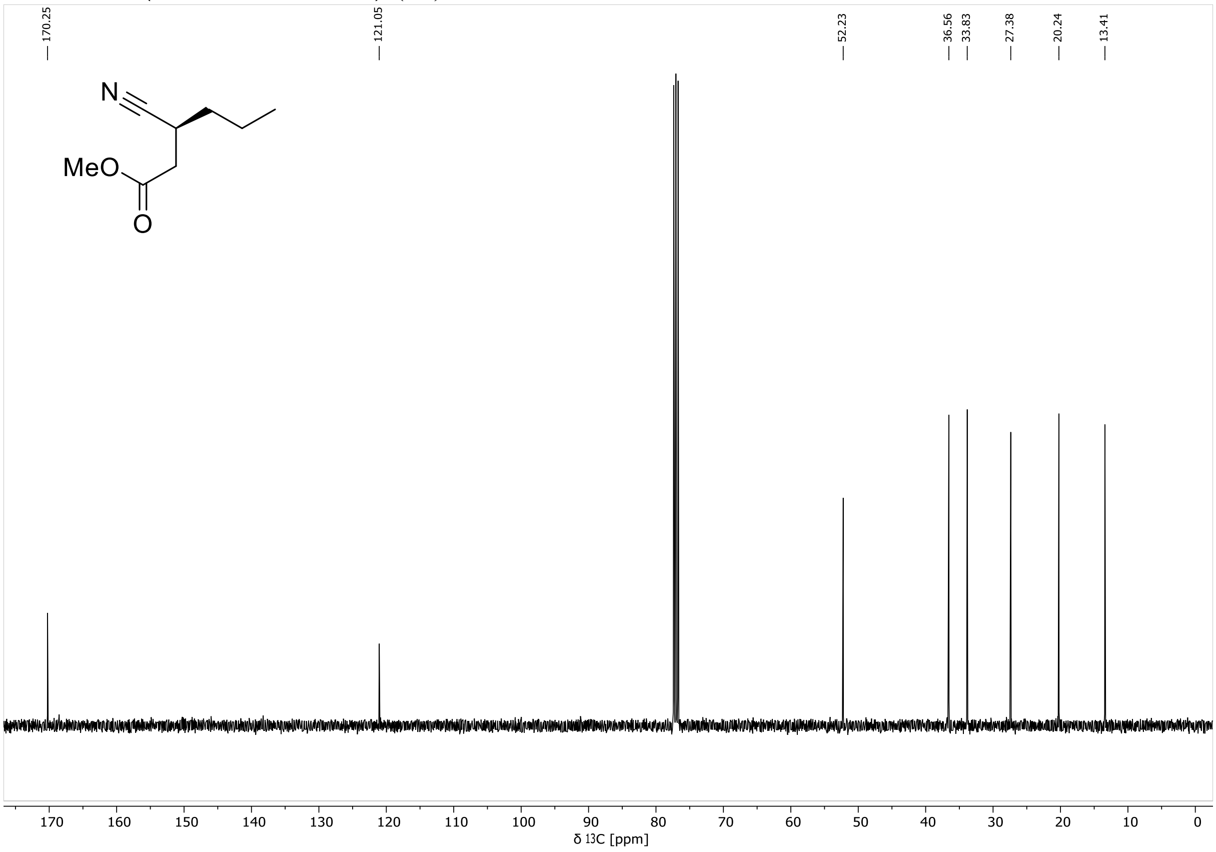

**$^{1}$H NMR** (400 MHz, $CDCl_3$) (**6g**):

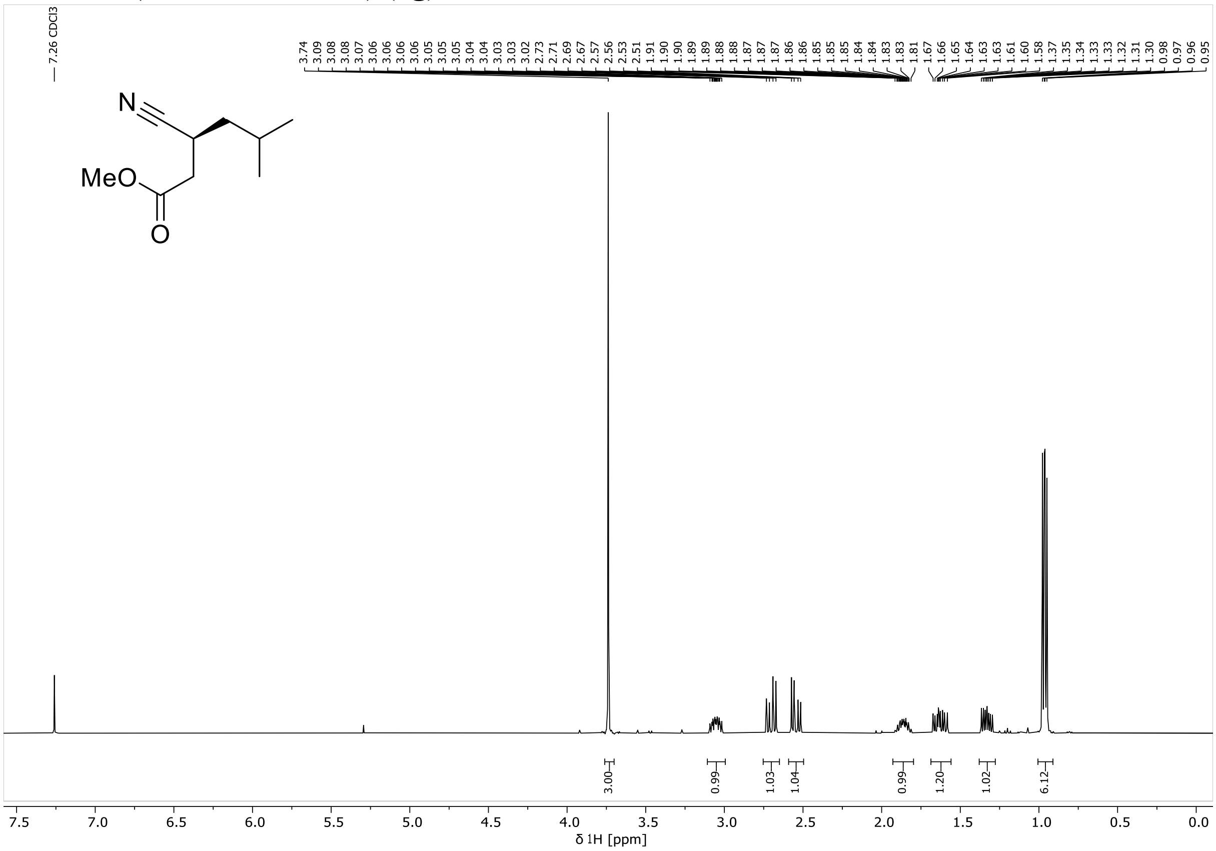


**$^{13}$C NMR** (101 MHz, $CDCl_3$) (**6g**):

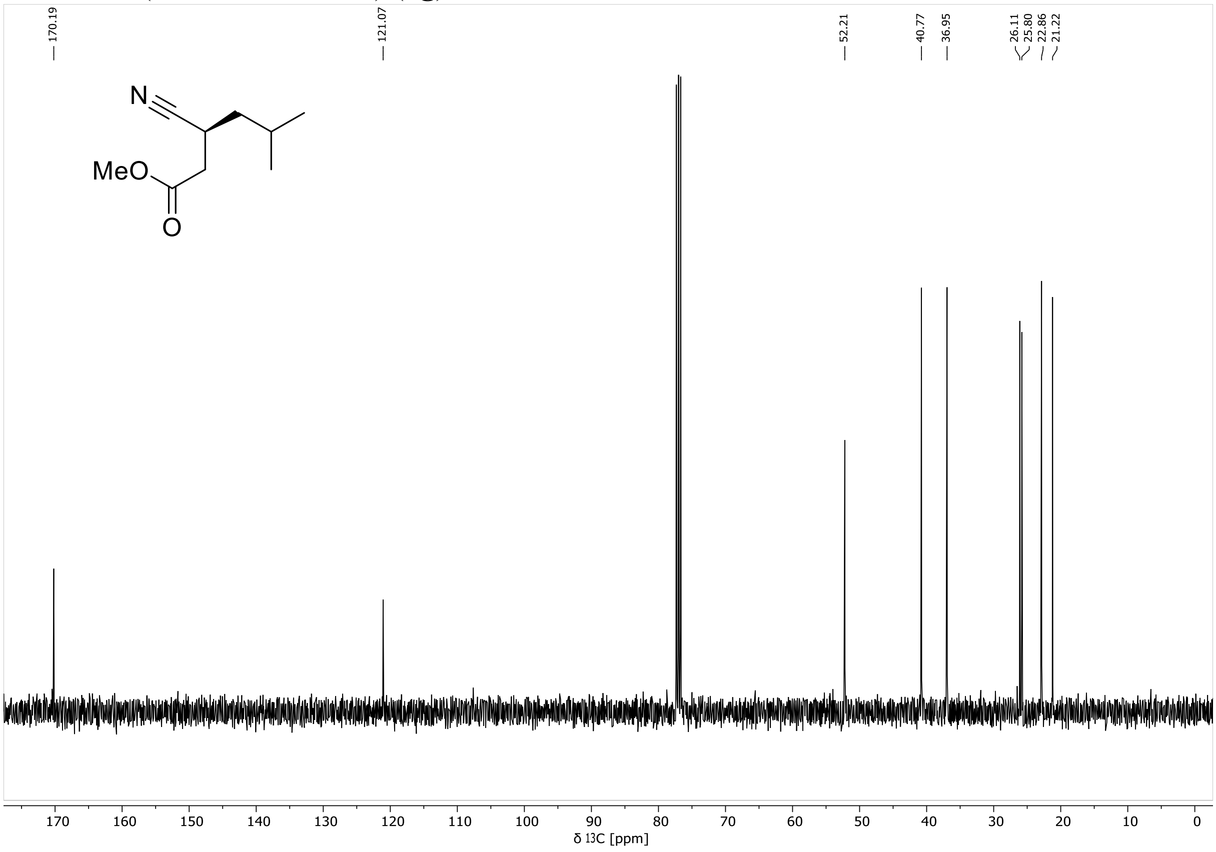

**$^{1}$H NMR** (400 MHz, $CDCl_3$) (*anti*-**5ab**):

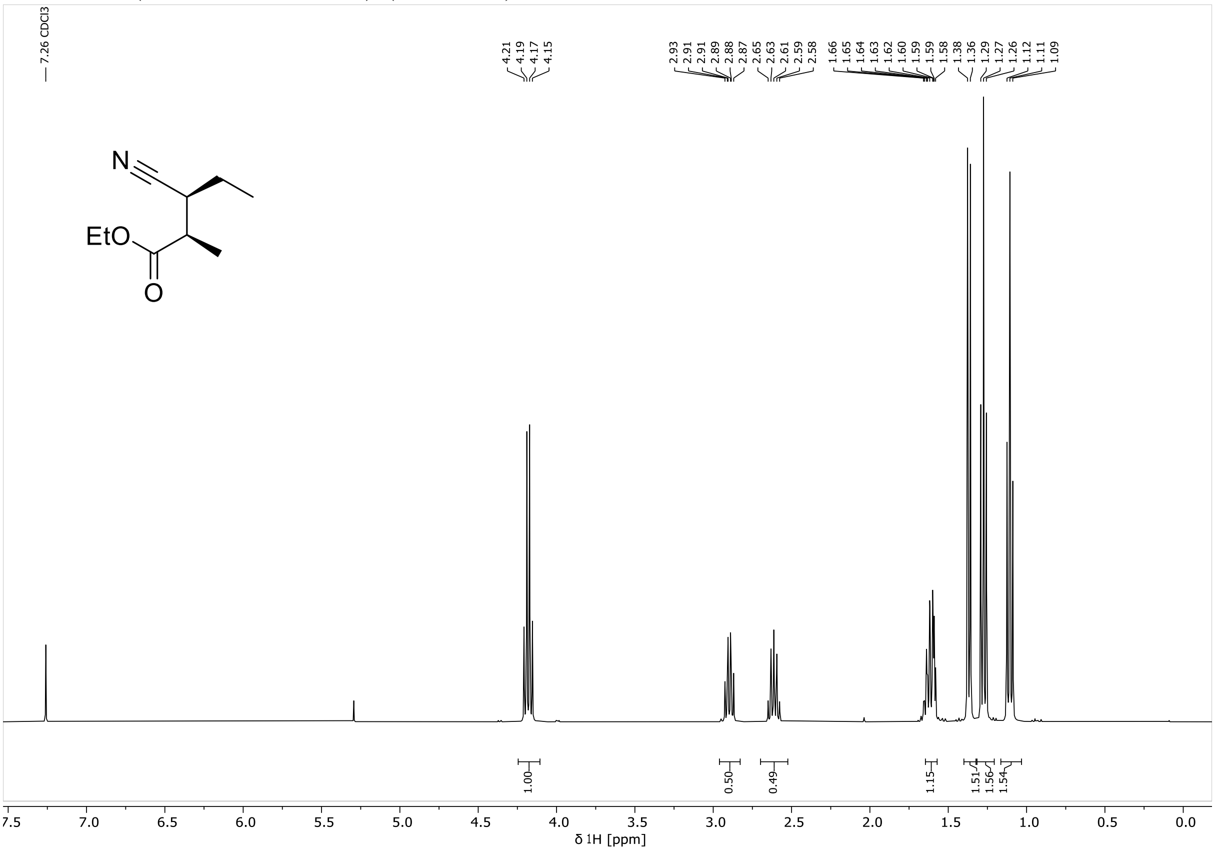


**$^{13}$C NMR** (101 MHz, $CDCl_3$) (*anti*-**5ab**):

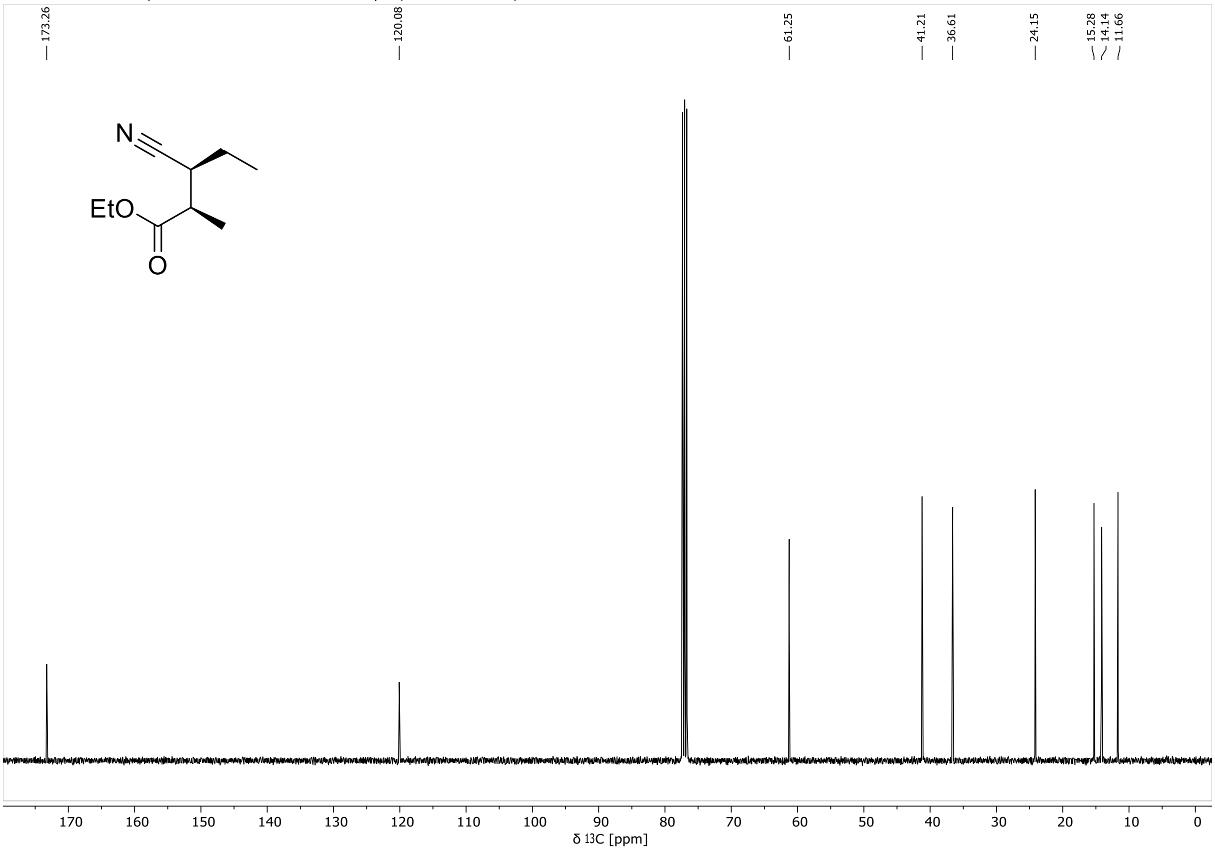

**$^{1}$H NMR** (400 MHz, CDCl$_3$) (*syn*-**5ab**):

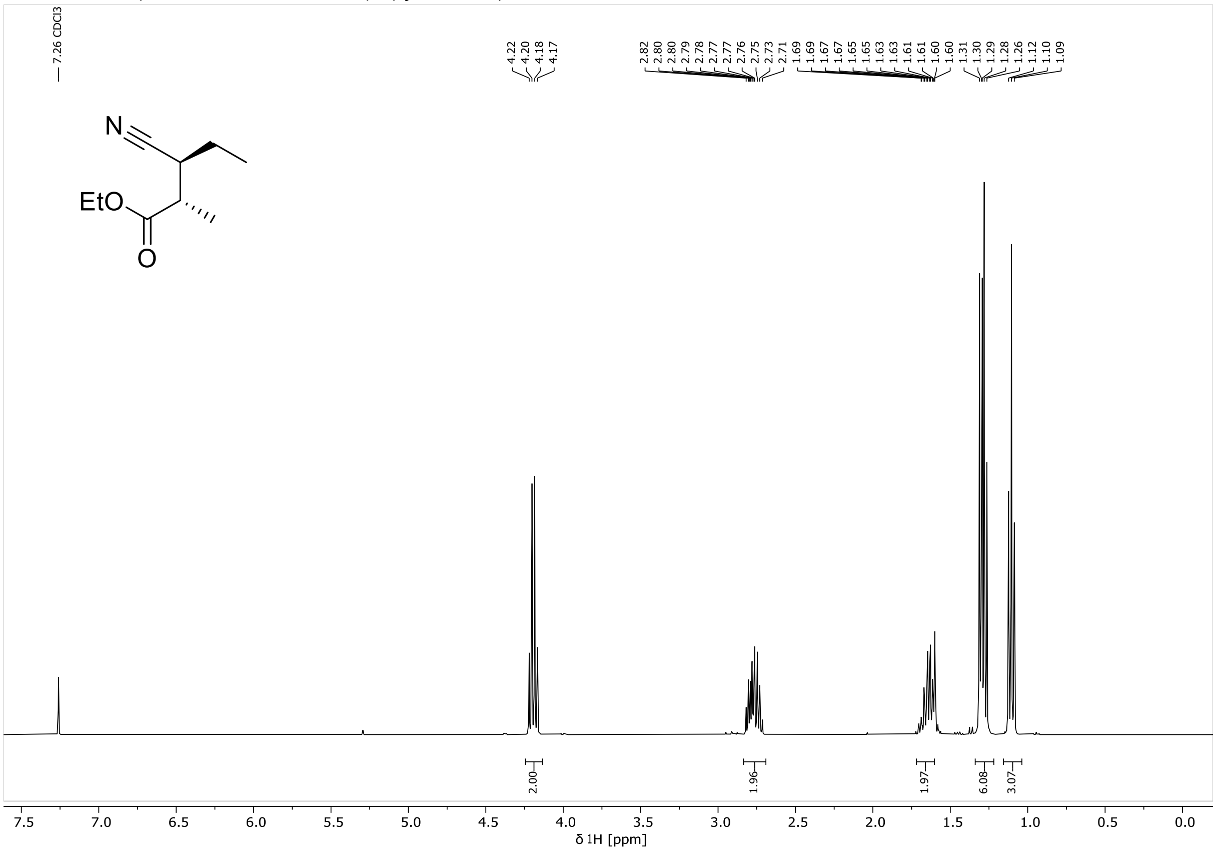

**$^{13}$C NMR** (101 MHz, CDCl$_3$) (*syn*-**5ab**):

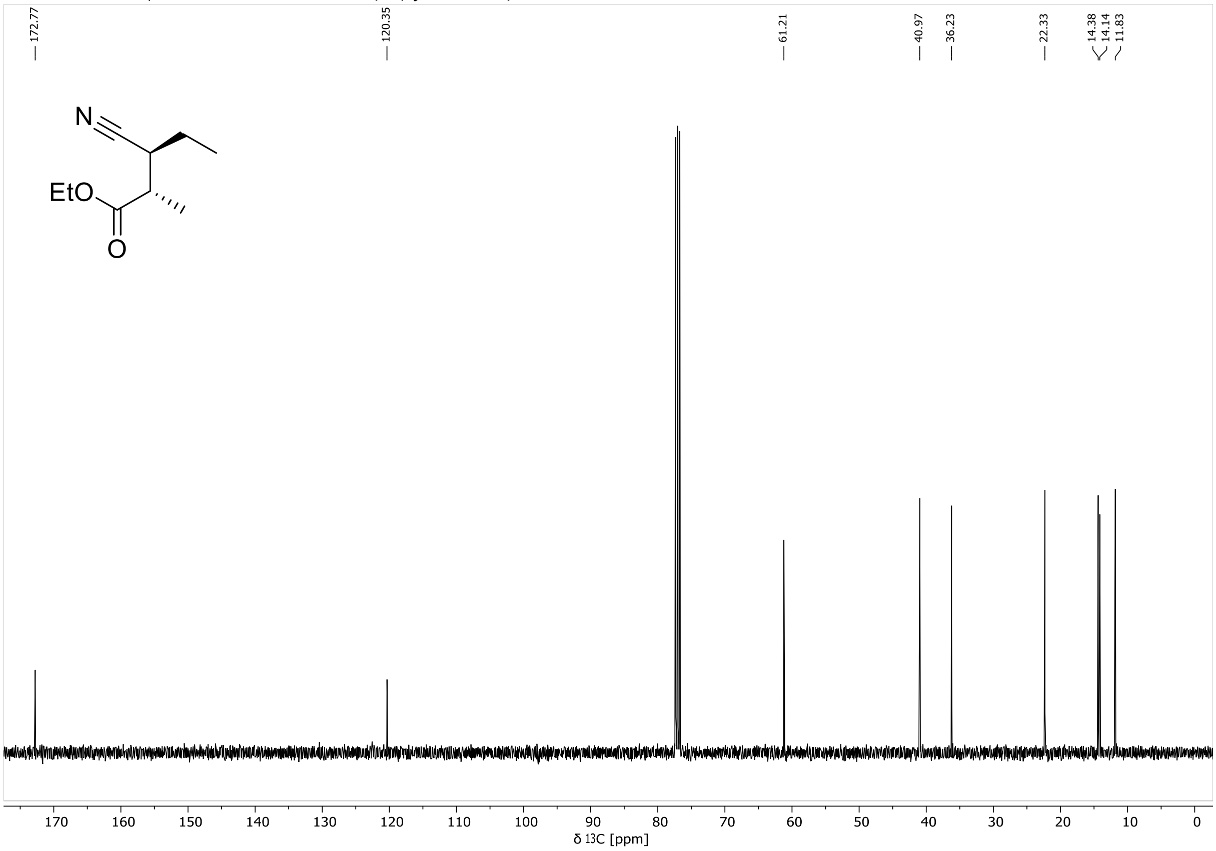

**$^{1}$H NMR** (400 MHz, $CDCl_3$) (**7c**):

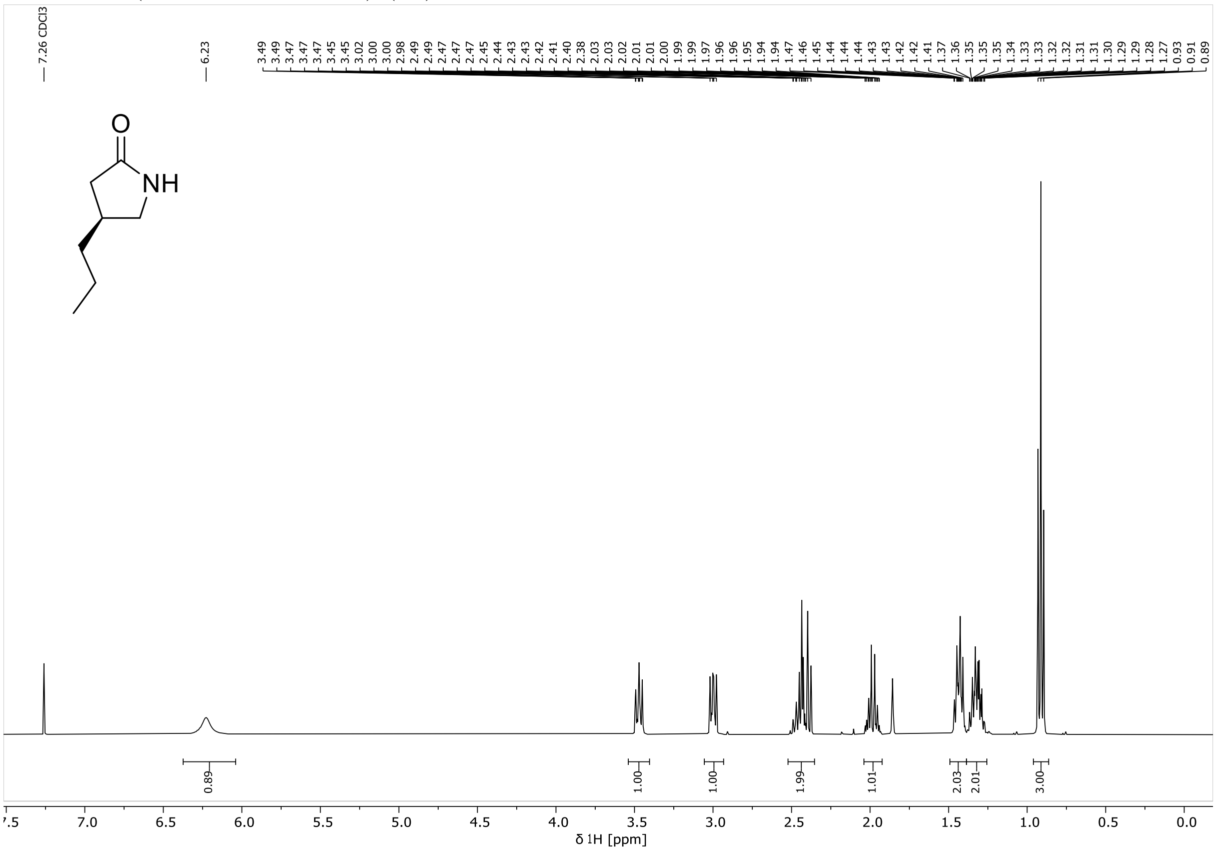

**$^{13}$C NMR** (101 MHz, $CDCl_3$) (**7c**):

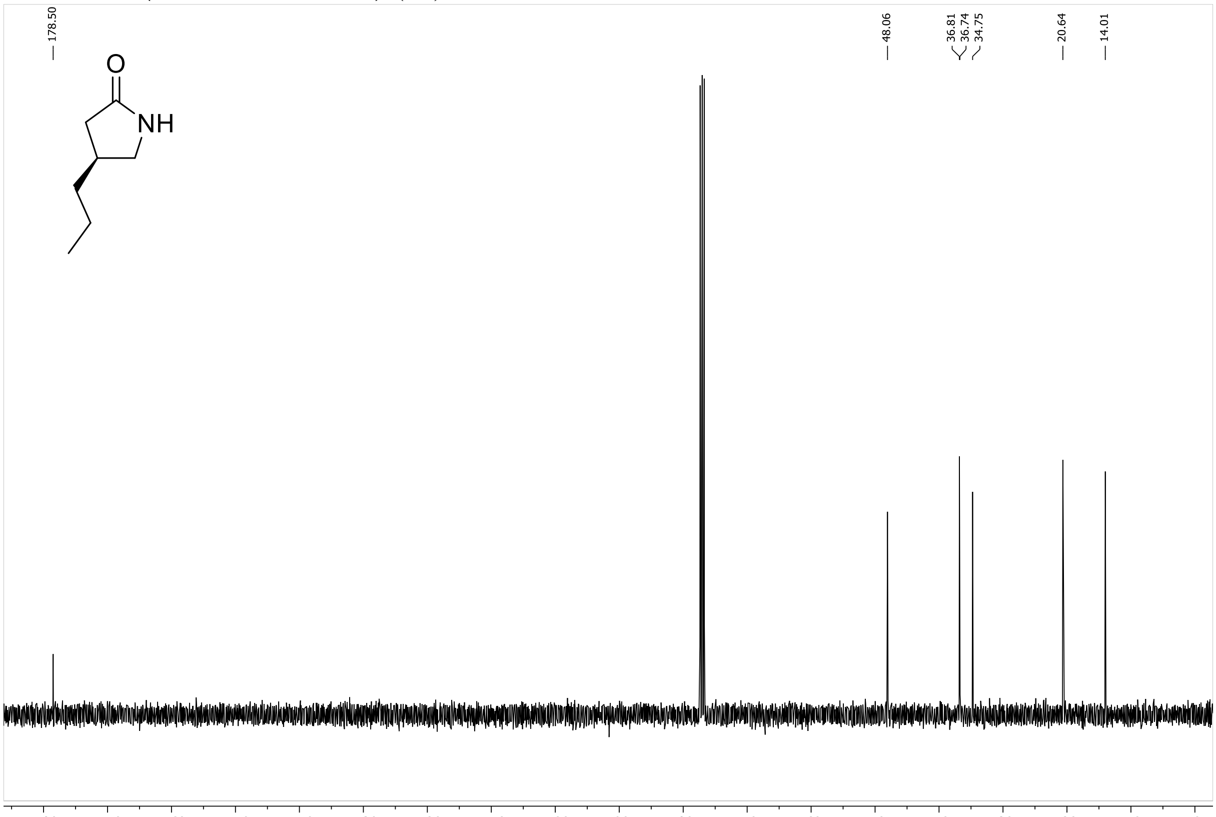

**$^{1}$H NMR** (400 MHz, $D_2O$) (**pregabalin**):

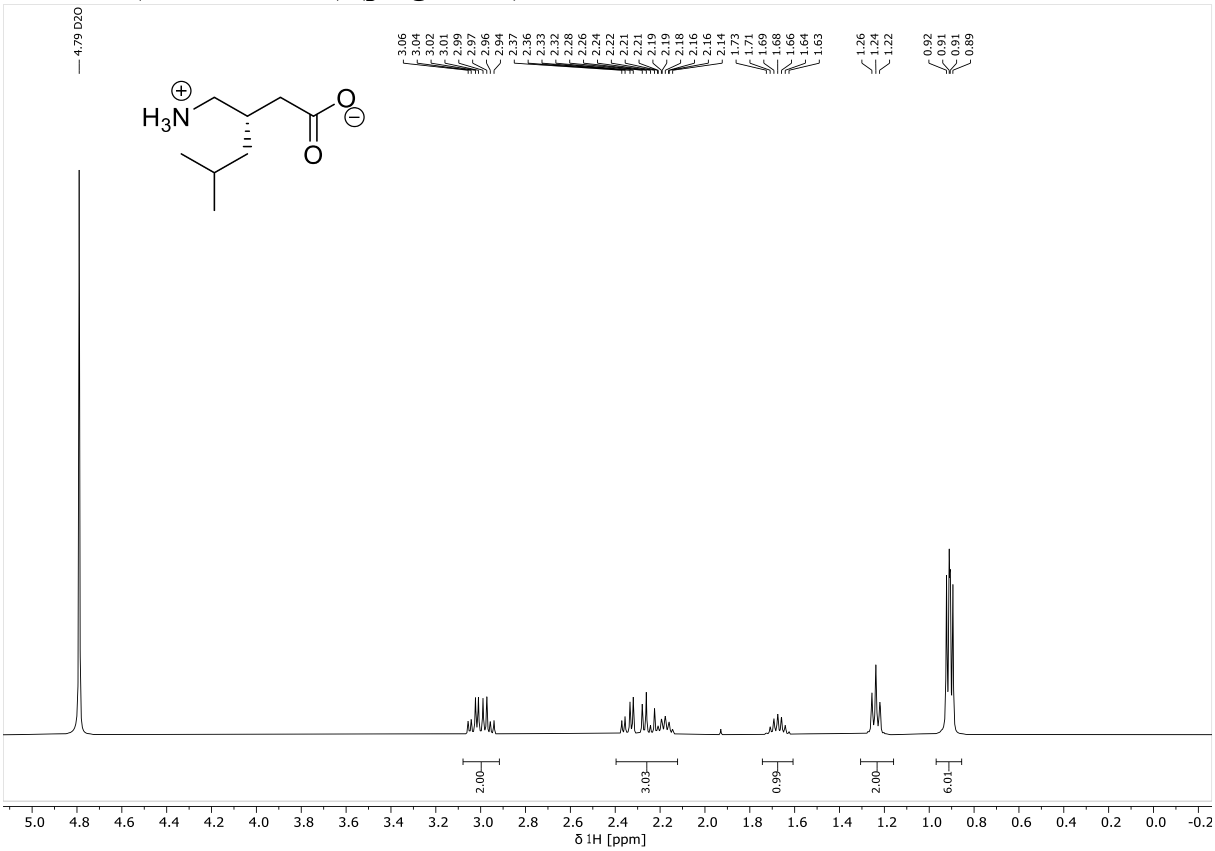

**$^{13}$C NMR** (101 MHz, $D_2O$) (**pregabalin**):

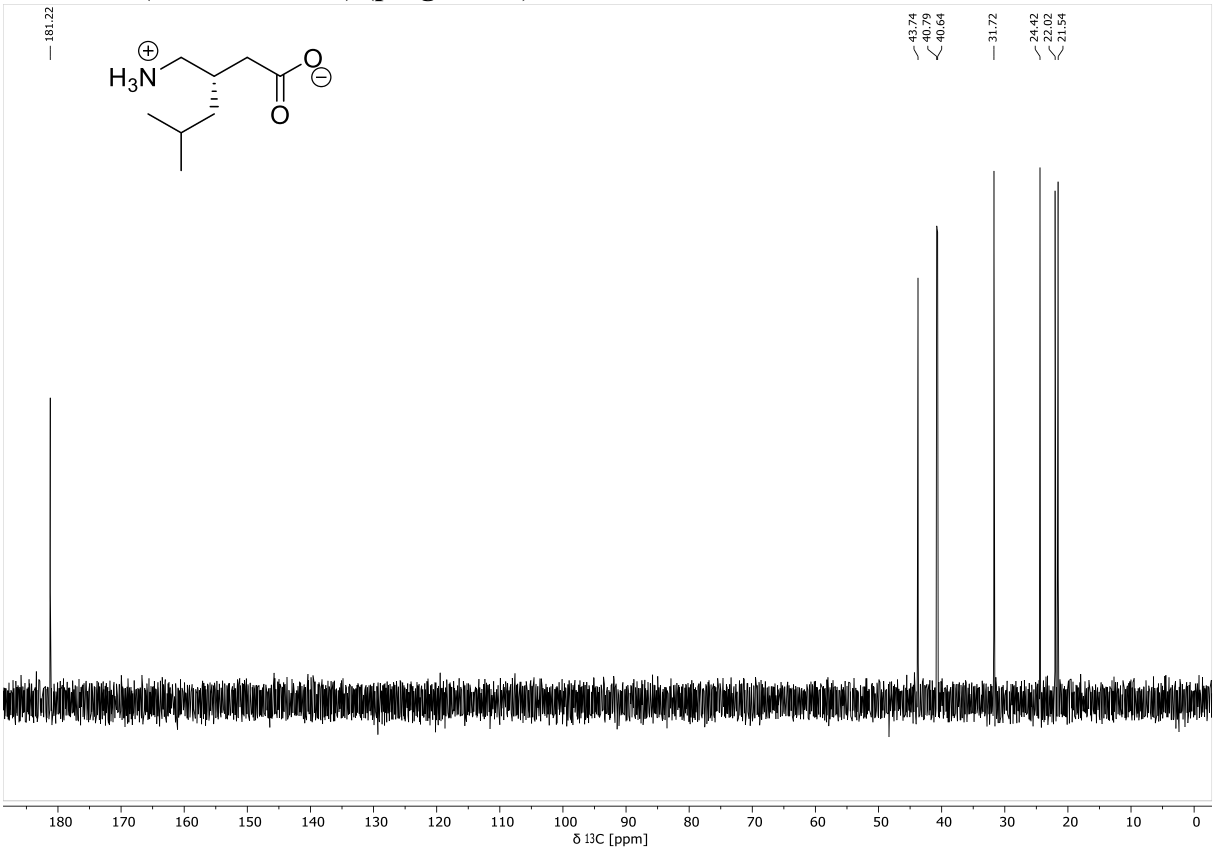

**$^{1}$H NMR** (400 MHz, $CDCl_3$) (*anti*-**10ab**):

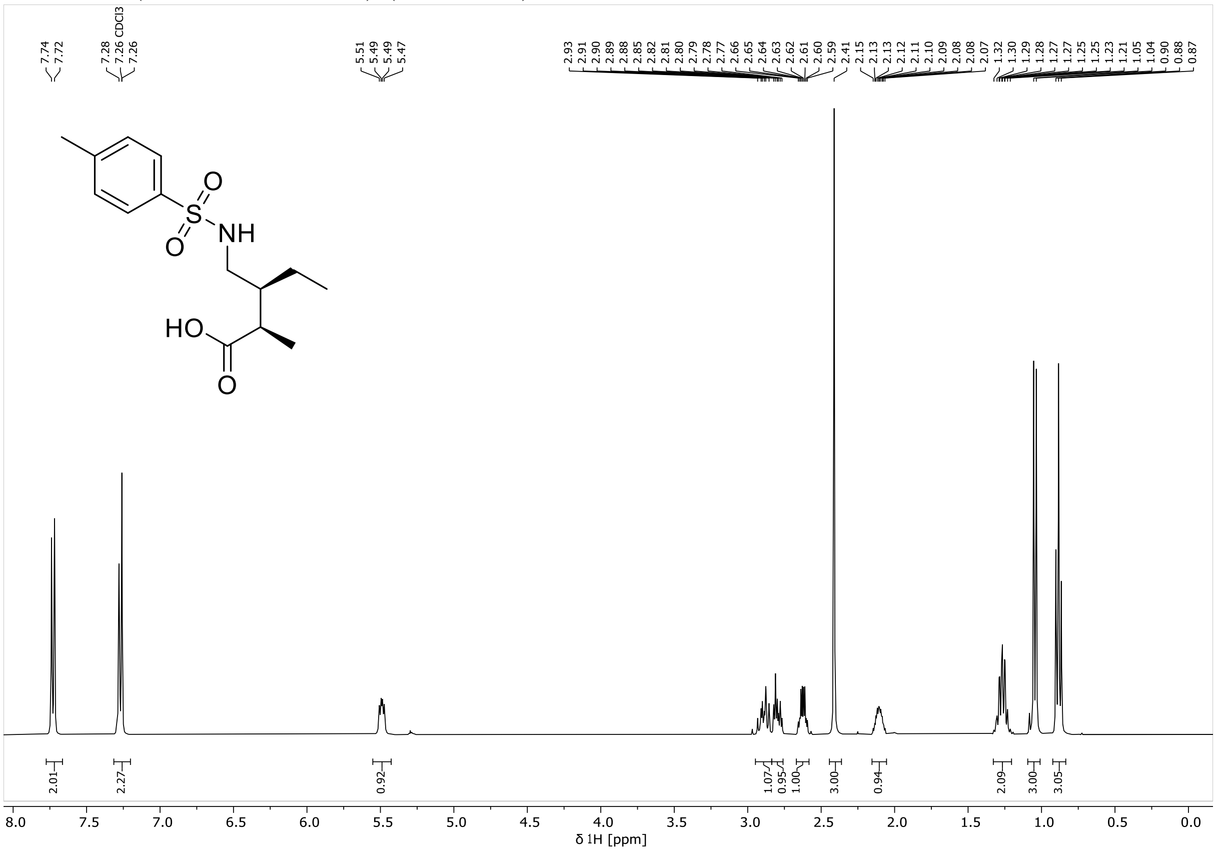

**$^{13}$C NMR** (101 MHz, $CDCl_3$) (*anti*-**10ab**):

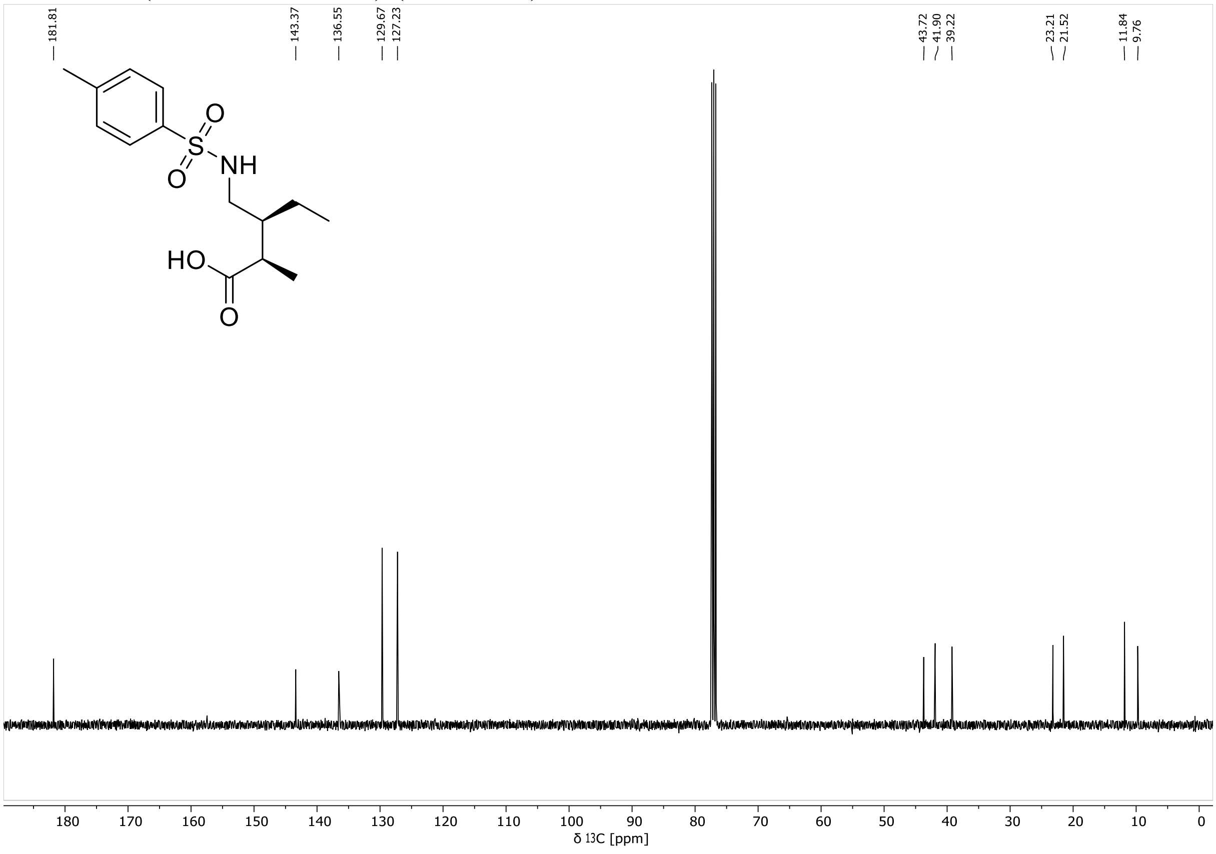

**Bromides and BINOLs:**

**$^1$H NMR** (400 MHz, $CDCl_3$):

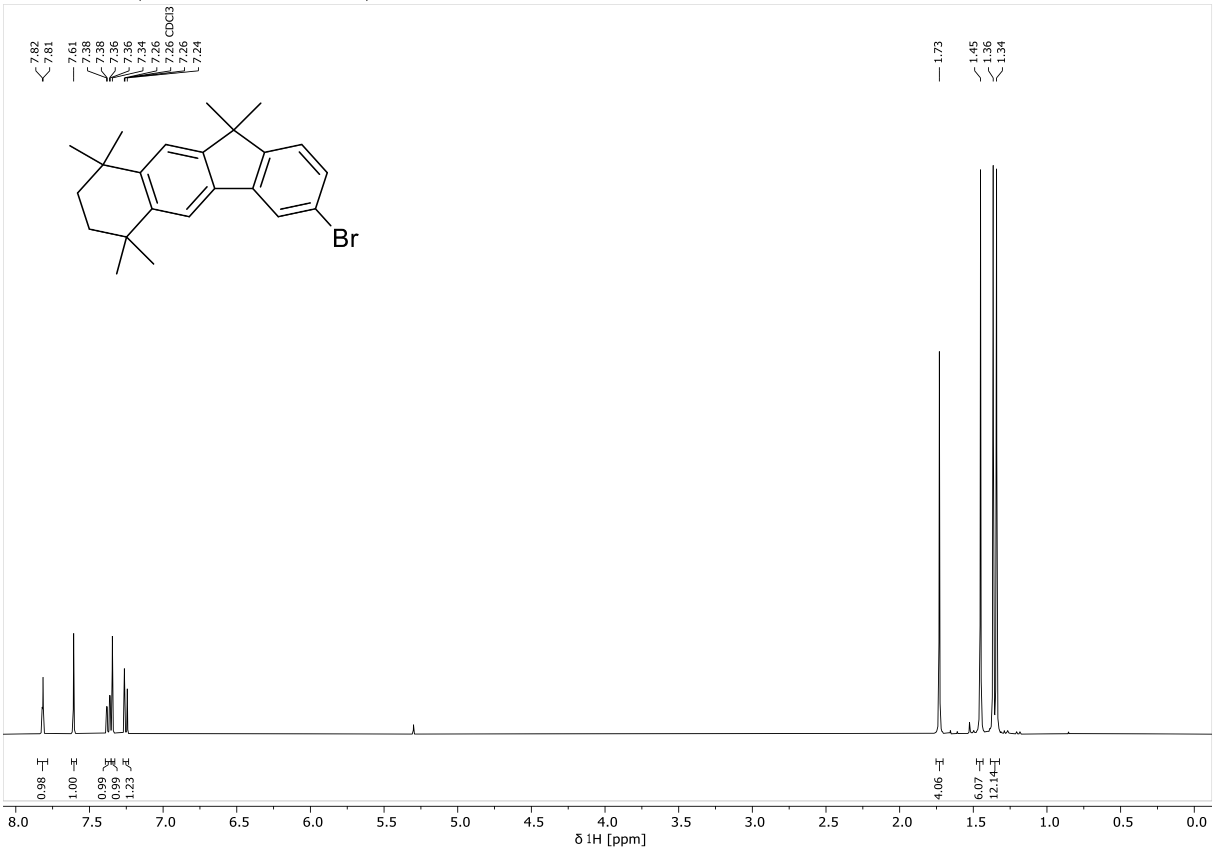

**$^{13}$C NMR** (101 MHz, $CDCl_3$):

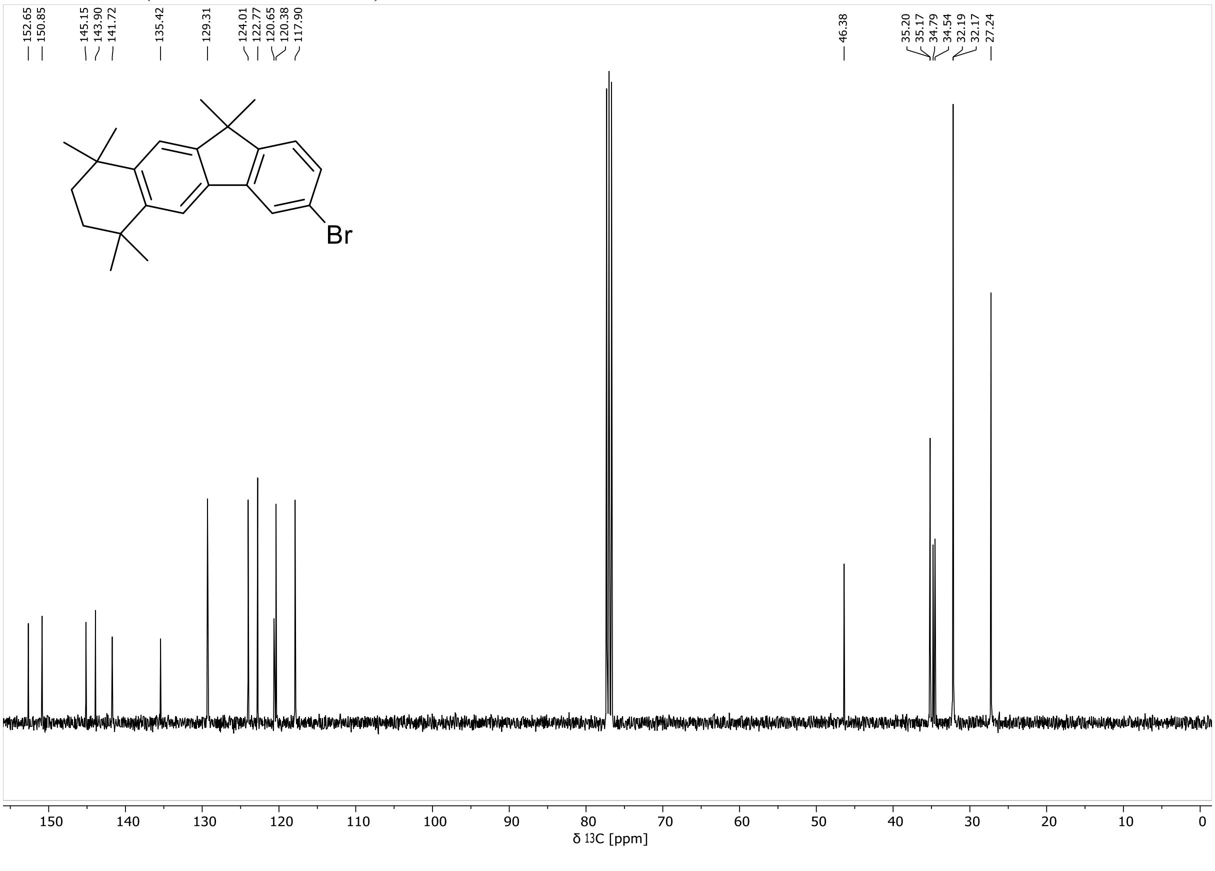

**$^{1}$H NMR** (400 MHz, $CDCl_3$):

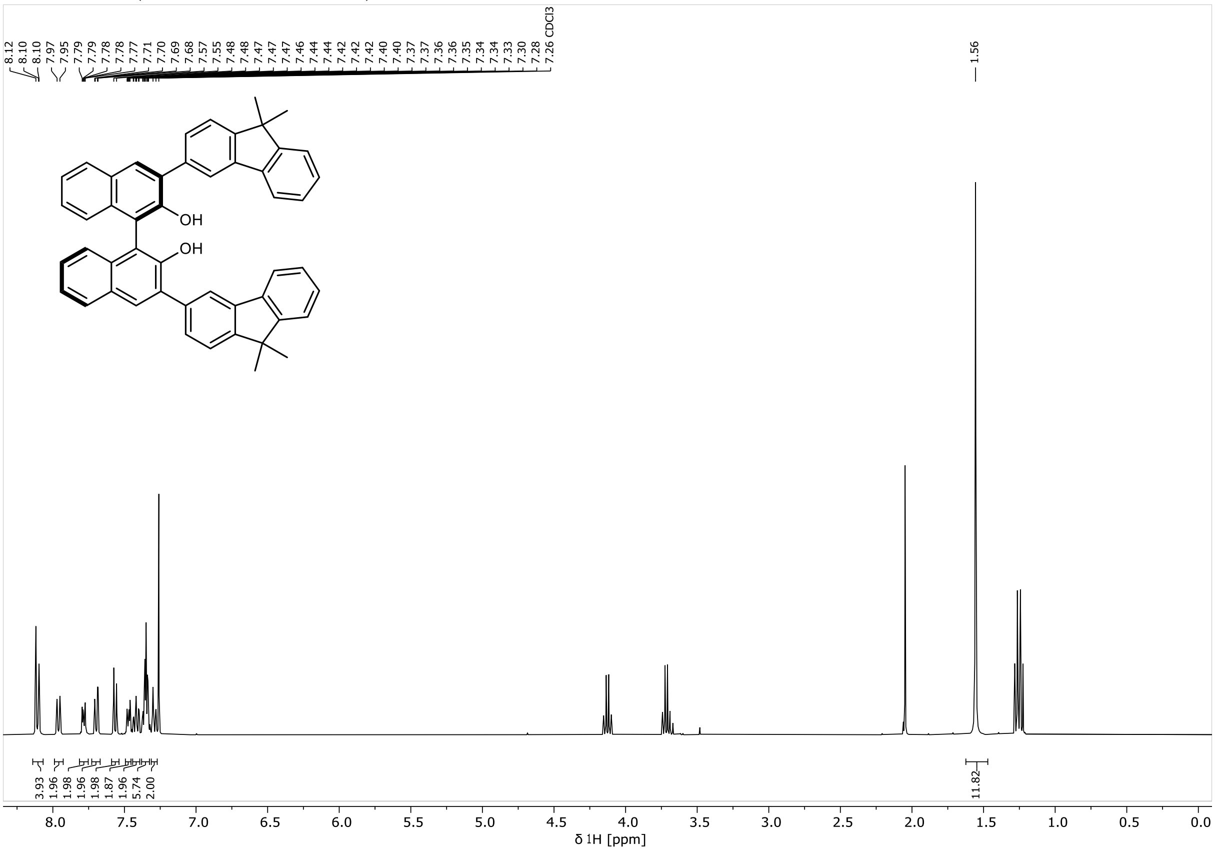


**$^{13}$C NMR** (101 MHz, $CDCl_3$):

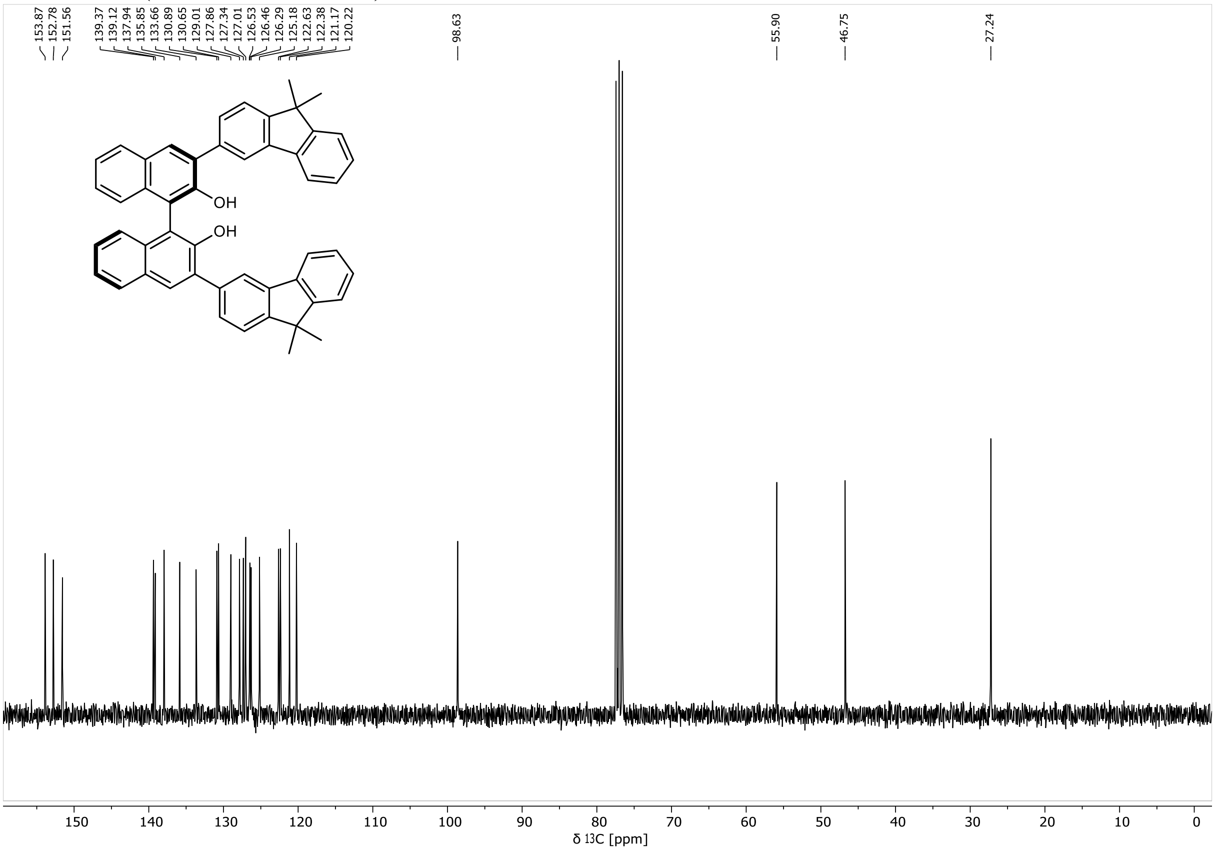

**$^1$H NMR** (400 MHz, $CDCl_3$):

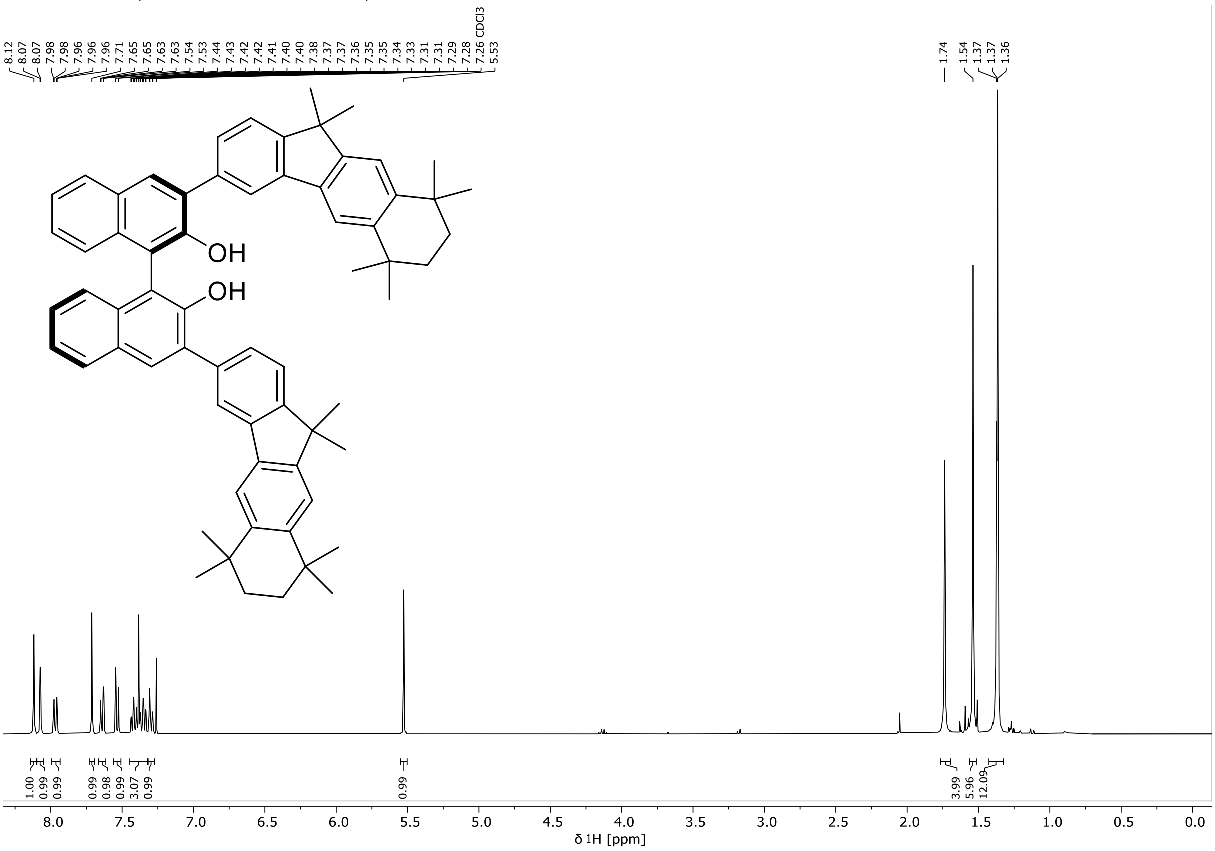

**$^{13}$C NMR** (101 MHz, $CDCl_3$):

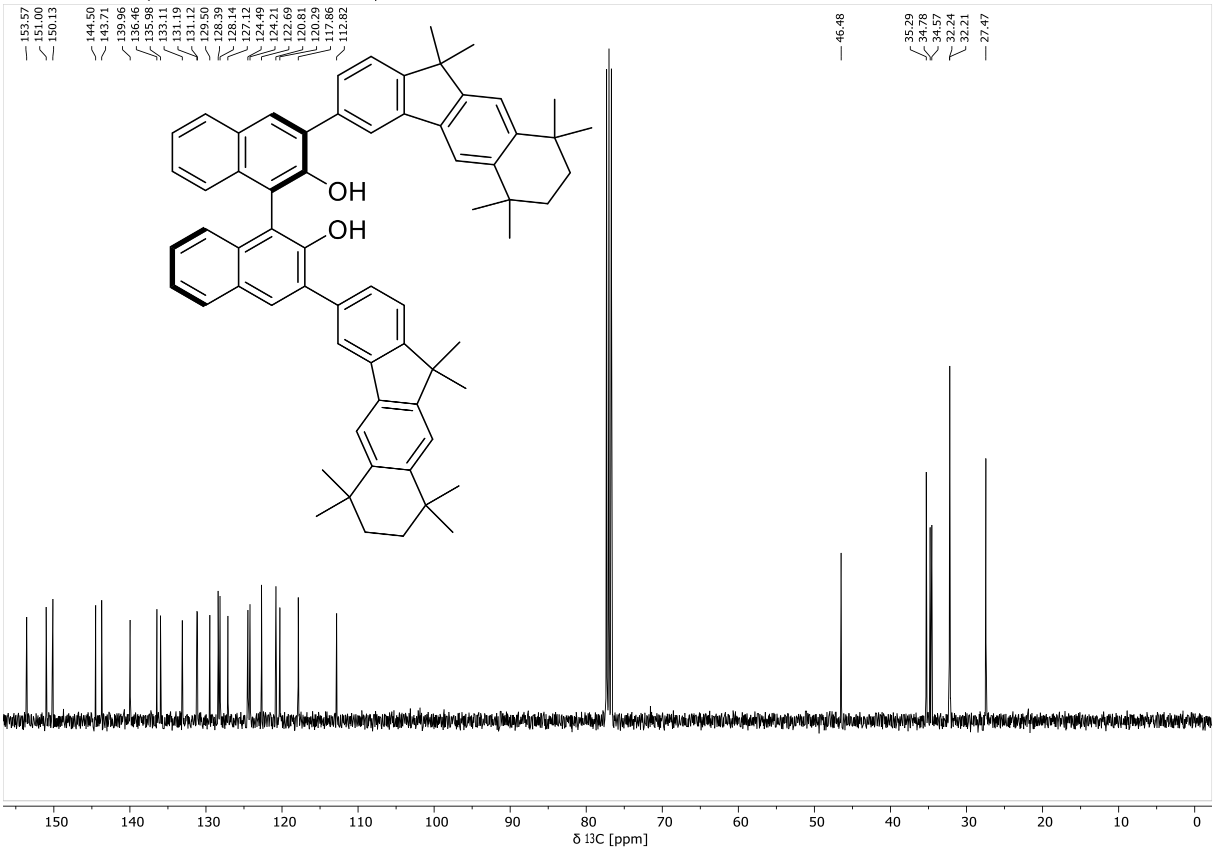

**$^{1}$H NMR** (400 MHz, $CDCl_3$):

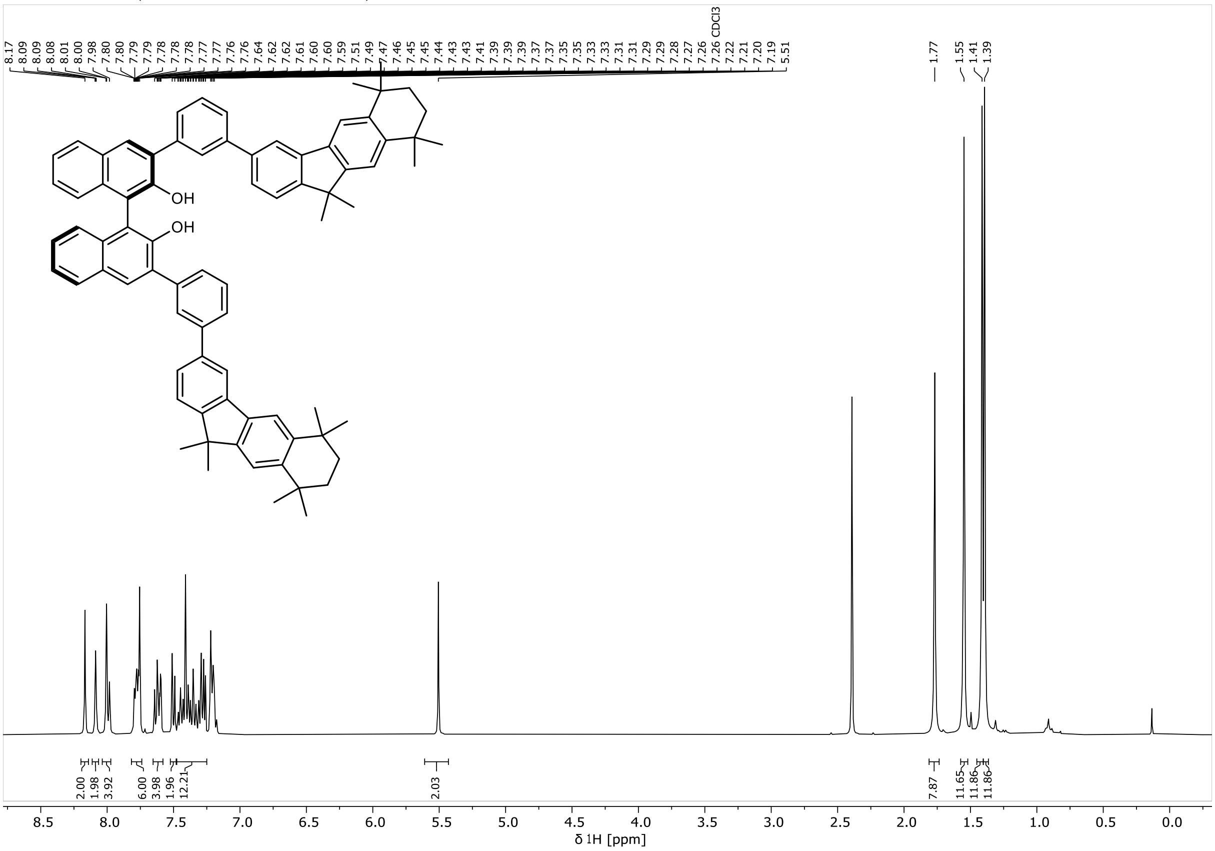

**$^{13}$C NMR** (101 MHz, $CDCl_3$):

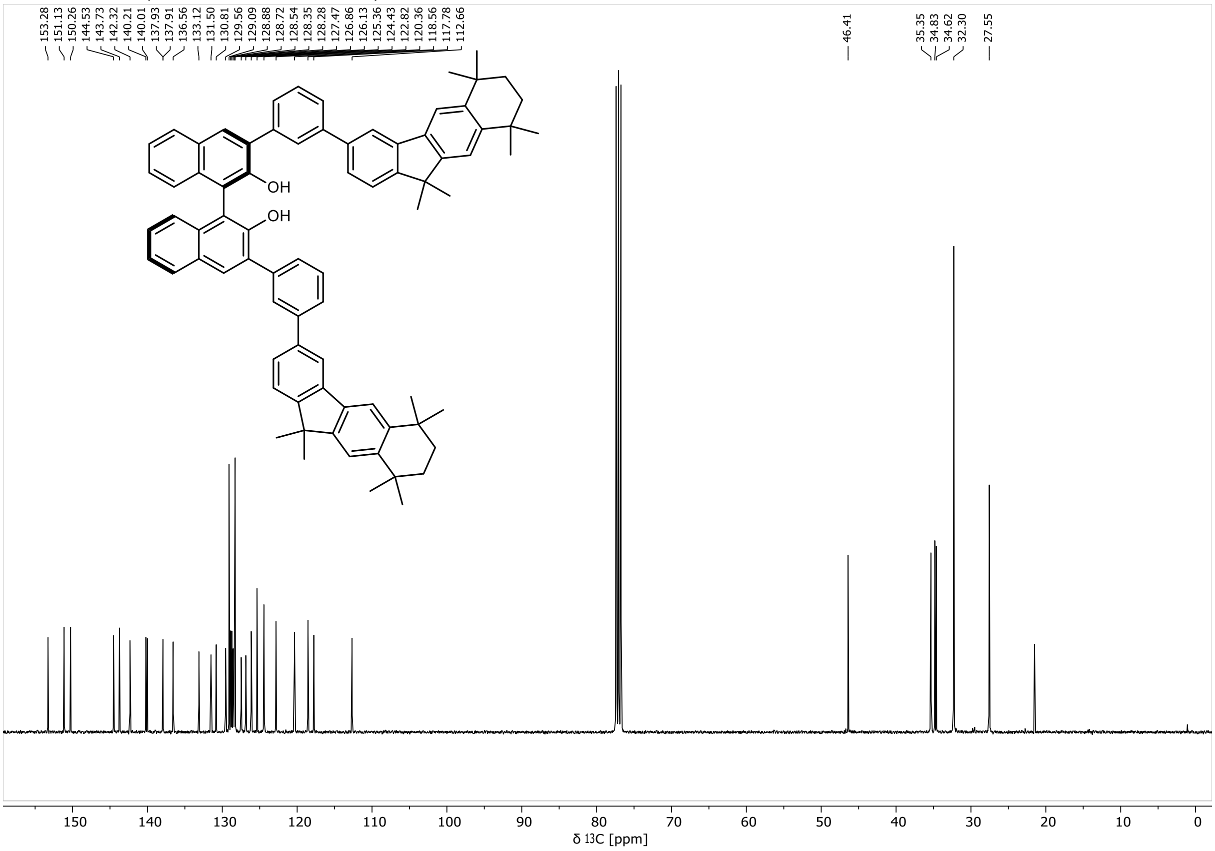

**IDPis:**

$^{1}$**H NMR** (400 MHz, $CDCl_3$) (**3f**):

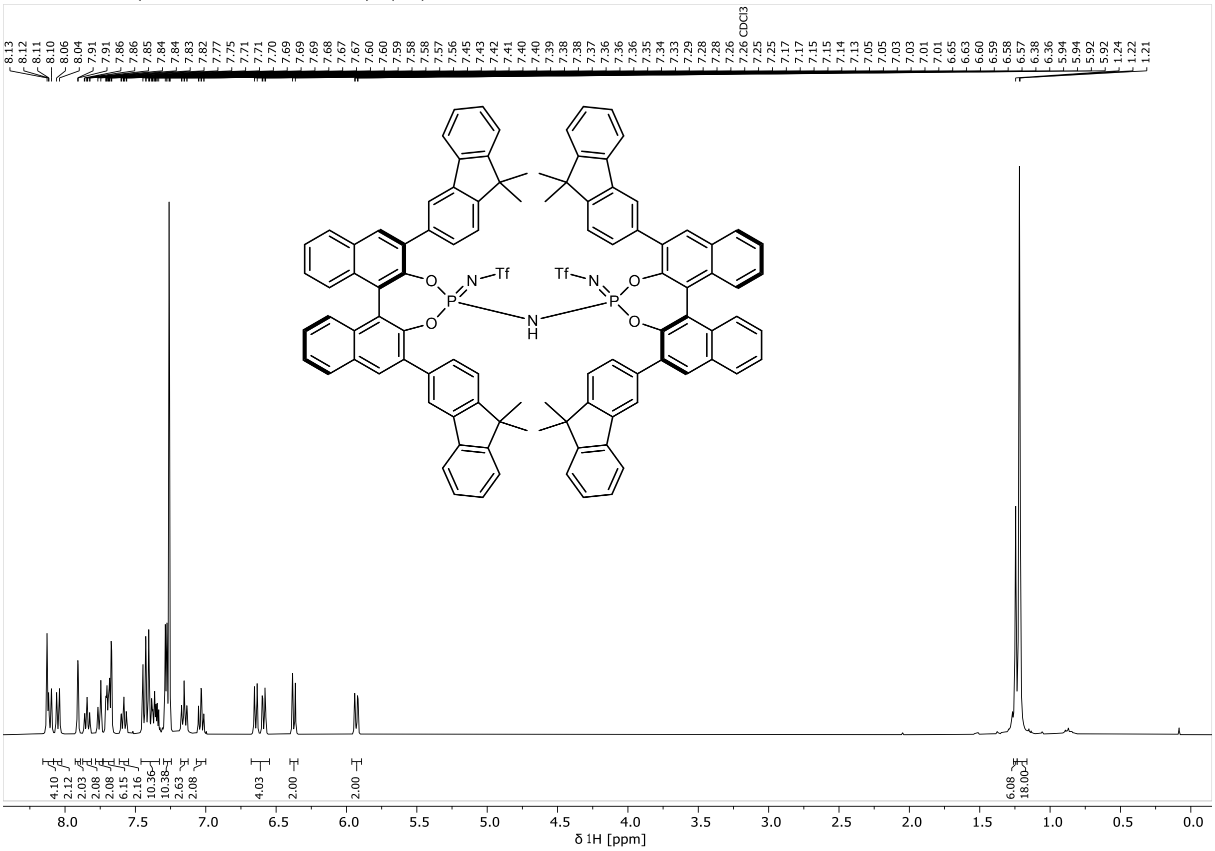


$^{13}$**C NMR** (101 MHz, $CDCl_3$) (**3f**):

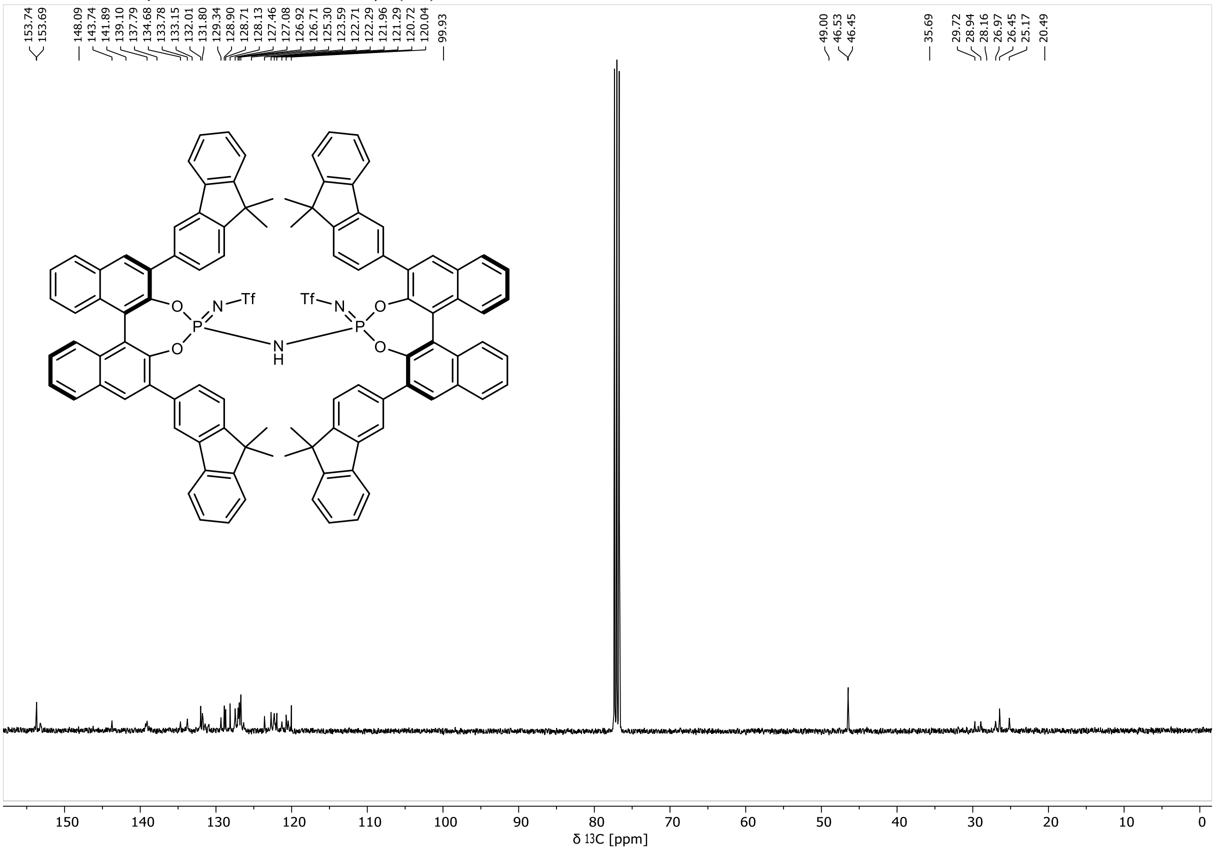

**$^{19}$F NMR** (377 MHz, $CDCl_3$) (**3f**):

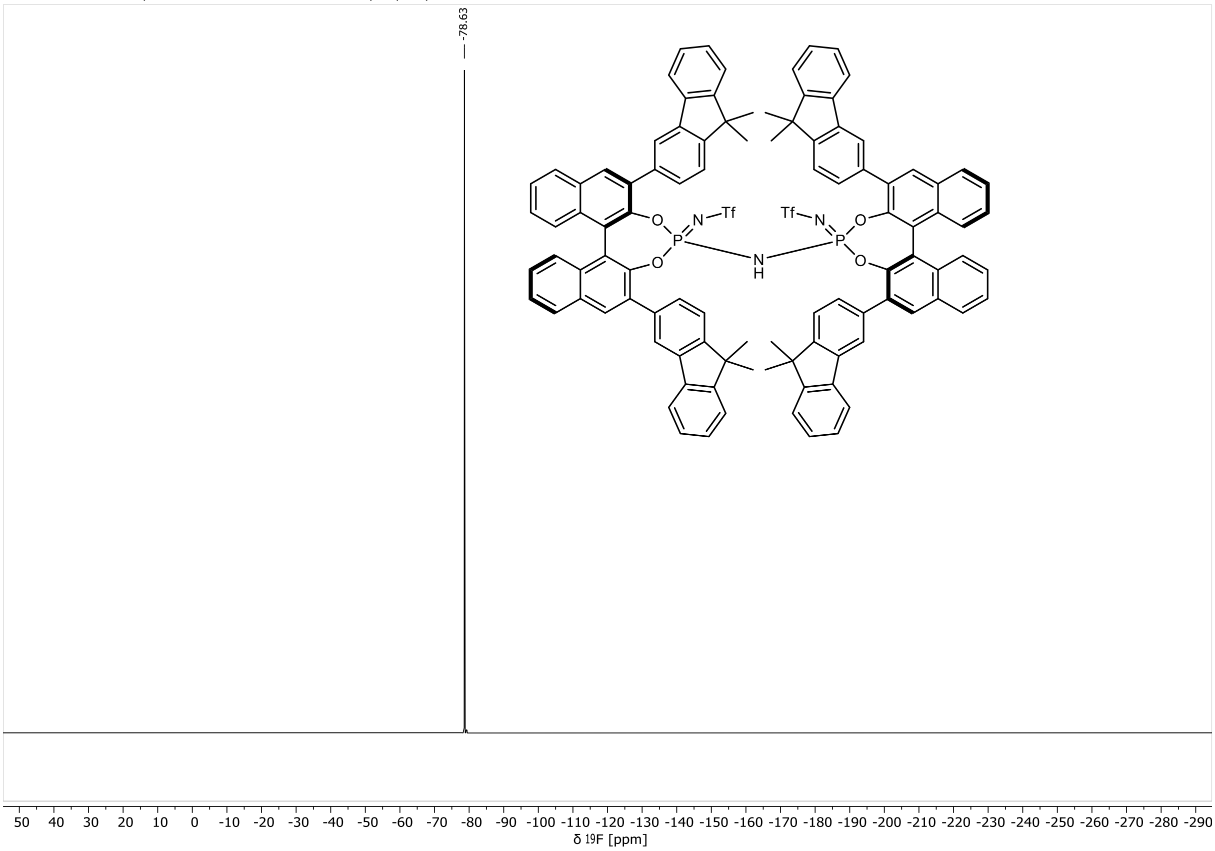


**$^{31}$P NMR** (162 MHz, $CDCl_3$) (**3f**):

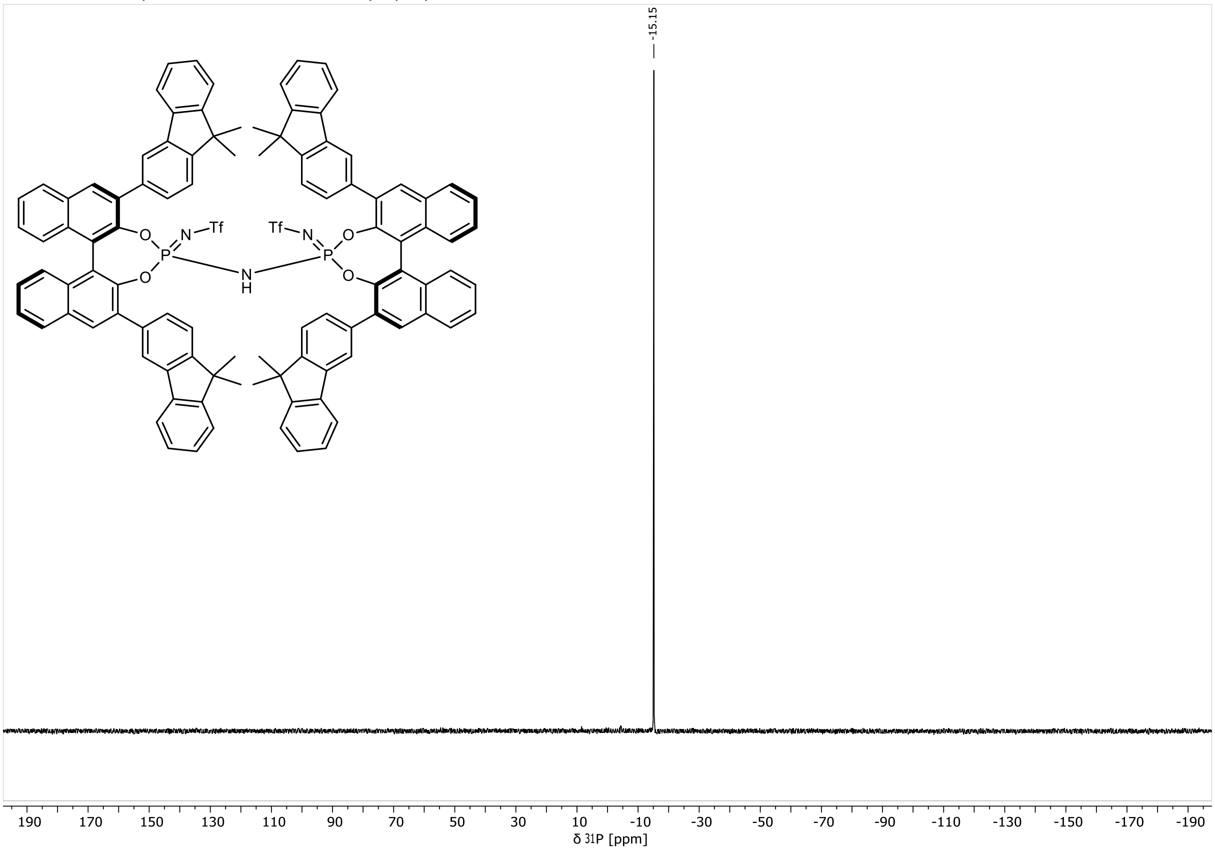

**$^{1}$H NMR** (400 MHz, $CDCl_3$) (**3g**):

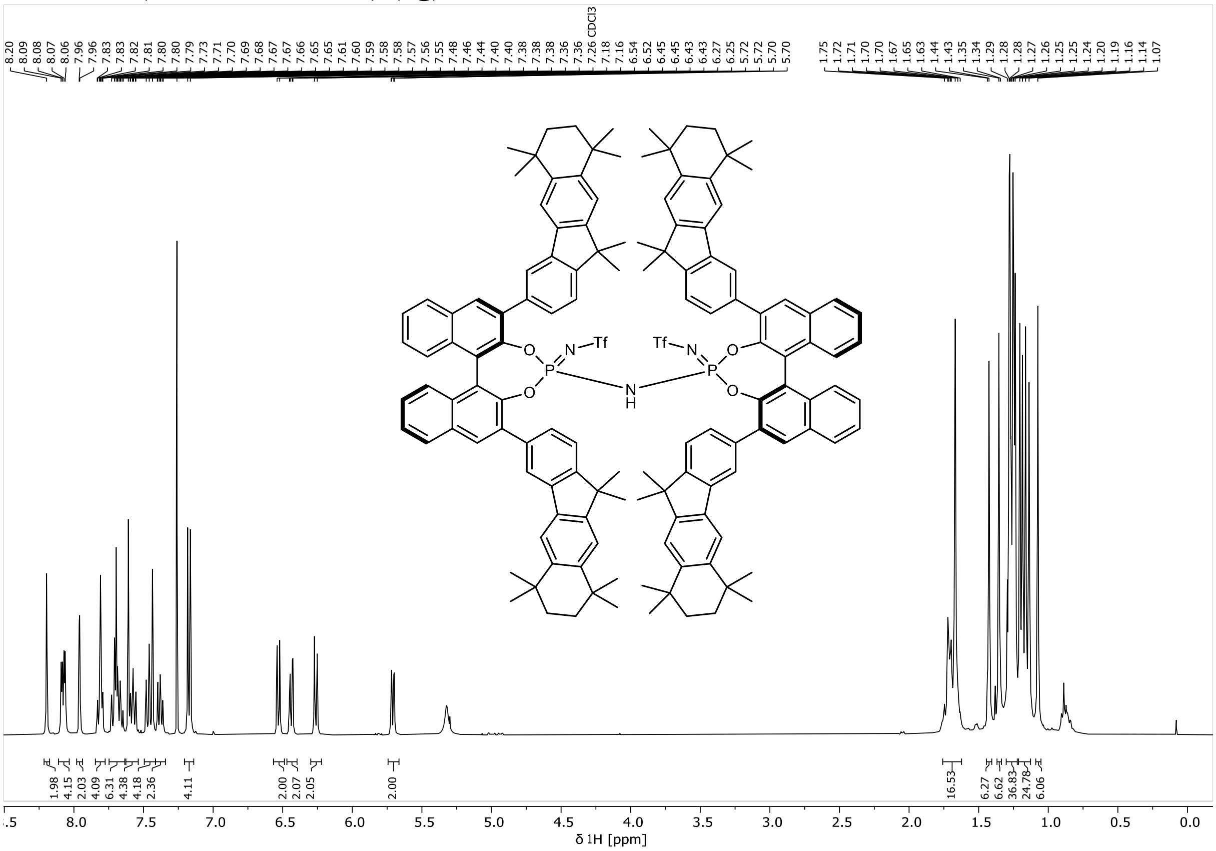


**$^{13}$C NMR** (101 MHz, $CDCl_3$) (**3g**):

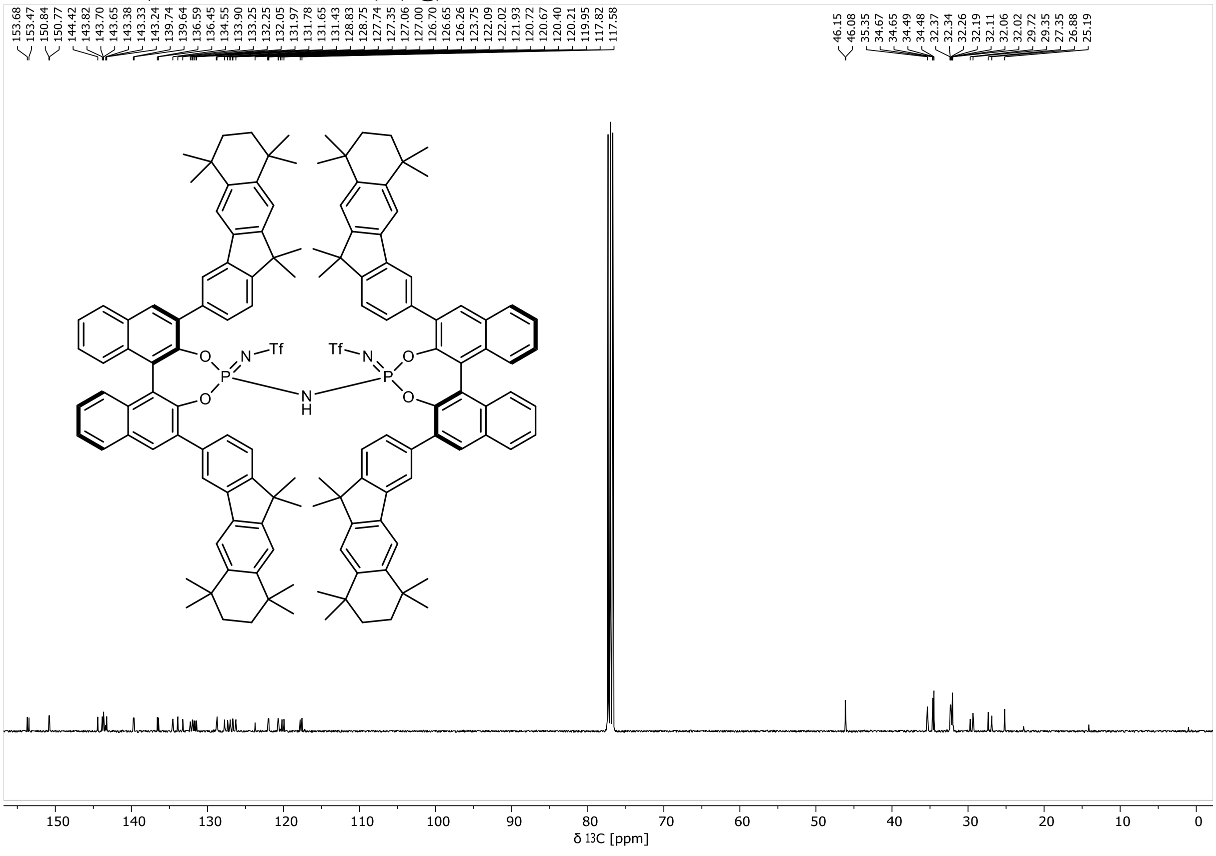

**$^{19}$F NMR** (377 MHz, $CDCl_3$) (**3g**):

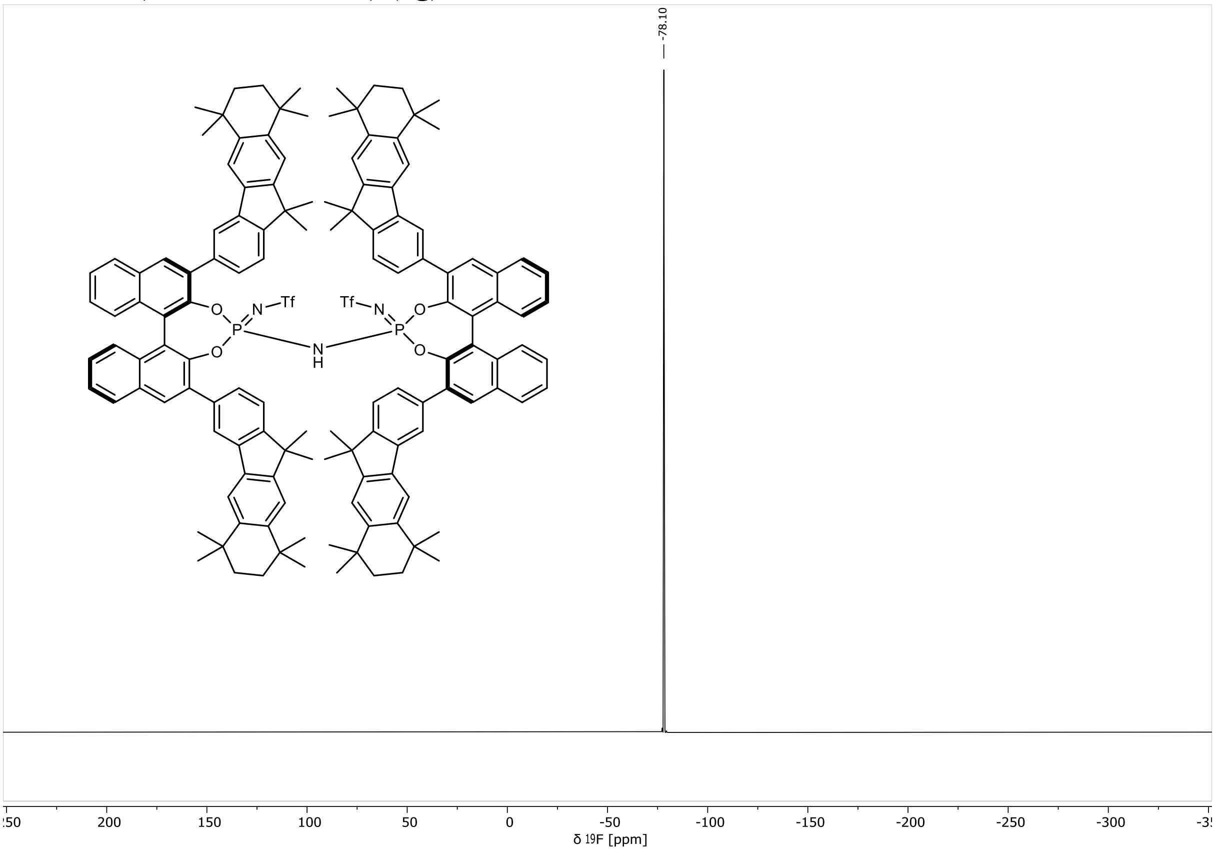


**$^{31}$P NMR** (162 MHz, $CDCl_3$) (**3g**):

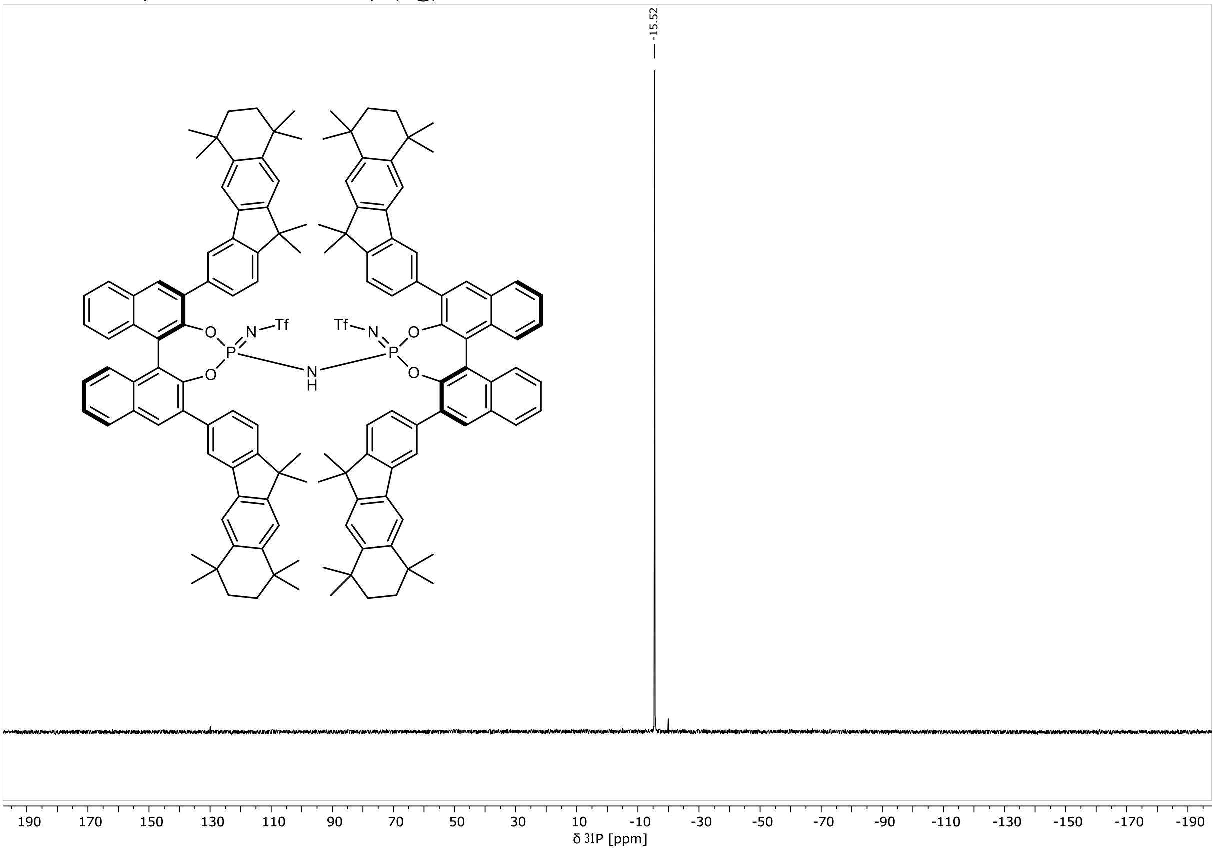

**$^{1}$H NMR** (400 MHz, $CDCl_3$) (**3h**):

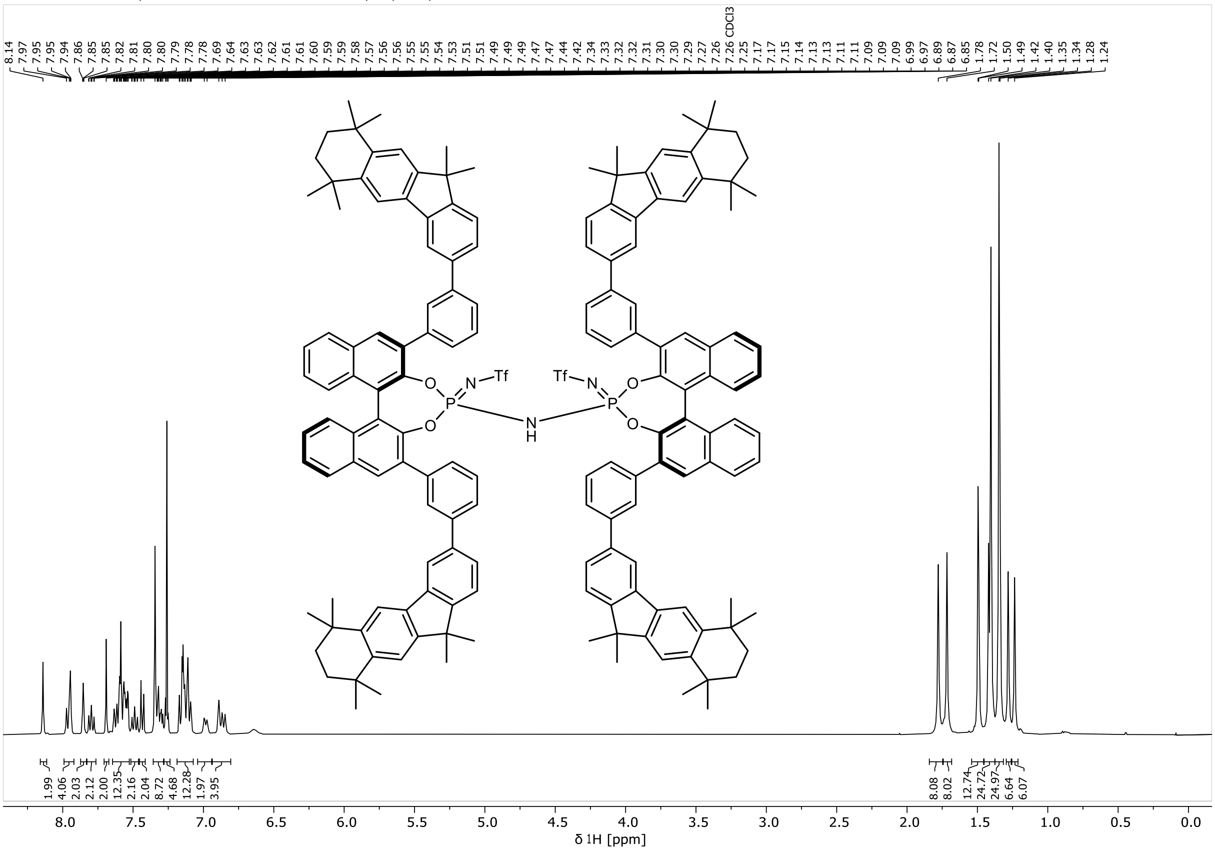

**$^{19}$F NMR** (377 MHz, $CDCl_3$) (**3h**):

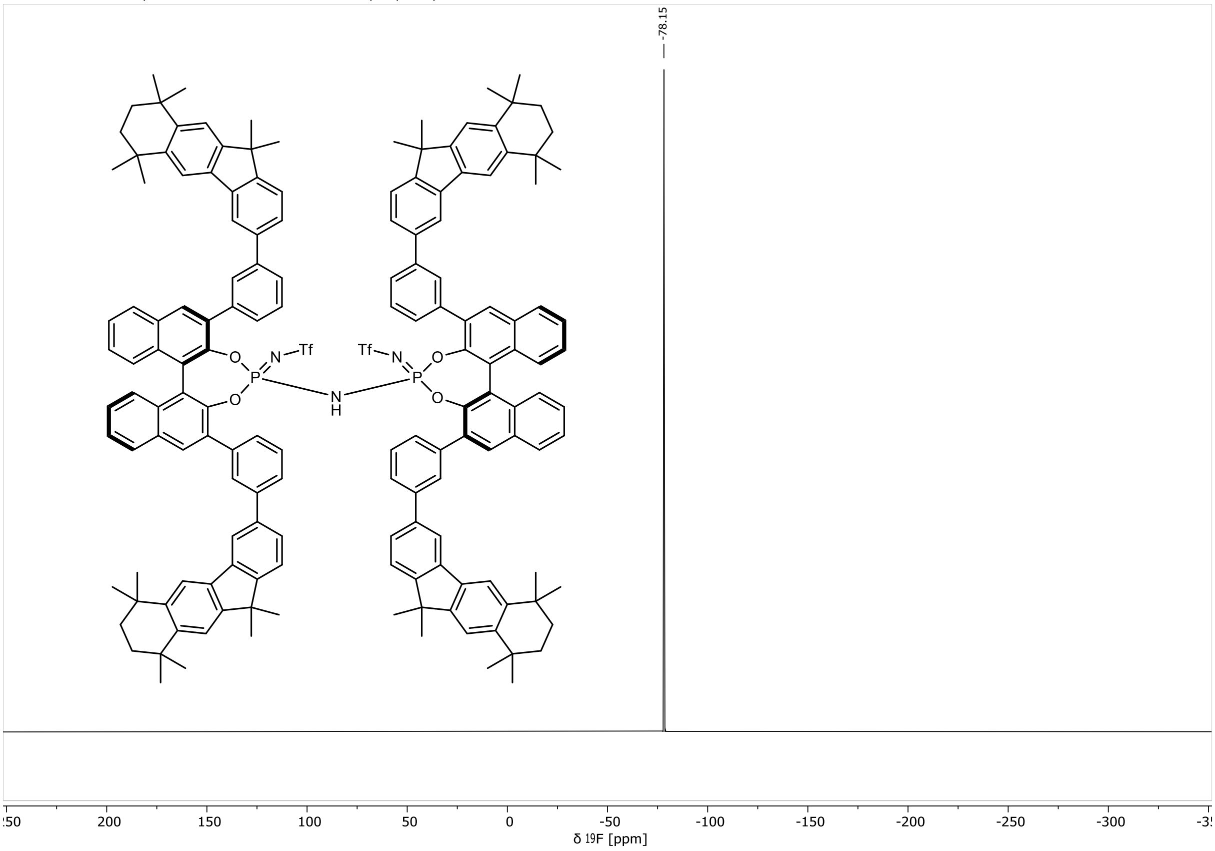

**$^{31}$P NMR** (162 MHz, $CDCl_3$) (**3h**):

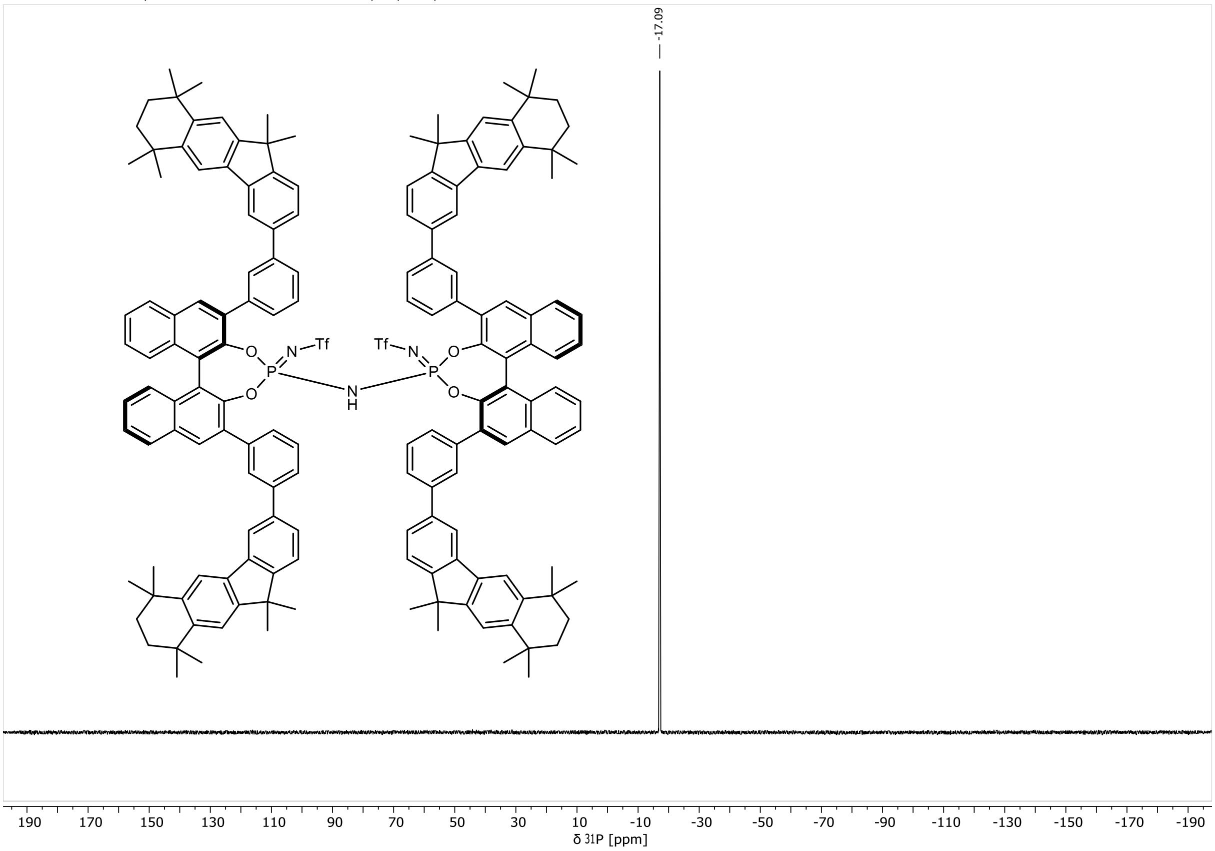


**$^{13}$C NMR** (101 MHz, $CDCl_3$) (**3h**):

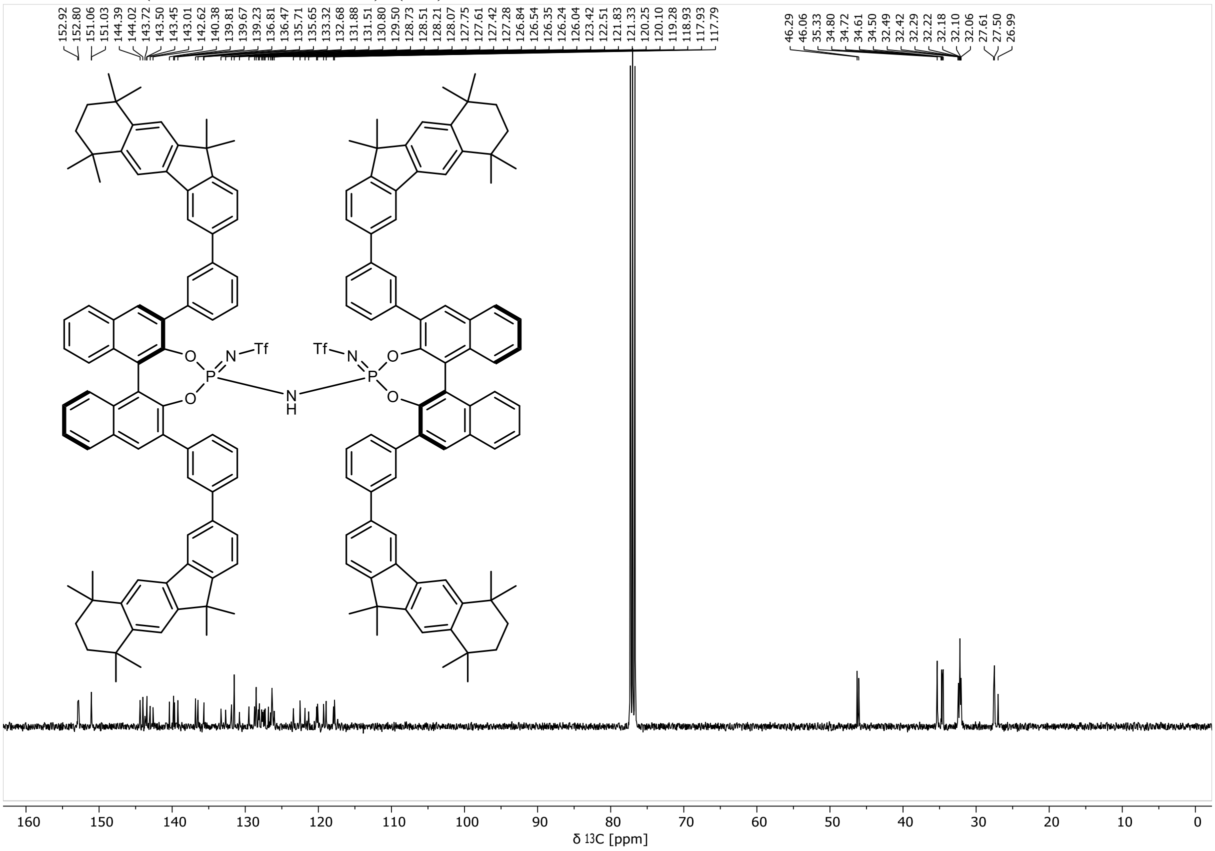

# HPLC Chromatograms

**4a:** Chiralpak IA, hexane/iPrOH 99/1, flow rate = 1.0 mL/min, λ = 248 nm

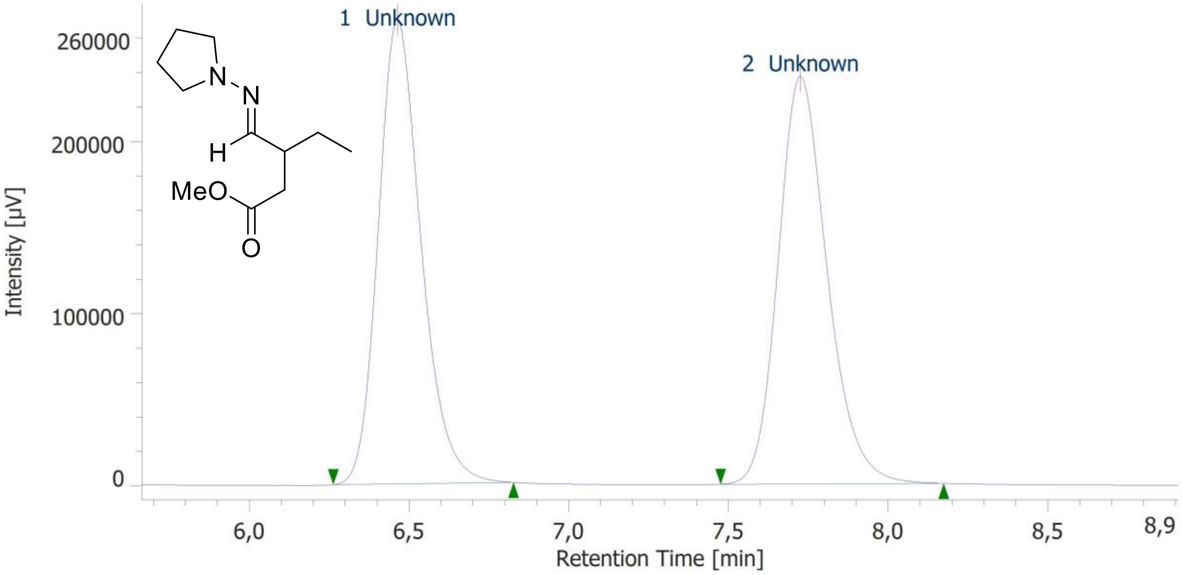


| # | Peak Name | CH | tR [min] | Area [µV·sec] | Height [µV] | Area% | Height% | Quantity | NTP | Resolution | Symmetry Factor | Warning |
|---|---|---|---|---|---|---|---|---|---|---|---|---|
| 1 | Unknown | 11 | 6,463 | 2508659 | 269524 | 49,830 | 53,245 | N/A | 11638 | 4,921 | 1,236 | |
| 2 | Unknown | 11 | 7,725 | 2525785 | 236675 | 50,170 | 46,755 | N/A | 12671 | N/A | 1,209 | |

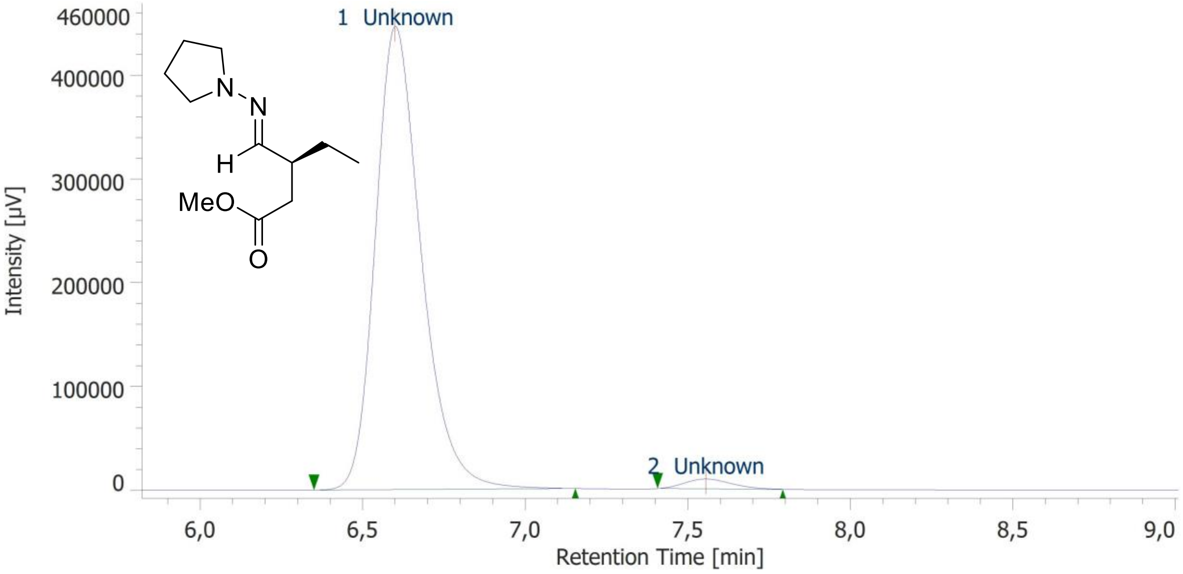


| # | Peak Name | CH | tR [min] | Area [µV·sec] | Height [µV] | Area% | Height% | Quantity | NTP | Resolution | Symmetry Factor | Warning |
|---|---|---|---|---|---|---|---|---|---|---|---|---|
| 1 | Unknown | 11 | 6,600 | 4373052 | 446275 | 97,873 | 97,913 | N/A | 11171 | 3,698 | 1,285 | |
| 2 | Unknown | 11 | 7,555 | 95059 | 9513 | 2,127 | 2,087 | N/A | 12706 | N/A | 1,216 | |

**4b:** Chiralpak IA, hexane/iPrOH 99/1, flow rate = 1.0 mL/min, λ = 248 nm

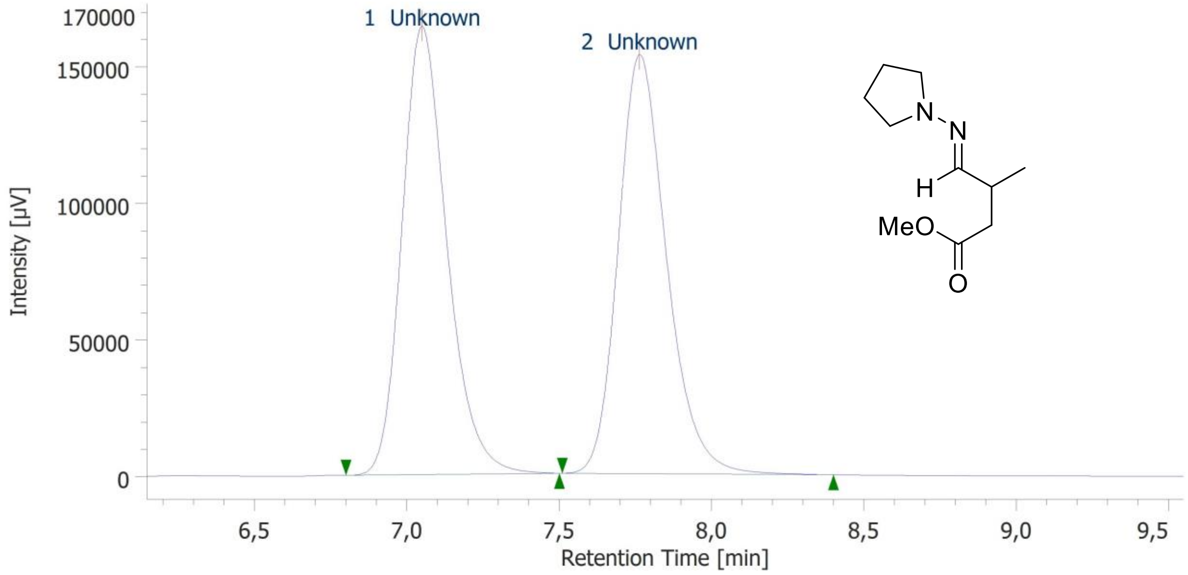


| # | Peak Name | CH | tR [min] | Area [µV·sec] | Height [µV] | Area% | Height% | Quantity | NTP | Resolution | Symmetry Factor | Warning |
|---|---|---|---|---|---|---|---|---|---|---|---|---|
| 1 | Unknown | 11 | 7,050 | 1681925 | 164039 | 49,813 | 51,682 | N/A | 11461 | 2,618 | 1,237 | |
| 2 | Unknown | 11 | 7,763 | 1694537 | 153359 | 50,187 | 48,318 | N/A | 12049 | N/A | 1,244 | |

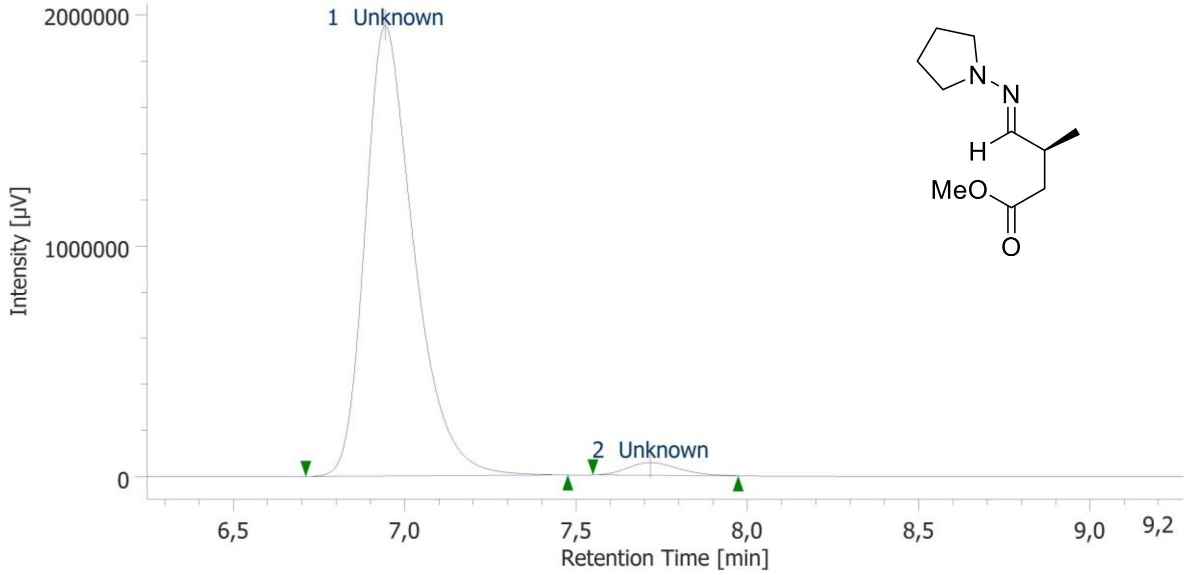


| # | Peak Name | CH | tR [min] | Area [µV·sec] | Height [µV] | Area% | Height% | Quantity | NTP | Resolution | Symmetry Factor | Warning |
|---|---|---|---|---|---|---|---|---|---|---|---|---|
| 1 | Unknown | 11 | 6,943 | 19345608 | 1951491 | 97,169 | 97,269 | N/A | 12204 | 2,960 | 1,373 | |
| 2 | Unknown | 11 | 7,717 | 563592 | 54789 | 2,831 | 2,731 | N/A | 12830 | N/A | 1,209 | |

**4c:** Chiralpak IA, hexane/iPrOH 99/1, flow rate = 1.0 mL/min, λ = 248 nm

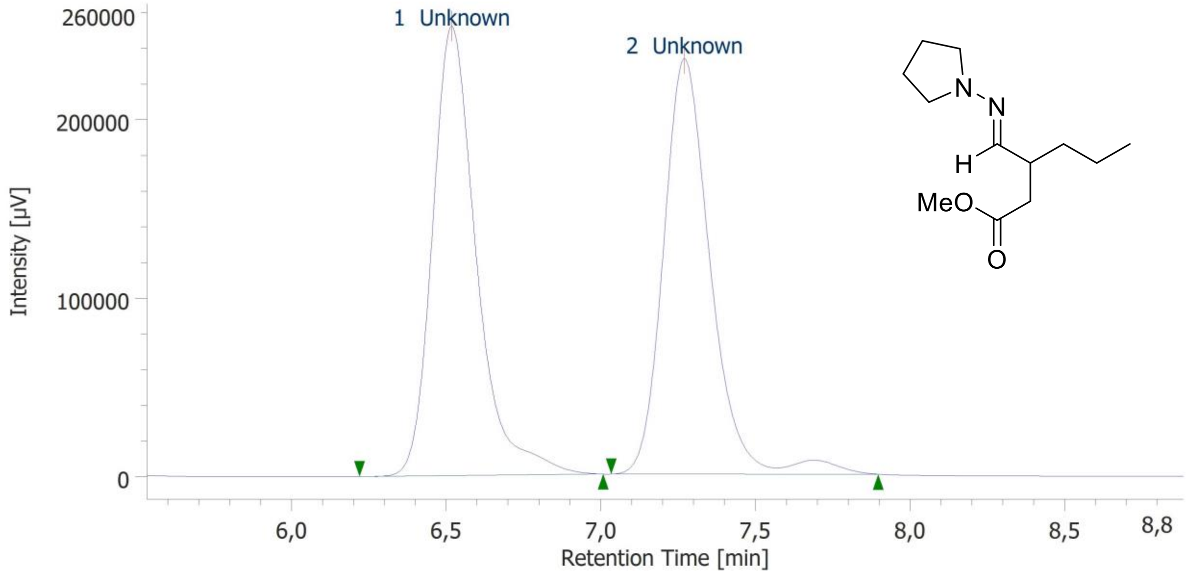


| # | Peak Name | CH | tR [min] | Area [µV·sec] | Height [µV] | Area% | Height% | Quantity | NTP | Resolution | Symmetry Factor | Warning |
|---|---|---|---|---|---|---|---|---|---|---|---|---|
| 1 | Unknown | 11 | 6,517 | 2492160 | 251763 | 50,103 | 51,957 | N/A | 11269 | 2,956 | 1,432 | |
| 2 | Unknown | 11 | 7,270 | 2481900 | 232793 | 49,897 | 48,043 | N/A | 11994 | N/A | 1,202 | |

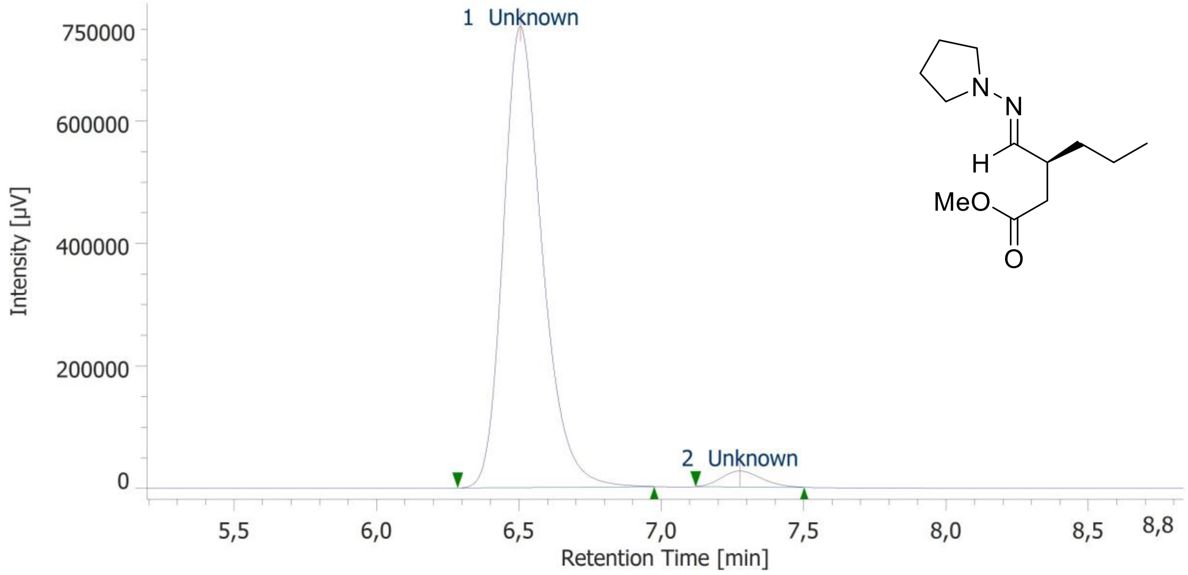


| # | Peak Name | CH | tR [min] | Area [µV·sec] | Height [µV] | Area% | Height% | Quantity | NTP | Resolution | Symmetry Factor | Warning |
|---|---|---|---|---|---|---|---|---|---|---|---|---|
| 1 | Unknown | 11 | 6,505 | 7168995 | 753066 | 96,602 | 96,670 | N/A | 11451 | 3,071 | 1,267 | |
| 2 | Unknown | 11 | 7,275 | 252204 | 25940 | 3,398 | 3,330 | N/A | 12566 | N/A | 1,181 | |

**4d:** Chiralpak IA, hexane/iPrOH 99/1, flow rate = 1.0 mL/min, λ = 248 nm

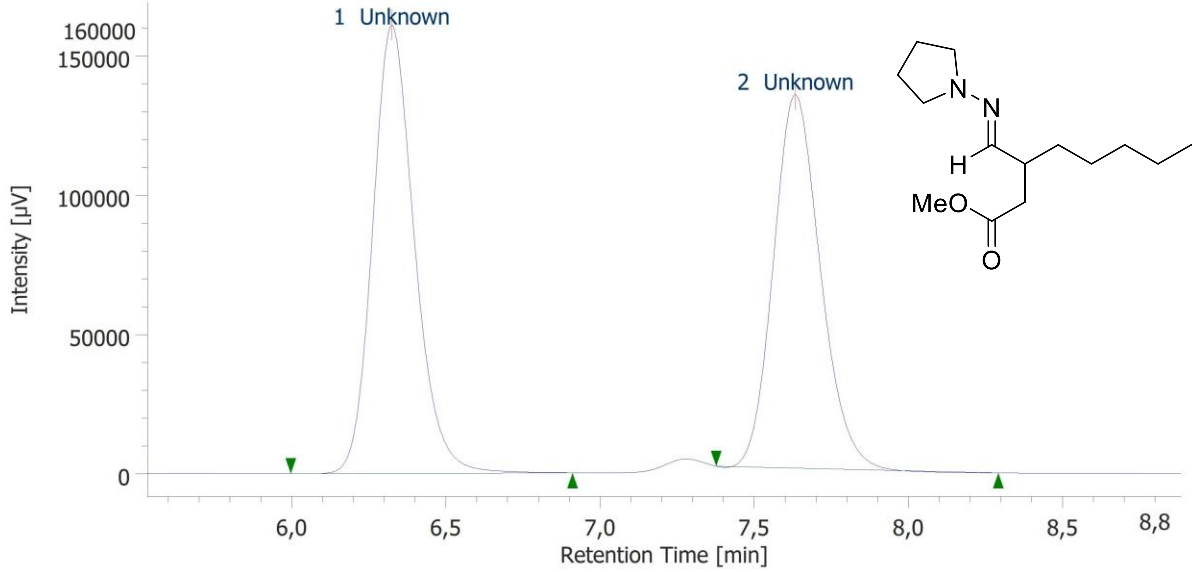


| # | Peak Name | CH | tR [min] | Area [µV·sec] | Height [µV] | Area% | Height% | Quantity | NTP | Resolution | Symmetry Factor | Warning |
|---|---|---|---|---|---|---|---|---|---|---|---|---|
| 1 | Unknown | 11 | 6,323 | 1517196 | 160823 | 51,646 | 54,545 | N/A | 10925 | 5,066 | 1,195 | |
| 2 | Unknown | 11 | 7,633 | 1420467 | 134022 | 48,354 | 45,455 | N/A | 12185 | N/A | 1,156 | |

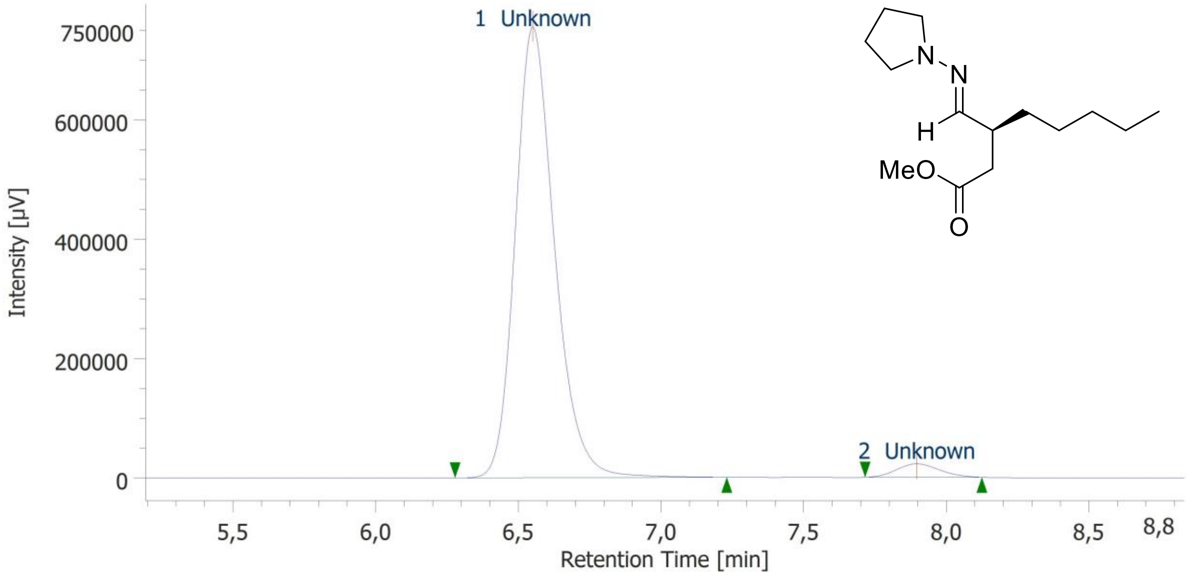


| # | Peak Name | CH | tR [min] | Area [µV·sec] | Height [µV] | Area% | Height% | Quantity | NTP | Resolution | Symmetry Factor | Warning |
|---|---|---|---|---|---|---|---|---|---|---|---|---|
| 1 | Unknown | 11 | 6,550 | 7215571 | 754871 | 96,879 | 97,115 | N/A | 11508 | 5,177 | 1,229 | |
| 2 | Unknown | 11 | 7,895 | 232479 | 22428 | 3,121 | 2,885 | N/A | 13021 | N/A | 1,134 | |

**4e:** Chiralpak IA, hexane/iPrOH 99/1, flow rate = 1.0 mL/min, λ = 248 nm

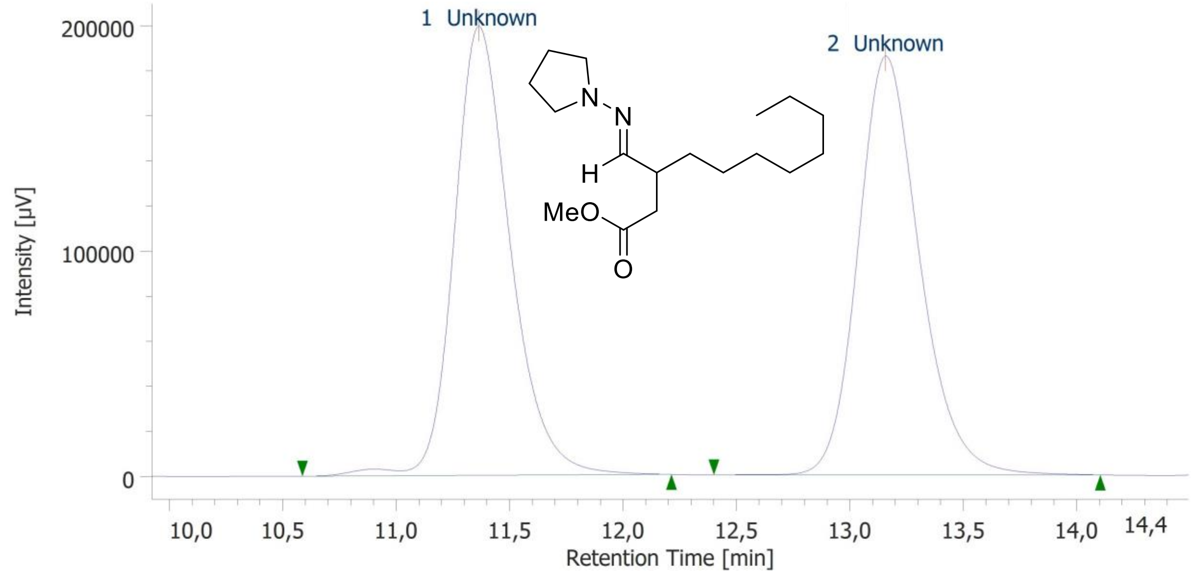


| # | Peak Name | CH | tR [min] | Area [µV·sec] | Height [µV] | Area% | Height% | Quantity | NTP | Resolution | Symmetry Factor | Warning |
|---|---|---|---|---|---|---|---|---|---|---|---|---|
| 1 | Unknown | 11 | 6,027 | 2306922 | 231046 | 50,122 | 52,241 | N/A | 9371 | 3,724 | 1,343 | |
| 2 | Unknown | 11 | 6,993 | 2295678 | 211220 | 49,878 | 47,759 | N/A | 10613 | N/A | 1,175 | |

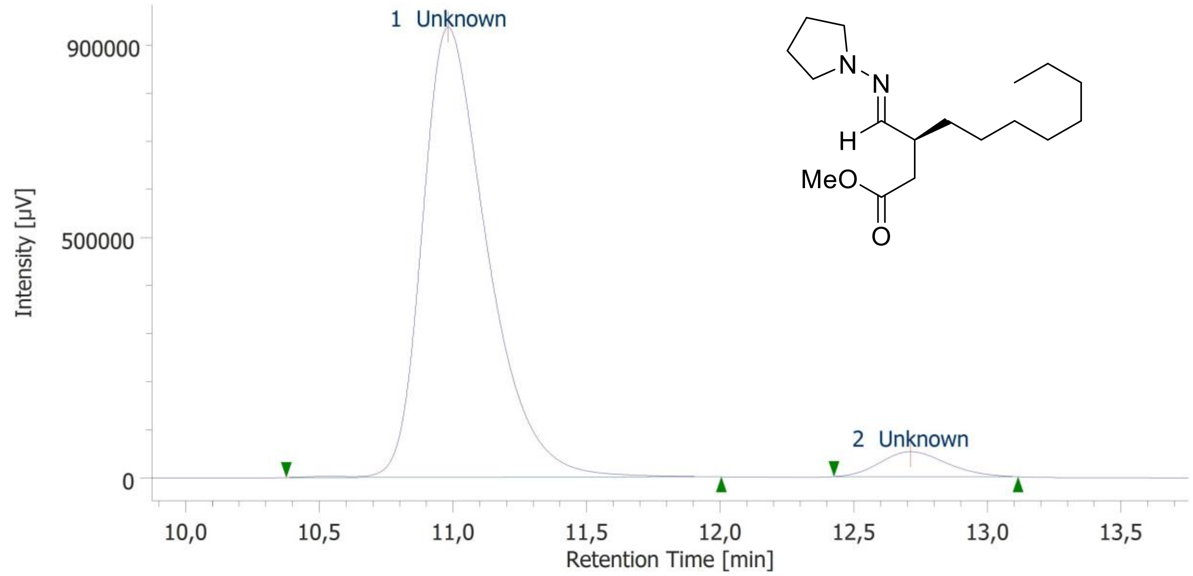


| # | Peak Name | CH | tR [min] | Area [µV·sec] | Height [µV] | Area% | Height% | Quantity | NTP | Resolution | Symmetry Factor | Warning |
|---|---|---|---|---|---|---|---|---|---|---|---|---|
| 1 | Unknown | 11 | 5,870 | 10846555 | 1165613 | 96,817 | 96,989 | N/A | 10703 | 3,815 | 1,425 | |
| 2 | Unknown | 11 | 6,797 | 356599 | 36185 | 3,183 | 3,011 | N/A | 10927 | N/A | 1,127 | |

**4f:** Chiralpak IA, hexane/iPrOH 99/1, flow rate = 1.0 mL/min, λ = 248 nm

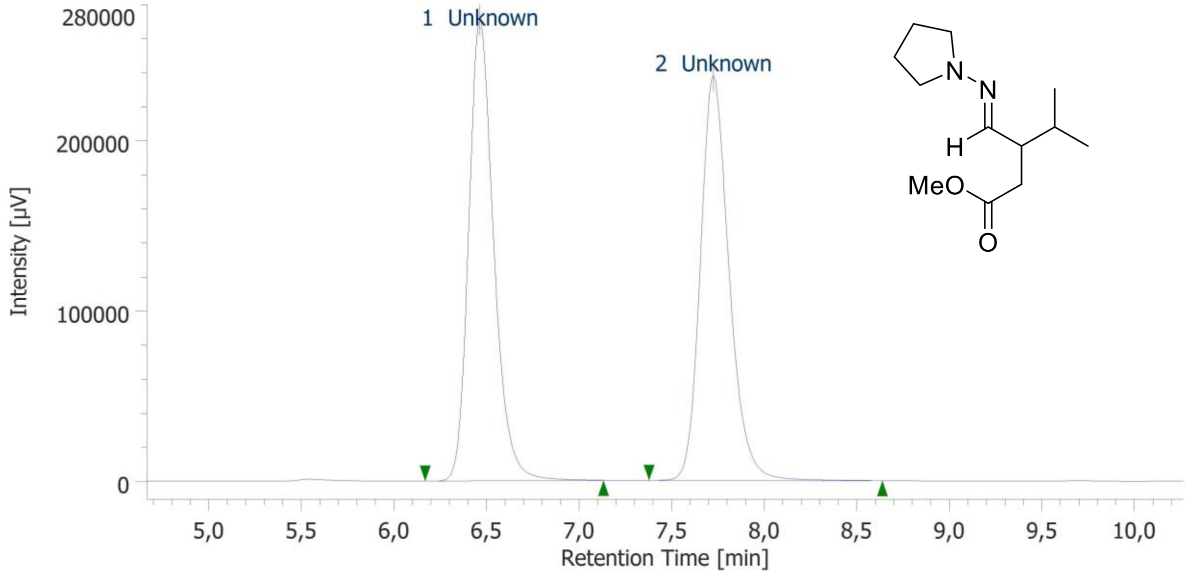


| # | Peak Name | CH | tR [min] | Area [µV·sec] | Height [µV] | Area% | Height% | Quantity | NTP | Resolution | Symmetry Factor | Warning |
|---|---|---|---|---|---|---|---|---|---|---|---|---|
| 1 | Unknown | 11 | 6,463 | 2548157 | 270269 | 49,899 | 53,262 | N/A | 11585 | 4,911 | 1,247 | |
| 2 | Unknown | 11 | 7,725 | 2558471 | 237167 | 50,101 | 46,738 | N/A | 12628 | N/A | 1,215 | |

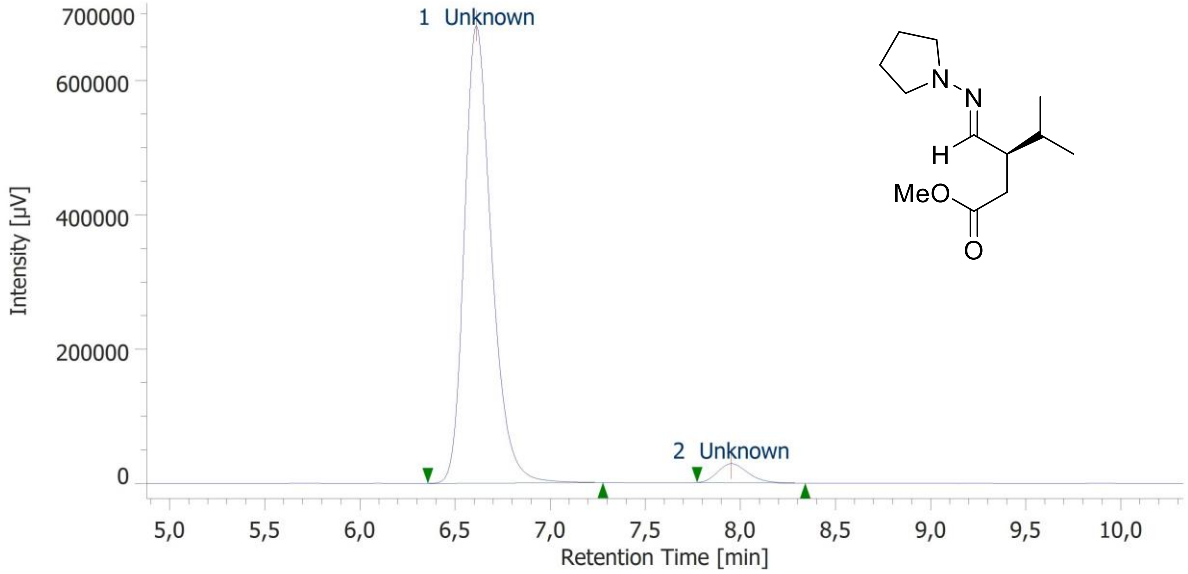


| # | Peak Name | CH | tR [min] | Area [µV·sec] | Height [µV] | Area% | Height% | Quantity | NTP | Resolution | Symmetry Factor | Warning |
|---|---|---|---|---|---|---|---|---|---|---|---|---|
| 1 | Unknown | 11 | 6,612 | 6751678 | 680578 | 95,577 | 95,990 | N/A | 10946 | 4,978 | 1,274 | |
| 2 | Unknown | 11 | 7,952 | 312446 | 28433 | 4,423 | 4,010 | N/A | 12277 | N/A | 1,230 | |

**4g:** Chiralpak IF, hexane/iPrOH 99/1, flow rate = 1.0 mL/min, λ = 248 nm

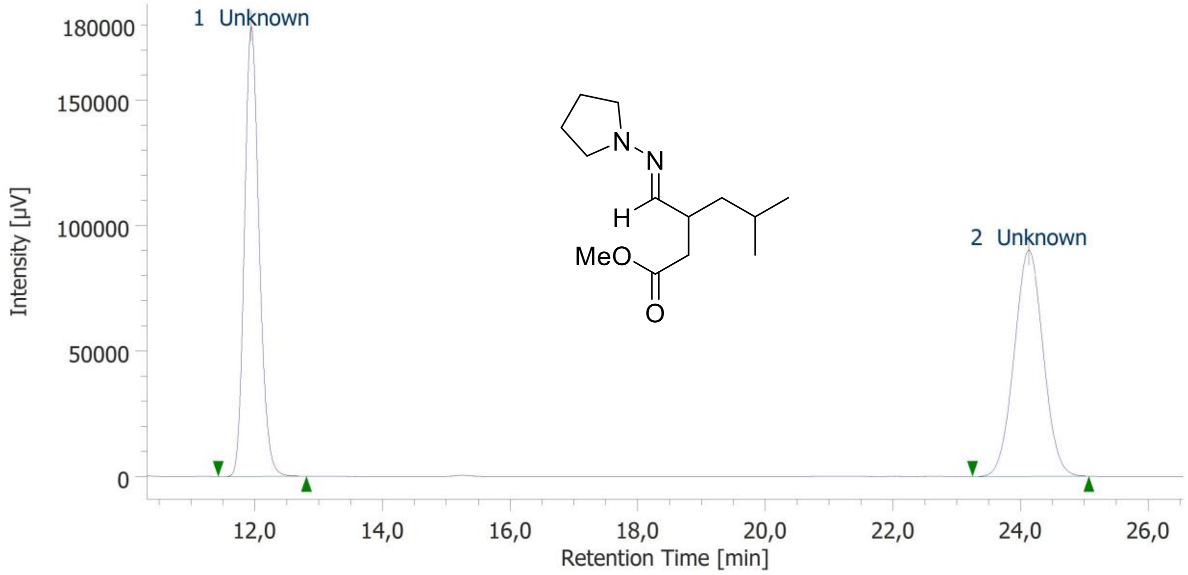


| # | Peak Name | CH | tR [min] | Area [µV·sec] | Height [µV] | Area% | Height% | Quantity | NTP | Resolution | Symmetry Factor | Warning |
|---|---|---|---|---|---|---|---|---|---|---|---|---|
| 1 | Unknown | 11 | 11,943 | 2869219 | 179297 | 50,102 | 66,552 | N/A | 13186 | 19,490 | 1,187 | |
| 2 | Unknown | 11 | 24,127 | 2857550 | 90112 | 49,898 | 33,448 | N/A | 13278 | N/A | 1,021 | |

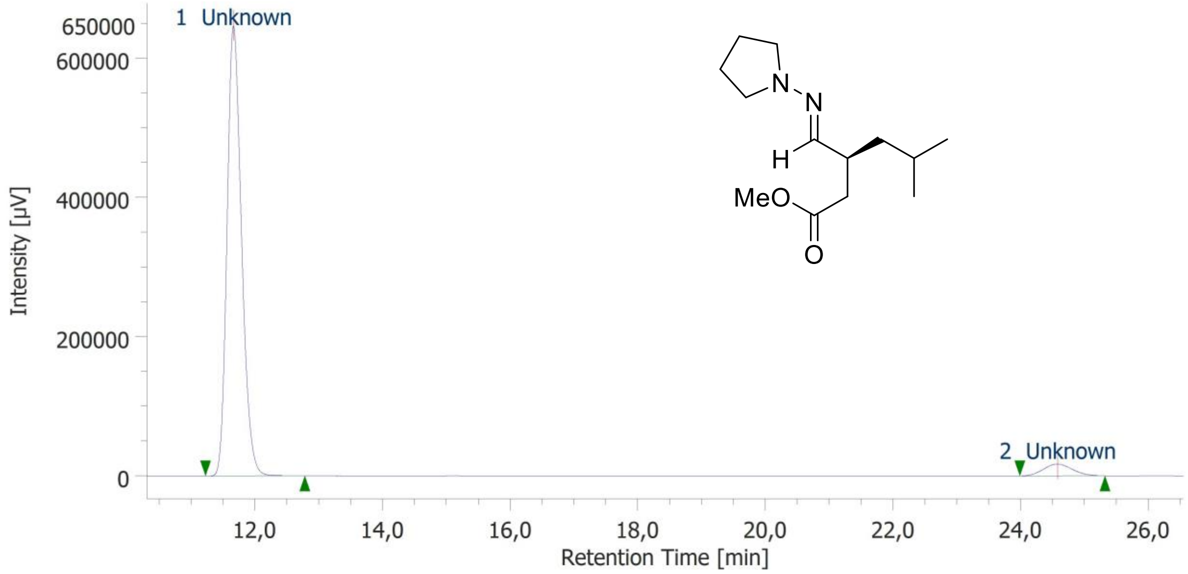


| # | Peak Name | CH | tR [min] | Area [µV·sec] | Height [µV] | Area% | Height% | Quantity | NTP | Resolution | Symmetry Factor | Warning |
|---|---|---|---|---|---|---|---|---|---|---|---|---|
| 1 | Unknown | 11 | 11,667 | 10193447 | 646304 | 94,969 | 97,430 | N/A | 12879 | 20,665 | 1,229 | |
| 2 | Unknown | 11 | 24,577 | 540014 | 17047 | 5,031 | 2,570 | N/A | 13645 | N/A | 1,092 | |

**4h:** Chiralpak IA, hexane/iPrOH 95/5, flow rate = 1.0 mL/min, λ = 248 nm

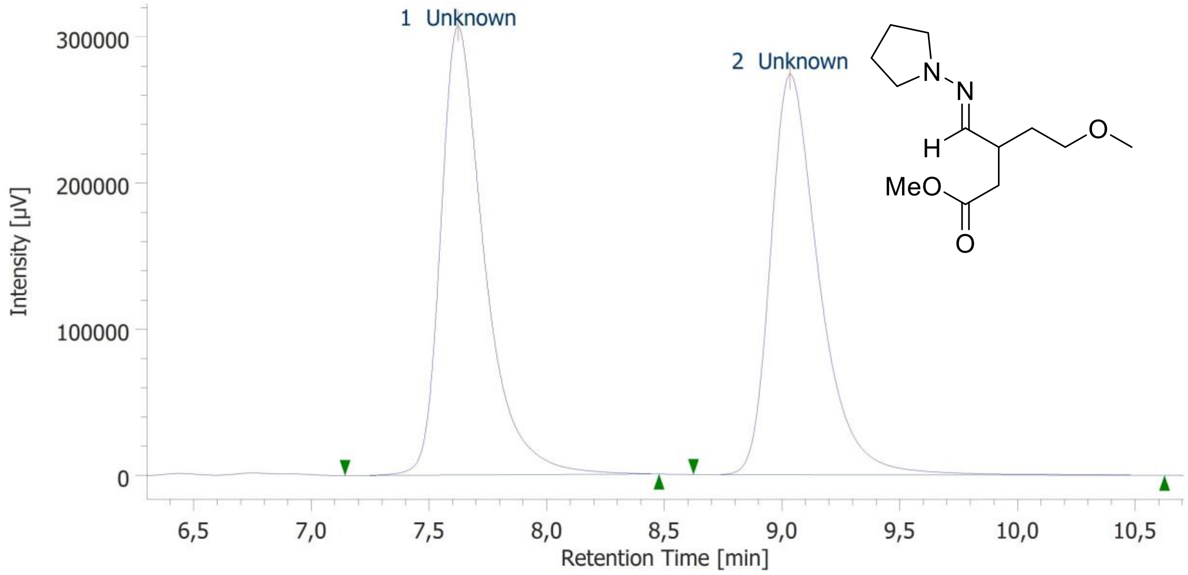


| # | Peak Name | CH | tR [min] | Area [µV·sec] | Height [µV] | Area% | Height% | Quantity | NTP | Resolution | Symmetry Factor | Warning |
|---|---|---|---|---|---|---|---|---|---|---|---|---|
| 1 | Unknown | 11 | 7,623 | 3972396 | 306945 | 50,218 | 52,835 | N/A | 9177 | 4,180 | 1,521 | |
| 2 | Unknown | 11 | 9,033 | 3937947 | 274007 | 49,782 | 47,165 | N/A | 10176 | N/A | 1,439 | |

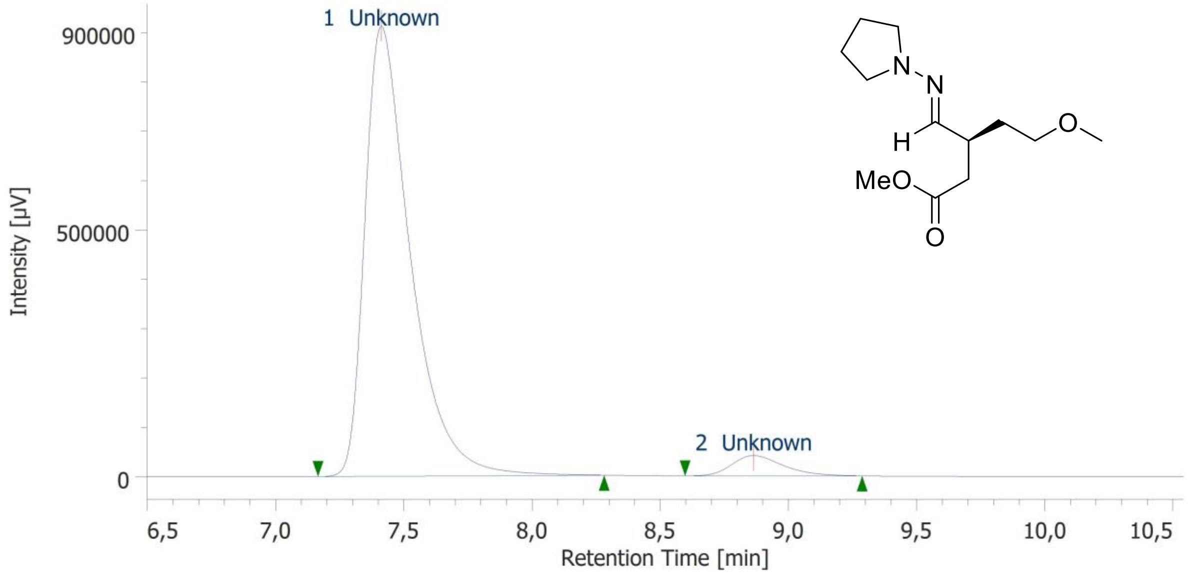


| # | Peak Name | CH | tR [min] | Area [µV·sec] | Height [µV] | Area% | Height% | Quantity | NTP | Resolution | Symmetry Factor | Warning |
|---|---|---|---|---|---|---|---|---|---|---|---|---|
| 1 | Unknown | 11 | 7,412 | 11289411 | 911678 | 95,201 | 95,693 | N/A | 9228 | 4,370 | 1,705 | |
| 2 | Unknown | 11 | 8,863 | 569083 | 41033 | 4,799 | 4,307 | N/A | 9836 | N/A | 1,333 | |

**4i:** Chiralpak IA, hexane/iPrOH 98/2, flow rate = 1.0 mL/min, λ = 248 nm

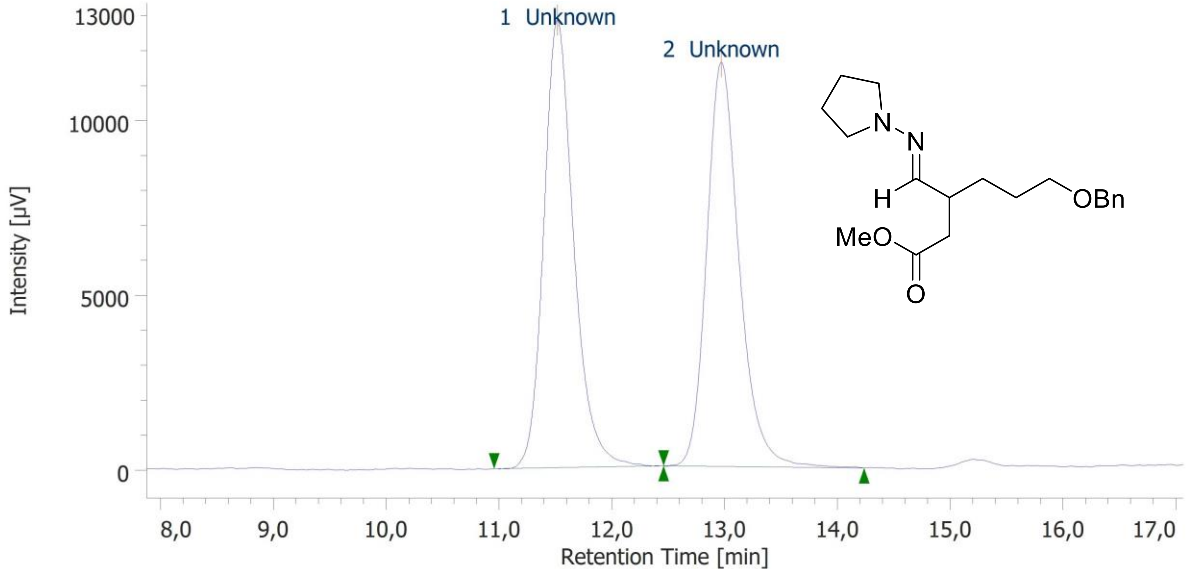


| # | Peak Name | CH | tR [min] | Area [µV·sec] | Height [µV] | Area% | Height% | Quantity | NTP | Resolution | Symmetry Factor | Warning |
|---|---|---|---|---|---|---|---|---|---|---|---|---|
| 1 | Unknown | 11 | 11,517 | 236509 | 12801 | 50,512 | 52,541 | N/A | 9769 | 2,998 | 1,221 | |
| 2 | Unknown | 11 | 12,970 | 231714 | 11563 | 49,488 | 47,459 | N/A | 10509 | N/A | 1,235 | |

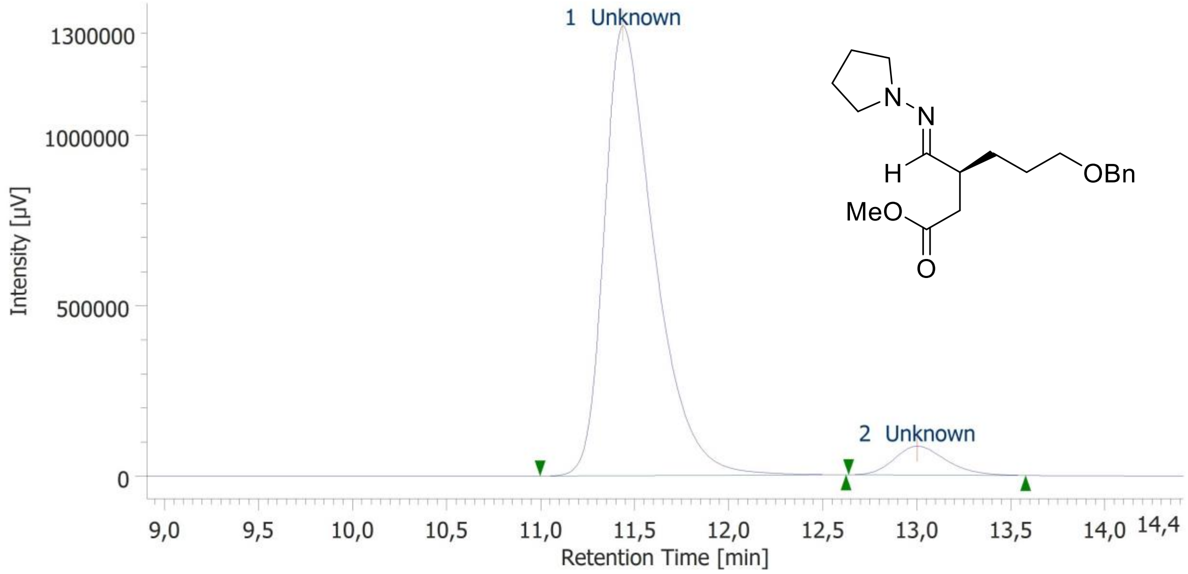


| # | Peak Name | CH | tR [min] | Area [µV·sec] | Height [µV] | Area% | Height% | Quantity | NTP | Resolution | Symmetry Factor | Warning |
|---|---|---|---|---|---|---|---|---|---|---|---|---|
| 1 | Unknown | 11 | 11,437 | 24394771 | 1319329 | 93,841 | 94,009 | N/A | 9567 | 3,254 | 1,517 | |
| 2 | Unknown | 11 | 13,000 | 1601013 | 84085 | 6,159 | 5,991 | N/A | 10996 | N/A | 1,226 | |

**4j:** Chiralpak IA, hexane/iPrOH 99/1, flow rate = 1.0 mL/min, λ = 248 nm

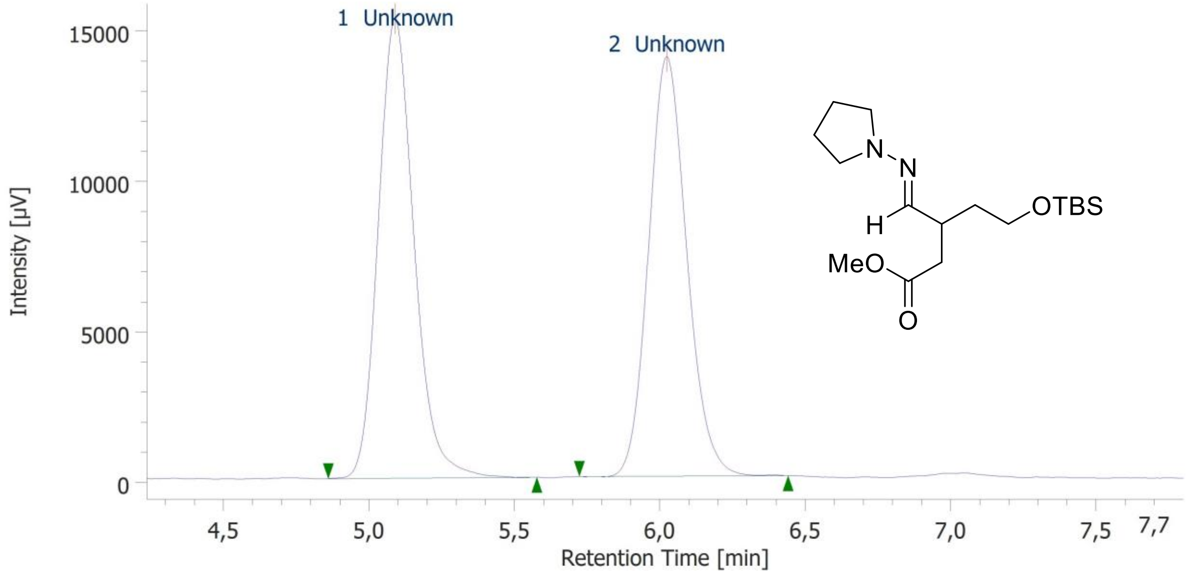


| # | Peak Name | CH | tR [min] | Area [µV·sec] | Height [µV] | Area% | Height% | Quantity | NTP | Resolution | Symmetry Factor | Warning |
|---|---|---|---|---|---|---|---|---|---|---|---|---|
| 1 | Unknown | 11 | 5,090 | 130342 | 15269 | 50,599 | 52,269 | N/A | 8602 | 4,093 | 1,174 | |
| 2 | Unknown | 11 | 6,023 | 127255 | 13943 | 49,401 | 47,731 | N/A | 10268 | N/A | 1,135 | |

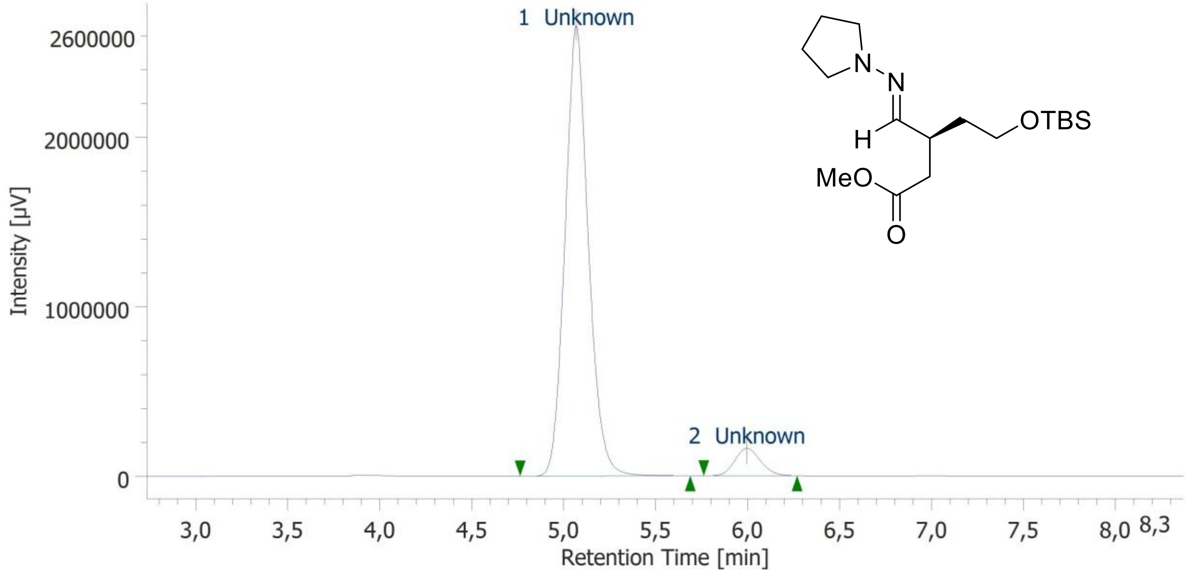


| # | Peak Name | CH | tR [min] | Area [µV·sec] | Height [µV] | Area% | Height% | Quantity | NTP | Resolution | Symmetry Factor | Warning |
|---|---|---|---|---|---|---|---|---|---|---|---|---|
| 1 | Unknown | 11 | 5,067 | 23326495 | 2656188 | 93,686 | 94,276 | N/A | 8035 | 3,855 | 1,127 | |
| 2 | Unknown | 11 | 5,995 | 1572093 | 161262 | 6,314 | 5,724 | N/A | 8722 | N/A | 1,139 | |

**4k:** Chiralpak IF, hexane/iPrOH 99/1, flow rate = 1.0 mL/min, λ = 248 nm

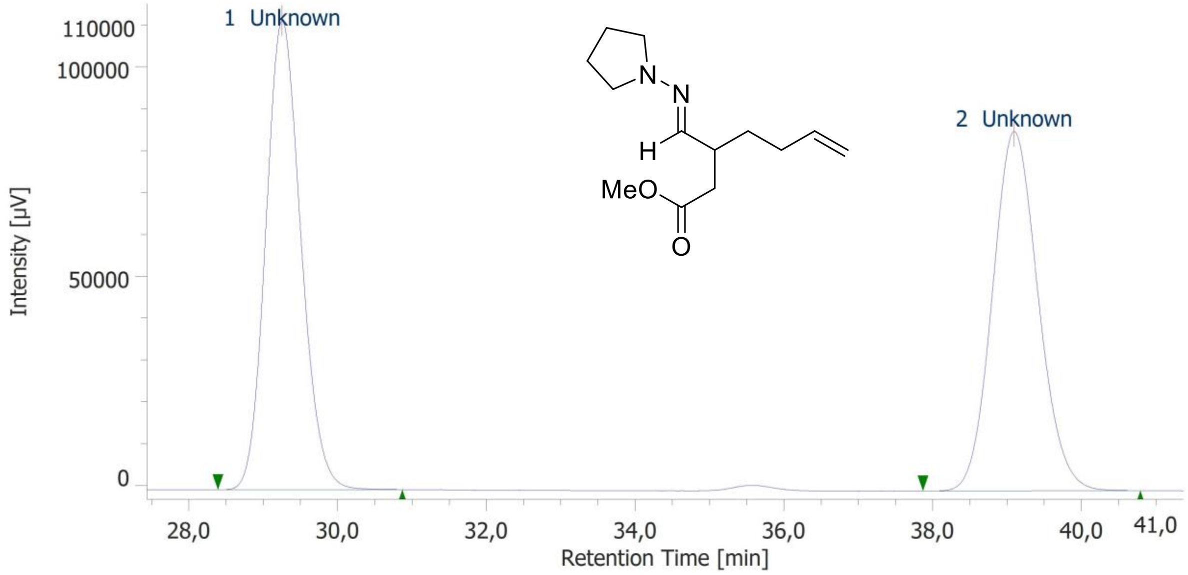


| # | Peak Name | CH | tR [min] | Area [µV·sec] | Height [µV] | Area% | Height% | Quantity | NTP | Resolution | Symmetry Factor | Warning |
|---|---|---|---|---|---|---|---|---|---|---|---|---|
| 1 | Unknown | 11 | 29,253 | 3679377 | 112066 | 50,031 | 56,618 | N/A | 18490 | 9,933 | 1,143 | |
| 2 | Unknown | 11 | 39,090 | 3674755 | 85866 | 49,969 | 43,382 | N/A | 19308 | N/A | 1,112 | |

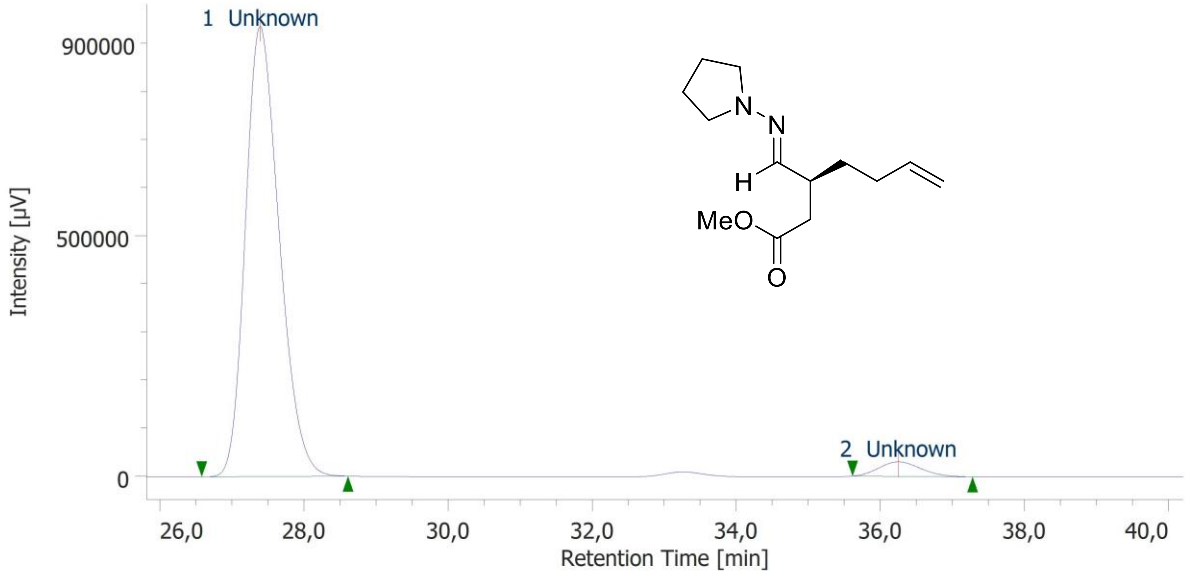


| # | Peak Name | CH | tR [min] | Area [µV·sec] | Height [µV] | Area% | Height% | Quantity | NTP | Resolution | Symmetry Factor | Warning |
|---|---|---|---|---|---|---|---|---|---|---|---|---|
| 1 | Unknown | 11 | 27,393 | 30549437 | 935198 | 96,185 | 96,881 | N/A | 16272 | 9,159 | 1,247 | |
| 2 | Unknown | 11 | 36,247 | 1211583 | 30113 | 3,815 | 3,119 | N/A | 18039 | N/A | 1,153 | |

**4l:** Chiralpak IA, hexane/iPrOH 99/1, flow rate = 1.0 mL/min, λ = 248 nm

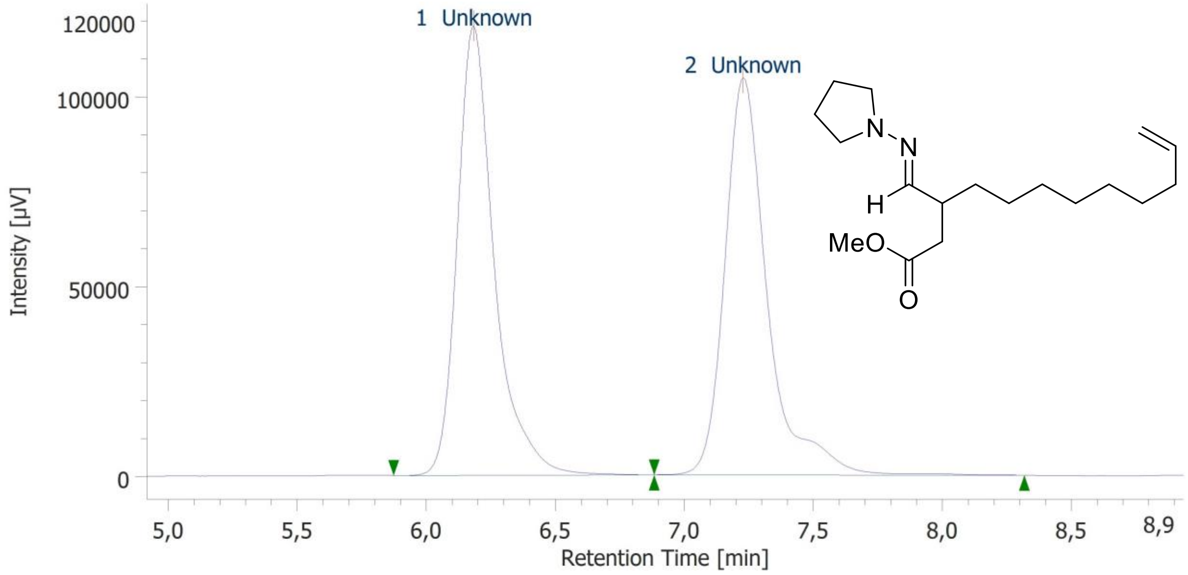


| # | Peak Name | CH | tR [min] | Area [µV·sec] | Height [µV] | Area% | Height% | Quantity | NTP | Resolution | Symmetry Factor | Warning |
|---|---|---|---|---|---|---|---|---|---|---|---|---|
| 1 | Unknown | 11 | 6,183 | 1178666 | 118200 | 49,566 | 53,070 | N/A | 10415 | 4,063 | 1,385 | |
| 2 | Unknown | 11 | 7,227 | 1199314 | 104526 | 50,434 | 46,930 | N/A | 11243 | N/A | 1,580 | |

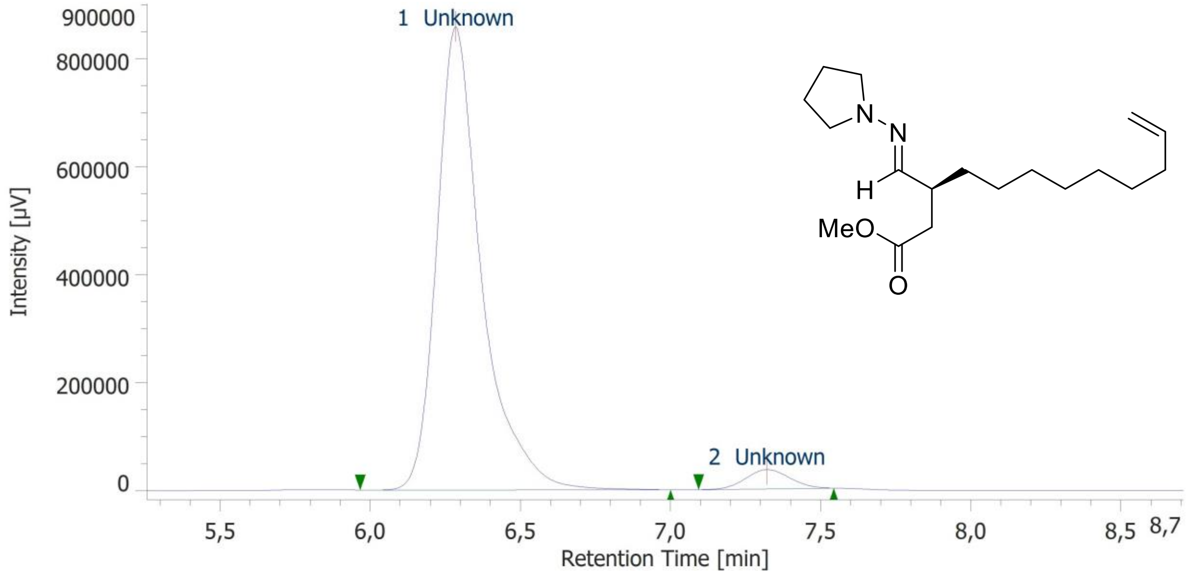


| # | Peak Name | CH | tR [min] | Area [µV·sec] | Height [µV] | Area% | Height% | Quantity | NTP | Resolution | Symmetry Factor | Warning |
|---|---|---|---|---|---|---|---|---|---|---|---|---|
| 1 | Unknown | 11 | 6,283 | 8865596 | 856956 | 96,075 | 96,009 | N/A | 10294 | 4,004 | 1,461 | |
| 2 | Unknown | 11 | 7,320 | 362190 | 35625 | 3,925 | 3,991 | N/A | 11638 | N/A | 1,068 | |

**4m:** Chiralpak IA, hexane/iPrOH 98/2, flow rate = 1.0 mL/min, λ = 248 nm

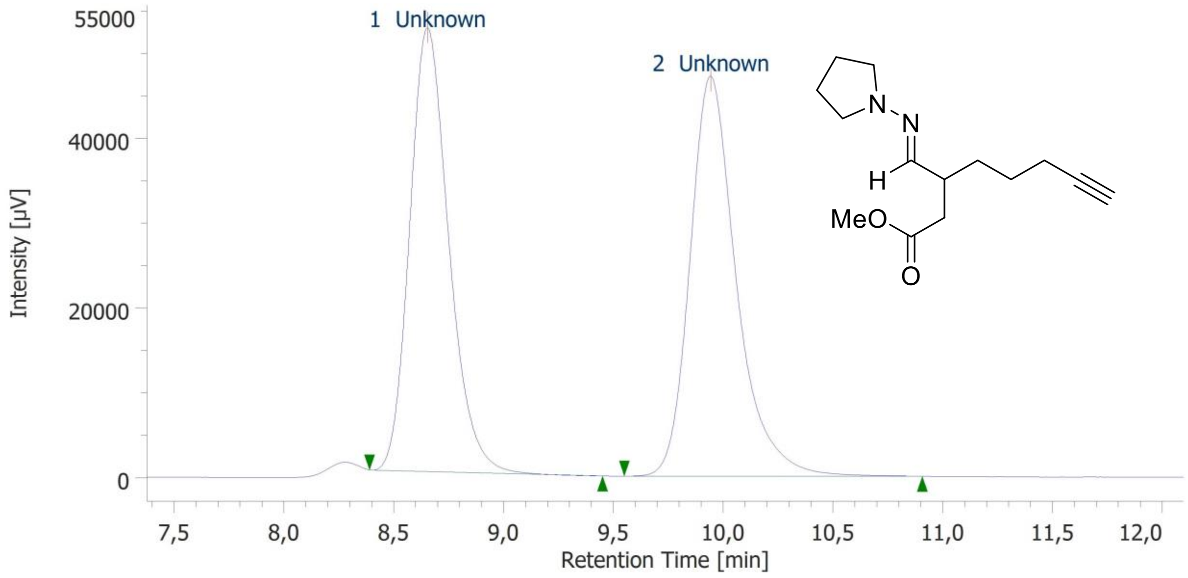


| # | Peak Name | CH | tR [min] | Area [µV·sec] | Height [µV] | Area% | Height% | Quantity | NTP | Resolution | Symmetry Factor | Warning |
|---|---|---|---|---|---|---|---|---|---|---|---|---|
| 1 | Unknown | 11 | 8,653 | 646820 | 52280 | 48,320 | 52,597 | N/A | 11726 | 3,778 | 1,218 | |
| 2 | Unknown | 11 | 9,943 | 691808 | 47118 | 51,680 | 47,403 | N/A | 11868 | N/A | 1,307 | |

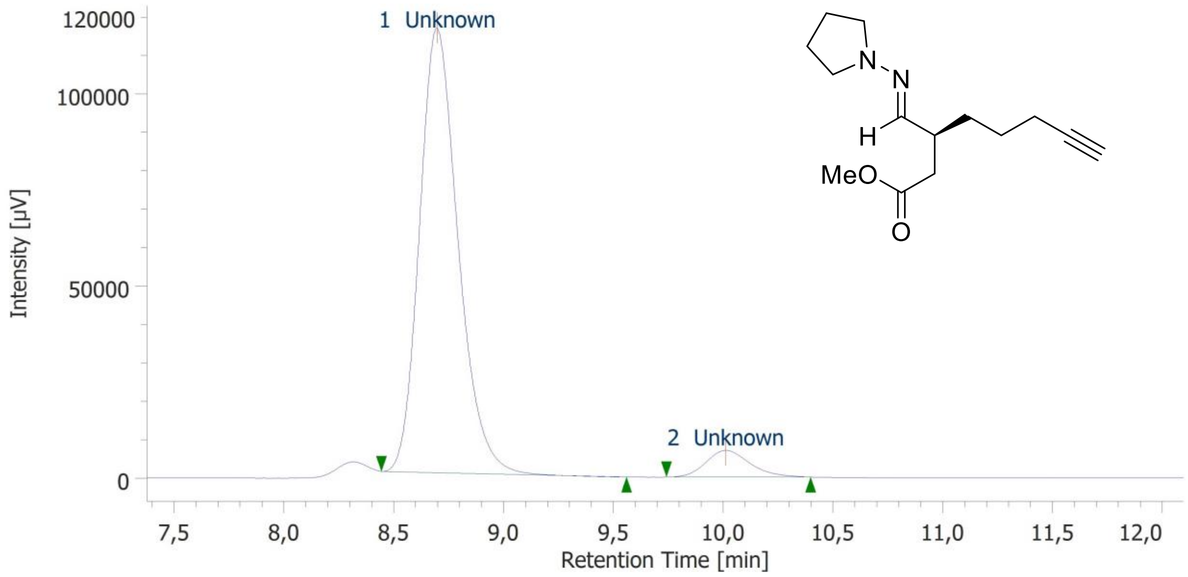


| # | Peak Name | CH | tR [min] | Area [µV·sec] | Height [µV] | Area% | Height% | Quantity | NTP | Resolution | Symmetry Factor | Warning |
|---|---|---|---|---|---|---|---|---|---|---|---|---|
| 1 | Unknown | 11 | 8,697 | 1411707 | 115587 | 93,643 | 94,343 | N/A | 12194 | 3,924 | 1,230 | |
| 2 | Unknown | 11 | 10,012 | 95828 | 6930 | 6,357 | 5,657 | N/A | 12586 | N/A | 1,223 | |

**4n:** Chiralpak IA, hexane/iPrOH 99/1, flow rate = 1.0 mL/min, λ = 248 nm

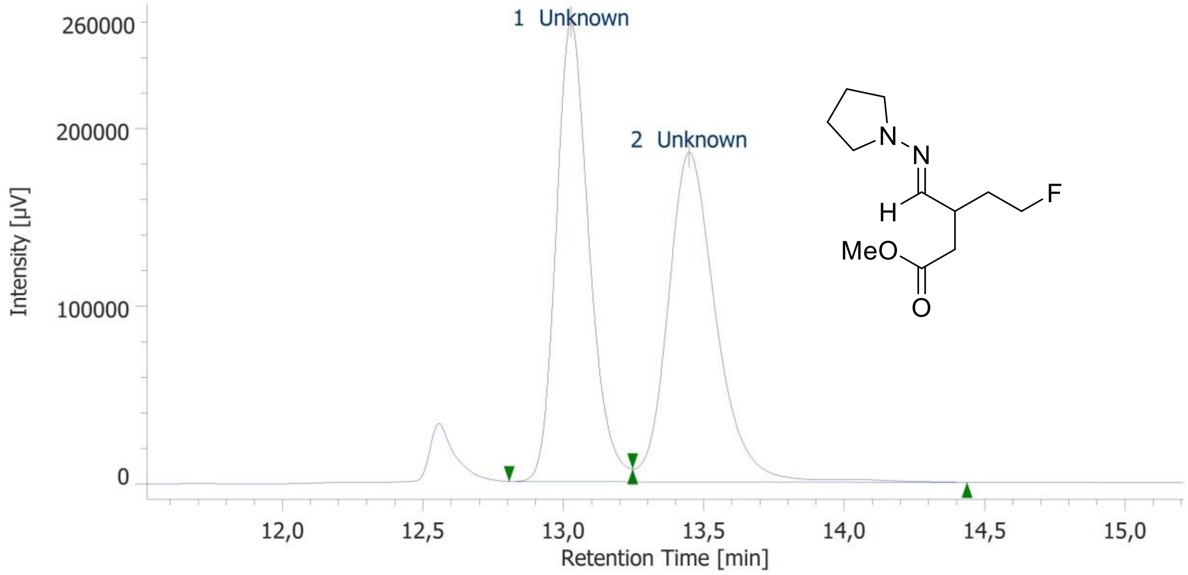


| # | Peak Name | CH | tR [min] | Area [µV·sec] | Height [µV] | Area% | Height% | Quantity | NTP | Resolution | Symmetry Factor | Warning |
|---|---|---|---|---|---|---|---|---|---|---|---|---|
| 1 | Unknown | 11 | 13,027 | 2114838 | 258681 | 48,955 | 58,261 | N/A | 61253 | 1,656 | 1,245 | |
| 2 | Unknown | 11 | 13,447 | 2205092 | 185321 | 51,045 | 41,739 | N/A | 32579 | N/A | 1,219 | |

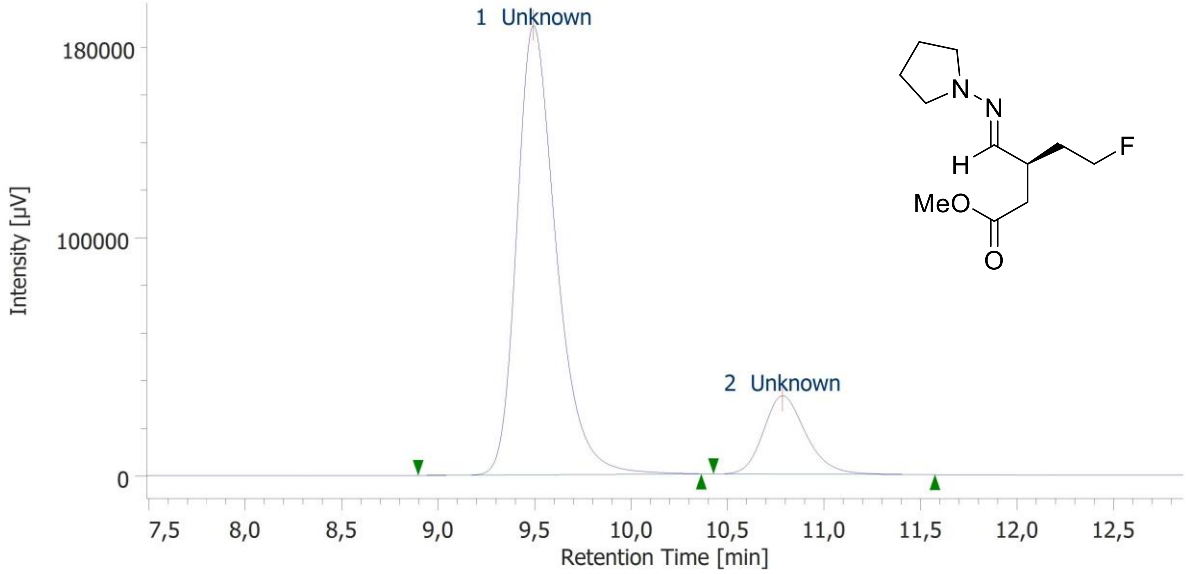


| # | Peak Name | CH | tR [min] | Area [µV·sec] | Height [µV] | Area% | Height% | Quantity | NTP | Resolution | Symmetry Factor | Warning |
|---|---|---|---|---|---|---|---|---|---|---|---|---|
| 1 | Unknown | 11 | 9,493 | 2675058 | 188579 | 84,139 | 85,140 | N/A | 11159 | 3,440 | 1,338 | |
| 2 | Unknown | 11 | 10,783 | 504279 | 32913 | 15,861 | 14,860 | N/A | 12079 | N/A | 1,225 | |

**4o:** Chiralpak IA, hexane/iPrOH 99/1, flow rate = 1.0 mL/min, λ = 248 nm

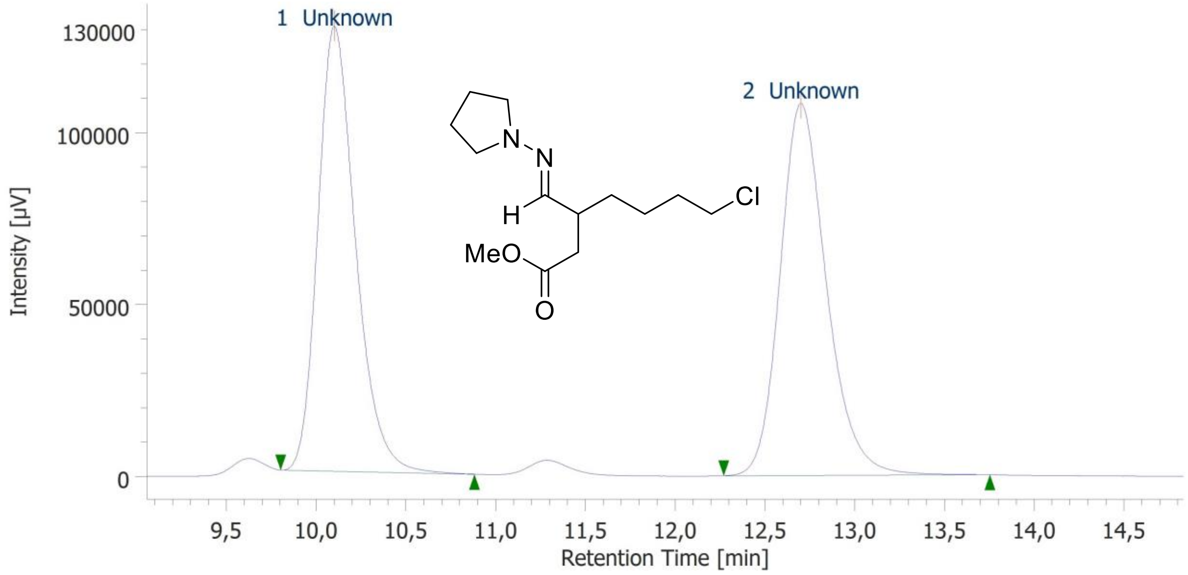


| # | Peak Name | CH | tR [min] | Area [µV·sec] | Height [µV] | Area% | Height% | Quantity | NTP | Resolution | Symmetry Factor | Warning |
|---|---|---|---|---|---|---|---|---|---|---|---|---|
| 1 | Unknown | 11 | 10,102 | 1889481 | 129381 | 49,349 | 54,472 | N/A | 11642 | 6,262 | 1,273 | |
| 2 | Unknown | 11 | 12,700 | 1939329 | 108136 | 50,651 | 45,528 | N/A | 12325 | N/A | 1,217 | |

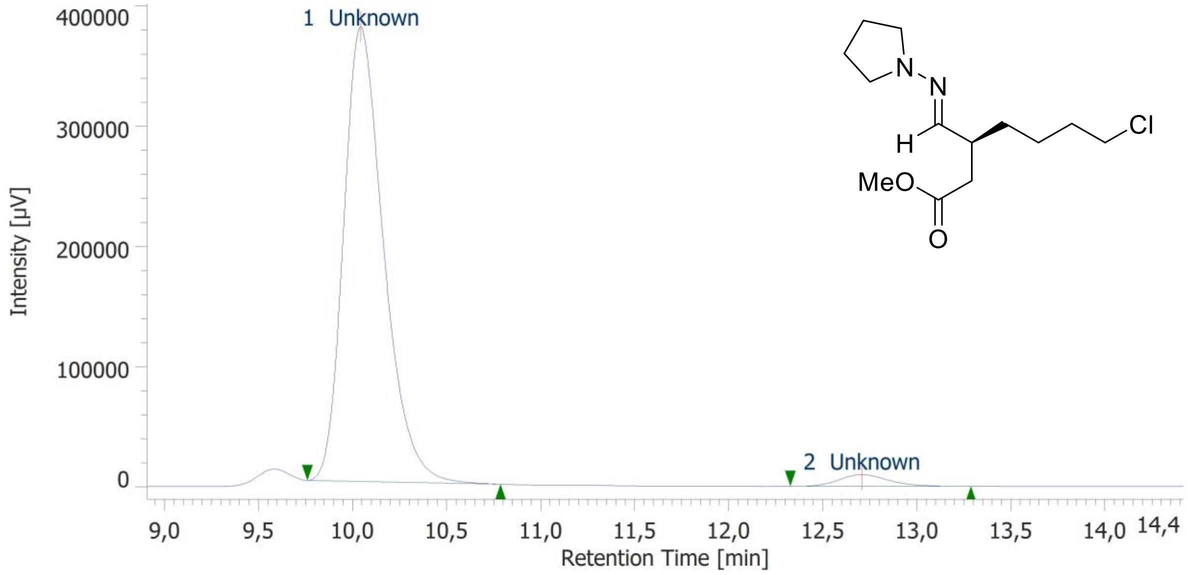


| # | Peak Name | CH | tR [min] | Area [µV·sec] | Height [µV] | Area% | Height% | Quantity | NTP | Resolution | Symmetry Factor | Warning |
|---|---|---|---|---|---|---|---|---|---|---|---|---|
| 1 | Unknown | 11 | 10,043 | 5594377 | 378396 | 96,964 | 97,489 | N/A | 11169 | 6,315 | 1,314 | |
| 2 | Unknown | 11 | 12,707 | 175165 | 9745 | 3,036 | 2,511 | N/A | 11916 | N/A | 1,172 | |

**4p:** Chiralpak IA, hexane/iPrOH 98/2, flow rate = 1.0 mL/min, $\lambda$ = 248 nm

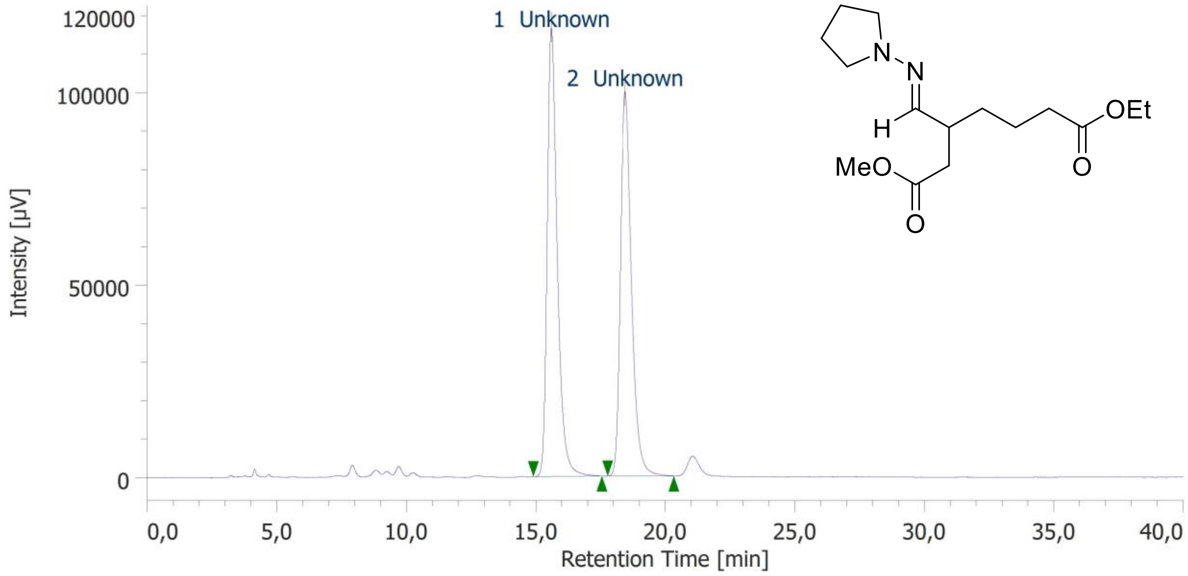


| # | Peak Name | CH | tR [min] | Area [µV·sec] | Height [µV] | Area% | Height% | Quantity | NTP | Resolution | Symmetry Factor | Warning |
|---|---|---|---|---|---|---|---|---|---|---|---|---|
| 1 | Unknown | 11 | 15,597 | 3144131 | 116549 | 51,493 | 53,842 | N/A | 8540 | 4,011 | 1,396 | |
| 2 | Unknown | 11 | 18,447 | 2961786 | 99914 | 48,507 | 46,158 | N/A | 9687 | N/A | 1,350 | |

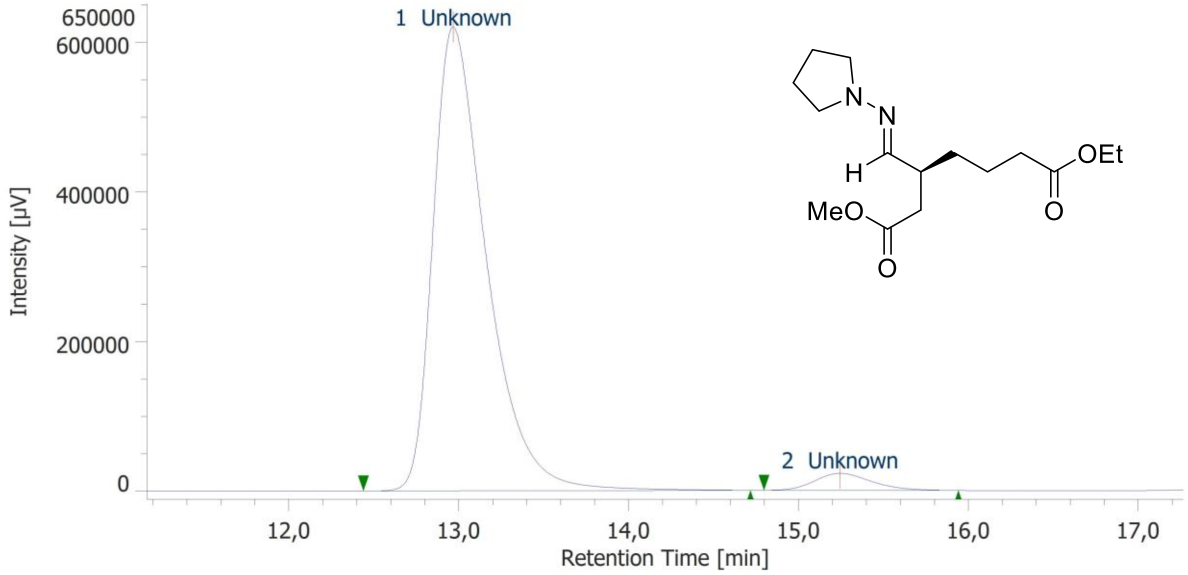


| # | Peak Name | CH | tR [min] | Area [µV·sec] | Height [µV] | Area% | Height% | Quantity | NTP | Resolution | Symmetry Factor | Warning |
|---|---|---|---|---|---|---|---|---|---|---|---|---|
| 1 | Unknown | 11 | 12,967 | 13537707 | 619863 | 96,272 | 96,482 | N/A | 8851 | 3,960 | 1,568 | |
| 2 | Unknown | 11 | 15,243 | 524237 | 22604 | 3,728 | 3,518 | N/A | 10276 | N/A | 1,235 | |

**4q:** Chiralpak IA, hexane/iPrOH 98/2, flow rate = 1.0 mL/min, λ = 248 nm

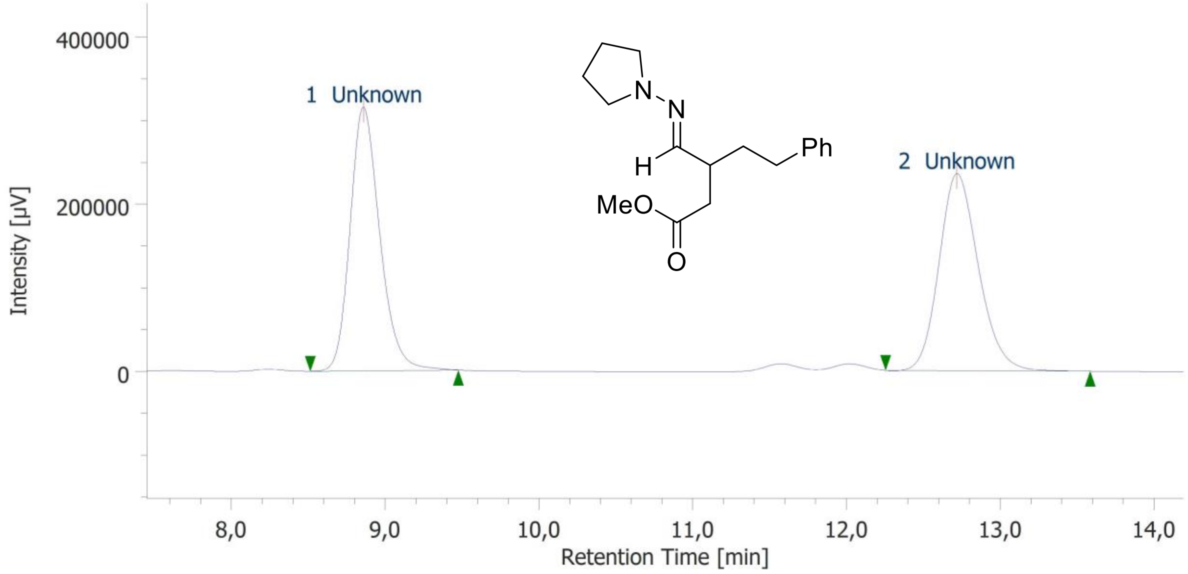


| # | Peak Name | CH | tR [min] | Area [µV·sec] | Height [µV] | Area% | Height% | Quantity | NTP | Resolution | Symmetry Factor | Warning |
|---|---|---|---|---|---|---|---|---|---|---|---|---|
| 1 | Unknown | 11 | 8,860 | 4228988 | 314993 | 50,262 | 57,224 | N/A | 10618 | 9,613 | 1,212 | |
| 2 | Unknown | 11 | 12,717 | 4184889 | 235464 | 49,738 | 42,776 | N/A | 12196 | N/A | 1,179 | |

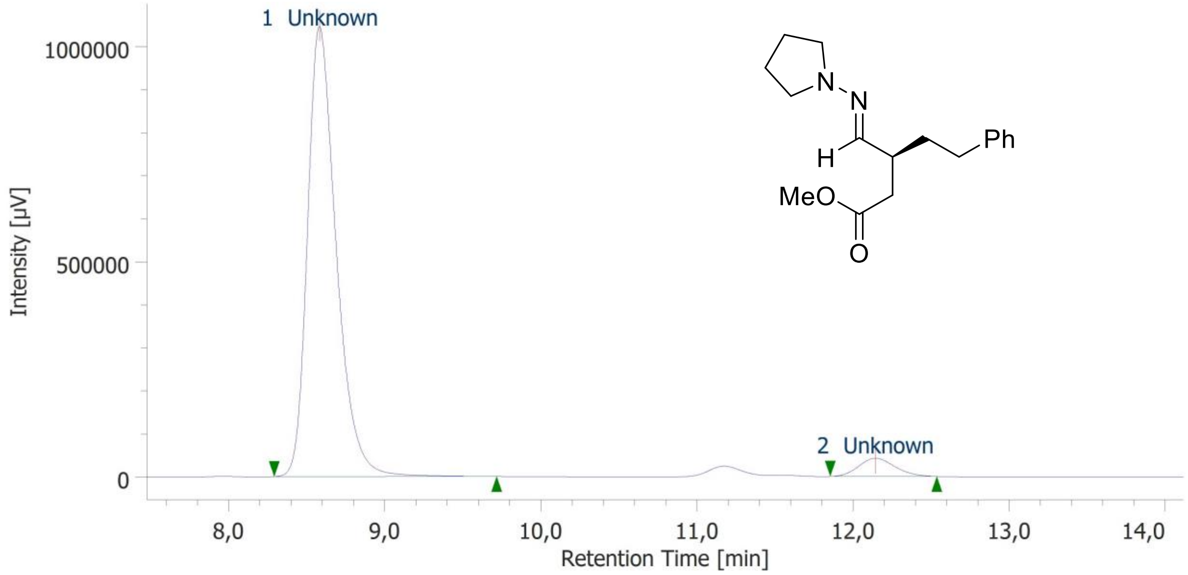


| # | Peak Name | CH | tR [min] | Area [µV·sec] | Height [µV] | Area% | Height% | Quantity | NTP | Resolution | Symmetry Factor | Warning |
|---|---|---|---|---|---|---|---|---|---|---|---|---|
| 1 | Unknown | 11 | 8,582 | 13366924 | 1046173 | 95,149 | 96,120 | N/A | 11211 | 9,514 | 1,353 | |
| 2 | Unknown | 11 | 12,142 | 681462 | 42225 | 4,851 | 3,880 | N/A | 12986 | N/A | 1,161 | |

**4r:** Chiralpak IA, hexane/iPrOH 99/1, flow rate = 1.0 mL/min, λ = 248 nm

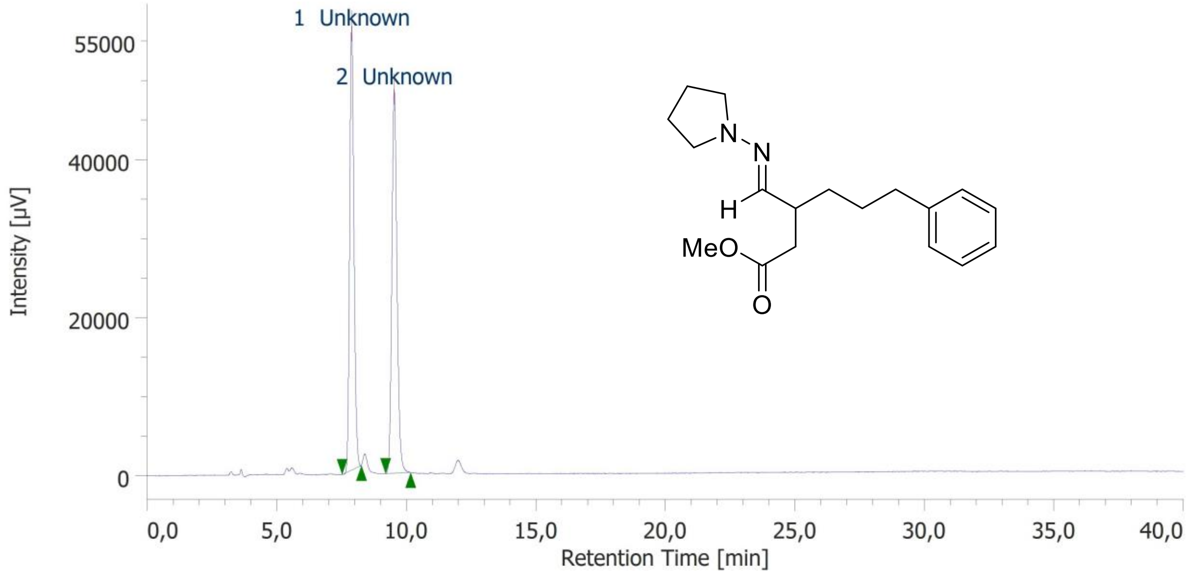


| # | Peak Name | CH | tR [min] | Area [µV·sec] | Height [µV] | Area% | Height% | Quantity | NTP | Resolution | Symmetry Factor | Warning |
|---|---|---|---|---|---|---|---|---|---|---|---|---|
| 1 | Unknown | 11 | 7,890 | 647734 | 56141 | 49,192 | 53,617 | N/A | 11024 | 5,036 | 1,172 | |
| 2 | Unknown | 11 | 9,533 | 669009 | 48565 | 50,808 | 46,383 | N/A | 11616 | N/A | 1,181 | |

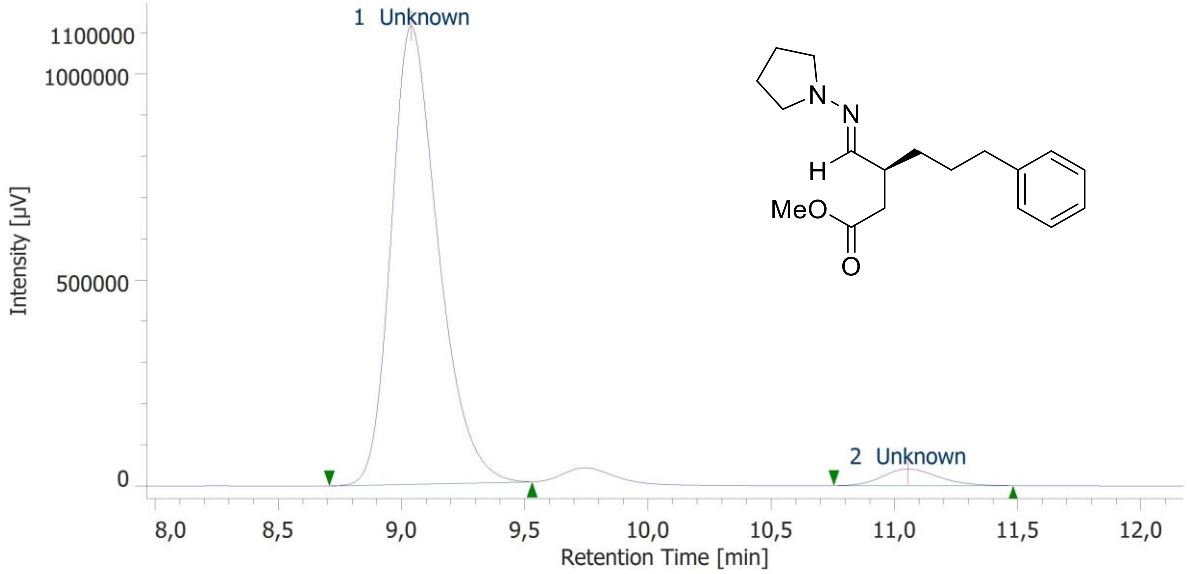


| # | Peak Name | CH | tR [min] | Area [µV·sec] | Height [µV] | Area% | Height% | Quantity | NTP | Resolution | Symmetry Factor | Warning |
|---|---|---|---|---|---|---|---|---|---|---|---|---|
| 1 | Unknown | 11 | 9,040 | 14769905 | 1111105 | 95,907 | 96,500 | N/A | 11207 | 5,380 | 1,302 | |
| 2 | Unknown | 11 | 11,053 | 630299 | 40294 | 4,093 | 3,500 | N/A | 11693 | N/A | 1,180 | |

**4s:** Chiralpak IA, hexane/iPrOH 98/2, flow rate = 1.0 mL/min, λ = 248 nm

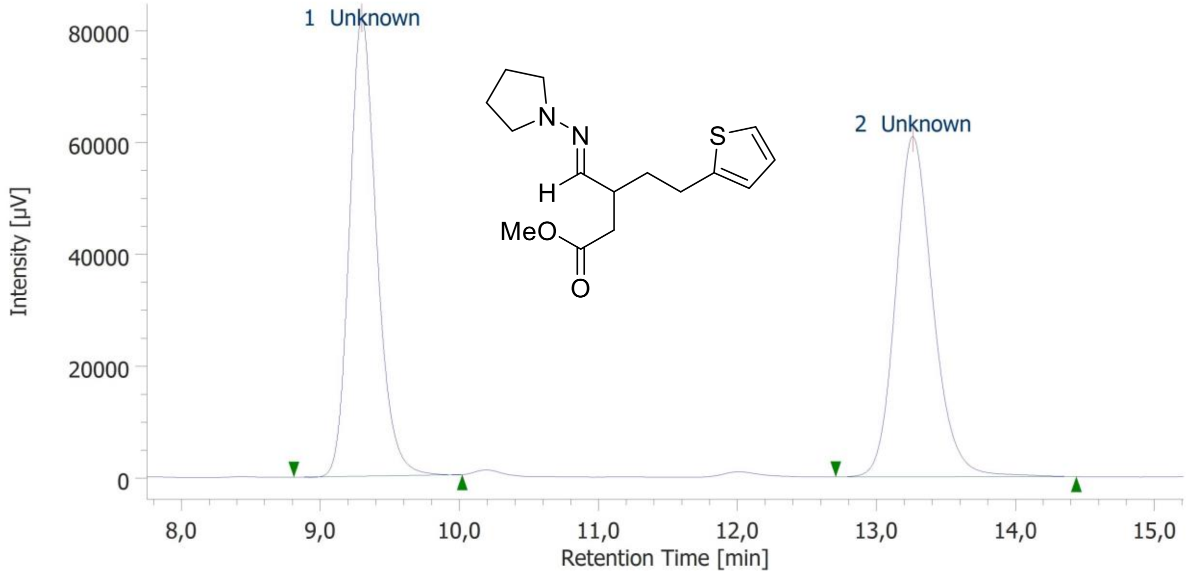


| # | Peak Name | CH | tR [min] | Area [µV·sec] | Height [µV] | Area% | Height% | Quantity | NTP | Resolution | Symmetry Factor | Warning |
|---|---|---|---|---|---|---|---|---|---|---|---|---|
| 1 | Unknown | 11 | 9,297 | 1113148 | 82052 | 49,505 | 57,438 | N/A | 11453 | 9,651 | 1,218 | |
| 2 | Unknown | 11 | 13,260 | 1135427 | 60802 | 50,495 | 42,562 | N/A | 12415 | N/A | 1,170 | |

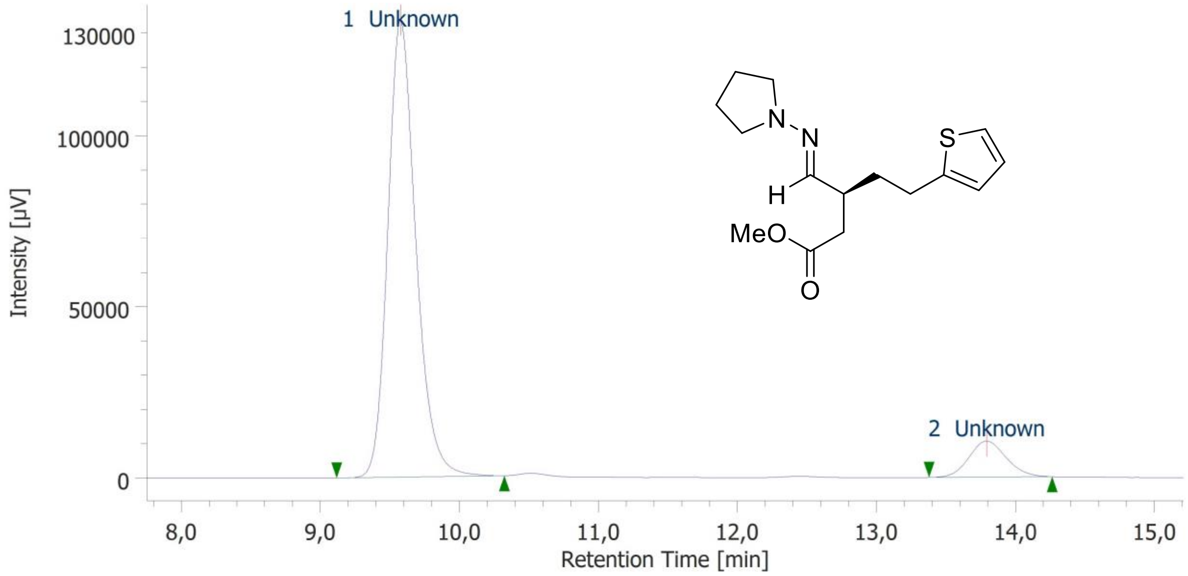


| # | Peak Name | CH | tR [min] | Area [µV·sec] | Height [µV] | Area% | Height% | Quantity | NTP | Resolution | Symmetry Factor | Warning |
|---|---|---|---|---|---|---|---|---|---|---|---|---|
| 1 | Unknown | 11 | 9,577 | 1869025 | 133394 | 90,538 | 92,702 | N/A | 11472 | 10,008 | 1,216 | |
| 2 | Unknown | 11 | 13,790 | 195323 | 10502 | 9,462 | 7,298 | N/A | 12849 | N/A | 1,116 | |

**4t:** Chiralpak IA, hexane/iPrOH 98/2, flow rate = 1.0 mL/min, λ = 248 nm

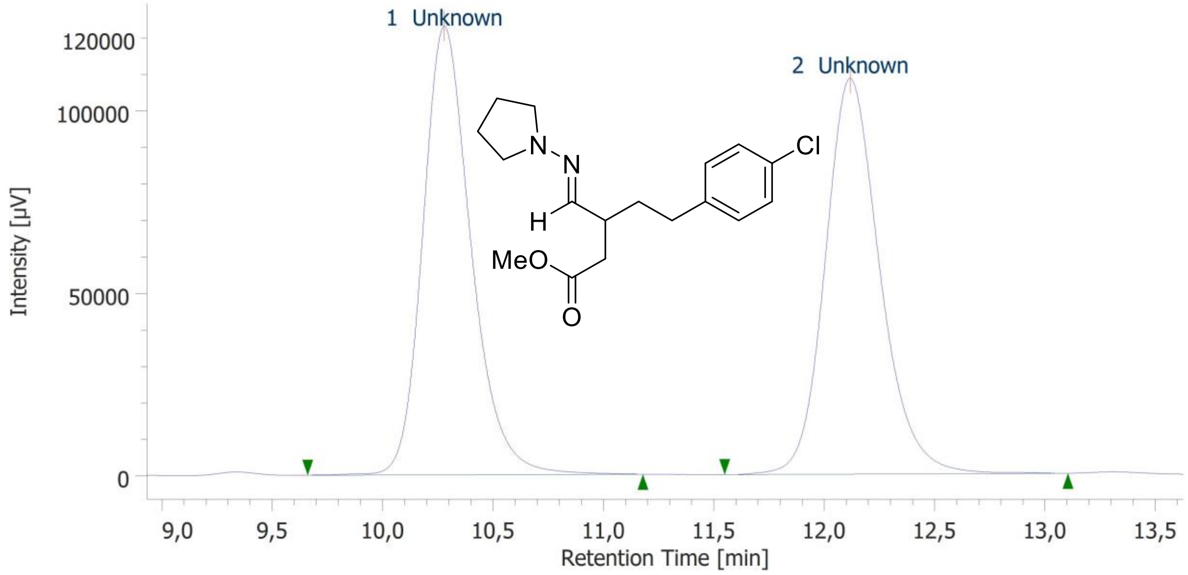


| # | Peak Name | CH | tR [min] | Area [µV·sec] | Height [µV] | Area% | Height% | Quantity | NTP | Resolution | Symmetry Factor | Warning |
|---|---|---|---|---|---|---|---|---|---|---|---|---|
| 1 | Unknown | 11 | 10,278 | 1867451 | 122902 | 49,954 | 53,114 | N/A | 11358 | 4,471 | 1,245 | |
| 2 | Unknown | 11 | 12,117 | 1870875 | 108493 | 50,046 | 46,886 | N/A | 12203 | N/A | 1,181 | |

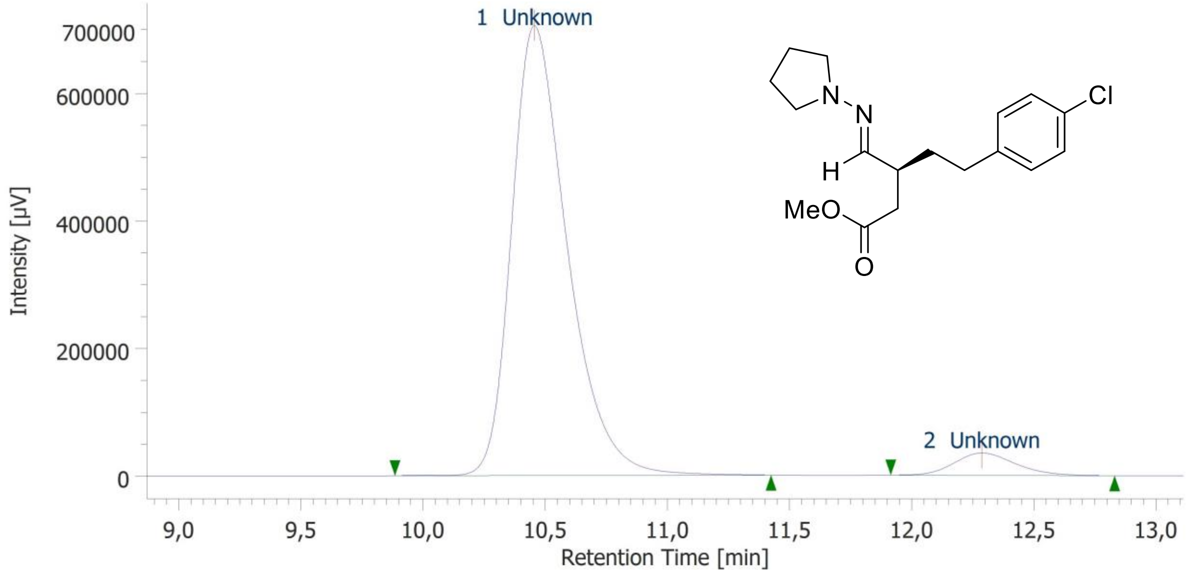


| # | Peak Name | CH | tR [min] | Area [µV·sec] | Height [µV] | Area% | Height% | Quantity | NTP | Resolution | Symmetry Factor | Warning |
|---|---|---|---|---|---|---|---|---|---|---|---|---|
| 1 | Unknown | 11 | 10,455 | 11158079 | 704976 | 94,862 | 95,289 | N/A | 10767 | 4,301 | 1,393 | |
| 2 | Unknown | 11 | 12,287 | 604309 | 34850 | 5,138 | 4,711 | N/A | 11879 | N/A | 1,194 | |

**4u:** Chiralpak IA, hexane/iPrOH 98/2, flow rate = 1.0 mL/min, λ = 248 nm

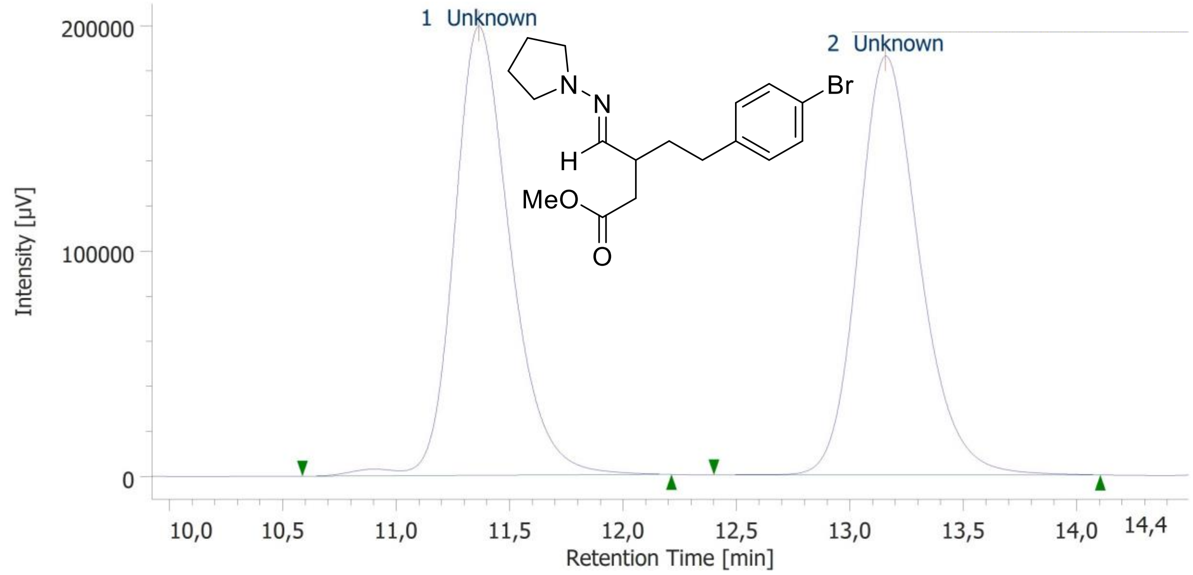


| # | Peak Name | CH | tR [min] | Area [µV·sec] | Height [µV] | Area% | Height% | Quantity | NTP | Resolution | Symmetry Factor | Warning |
|---|---|---|---|---|---|---|---|---|---|---|---|---|
| 1 | Unknown | 11 | 11,363 | 3439974 | 199221 | 49,495 | 51,752 | N/A | 10925 | 3,916 | 1,264 | |
| 2 | Unknown | 11 | 13,157 | 3510158 | 185735 | 50,505 | 48,248 | N/A | 11849 | N/A | 1,209 | |

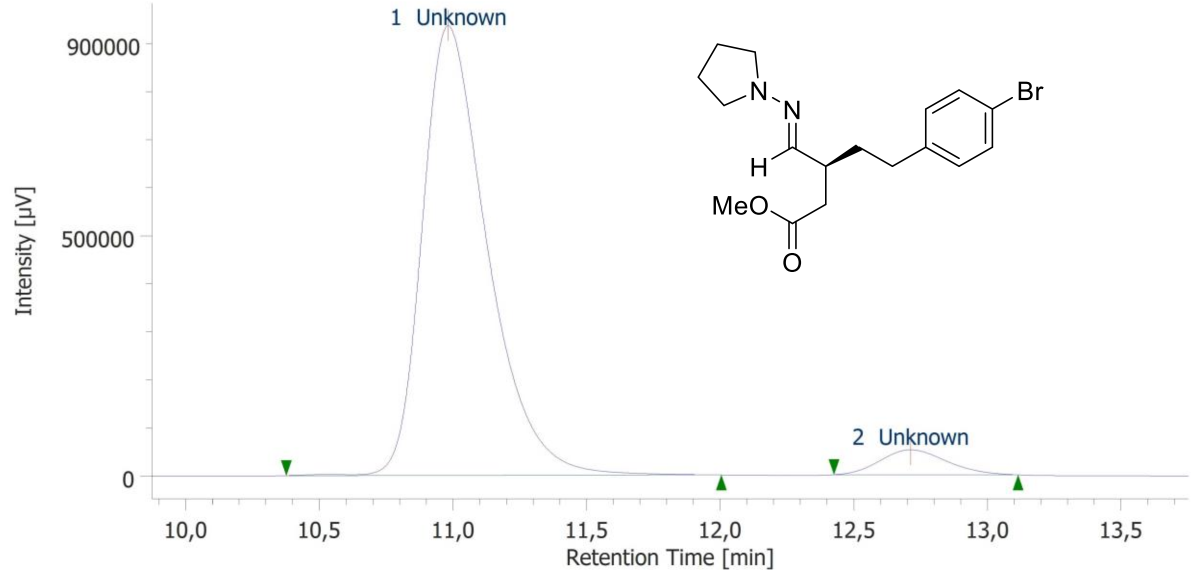


| # | Peak Name | CH | tR [min] | Area [µV·sec] | Height [µV] | Area% | Height% | Quantity | NTP | Resolution | Symmetry Factor | Warning |
|---|---|---|---|---|---|---|---|---|---|---|---|---|
| 1 | Unknown | 11 | 10,982 | 15835490 | 935138 | 94,650 | 94,741 | N/A | 10412 | 3,904 | 1,444 | |
| 2 | Unknown | 11 | 12,712 | 895051 | 51914 | 5,350 | 5,259 | N/A | 12320 | N/A | 1,171 | |

**4v:** Chiralpak IA, hexane/iPrOH 98/2, flow rate = 1.0 mL/min, λ = 248 nm

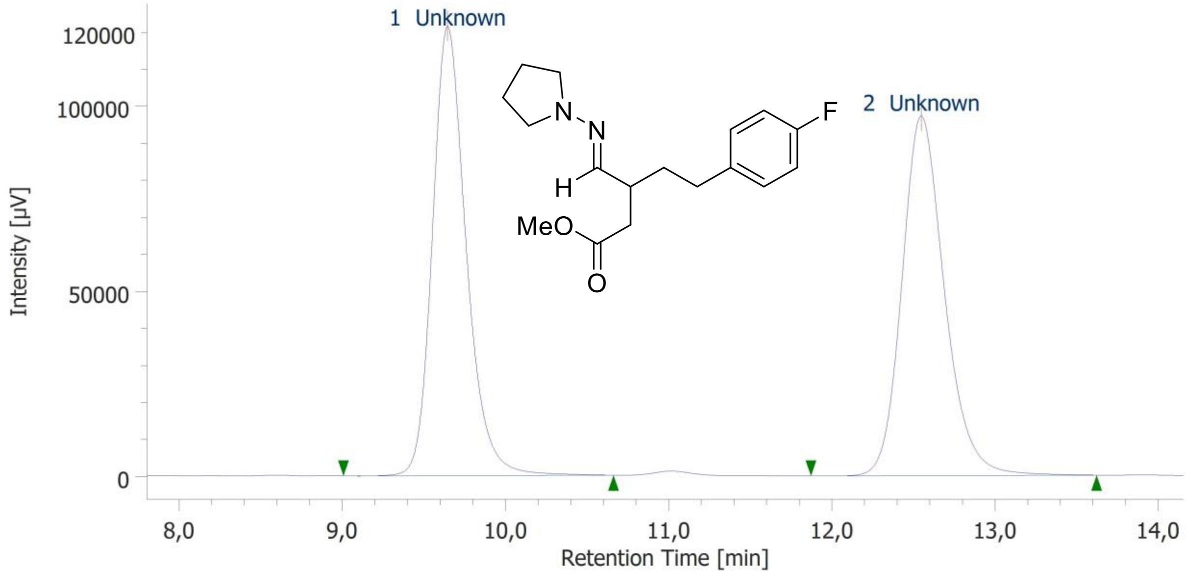


| # | Peak Name | CH | tR [min] | Area [µV·sec] | Height [µV] | Area% | Height% | Quantity | NTP | Resolution | Symmetry Factor | Warning |
|---|---|---|---|---|---|---|---|---|---|---|---|---|
| 1 | Unknown | 11 | 9,643 | 1743172 | 121345 | 49,804 | 55,548 | N/A | 11252 | 7,059 | 1,236 | |
| 2 | Unknown | 11 | 12,547 | 1756875 | 97108 | 50,196 | 44,452 | N/A | 11846 | N/A | 1,193 | |

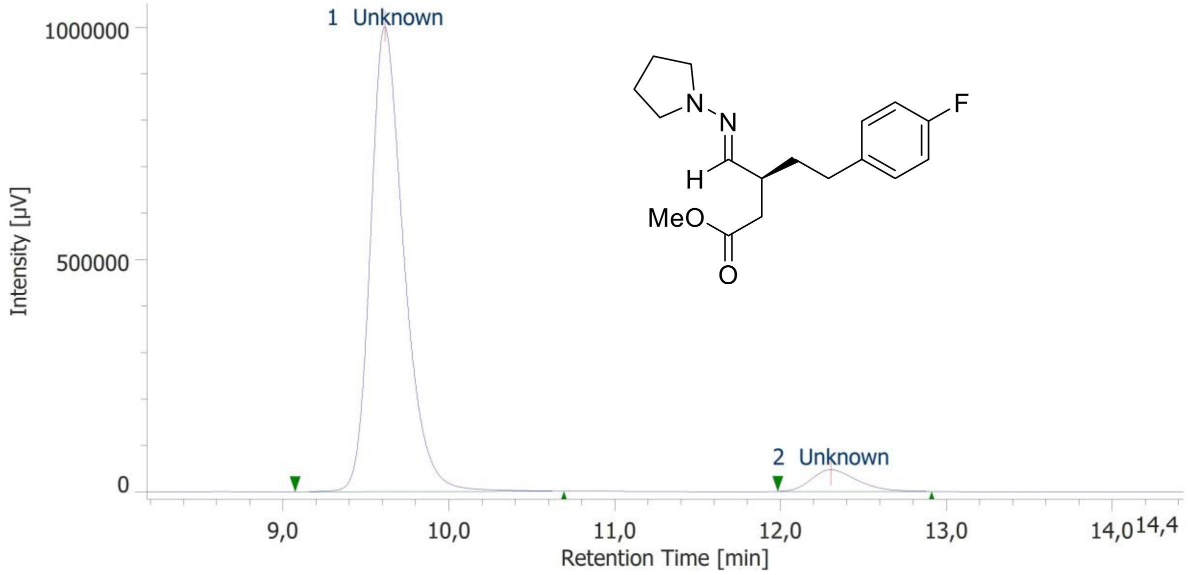


| # | Peak Name | CH | tR [min] | Area [µV·sec] | Height [µV] | Area% | Height% | Quantity | NTP | Resolution | Symmetry Factor | Warning |
|---|---|---|---|---|---|---|---|---|---|---|---|---|
| 1 | Unknown | 11 | 9,613 | 14014356 | 1000617 | 94,018 | 95,551 | N/A | 11745 | 6,332 | 1,300 | |
| 2 | Unknown | 11 | 12,303 | 891649 | 46586 | 5,982 | 4,449 | N/A | 9803 | N/A | 1,305 | |

**4w:** Chiralpak IA, hexane/iPrOH 98/2, flow rate = 1.0 mL/min, λ = 248 nm

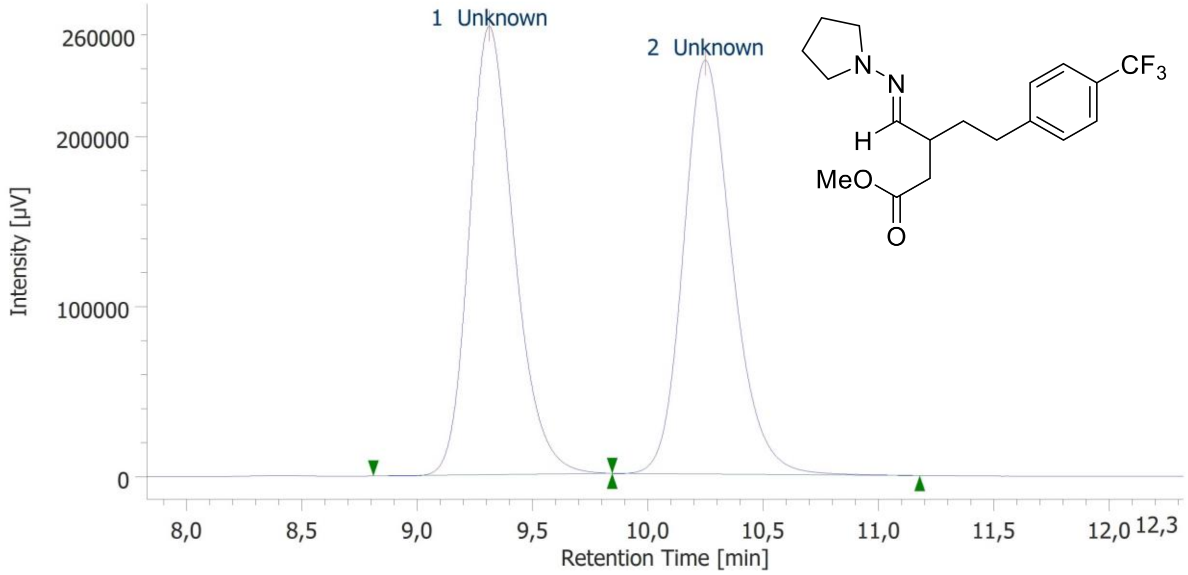


| # | Peak Name | CH | tR [min] | Area [µV·sec] | Height [µV] | Area% | Height% | Quantity | NTP | Resolution | Symmetry Factor | Warning |
|---|---|---|---|---|---|---|---|---|---|---|---|---|
| 1 | Unknown | 11 | 9,312 | 3551337 | 263772 | 49,795 | 52,004 | N/A | 11525 | 2,595 | 1,237 | |
| 2 | Unknown | 11 | 10,248 | 3580552 | 243445 | 50,205 | 47,996 | N/A | 11834 | N/A | 1,209 | |

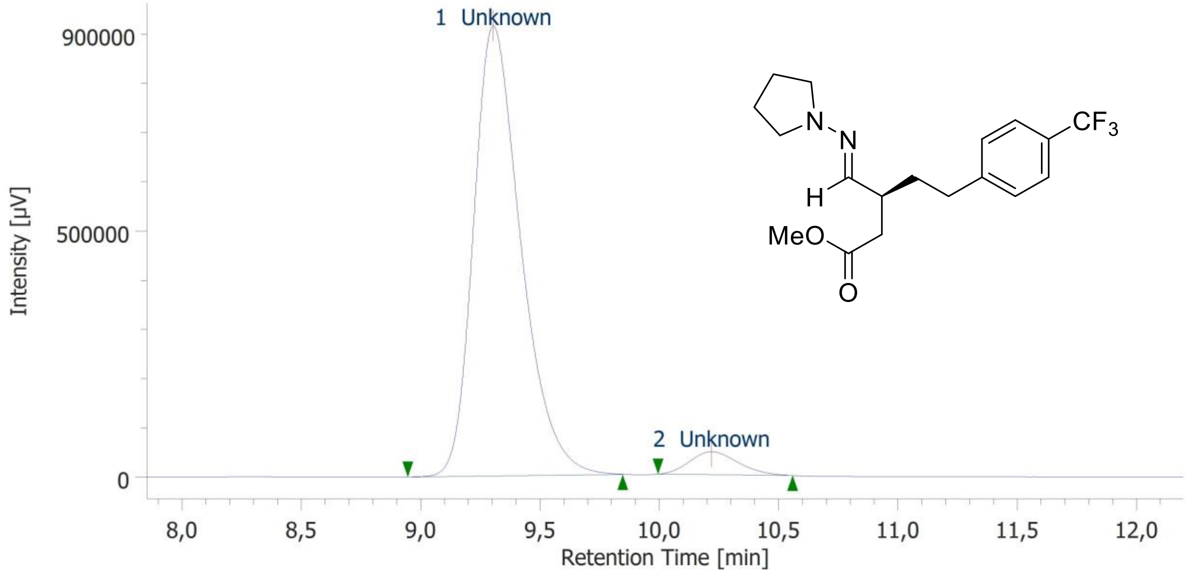


| # | Peak Name | CH | tR [min] | Area [µV·sec] | Height [µV] | Area% | Height% | Quantity | NTP | Resolution | Symmetry Factor | Warning |
|---|---|---|---|---|---|---|---|---|---|---|---|---|
| 1 | Unknown | 11 | 9,303 | 12605993 | 914612 | 95,043 | 95,144 | N/A | 11029 | 2,519 | 1,351 | |
| 2 | Unknown | 11 | 10,218 | 657492 | 46676 | 4,957 | 4,856 | N/A | 11931 | N/A | 1,200 | |

**4x:** Chiralpak IA, hexane/iPrOH 99/1, flow rate = 1.0 mL/min, λ = 248 nm

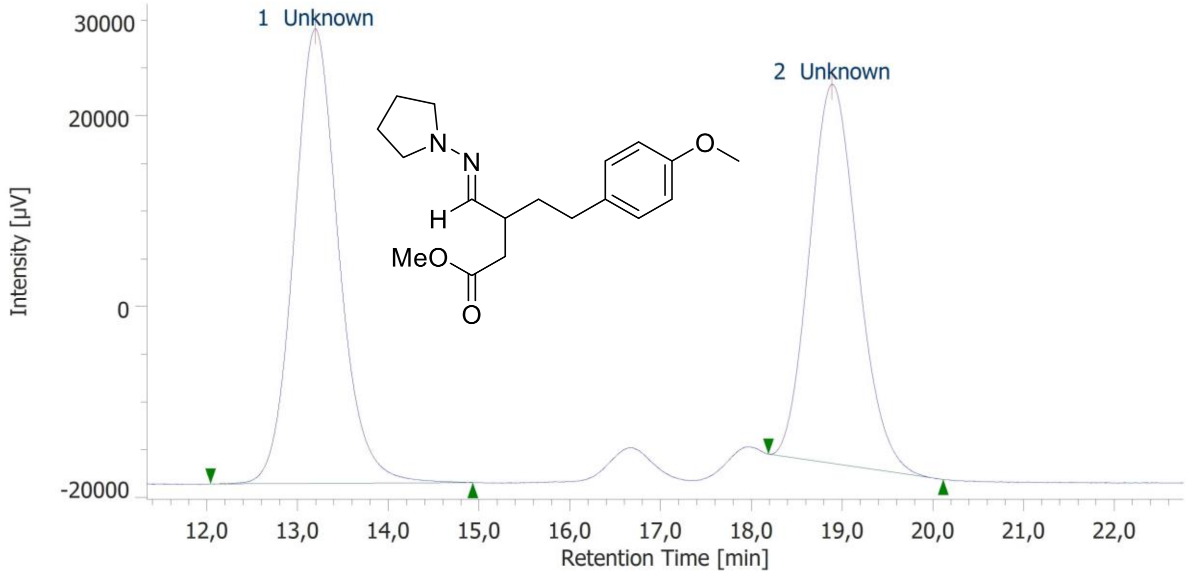


| # | Peak Name | CH | tR [min] | Area [µV·sec] | Height [µV] | Area% | Height% | Quantity | NTP | Resolution | Symmetry Factor | Warning |
|---|---|---|---|---|---|---|---|---|---|---|---|---|
| 1 | Unknown | 11 | 13,195 | 1635315 | 47604 | 52,680 | 54,538 | N/A | 3641 | 6,158 | 1,128 | |
| 2 | Unknown | 11 | 18,887 | 1468907 | 39682 | 47,320 | 45,462 | N/A | 5956 | N/A | 1,156 | |

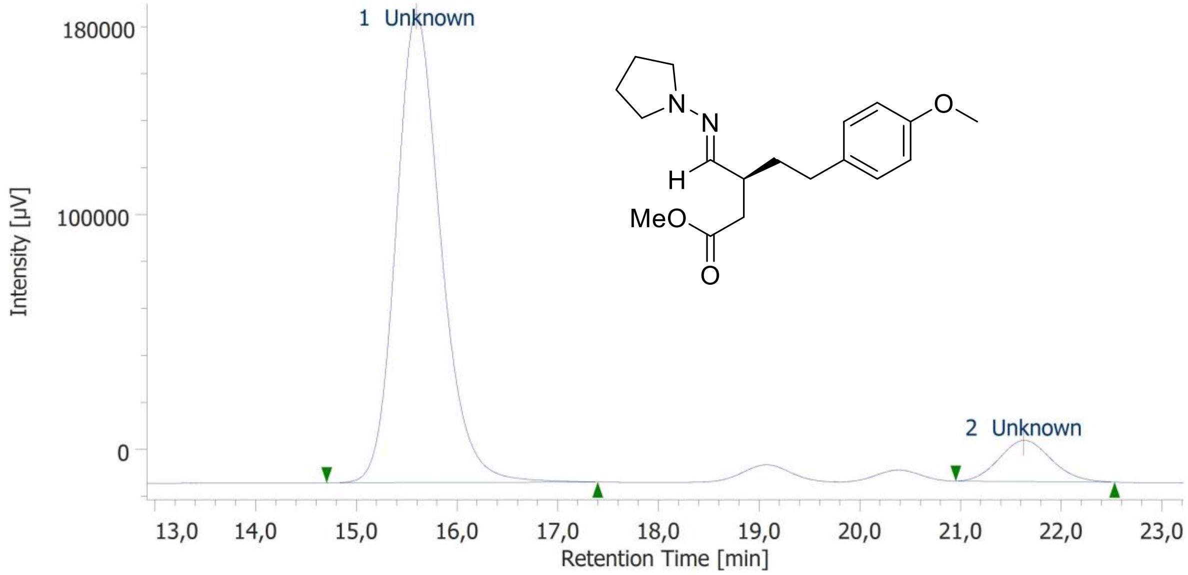


| # | Peak Name | CH | tR [min] | Area [µV·sec] | Height [µV] | Area% | Height% | Quantity | NTP | Resolution | Symmetry Factor | Warning |
|---|---|---|---|---|---|---|---|---|---|---|---|---|
| 1 | Unknown | 11 | 15,597 | 6162270 | 199414 | 90,743 | 91,940 | N/A | 6240 | 6,938 | 1,202 | |
| 2 | Unknown | 11 | 21,623 | 628666 | 17481 | 9,257 | 8,060 | N/A | 8252 | N/A | 1,120 | |

**4y:** Chiralpak IA, hexane/iPrOH 95/5, flow rate = 1.0 mL/min, λ = 248 nm

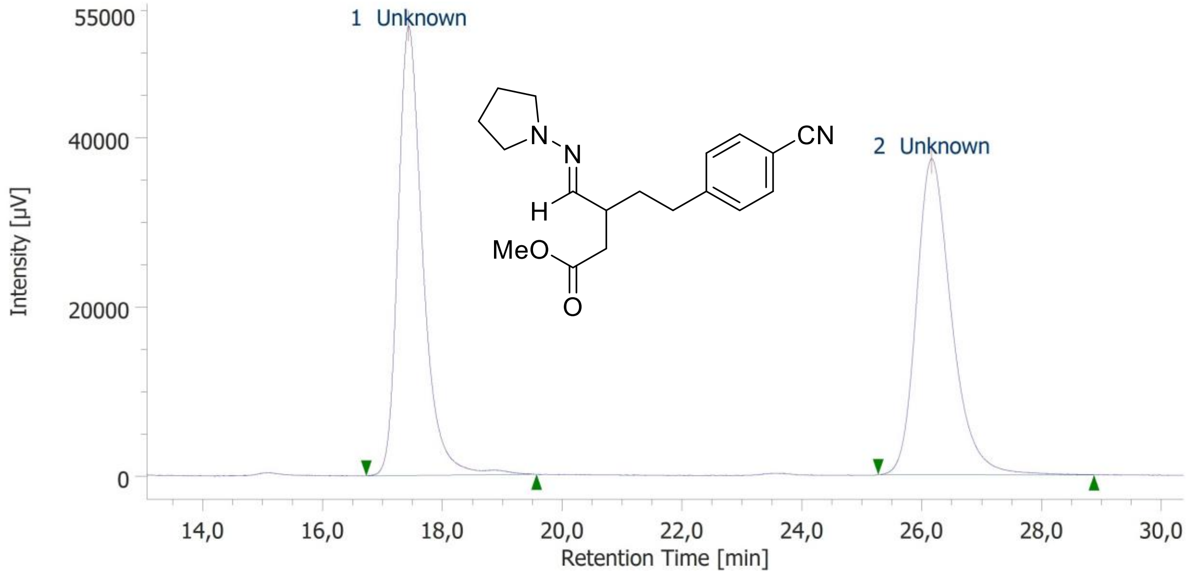


| # | Peak Name | CH | tR [min] | Area [µV·sec] | Height [µV] | Area% | Height% | Quantity | NTP | Resolution | Symmetry Factor | Warning |
|---|---|---|---|---|---|---|---|---|---|---|---|---|
| 1 | Unknown | 11 | 17,435 | 1528032 | 53048 | 50,157 | 58,712 | N/A | 9533 | 10,057 | 1,345 | |
| 2 | Unknown | 11 | 26,160 | 1518450 | 37304 | 49,843 | 41,288 | N/A | 10414 | N/A | 1,292 | |

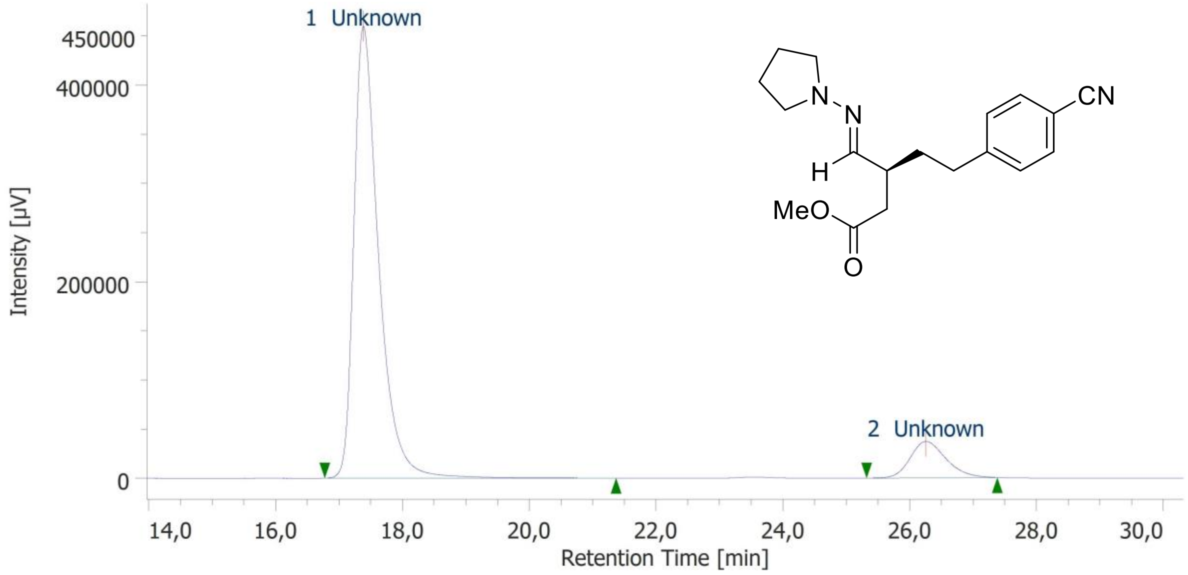


| # | Peak Name | CH | tR [min] | Area [µV·sec] | Height [µV] | Area% | Height% | Quantity | NTP | Resolution | Symmetry Factor | Warning |
|---|---|---|---|---|---|---|---|---|---|---|---|---|
| 1 | Unknown | 11 | 17,385 | 12486129 | 459566 | 89,643 | 92,564 | N/A | 10657 | 10,559 | 1,544 | |
| 2 | Unknown | 11 | 26,252 | 1442637 | 36920 | 10,357 | 7,436 | N/A | 10803 | N/A | 1,233 | |

**4ab:** Chiralcel ODH, hexane/EtOAc 98/2, flow rate = 1.0 mL/min, λ = 248 nm, 298 K

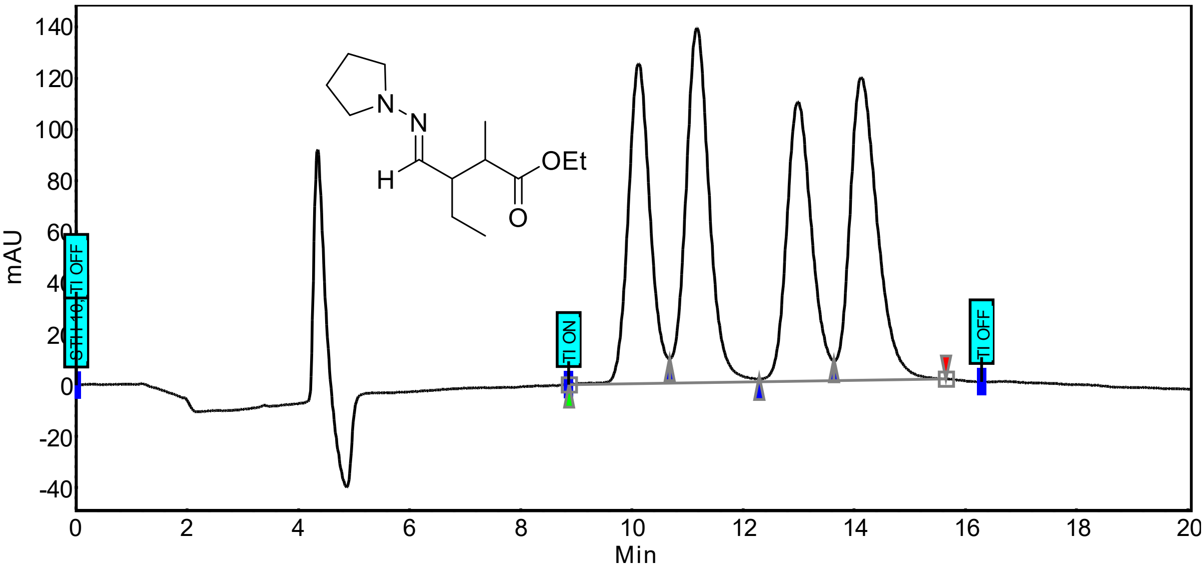


| Index | Name | Time [Min] | Quantity [% Area] | Height [mAU] | Area [mAU.Min] | Area % [%] |
|---|---|---|---|---|---|---|
| 1 | UNKNOWN | 10.119 | 22.88 | 125.1 | 57.2 | 22.884 |
| 2 | UNKNOWN | 11.173 | 27.14 | 138.6 | 67.8 | 27.144 |
| 3 | UNKNOWN | 12.986 | 22.83 | 109.2 | 57.0 | 22.826 |
| 4 | UNKNOWN | 14.119 | 27.15 | 118.4 | 67.8 | 27.146 |
| | | | | | | |
| Total | | | 100.00 | 491.2 | 249.8 | 100.000 |

Chiralcel ODH, hexane/EtOAc 98/2, flow rate = 1.0 mL/min, λ = 248 nm, 298 K

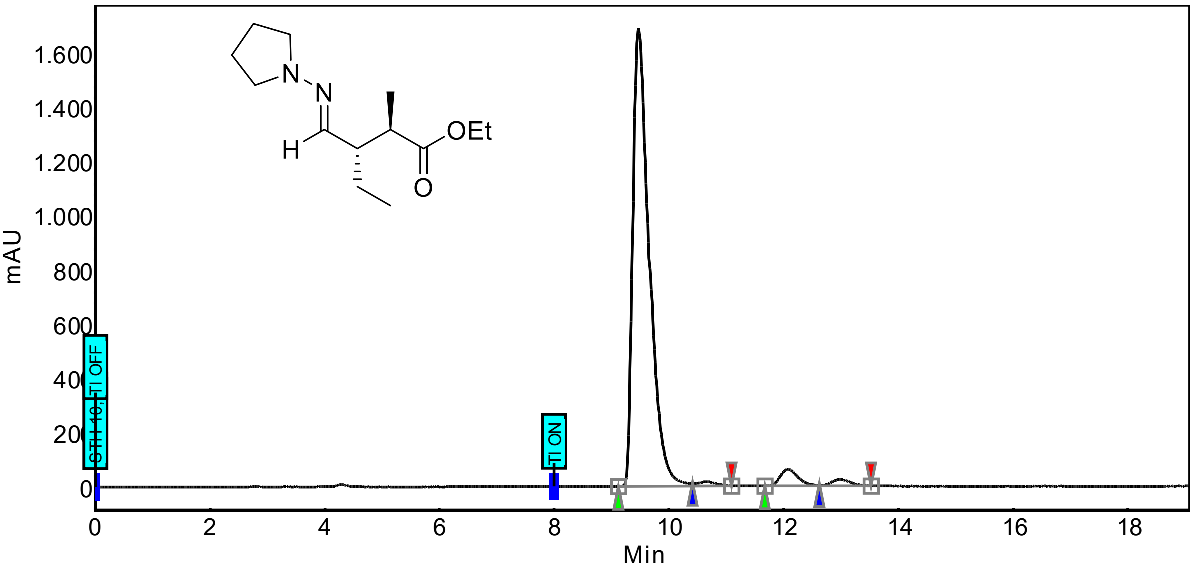


| Index | Name | Time [Min] | Quantity [% Area] | Height [mAU] | Area [mAU.Min] | Area % [%] |
|---|---|---|---|---|---|---|
| 1 | UNKNOWN | 9.466 | 93.89 | 1695.8 | 565.8 | 93.885 |
| 2 | UNKNOWN | 10.653 | 0.92 | 15.4 | 5.5 | 0.920 |
| 3 | UNKNOWN | 12.066 | 3.68 | 61.7 | 22.2 | 3.680 |
| 4 | UNKNOWN | 12.972 | 1.51 | 23.8 | 9.1 | 1.515 |
| | | | | | | |
| Total | | | 100.00 | 1796.7 | 602.7 | 100.000 |

Chiralcel ODH, hexane/EtOAc 98/2, flow rate = 1.0 mL/min, λ = 248 nm, 298 K

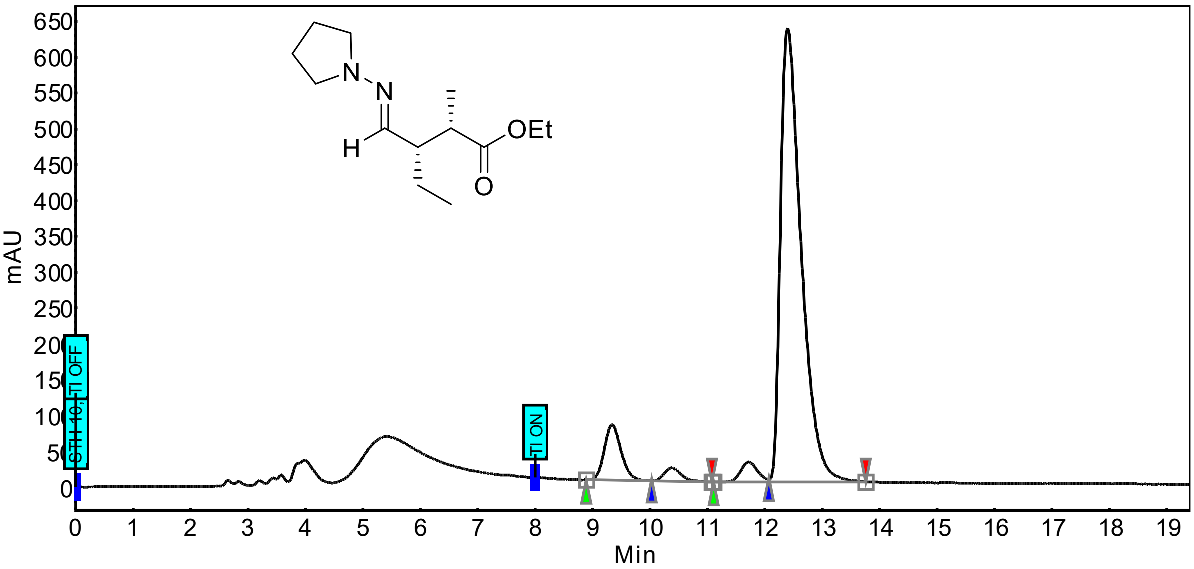


| Index | Name | Time [Min] | Quantity [% Area] | Height [mAU] | Area [mAU.Min] | Area % [%] |
|---|---|---|---|---|---|---|
| 1 | UNKNOWN | 9.346 | 8.58 | 77.3 | 26.4 | 8.584 |
| 2 | UNKNOWN | 10.386 | 2.09 | 18.8 | 6.4 | 2.090 |
| 3 | UNKNOWN | 11.719 | 3.04 | 27.7 | 9.3 | 3.038 |
| 4 | UNKNOWN | 12.399 | 86.29 | 633.3 | 265.4 | 86.288 |
| | | | | | | |
| Total | | | 100.00 | 757.2 | 307.6 | 100.000 |

**4ac:** Chiralcel ODH, hexane/EtOAc 98/2, flow rate = 1.0 mL/min, λ = 248 nm, 298 K

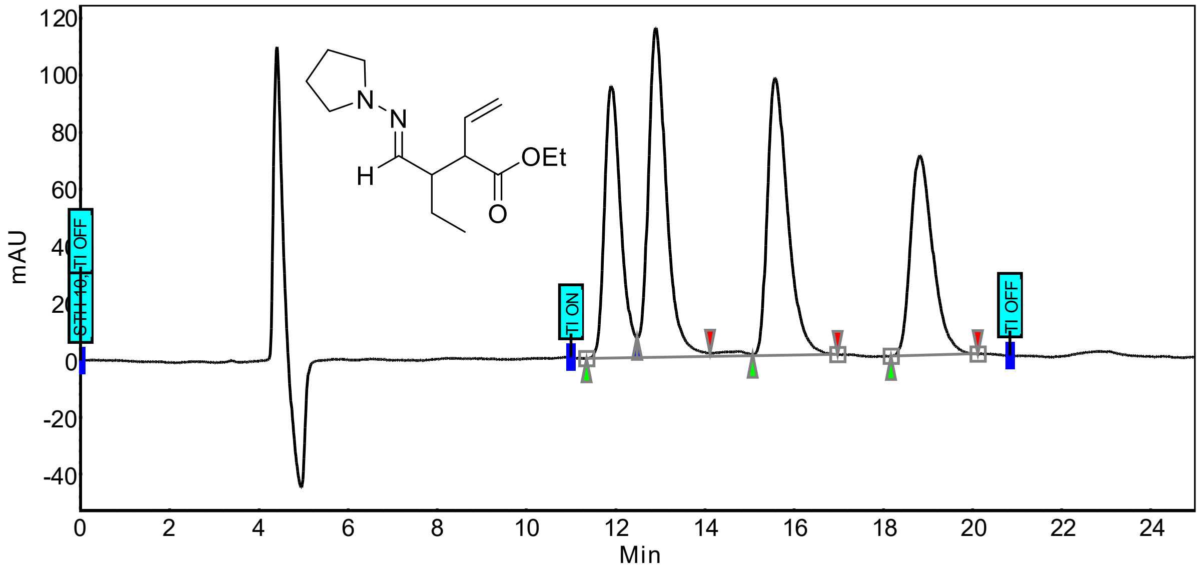


| Index | Name | Time [Min] | Quantity [% Area] | Height [mAU] | Area [mAU.Min] | Area % [%] |
|---|---|---|---|---|---|---|
| 1 | UNKNOWN | 11.906 | 21.62 | 95.4 | 40.5 | 21.615 |
| 2 | UNKNOWN | 12.892 | 29.08 | 115.5 | 54.5 | 29.080 |
| 3 | UNKNOWN | 15.572 | 27.55 | 97.4 | 51.6 | 27.550 |
| 4 | UNKNOWN | 18.812 | 21.75 | 70.2 | 40.8 | 21.754 |
| | | | | | | |
| Total | | | 100.00 | 378.6 | 187.4 | 100.000 |

Chiralcel ODH, hexane/EtOAc 98/2, flow rate = 1.0 mL/min, λ = 248 nm, 298 K

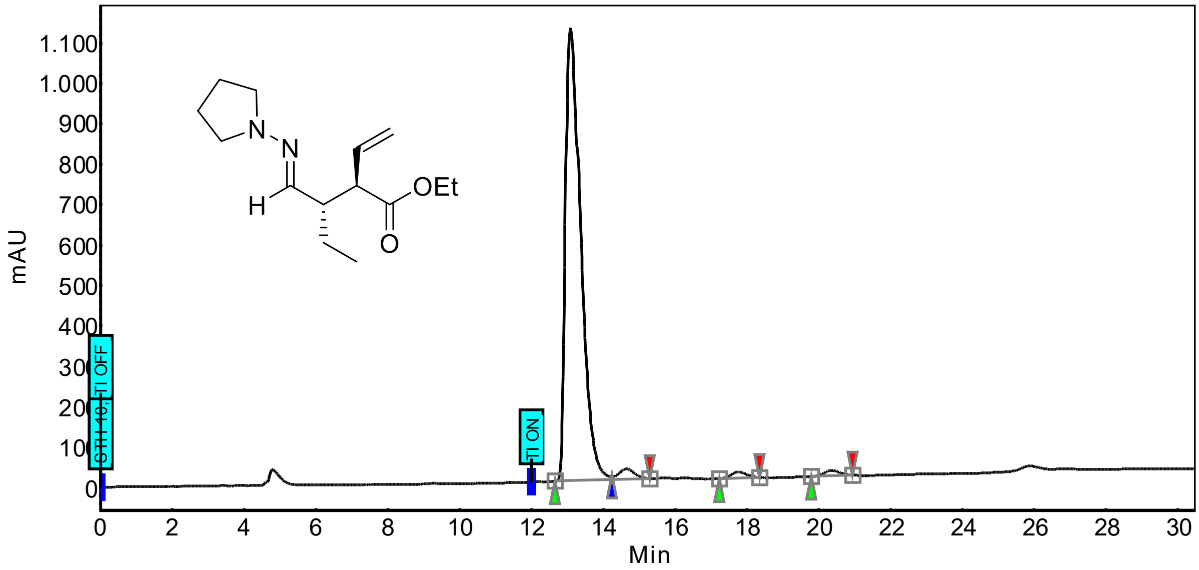


| Index | Name | Time [Min] | Quantity [% Area] | Height [mAU] | Area [mAU.Min] | Area % [%] |
|---|---|---|---|---|---|---|
| 1 | UNKNOWN | 13.079 | 95.33 | 1122.0 | 557.5 | 95.333 |
| 2 | UNKNOWN | 14.639 | 2.23 | 26.3 | 13.1 | 2.233 |
| 3 | UNKNOWN | 17.759 | 1.29 | 15.3 | 7.5 | 1.286 |
| 4 | UNKNOWN | 20.345 | 1.15 | 13.1 | 6.7 | 1.147 |
| | | | | | | |
| Total | | | 100.00 | 1176.8 | 584.8 | 100.000 |

**5g:** Chiralpak IC, hexane/iPrOH 98/2, flow rate = 1.0 mL/min, λ = 212 nm

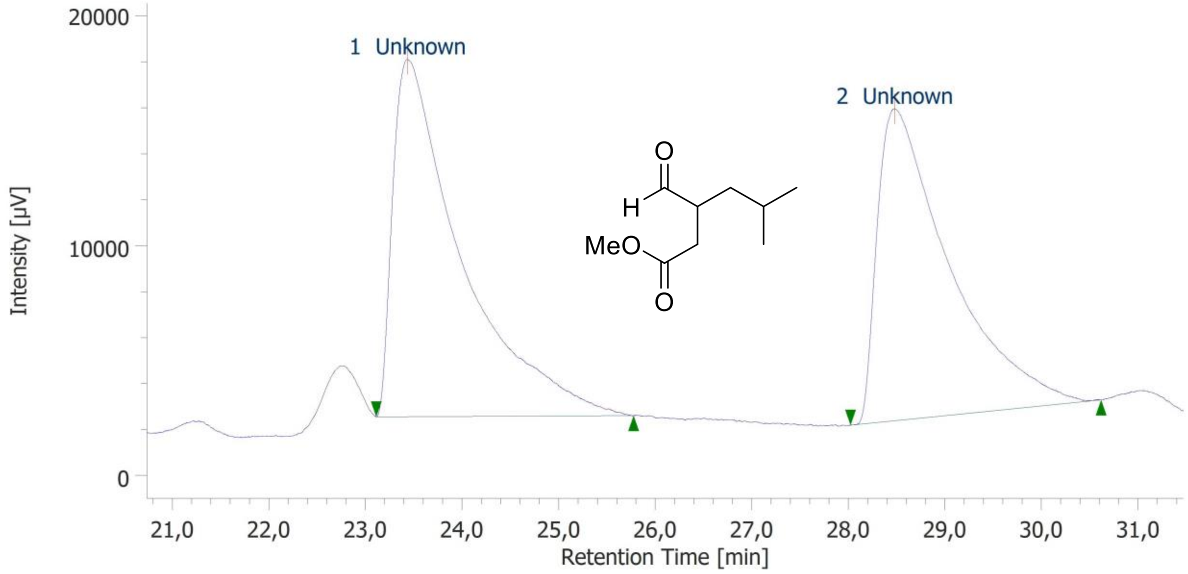


| # | Peak Name | CH | tR [min] | Area [µV·sec] | Height [µV] | Area% | Height% | Quantity | NTP | Resolution | Symmetry Factor | Warning |
|---|---|---|---|---|---|---|---|---|---|---|---|---|
| 1 | Unknown | 11 | 23,433 | 762656 | 15540 | 52,024 | 53,385 | N/A | 7008 | 4,223 | 3,764 | |
| 2 | Unknown | 11 | 28,477 | 703315 | 13569 | 47,976 | 46,615 | N/A | 7979 | N/A | 3,037 | |

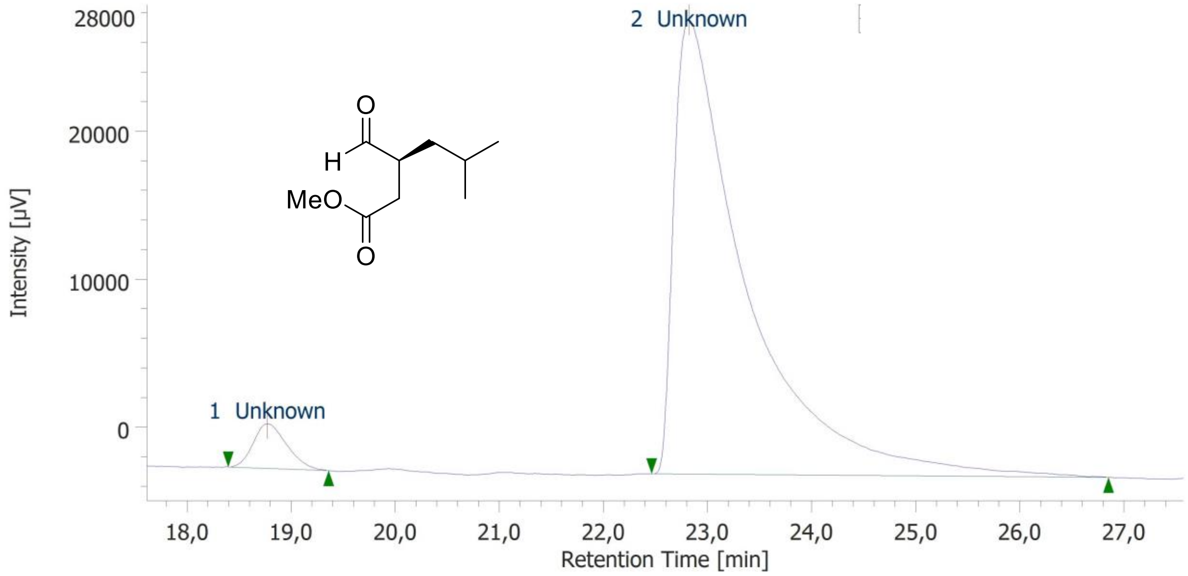


| # | Peak Name | CH | tR [min] | Area [µV·sec] | Height [µV] | Area% | Height% | Quantity | NTP | Resolution | Symmetry Factor | Warning |
|---|---|---|---|---|---|---|---|---|---|---|---|---|
| 1 | Unknown | 11 | 18,770 | 67419 | 3020 | 4,376 | 8,963 | N/A | 16147 | 4,973 | 1,272 | |
| 2 | Unknown | 11 | 22,820 | 1473372 | 30676 | 95,624 | 91,037 | N/A | 7671 | N/A | 4,151 | |

# Crystallographic data

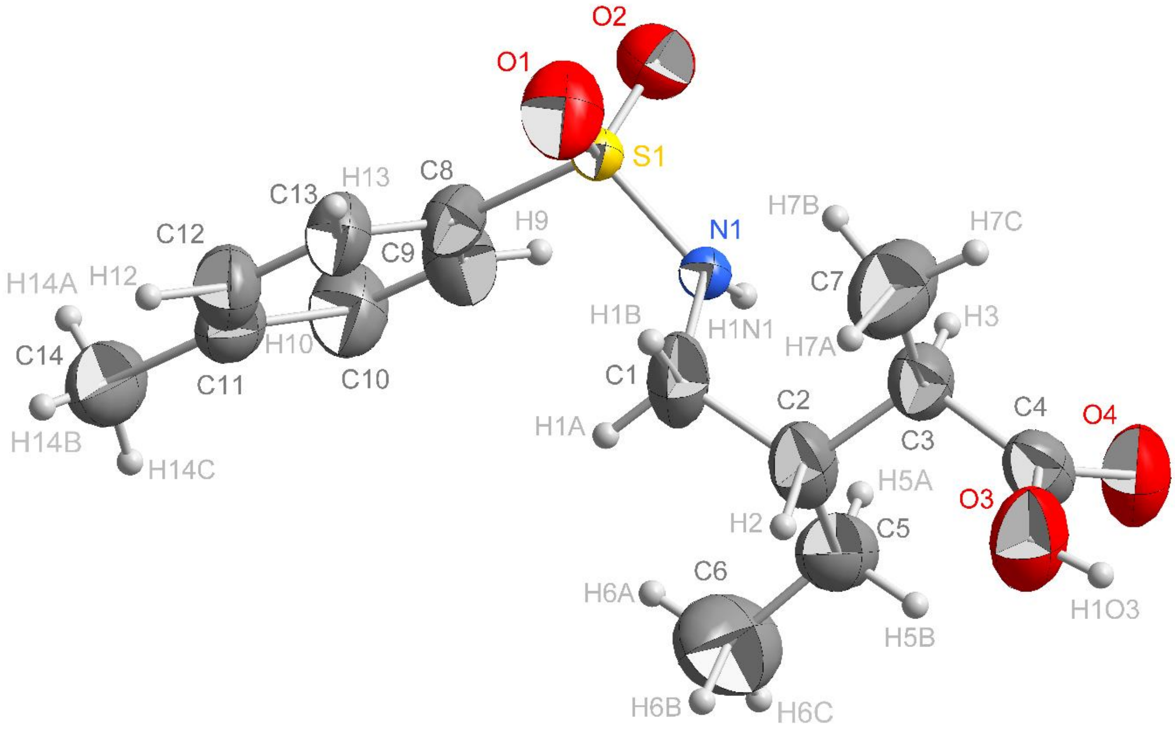


**Supplementary Figure 14:** crystal structure of the sulfonamido acid derived from *anti*-**4ab**, **Cry1**.

Table 1. Crystal data and structure refinement for x3714fin.

| | | |
|---|---|---|
| Identification code | x3714fin | |
| Empirical formula | C14 H21 N O4 S | |
| Formula weight | 299.38 | |
| Temperature | 298(2) K | |
| Wavelength | 71.073 pm | |
| Crystal system | Triclinic | |
| Space group | P 1 | |
| Unit cell dimensions | a = 820.75(3) pm | $\alpha$= 77.794(4)°. |
| | b = 990.23(5) pm | $\beta$= 79.401(4)°. |
| | c = 1028.69(5) pm | $\gamma$ = 82.542(4)°. |
| Volume | 0.79960(7) $nm^3$ | |
| Z | 2 | |
| Density (calculated) | 1.243 $Mg/m^3$ | |
| Absorption coefficient | 0.214 $mm^{-1}$ | |
| F(000) | 320 | |
| Crystal size | 0.48 x 0.27 x 0.06 $mm^3$ | |
| Theta range for data collection | 2.536 to 29.014°. | |

| | |
|---|---|
| Index ranges | -11<=h<=11, -13<=k<=13, -13<=l<=13 |
| Reflections collected | 22645 |
| Independent reflections | 7390 [R(int) = 0.0266] |
| Completeness to theta = 25.350° | 99.9 % |
| Absorption correction | Semi-empirical from equivalents |
| Max. and min. transmission | 1.00000 and 0.99167 |
| Refinement method | Full-matrix least-squares on $F^2$ |
| Data / restraints / parameters | 7390 / 122 / 466 |
| Goodness-of-fit on $F^2$ | 1.016 |
| Final R indices [I>2sigma(I)] | R1 = 0.0496, wR2 = 0.1190 |
| R indices (all data) | R1 = 0.0776, wR2 = 0.1353 |
| Absolute structure parameter | -0.01(3) |
| Largest diff. peak and hole | 0.304 and -0.246 e.Å$^{-3}$ |

**Comments:** Structure solution with SHELXT-2018 (dual-space method). Anisotropic refinement of all non-hydrogen atoms with SHELXL-2018. Except NH, all hydrogen atoms were calculated on idealized positions. One substituent (C15 to C21, N2, O7 and O8) is disordered on two positions with a ratio of 0.614(6) : 0.386(6). As a result of a relatively short bond length (C5-C6 = 145.5 pm), the same substituent of the second molecule (C1 to C7, N1, O3 and O4) may be marginally disordered as well. However, all attempts to describe this disorder failed and the bond length of C5-C6 had been restraint with a DFIX command. C2, C16, C16F are S- and C3, C17 and C17F are R-configured. For significant intermolecular hydrogen donor acceptor bonds check Table 6. With these intermolecular donor acceptor bonds zig-zag chains along (1 1 $\bar{1}$) are formed.

Table 2. Atomic coordinates ( x $10^4$) and equivalent isotropic displacement parameters ($pm^2$x $10^{-1}$) for x3714fin. U(eq) is defined as one third of the trace of the orthogonalized $U^{ij}$ tensor.

| | x | y | z | U(eq) |
|---|---|---|---|---|
| S(1) | 1695(3) | -1816(2) | 9436(2) | 58(1) |
| S(2) | 8165(3) | 11832(2) | 556(2) | 57(1) |
| O(1) | 3291(5) | -2558(5) | 9291(5) | 79(1) |
| O(2) | 983(6) | -1479(5) | 10716(4) | 70(1) |
| O(5) | 6560(5) | 12576(5) | 755(4) | 73(1) |
| O(6) | 8875(5) | 11558(5) | -765(4) | 67(1) |
| O(3) | 5409(6) | 3231(4) | 5105(4) | 91(1) |
| O(4) | 3620(6) | 4311(4) | 6530(4) | 83(1) |
| N(1) | 1811(6) | -346(4) | 8390(4) | 60(1) |
| C(1) | 2606(7) | -336(4) | 7000(5) | 71(2) |
| C(2) | 2889(5) | 1151(4) | 6272(4) | 66(1) |
| C(3) | 3922(5) | 1828(4) | 6991(4) | 60(1) |
| C(4) | 4347(6) | 3231(5) | 6183(5) | 63(1) |
| C(5) | 1285(6) | 2044(5) | 5984(5) | 73(2) |
| C(6) | 265(9) | 1478(9) | 5186(9) | 133(3) |
| C(7) | 5497(8) | 975(6) | 7352(7) | 109(3) |
| C(8) | 298(7) | -2752(6) | 8963(5) | 52(1) |
| C(9) | -1386(7) | -2294(7) | 9136(6) | 69(2) |
| C(10) | -2501(8) | -3019(7) | 8796(7) | 78(2) |
| C(11) | -1947(9) | -4189(7) | 8230(6) | 68(2) |
| C(12) | -284(9) | -4619(7) | 8069(7) | 74(2) |
| C(13) | 849(8) | -3909(6) | 8436(6) | 62(1) |
| C(14) | -3163(10) | -4998(8) | 7828(8) | 98(2) |
| C(22) | 9577(8) | 12726(6) | 1072(6) | 55(1) |
| C(23) | 11231(8) | 12311(7) | 838(7) | 79(2) |
| C(24) | 12335(9) | 13007(9) | 1224(9) | 90(2) |
| C(25) | 11847(10) | 14129(8) | 1822(7) | 82(2) |
| C(26) | 10150(11) | 14533(7) | 2054(7) | 88(2) |
| C(27) | 9005(8) | 13834(7) | 1682(7) | 72(2) |
| C(28) | 13076(12) | 14871(10) | 2242(9) | 120(3) |
| O(7) | 3915(9) | 6795(6) | 5244(7) | 92(2) |
| O(8) | 5961(9) | 5924(5) | 3836(8) | 78(2) |
| N(2) | 8201(13) | 10280(7) | 1452(9) | 51(2) |
| C(15) | 7569(10) | 10145(6) | 2888(8) | 66(3) |

| | | | | |
|---|---|---|---|---|
| C(16) | 7250(6) | 8638(6) | 3510(5) | 70(3) |
| C(17) | 5448(6) | 8395(5) | 3584(5) | 73(2) |
| C(18) | 5120(7) | 6927(5) | 4256(6) | 61(2) |
| C(19) | 7890(8) | 8113(9) | 4847(7) | 81(3) |
| C(20) | 9753(9) | 7900(14) | 4741(11) | 111(4) |
| C(21) | 4795(10) | 8757(10) | 2260(7) | 73(3) |
| O(7F) | 5059(13) | 6657(9) | 5124(11) | 83(4) |
| O(8F) | 6772(15) | 5392(8) | 3818(13) | 96(5) |
| N(2F) | 7820(20) | 10423(9) | 1647(15) | 51(2) |
| C(15F) | 7082(16) | 10384(7) | 3054(15) | 75(5) |
| C(16F) | 6109(9) | 9110(7) | 3595(7) | 74(3) |
| C(17F) | 7235(7) | 7770(6) | 3605(7) | 67(3) |
| C(18F) | 6267(10) | 6523(6) | 4164(9) | 64(3) |
| C(19F) | 4585(10) | 9143(12) | 2930(13) | 114(6) |
| C(20F) | 4950(20) | 9000(30) | 1478(15) | 162(9) |
| C(21F) | 8703(11) | 7649(12) | 4335(15) | 91(6) |

Table 3. Bond lengths [pm] and angles [°] for x3714fin.

| | |
|---|---|
| S(1)-O(1) | 141.5(4) |
| S(1)-O(2) | 142.8(4) |
| S(1)-N(1) | 161.9(4) |
| S(1)-C(8) | 175.5(5) |
| S(2)-O(5) | 142.6(4) |
| S(2)-O(6) | 144.4(4) |
| S(2)-N(2) | 161.5(4) |
| S(2)-N(2F) | 161.5(4) |
| S(2)-C(22) | 176.1(6) |
| O(3)-C(4) | 127.9(5) |
| O(3)-H(1O3) | 82.00 |
| O(4)-C(4) | 124.3(5) |
| N(1)-C(1) | 145.7(4) |
| N(1)-H(1N1) | 97.97(15) |
| C(1)-C(2) | 153.0(5) |
| C(1)-H(1A) | 97.00 |
| C(1)-H(1B) | 97.00 |
| C(2)-C(3) | 151.8(5) |
| C(2)-C(5) | 152.8(6) |
| C(2)-H(2) | 98.00 |
| C(3)-C(4) | 150.8(5) |
| C(3)-C(7) | 151.2(6) |
| C(3)-H(3) | 98.00 |
| C(5)-C(6) | 150.3(3) |
| C(5)-H(5A) | 97.00 |
| C(5)-H(5B) | 97.00 |
| C(6)-H(6A) | 96.00 |
| C(6)-H(6B) | 96.00 |
| C(6)-H(6C) | 96.00 |
| C(7)-H(7A) | 96.00 |
| C(7)-H(7B) | 96.00 |
| C(7)-H(7C) | 96.00 |
| C(8)-C(13) | 136.0(7) |
| C(8)-C(9) | 138.9(7) |
| C(9)-C(10) | 136.8(8) |
| C(9)-H(9) | 93.00 |
| C(10)-C(11) | 139.2(9) |

| | |
|---|---|
| C(10)-H(10) | 93.00 |
| C(11)-C(12) | 136.6(8) |
| C(11)-C(14) | 151.9(8) |
| C(12)-C(13) | 138.3(8) |
| C(12)-H(12) | 93.00 |
| C(13)-H(13) | 93.00 |
| C(14)-H(14A) | 96.00 |
| C(14)-H(14B) | 96.00 |
| C(14)-H(14C) | 96.00 |
| C(22)-C(23) | 135.9(8) |
| C(22)-C(27) | 136.6(8) |
| C(23)-C(24) | 136.5(9) |
| C(23)-H(23) | 93.00 |
| C(24)-C(25) | 136.2(11) |
| C(24)-H(24) | 93.00 |
| C(25)-C(26) | 138.9(10) |
| C(25)-C(28) | 149.4(10) |
| C(26)-C(27) | 138.6(9) |
| C(26)-H(26) | 93.00 |
| C(27)-H(27) | 93.00 |
| C(28)-H(28A) | 96.00 |
| C(28)-H(28B) | 96.00 |
| C(28)-H(28C) | 96.00 |
| O(7)-C(18) | 127.9(5) |
| O(7)-H(1O7) | 82.00 |
| O(8)-C(18) | 124.3(5) |
| N(2)-C(15) | 145.7(4) |
| N(2)-H(1N2) | 97.99(15) |
| C(15)-C(16) | 153.0(5) |
| C(15)-H(15A) | 97.00 |
| C(15)-H(15B) | 97.00 |
| C(16)-C(17) | 151.5(5) |
| C(16)-C(19) | 152.7(6) |
| C(16)-H(16) | 98.00 |
| C(17)-C(18) | 150.7(5) |
| C(17)-C(21) | 151.1(6) |
| C(17)-H(17) | 98.00 |
| C(19)-C(20) | 150.2(3) |
| C(19)-H(19A) | 97.00 |

| | |
|---|---|
| C(19)-H(19B) | 97.00 |
| C(20)-H(20A) | 96.00 |
| C(20)-H(20B) | 96.00 |
| C(20)-H(20C) | 96.00 |
| C(21)-H(21A) | 96.00 |
| C(21)-H(21B) | 96.00 |
| C(21)-H(21C) | 96.00 |
| O(7F)-C(18F) | 127.9(5) |
| O(7F)-H(1OF) | 82.00 |
| O(8F)-C(18F) | 124.4(5) |
| N(2F)-C(15F) | 145.7(4) |
| N(2F)-H(1NF) | 98.00(15) |
| C(15F)-C(16F) | 152.9(5) |
| C(15F)-H(15C) | 97.00 |
| C(15F)-H(15D) | 97.00 |
| C(16F)-C(17F) | 151.5(5) |
| C(16F)-C(19F) | 152.6(6) |
| C(16F)-H(16F) | 98.00 |
| C(17F)-C(18F) | 150.8(5) |
| C(17F)-C(21F) | 151.2(6) |
| C(17F)-H(17F) | 98.00 |
| C(19F)-C(20F) | 150.2(3) |
| C(19F)-H(19C) | 97.00 |
| C(19F)-H(19D) | 97.00 |
| C(20F)-H(20D) | 96.00 |
| C(20F)-H(20E) | 96.00 |
| C(20F)-H(20F) | 96.00 |
| C(21F)-H(21D) | 96.00 |
| C(21F)-H(21E) | 96.00 |
| C(21F)-H(21F) | 96.00 |
| | |
| O(1)-S(1)-O(2) | 118.9(3) |
| O(1)-S(1)-N(1) | 107.4(3) |
| O(2)-S(1)-N(1) | 105.6(2) |
| O(1)-S(1)-C(8) | 108.3(3) |
| O(2)-S(1)-C(8) | 108.1(2) |
| N(1)-S(1)-C(8) | 108.1(2) |
| O(5)-S(2)-O(6) | 119.5(2) |
| O(5)-S(2)-N(2) | 112.0(3) |

| | |
|---|---|
| O(6)-S(2)-N(2) | 101.3(4) |
| O(5)-S(2)-N(2F) | 99.5(5) |
| O(6)-S(2)-N(2F) | 112.2(6) |
| O(5)-S(2)-C(22) | 107.9(3) |
| O(6)-S(2)-C(22) | 107.8(2) |
| N(2)-S(2)-C(22) | 107.7(6) |
| N(2F)-S(2)-C(22) | 109.4(9) |
| C(4)-O(3)-H(1O3) | 109.5 |
| C(1)-N(1)-S(1) | 118.9(3) |
| C(1)-N(1)-H(1N1) | 119(2) |
| S(1)-N(1)-H(1N1) | 110.0(18) |
| N(1)-C(1)-C(2) | 110.1(3) |
| N(1)-C(1)-H(1A) | 109.6 |
| C(2)-C(1)-H(1A) | 109.6 |
| N(1)-C(1)-H(1B) | 109.6 |
| C(2)-C(1)-H(1B) | 109.6 |
| H(1A)-C(1)-H(1B) | 108.2 |
| C(3)-C(2)-C(5) | 112.5(3) |
| C(3)-C(2)-C(1) | 111.5(3) |
| C(5)-C(2)-C(1) | 113.4(3) |
| C(3)-C(2)-H(2) | 106.3 |
| C(5)-C(2)-H(2) | 106.3 |
| C(1)-C(2)-H(2) | 106.3 |
| C(4)-C(3)-C(7) | 109.3(3) |
| C(4)-C(3)-C(2) | 110.8(3) |
| C(7)-C(3)-C(2) | 115.2(3) |
| C(4)-C(3)-H(3) | 107.0 |
| C(7)-C(3)-H(3) | 107.0 |
| C(2)-C(3)-H(3) | 107.0 |
| O(4)-C(4)-O(3) | 123.1(4) |
| O(4)-C(4)-C(3) | 120.6(3) |
| O(3)-C(4)-C(3) | 116.2(3) |
| C(6)-C(5)-C(2) | 115.1(4) |
| C(6)-C(5)-H(5A) | 108.5 |
| C(2)-C(5)-H(5A) | 108.5 |
| C(6)-C(5)-H(5B) | 108.5 |
| C(2)-C(5)-H(5B) | 108.5 |
| H(5A)-C(5)-H(5B) | 107.5 |
| C(5)-C(6)-H(6A) | 109.5 |

| | |
|---|---|
| C(5)-C(6)-H(6B) | 109.5 |
| H(6A)-C(6)-H(6B) | 109.5 |
| C(5)-C(6)-H(6C) | 109.5 |
| H(6A)-C(6)-H(6C) | 109.5 |
| H(6B)-C(6)-H(6C) | 109.5 |
| C(3)-C(7)-H(7A) | 109.5 |
| C(3)-C(7)-H(7B) | 109.5 |
| H(7A)-C(7)-H(7B) | 109.5 |
| C(3)-C(7)-H(7C) | 109.5 |
| H(7A)-C(7)-H(7C) | 109.5 |
| H(7B)-C(7)-H(7C) | 109.5 |
| C(13)-C(8)-C(9) | 120.2(5) |
| C(13)-C(8)-S(1) | 120.8(4) |
| C(9)-C(8)-S(1) | 119.0(4) |
| C(10)-C(9)-C(8) | 120.0(5) |
| C(10)-C(9)-H(9) | 120.0 |
| C(8)-C(9)-H(9) | 120.0 |
| C(9)-C(10)-C(11) | 120.2(6) |
| C(9)-C(10)-H(10) | 119.9 |
| C(11)-C(10)-H(10) | 119.9 |
| C(12)-C(11)-C(10) | 118.7(6) |
| C(12)-C(11)-C(14) | 120.3(7) |
| C(10)-C(11)-C(14) | 121.0(6) |
| C(11)-C(12)-C(13) | 121.6(6) |
| C(11)-C(12)-H(12) | 119.2 |
| C(13)-C(12)-H(12) | 119.2 |
| C(8)-C(13)-C(12) | 119.3(6) |
| C(8)-C(13)-H(13) | 120.4 |
| C(12)-C(13)-H(13) | 120.4 |
| C(11)-C(14)-H(14A) | 109.5 |
| C(11)-C(14)-H(14B) | 109.5 |
| H(14A)-C(14)-H(14B) | 109.5 |
| C(11)-C(14)-H(14C) | 109.5 |
| H(14A)-C(14)-H(14C) | 109.5 |
| H(14B)-C(14)-H(14C) | 109.5 |
| C(23)-C(22)-C(27) | 120.9(6) |
| C(23)-C(22)-S(2) | 119.2(5) |
| C(27)-C(22)-S(2) | 119.9(5) |
| C(22)-C(23)-C(24) | 119.5(7) |

| | |
|---|---|
| C(22)-C(23)-H(23) | 120.2 |
| C(24)-C(23)-H(23) | 120.2 |
| C(25)-C(24)-C(23) | 122.4(7) |
| C(25)-C(24)-H(24) | 118.8 |
| C(23)-C(24)-H(24) | 118.8 |
| C(24)-C(25)-C(26) | 117.1(7) |
| C(24)-C(25)-C(28) | 121.5(8) |
| C(26)-C(25)-C(28) | 121.4(8) |
| C(27)-C(26)-C(25) | 121.5(7) |
| C(27)-C(26)-H(26) | 119.2 |
| C(25)-C(26)-H(26) | 119.2 |
| C(22)-C(27)-C(26) | 118.6(6) |
| C(22)-C(27)-H(27) | 120.7 |
| C(26)-C(27)-H(27) | 120.7 |
| C(25)-C(28)-H(28A) | 109.5 |
| C(25)-C(28)-H(28B) | 109.5 |
| H(28A)-C(28)-H(28B) | 109.5 |
| C(25)-C(28)-H(28C) | 109.5 |
| H(28A)-C(28)-H(28C) | 109.5 |
| H(28B)-C(28)-H(28C) | 109.5 |
| C(18)-O(7)-H(1O7) | 109.5 |
| C(15)-N(2)-S(2) | 117.0(6) |
| C(15)-N(2)-H(1N2) | 118(2) |
| S(2)-N(2)-H(1N2) | 110(2) |
| N(2)-C(15)-C(16) | 110.2(3) |
| N(2)-C(15)-H(15A) | 109.6 |
| C(16)-C(15)-H(15A) | 109.6 |
| N(2)-C(15)-H(15B) | 109.6 |
| C(16)-C(15)-H(15B) | 109.6 |
| H(15A)-C(15)-H(15B) | 108.1 |
| C(17)-C(16)-C(19) | 113.1(3) |
| C(17)-C(16)-C(15) | 111.7(4) |
| C(19)-C(16)-C(15) | 113.3(4) |
| C(17)-C(16)-H(16) | 106.0 |
| C(19)-C(16)-H(16) | 106.0 |
| C(15)-C(16)-H(16) | 106.0 |
| C(18)-C(17)-C(21) | 109.5(4) |
| C(18)-C(17)-C(16) | 111.5(3) |
| C(21)-C(17)-C(16) | 115.6(4) |

| | |
|---|---|
| C(18)-C(17)-H(17) | 106.6 |
| C(21)-C(17)-H(17) | 106.6 |
| C(16)-C(17)-H(17) | 106.6 |
| O(8)-C(18)-O(7) | 123.2(4) |
| O(8)-C(18)-C(17) | 120.9(4) |
| O(7)-C(18)-C(17) | 115.9(4) |
| C(20)-C(19)-C(16) | 115.1(4) |
| C(20)-C(19)-H(19A) | 108.5 |
| C(16)-C(19)-H(19A) | 108.5 |
| C(20)-C(19)-H(19B) | 108.5 |
| C(16)-C(19)-H(19B) | 108.5 |
| H(19A)-C(19)-H(19B) | 107.5 |
| C(19)-C(20)-H(20A) | 109.5 |
| C(19)-C(20)-H(20B) | 109.5 |
| H(20A)-C(20)-H(20B) | 109.5 |
| C(19)-C(20)-H(20C) | 109.5 |
| H(20A)-C(20)-H(20C) | 109.5 |
| H(20B)-C(20)-H(20C) | 109.5 |
| C(17)-C(21)-H(21A) | 109.5 |
| C(17)-C(21)-H(21B) | 109.5 |
| H(21A)-C(21)-H(21B) | 109.5 |
| C(17)-C(21)-H(21C) | 109.5 |
| H(21A)-C(21)-H(21C) | 109.5 |
| H(21B)-C(21)-H(21C) | 109.5 |
| C(18F)-O(7F)-H(1OF) | 109.5 |
| C(15F)-N(2F)-S(2) | 124.3(9) |
| C(15F)-N(2F)-H(1NF) | 119(2) |
| S(2)-N(2F)-H(1NF) | 110(2) |
| N(2F)-C(15F)-C(16F) | 110.3(3) |
| N(2F)-C(15F)-H(15C) | 109.6 |
| C(16F)-C(15F)-H(15C) | 109.6 |
| N(2F)-C(15F)-H(15D) | 109.6 |
| C(16F)-C(15F)-H(15D) | 109.6 |
| H(15C)-C(15F)-H(15D) | 108.1 |
| C(17F)-C(16F)-C(19F) | 113.3(4) |
| C(17F)-C(16F)-C(15F) | 111.9(4) |
| C(19F)-C(16F)-C(15F) | 113.7(4) |
| C(17F)-C(16F)-H(16F) | 105.7 |
| C(19F)-C(16F)-H(16F) | 105.7 |

| | |
|---|---|
| C(15F)-C(16F)-H(16F) | 105.7 |
| C(18F)-C(17F)-C(21F) | 109.3(4) |
| C(18F)-C(17F)-C(16F) | 111.3(3) |
| C(21F)-C(17F)-C(16F) | 115.3(4) |
| C(18F)-C(17F)-H(17F) | 106.8 |
| C(21F)-C(17F)-H(17F) | 106.8 |
| C(16F)-C(17F)-H(17F) | 106.8 |
| O(8F)-C(18F)-O(7F) | 122.9(4) |
| O(8F)-C(18F)-C(17F) | 120.4(4) |
| O(7F)-C(18F)-C(17F) | 116.0(4) |
| C(20F)-C(19F)-C(16F) | 115.3(4) |
| C(20F)-C(19F)-H(19C) | 108.5 |
| C(16F)-C(19F)-H(19C) | 108.5 |
| C(20F)-C(19F)-H(19D) | 108.5 |
| C(16F)-C(19F)-H(19D) | 108.5 |
| H(19C)-C(19F)-H(19D) | 107.5 |
| C(19F)-C(20F)-H(20D) | 109.5 |
| C(19F)-C(20F)-H(20E) | 109.5 |
| H(20D)-C(20F)-H(20E) | 109.5 |
| C(19F)-C(20F)-H(20F) | 109.5 |
| H(20D)-C(20F)-H(20F) | 109.5 |
| H(20E)-C(20F)-H(20F) | 109.5 |
| C(17F)-C(21F)-H(21D) | 109.5 |
| C(17F)-C(21F)-H(21E) | 109.5 |
| H(21D)-C(21F)-H(21E) | 109.5 |
| C(17F)-C(21F)-H(21F) | 109.5 |
| H(21D)-C(21F)-H(21F) | 109.5 |
| H(21E)-C(21F)-H(21F) | 109.5 |

Symmetry transformations used to generate equivalent atoms:

Table 4. Anisotropic displacement parameters (pm$^2$x 10$^{-1}$)for x3714fin. The anisotropic displacement factor exponent takes the form: $-2\pi^2$[ $h^2a^{*2}U^{11}$ + ... + 2 h k a* b* $U^{12}$ ]

| | $U^{11}$ | $U^{22}$ | $U^{33}$ | $U^{23}$ | $U^{13}$ | $U^{12}$ |
|---|---|---|---|---|---|---|
| S(1) | 46(1) | 52(1) | 79(1) | -12(1) | -14(1) | -6(1) |
| S(2) | 50(1) | 55(1) | 62(1) | -7(1) | -5(1) | -8(1) |
| O(1) | 44(2) | 75(3) | 123(4) | -24(3) | -24(2) | 3(2) |
| O(2) | 74(3) | 71(3) | 66(2) | -13(2) | -18(2) | -10(2) |
| O(5) | 52(2) | 76(3) | 88(3) | -13(2) | -8(2) | -1(2) |
| O(6) | 71(2) | 69(2) | 59(2) | -12(2) | -5(2) | -6(2) |
| O(3) | 96(3) | 80(3) | 90(3) | -20(2) | 22(2) | -38(2) |
| O(4) | 93(3) | 62(2) | 94(3) | -27(2) | 9(2) | -22(2) |
| N(1) | 57(3) | 48(3) | 75(3) | -17(2) | 0(2) | -13(2) |
| C(1) | 80(4) | 56(3) | 79(4) | -24(3) | 13(3) | -28(3) |
| C(2) | 64(3) | 70(3) | 65(3) | -19(3) | 10(3) | -32(3) |
| C(3) | 61(3) | 57(3) | 63(3) | -10(2) | -6(2) | -22(2) |
| C(4) | 60(3) | 75(3) | 58(3) | -16(2) | -3(2) | -28(3) |
| C(5) | 68(3) | 81(3) | 71(3) | -3(3) | -15(3) | -22(3) |
| C(6) | 121(6) | 152(8) | 141(7) | -3(6) | -58(5) | -57(5) |
| C(7) | 98(5) | 85(4) | 148(7) | 10(4) | -53(5) | -27(4) |
| C(8) | 46(3) | 44(3) | 64(3) | -4(2) | -8(2) | -7(2) |
| C(9) | 49(3) | 69(4) | 98(4) | -35(3) | -16(3) | 3(3) |
| C(10) | 50(3) | 74(4) | 112(5) | -17(4) | -22(3) | -9(3) |
| C(11) | 76(4) | 60(4) | 73(4) | 5(3) | -27(3) | -28(3) |
| C(12) | 87(4) | 54(3) | 87(4) | -19(3) | -21(3) | -9(3) |
| C(13) | 59(3) | 48(3) | 80(4) | -16(3) | -10(3) | -4(2) |
| C(14) | 109(6) | 84(5) | 113(5) | 8(4) | -55(5) | -43(4) |
| C(22) | 53(3) | 48(3) | 61(3) | -9(2) | -4(2) | -12(2) |
| C(23) | 58(4) | 75(4) | 106(5) | -23(4) | -4(3) | -17(3) |
| C(24) | 66(4) | 98(6) | 112(5) | -22(4) | -13(4) | -25(4) |
| C(25) | 87(5) | 74(5) | 88(4) | 5(3) | -27(4) | -34(4) |
| C(26) | 118(7) | 59(4) | 95(5) | -21(3) | -21(5) | -19(4) |
| C(27) | 65(4) | 67(4) | 82(4) | -12(3) | -9(3) | -7(3) |
| C(28) | 141(8) | 101(6) | 138(7) | -8(5) | -52(6) | -63(6) |
| O(7) | 94(5) | 63(4) | 107(5) | -9(3) | 21(4) | -25(4) |
| O(8) | 85(5) | 47(4) | 92(5) | -11(4) | 13(4) | -16(3) |
| N(2) | 40(5) | 51(3) | 55(4) | -11(3) | 16(3) | -7(3) |
| C(15) | 92(7) | 56(5) | 57(6) | -20(4) | -6(5) | -26(5) |

| | | | | | | |
|---|---|---|---|---|---|---|
| C(16) | 79(6) | 69(6) | 57(5) | -2(4) | 3(4) | -33(5) |
| C(17) | 66(5) | 66(5) | 81(6) | -4(4) | -18(5) | -1(4) |
| C(18) | 69(6) | 59(5) | 57(5) | -12(4) | 6(5) | -29(4) |
| C(19) | 93(7) | 92(7) | 62(5) | -1(5) | -15(5) | -49(6) |
| C(20) | 100(8) | 136(11) | 108(8) | -14(7) | -49(7) | -24(7) |
| C(21) | 74(6) | 89(6) | 54(6) | -3(5) | -8(5) | -25(5) |
| O(7F) | 79(7) | 75(6) | 80(7) | -10(5) | 42(7) | -37(6) |
| O(8F) | 112(10) | 81(8) | 92(8) | -37(7) | 30(8) | -28(7) |
| N(2F) | 40(5) | 51(3) | 55(4) | -11(3) | 16(3) | -7(3) |
| C(15F) | 64(9) | 89(11) | 64(9) | -11(8) | 12(7) | -16(8) |
| C(16F) | 60(6) | 91(8) | 67(6) | -10(6) | 12(6) | -36(6) |
| C(17F) | 59(6) | 84(7) | 56(6) | -8(6) | 6(5) | -30(6) |
| C(18F) | 66(7) | 71(8) | 56(6) | -22(6) | 18(6) | -27(6) |
| C(19F) | 101(11) | 146(13) | 89(10) | -8(10) | 3(10) | -41(10) |
| C(20F) | 147(18) | 220(20) | 141(18) | -52(18) | -28(15) | -55(17) |
| C(21F) | 62(10) | 99(12) | 101(13) | 1(10) | 2(9) | -23(9) |

Table 5. Hydrogen coordinates ( x $10^4$) and isotropic displacement parameters ($pm^2$x $10^{-1}$) for x3714fin.

| | x | y | z | U(eq) |
|---|---|---|---|---|
| H(1O3) | 5656 | 4024 | 4788 | 136 |
| H(1A) | 1905 | -728 | 6540 | 86 |
| H(1B) | 3664 | -902 | 6985 | 86 |
| H(2) | 3559 | 1080 | 5392 | 79 |
| H(3) | 3224 | 1984 | 7837 | 72 |
| H(5A) | 603 | 2160 | 6836 | 88 |
| H(5B) | 1569 | 2956 | 5498 | 88 |
| H(6A) | -107 | 610 | 5690 | 200 |
| H(6B) | 932 | 1336 | 4347 | 200 |
| H(6C) | -683 | 2126 | 5011 | 200 |
| H(7A) | 6114 | 646 | 6573 | 164 |
| H(7B) | 5215 | 198 | 8058 | 164 |
| H(7C) | 6164 | 1539 | 7652 | 164 |
| H(9) | -1755 | -1494 | 9484 | 83 |
| H(10) | -3633 | -2730 | 8943 | 93 |
| H(12) | 94 | -5407 | 7705 | 89 |
| H(13) | 1976 | -4220 | 8323 | 75 |
| H(14A) | -3776 | -5515 | 8623 | 147 |
| H(14B) | -2560 | -5626 | 7275 | 147 |
| H(14C) | -3922 | -4365 | 7332 | 147 |
| H(23) | 11608 | 11558 | 419 | 95 |
| H(24) | 13463 | 12704 | 1073 | 108 |
| H(26) | 9773 | 15290 | 2468 | 106 |
| H(27) | 7871 | 14113 | 1844 | 87 |
| H(28A) | 14151 | 14719 | 1712 | 180 |
| H(28B) | 13140 | 14524 | 3178 | 180 |
| H(28C) | 12727 | 15847 | 2106 | 180 |
| H(1O7) | 3749 | 5973 | 5487 | 138 |
| H(15A) | 6542 | 10741 | 3022 | 79 |
| H(15B) | 8373 | 10435 | 3331 | 79 |
| H(16) | 7901 | 8077 | 2881 | 83 |
| H(17) | 4780 | 8998 | 4166 | 87 |
| H(19A) | 7466 | 8771 | 5434 | 97 |

| | | | | |
|---|---|---|---|---|
| H(19B) | 7439 | 7239 | 5272 | 97 |
| H(20A) | 10168 | 7107 | 4337 | 167 |
| H(20B) | 10049 | 7748 | 5624 | 167 |
| H(20C) | 10231 | 8708 | 4193 | 167 |
| H(21A) | 5468 | 8236 | 1634 | 109 |
| H(21B) | 4838 | 9731 | 1903 | 109 |
| H(21C) | 3664 | 8533 | 2403 | 109 |
| H(1OF) | 4609 | 5933 | 5368 | 125 |
| H(15C) | 6340 | 11219 | 3134 | 90 |
| H(15D) | 7953 | 10352 | 3585 | 90 |
| H(16F) | 5677 | 9141 | 4542 | 89 |
| H(17F) | 7691 | 7727 | 2663 | 80 |
| H(19C) | 3930 | 8398 | 3436 | 137 |
| H(19D) | 3906 | 10011 | 2995 | 137 |
| H(20D) | 5491 | 9789 | 947 | 243 |
| H(20E) | 3931 | 8957 | 1167 | 243 |
| H(20F) | 5671 | 8169 | 1390 | 243 |
| H(21D) | 8361 | 8027 | 5139 | 136 |
| H(21E) | 9573 | 8152 | 3759 | 136 |
| H(21F) | 9106 | 6689 | 4571 | 136 |
| H(1N2) | 9250(30) | 9740(40) | 1180(40) | 70(20) |
| H(1NF) | 8640(70) | 9660(30) | 1420(50) | 70(20) |
| H(1N1) | 790(30) | 270(30) | 8570(30) | 74(17) |

Table 6. Hydrogen bonds for x3714fin [pm and °].

| D-H...A | d(D-H) | d(H...A) | d(D...A) | <(DHA) |
|---|---|---|---|---|
| O(3)-H(1O3)...O(8) | 82 | 195 | 276.5(7) | 170.3 |
| O(3)-H(1O3)...O(8F) | 82 | 176 | 254.2(9) | 159.9 |
| O(7)-H(1O7)...O(4) | 82 | 177 | 255.3(6) | 159.4 |
| O(7F)-H(1OF)...O(4) | 82 | 196 | 274.7(7) | 161.1 |
| N(1)-H(1N1)...O(6)#1 | 97.97(15) | 200.8(9) | 297.6(6) | 170(4) |
| N(2)-H(1N2)...O(2)#2 | 97.99(15) | 180.4(10) | 277.7(8) | 171(4) |
| N(2F)-H(1NF)...O(2)#2 | 98.00(15) | 218(2) | 312.9(14) | 161(5) |

Symmetry transformations used to generate equivalent atoms:

#1 x-1,y-1,z+1 #2 x+1,y+1,z-1

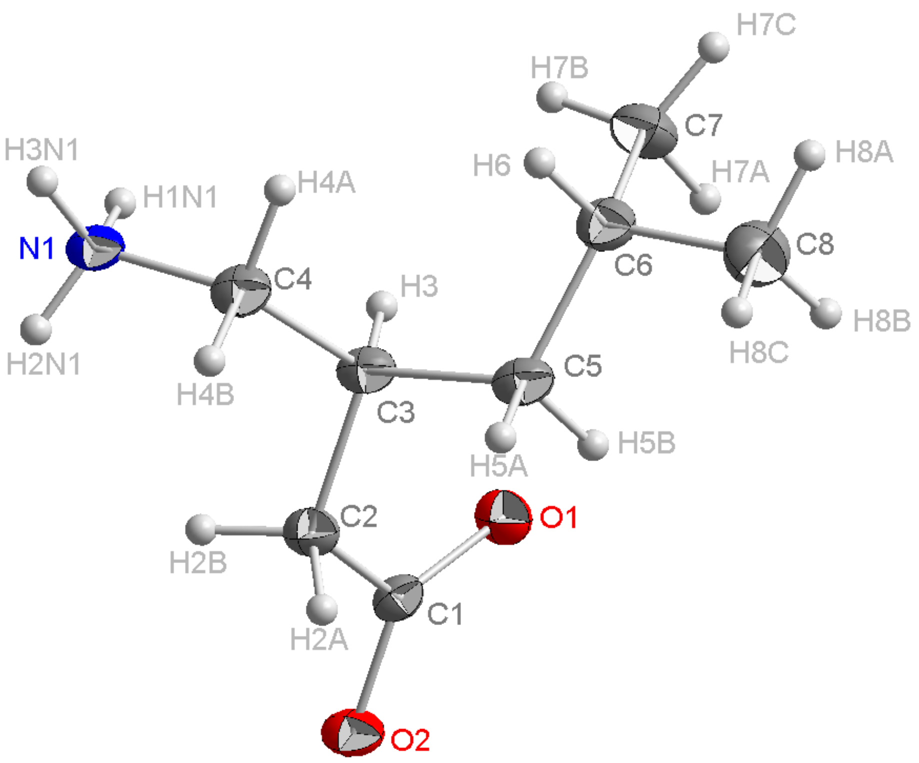


**Supplementary Figure 15:** crystal structure of (+) *S*-pregabalin **Cry2**.

| | | |
|---|---|---|
| Identification code | x3707fin | |
| Empirical formula | C8 H17 N O2 | |
| Formula weight | 159.22 | |
| Temperature | 130(2) K | |
| Wavelength | 154.184 pm | |
| Crystal system | Orthorhombic | |
| Space group | P 21 21 21 | |
| Unit cell dimensions | a = 634.460(10) pm | $\alpha$= 90°. |
| | b = 781.250(10) pm | $\beta$= 90°. |
| | c = 1849.78(4) pm | $\gamma$ = 90°. |
| Volume | 0.91688(3) $nm^3$ | |
| Z | 4 | |
| Density (calculated) | 1.153 $Mg/m^3$ | |
| Absorption coefficient | 0.659 $mm^{-1}$ | |
| F(000) | 352 | |
| Crystal size | 0.28 x 0.07 x 0.04 $mm^3$ | |
| Theta range for data collection | 4.781 to 67.694°. | |
| Index ranges | -7<=h<=7, -9<=k<=9, -22<=l<=20 | |
| Reflections collected | 9380 | |

| | |
|---|---|
| Independent reflections | 1654 [R(int) = 0.0363] |
| Completeness to theta = 67.684° | 99.9 % |
| Absorption correction | Semi-empirical from equivalents |
| Max. and min. transmission | 1.00000 and 0.57343 |
| Refinement method | Full-matrix least-squares on $F^2$ |
| Data / restraints / parameters | 1654 / 0 / 168 |
| Goodness-of-fit on $F^2$ | 1.103 |
| Final R indices [I>2sigma(I)] | R1 = 0.0331, wR2 = 0.0857 |
| R indices (all data) | R1 = 0.0346, wR2 = 0.0870 |
| Absolute structure parameter | 0.03(10) |
| Largest diff. peak and hole | 0.286 and -0.130 e.Å$^{-3}$ |

**Comments:** Structure solution with SHELXT-2018 (dual-space method). Anisotropic refinement of all non-hydrogen atoms with SHELXL-2018. All H atoms were located on difference Fourier maps calculated at the final stage of the structure refinement. C3 is chiral and s-configured. The results are comparable with the published structure (Vera Vasylyeva et al., Cryst. Eng. Comm., 2022, **24**, 8390–8398).[15,16]

Table 2. Atomic coordinates ( x $10^4$) and equivalent isotropic displacement parameters ($pm^2$x $10^{-1}$) for x3707fin. U(eq) is defined as one third of the trace of the orthogonalized $U^{ij}$ tensor.

| | x | y | z | U(eq) |
|---|---|---|---|---|
| O(1) | 3135(2) | 352(2) | 5783(1) | 24(1) |
| O(2) | 359(2) | -529(2) | 5150(1) | 25(1) |
| N(1) | 1141(3) | 6033(2) | 5131(1) | 21(1) |
| C(1) | 1352(3) | 581(2) | 5515(1) | 19(1) |
| C(2) | 274(3) | 2314(2) | 5597(1) | 22(1) |
| C(3) | 1474(3) | 3648(2) | 6037(1) | 21(1) |
| C(4) | 676(3) | 5460(3) | 5881(1) | 23(1) |
| C(5) | 1335(3) | 3276(3) | 6848(1) | 23(1) |
| C(6) | 2807(3) | 4319(3) | 7335(1) | 25(1) |
| C(7) | 5119(4) | 4057(3) | 7140(1) | 33(1) |
| C(8) | 2445(4) | 3856(3) | 8125(1) | 36(1) |

Table 3. Bond lengths [pm] and angles [°] for x3707fin.

| | |
|---|---|
| O(1)-C(1) | 124.8(2) |
| O(2)-C(1) | 126.7(2) |
| N(1)-C(4) | 148.8(3) |
| N(1)-H(1N1) | 89(3) |
| N(1)-H(2N1) | 87(3) |
| N(1)-H(3N1) | 88(3) |
| C(1)-C(2) | 152.4(3) |
| C(2)-C(3) | 152.7(3) |
| C(2)-H(2A) | 94(3) |
| C(2)-H(2B) | 97(3) |
| C(3)-C(5) | 152.9(3) |
| C(3)-C(4) | 153.1(3) |
| C(3)-H(3) | 96(2) |
| C(4)-H(4A) | 98(3) |
| C(4)-H(4B) | 96(2) |
| C(5)-C(6) | 153.3(3) |
| C(5)-H(5A) | 100(3) |
| C(5)-H(5B) | 97(2) |
| C(6)-C(8) | 152.2(3) |
| C(6)-C(7) | 152.5(3) |
| C(6)-H(6) | 95(3) |
| C(7)-H(7A) | 100(3) |
| C(7)-H(7B) | 96(3) |
| C(7)-H(7C) | 94(3) |
| C(8)-H(8A) | 97(3) |
| C(8)-H(8B) | 93(3) |
| C(8)-H(8C) | 97(3) |

| | |
|---|---|
| C(4)-N(1)-H(1N1) | 110.6(16) |
| C(4)-N(1)-H(2N1) | 111.6(18) |
| H(1N1)-N(1)-H(2N1) | 109(2) |
| C(4)-N(1)-H(3N1) | 107.3(16) |
| H(1N1)-N(1)-H(3N1) | 110(3) |
| H(2N1)-N(1)-H(3N1) | 108(3) |
| O(1)-C(1)-O(2) | 124.33(18) |
| O(1)-C(1)-C(2) | 119.69(17) |
| O(2)-C(1)-C(2) | 115.93(16) |
| C(1)-C(2)-C(3) | 115.81(16) |
| C(1)-C(2)-H(2A) | 105.6(15) |
| C(3)-C(2)-H(2A) | 111.5(15) |
| C(1)-C(2)-H(2B) | 103.4(14) |
| C(3)-C(2)-H(2B) | 111.6(14) |
| H(2A)-C(2)-H(2B) | 108(2) |
| C(2)-C(3)-C(5) | 111.38(16) |
| C(2)-C(3)-C(4) | 111.45(16) |
| C(5)-C(3)-C(4) | 110.01(16) |
| C(2)-C(3)-H(3) | 106.2(14) |
| C(5)-C(3)-H(3) | 108.7(14) |
| C(4)-C(3)-H(3) | 108.9(15) |
| N(1)-C(4)-C(3) | 112.88(16) |
| N(1)-C(4)-H(4A) | 104.4(15) |
| C(3)-C(4)-H(4A) | 110.9(15) |
| N(1)-C(4)-H(4B) | 107.0(14) |
| C(3)-C(4)-H(4B) | 110.1(16) |
| H(4A)-C(4)-H(4B) | 111(2) |
| C(3)-C(5)-C(6) | 116.15(17) |
| C(3)-C(5)-H(5A) | 106.9(14) |
| C(6)-C(5)-H(5A) | 109.1(14) |
| C(3)-C(5)-H(5B) | 106.4(13) |
| C(6)-C(5)-H(5B) | 108.4(13) |
| H(5A)-C(5)-H(5B) | 110(2) |
| C(8)-C(6)-C(7) | 109.91(19) |
| C(8)-C(6)-C(5) | 110.26(18) |
| C(7)-C(6)-C(5) | 112.00(18) |
| C(8)-C(6)-H(6) | 106.4(14) |
| C(7)-C(6)-H(6) | 109.1(14) |
| C(5)-C(6)-H(6) | 108.9(14) |
| C(6)-C(7)-H(7A) | 110.0(18) |
| C(6)-C(7)-H(7B) | 114.0(17) |
| H(7A)-C(7)-H(7B) | 105(2) |
| C(6)-C(7)-H(7C) | 110.1(17) |
| H(7A)-C(7)-H(7C) | 104(3) |
| H(7B)-C(7)-H(7C) | 113(2) |
| C(6)-C(8)-H(8A) | 111.7(19) |

| | | | |
|---|---|---|---|
| C(6)-C(8)-H(8B) | 108.3(19) | H(8A)-C(8)-H(8C) | 108(3) |
| H(8A)-C(8)-H(8B) | 108(3) | H(8B)-C(8)-H(8C) | 108(3) |
| C(6)-C(8)-H(8C) | 112.4(19) | | |

Symmetry transformations used to generate equivalent atoms:

Table 4. Anisotropic displacement parameters ($pm^2 x 10^{-1}$)for x3707fin. The anisotropic displacement factor exponent takes the form: $-2\pi^2[ h^2a^{*2}U^{11} + ... + 2 h k a^* b^* U^{12} ]$

| | $U^{11}$ | $U^{22}$ | $U^{33}$ | $U^{23}$ | $U^{13}$ | $U^{12}$ |
|---|---|---|---|---|---|---|
| O(1) | 18(1) | 22(1) | 31(1) | -1(1) | -2(1) | 4(1) |
| O(2) | 22(1) | 15(1) | 36(1) | -3(1) | -2(1) | 1(1) |
| N(1) | 18(1) | 14(1) | 33(1) | -1(1) | -1(1) | 1(1) |
| C(1) | 19(1) | 17(1) | 21(1) | 2(1) | 4(1) | -1(1) |
| C(2) | 18(1) | 19(1) | 30(1) | -1(1) | -2(1) | 1(1) |
| C(3) | 17(1) | 16(1) | 30(1) | -2(1) | 2(1) | 1(1) |
| C(4) | 22(1) | 18(1) | 30(1) | -3(1) | 2(1) | 2(1) |
| C(5) | 19(1) | 20(1) | 31(1) | -1(1) | 3(1) | -2(1) |
| C(6) | 26(1) | 19(1) | 30(1) | -2(1) | -1(1) | 0(1) |
| C(7) | 24(1) | 36(1) | 39(1) | 0(1) | -4(1) | -6(1) |
| C(8) | 43(2) | 33(1) | 31(1) | -3(1) | 0(1) | -2(1) |

Table 5. Hydrogen coordinates ( x $10^4$) and isotropic displacement parameters ($pm^2$x $10^{-1}$) for x3707fin.

| | x | y | z | U(eq) |
|---|---|---|---|---|
| H(1N1) | 2460(40) | 5760(30) | 5009(13) | 24(6) |
| H(2N1) | 280(50) | 5580(40) | 4821(15) | 33(7) |
| H(3N1) | 970(40) | 7140(40) | 5119(13) | 23(6) |
| H(2A) | -1060(40) | 2080(30) | 5801(13) | 19(5) |
| H(2B) | 70(40) | 2690(30) | 5099(14) | 21(6) |
| H(3) | 2920(40) | 3570(30) | 5890(12) | 19(5) |
| H(4A) | 1390(40) | 6300(30) | 6188(14) | 26(6) |
| H(4B) | -820(40) | 5510(30) | 5941(12) | 19(5) |
| H(5A) | -150(40) | 3490(30) | 6995(13) | 26(6) |
| H(5B) | 1670(40) | 2070(30) | 6907(11) | 15(5) |
| H(6) | 2470(40) | 5490(40) | 7290(12) | 22(6) |
| H(7A) | 5460(50) | 2810(40) | 7140(15) | 38(7) |
| H(7B) | 5490(40) | 4460(40) | 6667(16) | 36(7) |
| H(7C) | 5990(40) | 4510(40) | 7506(16) | 38(7) |
| H(8A) | 3330(50) | 4530(40) | 8446(17) | 47(8) |
| H(8B) | 2790(50) | 2700(40) | 8188(16) | 38(7) |
| H(8C) | 980(50) | 4020(40) | 8272(17) | 46(8) |

Table 6. Hydrogen bonds for x3707fin [pm and °].

| D-H...A | d(D-H) | d(H...A) | d(D...A) | <(DHA) |
|---|---|---|---|---|
| N(1)-H(1N1)...O(2)#1 | 89(3) | 187(3) | 275.4(2) | 170(2) |
| N(1)-H(2N1)...O(1)#2 | 87(3) | 190(3) | 276.8(2) | 174(3) |
| N(1)-H(3N1)...O(2)#3 | 88(3) | 186(3) | 273.2(2) | 174(3) |
| C(2)-H(2B)...O(1)#2 | 97(3) | 255(3) | 341.8(3) | 148.0(19) |

Symmetry transformations used to generate equivalent atoms:

#1 x+1/2,-y+1/2,-z+1 #2 x-1/2,-y+1/2,-z+1 #3 x,y+1,z

# Computational details

All calculations were performed using the release version of ORCA 5.0.4[17] in combination with XTB v. 6.7.0.[18,19] Considering the complexity of the transformation and the enormous size of the structures that are required to be evaluated, we limited our computational analysis to the stereo-determining step that follows the formation of the 1-azaallyl cation while also employing an ONIOM-approach (PBE-D3BJ/def2-SVP: GFN2-xTB) as the calculation method. Based on computational investigations on related systems by List et al.[4,20,21], the PBE functional[22] in conjunction with the D3 version of Grimme's dispersion correction with Becke-Johnson damping function[23,24] was used. For geometry optimization, frequencies and TS-search, the def2-SVP basis set was used for all atoms.[25] The reactants were included in the high-level region, and the catalyst was treated at the lower-level. A manual conformational search has been performed on possible catalyst substrate orientations that were subsequently optimized at the ONIOM(PBE-D3BJ/def2-SVP: GFN2-xTB, gas phase) level of theory. Transition states were located by employing the Climbing Image Nudged Elastic Band (NEB-CI)[26] method starting from relaxed ground state structures of the reactants followed by TS-optimization and frequency analysis at the same level of theory. Ground states and transition states were verified by numerical frequency calculations (at 253 K) showing zero or exactly one negative frequency, respectively. Finally, the electronic part of the Gibbs free energy was further refined using a perturbatively corrected double hybrid DFT method for the higher quantum mechanical region in combination with the ALPB solvation model for the complete system and *n*-hexane as replacement solvent for *n*-pentane, which is not parametrized in the ALPB solvation model, (referred to as ONIOM(B2PLYP-D3BJ/def2-TZVP(ALPB:*n*-hexane):GFN2-xTB)).[27,28]

Images of molecular structures were generated using the CYLview20 software.[29]

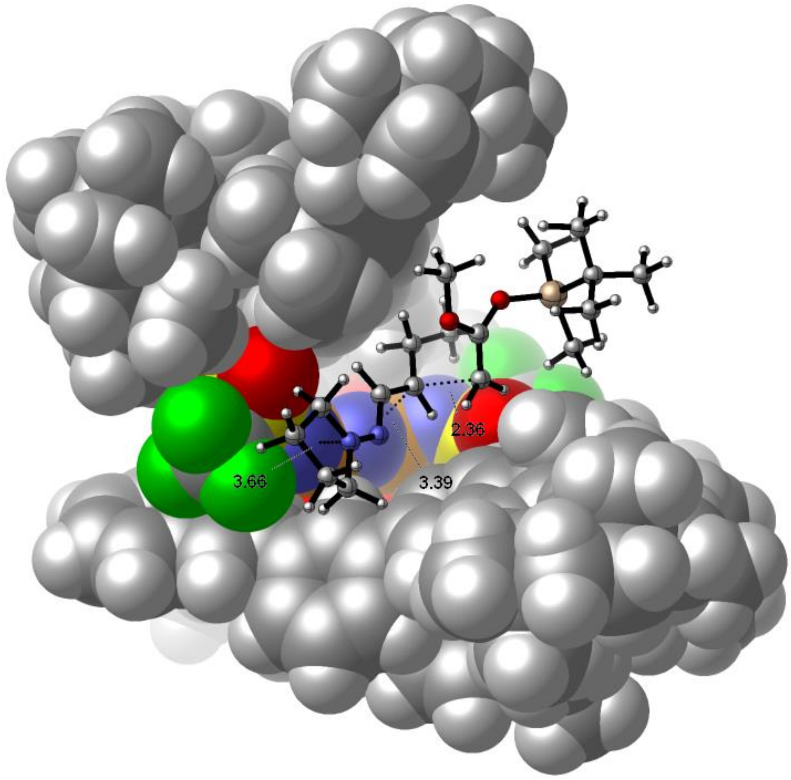


**Supplementary Figure 16:** Modelled transition state for the *re*-face attack of silyl ketene acetal **2a** on hydrazone **1a** catalyzed by IDPi **3h**. **ΔΔGrel = 0.0 [kcal/mol ]**

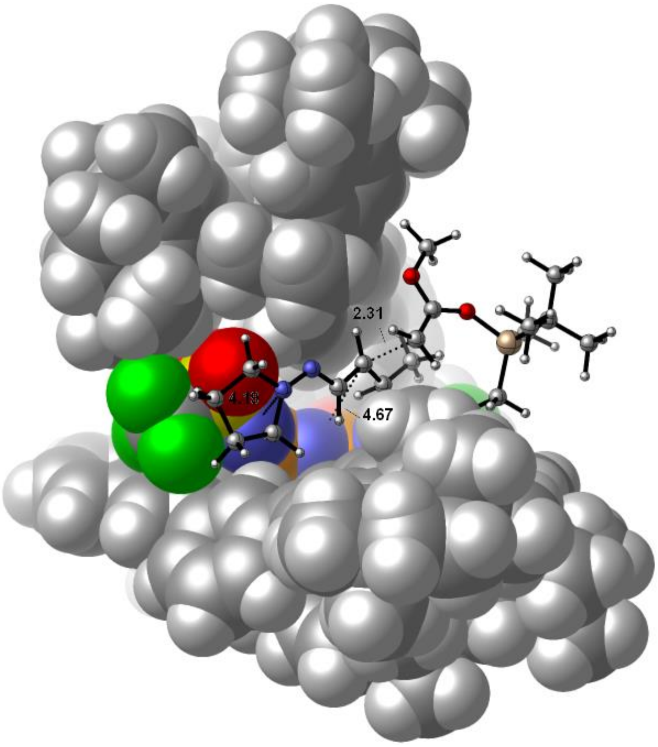


**Supplementary Figure 17:** Modelled transition state for the *si*-face attack of silyl ketene acetal **2a** on hydrazone **1a** catalyzed by IDPi **3h**. **ΔΔGrel = 1.9 [kcal/mol ]**

Cartesian Coordinates and Energies

(all energies are reported in Hartree unless stated otherwise)

SM-Re

ONIOM(PBE-D3BJ/def2-SVP: GFN2-xTB, gas phase) G = -1707.276608

ONIOM(B2PLYP-D3BJ/def2-TZVP(ALPB:n-hexane):GFN2-xTB) G = -1708.921619

Number of imaginary frequencies: 0

Coordinates:

| | | | |
|---|---|---|---|
| C | -2.211366 | 1.355987 | -4.739920 |
| C | -2.006816 | 2.682277 | -4.475918 |
| O | -1.102962 | 3.356856 | -5.225334 |
| C | -1.027069 | 4.774795 | -5.099998 |
| O | -2.581129 | 3.405071 | -3.513677 |
| Si | -4.115755 | 3.431776 | -2.681540 |
| C | -5.024202 | 4.930223 | -3.499938 |
| C | -5.147306 | 4.696289 | -5.018225 |

| | | | |
|---|---|---|---|
| C | -6.432600 | 5.063251 | -2.883195 |
| C | -4.226011 | 6.221097 | -3.230645 |
| C | -5.079664 | 1.838099 | -2.889469 |
| C | -3.686309 | 3.767394 | -0.897157 |
| H | -2.919570 | 0.779457 | -4.141196 |
| H | -1.713572 | 0.902440 | -5.604843 |
| H | -0.856159 | 5.081210 | -4.049961 |
| H | -1.953780 | 5.261252 | -5.473952 |
| H | -0.165222 | 5.083395 | -5.718782 |
| H | -5.709833 | 3.768801 | -5.248821 |
| H | -4.153583 | 4.610260 | -5.501895 |
| H | -5.683301 | 5.543230 | -5.500999 |
| H | -6.392428 | 5.197444 | -1.782488 |
| H | -6.955083 | 5.948850 | -3.308104 |
| H | -7.062413 | 4.174705 | -3.090866 |
| H | -4.146000 | 6.434180 | -2.145285 |
| H | -4.724692 | 7.092521 | -3.710140 |
| H | -3.195604 | 6.155794 | -3.632086 |
| H | -5.118386 | 1.467549 | -3.931308 |
| H | -4.648800 | 1.064409 | -2.217124 |
| H | -6.119726 | 2.004603 | -2.542034 |
| H | -2.923391 | 4.562681 | -0.792878 |
| H | -3.294827 | 2.842336 | -0.427875 |
| H | -4.585830 | 4.060155 | -0.320225 |
| H | 4.115052 | -2.683623 | -2.832433 |
| H | 5.049522 | -3.721330 | -0.081731 |
| H | 5.937552 | -3.817307 | -1.634127 |
| H | 4.350410 | -5.520846 | -2.510444 |
| C | 0.096569 | -1.116811 | -2.111199 |
| C | -0.206340 | 0.282740 | -2.413339 |
| C | -1.172162 | 0.932940 | -1.409761 |

| | | | |
|---|---|---|---|
| C | 1.373695 | -1.634271 | -2.005425 |
| N | 1.490337 | -2.949183 | -1.689563 |
| N | 2.644050 | -3.473392 | -1.558898 |
| C | 3.973144 | -2.843367 | -1.739753 |
| C | 4.935453 | -3.888898 | -1.171978 |
| C | 4.223599 | -5.224836 | -1.448157 |
| C | 2.761047 | -4.890767 | -1.159940 |
| H | 4.592187 | -6.053540 | -0.815462 |
| H | 2.519928 | -4.954444 | -0.076909 |
| H | 2.004013 | -5.475196 | -1.715033 |
| H | -0.741392 | -1.817159 | -1.934436 |
| H | 0.723490 | 0.879233 | -2.496363 |
| H | -0.728055 | 0.353446 | -3.419759 |
| H | -0.788015 | 0.851164 | -0.373882 |
| H | -1.280900 | 2.003342 | -1.664876 |
| H | -2.180334 | 0.470076 | -1.416392 |
| H | 2.264323 | -0.982736 | -2.081919 |
| H | 3.999869 | -1.844064 | -1.247383 |
| N | -0.243454 | 0.031950 | 1.739182 |
| P | -1.534348 | 0.656230 | 2.440694 |
| N | -2.673424 | 1.057708 | 1.391581 |
| S | -3.827595 | 0.076331 | 1.021650 |
| O | -4.144107 | 0.087744 | -0.380086 |
| O | -3.849886 | -1.198827 | 1.666363 |
| C | -5.265857 | 1.007192 | 1.819915 |
| F | -6.476339 | 0.666078 | 1.372180 |
| F | -5.158022 | 2.328239 | 1.649838 |
| F | -5.251632 | 0.763038 | 3.135392 |
| O | -1.136607 | 2.000121 | 3.221029 |
| O | -2.155872 | -0.321480 | 3.546763 |
| F | 5.921298 | -0.197619 | 1.441875 |

| | | | |
|---|---|---|---|
| N | 2.159270 | -0.972948 | 1.302226 |
| P | 1.078610 | -0.531584 | 2.412989 |
| S | 3.266361 | 0.040983 | 0.893593 |
| O | 1.793891 | 0.492024 | 3.420261 |
| O | 0.769171 | -1.839799 | 3.292083 |
| O | 3.162010 | 1.394944 | 1.317209 |
| O | 3.694522 | -0.188382 | -0.466080 |
| C | 4.736567 | -0.640385 | 1.867413 |
| F | 4.639142 | -0.327312 | 3.159617 |
| F | 4.773142 | -1.979370 | 1.764384 |
| H | -3.032986 | -2.615270 | 3.734981 |
| C | -2.277466 | 2.700322 | 3.643392 |
| C | -2.991273 | 2.168622 | 4.694121 |
| C | -4.269339 | 2.697861 | 5.032606 |
| C | -5.123120 | 2.095483 | 5.980315 |
| C | -6.367139 | 2.608020 | 6.223814 |
| C | -6.820885 | 3.743234 | 5.535745 |
| C | -6.025325 | 4.335187 | 4.596445 |
| C | -4.740058 | 3.825097 | 4.315400 |
| C | -3.908591 | 4.415120 | 3.348969 |
| C | -2.683025 | 3.892807 | 2.996487 |
| C | -1.833389 | 4.672459 | 2.078932 |
| C | -1.869345 | 6.062330 | 2.222622 |
| C | -1.090189 | 6.875688 | 1.425119 |
| C | -0.257612 | 6.317526 | 0.476616 |
| C | -0.212253 | 4.935331 | 0.299693 |
| C | -1.004305 | 4.119657 | 1.109191 |
| H | -1.006086 | 3.050700 | 0.961976 |
| H | 0.177152 | 7.526612 | -6.976952 |
| H | -9.034916 | -2.536047 | 1.504812 |
| H | 0.242633 | -2.859411 | 6.451918 |

| | | | |
|---|---|---|---|
| H | 0.384838 | 6.956681 | -0.108851 |
| H | -1.118217 | 7.948305 | 1.557036 |
| H | -0.663266 | -6.179204 | 3.985276 |
| H | 0.723974 | -5.144147 | 5.674598 |
| H | -2.482307 | 6.507515 | 2.991590 |
| H | -4.276049 | 5.290981 | 2.836381 |
| H | -6.370357 | 5.196484 | 4.042979 |
| H | -7.806138 | 4.133597 | 5.744076 |
| H | -7.015091 | 2.130866 | 6.943928 |
| H | -4.796694 | 1.206659 | 6.496536 |
| C | -2.434760 | 1.056953 | 5.493581 |
| C | -2.086508 | -0.144955 | 4.916784 |
| C | -1.712219 | -1.264593 | 5.702736 |
| C | -1.628246 | -1.100127 | 7.065656 |
| C | -1.864903 | 0.135968 | 7.689207 |
| C | -1.716606 | 0.296302 | 9.082956 |
| C | -1.936117 | 1.507724 | 9.674236 |
| C | -2.315199 | 2.608374 | 8.891988 |
| C | -2.488729 | 2.477518 | 7.541557 |
| C | -2.278200 | 1.237039 | 6.899590 |
| H | -2.787710 | 3.333183 | 6.955830 |
| H | -2.472950 | 3.566621 | 9.364684 |
| H | -1.817379 | 1.627562 | 10.741008 |
| H | -1.416712 | -0.559887 | 9.669034 |
| H | -1.380370 | -1.946046 | 7.689471 |
| C | -1.431201 | -2.595740 | 5.137403 |
| C | -0.374148 | -3.313641 | 5.693219 |
| C | -0.107564 | -4.593090 | 5.257398 |
| C | -0.904805 | -5.176949 | 4.294618 |
| C | -1.980193 | -4.493482 | 3.721871 |
| C | -2.217101 | -3.183914 | 4.151053 |

| | | | |
|---|---|---|---|
| C | 3.393474 | 5.783016 | -6.572251 |
| C | 4.481319 | 2.491051 | -3.687820 |
| C | 3.053429 | 2.931956 | -3.990615 |
| C | 2.303784 | 1.821872 | -4.743671 |
| C | 2.293082 | 3.318556 | -2.740977 |
| C | 2.106091 | 2.615345 | -1.569619 |
| C | 1.291618 | 3.148185 | -0.579341 |
| C | 0.660206 | 4.379472 | -0.744988 |
| C | 0.891223 | 5.108728 | -1.913336 |
| C | 1.714521 | 4.586726 | -2.891836 |
| C | 2.167698 | 5.150181 | -4.154017 |
| C | 2.986753 | 4.211949 | -4.790292 |
| C | 3.586707 | 4.530224 | -5.982749 |
| C | 4.116162 | 6.067250 | -7.876957 |
| C | 3.448675 | 5.255416 | -8.996486 |
| C | 5.595044 | 5.660625 | -7.782677 |
| C | 4.076222 | 7.563792 | -8.206258 |
| C | 2.692409 | 8.155534 | -8.007040 |
| C | 2.235311 | 8.072206 | -6.546270 |
| C | 2.949259 | 9.137582 | -5.702296 |
| C | 0.724250 | 8.351503 | -6.524767 |
| C | 2.538824 | 6.704305 | -5.959505 |
| C | 1.946936 | 6.380161 | -4.739298 |
| H | 4.467627 | 1.647439 | -3.003192 |
| H | 4.991056 | 2.194933 | -4.603393 |
| H | 5.032498 | 3.302113 | -3.220736 |
| H | 2.333313 | 0.895277 | -4.174870 |
| H | 1.266123 | 2.105436 | -4.909345 |
| H | 2.772239 | 1.649382 | -5.710799 |
| H | 2.603742 | 1.674934 | -1.387889 |
| H | 1.170106 | 2.601100 | 0.342846 |

| | | | |
|---|---|---|---|
| H | 1.311652 | 7.095705 | -4.240926 |
| H | 0.420640 | 6.070308 | -2.060321 |
| H | 4.221777 | 3.804976 | -6.467569 |
| H | 3.955424 | 5.430354 | -9.943603 |
| H | 3.492364 | 4.192277 | -8.772772 |
| H | 2.402413 | 5.528999 | -9.106193 |
| H | 5.720972 | 4.582119 | -7.765745 |
| H | 6.134868 | 6.043848 | -8.646618 |
| H | 6.046111 | 6.071814 | -6.882567 |
| H | 4.394814 | 7.712151 | -9.240798 |
| H | 4.789583 | 8.086428 | -7.566362 |
| H | 1.976687 | 7.621885 | -8.635108 |
| H | 2.689547 | 9.200944 | -8.324682 |
| H | 2.734582 | 10.132371 | -6.087984 |
| H | 2.618691 | 9.087904 | -4.667994 |
| H | 4.025420 | 8.985196 | -5.715387 |
| H | 0.513542 | 9.256260 | -7.091862 |
| H | 0.351004 | 8.497354 | -5.515572 |
| C | -7.980610 | -4.443378 | -1.334176 |
| C | -4.582797 | -7.440799 | -1.486218 |
| C | -5.350636 | -7.030424 | -0.223779 |
| C | -6.202072 | -8.193579 | 0.290248 |
| C | -4.397899 | -6.519342 | 0.830352 |
| C | -3.477005 | -7.219185 | 1.574869 |
| C | -2.730318 | -6.545584 | 2.525985 |
| C | -2.834714 | -5.163541 | 2.720232 |
| C | -3.778327 | -4.462478 | 1.960791 |
| C | -4.568569 | -5.145436 | 1.051410 |
| C | -5.699437 | -4.701879 | 0.246293 |
| C | -6.184901 | -5.797590 | -0.478962 |
| C | -7.295809 | -5.660007 | -1.272159 |

| | | | |
|---|---|---|---|
| C | -9.200425 | -4.367836 | -2.234788 |
| C | -10.347887 | -5.156621 | -1.587855 |
| C | -8.903859 | -4.975027 | -3.615366 |
| C | -9.604578 | -2.906960 | -2.455712 |
| C | -9.661795 | -2.137232 | -1.148846 |
| C | -8.287183 | -2.048634 | -0.471076 |
| C | -7.466782 | -0.929141 | -1.125875 |
| C | -8.530162 | -1.709486 | 1.007096 |
| C | -7.535196 | -3.365396 | -0.562946 |
| C | -6.366616 | -3.495038 | 0.192437 |
| H | -3.935734 | -8.289988 | -1.275847 |
| H | -5.275034 | -7.718267 | -2.278391 |
| H | -3.969489 | -6.613635 | -1.836357 |
| H | -5.574031 | -9.047855 | 0.532959 |
| H | -6.742735 | -7.893833 | 1.185358 |
| H | -6.922677 | -8.498457 | -0.465288 |
| H | -3.346957 | -8.284874 | 1.445757 |
| H | -2.064013 | -7.133830 | 3.135621 |
| H | -5.978597 | -2.658031 | 0.751980 |
| H | -3.901934 | -3.396902 | 2.063056 |
| H | -7.653289 | -6.506544 | -1.838393 |
| H | -11.242772 | -5.099080 | -2.204912 |
| H | -10.072374 | -6.202714 | -1.476430 |
| H | -10.580072 | -4.768374 | -0.599881 |
| H | -8.807284 | -6.056145 | -3.572497 |
| H | -9.719240 | -4.742768 | -4.298295 |
| H | -7.983209 | -4.563984 | -4.022229 |
| H | -10.578227 | -2.871838 | -2.950950 |
| H | -8.876839 | -2.438961 | -3.121474 |
| H | -10.367602 | -2.625613 | -0.475207 |
| H | -10.033219 | -1.126181 | -1.330225 |

| | | | |
|---|---|---|---|
| H | -7.934241 | 0.033478 | -0.931053 |
| H | -6.454527 | -0.892808 | -0.732849 |
| H | -7.410503 | -1.073746 | -2.202340 |
| H | -9.155156 | -0.822275 | 1.083192 |
| H | -7.602379 | -1.510241 | 1.530216 |
| H | 7.292943 | 1.742051 | 0.991795 |
| H | 8.808773 | 1.051993 | 0.428223 |
| H | 6.666482 | 0.766682 | -2.587853 |
| H | 5.864383 | 0.808835 | -1.019747 |
| H | 7.351278 | -0.111286 | -1.217793 |
| H | 9.304680 | 1.034181 | -2.138419 |
| H | 9.717192 | 2.620202 | -1.502876 |
| H | 7.852887 | 2.148513 | -3.860560 |
| H | 9.555313 | 2.592075 | -4.001655 |
| H | 6.795918 | 4.175389 | -4.818071 |
| H | 8.463231 | 4.336954 | -5.383163 |
| H | 7.648873 | 5.706356 | -4.642137 |
| H | 9.894824 | 4.722780 | -1.884283 |
| H | 9.242235 | 6.058294 | -2.818280 |
| H | 10.297403 | 4.877742 | -3.598395 |
| H | 6.855547 | 6.358583 | -2.749945 |
| H | 3.394890 | 3.511333 | 1.608644 |
| H | 5.456666 | 2.689542 | 0.234460 |
| H | 1.890590 | 7.353531 | 2.805347 |
| H | 3.089510 | 8.420664 | 0.996032 |
| H | 6.496891 | 8.388500 | -1.227966 |
| H | 6.442706 | 7.769763 | 0.426333 |
| H | 5.307015 | 9.022513 | -0.087129 |
| H | 3.279546 | 6.765969 | -2.295161 |
| H | 4.614168 | 7.769157 | -2.865514 |
| H | 3.440737 | 8.432757 | -1.720565 |

| | | | |
|---|---|---|---|
| H | -0.155483 | -8.191152 | -5.419645 |
| H | -0.147094 | -8.786497 | -7.072483 |
| H | -3.699447 | -9.185373 | -5.800719 |
| H | -2.320314 | -9.164160 | -4.713720 |
| H | -2.239043 | -10.089403 | -6.216605 |
| H | -2.256203 | -8.969952 | -8.372402 |
| H | -1.813861 | -7.271834 | -8.517387 |
| H | -4.534983 | -8.296582 | -7.613464 |
| H | -4.163827 | -7.709656 | -9.231032 |
| H | -6.123184 | -6.587192 | -6.785974 |
| H | -6.188448 | -6.434835 | -8.544843 |
| H | -6.046495 | -4.997839 | -7.545403 |
| H | -2.593769 | -5.249686 | -8.851988 |
| H | -3.896807 | -4.147280 | -8.434902 |
| H | -4.130582 | -5.335376 | -9.720192 |
| H | -4.732356 | -4.093945 | -6.137017 |
| H | -0.669096 | -6.033557 | -1.803670 |
| H | -1.334104 | -7.012025 | -4.087290 |
| H | -1.786741 | -2.733837 | 0.696704 |
| H | -3.405006 | -1.982491 | -0.970182 |
| H | -4.040805 | -1.721423 | -5.031627 |
| H | -2.398963 | -1.884347 | -4.396079 |
| H | -3.665690 | -1.145035 | -3.399623 |
| H | -5.583870 | -4.276480 | -2.785616 |
| H | -5.913555 | -3.077171 | -4.043952 |
| H | -5.503336 | -2.558339 | -2.402361 |
| H | 1.105505 | 2.411791 | 7.462934 |
| H | 1.179574 | 1.164719 | 9.532162 |
| H | 1.627750 | -0.939108 | 10.743645 |
| H | 2.278658 | -2.961878 | 9.491933 |
| H | 2.535883 | -2.895069 | 7.065533 |

| | | | |
|---|---|---|---|
| H | 4.495703 | -0.790113 | 6.423477 |
| H | 6.741295 | -1.646753 | 6.868677 |
| H | 7.546456 | -3.695944 | 5.759564 |
| H | 6.096656 | -4.874324 | 4.149578 |
| H | 3.978424 | -5.072793 | 3.000572 |
| H | 2.219722 | -6.314717 | 3.249533 |
| H | -0.972882 | 5.517150 | 5.375800 |
| H | 0.402973 | 6.456241 | 3.617973 |
| H | 0.856196 | -7.852879 | 1.919894 |
| H | -0.729579 | -6.964930 | 0.256981 |
| H | -0.531000 | 3.250336 | 6.221701 |
| H | 8.654562 | 2.801498 | 0.633431 |
| H | -0.091783 | -7.048169 | -6.762150 |
| H | 0.566721 | -2.984501 | 1.128774 |
| H | 2.676883 | 2.828772 | 3.442298 |
| C | 5.780955 | 3.488355 | -0.414712 |
| C | 6.866238 | 3.303696 | -1.276522 |
| C | 8.131879 | 1.897813 | 0.324303 |
| C | 6.831293 | 0.798080 | -1.513554 |
| C | 7.673388 | 2.023203 | -1.136812 |
| C | 8.935422 | 2.054479 | -2.011549 |
| C | 8.669149 | 2.681082 | -3.368300 |
| C | 7.761375 | 4.626586 | -4.602887 |
| C | 9.507928 | 5.004798 | -2.859788 |
| C | 8.284198 | 4.157513 | -3.235498 |
| C | 6.556992 | 5.551638 | -2.098271 |
| C | 5.563542 | 5.758866 | -1.175638 |
| C | 5.129956 | 4.705412 | -0.359665 |
| C | 4.095837 | 5.213000 | 0.535746 |
| C | 3.338895 | 4.582285 | 1.509694 |
| C | 2.497598 | 5.340726 | 2.330931 |

| | | | |
|---|---|---|---|
| C | 2.465445 | 6.727027 | 2.142699 |
| C | 3.167497 | 7.350112 | 1.126003 |
| C | 3.975610 | 6.591950 | 0.310395 |
| C | 5.837051 | 8.127034 | -0.403751 |
| C | 4.845586 | 7.043041 | -0.837714 |
| C | 3.988486 | 7.536847 | -2.006475 |
| C | 7.211196 | 4.319903 | -2.174124 |
| C | -2.072821 | -6.376274 | -4.550062 |
| C | -2.580971 | -6.695197 | -5.808774 |
| C | -0.514838 | -7.992430 | -6.425229 |
| C | -2.613433 | -9.174787 | -5.760703 |
| C | -2.049593 | -7.945710 | -6.487930 |
| C | -2.445619 | -7.972608 | -7.968239 |
| C | -3.904069 | -7.605234 | -8.174934 |
| C | -5.736368 | -6.034331 | -7.639420 |
| C | -3.672488 | -5.163996 | -8.748050 |
| C | -4.207850 | -6.172811 | -7.721575 |
| C | -3.988162 | -4.737059 | -5.693971 |
| C | -3.474633 | -4.422553 | -4.460778 |
| C | -2.513988 | -5.256203 | -3.877027 |
| C | -2.172024 | -4.719110 | -2.569674 |
| C | -1.270234 | -5.154696 | -1.619871 |
| C | -1.125044 | -4.435187 | -0.433530 |
| C | -1.893201 | -3.292558 | -0.220940 |
| C | -2.812959 | -2.861935 | -1.169018 |
| C | -2.946178 | -3.571456 | -2.344208 |
| C | -3.455274 | -1.916390 | -4.135879 |
| C | -3.822458 | -3.277368 | -3.539647 |
| C | -5.304433 | -3.299257 | -3.169844 |
| C | -3.567864 | -5.882769 | -6.375522 |
| C | 1.883677 | 3.429537 | 3.857391 |

| | | | |
|---|---|---|---|
| C | 1.664721 | 4.726960 | 3.384837 |
| C | 0.625089 | 5.460615 | 3.960665 |
| C | -0.164540 | 4.931718 | 4.960764 |
| C | 0.081259 | 3.662520 | 5.435764 |
| C | 1.111173 | 2.900037 | 4.886805 |
| C | 1.998313 | -0.813215 | 6.875957 |
| C | 2.239784 | -2.003611 | 7.597092 |
| C | 2.098447 | -2.041013 | 8.957029 |
| C | 1.722327 | -0.892369 | 9.668855 |
| C | 1.476410 | 0.272910 | 9.000468 |
| C | 1.592559 | 0.336803 | 7.595805 |
| C | 1.338584 | 1.525120 | 6.892560 |
| C | 1.390968 | 1.599212 | 5.519032 |
| C | 1.746551 | 0.427537 | 4.804625 |
| C | 2.117175 | -0.727789 | 5.458422 |
| C | 0.613062 | -4.045636 | 1.322719 |
| C | -0.185160 | -4.918755 | 0.584335 |
| C | -0.086976 | -6.291669 | 0.802678 |
| C | 0.793107 | -6.788551 | 1.743097 |
| C | 1.573452 | -5.920490 | 2.480305 |
| C | 1.497217 | -4.537983 | 2.278539 |
| C | 2.358932 | -3.697208 | 3.126591 |
| C | 3.604658 | -4.174942 | 3.470488 |
| C | 4.445415 | -3.520261 | 4.385917 |
| C | 5.746422 | -3.990546 | 4.663302 |
| C | 6.549754 | -3.335270 | 5.552653 |
| C | 6.087948 | -2.173815 | 6.189563 |
| C | 4.828521 | -1.698881 | 5.948181 |
| C | 3.965761 | -2.367389 | 5.054302 |
| C | 2.668917 | -1.881446 | 4.719936 |
| C | 1.939912 | -2.479926 | 3.717402 |

TS-Re

ONIOM(PBE-D3BJ/def2-SVP: GFN2-xTB, gas phase) G = -1707.249287

ONIOM(B2PLYP-D3BJ/def2-TZVP(ALPB:n-hexane):GFN2-xTB) G = -1708.891100

Activation energy: ΔG‡ = 19.1 kcal/mol

Number of imaginary frequencies: 1 (-152.57 cm-1)

Coordinates:

| | | | |
|---|---|---|---|
| C | -1.743111 | -0.050479 | -3.414730 |
| C | -1.313791 | 0.930496 | -4.286555 |
| O | -0.106044 | 0.787207 | -4.871552 |
| C | 0.359194 | 1.825065 | -5.743085 |
| O | -1.923381 | 2.078621 | -4.540163 |
| Si | -3.603940 | 2.560762 | -4.686309 |
| C | -4.002769 | 2.296877 | -6.541686 |
| C | -3.667090 | 0.844881 | -6.931878 |
| C | -5.507204 | 2.559145 | -6.753017 |
| C | -3.181097 | 3.273538 | -7.401553 |
| C | -4.772229 | 1.566823 | -3.601208 |
| C | -3.545472 | 4.370966 | -4.203132 |
| H | -2.722121 | 0.035103 | -2.934899 |
| H | -1.251394 | -1.027481 | -3.428535 |
| H | 0.430437 | 2.802104 | -5.223741 |
| H | -0.304521 | 1.931496 | -6.625436 |
| H | 1.363710 | 1.509932 | -6.075981 |
| H | -4.214711 | 0.104836 | -6.313093 |
| H | -2.584443 | 0.625439 | -6.829230 |
| H | -3.940655 | 0.656939 | -7.993639 |
| H | -5.799290 | 3.588028 | -6.454541 |
| H | -5.770149 | 2.445273 | -7.828074 |
| H | -6.138863 | 1.847449 | -6.183539 |
| H | -3.430859 | 4.331952 | -7.182003 |

| | | | |
|---|---|---|---|
| H | -3.381833 | 3.103310 | -8.482533 |
| H | -2.089948 | 3.145020 | -7.243436 |
| H | -4.955241 | 0.553611 | -4.010352 |
| H | -4.443947 | 1.457795 | -2.548036 |
| H | -5.746322 | 2.100014 | -3.589704 |
| H | -2.685576 | 4.877588 | -4.683567 |
| H | -3.440082 | 4.480048 | -3.106229 |
| H | -4.475138 | 4.888104 | -4.516496 |
| H | 2.732092 | -1.145684 | -3.678681 |
| H | 4.657775 | -2.986400 | -2.129219 |
| H | 4.583963 | -2.738611 | -3.896939 |
| H | 2.518128 | -4.097846 | -4.069553 |
| C | -0.437396 | 0.410713 | -1.503411 |
| C | -0.413627 | 1.899579 | -1.424143 |
| C | -1.741511 | 2.637098 | -1.315957 |
| C | 0.765645 | -0.265275 | -1.790990 |
| N | 0.809033 | -1.596293 | -1.711723 |
| N | 1.868784 | -2.226586 | -2.090017 |
| C | 3.062994 | -1.645877 | -2.738660 |
| C | 3.967169 | -2.858297 | -2.985717 |
| C | 2.989602 | -4.045277 | -3.065240 |
| C | 1.937080 | -3.691124 | -2.013758 |
| H | 3.470956 | -5.022035 | -2.867956 |
| H | 2.264210 | -3.983504 | -0.991160 |
| H | 0.928051 | -4.111892 | -2.187022 |
| H | -1.244133 | -0.135659 | -0.990793 |
| H | 0.200370 | 2.115322 | -0.515574 |
| H | 0.208123 | 2.305184 | -2.253680 |
| H | -2.279256 | 2.352646 | -0.396961 |
| H | -1.568190 | 3.730865 | -1.305450 |
| H | -2.399506 | 2.405318 | -2.169162 |

| | | | |
|---|---|---|---|
| H | 1.647924 | 0.332180 | -2.080084 |
| H | 3.511225 | -0.876259 | -2.078360 |
| N | -0.053403 | 0.101169 | 1.846873 |
| P | -1.418217 | 0.579883 | 2.510406 |
| N | -2.521079 | 0.981227 | 1.415634 |
| S | -3.815454 | 0.269007 | 0.836477 |
| O | -3.722151 | 0.085187 | -0.641319 |
| O | -4.360005 | -0.856354 | 1.627908 |
| C | -5.076230 | 1.669941 | 1.014215 |
| F | -6.047635 | 1.521321 | 0.098776 |
| F | -4.505756 | 2.872247 | 0.820841 |
| F | -5.626694 | 1.635387 | 2.236432 |
| O | -1.085994 | 1.921760 | 3.402053 |
| O | -2.059334 | -0.533452 | 3.525402 |
| F | 5.798420 | -1.544123 | -0.201592 |
| N | 2.380920 | -0.734417 | 1.207643 |
| P | 1.333636 | -0.452693 | 2.397089 |
| S | 3.808307 | -0.123972 | 0.878030 |
| O | 2.029767 | 0.562362 | 3.474885 |
| O | 1.004095 | -1.854434 | 3.191770 |
| O | 4.458178 | 0.709850 | 1.912738 |
| O | 3.860240 | 0.384476 | -0.522198 |
| C | 4.877065 | -1.691313 | 0.767609 |
| F | 5.511774 | -1.903626 | 1.925752 |
| F | 4.135852 | -2.778110 | 0.460568 |
| H | -2.855766 | -2.808327 | 3.786147 |
| C | -2.301258 | 2.531657 | 3.811184 |
| C | -3.034726 | 1.863810 | 4.763622 |
| C | -4.359415 | 2.277738 | 5.066995 |
| C | -5.212510 | 1.543951 | 5.916118 |
| C | -6.496515 | 1.958543 | 6.136670 |

| | | | |
|---|---|---|---|
| C | -6.985685 | 3.123712 | 5.526901 |
| C | -6.185028 | 3.848901 | 4.690925 |
| C | -4.858587 | 3.442763 | 4.433251 |
| C | -4.008714 | 4.176598 | 3.588263 |
| C | -2.732288 | 3.763833 | 3.265731 |
| C | -1.874282 | 4.668606 | 2.480839 |
| C | -2.016463 | 6.043192 | 2.694101 |
| C | -1.256590 | 6.953968 | 1.987412 |
| C | -0.337095 | 6.511694 | 1.055633 |
| C | -0.161769 | 5.147928 | 0.843907 |
| C | -0.926560 | 4.232418 | 1.562100 |
| H | -0.806966 | 3.176380 | 1.379813 |
| H | -1.264668 | 5.559936 | -6.580158 |
| H | -8.311711 | -1.697704 | 1.686260 |
| H | 0.590419 | -3.057702 | 6.277532 |
| H | 0.270584 | 7.217216 | 0.508103 |
| H | -1.377569 | 8.012549 | 2.169776 |
| H | -0.464399 | -6.359397 | 3.832004 |
| H | 1.027872 | -5.332155 | 5.449613 |
| H | -2.707029 | 6.403550 | 3.441447 |
| H | -4.395667 | 5.084283 | 3.150165 |
| H | -6.556303 | 4.740951 | 4.207147 |
| H | -8.001493 | 3.436610 | 5.718453 |
| H | -7.146752 | 1.384604 | 6.780009 |
| H | -4.845627 | 0.639076 | 6.376015 |
| C | -2.409091 | 0.762819 | 5.525573 |
| C | -1.962587 | -0.384632 | 4.913060 |
| C | -1.473681 | -1.481326 | 5.667231 |
| C | -1.362458 | -1.337057 | 7.029854 |
| C | -1.711954 | -0.146692 | 7.688635 |
| C | -1.561214 | -0.005384 | 9.084090 |

| | | | |
|---|---|---|---|
| C | -1.902520 | 1.159980 | 9.709500 |
| C | -2.409059 | 2.232638 | 8.961154 |
| C | -2.585999 | 2.117756 | 7.609657 |
| C | -2.254357 | 0.922666 | 6.933918 |
| H | -2.975884 | 2.953248 | 7.048644 |
| H | -2.660762 | 3.156530 | 9.460674 |
| H | -1.781875 | 1.265872 | 10.777623 |
| H | -1.163236 | -0.839064 | 9.643650 |
| H | -1.015365 | -2.168613 | 7.625171 |
| C | -1.160326 | -2.786961 | 5.068149 |
| C | -0.067130 | -3.503319 | 5.548641 |
| C | 0.174297 | -4.778648 | 5.083889 |
| C | -0.678061 | -5.358218 | 4.165400 |
| C | -1.780216 | -4.665858 | 3.662123 |
| C | -1.998964 | -3.367823 | 4.124957 |
| C | 2.396230 | 5.239845 | -6.526000 |
| C | 4.960411 | 4.020013 | -3.037313 |
| C | 3.525050 | 3.531002 | -3.253287 |
| C | 3.526436 | 2.030168 | -3.552536 |
| C | 2.661948 | 3.857147 | -2.055028 |
| C | 2.708326 | 3.323442 | -0.785522 |
| C | 1.754997 | 3.719913 | 0.143024 |
| C | 0.817535 | 4.705075 | -0.161705 |
| C | 0.789360 | 5.262213 | -1.438830 |
| C | 1.683951 | 4.806785 | -2.389846 |
| C | 1.797524 | 5.098574 | -3.809969 |
| C | 2.850155 | 4.339362 | -4.336773 |
| C | 3.141918 | 4.418281 | -5.675635 |
| C | 2.784996 | 5.268645 | -7.993381 |
| C | 2.363452 | 3.938798 | -8.636271 |
| C | 4.303357 | 5.439605 | -8.155682 |

| | | | |
|---|---|---|---|
| C | 2.115330 | 6.444317 | -8.713125 |
| C | 0.646255 | 6.571556 | -8.352825 |
| C | 0.441991 | 6.865093 | -6.862328 |
| C | 0.785412 | 8.329154 | -6.553604 |
| C | -1.043532 | 6.624921 | -6.552171 |
| C | 1.321666 | 5.969015 | -6.007385 |
| C | 1.044758 | 5.901593 | -4.641875 |
| H | 5.398893 | 3.515316 | -2.182583 |
| H | 5.562995 | 3.816652 | -3.920646 |
| H | 4.975767 | 5.090779 | -2.850142 |
| H | 3.869387 | 1.485768 | -2.675898 |
| H | 2.524285 | 1.691335 | -3.811692 |
| H | 4.192413 | 1.810278 | -4.384801 |
| H | 3.437149 | 2.576270 | -0.520455 |
| H | 1.738463 | 3.265424 | 1.122174 |
| H | 0.234165 | 6.477142 | -4.223375 |
| H | 0.047892 | 6.007843 | -1.688617 |
| H | 3.957930 | 3.835891 | -6.074046 |
| H | 2.634759 | 3.926608 | -9.689923 |
| H | 2.857454 | 3.105089 | -8.143152 |
| H | 1.290188 | 3.787834 | -8.550904 |
| H | 4.847093 | 4.543816 | -7.870391 |
| H | 4.537610 | 5.652131 | -9.197033 |
| H | 4.659630 | 6.266864 | -7.545709 |
| H | 2.219937 | 6.311164 | -9.792452 |
| H | 2.635057 | 7.365949 | -8.445721 |
| H | 0.128017 | 5.644838 | -8.607051 |
| H | 0.191510 | 7.372076 | -8.940909 |
| H | 0.159136 | 8.996701 | -7.142213 |
| H | 0.623741 | 8.542980 | -5.499952 |
| H | 1.827343 | 8.540368 | -6.779589 |

| | | | |
|---|---|---|---|
| H | -1.657925 | 7.125466 | -7.298276 |
| H | -1.323576 | 7.014371 | -5.577593 |
| C | -8.044399 | -4.273571 | -0.975162 |
| C | -4.986369 | -7.624091 | -1.214100 |
| C | -5.602509 | -7.071154 | 0.077576 |
| C | -6.509548 | -8.116231 | 0.730233 |
| C | -4.511509 | -6.603949 | 1.013099 |
| C | -3.595936 | -7.348493 | 1.721180 |
| C | -2.723842 | -6.699643 | 2.579530 |
| C | -2.709548 | -5.308069 | 2.715996 |
| C | -3.624097 | -4.558417 | 1.970328 |
| C | -4.529249 | -5.209514 | 1.152954 |
| C | -5.666811 | -4.695574 | 0.406508 |
| C | -6.335460 | -5.774189 | -0.184068 |
| C | -7.506198 | -5.559402 | -0.867599 |
| C | -9.362998 | -4.126075 | -1.714813 |
| C | -10.482387 | -4.690391 | -0.827554 |
| C | -9.347087 | -4.902118 | -3.041115 |
| C | -9.640071 | -2.656699 | -2.050518 |
| C | -9.369422 | -1.741840 | -0.869544 |
| C | -7.897146 | -1.770870 | -0.441215 |
| C | -7.038578 | -0.985173 | -1.443104 |
| C | -7.807437 | -1.093596 | 0.934047 |
| C | -7.381315 | -3.197864 | -0.374214 |
| C | -6.177283 | -3.418587 | 0.297214 |
| H | -4.424284 | -8.531954 | -1.004704 |
| H | -5.764460 | -7.855093 | -1.938956 |
| H | -4.307377 | -6.893332 | -1.649997 |
| H | -5.943831 | -9.011718 | 0.977526 |
| H | -6.943369 | -7.715495 | 1.643763 |
| H | -7.315708 | -8.392952 | 0.054447 |

| | | | |
|---|---|---|---|
| H | -3.569444 | -8.426413 | 1.641234 |
| H | -2.061039 | -7.307491 | 3.174658 |
| H | -5.639704 | -2.599584 | 0.745621 |
| H | -3.651865 | -3.481820 | 2.027522 |
| H | -8.020891 | -6.395327 | -1.315939 |
| H | -11.444126 | -4.606441 | -1.330645 |
| H | -10.298811 | -5.739085 | -0.605930 |
| H | -10.535359 | -4.155656 | 0.117022 |
| H | -9.382666 | -5.976424 | -2.885136 |
| H | -10.215926 | -4.625706 | -3.636139 |
| H | -8.449378 | -4.666753 | -3.607825 |
| H | -10.680635 | -2.552039 | -2.368655 |
| H | -9.009321 | -2.360424 | -2.890609 |
| H | -9.992214 | -2.046060 | -0.026376 |
| H | -9.646516 | -0.716741 | -1.126750 |
| H | -7.391191 | 0.040996 | -1.514319 |
| H | -6.000180 | -0.961464 | -1.121451 |
| H | -7.084446 | -1.435734 | -2.432181 |
| H | -8.290143 | -0.118936 | 0.892226 |
| H | -6.779362 | -0.945518 | 1.248139 |
| H | 6.840619 | 0.870531 | 1.155928 |
| H | 8.356283 | 0.093548 | 0.725802 |
| H | 6.994273 | 1.559794 | -2.484975 |
| H | 5.993212 | 0.990447 | -1.153188 |
| H | 7.380519 | 0.038215 | -1.672587 |
| H | 9.665997 | 0.782141 | -1.230009 |
| H | 9.995889 | 2.157513 | -0.179488 |
| H | 8.914525 | 2.333962 | -3.020666 |
| H | 10.588570 | 2.616959 | -2.559662 |
| H | 8.104415 | 4.558789 | -3.730550 |
| H | 9.859925 | 4.603471 | -3.936859 |

| | | | |
|---|---|---|---|
| H | 9.032265 | 5.946751 | -3.164824 |
| H | 10.585551 | 4.304597 | -0.227588 |
| H | 10.183966 | 5.854158 | -0.953062 |
| H | 11.305773 | 4.765748 | -1.774150 |
| H | 7.901963 | 6.409708 | -1.424403 |
| H | 3.875999 | 3.498834 | 2.313168 |
| H | 5.774193 | 2.618718 | 0.905866 |
| H | 2.096149 | 7.285483 | 3.304990 |
| H | 3.579150 | 8.415898 | 1.755214 |
| H | 7.322451 | 8.407988 | 0.068002 |
| H | 7.042591 | 7.713049 | 1.668568 |
| H | 6.002897 | 9.012729 | 1.074825 |
| H | 4.210731 | 6.926589 | -1.464153 |
| H | 5.672641 | 7.846719 | -1.853953 |
| H | 4.412998 | 8.567273 | -0.844075 |
| H | 0.172353 | -8.354412 | -5.354983 |
| H | 0.231047 | -9.008950 | -6.984126 |
| H | -3.250245 | -9.728524 | -5.653747 |
| H | -1.874612 | -9.513885 | -4.583023 |
| H | -1.703882 | -10.489132 | -6.045921 |
| H | -1.859143 | -9.471638 | -8.246916 |
| H | -1.595413 | -7.744956 | -8.468420 |
| H | -4.189257 | -9.003950 | -7.489360 |
| H | -3.893494 | -8.453056 | -9.134977 |
| H | -5.939673 | -7.437621 | -6.720541 |
| H | -6.032931 | -7.361271 | -8.483076 |
| H | -6.033398 | -5.879248 | -7.540501 |
| H | -2.582334 | -5.830098 | -8.878016 |
| H | -3.989891 | -4.850846 | -8.494233 |
| H | -4.107509 | -6.111728 | -9.725233 |
| H | -4.807124 | -4.782945 | -6.192597 |

| | | | |
|---|---|---|---|
| H | -0.561437 | -6.143601 | -1.837603 |
| H | -1.109683 | -7.243308 | -4.061563 |
| H | -1.706839 | -2.676617 | 0.401206 |
| H | -3.402155 | -2.126603 | -1.269841 |
| H | -4.636628 | -2.423841 | -5.199320 |
| H | -2.948117 | -2.173990 | -4.737212 |
| H | -4.245805 | -1.632904 | -3.663230 |
| H | -5.423194 | -5.033496 | -2.692731 |
| H | -6.080361 | -3.997201 | -3.960443 |
| H | -5.649732 | -3.317677 | -2.381162 |
| H | 1.122849 | 1.941181 | 7.693732 |
| H | 1.325787 | 0.491136 | 9.620318 |
| H | 1.969334 | -1.678502 | 10.609940 |
| H | 2.788679 | -3.493889 | 9.154815 |
| H | 3.018291 | -3.154705 | 6.751644 |
| H | 4.885330 | -0.876178 | 6.140452 |
| H | 7.152812 | -1.750461 | 6.467593 |
| H | 7.886963 | -3.791659 | 5.299531 |
| H | 6.362847 | -4.939708 | 3.741235 |
| H | 4.191762 | -5.093556 | 2.661720 |
| H | 2.423284 | -6.300962 | 3.022417 |
| H | -0.964473 | 5.243356 | 5.575018 |
| H | 0.534816 | 6.305689 | 3.990954 |
| H | 0.943821 | -7.831935 | 1.815236 |
| H | -0.713967 | -6.939648 | 0.232012 |
| H | -0.533793 | 2.947208 | 6.360090 |
| H | 8.370479 | 1.632250 | 1.593838 |
| H | 0.105168 | -7.263759 | -6.739267 |
| H | 0.717945 | -2.969681 | 0.939374 |
| H | 2.965679 | 2.777452 | 3.945009 |
| C | 6.255026 | 3.438738 | 0.398042 |

| | | | |
|---|---|---|---|
| C | 7.386199 | 3.218330 | -0.389565 |
| C | 7.858006 | 1.056315 | 0.825402 |
| C | 7.012682 | 1.051205 | -1.524298 |
| C | 7.901982 | 1.796568 | -0.518961 |
| C | 9.359742 | 1.803986 | -0.993551 |
| C | 9.554817 | 2.688974 | -2.211813 |
| C | 9.040686 | 4.865668 | -3.269522 |
| C | 10.400483 | 4.811289 | -1.171279 |
| C | 9.227985 | 4.157794 | -1.918282 |
| C | 7.442084 | 5.575118 | -0.917452 |
| C | 6.339443 | 5.788686 | -0.129084 |
| C | 5.745137 | 4.713481 | 0.540849 |
| C | 4.650036 | 5.223870 | 1.350260 |
| C | 3.790660 | 4.569393 | 2.211503 |
| C | 2.833033 | 5.301763 | 2.921070 |
| C | 2.793891 | 6.687410 | 2.740681 |
| C | 3.645974 | 7.342652 | 1.866758 |
| C | 4.577656 | 6.611051 | 1.165896 |
| C | 6.556506 | 8.122190 | 0.785541 |
| C | 5.615585 | 7.081543 | 0.173777 |
| C | 4.935212 | 7.642523 | -1.080388 |
| C | 7.980685 | 4.293064 | -1.060766 |
| C | -1.914576 | -6.708873 | -4.541555 |
| C | -2.399493 | -7.134982 | -5.777473 |
| C | -0.213782 | -8.233795 | -6.362962 |
| C | -2.171275 | -9.600752 | -5.625472 |
| C | -1.745444 | -8.351415 | -6.409697 |
| C | -2.147497 | -8.482632 | -7.882858 |
| C | -3.637617 | -8.276527 | -8.087498 |
| C | -5.618429 | -6.881240 | -7.598529 |
| C | -3.663457 | -5.851728 | -8.766271 |

| | | | |
|---|---|---|---|
| C | -4.084447 | -6.864756 | -7.691687 |
| C | -3.996596 | -5.325005 | -5.730838 |
| C | -3.499938 | -4.898439 | -4.524445 |
| C | -2.456394 | -5.604844 | -3.917368 |
| C | -2.132136 | -4.953064 | -2.659700 |
| C | -1.192128 | -5.276103 | -1.702960 |
| C | -1.049787 | -4.468425 | -0.570784 |
| C | -1.834586 | -3.324087 | -0.453944 |
| C | -2.799549 | -3.010895 | -1.405526 |
| C | -2.963360 | -3.833427 | -2.501672 |
| C | -3.941324 | -2.407092 | -4.361826 |
| C | -3.954447 | -3.755868 | -3.645867 |
| C | -5.370474 | -4.043837 | -3.136831 |
| C | -3.468232 | -6.452366 | -6.366455 |
| C | 2.100362 | 3.330133 | 4.273886 |
| C | 1.890522 | 4.639651 | 3.840817 |
| C | 0.761578 | 5.305686 | 4.321413 |
| C | -0.100471 | 4.702190 | 5.215974 |
| C | 0.135781 | 3.416156 | 5.656225 |
| C | 1.234565 | 2.715097 | 5.168796 |
| C | 2.310851 | -1.115177 | 6.775558 |
| C | 2.655121 | -2.349324 | 7.371075 |
| C | 2.525996 | -2.541064 | 8.719401 |
| C | 2.053442 | -1.511682 | 9.546151 |
| C | 1.699697 | -0.310527 | 9.000698 |
| C | 1.804844 | -0.088396 | 7.611589 |
| C | 1.447066 | 1.144539 | 7.040333 |
| C | 1.530189 | 1.372068 | 5.688564 |
| C | 1.973867 | 0.317414 | 4.851165 |
| C | 2.416155 | -0.875637 | 5.372570 |
| C | 0.756373 | -4.030118 | 1.146968 |

| | | | |
|---|---|---|---|
| C | -0.090866 | -4.901298 | 0.460611 |
| C | -0.022383 | -6.270182 | 0.720342 |
| C | 0.895432 | -6.769884 | 1.620162 |
| C | 1.738484 | -5.905710 | 2.287865 |
| C | 1.674975 | -4.526461 | 2.069836 |
| C | 2.592526 | -3.707054 | 2.884120 |
| C | 3.849022 | -4.202198 | 3.166289 |
| C | 4.736120 | -3.569207 | 4.053680 |
| C | 6.042496 | -4.055392 | 4.273395 |
| C | 6.886976 | -3.417541 | 5.136791 |
| C | 6.465387 | -2.260483 | 5.809337 |
| C | 5.200241 | -1.774098 | 5.631361 |
| C | 4.299073 | -2.422356 | 4.762213 |
| C | 2.985977 | -1.936876 | 4.516375 |
| C | 2.214528 | -2.508611 | 3.530657 |

P-Re

ONIOM(PBE-D3BJ/def2-SVP: GFN2-xTB, gas phase) G = -1707.294189

ONIOM(B2PLYP-D3BJ/def2-TZVP(ALPB:n-hexane):GFN2-xTB) G = -1708.934878

Number of imaginary frequencies: 0

Coordinates:

| | | | |
|---|---|---|---|
| C | -1.054840 | -1.333051 | -2.289257 |
| C | -0.963439 | -1.267296 | -3.750560 |
| O | -0.330207 | -2.278773 | -4.293621 |
| C | -0.080143 | -2.302774 | -5.714170 |
| O | -1.320083 | -0.315259 | -4.533023 |
| Si | -2.295705 | 1.196329 | -4.700165 |
| C | -2.852536 | 1.018635 | -6.536520 |
| C | -3.622730 | -0.297353 | -6.759475 |
| C | -3.788615 | 2.212354 | -6.837999 |
| C | -1.627506 | 1.076922 | -7.470758 |

| | | | |
|---|---|---|---|
| C | -3.738294 | 1.224589 | -3.519812 |
| C | -1.071787 | 2.597067 | -4.512017 |
| H | -1.946669 | -0.808486 | -1.887706 |
| H | -1.102547 | -2.398763 | -2.013547 |
| H | 0.419810 | -1.369968 | -6.036587 |
| H | -1.036539 | -2.435226 | -6.256884 |
| H | 0.571799 | -3.177917 | -5.869208 |
| H | -4.521125 | -0.364015 | -6.113562 |
| H | -2.995732 | -1.188397 | -6.552145 |
| H | -3.963579 | -0.363791 | -7.815778 |
| H | -3.280496 | 3.188705 | -6.700755 |
| H | -4.133467 | 2.165445 | -7.893782 |
| H | -4.690241 | 2.207653 | -6.192586 |
| H | -1.063644 | 2.024146 | -7.354016 |
| H | -1.949411 | 1.002306 | -8.532145 |
| H | -0.923572 | 0.243998 | -7.274335 |
| H | -3.514375 | 0.779822 | -2.526207 |
| H | -4.009016 | 2.284525 | -3.338923 |
| H | -4.625011 | 0.706463 | -3.930074 |
| H | -0.126518 | 2.419076 | -5.060681 |
| H | -0.828808 | 2.778457 | -3.449547 |
| H | -1.520301 | 3.527763 | -4.916234 |
| H | 3.984064 | -2.510488 | -3.373136 |
| H | 5.714136 | -3.153966 | -0.932954 |
| H | 6.044039 | -3.580945 | -2.637205 |
| H | 4.400492 | -5.434818 | -2.550710 |
| C | 0.255777 | -0.768513 | -1.583052 |
| C | 0.428235 | 0.734904 | -1.795215 |
| C | -0.693716 | 1.570153 | -1.195742 |
| C | 1.511415 | -1.502090 | -1.944342 |
| N | 1.748204 | -2.688909 | -1.470007 |

| | | | |
|---|---|---|---|
| N | 2.902721 | -3.288090 | -1.717001 |
| C | 4.088247 | -2.613853 | -2.267027 |
| C | 5.252081 | -3.536333 | -1.864680 |
| C | 4.583107 | -4.896898 | -1.596420 |
| C | 3.253667 | -4.489964 | -0.960482 |
| H | 5.185874 | -5.552870 | -0.939840 |
| H | 3.379230 | -4.248542 | 0.120227 |
| H | 2.445979 | -5.243202 | -1.049994 |
| H | 0.038330 | -0.939500 | -0.506459 |
| H | 1.397187 | 1.013026 | -1.331336 |
| H | 0.552907 | 0.949454 | -2.881700 |
| H | -0.757074 | 1.379355 | -0.107268 |
| H | -0.532522 | 2.652808 | -1.354446 |
| H | -1.694825 | 1.303637 | -1.585943 |
| H | 2.270254 | -0.936256 | -2.520599 |
| H | 4.170410 | -1.585430 | -1.843684 |
| N | -0.115998 | 0.000214 | 1.780506 |
| P | -1.418501 | 0.544513 | 2.515378 |
| N | -2.549710 | 1.022221 | 1.498782 |
| S | -3.635560 | 0.175009 | 0.786729 |
| O | -3.425422 | -0.046143 | -0.625104 |
| O | -4.212499 | -0.918785 | 1.507307 |
| C | -4.930389 | 1.544222 | 0.813085 |
| F | -5.938735 | 1.382262 | -0.052893 |
| F | -4.369357 | 2.719605 | 0.516464 |
| F | -5.468692 | 1.644739 | 2.030877 |
| O | -1.078558 | 1.850089 | 3.388918 |
| O | -2.036995 | -0.527526 | 3.546097 |
| F | 6.085585 | -0.791530 | 0.833939 |
| N | 2.327444 | -0.911477 | 1.327848 |
| P | 1.235279 | -0.535287 | 2.431643 |

| | | | |
|---|---|---|---|
| S | 3.481248 | 0.037364 | 0.893363 |
| O | 1.866818 | 0.490397 | 3.493637 |
| O | 0.931097 | -1.879037 | 3.262201 |
| O | 3.631660 | 1.280277 | 1.583306 |
| O | 3.693029 | 0.064466 | -0.528356 |
| C | 4.940954 | -1.010129 | 1.490116 |
| F | 5.180391 | -0.784855 | 2.785161 |
| F | 4.678499 | -2.314478 | 1.343561 |
| H | -3.056355 | -2.827896 | 3.937685 |
| C | -2.260273 | 2.490343 | 3.795051 |
| C | -2.996446 | 1.862475 | 4.774309 |
| C | -4.319610 | 2.289163 | 5.068136 |
| C | -5.174223 | 1.587757 | 5.943470 |
| C | -6.460130 | 2.007091 | 6.143954 |
| C | -6.952196 | 3.143797 | 5.485064 |
| C | -6.151275 | 3.834985 | 4.621007 |
| C | -4.822568 | 3.423711 | 4.385529 |
| C | -3.975595 | 4.120044 | 3.507359 |
| C | -2.698987 | 3.697867 | 3.199173 |
| C | -1.867431 | 4.574883 | 2.355583 |
| C | -2.022096 | 5.955200 | 2.517216 |
| C | -1.314511 | 6.845795 | 1.734802 |
| C | -0.433896 | 6.377404 | 0.779266 |
| C | -0.237299 | 5.008752 | 0.623899 |
| C | -0.951058 | 4.114350 | 1.417611 |
| H | -0.820845 | 3.054116 | 1.277613 |
| H | -1.627600 | 5.056769 | -6.854252 |
| H | -8.540050 | -1.445519 | 1.854599 |
| H | 0.550075 | -3.132647 | 6.176864 |
| H | 0.129517 | 7.068137 | 0.169375 |
| H | -1.447190 | 7.909527 | 1.875135 |

| | | | |
|---|---|---|---|
| H | -0.684271 | -6.388089 | 3.738627 |
| H | 0.922076 | -5.396424 | 5.276222 |
| H | -2.683800 | 6.335847 | 3.280741 |
| H | -4.368688 | 5.001881 | 3.024286 |
| H | -6.524155 | 4.702963 | 4.096820 |
| H | -7.970211 | 3.459646 | 5.659396 |
| H | -7.109813 | 1.457563 | 6.809021 |
| H | -4.808645 | 0.702583 | 6.441171 |
| C | -2.392282 | 0.754742 | 5.544208 |
| C | -1.974754 | -0.401696 | 4.925727 |
| C | -1.519734 | -1.513104 | 5.680501 |
| C | -1.392560 | -1.374769 | 7.041401 |
| C | -1.713139 | -0.178029 | 7.704236 |
| C | -1.554911 | -0.042980 | 9.099272 |
| C | -1.878658 | 1.124982 | 9.729268 |
| C | -2.376243 | 2.205644 | 8.986520 |
| C | -2.558552 | 2.097771 | 7.635124 |
| C | -2.240625 | 0.902071 | 6.953903 |
| H | -2.945705 | 2.936916 | 7.077728 |
| H | -2.617925 | 3.129861 | 9.490455 |
| H | -1.753045 | 1.225537 | 10.797279 |
| H | -1.167678 | -0.883898 | 9.655364 |
| H | -1.058438 | -2.216378 | 7.630261 |
| C | -1.262732 | -2.829454 | 5.076803 |
| C | -0.153230 | -3.564528 | 5.482979 |
| C | 0.046541 | -4.834878 | 4.982842 |
| C | -0.873307 | -5.398136 | 4.120439 |
| C | -2.020553 | -4.703174 | 3.735645 |
| C | -2.182939 | -3.400404 | 4.207389 |
| C | 2.041948 | 4.888120 | -6.834117 |
| C | 4.764249 | 3.871430 | -3.393211 |

| | | | |
|---|---|---|---|
| C | 3.340612 | 3.328292 | -3.548340 |
| C | 3.383445 | 1.820649 | -3.809155 |
| C | 2.509772 | 3.670616 | -2.331625 |
| C | 2.605653 | 3.177850 | -1.047932 |
| C | 1.679065 | 3.586887 | -0.099189 |
| C | 0.713443 | 4.544166 | -0.399982 |
| C | 0.634579 | 5.062623 | -1.692634 |
| C | 1.505085 | 4.593628 | -2.659773 |
| C | 1.560880 | 4.841024 | -4.091845 |
| C | 2.607355 | 4.084017 | -4.632772 |
| C | 2.842690 | 4.118612 | -5.984559 |
| C | 2.376017 | 4.879561 | -8.315428 |
| C | 1.961095 | 3.524861 | -8.908401 |
| C | 3.883820 | 5.076123 | -8.537645 |
| C | 1.657699 | 6.024144 | -9.038321 |
| C | 0.195448 | 6.118868 | -8.642291 |
| C | 0.020543 | 6.435436 | -7.152221 |
| C | 0.294755 | 7.922805 | -6.890116 |
| C | -1.442005 | 6.128060 | -6.794471 |
| C | 0.969124 | 5.608235 | -6.300142 |
| C | 0.756060 | 5.593868 | -4.921549 |
| H | 5.250150 | 3.400230 | -2.544861 |
| H | 5.341824 | 3.671485 | -4.294170 |
| H | 4.746160 | 4.945337 | -3.223508 |
| H | 3.728044 | 1.298670 | -2.919631 |
| H | 2.391193 | 1.453945 | -4.065357 |
| H | 4.058790 | 1.598063 | -4.633350 |
| H | 3.360856 | 2.458802 | -0.774305 |
| H | 1.703896 | 3.147044 | 0.884742 |
| H | -0.048007 | 6.169553 | -4.490414 |
| H | -0.131788 | 5.783343 | -1.940742 |

| | | | |
|---|---|---|---|
| H | 3.655453 | 3.539857 | -6.395270 |
| H | 2.206815 | 3.485366 | -9.967777 |
| H | 2.481716 | 2.714753 | -8.403076 |
| H | 0.892844 | 3.357871 | -8.794057 |
| H | 4.455649 | 4.198101 | -8.251892 |
| H | 4.075736 | 5.268045 | -9.591680 |
| H | 4.245206 | 5.924613 | -7.960755 |
| H | 1.738327 | 5.874828 | -10.117681 |
| H | 2.159665 | 6.963245 | -8.798867 |
| H | -0.302367 | 5.173810 | -8.868362 |
| H | -0.296670 | 6.895009 | -9.233004 |
| H | -0.381619 | 8.542151 | -7.476107 |
| H | 0.153131 | 8.157069 | -5.838144 |
| H | 1.317462 | 8.179784 | -7.153710 |
| H | -2.102953 | 6.629924 | -7.498763 |
| H | -1.698933 | 6.468399 | -5.795767 |
| C | -8.472561 | -4.222121 | -0.647034 |
| C | -5.747106 | -7.835555 | -0.653951 |
| C | -6.225597 | -7.101631 | 0.602942 |
| C | -7.173965 | -7.989509 | 1.413241 |
| C | -5.048940 | -6.643605 | 1.434564 |
| C | -4.100793 | -7.392836 | 2.094274 |
| C | -3.123380 | -6.742356 | 2.830178 |
| C | -3.055108 | -5.348291 | 2.909392 |
| C | -4.018595 | -4.595625 | 2.232897 |
| C | -5.002096 | -5.245410 | 1.512662 |
| C | -6.138766 | -4.711359 | 0.782123 |
| C | -6.873478 | -5.779527 | 0.255886 |
| C | -8.023689 | -5.531801 | -0.450703 |
| C | -9.762852 | -4.034701 | -1.426749 |
| C | -10.934055 | -4.501673 | -0.550195 |

| | | | |
|---|---|---|---|
| C | -9.747259 | -4.866435 | -2.719178 |
| C | -9.958343 | -2.568482 | -1.829938 |
| C | -9.635421 | -1.614708 | -0.693348 |
| C | -8.166303 | -1.707892 | -0.266698 |
| C | -7.257154 | -1.070202 | -1.326303 |
| C | -8.015906 | -0.933015 | 1.050063 |
| C | -7.748461 | -3.157290 | -0.099751 |
| C | -6.568102 | -3.413257 | 0.599069 |
| H | -5.269332 | -8.774808 | -0.383711 |
| H | -6.586459 | -8.047503 | -1.312808 |
| H | -5.025100 | -7.223264 | -1.190660 |
| H | -6.668165 | -8.900229 | 1.726127 |
| H | -7.515511 | -7.459623 | 2.299767 |
| H | -8.040849 | -8.261583 | 0.815154 |
| H | -4.118850 | -8.473212 | 2.060925 |
| H | -2.416703 | -7.346139 | 3.377680 |
| H | -5.982855 | -2.603306 | 1.004495 |
| H | -4.014740 | -3.517228 | 2.257741 |
| H | -8.590367 | -6.358265 | -0.851281 |
| H | -11.874437 | -4.400643 | -1.089067 |
| H | -10.808141 | -5.544328 | -0.268506 |
| H | -10.991400 | -3.914857 | 0.362814 |
| H | -9.840138 | -5.930019 | -2.519705 |
| H | -10.583205 | -4.573987 | -3.352271 |
| H | -8.821955 | -4.699639 | -3.266384 |
| H | -10.992088 | -2.422128 | -2.153202 |
| H | -9.314842 | -2.344596 | -2.682509 |
| H | -10.269934 | -1.843528 | 0.164893 |
| H | -9.855387 | -0.589231 | -0.999092 |
| H | -7.552791 | -0.039772 | -1.507753 |
| H | -6.226280 | -1.067175 | -0.980571 |

| | | | |
|---|---|---|---|
| H | -7.305041 | -1.621405 | -2.262369 |
| H | -8.441690 | 0.061991 | 0.937034 |
| H | -6.975235 | -0.816346 | 1.337044 |
| H | 7.071958 | 1.498770 | 1.289285 |
| H | 8.613749 | 0.823154 | 0.793082 |
| H | 6.821722 | 1.495254 | -2.440863 |
| H | 5.925970 | 1.159506 | -0.961506 |
| H | 7.362236 | 0.220659 | -1.343892 |
| H | 9.535216 | 1.290959 | -1.514429 |
| H | 9.889756 | 2.749282 | -0.596294 |
| H | 8.447458 | 2.683267 | -3.276528 |
| H | 10.146272 | 3.093634 | -3.050319 |
| H | 7.559254 | 4.855679 | -4.098862 |
| H | 9.300869 | 4.985055 | -4.370603 |
| H | 8.432037 | 6.309911 | -3.614900 |
| H | 10.147810 | 4.904830 | -0.666352 |
| H | 9.647847 | 6.376941 | -1.483543 |
| H | 10.814335 | 5.298160 | -2.255730 |
| H | 7.204271 | 6.707699 | -1.933015 |
| H | 3.663709 | 3.653571 | 2.173246 |
| H | 5.608398 | 2.889548 | 0.749287 |
| H | 1.567353 | 7.315777 | 3.014271 |
| H | 2.841190 | 8.466433 | 1.320683 |
| H | 6.422672 | 8.742720 | -0.628399 |
| H | 6.306374 | 8.188416 | 1.045658 |
| H | 5.081516 | 9.279027 | 0.387677 |
| H | 3.486977 | 6.716263 | -1.842504 |
| H | 4.766173 | 7.816443 | -2.379285 |
| H | 3.446760 | 8.417364 | -1.367178 |
| H | 0.821821 | -6.441845 | -6.059050 |
| H | 1.008067 | -6.705206 | -7.785817 |

| | | | |
|---|---|---|---|
| H | -2.242644 | -8.377348 | -6.918291 |
| H | -0.895052 | -8.239638 | -5.799858 |
| H | -0.595136 | -8.669988 | -7.485981 |
| H | -0.969391 | -7.085946 | -9.267526 |
| H | -1.062321 | -5.356843 | -8.955706 |
| H | -3.333357 | -7.339257 | -8.527373 |
| H | -3.177316 | -6.260438 | -9.909774 |
| H | -5.378499 | -6.450943 | -7.436154 |
| H | -5.471941 | -5.857015 | -9.096627 |
| H | -5.778832 | -4.763359 | -7.758038 |
| H | -2.404787 | -3.638118 | -8.764410 |
| H | -3.986719 | -3.125471 | -8.187133 |
| H | -3.838827 | -3.923364 | -9.755023 |
| H | -4.831996 | -4.009763 | -6.017505 |
| H | -0.270893 | -5.659675 | -2.057545 |
| H | -0.626244 | -6.124510 | -4.569431 |
| H | -2.045715 | -3.216206 | 0.963858 |
| H | -3.950377 | -2.716563 | -0.501707 |
| H | -5.227672 | -2.058335 | -4.231140 |
| H | -3.504072 | -1.756670 | -3.975607 |
| H | -4.621128 | -1.649423 | -2.618084 |
| H | -5.358694 | -5.400993 | -2.685306 |
| H | -6.210538 | -4.255899 | -3.722046 |
| H | -5.945638 | -3.881846 | -2.010785 |
| H | 1.123592 | 2.125196 | 7.646681 |
| H | 1.305888 | 0.769865 | 9.640822 |
| H | 1.866884 | -1.378158 | 10.722710 |
| H | 2.581745 | -3.294964 | 9.344789 |
| H | 2.802665 | -3.075795 | 6.926562 |
| H | 4.697642 | -0.847751 | 6.331219 |
| H | 6.970351 | -1.675747 | 6.701584 |

| | | | |
|---|---|---|---|
| H | 7.777063 | -3.690479 | 5.533844 |
| H | 6.302337 | -4.869083 | 3.947027 |
| H | 4.152295 | -5.088923 | 2.859721 |
| H | 2.443497 | -6.358939 | 3.093853 |
| H | -1.078228 | 5.276805 | 5.696185 |
| H | 0.297218 | 6.364946 | 4.034361 |
| H | 1.121314 | -7.897728 | 1.730801 |
| H | -0.508816 | -7.005092 | 0.100705 |
| H | -0.572316 | 2.983023 | 6.430014 |
| H | 8.528703 | 2.487661 | 1.386064 |
| H | 0.549743 | -5.110687 | -7.187282 |
| H | 0.644628 | -3.017399 | 1.107933 |
| H | 2.692883 | 2.820815 | 3.701126 |
| C | 5.982228 | 3.719682 | 0.170991 |
| C | 7.110519 | 3.572686 | -0.638665 |
| C | 8.011778 | 1.729481 | 0.800083 |
| C | 6.921259 | 1.213707 | -1.395004 |
| C | 7.800872 | 2.220046 | -0.639364 |
| C | 9.183658 | 2.302576 | -1.298433 |
| C | 9.161532 | 3.121281 | -2.576613 |
| C | 8.492416 | 5.227479 | -3.682852 |
| C | 9.918991 | 5.335072 | -1.637651 |
| C | 8.767754 | 4.579072 | -2.317026 |
| C | 6.864406 | 5.868021 | -1.346521 |
| C | 5.772782 | 6.012906 | -0.527979 |
| C | 5.328984 | 4.932493 | 0.243250 |
| C | 4.215221 | 5.377984 | 1.067241 |
| C | 3.467967 | 4.701714 | 2.014524 |
| C | 2.470977 | 5.380326 | 2.724715 |
| C | 2.291665 | 6.742535 | 2.459647 |
| C | 3.025085 | 7.415783 | 1.497544 |

| | | | |
|---|---|---|---|
| C | 3.987705 | 6.732414 | 0.791195 |
| C | 5.732798 | 8.439210 | 0.155967 |
| C | 4.909555 | 7.231586 | -0.295954 |
| C | 4.100511 | 7.568175 | -1.554986 |
| C | 7.547837 | 4.650982 | -1.415399 |
| C | -1.543135 | -5.664609 | -4.902278 |
| C | -1.941045 | -5.771802 | -6.235179 |
| C | 0.421508 | -6.180695 | -7.033823 |
| C | -1.207507 | -8.059394 | -6.825382 |
| C | -1.056729 | -6.572233 | -7.177483 |
| C | -1.446081 | -6.329275 | -8.640222 |
| C | -2.949264 | -6.369066 | -8.846851 |
| C | -5.169410 | -5.601645 | -8.082889 |
| C | -3.459391 | -3.902575 | -8.735442 |
| C | -3.669668 | -5.267325 | -8.062216 |
| C | -3.911753 | -4.479344 | -5.706938 |
| C | -3.510037 | -4.373979 | -4.398573 |
| C | -2.313946 | -4.974742 | -3.989543 |
| C | -2.105981 | -4.676414 | -2.581927 |
| C | -1.076457 | -5.021729 | -1.725968 |
| C | -1.071369 | -4.507068 | -0.430640 |
| C | -2.089819 | -3.649763 | -0.022494 |
| C | -3.161707 | -3.359230 | -0.856477 |
| C | -3.169871 | -3.875476 | -2.135916 |
| C | -4.400746 | -2.185931 | -3.535663 |
| C | -4.181979 | -3.670661 | -3.241398 |
| C | -5.512214 | -4.344685 | -2.891963 |
| C | -3.147810 | -5.190068 | -6.637959 |
| C | 1.875795 | 3.379872 | 4.127844 |
| C | 1.627666 | 4.695747 | 3.725343 |
| C | 0.548539 | 5.357086 | 4.316829 |

| | | | |
|---|---|---|---|
| C | -0.241760 | 4.743169 | 5.267170 |
| C | 0.038326 | 3.458187 | 5.678671 |
| C | 1.103565 | 2.765482 | 5.107280 |
| C | 2.176359 | -1.010265 | 6.863404 |
| C | 2.476503 | -2.229148 | 7.511103 |
| C | 2.355468 | -2.352601 | 8.867949 |
| C | 1.941933 | -1.265219 | 9.651178 |
| C | 1.633908 | -0.075843 | 9.054632 |
| C | 1.725555 | 0.074179 | 7.654998 |
| C | 1.400589 | 1.286438 | 7.025419 |
| C | 1.438441 | 1.442174 | 5.659359 |
| C | 1.846718 | 0.336372 | 4.869639 |
| C | 2.276662 | -0.836524 | 5.451340 |
| C | 0.728813 | -4.079910 | 1.275094 |
| C | -0.045959 | -4.958121 | 0.523709 |
| C | 0.108239 | -6.333110 | 0.679569 |
| C | 1.014834 | -6.830813 | 1.594278 |
| C | 1.771643 | -5.958973 | 2.349802 |
| C | 1.652663 | -4.574076 | 2.191618 |
| C | 2.519423 | -3.735652 | 3.038833 |
| C | 3.778626 | -4.203284 | 3.351464 |
| C | 4.636957 | -3.545794 | 4.248458 |
| C | 5.952252 | -3.999060 | 4.483898 |
| C | 6.769082 | -3.343323 | 5.360548 |
| C | 6.306989 | -2.200436 | 6.030271 |
| C | 5.034988 | -1.741110 | 5.829592 |
| C | 4.161798 | -2.407461 | 4.944959 |
| C | 2.851868 | -1.936841 | 4.650216 |
| C | 2.108483 | -2.527717 | 3.652825 |

SM-Si

ONIOM(PBE-D3BJ/def2-SVP: GFN2-xTB, gas phase) G = -1707.271929

ONIOM(B2PLYP-D3BJ/def2-TZVP(ALPB:n-hexane):GFN2-xTB) G = -1708.919957

Number of imaginary frequencies: 0

Coordinates:

| | | | |
|---|---|---|---|
| C | -4.558962 | 2.069866 | -4.679385 |
| C | -3.702271 | 2.977377 | -4.155409 |
| O | -2.435689 | 3.075408 | -4.658597 |
| C | -1.733609 | 4.298812 | -4.464373 |
| O | -3.930044 | 3.831247 | -3.136658 |
| Si | -5.157061 | 4.041987 | -1.937398 |
| C | -6.322751 | 5.402015 | -2.654445 |
| C | -6.991109 | 4.881904 | -3.943292 |
| C | -7.404645 | 5.746176 | -1.610491 |
| C | -5.489790 | 6.658109 | -2.976968 |
| C | -6.058008 | 2.448325 | -1.544866 |
| C | -4.215478 | 4.629701 | -0.424675 |
| H | -5.546305 | 1.902406 | -4.239354 |
| H | -4.224359 | 1.458713 | -5.525450 |
| H | -1.549095 | 4.509536 | -3.391135 |
| H | -2.283383 | 5.157909 | -4.908863 |
| H | -0.760057 | 4.178152 | -4.976432 |
| H | -7.617806 | 3.987924 | -3.747975 |
| H | -6.241780 | 4.600652 | -4.710082 |
| H | -7.651930 | 5.664157 | -4.378825 |
| H | -6.963836 | 6.140510 | -0.671789 |
| H | -8.093457 | 6.524922 | -2.007397 |
| H | -8.020739 | 4.862011 | -1.346201 |
| H | -5.012817 | 7.079234 | -2.068385 |
| H | -6.134443 | 7.450418 | -3.417911 |
| H | -4.683510 | 6.430677 | -3.702951 |
| H | -6.723548 | 2.092007 | -2.353157 |

| | | | |
|---|---|---|---|
| H | -5.327921 | 1.646547 | -1.300717 |
| H | -6.673062 | 2.609123 | -0.635697 |
| H | -3.533189 | 5.476681 | -0.633936 |
| H | -3.611849 | 3.786350 | -0.032294 |
| H | -4.917425 | 4.920758 | 0.381871 |
| H | 0.943242 | -3.580516 | -3.516719 |
| H | 4.012477 | -3.242725 | -3.476031 |
| H | 3.081458 | -4.634215 | -4.121361 |
| H | 2.551084 | -5.403387 | -1.812841 |
| C | -0.707054 | -0.262372 | -1.316613 |
| C | -1.699472 | 0.602867 | -1.963164 |
| C | -1.576894 | 2.077417 | -1.559886 |
| C | 0.057927 | -1.203633 | -1.970403 |
| N | 0.951500 | -1.905489 | -1.224668 |
| N | 1.753163 | -2.710238 | -1.774544 |
| C | 1.868554 | -3.037371 | -3.222823 |
| C | 3.134627 | -3.895782 | -3.298975 |
| C | 3.235959 | -4.534803 | -1.902968 |
| C | 2.777114 | -3.412183 | -0.975790 |
| H | 4.258323 | -4.876077 | -1.658240 |
| H | 3.556558 | -2.644651 | -0.763100 |
| H | 2.328485 | -3.707939 | -0.009162 |
| H | -0.536077 | -0.147442 | -0.226117 |
| H | -1.717397 | 0.474926 | -3.063699 |
| H | -2.690462 | 0.266836 | -1.546338 |
| H | -0.623099 | 2.508483 | -1.914135 |
| H | -2.411943 | 2.650555 | -1.991752 |
| H | -1.636536 | 2.162083 | -0.458779 |
| H | -0.046215 | -1.365897 | -3.059794 |
| H | 1.919856 | -2.098425 | -3.807828 |
| N | -0.139606 | 0.152077 | 1.948398 |

| | | | |
|---|---|---|---|
| P | -1.431578 | 0.768933 | 2.650543 |
| N | -2.548984 | 1.145809 | 1.561827 |
| S | -3.707317 | 0.158657 | 1.228880 |
| O | -4.073738 | 0.203250 | -0.162706 |
| O | -3.670647 | -1.138470 | 1.826855 |
| C | -5.144721 | 1.022800 | 2.095226 |
| F | -6.350141 | 0.646126 | 1.664342 |
| F | -5.077644 | 2.348970 | 1.950560 |
| F | -5.089854 | 0.752633 | 3.404094 |
| O | -1.050265 | 2.124334 | 3.418038 |
| O | -2.077801 | -0.191529 | 3.756920 |
| F | 6.012639 | -0.806315 | 1.493664 |
| N | 2.183964 | -1.091519 | 1.587897 |
| P | 1.142778 | -0.488909 | 2.645158 |
| S | 3.375735 | -0.270070 | 1.021392 |
| O | 1.922294 | 0.522297 | 3.616460 |
| O | 0.706710 | -1.714797 | 3.584558 |
| O | 3.497470 | 1.098985 | 1.401317 |
| O | 3.652970 | -0.611560 | -0.351989 |
| C | 4.793314 | -1.126313 | 1.933328 |
| F | 4.750165 | -0.819769 | 3.231568 |
| F | 4.683819 | -2.459060 | 1.818194 |
| H | -2.927994 | -2.504006 | 3.918925 |
| C | -2.185378 | 2.844644 | 3.822879 |
| C | -2.905965 | 2.326292 | 4.873899 |
| C | -4.175683 | 2.874159 | 5.214369 |
| C | -5.034904 | 2.286952 | 6.166202 |
| C | -6.269062 | 2.821126 | 6.414253 |
| C | -6.705785 | 3.963598 | 5.727140 |
| C | -5.903666 | 4.542149 | 4.784936 |
| C | -4.628217 | 4.010323 | 4.499537 |

| | | | |
|---|---|---|---|
| C | -3.788711 | 4.587074 | 3.532022 |
| C | -2.573095 | 4.044768 | 3.175651 |
| C | -1.720708 | 4.785329 | 2.231579 |
| C | -1.756576 | 6.182067 | 2.259760 |
| C | -0.963705 | 6.923199 | 1.405686 |
| C | -0.120112 | 6.291351 | 0.512320 |
| C | -0.068129 | 4.900596 | 0.461896 |
| C | -0.872055 | 4.161891 | 1.327111 |
| H | -0.855774 | 3.086090 | 1.278412 |
| H | -0.221609 | 6.418058 | -7.045557 |
| H | -8.840261 | -2.605745 | 1.329262 |
| H | 0.173644 | -2.716440 | 6.833589 |
| H | 0.521848 | 6.878048 | -0.126568 |
| H | -0.994073 | 8.003134 | 1.447349 |
| H | -0.663757 | -6.109480 | 4.453248 |
| H | 0.643930 | -5.038011 | 6.177930 |
| H | -2.379151 | 6.693756 | 2.977680 |
| H | -4.140857 | 5.471426 | 3.023901 |
| H | -6.235810 | 5.410032 | 4.233919 |
| H | -7.683292 | 4.370933 | 5.939175 |
| H | -6.921816 | 2.356428 | 7.138108 |
| H | -4.720875 | 1.393290 | 6.681775 |
| C | -2.360457 | 1.216421 | 5.682404 |
| C | -2.028835 | -0.001368 | 5.128458 |
| C | -1.699063 | -1.116901 | 5.940552 |
| C | -1.650077 | -0.929118 | 7.303615 |
| C | -1.846560 | 0.325850 | 7.901458 |
| C | -1.709433 | 0.510478 | 9.293239 |
| C | -1.879355 | 1.744631 | 9.853069 |
| C | -2.193659 | 2.844028 | 9.040725 |
| C | -2.359540 | 2.689673 | 7.691870 |

| | | | |
|---|---|---|---|
| C | -2.202881 | 1.425193 | 7.083714 |
| H | -2.606856 | 3.544664 | 7.081339 |
| H | -2.307298 | 3.820021 | 9.489169 |
| H | -1.768075 | 1.883900 | 10.918223 |
| H | -1.455374 | -0.344151 | 9.902667 |
| H | -1.453897 | -1.771735 | 7.949522 |
| C | -1.421769 | -2.468732 | 5.422049 |
| C | -0.412009 | -3.186794 | 6.060852 |
| C | -0.148867 | -4.487117 | 5.691213 |
| C | -0.899502 | -5.089807 | 4.703782 |
| C | -1.918251 | -4.404328 | 4.036136 |
| C | -2.155519 | -3.076130 | 4.406842 |
| C | 2.940467 | 4.594219 | -6.734298 |
| C | 4.213095 | 1.738966 | -3.478728 |
| C | 2.775965 | 2.192845 | -3.714903 |
| C | 1.918087 | 1.005643 | -4.172107 |
| C | 2.162359 | 2.801025 | -2.475086 |
| C | 2.016578 | 2.250914 | -1.219747 |
| C | 1.332752 | 2.966157 | -0.248216 |
| C | 0.775727 | 4.216054 | -0.525288 |
| C | 0.964676 | 4.785710 | -1.785138 |
| C | 1.659603 | 4.080380 | -2.749000 |
| C | 1.988771 | 4.416447 | -4.125320 |
| C | 2.672995 | 3.336914 | -4.695939 |
| C | 3.141333 | 3.432757 | -5.982176 |
| C | 3.528089 | 4.637082 | -8.133577 |
| C | 2.716307 | 3.701708 | -9.041148 |
| C | 4.992778 | 4.171406 | -8.121127 |
| C | 3.508711 | 6.066040 | -8.687957 |
| C | 2.178747 | 6.755571 | -8.441007 |
| C | 1.887430 | 6.923766 | -6.945534 |

| | | | |
|---|---|---|---|
| C | 2.729918 | 8.067426 | -6.363093 |
| C | 0.398825 | 7.279126 | -6.805718 |
| C | 2.204623 | 5.648212 | -6.184979 |
| C | 1.749918 | 5.552282 | -4.870295 |
| H | 4.235494 | 1.004500 | -2.678069 |
| H | 4.626587 | 1.294698 | -4.383166 |
| H | 4.829396 | 2.584826 | -3.187509 |
| H | 1.931317 | 0.245751 | -3.394050 |
| H | 0.889782 | 1.321358 | -4.339108 |
| H | 2.310950 | 0.587697 | -5.097674 |
| H | 2.419425 | 1.280048 | -0.978628 |
| H | 1.226310 | 2.531349 | 0.733742 |
| H | 1.209502 | 6.371446 | -4.422687 |
| H | 0.536710 | 5.748672 | -2.021057 |
| H | 3.677830 | 2.602551 | -6.415166 |
| H | 3.125452 | 3.705407 | -10.049607 |
| H | 2.744490 | 2.683390 | -8.661100 |
| H | 1.675176 | 4.010612 | -9.086934 |
| H | 5.078831 | 3.102760 | -7.947712 |
| H | 5.453798 | 4.389764 | -9.082578 |
| H | 5.550377 | 4.689721 | -7.344223 |
| H | 3.715921 | 6.037723 | -9.760340 |
| H | 4.307866 | 6.640160 | -8.215577 |
| H | 1.377361 | 6.170512 | -8.895793 |
| H | 2.178456 | 7.737807 | -8.919403 |
| H | 2.505688 | 9.001014 | -6.875457 |
| H | 2.519649 | 8.194133 | -5.304115 |
| H | 3.791654 | 7.859755 | -6.468804 |
| H | 0.150626 | 8.086382 | -7.492235 |
| H | 0.152439 | 7.608948 | -5.800862 |
| C | -7.541401 | -4.483951 | -1.448157 |

| | | | |
|---|---|---|---|
| C | -3.951037 | -7.260064 | -1.414949 |
| C | -4.867066 | -6.967167 | -0.219032 |
| C | -5.687677 | -8.204279 | 0.147857 |
| C | -4.048908 | -6.455111 | 0.941650 |
| C | -3.180663 | -7.142658 | 1.756669 |
| C | -2.539330 | -6.464653 | 2.778338 |
| C | -2.696940 | -5.088820 | 2.982523 |
| C | -3.607131 | -4.407196 | 2.166047 |
| C | -4.289796 | -5.093935 | 1.175650 |
| C | -5.362144 | -4.673557 | 0.281613 |
| C | -5.740231 | -5.768792 | -0.505045 |
| C | -6.800621 | -5.665320 | -1.369057 |
| C | -8.738769 | -4.457243 | -2.380948 |
| C | -9.883685 | -5.242936 | -1.724596 |
| C | -8.419144 | -5.105994 | -3.736320 |
| C | -9.155527 | -3.009024 | -2.654535 |
| C | -9.304528 | -2.216399 | -1.368876 |
| C | -7.981612 | -2.103396 | -0.597735 |
| C | -7.138706 | -0.969221 | -1.194752 |
| C | -8.332777 | -1.765245 | 0.858561 |
| C | -7.188007 | -3.398053 | -0.640538 |
| C | -6.070276 | -3.492522 | 0.192864 |
| H | -3.274530 | -8.079726 | -1.180832 |
| H | -4.542065 | -7.534624 | -2.286387 |
| H | -3.361245 | -6.377738 | -1.657286 |
| H | -5.033268 | -9.031179 | 0.414403 |
| H | -6.334267 | -7.985408 | 0.994760 |
| H | -6.306801 | -8.511888 | -0.691831 |
| H | -3.006686 | -8.201300 | 1.623158 |
| H | -1.908190 | -7.046987 | 3.428658 |
| H | -5.759809 | -2.650294 | 0.791539 |

| | | | |
|---|---|---|---|
| H | -3.785371 | -3.351121 | 2.285165 |
| H | -7.080754 | -6.515564 | -1.971462 |
| H | -10.777036 | -5.201817 | -2.345070 |
| H | -9.598672 | -6.284824 | -1.597153 |
| H | -10.121109 | -4.843206 | -0.742628 |
| H | -8.318141 | -6.184743 | -3.658011 |
| H | -9.228008 | -4.901464 | -4.435607 |
| H | -7.497275 | -4.701404 | -4.145389 |
| H | -10.099596 | -3.001251 | -3.205109 |
| H | -8.398666 | -2.540691 | -3.287264 |
| H | -10.053537 | -2.696282 | -0.737411 |
| H | -9.668640 | -1.211584 | -1.594701 |
| H | -7.652427 | -0.020098 | -1.064319 |
| H | -6.170357 | -0.892161 | -0.708511 |
| H | -6.973992 | -1.130166 | -2.258351 |
| H | -8.991966 | -0.900181 | 0.886008 |
| H | -7.449363 | -1.528394 | 1.439515 |
| H | 7.297125 | 1.245161 | 0.940484 |
| H | 8.792297 | 0.510012 | 0.381924 |
| H | 6.533907 | 0.114764 | -2.549195 |
| H | 5.810679 | 0.254492 | -0.950093 |
| H | 7.265221 | -0.712229 | -1.170638 |
| H | 9.250248 | 0.338451 | -2.144214 |
| H | 9.664417 | 1.983184 | -1.677151 |
| H | 7.744506 | 1.271196 | -3.928149 |
| H | 9.439132 | 1.700016 | -4.161029 |
| H | 6.595657 | 3.167825 | -4.989879 |
| H | 8.217956 | 3.284301 | -5.683196 |
| H | 7.430620 | 4.717603 | -5.037140 |
| H | 9.897206 | 4.019815 | -2.364526 |
| H | 9.172137 | 5.263749 | -3.369938 |

| | | | |
|---|---|---|---|
| H | 10.162089 | 4.005379 | -4.111631 |
| H | 6.859749 | 5.616508 | -3.155058 |
| H | 3.586355 | 3.290924 | 1.627899 |
| H | 5.518774 | 2.278150 | 0.219155 |
| H | 2.236920 | 7.269378 | 2.540706 |
| H | 3.383833 | 8.136777 | 0.594963 |
| H | 6.664555 | 7.785188 | -1.790390 |
| H | 6.676194 | 7.317482 | -0.086539 |
| H | 5.557307 | 8.561936 | -0.654462 |
| H | 3.349869 | 6.193896 | -2.556618 |
| H | 4.679131 | 7.113007 | -3.265961 |
| H | 3.577728 | 7.895338 | -2.125064 |
| H | 1.559813 | -6.502261 | -5.660073 |
| H | 1.976289 | -6.685369 | -7.355980 |
| H | -1.470604 | -8.210478 | -6.909776 |
| H | -0.328555 | -8.095158 | -5.580426 |
| H | 0.228478 | -8.616732 | -7.173120 |
| H | 0.214082 | -7.092238 | -9.062120 |
| H | 0.186338 | -5.348216 | -8.821361 |
| H | -2.248131 | -7.170846 | -8.664655 |
| H | -1.820742 | -6.175965 | -10.052080 |
| H | -4.345589 | -6.121136 | -7.887975 |
| H | -4.199937 | -5.602196 | -9.570039 |
| H | -4.604260 | -4.432122 | -8.324470 |
| H | -1.065190 | -3.552412 | -8.967188 |
| H | -2.666924 | -2.912885 | -8.630422 |
| H | -2.379540 | -3.820279 | -10.116866 |
| H | -3.788990 | -3.534940 | -6.570919 |
| H | -0.114916 | -5.451069 | -1.895202 |
| H | -0.085054 | -5.960226 | -4.408979 |
| H | -2.028095 | -2.680976 | 0.746638 |

| | | | |
|---|---|---|---|
| H | -3.543607 | -1.976504 | -1.033540 |
| H | -4.129190 | -1.447042 | -5.065162 |
| H | -2.508940 | -1.209291 | -4.414496 |
| H | -3.924005 | -0.949650 | -3.385128 |
| H | -4.888320 | -4.600981 | -3.267399 |
| H | -5.474559 | -3.435393 | -4.463573 |
| H | -5.384127 | -2.992939 | -2.749278 |
| H | 1.233939 | 2.648073 | 7.553548 |
| H | 1.220706 | 1.501740 | 9.677284 |
| H | 1.542427 | -0.560378 | 10.995434 |
| H | 2.110311 | -2.671102 | 9.852299 |
| H | 2.404468 | -2.735301 | 7.431563 |
| H | 4.472468 | -0.846317 | 6.721272 |
| H | 6.634630 | -1.864789 | 7.233790 |
| H | 7.284378 | -4.012656 | 6.213478 |
| H | 5.769237 | -5.123706 | 4.615164 |
| H | 3.693277 | -5.159934 | 3.390712 |
| H | 1.874985 | -6.288065 | 3.472870 |
| H | -0.675346 | 5.732677 | 5.261170 |
| H | 0.726602 | 6.493324 | 3.441286 |
| H | 0.630454 | -7.708670 | 1.917265 |
| H | -0.701717 | -6.683442 | 0.110320 |
| H | -0.336341 | 3.502642 | 6.245556 |
| H | 8.663991 | 2.269625 | 0.502136 |
| H | 1.499081 | -5.101659 | -6.736456 |
| H | 0.519024 | -2.797487 | 1.392557 |
| H | 2.875951 | 2.788249 | 3.528138 |
| C | 5.835976 | 3.005382 | -0.511780 |
| C | 6.862848 | 2.706347 | -1.412010 |
| C | 8.124136 | 1.358648 | 0.249169 |
| C | 6.752391 | 0.194044 | -1.487183 |

| | | | |
|---|---|---|---|
| C | 7.636205 | 1.414468 | -1.206522 |
| C | 8.876583 | 1.364379 | -2.109603 |
| C | 8.571168 | 1.851976 | -3.514603 |
| C | 7.565736 | 3.649580 | -4.891623 |
| C | 9.433490 | 4.209342 | -3.328963 |
| C | 8.186639 | 3.334645 | -3.520785 |
| C | 6.576889 | 4.882575 | -2.415658 |
| C | 5.649249 | 5.208353 | -1.459546 |
| C | 5.231949 | 4.247466 | -0.528779 |
| C | 4.267542 | 4.868484 | 0.371892 |
| C | 3.544479 | 4.350159 | 1.434127 |
| C | 2.757989 | 5.203899 | 2.215672 |
| C | 2.761580 | 6.569711 | 1.910209 |
| C | 3.434068 | 7.079016 | 0.813342 |
| C | 4.173583 | 6.224913 | 0.027993 |
| C | 6.040198 | 7.622677 | -0.914688 |
| C | 4.992086 | 6.542491 | -1.200071 |
| C | 4.090110 | 6.965263 | -2.363292 |
| C | 7.186527 | 3.625577 | -2.415949 |
| C | -0.886668 | -5.424973 | -4.895366 |
| C | -1.099929 | -5.579962 | -6.264434 |
| C | 1.311176 | -6.171470 | -6.664795 |
| C | -0.449289 | -7.944621 | -6.650539 |
| C | -0.149588 | -6.486814 | -7.026710 |
| C | -0.300384 | -6.283431 | -8.538147 |
| C | -1.757302 | -6.243044 | -8.963670 |
| C | -4.009739 | -5.315554 | -8.537611 |
| C | -2.129330 | -3.754525 | -9.060018 |
| C | -2.507224 | -5.058844 | -8.342926 |
| C | -2.976013 | -4.066973 | -6.102618 |
| C | -2.741447 | -3.893689 | -4.761326 |

| | | | |
|---|---|---|---|
| C | -1.695891 | -4.593695 | -4.148871 |
| C | -1.661796 | -4.230164 | -2.742539 |
| C | -0.851245 | -4.678721 | -1.717904 |
| C | -0.992568 | -4.127232 | -0.445026 |
| C | -1.935358 | -3.126599 | -0.230622 |
| C | -2.789957 | -2.717715 | -1.246463 |
| C | -2.658052 | -3.271220 | -2.502697 |
| C | -3.509534 | -1.564663 | -4.179327 |
| C | -3.473060 | -3.033205 | -3.755473 |
| C | -4.900241 | -3.549829 | -3.546387 |
| C | -2.173777 | -4.918566 | -6.868591 |
| C | 2.100409 | 3.441465 | 3.895446 |
| C | 1.926368 | 4.708291 | 3.330846 |
| C | 0.916478 | 5.518125 | 3.855173 |
| C | 0.109209 | 5.089156 | 4.888830 |
| C | 0.300715 | 3.843391 | 5.444144 |
| C | 1.304488 | 3.009596 | 4.952297 |
| C | 1.968570 | -0.642717 | 7.130475 |
| C | 2.140607 | -1.806324 | 7.913048 |
| C | 1.980875 | -1.769961 | 9.271029 |
| C | 1.653368 | -0.571147 | 9.921282 |
| C | 1.477077 | 0.570822 | 9.193668 |
| C | 1.616554 | 0.560498 | 7.789632 |
| C | 1.435013 | 1.726126 | 7.028769 |
| C | 1.520184 | 1.733849 | 5.655139 |
| C | 1.832225 | 0.515640 | 5.000999 |
| C | 2.111653 | -0.632085 | 5.712307 |
| C | 0.537989 | -3.868408 | 1.528530 |
| C | -0.181405 | -4.678326 | 0.651994 |
| C | -0.129400 | -6.062931 | 0.783341 |
| C | 0.612089 | -6.634921 | 1.797989 |

| | | | |
|---|---|---|---|
| C | 1.323440 | -5.831526 | 2.665379 |
| C | 1.316909 | -4.438988 | 2.532393 |
| C | 2.170052 | -3.675003 | 3.460116 |
| C | 3.366378 | -4.239018 | 3.849134 |
| C | 4.230191 | -3.633712 | 4.776469 |
| C | 5.483869 | -4.198436 | 5.094489 |
| C | 6.323828 | -3.579893 | 5.976015 |
| C | 5.949751 | -2.361691 | 6.562960 |
| C | 4.737160 | -1.795368 | 6.283056 |
| C | 3.834799 | -2.425232 | 5.400163 |
| C | 2.584427 | -1.853964 | 5.030066 |
| C | 1.824957 | -2.428860 | 4.035556 |

TS-Si

ONIOM(PBE-D3BJ/def2-SVP: GFN2-xTB, gas phase) G = -1707.247190

ONIOM(B2PLYP-D3BJ/def2-TZVP(ALPB:n-hexane):GFN2-xTB) G = -1708.886422

Activation energy: ΔG‡ = 21.0 kcal/mol

Number of imaginary frequencies: 1 (-198.49 cm-1)

Coordinates:

| | | | |
|---|---|---|---|
| C | -1.410844 | 0.912672 | -4.579720 |
| C | -1.640584 | 2.276064 | -4.693763 |
| O | -0.588577 | 3.090408 | -4.877216 |
| C | -0.822799 | 4.502570 | -4.928299 |
| O | -2.801866 | 2.891658 | -4.576753 |
| Si | -4.498906 | 2.456491 | -4.471153 |
| C | -5.143222 | 2.373588 | -6.421082 |
| C | -4.429574 | 1.204689 | -7.126538 |
| C | -6.666727 | 2.137891 | -6.429710 |
| C | -4.813513 | 3.693275 | -7.141813 |
| C | -4.833354 | 0.875144 | -3.521385 |
| C | -5.187983 | 3.940106 | -3.552485 |

| | | | |
|---|---|---|---|
| H | -2.290122 | 0.267091 | -4.507979 |
| H | -0.502313 | 0.507489 | -5.033763 |
| H | -1.305967 | 4.854602 | -3.995137 |
| H | -1.468823 | 4.766690 | -5.790750 |
| H | 0.173827 | 4.967922 | -5.035337 |
| H | -4.661128 | 0.228596 | -6.653008 |
| H | -3.326410 | 1.325201 | -7.124378 |
| H | -4.753740 | 1.139916 | -8.188895 |
| H | -7.218433 | 2.964803 | -5.936463 |
| H | -7.038784 | 2.071367 | -7.475769 |
| H | -6.945034 | 1.192043 | -5.920772 |
| H | -5.321493 | 4.562026 | -6.674725 |
| H | -5.145228 | 3.646483 | -8.202465 |
| H | -3.723386 | 3.899168 | -7.143145 |
| H | -4.546965 | -0.045117 | -4.066355 |
| H | -4.375973 | 0.847869 | -2.510709 |
| H | -5.931671 | 0.832517 | -3.358406 |
| H | -4.887480 | 4.890355 | -4.036477 |
| H | -4.814910 | 3.946725 | -2.508519 |
| H | -6.295472 | 3.900642 | -3.516588 |
| H | 1.826157 | -2.402632 | -0.925286 |
| H | 2.573637 | -4.778956 | -2.745541 |
| H | 3.165584 | -4.426621 | -1.092719 |
| H | 4.586921 | -2.588398 | -1.894767 |
| C | -0.299040 | 0.683372 | -2.567902 |
| C | -1.325510 | 1.015896 | -1.547281 |
| C | -2.022519 | 2.363724 | -1.637516 |
| C | 0.270414 | -0.606123 | -2.517229 |
| N | 1.407671 | -0.856147 | -3.173712 |
| N | 2.059721 | -1.940957 | -2.953234 |
| C | 1.778033 | -2.935898 | -1.900145 |

| | | | |
|---|---|---|---|
| C | 2.898829 | -3.966481 | -2.062537 |
| C | 4.057944 | -3.160539 | -2.679779 |
| C | 3.353213 | -2.176753 | -3.616487 |
| H | 4.793663 | -3.794220 | -3.210642 |
| H | 3.169502 | -2.611108 | -4.625089 |
| H | 3.874587 | -1.209097 | -3.736936 |
| H | 0.318155 | 1.491445 | -2.991265 |
| H | -2.075684 | 0.201858 | -1.468869 |
| H | -0.788975 | 0.937902 | -0.572043 |
| H | -2.725862 | 2.391739 | -2.485934 |
| H | -2.608109 | 2.542617 | -0.717037 |
| H | -1.302852 | 3.196994 | -1.770752 |
| H | -0.195031 | -1.367229 | -1.861179 |
| H | 0.760214 | -3.360495 | -2.020860 |
| N | -0.203685 | -0.191251 | 2.014693 |
| P | -1.577526 | 0.421610 | 2.510643 |
| N | -2.603686 | 0.690452 | 1.297519 |
| S | -3.976663 | 0.000340 | 0.887272 |
| O | -4.059053 | -0.153339 | -0.593928 |
| O | -4.423560 | -1.140093 | 1.714746 |
| C | -5.232128 | 1.363285 | 1.221226 |
| F | -6.373809 | 1.106020 | 0.565893 |
| F | -4.774153 | 2.562052 | 0.806556 |
| F | -5.499568 | 1.424093 | 2.535180 |
| O | -1.214508 | 1.849922 | 3.238204 |
| O | -2.355380 | -0.487673 | 3.621814 |
| F | 5.855965 | -1.761661 | 0.324783 |
| N | 2.145194 | -0.978582 | 1.125609 |
| P | 1.260385 | -0.632140 | 2.431824 |
| S | 3.546582 | -0.444725 | 0.591052 |
| O | 2.080348 | 0.469770 | 3.322799 |

| | | | |
|---|---|---|---|
| O | 1.081677 | -1.961199 | 3.382043 |
| O | 4.095226 | 0.760904 | 1.247740 |
| O | 3.561913 | -0.508648 | -0.894657 |
| C | 4.740147 | -1.824552 | 1.070183 |
| F | 5.085884 | -1.706988 | 2.360844 |
| F | 4.182542 | -3.037716 | 0.866865 |
| H | -2.965331 | -2.773713 | 3.883560 |
| C | -2.391325 | 2.534055 | 3.637360 |
| C | -3.079674 | 2.018278 | 4.711775 |
| C | -4.357074 | 2.534967 | 5.060703 |
| C | -5.174740 | 1.952579 | 6.050450 |
| C | -6.420086 | 2.454788 | 6.308063 |
| C | -6.907295 | 3.559328 | 5.593381 |
| C | -6.144447 | 4.133960 | 4.617002 |
| C | -4.857750 | 3.636123 | 4.322411 |
| C | -4.051467 | 4.213663 | 3.327457 |
| C | -2.815657 | 3.711582 | 2.975322 |
| C | -1.968096 | 4.500331 | 2.062586 |
| C | -2.065462 | 5.891662 | 2.160569 |
| C | -1.273087 | 6.709946 | 1.382563 |
| C | -0.365328 | 6.158304 | 0.500798 |
| C | -0.256311 | 4.774649 | 0.366461 |
| C | -1.063787 | 3.955350 | 1.157167 |
| H | -1.006749 | 2.883697 | 1.054023 |
| H | 0.539672 | 7.353240 | -6.965751 |
| H | -8.529643 | -2.425669 | 1.471725 |
| H | 0.430079 | -2.809775 | 6.464575 |
| H | 0.287059 | 6.807684 | -0.062063 |
| H | -1.346556 | 7.783942 | 1.482585 |
| H | -0.327835 | -6.144930 | 3.959579 |
| H | 1.025451 | -5.055847 | 5.650104 |

| | | | |
|---|---|---|---|
| H | -2.736321 | 6.338308 | 2.878196 |
| H | -4.440369 | 5.074257 | 2.803969 |
| H | -6.516181 | 4.975297 | 4.049758 |
| H | -7.893946 | 3.939869 | 5.811390 |
| H | -7.041835 | 1.995894 | 7.062400 |
| H | -4.817185 | 1.088989 | 6.589559 |
| C | -2.469589 | 0.952328 | 5.532366 |
| C | -2.131210 | -0.262847 | 4.984386 |
| C | -1.646210 | -1.331827 | 5.775680 |
| C | -1.506239 | -1.119304 | 7.128091 |
| C | -1.782381 | 0.121762 | 7.725543 |
| C | -1.618084 | 0.325522 | 9.111915 |
| C | -1.859744 | 1.547606 | 9.672263 |
| C | -2.268268 | 2.620332 | 8.866048 |
| C | -2.463853 | 2.446594 | 7.523675 |
| C | -2.247040 | 1.190033 | 6.918496 |
| H | -2.789401 | 3.277501 | 6.916114 |
| H | -2.432693 | 3.589190 | 9.314299 |
| H | -1.732203 | 1.698367 | 10.734164 |
| H | -1.289422 | -0.506067 | 9.717520 |
| H | -1.185017 | -1.931180 | 7.763638 |
| C | -1.305762 | -2.647754 | 5.214027 |
| C | -0.184029 | -3.298436 | 5.724458 |
| C | 0.150263 | -4.553044 | 5.262815 |
| C | -0.628258 | -5.170817 | 4.305037 |
| C | -1.760720 | -4.550237 | 3.775204 |
| C | -2.083498 | -3.276290 | 4.247154 |
| C | 3.770141 | 5.797171 | -6.219166 |
| C | 4.703078 | 2.450257 | -3.343583 |
| C | 3.293722 | 2.866747 | -3.749555 |
| C | 2.642079 | 1.760009 | -4.589364 |

| | | | |
|---|---|---|---|
| C | 2.430512 | 3.204323 | -2.553145 |
| C | 2.171675 | 2.472961 | -1.411747 |
| C | 1.316634 | 2.994845 | -0.448815 |
| C | 0.686109 | 4.228028 | -0.624110 |
| C | 0.960807 | 4.964723 | -1.779049 |
| C | 1.841342 | 4.465744 | -2.716832 |
| C | 2.355831 | 5.059134 | -3.939751 |
| C | 3.258758 | 4.163316 | -4.522367 |
| C | 3.955242 | 4.536014 | -5.644543 |
| C | 4.636633 | 6.161032 | -7.411245 |
| C | 4.187569 | 5.340762 | -8.628580 |
| C | 6.111587 | 5.846406 | -7.113884 |
| C | 4.541665 | 7.660655 | -7.715603 |
| C | 3.108213 | 8.159518 | -7.677670 |
| C | 2.490291 | 8.019532 | -6.282001 |
| C | 3.029221 | 9.111660 | -5.347398 |
| C | 0.971380 | 8.198811 | -6.433633 |
| C | 2.810371 | 6.661051 | -5.683545 |
| C | 2.123564 | 6.285056 | -4.529735 |
| H | 4.648352 | 1.597594 | -2.673578 |
| H | 5.285718 | 2.175485 | -4.221160 |
| H | 5.207286 | 3.264422 | -2.832780 |
| H | 2.502167 | 0.864586 | -3.989531 |
| H | 1.672459 | 2.081600 | -4.963951 |
| H | 3.275719 | 1.523103 | -5.442194 |
| H | 2.627118 | 1.508494 | -1.249198 |
| H | 1.146364 | 2.430214 | 0.456954 |
| H | 1.414513 | 6.962368 | -4.078670 |
| H | 0.477870 | 5.914713 | -1.953010 |
| H | 4.665005 | 3.849972 | -6.079524 |
| H | 4.815247 | 5.567263 | -9.488270 |

| | | | |
|---|---|---|---|
| H | 4.260714 | 4.276418 | -8.419170 |
| H | 3.154224 | 5.559309 | -8.885954 |
| H | 6.304908 | 4.777938 | -7.099545 |
| H | 6.742826 | 6.288815 | -7.882230 |
| H | 6.399464 | 6.258267 | -6.149020 |
| H | 4.974597 | 7.855410 | -8.699558 |
| H | 5.134612 | 8.210081 | -6.982309 |
| H | 2.508159 | 7.592305 | -8.391790 |
| H | 3.073688 | 9.208143 | -7.982396 |
| H | 2.811558 | 10.098069 | -5.752396 |
| H | 2.569806 | 9.032846 | -4.365421 |
| H | 4.104180 | 9.016599 | -5.219145 |
| H | 0.766361 | 9.104293 | -7.001635 |
| H | 0.474065 | 8.289028 | -5.472385 |
| C | -7.663447 | -4.612551 | -1.376883 |
| C | -4.336713 | -7.712859 | -1.365072 |
| C | -5.084425 | -7.193189 | -0.133568 |
| C | -5.972461 | -8.292452 | 0.457372 |
| C | -4.122674 | -6.648934 | 0.896800 |
| C | -3.203172 | -7.327118 | 1.663646 |
| C | -2.467465 | -6.627824 | 2.605082 |
| C | -2.588321 | -5.244077 | 2.770053 |
| C | -3.518185 | -4.561035 | 1.978866 |
| C | -4.293786 | -5.268954 | 1.076749 |
| C | -5.411344 | -4.844719 | 0.244990 |
| C | -5.891122 | -5.957338 | -0.456938 |
| C | -6.985238 | -5.831080 | -1.275275 |
| C | -8.872858 | -4.567965 | -2.295521 |
| C | -9.986875 | -5.440997 | -1.699749 |
| C | -8.503735 | -5.108958 | -3.686268 |
| C | -9.380063 | -3.132834 | -2.476595 |

| | | | |
|---|---|---|---|
| C | -9.377581 | -2.363296 | -1.167446 |
| C | -7.959498 | -2.191425 | -0.609208 |
| C | -7.183463 | -1.165249 | -1.443559 |
| C | -8.086614 | -1.667511 | 0.828377 |
| C | -7.221706 | -3.517905 | -0.626762 |
| C | -6.066615 | -3.633802 | 0.148929 |
| H | -3.713405 | -8.563848 | -1.098397 |
| H | -5.040675 | -8.024792 | -2.133703 |
| H | -3.703011 | -6.931041 | -1.775060 |
| H | -5.371305 | -9.148337 | 0.756049 |
| H | -6.501102 | -7.916625 | 1.330697 |
| H | -6.704494 | -8.622499 | -0.276408 |
| H | -3.069288 | -8.395202 | 1.561107 |
| H | -1.802500 | -7.192432 | 3.239006 |
| H | -5.681626 | -2.787518 | 0.692077 |
| H | -3.651287 | -3.493417 | 2.060215 |
| H | -7.336933 | -6.689188 | -1.828189 |
| H | -10.854216 | -5.447861 | -2.357709 |
| H | -9.647049 | -6.465251 | -1.569043 |
| H | -10.292030 | -5.064333 | -0.726910 |
| H | -8.325988 | -6.180423 | -3.669920 |
| H | -9.317099 | -4.916713 | -4.384029 |
| H | -7.604957 | -4.619967 | -4.056051 |
| H | -10.391351 | -3.159880 | -2.890579 |
| H | -8.745513 | -2.617852 | -3.199523 |
| H | -9.988019 | -2.893481 | -0.433754 |
| H | -9.826341 | -1.378480 | -1.317377 |
| H | -7.700906 | -0.208396 | -1.429507 |
| H | -6.186872 | -1.016878 | -1.038897 |
| H | -7.086301 | -1.499647 | -2.473062 |
| H | -8.723336 | -0.784981 | 0.840289 |

| | | | |
|---|---|---|---|
| H | -7.121742 | -1.388342 | 1.238895 |
| H | 6.827595 | 0.663121 | 1.024883 |
| H | 8.352517 | -0.164697 | 0.740633 |
| H | 6.932503 | 0.504561 | -2.685157 |
| H | 5.938738 | 0.239556 | -1.255616 |
| H | 7.351230 | -0.775369 | -1.539964 |
| H | 9.605470 | 0.127430 | -1.389144 |
| H | 9.919387 | 1.680501 | -0.619139 |
| H | 8.760933 | 1.321861 | -3.411501 |
| H | 10.445770 | 1.693868 | -3.065785 |
| H | 7.891822 | 3.360429 | -4.471742 |
| H | 9.636955 | 3.422013 | -4.750379 |
| H | 8.784140 | 4.853051 | -4.189616 |
| H | 10.501295 | 3.773602 | -1.078527 |
| H | 10.055051 | 5.164247 | -2.055714 |
| H | 11.158342 | 3.951777 | -2.709274 |
| H | 7.796490 | 5.631716 | -2.450848 |
| H | 3.655203 | 3.246191 | 1.537528 |
| H | 5.611567 | 2.197667 | 0.331603 |
| H | 2.100060 | 7.199482 | 2.212705 |
| H | 3.549297 | 8.092193 | 0.493427 |
| H | 7.310867 | 7.772931 | -1.099828 |
| H | 6.939832 | 7.258535 | 0.549561 |
| H | 5.984469 | 8.533721 | -0.215211 |
| H | 4.239287 | 6.253518 | -2.637771 |
| H | 5.700176 | 7.176276 | -2.996015 |
| H | 4.373347 | 7.934324 | -2.108752 |
| H | -0.066232 | -8.956570 | -4.823982 |
| H | -0.205847 | -9.736443 | -6.391834 |
| H | -3.692023 | -9.705848 | -4.895094 |
| H | -2.246672 | -9.685085 | -3.898731 |

| | | | |
|---|---|---|---|
| H | -2.335121 | -10.766767 | -5.292636 |
| H | -2.402510 | -9.885324 | -7.554306 |
| H | -1.844934 | -8.253315 | -7.910011 |
| H | -4.571926 | -8.949793 | -6.748289 |
| H | -4.262910 | -8.576671 | -8.441010 |
| H | -5.968446 | -7.038789 | -6.030683 |
| H | -6.139604 | -7.071271 | -7.788708 |
| H | -5.825189 | -5.552093 | -6.965991 |
| H | -2.494992 | -6.226244 | -8.436916 |
| H | -3.680989 | -4.981552 | -8.073374 |
| H | -4.088640 | -6.280006 | -9.198092 |
| H | -4.343780 | -4.605428 | -5.760034 |
| H | -0.260963 | -6.444467 | -1.400452 |
| H | -1.086927 | -7.575445 | -3.557816 |
| H | -1.135836 | -2.917628 | 0.876734 |
| H | -2.709130 | -2.162701 | -0.842750 |
| H | -3.103563 | -2.227628 | -5.013044 |
| H | -1.525694 | -2.643334 | -4.342457 |
| H | -2.577928 | -1.530729 | -3.461201 |
| H | -5.115486 | -4.125876 | -2.505776 |
| H | -5.209195 | -3.056604 | -3.909129 |
| H | -4.703772 | -2.416824 | -2.337194 |
| H | 0.993310 | 2.544889 | 7.228365 |
| H | 1.108458 | 1.434360 | 9.373659 |
| H | 1.647707 | -0.564659 | 10.717352 |
| H | 2.435864 | -2.618646 | 9.602447 |
| H | 2.769907 | -2.671239 | 7.183850 |
| H | 4.686752 | -0.418387 | 6.444986 |
| H | 6.938896 | -1.140581 | 7.079237 |
| H | 7.856444 | -3.267452 | 6.238288 |
| H | 6.523083 | -4.666431 | 4.707264 |

| | | | |
|---|---|---|---|
| H | 4.461557 | -5.063663 | 3.491291 |
| H | 2.773152 | -6.385690 | 3.630003 |
| H | -0.868356 | 5.598786 | 4.893771 |
| H | 0.619008 | 6.423202 | 3.173779 |
| H | 1.473035 | -8.011849 | 2.347245 |
| H | -0.136983 | -7.244100 | 0.653014 |
| H | -0.551871 | 3.353459 | 5.851016 |
| H | 8.347409 | 1.529316 | 1.247491 |
| H | -0.005911 | -7.984038 | -6.294158 |
| H | 1.077408 | -3.203942 | 1.331735 |
| H | 2.810300 | 2.745783 | 3.302216 |
| C | 6.113927 | 2.940268 | -0.264454 |
| C | 7.266911 | 2.618225 | -0.982253 |
| C | 7.834296 | 0.787018 | 0.638700 |
| C | 6.958030 | 0.234979 | -1.632507 |
| C | 7.828503 | 1.215317 | -0.835843 |
| C | 9.276673 | 1.168065 | -1.337775 |
| C | 9.422989 | 1.819860 | -2.701518 |
| C | 8.830977 | 3.771042 | -4.107097 |
| C | 10.275198 | 4.100277 | -2.090318 |
| C | 9.085515 | 3.314865 | -2.661369 |
| C | 7.328233 | 4.875172 | -1.839296 |
| C | 6.225824 | 5.200534 | -1.089612 |
| C | 5.600609 | 4.220506 | -0.309229 |
| C | 4.499350 | 4.838320 | 0.413904 |
| C | 3.624490 | 4.306669 | 1.344107 |
| C | 2.734622 | 5.151518 | 2.015997 |
| C | 2.743764 | 6.511606 | 1.688344 |
| C | 3.582965 | 7.035138 | 0.718883 |
| C | 4.473789 | 6.200260 | 0.083540 |
| C | 6.506319 | 7.595381 | -0.389585 |

| | | | |
|---|---|---|---|
| C | 5.539774 | 6.538234 | -0.933141 |
| C | 4.922706 | 7.006611 | -2.254057 |
| C | 7.860617 | 3.582556 | -1.803319 |
| C | -1.796867 | -6.931522 | -4.052665 |
| C | -2.396913 | -7.333361 | -5.245274 |
| C | -0.473894 | -8.847121 | -5.824670 |
| C | -2.608114 | -9.781678 | -4.919461 |
| C | -2.001999 | -8.687003 | -5.809031 |
| C | -2.491374 | -8.838877 | -7.253408 |
| C | -3.928591 | -8.378708 | -7.419915 |
| C | -5.599289 | -6.614358 | -6.961724 |
| C | -3.554791 | -6.041555 | -8.280239 |
| C | -4.094206 | -6.885410 | -7.116324 |
| C | -3.632694 | -5.260513 | -5.282050 |
| C | -3.030745 | -4.867089 | -4.113772 |
| C | -2.115809 | -5.716919 | -3.482479 |
| C | -1.691185 | -5.092622 | -2.239533 |
| C | -0.802843 | -5.519395 | -1.272590 |
| C | -0.588828 | -4.725336 | -0.145243 |
| C | -1.285626 | -3.523923 | -0.004835 |
| C | -2.174182 | -3.090742 | -0.979820 |
| C | -2.365983 | -3.870723 | -2.101347 |
| C | -2.560310 | -2.416379 | -4.089780 |
| C | -3.197562 | -3.586861 | -3.330288 |
| C | -4.653035 | -3.276020 | -2.999316 |
| C | -3.348671 | -6.506340 | -5.849101 |
| C | 1.996988 | 3.375588 | 3.624119 |
| C | 1.828108 | 4.643835 | 3.063794 |
| C | 0.791001 | 5.434120 | 3.561656 |
| C | -0.062323 | 4.969871 | 4.543140 |
| C | 0.116759 | 3.716019 | 5.085585 |

| | | | |
|---|---|---|---|
| C | 1.164166 | 2.913225 | 4.636283 |
| C | 2.127838 | -0.636012 | 6.862662 |
| C | 2.408455 | -1.770250 | 7.655616 |
| C | 2.223429 | -1.740565 | 9.010443 |
| C | 1.765583 | -0.575756 | 9.643835 |
| C | 1.468267 | 0.531928 | 8.902349 |
| C | 1.618661 | 0.521622 | 7.499554 |
| C | 1.312751 | 1.647819 | 6.719403 |
| C | 1.438151 | 1.656488 | 5.348195 |
| C | 1.916715 | 0.481424 | 4.717923 |
| C | 2.319115 | -0.610996 | 5.451320 |
| C | 1.141498 | -4.252061 | 1.578319 |
| C | 0.361537 | -5.170155 | 0.880946 |
| C | 0.488007 | -6.528200 | 1.164207 |
| C | 1.380063 | -6.957527 | 2.127384 |
| C | 2.126986 | -6.038816 | 2.838151 |
| C | 2.013216 | -4.671292 | 2.575192 |
| C | 2.800071 | -3.739908 | 3.398054 |
| C | 4.041669 | -4.135346 | 3.848833 |
| C | 4.820651 | -3.356265 | 4.720448 |
| C | 6.121063 | -3.745521 | 5.104141 |
| C | 6.860204 | -2.967144 | 5.948883 |
| C | 6.334661 | -1.759877 | 6.433100 |
| C | 5.074768 | -1.358544 | 6.084549 |
| C | 4.279484 | -2.151448 | 5.232100 |
| C | 2.977351 | -1.764839 | 4.810413 |
| C | 2.314120 | -2.487102 | 3.846297 |

P-Si

ONIOM(PBE-D3BJ/def2-SVP: GFN2-xTB, gas phase) G = -1707.298849

ONIOM(B2PLYP-D3BJ/def2-TZVP(ALPB:n-hexane):GFN2-xTB) G = -1708.942315

Number of imaginary frequencies: 0

Coordinates:

| | | | |
|---|---|---|---|
| C | -1.040859 | 0.937334 | -4.084242 |
| C | -1.476767 | 2.266554 | -4.542898 |
| O | -0.525076 | 3.153180 | -4.692229 |
| C | -0.867260 | 4.526791 | -4.990655 |
| O | -2.680278 | 2.650075 | -4.768403 |
| Si | -4.366922 | 2.014665 | -4.712282 |
| C | -4.708910 | 1.766524 | -6.583088 |
| C | -3.717689 | 0.730162 | -7.150017 |
| C | -6.154160 | 1.239375 | -6.726585 |
| C | -4.562464 | 3.101673 | -7.337959 |
| C | -4.550948 | 0.455840 | -3.719328 |
| C | -5.264038 | 3.471448 | -3.953088 |
| H | -1.884809 | 0.227869 | -4.112604 |
| H | -0.225560 | 0.578977 | -4.746045 |
| H | -1.472634 | 4.940917 | -4.161990 |
| H | -1.437711 | 4.577649 | -5.938089 |
| H | 0.102574 | 5.050946 | -5.070792 |
| H | -3.790155 | -0.246271 | -6.629497 |
| H | -2.665190 | 1.073073 | -7.074867 |
| H | -3.927826 | 0.548236 | -8.226185 |
| H | -6.901510 | 1.956804 | -6.329694 |
| H | -6.392269 | 1.074728 | -7.799790 |
| H | -6.299865 | 0.272528 | -6.203235 |
| H | -5.277963 | 3.864903 | -6.971418 |
| H | -4.759458 | 2.952324 | -8.421418 |
| H | -3.540055 | 3.519623 | -7.239530 |
| H | -4.164044 | -0.440547 | -4.237213 |
| H | -4.115151 | 0.491571 | -2.698308 |

| | | | |
|---|---|---|---|
| H | -5.641372 | 0.307203 | -3.575511 |
| H | -5.002572 | 4.430897 | -4.440261 |
| H | -5.015518 | 3.537280 | -2.874376 |
| H | -6.360325 | 3.322209 | -4.033451 |
| H | 1.831807 | -2.013246 | -0.706131 |
| H | 2.525442 | -4.573365 | -2.293699 |
| H | 3.173954 | -4.045311 | -0.702153 |
| H | 4.464869 | -2.224485 | -1.748441 |
| C | -0.386711 | 0.974345 | -2.637309 |
| C | -1.363743 | 1.329059 | -1.515708 |
| C | -2.026292 | 2.704650 | -1.567974 |
| C | 0.263597 | -0.349294 | -2.343670 |
| N | 1.282243 | -0.725350 | -3.065208 |
| N | 1.929608 | -1.844535 | -2.816288 |
| C | 1.739101 | -2.657936 | -1.612633 |
| C | 2.864667 | -3.694193 | -1.704832 |
| C | 3.975289 | -2.931829 | -2.448220 |
| C | 3.202281 | -2.116480 | -3.489778 |
| H | 4.742134 | -3.592953 | -2.896744 |
| H | 3.028890 | -2.696716 | -4.427127 |
| H | 3.697344 | -1.161348 | -3.747442 |
| H | 0.398570 | 1.757883 | -2.705199 |
| H | -2.152072 | 0.550671 | -1.425997 |
| H | -0.800728 | 1.229923 | -0.564715 |
| H | -2.832044 | 2.761388 | -2.323722 |
| H | -2.512204 | 2.912259 | -0.595303 |
| H | -1.294673 | 3.515171 | -1.770533 |
| H | -0.131804 | -0.933153 | -1.489799 |
| H | 0.722665 | -3.107104 | -1.605180 |
| N | -0.130922 | -0.182545 | 1.774976 |
| P | -1.426832 | 0.457563 | 2.436412 |

| | | | |
|---|---|---|---|
| N | -2.534958 | 0.855301 | 1.352640 |
| S | -3.712951 | -0.064897 | 0.918786 |
| O | -3.828227 | -0.224548 | -0.508790 |
| O | -3.999483 | -1.218403 | 1.710288 |
| C | -5.107474 | 1.156777 | 1.292118 |
| F | -6.259404 | 0.919740 | 0.654190 |
| F | -4.738228 | 2.396628 | 0.940433 |
| F | -5.376521 | 1.165889 | 2.599420 |
| O | -1.052495 | 1.825178 | 3.201168 |
| O | -2.089559 | -0.490707 | 3.547057 |
| F | 6.041240 | -1.421206 | 0.902183 |
| N | 2.269629 | -1.103300 | 1.313771 |
| P | 1.233809 | -0.657996 | 2.446276 |
| S | 3.538064 | -0.306136 | 0.884871 |
| O | 1.903159 | 0.444797 | 3.401224 |
| O | 0.969239 | -1.937600 | 3.388409 |
| O | 3.819097 | 0.923868 | 1.555858 |
| O | 3.738647 | -0.333294 | -0.535374 |
| C | 4.854685 | -1.511718 | 1.507800 |
| F | 5.064258 | -1.318774 | 2.813253 |
| F | 4.443709 | -2.774399 | 1.337237 |
| H | -2.905546 | -2.783030 | 3.779124 |
| C | -2.198872 | 2.534728 | 3.583409 |
| C | -2.945858 | 2.013661 | 4.616062 |
| C | -4.238360 | 2.532034 | 4.905878 |
| C | -5.113439 | 1.935666 | 5.836908 |
| C | -6.371506 | 2.435464 | 6.029313 |
| C | -6.817228 | 3.550726 | 5.304551 |
| C | -5.998635 | 4.138209 | 4.382455 |
| C | -4.697542 | 3.642270 | 4.155629 |
| C | -3.833464 | 4.233975 | 3.219972 |

| | | | |
|---|---|---|---|
| C | -2.584824 | 3.728322 | 2.925106 |
| C | -1.699776 | 4.524463 | 2.057117 |
| C | -1.788507 | 5.915213 | 2.170178 |
| C | -0.979261 | 6.737951 | 1.414397 |
| C | -0.073749 | 6.191103 | 0.527591 |
| C | 0.025945 | 4.808477 | 0.381115 |
| C | -0.782105 | 3.983706 | 1.164142 |
| H | -0.721249 | 2.912626 | 1.058366 |
| H | 0.497762 | 7.359739 | -6.962480 |
| H | -8.507542 | -2.527450 | 1.246535 |
| H | 0.329105 | -2.910241 | 6.553962 |
| H | 0.583903 | 6.840689 | -0.029146 |
| H | -1.045860 | 7.811142 | 1.527740 |
| H | -0.472699 | -6.292396 | 4.129621 |
| H | 0.858412 | -5.206912 | 5.837954 |
| H | -2.466128 | 6.356462 | 2.884714 |
| H | -4.189760 | 5.103406 | 2.688286 |
| H | -6.336162 | 4.986445 | 3.804164 |
| H | -7.814208 | 3.930781 | 5.472693 |
| H | -7.036428 | 1.964364 | 6.737909 |
| H | -4.790076 | 1.063328 | 6.382940 |
| C | -2.396167 | 0.929060 | 5.453810 |
| C | -2.030334 | -0.283064 | 4.913154 |
| C | -1.647591 | -1.371924 | 5.736205 |
| C | -1.600718 | -1.173485 | 7.096573 |
| C | -1.870978 | 0.073254 | 7.684178 |
| C | -1.774527 | 0.269134 | 9.077698 |
| C | -2.016167 | 1.495299 | 9.628986 |
| C | -2.358671 | 2.577491 | 8.805248 |
| C | -2.486445 | 2.411208 | 7.453701 |
| C | -2.263774 | 1.152428 | 6.854978 |

| | | | |
|---|---|---|---|
| H | -2.759281 | 3.251839 | 6.833929 |
| H | -2.526191 | 3.548858 | 9.247064 |
| H | -1.938608 | 1.641809 | 10.696255 |
| H | -1.496770 | -0.571190 | 9.696612 |
| H | -1.353919 | -2.000645 | 7.745434 |
| C | -1.325729 | -2.706150 | 5.206154 |
| C | -0.265705 | -3.392760 | 5.794742 |
| C | 0.028952 | -4.676762 | 5.391501 |
| C | -0.735810 | -5.290219 | 4.420421 |
| C | -1.803805 | -4.631530 | 3.808300 |
| C | -2.079561 | -3.323450 | 4.213624 |
| C | 3.747346 | 5.749236 | -6.397746 |
| C | 4.801780 | 2.418126 | -3.528730 |
| C | 3.381548 | 2.857121 | -3.863949 |
| C | 2.653745 | 1.758372 | -4.653927 |
| C | 2.589426 | 3.219687 | -2.627620 |
| C | 2.384402 | 2.496980 | -1.469836 |
| C | 1.570168 | 3.026543 | -0.476455 |
| C | 0.939312 | 4.261155 | -0.634033 |
| C | 1.165773 | 4.993040 | -1.802227 |
| C | 1.999879 | 4.482738 | -2.776274 |
| C | 2.455688 | 5.058548 | -4.031285 |
| C | 3.319404 | 4.145964 | -4.649231 |
| C | 3.953299 | 4.494888 | -5.814823 |
| C | 4.539285 | 6.080332 | -7.649816 |
| C | 3.993722 | 5.250277 | -8.820679 |
| C | 6.025155 | 5.742255 | -7.449862 |
| C | 4.450215 | 7.576708 | -7.970863 |
| C | 3.031657 | 8.103409 | -7.846289 |
| C | 2.502430 | 7.991587 | -6.412086 |
| C | 3.122505 | 9.082202 | -5.527789 |

| | | | |
|---|---|---|---|
| C | 0.980620 | 8.201305 | -6.468437 |
| C | 2.833653 | 6.633792 | -5.817954 |
| C | 2.207529 | 6.281853 | -4.621884 |
| H | 4.767377 | 1.564384 | -2.858930 |
| H | 5.338377 | 2.137496 | -4.433526 |
| H | 5.339715 | 3.223310 | -3.036959 |
| H | 2.462305 | 0.893701 | -4.021652 |
| H | 1.700934 | 2.136649 | -5.019833 |
| H | 3.254876 | 1.454811 | -5.508699 |
| H | 2.844989 | 1.533294 | -1.321651 |
| H | 1.432493 | 2.464865 | 0.435886 |
| H | 1.541888 | 6.978689 | -4.134617 |
| H | 0.680949 | 5.946363 | -1.955230 |
| H | 4.630778 | 3.795194 | -6.278262 |
| H | 4.566307 | 5.451204 | -9.723914 |
| H | 4.061767 | 4.188078 | -8.598828 |
| H | 2.949585 | 5.485322 | -9.012518 |
| H | 6.200544 | 4.670750 | -7.433773 |
| H | 6.609301 | 6.162643 | -8.266253 |
| H | 6.386900 | 6.161342 | -6.513504 |
| H | 4.819741 | 7.748980 | -8.984372 |
| H | 5.100829 | 8.125360 | -7.287783 |
| H | 2.376225 | 7.539971 | -8.513451 |
| H | 2.997270 | 9.148949 | -8.161106 |
| H | 2.894618 | 10.068656 | -5.926914 |
| H | 2.732189 | 9.019971 | -4.515255 |
| H | 4.201916 | 8.969302 | -5.473707 |
| H | 0.758551 | 9.104736 | -7.032967 |
| H | 0.548006 | 8.313547 | -5.478545 |
| C | -7.519170 | -4.650798 | -1.548595 |
| C | -4.281652 | -7.849825 | -1.346894 |

| | | | |
|---|---|---|---|
| C | -5.058562 | -7.269661 | -0.161878 |
| C | -6.006949 | -8.321138 | 0.422833 |
| C | -4.121166 | -6.728868 | 0.892032 |
| C | -3.261186 | -7.416673 | 1.716912 |
| C | -2.541423 | -6.716149 | 2.669252 |
| C | -2.616304 | -5.324309 | 2.789860 |
| C | -3.489402 | -4.632701 | 1.943539 |
| C | -4.251185 | -5.339764 | 1.028887 |
| C | -5.323492 | -4.902910 | 0.145720 |
| C | -5.810038 | -6.017818 | -0.548453 |
| C | -6.870004 | -5.880874 | -1.408515 |
| C | -8.692659 | -4.590525 | -2.511569 |
| C | -9.851657 | -5.423695 | -1.946099 |
| C | -8.288525 | -5.163024 | -3.879949 |
| C | -9.147115 | -3.143594 | -2.731814 |
| C | -9.202104 | -2.367121 | -1.427961 |
| C | -7.815566 | -2.226208 | -0.786665 |
| C | -6.991767 | -1.180863 | -1.547208 |
| C | -8.021602 | -1.750949 | 0.658525 |
| C | -7.083440 | -3.556264 | -0.795013 |
| C | -5.948676 | -3.680060 | 0.009944 |
| H | -3.700326 | -8.713546 | -1.031028 |
| H | -4.964591 | -8.159636 | -2.135147 |
| H | -3.605371 | -7.101863 | -1.751208 |
| H | -5.447521 | -9.187404 | 0.768675 |
| H | -6.555949 | -7.903281 | 1.263949 |
| H | -6.720635 | -8.648040 | -0.330198 |
| H | -3.161736 | -8.491271 | 1.650290 |
| H | -1.924970 | -7.286403 | 3.345231 |
| H | -5.561986 | -2.831727 | 0.551768 |
| H | -3.589924 | -3.559709 | 1.992055 |

| | | | |
|---|---|---|---|
| H | -7.221454 | -6.739836 | -1.960063 |
| H | -10.698592 | -5.407042 | -2.629930 |
| H | -9.547509 | -6.457162 | -1.801223 |
| H | -10.174093 | -5.034190 | -0.984048 |
| H | -8.137422 | -6.237801 | -3.840287 |
| H | -9.072619 | -4.963258 | -4.608457 |
| H | -7.365926 | -4.702220 | -4.225988 |
| H | -10.131456 | -3.144368 | -3.206897 |
| H | -8.453318 | -2.653300 | -3.417209 |
| H | -9.868028 | -2.878916 | -0.731119 |
| H | -9.617020 | -1.372064 | -1.605665 |
| H | -7.483240 | -0.211294 | -1.496744 |
| H | -6.000479 | -1.080358 | -1.114231 |
| H | -6.885091 | -1.471218 | -2.589739 |
| H | -8.649498 | -0.862286 | 0.666572 |
| H | -7.080190 | -1.498018 | 1.134381 |
| H | 7.077784 | 0.893021 | 1.104410 |
| H | 8.572547 | 0.052531 | 0.724325 |
| H | 6.890072 | 0.381831 | -2.602644 |
| H | 5.948272 | 0.293131 | -1.116145 |
| H | 7.347381 | -0.753290 | -1.329351 |
| H | 9.569451 | 0.196774 | -1.606136 |
| H | 9.970824 | 1.753207 | -0.888669 |
| H | 8.528696 | 1.391570 | -3.544265 |
| H | 10.248554 | 1.735820 | -3.377486 |
| H | 7.666375 | 3.445959 | -4.583020 |
| H | 9.393492 | 3.496418 | -4.957593 |
| H | 8.585038 | 4.929944 | -4.340772 |
| H | 10.459133 | 3.834061 | -1.335528 |
| H | 9.983069 | 5.218272 | -2.306785 |
| H | 11.036705 | 3.982772 | -2.998919 |

| | | | |
|---|---|---|---|
| H | 7.678430 | 5.694147 | -2.550755 |
| H | 3.710269 | 3.282605 | 1.615126 |
| H | 5.650920 | 2.234325 | 0.321903 |
| H | 2.261379 | 7.253458 | 2.418259 |
| H | 3.656982 | 8.150308 | 0.663766 |
| H | 7.298087 | 7.807464 | -1.148164 |
| H | 7.017313 | 7.269519 | 0.511284 |
| H | 6.035191 | 8.568505 | -0.175231 |
| H | 4.121045 | 6.346941 | -2.521682 |
| H | 5.570713 | 7.252356 | -2.959436 |
| H | 4.313393 | 8.020097 | -1.982846 |
| H | -0.164450 | -9.311501 | -4.597964 |
| H | -0.298866 | -10.184617 | -6.116448 |
| H | -3.814562 | -9.925148 | -4.717164 |
| H | -2.394735 | -9.891090 | -3.684760 |
| H | -2.489367 | -11.057763 | -5.007803 |
| H | -2.473875 | -10.322901 | -7.321844 |
| H | -1.840416 | -8.742165 | -7.768394 |
| H | -4.619699 | -9.252131 | -6.633960 |
| H | -4.257393 | -9.000000 | -8.338161 |
| H | -5.950398 | -7.243017 | -6.071114 |
| H | -6.085837 | -7.384885 | -7.826982 |
| H | -5.726614 | -5.828016 | -7.097261 |
| H | -2.394686 | -6.728045 | -8.437557 |
| H | -3.536474 | -5.416173 | -8.185976 |
| H | -3.970904 | -6.768428 | -9.235404 |
| H | -4.232935 | -4.861707 | -5.919638 |
| H | -0.345443 | -6.581623 | -1.339957 |
| H | -1.158159 | -7.812408 | -3.447814 |
| H | -1.220920 | -2.935975 | 0.742369 |
| H | -2.683102 | -2.212374 | -1.080524 |

| | | | |
|---|---|---|---|
| H | -2.891734 | -2.441121 | -5.251145 |
| H | -1.361337 | -2.965619 | -4.541448 |
| H | -2.324121 | -1.749185 | -3.707514 |
| H | -5.082350 | -4.158489 | -2.762433 |
| H | -5.072504 | -3.131163 | -4.201049 |
| H | -4.599450 | -2.470175 | -2.624355 |
| H | 0.995120 | 2.583353 | 7.294457 |
| H | 0.998315 | 1.460750 | 9.436735 |
| H | 1.446343 | -0.556166 | 10.787992 |
| H | 2.190651 | -2.632589 | 9.684362 |
| H | 2.554082 | -2.698523 | 7.272007 |
| H | 4.498658 | -0.509206 | 6.584633 |
| H | 6.725709 | -1.269757 | 7.246448 |
| H | 7.629344 | -3.399879 | 6.397736 |
| H | 6.303360 | -4.760273 | 4.824363 |
| H | 4.265194 | -5.105251 | 3.559970 |
| H | 2.561715 | -6.389783 | 3.737047 |
| H | -0.734657 | 5.711028 | 5.050794 |
| H | 0.797065 | 6.518443 | 3.367220 |
| H | 1.277044 | -8.035368 | 2.462880 |
| H | -0.280318 | -7.294172 | 0.712090 |
| H | -0.525334 | 3.424328 | 5.936424 |
| H | 8.599391 | 1.783195 | 1.090561 |
| H | -0.029518 | -8.438837 | -6.124251 |
| H | 0.954994 | -3.245344 | 1.330981 |
| H | 2.797307 | 2.725839 | 3.355661 |
| C | 6.118480 | 2.984733 | -0.295578 |
| C | 7.248697 | 2.677667 | -1.057880 |
| C | 8.034429 | 0.990239 | 0.603006 |
| C | 6.952755 | 0.240991 | -1.526422 |
| C | 7.862742 | 1.299266 | -0.891351 |

| | | | |
|---|---|---|---|
| C | 9.254991 | 1.240260 | -1.533629 |
| C | 9.270925 | 1.879735 | -2.910440 |
| C | 8.627135 | 3.846895 | -4.268290 |
| C | 10.187393 | 4.150899 | -2.338979 |
| C | 8.959740 | 3.378357 | -2.842313 |
| C | 7.245739 | 4.932230 | -1.919964 |
| C | 6.184698 | 5.248303 | -1.110078 |
| C | 5.594239 | 4.261667 | -0.309972 |
| C | 4.534081 | 4.878978 | 0.476814 |
| C | 3.687932 | 4.345571 | 1.434747 |
| C | 2.831559 | 5.192535 | 2.148201 |
| C | 2.864782 | 6.560509 | 1.854840 |
| C | 3.674572 | 7.087715 | 0.864019 |
| C | 4.513464 | 6.246867 | 0.170018 |
| C | 6.533956 | 7.627165 | -0.395250 |
| C | 5.524765 | 6.589931 | -0.897677 |
| C | 4.837537 | 7.085717 | -2.173062 |
| C | 7.787929 | 3.644039 | -1.913239 |
| C | -1.828661 | -7.173514 | -4.000481 |
| C | -2.415154 | -7.627897 | -5.181356 |
| C | -0.543526 | -9.250746 | -5.613734 |
| C | -2.733886 | -10.040952 | -4.706586 |
| C | -2.063643 | -9.029764 | -5.647672 |
| C | -2.525575 | -9.256132 | -7.091487 |
| C | -3.938238 | -8.750550 | -7.323332 |
| C | -5.544833 | -6.895945 | -7.018953 |
| C | -3.449278 | -6.491622 | -8.321199 |
| C | -4.048940 | -7.235450 | -7.119130 |
| C | -3.561874 | -5.513331 | -5.381415 |
| C | -2.974613 | -5.070424 | -4.223567 |
| C | -2.112165 | -5.914330 | -3.514729 |

| | | | |
|---|---|---|---|
| C | -1.705397 | -5.236759 | -2.293000 |
| C | -0.868340 | -5.639738 | -1.272437 |
| C | -0.676200 | -4.800623 | -0.174337 |
| C | -1.347612 | -3.577728 | -0.117602 |
| C | -2.175970 | -3.163185 | -1.151598 |
| C | -2.342269 | -3.989413 | -2.243084 |
| C | -2.377451 | -2.649188 | -4.313696 |
| C | -3.110426 | -3.740095 | -3.521054 |
| C | -4.561348 | -3.347404 | -3.263556 |
| C | -3.316780 | -6.803691 | -5.860790 |
| C | 2.024525 | 3.390113 | 3.708640 |
| C | 1.918169 | 4.685106 | 3.192369 |
| C | 0.921053 | 5.508934 | 3.719023 |
| C | 0.042438 | 5.056293 | 4.682539 |
| C | 0.161679 | 3.779313 | 5.183954 |
| C | 1.173256 | 2.942625 | 4.713801 |
| C | 1.973634 | -0.646588 | 6.937651 |
| C | 2.211078 | -1.786733 | 7.736507 |
| C | 2.011713 | -1.749101 | 9.089198 |
| C | 1.581218 | -0.570849 | 9.716522 |
| C | 1.334678 | 0.546392 | 8.970891 |
| C | 1.506234 | 0.529986 | 7.570831 |
| C | 1.264723 | 1.669063 | 6.787370 |
| C | 1.386749 | 1.667394 | 5.415998 |
| C | 1.796967 | 0.466201 | 4.783626 |
| C | 2.163770 | -0.635537 | 5.525306 |
| C | 0.996392 | -4.288470 | 1.601145 |
| C | 0.230924 | -5.218527 | 0.901175 |
| C | 0.336765 | -6.570346 | 1.221672 |
| C | 1.198567 | -6.985447 | 2.217001 |
| C | 1.936468 | -6.056599 | 2.922798 |

| | | | |
|---|---|---|---|
| C | 1.840121 | -4.694007 | 2.627405 |
| C | 2.620359 | -3.763446 | 3.457711 |
| C | 3.848198 | -4.179518 | 3.926700 |
| C | 4.619879 | -3.425488 | 4.825108 |
| C | 5.907296 | -3.839227 | 5.227377 |
| C | 6.642201 | -3.082757 | 6.095149 |
| C | 6.123913 | -1.874521 | 6.584328 |
| C | 4.875351 | -1.452136 | 6.220278 |
| C | 4.081851 | -2.223741 | 5.346410 |
| C | 2.796607 | -1.809801 | 4.895853 |
| C | 2.139752 | -2.508084 | 3.908018 |

# References


1. Peyrical, L. C. *et al.* Rhodium-Catalyzed Intramolecular Cyclopropanation of Trifluoromethyl- and Pentafluorosulfanyl-Substituted Allylic Cyanodiazoacetates. *Org. Lett.* **25,** 2487–2491; 10.1021/acs.orglett.3c00642 (2023).

2. Ma, Y.-R., Lv, X.-J., Dong, Q., Ming, Y.-C. & Liu, Y.-K. Brønsted-Acid-Catalyzed In Situ Formation of Acyclic Tertiary Enamides and Its Application to the Preparation of Diverse Nitrogen-Containing Heterocyclic Compounds. *Org. Lett.* **25,** 5929–5934; 10.1021/acs.orglett.3c01919 (2023).

3. Ilisson, M., Tomson, K., Selyutina, A., Türk, S. & Mäeorg, U. Synthesis of Novel Saccharide Hydrazones. *Synth. Commun.* **45,** 1367–1373; 10.1080/00397911.2015.1021425 (2015).

4. Das, S. *et al.* Harnessing the ambiphilicity of silyl nitronates in a catalytic asymmetric approach to aliphatic β3-amino acids. *Nat. Catal.* **4,** 1043–1049; 10.1038/s41929-021-00714-x (2021).

5. Zhou, H. *et al.* Organocatalytic stereoselective cyanosilylation of small ketones. *Nature* **605,** 84–89; 10.1038/s41586-022-04531-5 (2022).

6. Uyanik, M., Suzuki, D., Yasui, T. & Ishihara, K. In situ generated (hypo)iodite catalysts for the direct α-oxyacylation of carbonyl compounds with carboxylic acids. *Angew. Chem. Int. Ed.* **50,** 5331–5334; 10.1002/anie.201101522 (2011).

7. Maaßen, R., Runsink, J. & Enders, D. First asymmetric nucleophilic displacement reactions of chiral α-substituted aldehyde hydrazones. *Tetrahedron: Asymmetry* **9,** 2155–2180 (1998).

8. Enders, D., Papadopoulus K. & Rendenbach B. Asymmetric Michael Additions via SAMP-/RAMP-Hydrazones Anti-Diastereo- and Enantioselective Synthesis of 3,4-Disubstituted 5-Oxo-Alkanoates. *Tetrahedron Let.* **27,** 3491–3494 (1986).

9. Niemeier, O. & Enders, D. Asymmetric Synthesis of β-substituted γ-Lactams employing the SAMP/RAMP-hydrazone Methodology. *Heterocycles* **66,** 385–403 (2005).

10. Burell, A. J. M. & Martinez, C. A. Process for the Preparation of (S)-3-Cyano-5-Methylhexanoic acid Derivatives and of Pregabalin, WO2012/025861A1 (2011).

11. Emimmal, M. E. S. & Sankar, V. Synthesis and Pancreatic Lipase Inhibitory Activity of Phenacyl Esters of N-Aroyl Amino Acids. *Cur. Enz. Inh.* **15,** 133–143; 10.2174/1573408015666190920092114 (2019).

12. Scharf, M. J. & List, B. A Catalytic Asymmetric Pictet-Spengler Platform as a Biomimetic Diversification Strategy toward Naturally Occurring Alkaloids. *J. Am. Chem. Soc.* **144,** 15451–15456; 10.1021/jacs.2c06664 (2022).

13. Grossmann, O., Maji, R., Aukland, M. H., Lee, S. & List, B. Catalytic Asymmetric Additions of Enol Silanes to In Situ Generated Cyclic, Aliphatic N-Acyliminium Ions. *Angew. Chem. Int. Ed.* **61,** e202115036; 10.1002/anie.202115036 (2022).

14. Burés, J. Variable Time Normalization Analysis: General Graphical Elucidation of Reaction Orders from Concentration Profiles. *Angew. Chem. Int. Ed.* **55,** 16084–16087; 10.1002/anie.201609757 (2016).

15. Venu, N., Vishweshwar, P., Ram, T., Surya, D. & Apurba, B. (S)-3-(Ammoniomethyl)-5-methylhexanoate (pregabalin). *Cryst. Struct. Commun.* **63,** o306-8; 10.1107/S0108270107016952 (2007).

16. Komisarek, D., Haj Hassani Sohi, T. & Vasylyeva, V. Co-crystals of zwitterionic GABA API's pregabalin and phenibut: properties and application. *CrystEngComm* **24,** 8390–8398; 10.1039/D2CE01416E (2022).

17. Neese, F. The ORCA program system. *WIREs Comput. Mol. Sci.* **2,** 73–78; 10.1002/wcms.81 (2012).

18. Bannwarth, C., Ehlert, S. & Grimme, S. GFN2-xTB—An Accurate and Broadly Parametrized Self-Consistent Tight-Binding Quantum Chemical Method with Multipole Electrostatics and Density-Dependent Dispersion Contributions. *J. Chem. Theory Comput.* **15,** 1652–1671; 10.1021/acs.jctc.8b01176 (2019).

19. Bannwarth, C. *et al.* Extended tight‐binding quantum chemistry methods. *WIREs Comput. Mol. Sci.* **11**; 10.1002/wcms.1493 (2021).

20. Yepes, D., Neese, F., List, B. & Bistoni, G. Unveiling the Delicate Balance of Steric and Dispersion Interactions in Organocatalysis Using High-Level Computational Methods. *J. Am. Chem. Soc.* **142,** 3613–3625; 10.1021/jacs.9b13725 (2020).

21. Zhu, C., Mandrelli, F., Zhou, H., Maji, R. & List, B. Catalytic Asymmetric Synthesis of Unprotected β 2 -Amino Acids. *J. Am. Chem. Soc.* **143,** 3312–3317; 10.1021/jacs.1c00249 (2021).

22. Zhang, Y. & Yang, W. Comment on "Generalized Gradient Approximation Made Simple". *Phys. Rev. Lett.* **80,** 890; 10.1103/PhysRevLett.80.890 (1998).

23. Grimme, S., Ehrlich, S. & Goerigk, L. Effect of the damping function in dispersion corrected density functional theory. *J. Comput. Chem.* **32,** 1456–1465; 10.1002/jcc.21759 (2011).

24. Grimme, S., Antony, J., Ehrlich, S. & Krieg, H. A consistent and accurate ab initio parametrization of density functional dispersion correction (DFT-D) for the 94 elements H-Pu. *J. Chem. Phys.* **132**; 10.1063/1.3382344 (2010).

25. Weigend, F. & Ahlrichs, R. Balanced basis sets of split valence, triple zeta valence and quadruple zeta valence quality for H to Rn: Design and assessment of accuracy. *PCCP* **7,** 3297; 10.1039/b508541a (2005).

26. Ásgeirsson, V. *et al.* Nudged Elastic Band Method for Molecular Reactions Using Energy-Weighted Springs Combined with Eigenvector Following. *J. Chem. Theory Comput.* **17,** 4929–4945; 10.1021/acs.jctc.1c00462 (2021).

27. Ehlert, S., Stahn, M., Spicher, S. & Grimme, S. Robust and Efficient Implicit Solvation Model for Fast Semiempirical Methods. *J. Chem. Theory Comput.* **17,** 4250–4261; 10.1021/acs.jctc.1c00471 (2021).

28. Grimme, S. Semiempirical hybrid density functional with perturbative second-order correlation. *J. Chem. Phys.* **124**; 10.1063/1.2148954 (2006).

29. CYLview & Legault, C. Y. Université de Sherbrooke, (http://www.cylview.org) (2020).
